# The adult human testis transcriptional cell atlas

**DOI:** 10.1038/s41422-018-0099-2

**Published:** 2018-10-12

**Authors:** Jingtao Guo, Edward J. Grow, Hana Mlcochova, Geoffrey J. Maher, Cecilia Lindskog, Xichen Nie, Yixuan Guo, Yodai Takei, Jina Yun, Long Cai, Robin Kim, Douglas T. Carrell, Anne Goriely, James M. Hotaling, Bradley R. Cairns

**Affiliations:** 1 Department of Oncological Sciences and Huntsman Cancer Institute, Howard Hughes Medical Institute, University of Utah School of Medicine, Salt Lake City, UT 84112 USA; 2 Department of Surgery (Andrology/Urology), Center for Reconstructive Urology and Men’s Health, University of Utah Health Sciences Center, Salt Lake City, UT 84122 USA; 3 Radcliffe Department of Medicine, MRC Weatherall Institute of Molecular Medicine, University of Oxford, Oxford, OX39DS UK; 4 Department of Immunology, Genetics and Pathology, Science for Life Laboratory, Uppsala University, SE-751 85 Uppsala, Sweden; 5 Division of Biology and Biological Engineering, California Institute of Technology, Pasadena, CA 91125 USA; 6 Section of Transplantation, Department of Surgery, University of Utah School of Medicine, Salt Lake City, UT 84132 USA

## Abstract

Human adult spermatogenesis balances spermatogonial stem cell (SSC) self-renewal and differentiation, alongside complex germ cell-niche interactions, to ensure long-term fertility and faithful genome propagation. Here, we performed single-cell RNA sequencing of ~6500 testicular cells from young adults. We found five niche/somatic cell types (Leydig, myoid, Sertoli, endothelial, macrophage), and observed germline-niche interactions and key human-mouse differences. Spermatogenesis, including meiosis, was reconstructed computationally, revealing sequential coding, non-coding, and repeat-element transcriptional signatures. Interestingly, we identified five discrete transcriptional/developmental spermatogonial states, including a novel early SSC state, termed State 0. Epigenetic features and nascent transcription analyses suggested developmental plasticity within spermatogonial States. To understand the origin of State 0, we profiled testicular cells from infants, and identified distinct similarities between adult State 0 and infant SSCs. Overall, our datasets describe key transcriptional and epigenetic signatures of the normal adult human testis, and provide new insights into germ cell developmental transitions and plasticity.

## Introduction

Human spermatogenesis involves the differentiation of adult spermatogonial stem cells (SSCs) into mature sperm through a complex developmental process, regulated by the testis niche. Human SSCs must carefully balance their self-renewal and differentiation, and then undergo niche-guided transitions between multiple cell states and cellular processes—including a commitment to mitosis, meiosis, and the subsequent stages of sperm maturation, which are accompanied by chromatin repackaging and major morphological changes.^1,2^ Through a wide range of approaches, considerable progress in understanding gametogenesis and germline-niche communication has been achieved in mice.^3,4^ In contrast, in humans, although adult testis physiology is well described,^5–7^ much less is known about SSCs and their regulation. Ultimately, a full understanding will require the integration of molecular, genomic, proteomic and physiological approaches.

Toward this goal, single cell RNA-seq (scRNA-seq) approaches can effectively delineate cell types, uncover heterogeneity, and infer developmental trajectories.^8^ These approaches have recently been applied to human fetal germ cells, providing important new biological insights.^9^ Single-cell approaches are well suited for addressing fundamental questions about SSCs, differentiating spermatogonia and gametogenesis. For example, what are the main molecular features that enable SSCs to serve as the long-term adult germline stem cells? How do SSCs transition from their initial, most naïve and quiescent states to spermatogonia that will eventually commit to meiosis? Are these transitions irreversible, or do spermatogonia possess bidirectional plasticity that helps ensure a lifelong pool of SSCs? Beyond spermatogonia, what are the subsequent sequential transcription and signaling programs that accompany gametogenesis? How are these processes influenced by communication with niche cells—what are the specific signaling and transcription pathways that regulate self-renewal, proliferation rates, metabolism, and transitions between differentiation states? Importantly, these questions overlap conceptually with other stem cell systems. Here, we aim to utilize single-cell transcriptome analysis from the full repertoire of germline and niche cells to address these questions.

Prior scRNA-seq efforts characterizing spermatogonia enriched via cell surface markers have provided initial insights into human spermatogenesis.^10^ However, thanks to new technological advances, it is now possible to use unbiased approaches to assess germline and niche cell transcriptional profiles. Here, we performed extensive scRNA-seq characterization of unselected human testicular cells of young adults using the 10× Genomics Chromium platform—yielding a transcriptional cell atlas of all cell types in the testis, including germline and niche cells. We delineate five distinct spermatogonial states in adults, including a novel early SSC state, termed State 0, which displays high similarity to infant SSCs. We further describe the genic and non-coding RNA expression programs that accompany spermatogenesis. Intriguingly, combining RNA ‘velocity’ analyses^11^ with chromatin mapping and DNA methylation (DNAme), we provide computational and molecular evidence that human spermatogonia possess considerable transcriptional/state plasticity, suggesting a conceptual framework for human spermatogonial homeostasis, similar to that described in other stem cell systems.

## Results

### Cell partitioning through the analysis of single cell transcriptomes

**Fig. 1:**
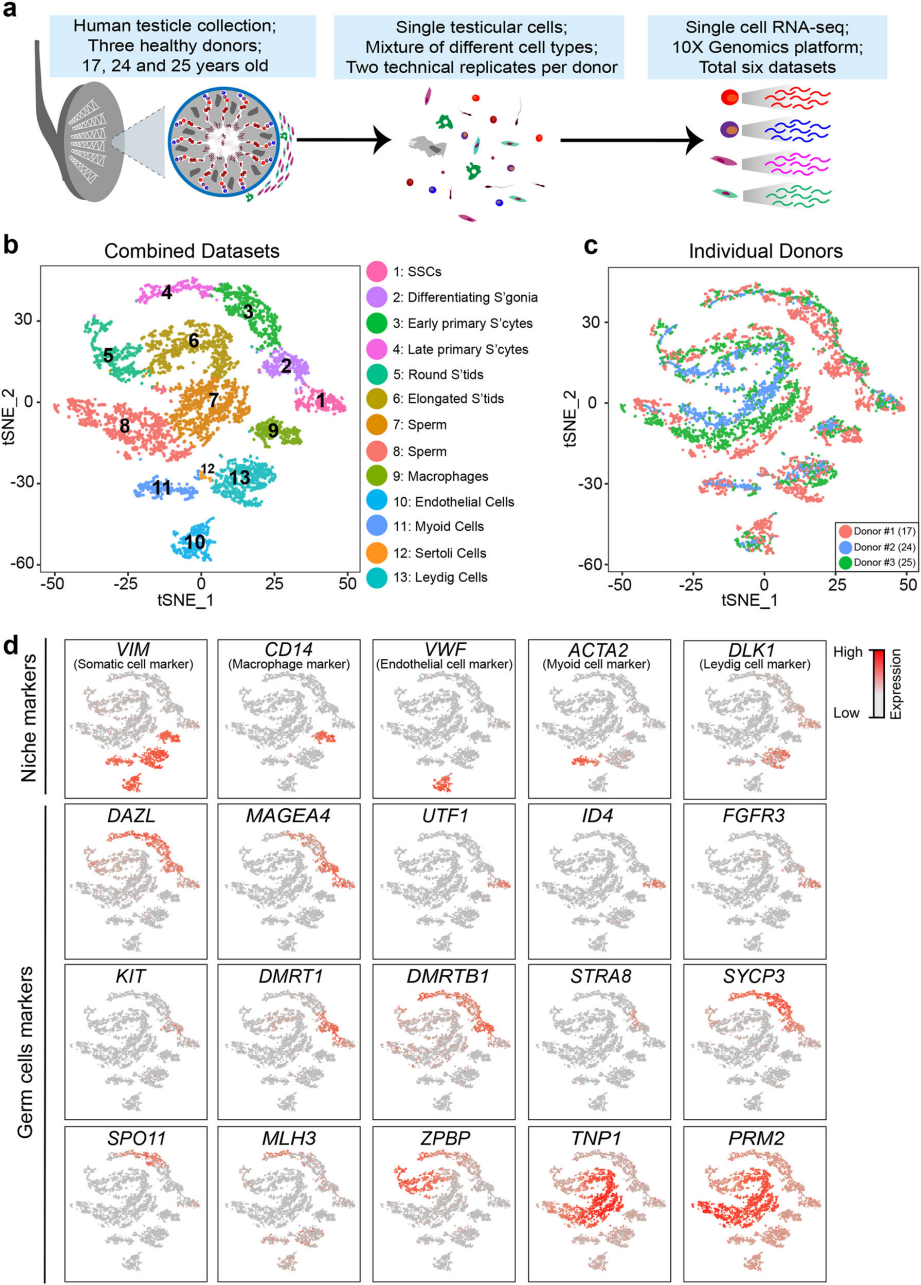
Single cell transcriptome profiling from healthy adult whole testes. **a** Schematic illustration of the experimental workflow. **b** tSNE and clustering analysis of combined single-cell transcriptome data from human testes (*n* = 6490). Each dot represents a single cell and is colored according to its cluster identity as indicated on the figure key. The 13 cluster identities were assigned based on marker gene expression shown in Fig. 1d and Supplementary information, Fig. S2. tSNE: t-distributed stochastic neighbor embedding. Note: the 40 µm filtering step likely limits capture of the large Sertoli cells. **c** tSNE plot of single cell transcriptome data with cells colored based on their donors of origin, as indicated on the figure key. **d** Expression patterns of selected markers projected on the tSNE plot. Red indicates high expression and gray indicates low or no expression, as shown on the figure key. Top row represents somatic/niche cell markers; bottom three rows are representative germ cell markers. For each cell type, we show one marker in the main figures and a gallery in supplementary information, Fig. S2

We isolated single cells from whole-testis of 3 individuals using a standard two-step procedure of enzymatic digestion and physical filtering.^7,10^ For each donor, two separate technical replicates were performed (Fig. 1a), resulting in six datasets. From a total of ~7000 cells, 6,490 passed standard quality control (QC) dataset filters and were retained for downstream analysis. We obtained ~250 K reads/cell which enabled the analysis of ~2500 genes/cell. The sequencing saturation rate was >83%, and technical replicates were highly similar (*r* > 0.96; Supplementary information, Fig. S1a).

Cell partitioning via t-distributed stochastic neighbor embedding (tSNE) analyses^12^ identified 13 clusters (Fig. 1b; Supplementary information, Table S1), with only minor variation based on batch/experiment or donor origin (Fig. 1c; Supplementary information, Fig. S1b and c). Cluster identity was assigned based on known cell-type marker expression (Fig. 1d; Supplementary information, Fig. S2). Clusters 9–13 correspond to macrophage, endothelial, myoid, Sertoli and Leydig cells, respectively (Fig. 1d; Supplementary information, Fig. S2a). Germline-specific markers were expressed solely in Clusters 1–8 (e.g., *DAZL* and *MAGEA4*; Fig. 1d; Supplementary information, Fig. S2b). Moreover, known SSC markers (e.g., *UTF1*, *ID4* and *FGFR3*), differentiating markers (e.g., *KIT* and *DMRT1*), meiosis markers (e.g., *SYCP3*, *SPO11*, and *MLH3*), spermatid structure proteins (e.g., *SPAG6*, *ZPBP*, *CAMK4* and *CREM*) and nuclear condensation/protamine repackaging factors (e.g., *TNP1* and *PRM2*) showed sequential expression peaks in Clusters 1 to 8, respectively—mirroring the temporal order of gametogenesis.

### Human-mouse comparisons in intra-niche and niche-germline interaction

**Fig. 2:**
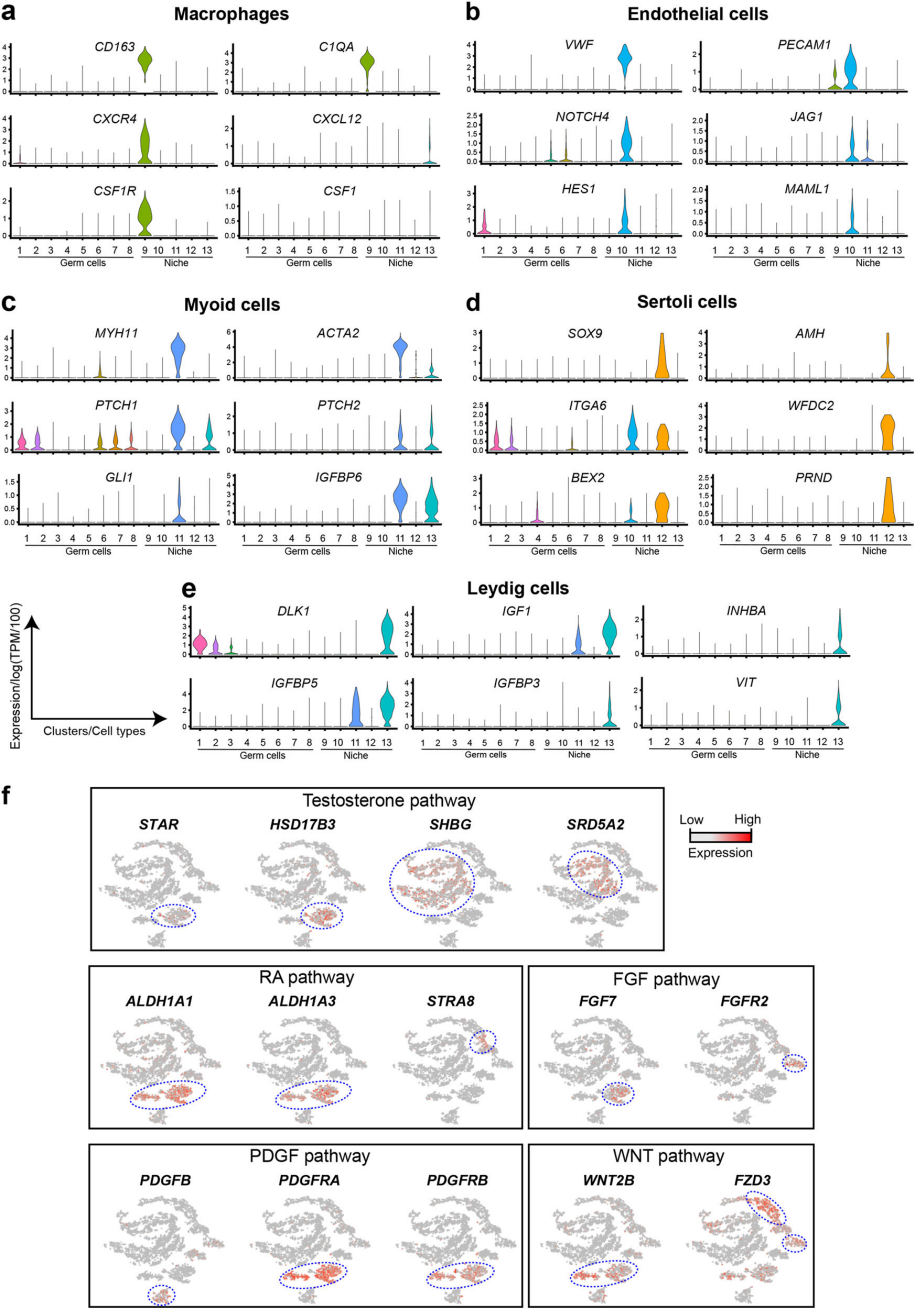
Expression patterns of representative genes marking niche cells, and Niche-Germline interactions. **a** Expression patterns (violin plot) of macrophage-specific genes across the 13 different Clusters (Clusters 1–8 = germ cells; Clusters 9–13 = Niche/somatic cells). **b** Expression patterns (violin plot) of endothelial cell-specific genes across the different clusters. **c** Expression patterns (violin plot) of myoid cell-specific genes across the different clusters. **d** Expression patterns (violin plot) of Sertoli cell specific genes across different clusters. **e** Expression patterns (violin plot) of Leydig cell specific genes across different clusters. **f** Relative expression levels of representative genes from different key signaling pathways projected onto the tSNE plot from Fig. 1b. Stage-specific expression is highlighted by blue dotted circles

We began by describing the niche cell datasets (Fig. 2). Testicular macrophages (Cluster 9) promote spermatogonia maintenance,^13,14^ and were identified by multiple specific markers (i.e., *CD14*, *CD163*, *C1QA*; Fig. 1d and 2a). Previous work showed that mouse Sertoli cells help maintain CXCR4^+^ spermatogonia population by secreting CXCL12, the ligand for CXCR4.^15^ Interestingly, in humans, RNA encoding CXCL12 was observed in Leydig cells, while the *CXCR4* receptor was expressed in both macrophages and spermatogonia (Fig. 2a), suggesting that CXCL12-CXCR4 promotes co-localization of macrophages and spermatogonia in humans. Furthermore, *CSF1R*, the receptor for *CSF1*, was specifically expressed in macrophages, whereas in mice its expression is confined to spermatogonia.^16^

In endothelial cells (Cluster 10, marked by *VWF* and *PECAM1*), the receptor (*NOTCH4*) and the downstream signaling factors (*JAG1*, *HES1* and *MAML1*) for NOTCH signaling were specifically up-regulated (Fig. 2b). Hedgehog signaling is important for mouse fetal myoid and Leydig cell development;^9,17^ but the receptors (*PTCH1*, *PTCH2*), and downstream signaling components (*GLI* and *IGFBP6*) of the Hedgehog pathway were highly expressed in human adult myoid (Cluster 11, marked by *MYH11* and *ACTA2*) and Leydig cells (Fig. 2c), indicating that both Hedgehog and NOTCH signaling activity persists through adulthood in human testes. Sertoli cells (Cluster 12, marked by *SOX9* and *AMH*) express *ITGA6*, an integrin found in the basal membrane of seminiferous tubules in humans (Fig. 2d). Notably, Sertoli cells express *WFDC2*, which is known to encode an epididymis protein that may promote sperm maturation,^18^ and *PRND*, which encodes a glycosylphosphatidylinositol-anchored glycoprotein with a putative interaction role with receptors from germ cells.^19^

Leydig cells (Cluster 13, marked by *DLK1* and *IGF1*) also expressed specific genes (Fig. 2e), including those encoding IGF binding proteins (*IGFBP5* and *IGFBP3*) and INHBA, a subunit of both inhibin and activin, and the extracellular matrix protein VIT. Interestingly, key genes for testosterone biosynthesis, *STAR* and *HSD17B3*, were expressed in both Sertoli and Leydig cells, while the expression of the responsive genes, *SHBG* and *SRD5A2*, were observed in maturing sperm (Fig. 2f). Retinoic acid (RA) induces germ cell differentiation, and enzymes for RA synthesis, *ALDH1A1* and *ALDH1A3*, were specifically expressed in Leydig and myoid cells; while *STRA8*, an RA target gene, was only observed during the transition of spermatogonia to spermatocytes (Fig. 2f). Interestingly, we found the WNT ligand, WNT2B, was expressed primarily in myoid cells, while WNT2B receptors were confined to primary spermatocytes, suggesting a role for *WNT2B* in human meiosis (Fig. 2f). *PDGFB* was expressed in endothelial cells, and its receptors *PDGFRA* and *PDGFRB* were found in Leydig and myoid cells, indicating that endothelial cells may indirectly affect germ cell development, via cross-talk mechanisms with other niche cells. Taken together, our data highlight both similarities and notable differences in germline-niche interactions in humans and mice that warrant further detailed functional investigations.

### Pseudotime and clustering analyses reveal dynamic gene expression patterns during spermatogenesis

**Fig. 3:**
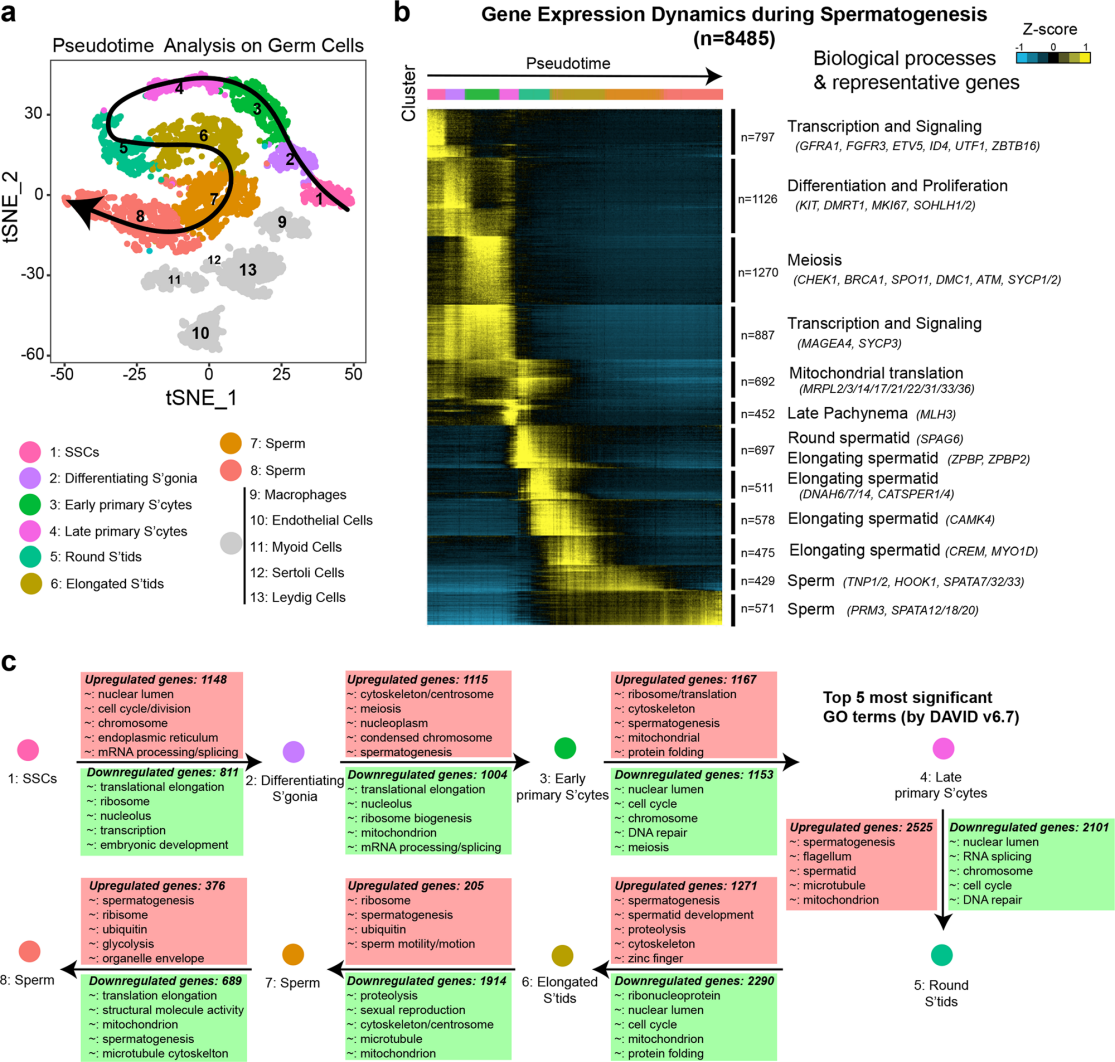
Gene expression dynamics during spermatogenesis. **a** Pseudotime analysis on germ cells (Clusters 1–8). Cluster 1 represents the start of pseudotime, with Cluster 8 at the end. **b** K-means clustering of genes exhibiting differential expression (*n* = 8485) across germ cell populations. Note: each row represents a gene, and each column represents a single cell, with columns/cells placed in pseudotime order as defined in Fig. 3a and depicted by a thick colored line (top, color code as in Fig. 3a). Differential gene expression levels utilize a *Z* score, which represents the variance from the mean, as defined on the color key in the right top corner. **c** Differentially-expressed genes and associated GO terms (using DAVID v6.7) characteristic of germ cell developmental transitions, based on the 8 germ cell Clusters represented in Fig. 2a. The 5 most significant up-regulated GO terms are annotated in pink boxes, and down-regulated GO terms in green boxes. GO: gene ontology

Noticeably, the germ cell clusters (1–8) formed a wave-like continuum, sometimes separated by distinct bottlenecks, that recapitulated the temporal order of spermatogenesis. Pseudotime analysis^20^ provided an arrow vector which aligned with the developmental order of gametogenesis (Fig. 3a). Clustering analysis of genes (rows) while fixing the order of cells (columns) along pseudotime, revealed 12 distinct gene cohorts. Gene ontology (GO) analysis of these clusters yielded a dynamic developmental, cellular and metabolic sequence of events, consistent with well-organized germline development (Fig. 3b; Supplementary information, Table S2). Next, differential analysis (bimodal test; adjusted *p*-value < 0.01; |logFC| > 0.25) identified differentially-expressed genes (Fig. 3c; Supplementary information, Table S3). As expected, ‘cell cycle’, ‘meiosis’ and ‘spermatogenesis’ were significantly-enriched GO terms during spermatogenic progression. Interestingly, >4600 genes were differentially expressed (2525 up and 2101 down) during the transition from spermatocytes to round spermatids, displaying the most dramatic transcriptomic change.

### Germline expression dynamics of transposable elements, lncRNAs and *XIST*

Single-cell datasets allow a refined examination of transposable elements (TE) and long non-coding RNAs (lncRNAs) (Supplementary information, Figs. S3a–c and S4a–b). Pseudotime and clustering analyses identified dynamic TE and lncRNA programs during spermatogenesis. Notably, LTR12C/D/E and the active TEs SVA_D and AluYa5 showed high expression during early spermatogenic stages. By contrast, LTR10A and LTR40c expression peaks during late spermatogonial or post-meiotic stages; and satellite and multiple MLT-family TEs at spermatid and sperm stages (Supplementary information, Fig. S3d). Moreover, we explored X chromosome inactivation and meiotic sex chromosome inactivation (MSCI) during spermatogenesis.^21,22^ Notably, we observed *XIST* expression during spermatogonial stages (Supplementary information, Fig. S4c and d), and unexpectedly selective attenuation of genes that are near the X-inactivation center during spermatogonial stages (Supplementary information, Fig. S4e and f), suggesting a role for *XIST*-mediated silencing in this process. Overall, our datasets and analyses provide a comprehensive resource to study TEs and lncRNA expression dynamics during male germline development.

### Analysis of meiotic cells reveals dynamic transcriptional programs and key factors during meiotic transition

**Fig. 4:**
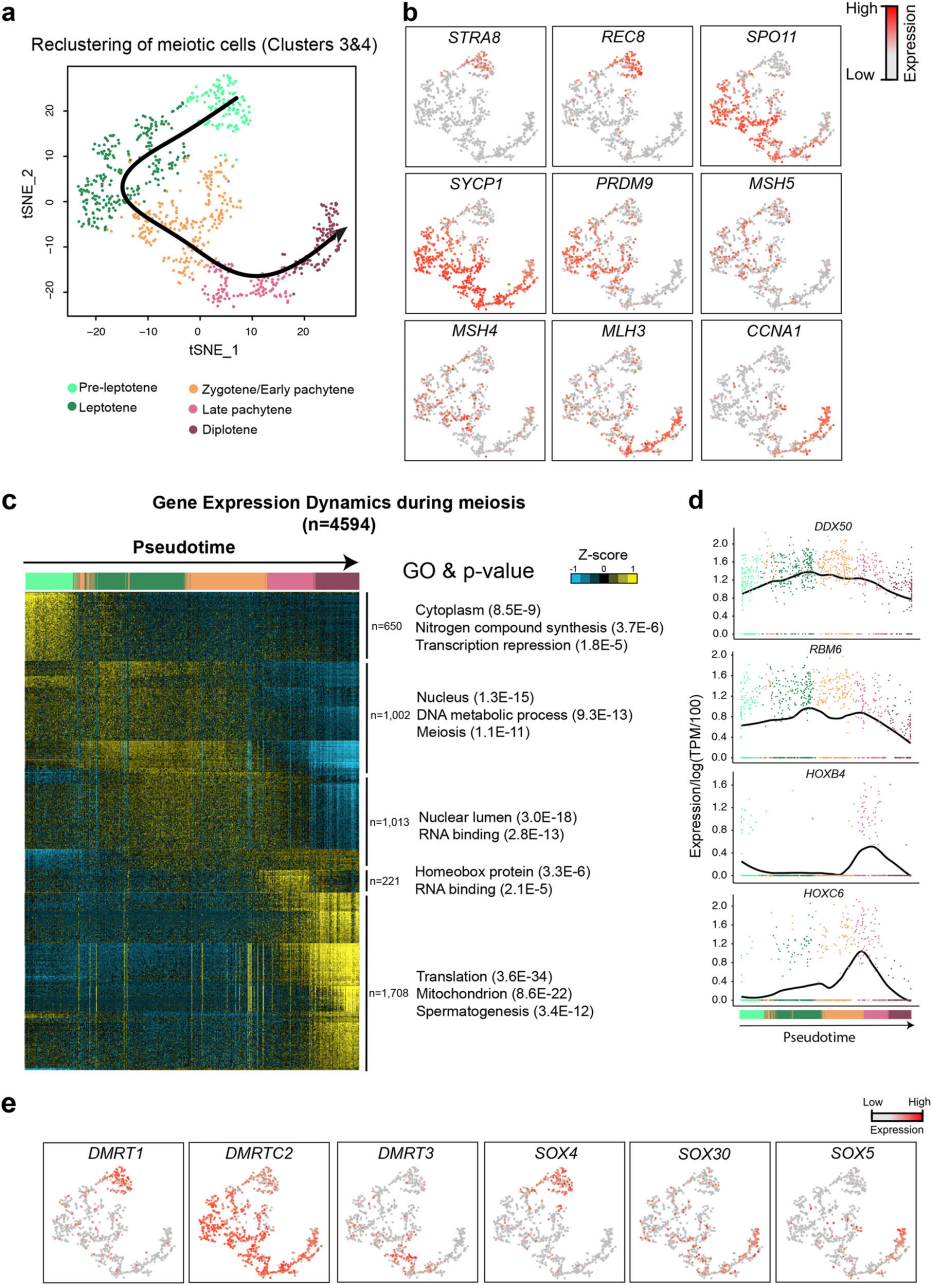
Gene Expression Dynamics during Meiosis. **a** Focused analysis (tSNE, clustering and pseudotime ordering) of the cells from Clusters 3 and 4 (from Figs. 1b and 3a) reveals developmental progression during meiosis I. **b** Expression patterns of known meiotic markers projected onto the tSNE plot. Red indicates high expression and gray indicates low or no expression, key on figure. **c** K-means clustering of genes exhibiting differential expression (*n* = 4594) during meiosis I. Note: each row represents a gene, and each column represents a single cell, with columns/cells placed in pseudotime order as defined in **a** and depicted by a thick colored line (top, color code as in **a**). Gene expression levels utilize a Z score, which depicts variance from the mean, as defined on the color key in the right top corner. **d** Expression levels of representative genes during meiosis progression. x-axis represents pseudotime (as defined on **a**), and *y*-axis represents gene expression levels. **e** Expression patterns of key transcription factors during meiosis, with their expression projected onto the tSNE plot (**a**)

Next, we singled out Clusters 3–4 (Fig. 1b) and performed re-clustering of the meiotic cells, which revealed five sub-clusters (Fig. 4a). Using known markers^23,24^ (Fig. 4b), we assigned pre-leptotene, leptotene, zygotene/early pachytene, late pachytene, and diplotene cell types, consistent with the pseudotime developmental order. Secondary spermatocytes were under-represented consistent with their rapid progression into round spermatids.^25^ Gene clustering analysis (Fig. 4c) identified five distinct molecular signatures (4594 genes) suggesting striking transcriptional changes during meiotic entry and exit, but only gradual changes during meiosis. Notably, several RNA binding proteins were upregulated at zygotene/early pachytene, and some HOX genes (e.g., *HOXB4* and *HOXC6*) showed late pachytene-specific expression (Fig. 4c, d).

We observed a dynamic expression pattern of DMRT and SOX family members (Fig. 4e): Consistent with their role in meiotic entry inhibition in mice,^26,27^ *DMRT1* and *SOX4* were only expressed in pre-leptotene cells. Although the function of *DMRTC2* and *DMRT3* is largely unknown, *Sox30* knockout causes murine germ cell development arrest at round spermatid stage, and reduces expression of *Sox5.*^28,29^ Overall, our data are consistent with findings in mice, but also provide evidence for candidate genes with human-specific functions during meiosis.

### Identification of known and novel spermatogonial stem cell states

**Fig. 5:**
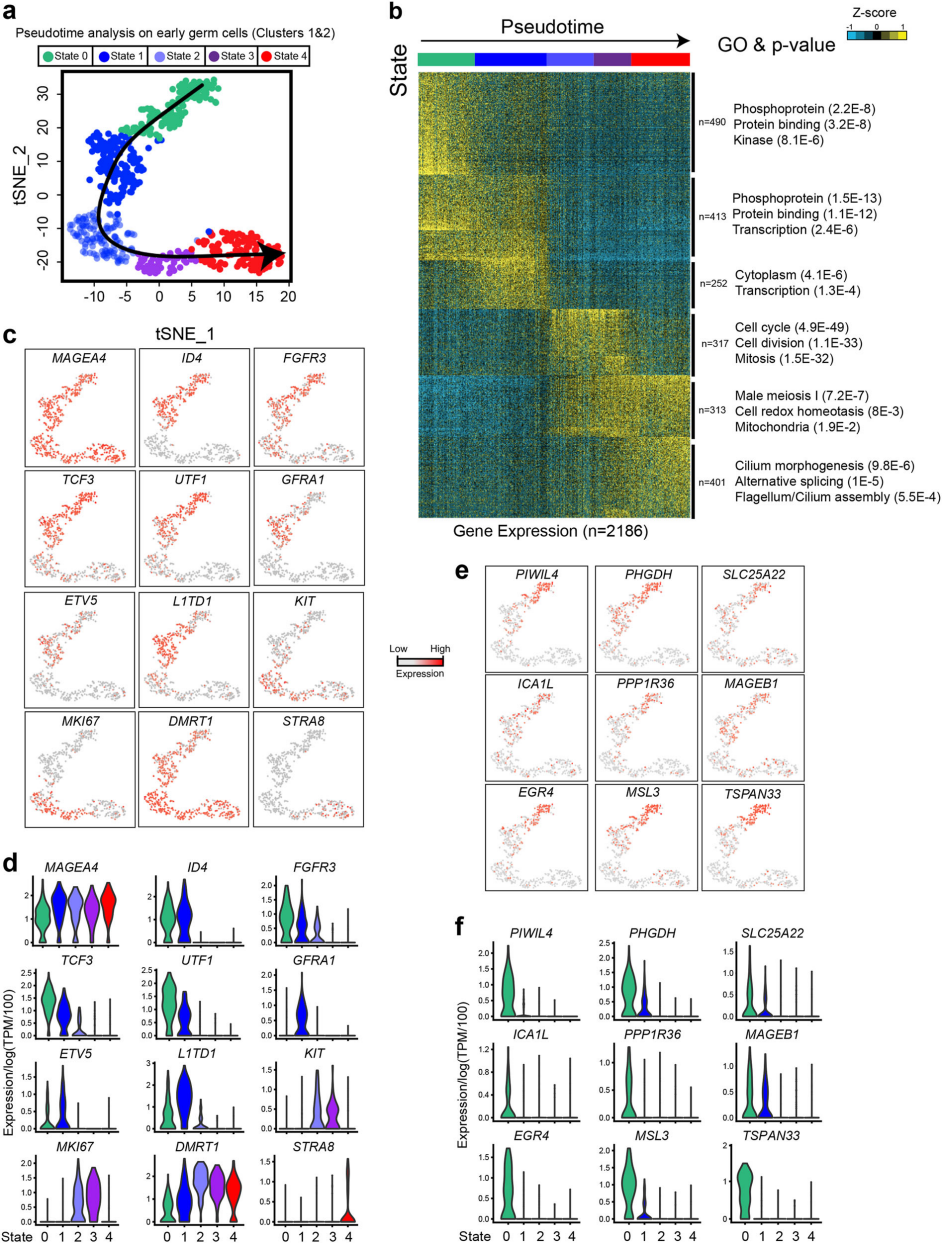
Identification of five discrete transcriptional states for SSCs. **a** Focused analysis (tSNE, clustering and pseudotime ordering) of Clusters 1 and 2 (from Fig. 1b and 3a) reveals five discrete cellular states (States 0 to 4) during SSC development. **b** K-means clustering (*k* = 6) of genes exhibiting differential gene expression in States 0–4. Six gene clusters (termed S1-S6) were identified. Gene ontology associated with each gene block is shown on the right. Note: each row represents a gene, and each column represents a single cell, with columns/cells placed in pseudotime order (depicted by different colors on the top of the figure) as defined in **a**. Gene expression levels utilize a *Z* score, which depicts variance from the mean, as defined on the color key in the right top corner. **c** Relative expression levels of selected SSC markers projected on the tSNE plot represented in **a**. **d** Violin plots representing the expression levels of the selected markers shown in **c** in States 0–4 (*x*-axis). **e** Relative expression levels of selective State 0-specific markers projected on the tSNE plot represented in **a**. **f** Violin plots representing the expression levels of selective State 0-specific markers shown in **e** in States 0–4 (*x*-axis)

To further characterize the spermatogonial ‘States’, we re-clustered the early germ cells Clusters 1–2 (Fig. 5a). This analysis yielded five distinct clusters: while four showed high similarity to the clusters/states previously described (States 1–4 in ref.^10^) an additional state, hereafter termed ‘State 0’ was identified (Supplementary information, Table S4). None of the clusters/states (including State 0) consisted of cells derived from a particular donor or within a specific cell cycle phase (Supplementary information, Fig. S5a–d). Pseudotime analysis revealed a wave-like progression from State 0 to State 4, and clustering analyses defined gene expression signatures associated with each State (Fig. 5b; Supplementary information, Table S5). Notably, we observed a striking shift in transcriptional programs between State 1 and State 2, dominated by expression of cell cycle/proliferation genes (e.g., *MKI67*), suggesting that this transition represents a critical developmental node (see Discussion).

While both State 0 and State 1 cells co-express many key stem cell signaling factors and TFs (Fig. 5c, d), our analyses identify 490 genes that are either most highly expressed, or specifically expressed, in State 0 (e.g., *PIWIL4*, *EGR4, TSPAN33, PHGDH*, *PPP1R36*, *ICA1L* (Fig. 5e, f)). Known early SSC markers (*ID4*, *FGFR3, TCF3* and *UTF1*) were expressed in States 0 and 1, whereas known markers of differentiation (*KIT)* or proliferation (*MKI67)* were specifically expressed during or after State 2, suggesting State 0 and State 1 may represent two distinct quiescent SSC states. Interestingly, most State 0 cells displayed low expression of *ST3GAL2*, the enzyme that catalyzes the formation of SSEA4 (Supplementary information, Fig. S5g), suggesting that State 0 cells do not express this spermatogonial cell surface marker.

### RNA velocity analysis and chromatin profiling suggest SSC plasticity

**Fig. 6:**
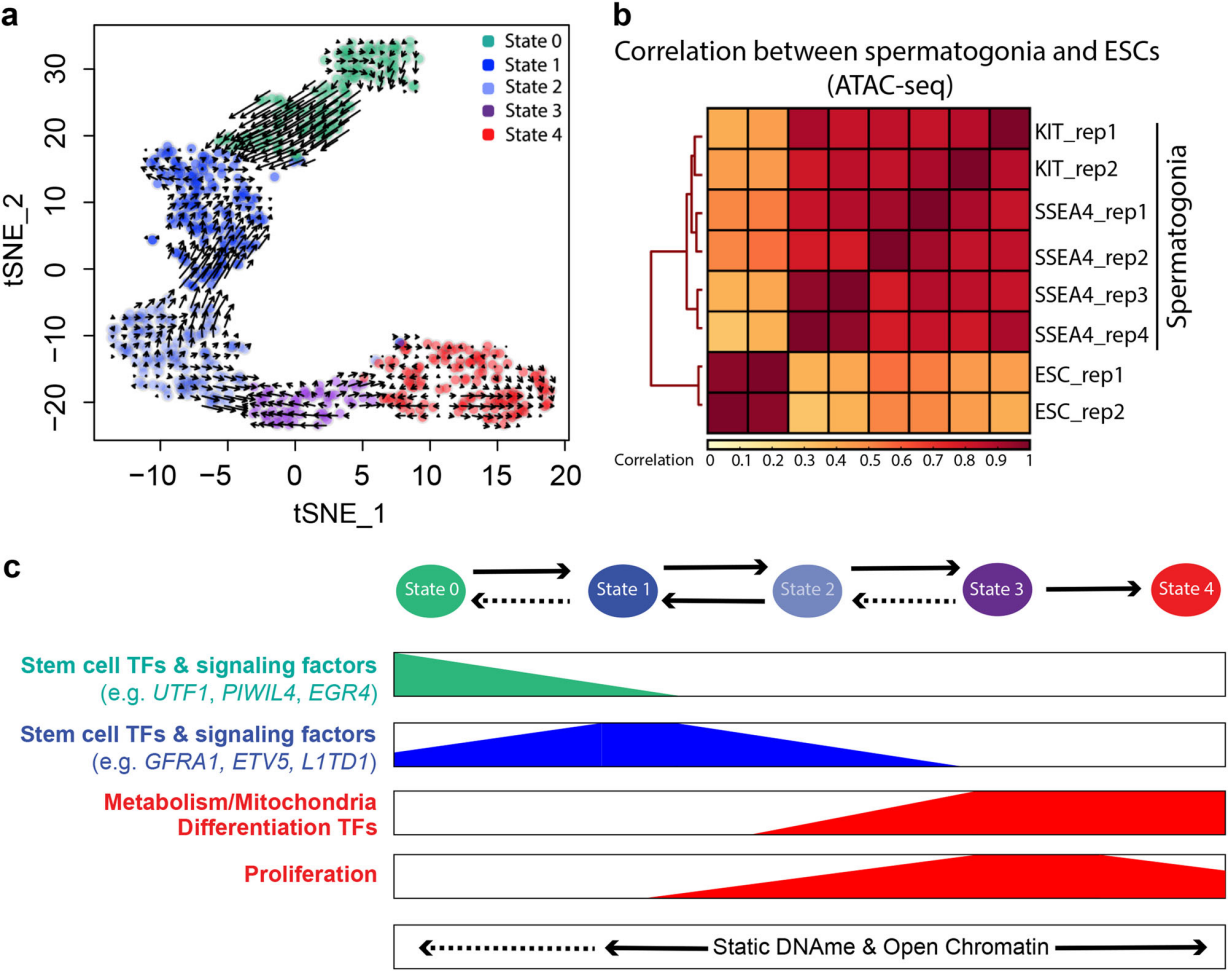
Computational and molecular examination of spermatogonial plasticity. **a** Visualization of the RNA velocity analysis results on the tSNE plot of SSCs (see main text for details on vectors). **b** Heatmap and hierarchical clustering of ATAC-seq data from KIT^+^ spermatogonia (two replicates), SSEA4^+^ SSCs (four replicates) and ESCs (two replicates). Note: SSEA4^+^ SSC and ESC data are from ref.^10^ **c** Schematic summarizing the combinatorial gene expression programs and cellular events promoting five distinct SSC states (States 0–4) and depiction of the proposed spermatogonial dynamics/kinetics and behavioral plasticity of States with main cellular events and molecular pathways. Dotted arrows are speculative

Next, we applied RNA ‘velocity’ analysis, a computational approach that utilizes nascent transcription in scRNA-seq datasets to infer developmental trajectories.^11^ Here, the ratio of unspliced to spliced reads for each transcript is used as a proxy measurement of new transcription. By comparison with steady state (spliced) transcripts in the other cells, a velocity vector representing the future transcriptional state of each individual cell can be defined. Within each cell of the tSNE plot (Fig. 6a), the amplitude and direction of the vector reflects a transcriptional trajectory. This analysis revealed two unexpected features. First, within the State 0 cluster, we observed two sub-populations: one proximal to State 1, bearing long vectors, indicating an apparent progression towards State 1—and a second sub-population lacking long vectors. This pattern suggests that the former cell sub-population is actively progressing/committing towards State 1, in response to specific developmental cues/signals, while the latter is not. Second, we also observed a sub-group of State 2 cells displaying long velocity vectors pointing back toward State 1. This forward and backward movement between States 0–1–2 raises the possibility that human spermatogonia display dynamic plasticity and metastable/uncommitted behaviors.

We then explored whether methylation or chromatin status may provide further evidence of plasticity. First, although > 8,000 genes show differential expression during spermatogonial development and spermiogenesis (Fig. 3b), we observed almost no differences in the DNAme profiles of SSEA4-enriched human SSCs and that of mature human sperm—which parallels a similar finding in mice.^10,30^ Thus, no DNAme barrier exists that might deter spermatogonial de-differentiation. Next, we profiled open chromatin from differentiating/committed spermatogonia (enriched using c-KIT), and compared it to profiles of self-renewing SSCs (enriched with SSEA4) (Fig. 6b). Notably, the open chromatin maps of c-KIT or SSEA4-enriched spermatogonia were highly similar (*r* > 0.83), and their nearest peak summits were typically overlapping (within a distance of ~120 bp), indicating that very few changes in the open chromatin landscape occurred during the commitment of undifferentiated SSEA4^+^ SSCs into differentiating c-KIT^+^ spermatogonia, in spite of the activation and repression of hundreds of genes. Taken together, the scRNA-seq analysis, the RNA velocity trends and evidence derived from the analysis of open chromatin landscape and DNAme are consistent with the proposal that SSCs follow a developmental progression involving 5 sequential transcriptional states characterized by a ‘flat’ chromatin/DNAme landscape, strongly suggestive of dynamic behaviors of spermatogonial cells (Fig. 6c).

### The adult State 0 is most similar to infant germ cells

**Fig. 7:**
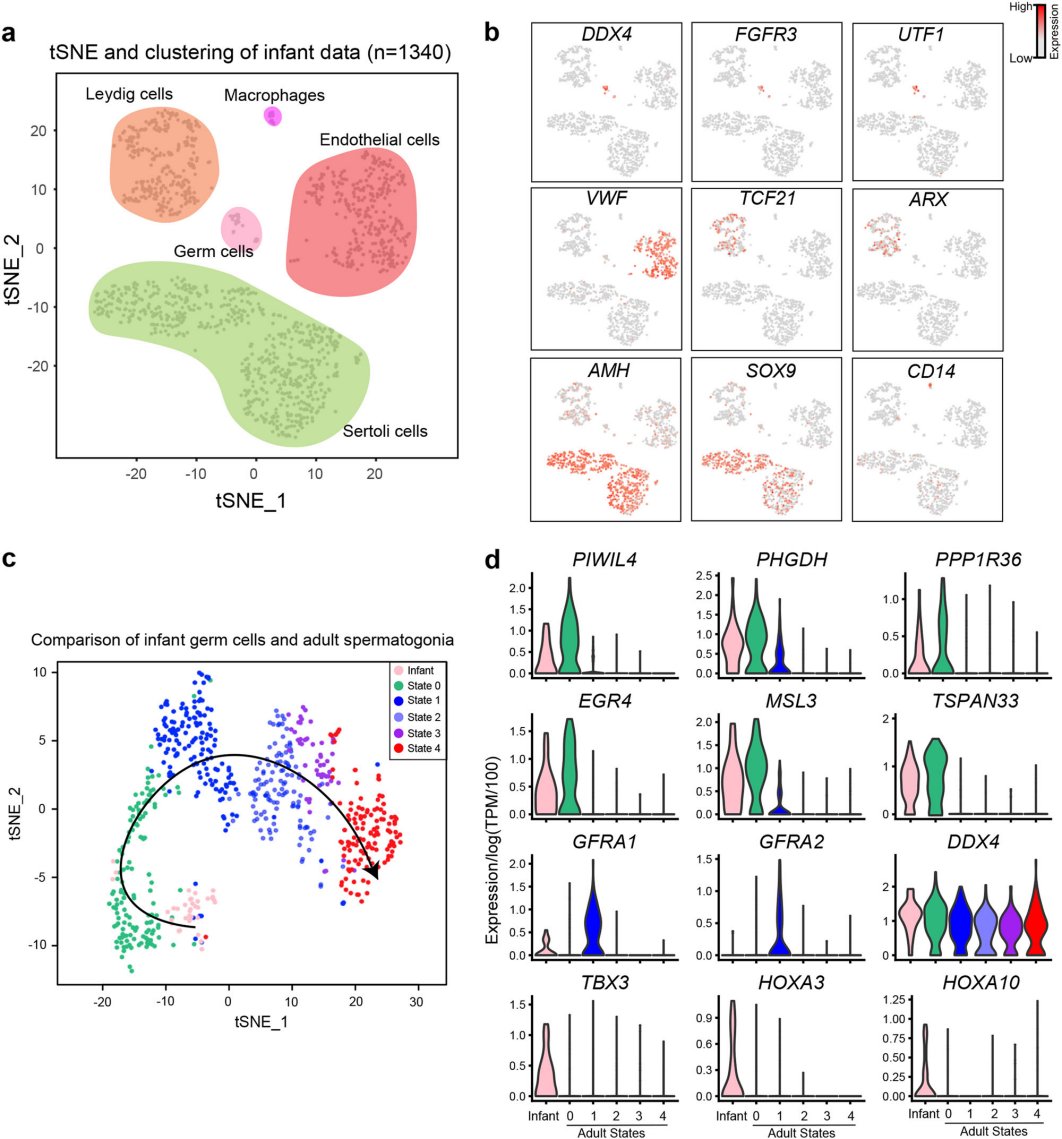
Single cell RNA profiling from infant testis and comparison to adult scRNA-seq data. **a** tSNE and clustering analysis of single-cell transcriptome from infant testis (*n* = 1340). **b** Expression patterns of representative markers to help assign cell identities. **c** tSNE and pseudotime analysis of infant germ cells and adult spermatogonia. **d** Expression patterns (violin plot) of representative genes in infant germ cells and adult spermatogonia

To assess whether State 0 may represent the earliest/naïve SSC in adults, we profiled testicular cells from infants (12–13 months old). After QC filtering, we obtained ~1300 single cells and assigned cell identities based on known markers. This analysis identified four somatic cell types, i.e., Sertoli, Leydig, endothelial and macrophage (Fig. 7a, b; Supplementary information, Fig. S7), and one tight cluster of 37 germ cells. Comparison to adult SSC States via tSNE and pseudotime analysis, positioned the infant germ cells adjacent to adult State 0, at the ‘beginning’ of the developmental trajectory (Fig. 7c). Moreover, most State 0 markers were highly expressed in infant germ cells (Fig. 7d). A small set of factors (e.g., *TBX3*, *HOXA3*) showed specific expression in infant spermatogonia,^31^ which may specify their germline identity. In addition, transcriptomic data of infant somatic cells should provide a useful resource for future analysis.

### Validation of SSC States via sequential mRNA fluorescence in situ hybridization

**Fig. 8:**
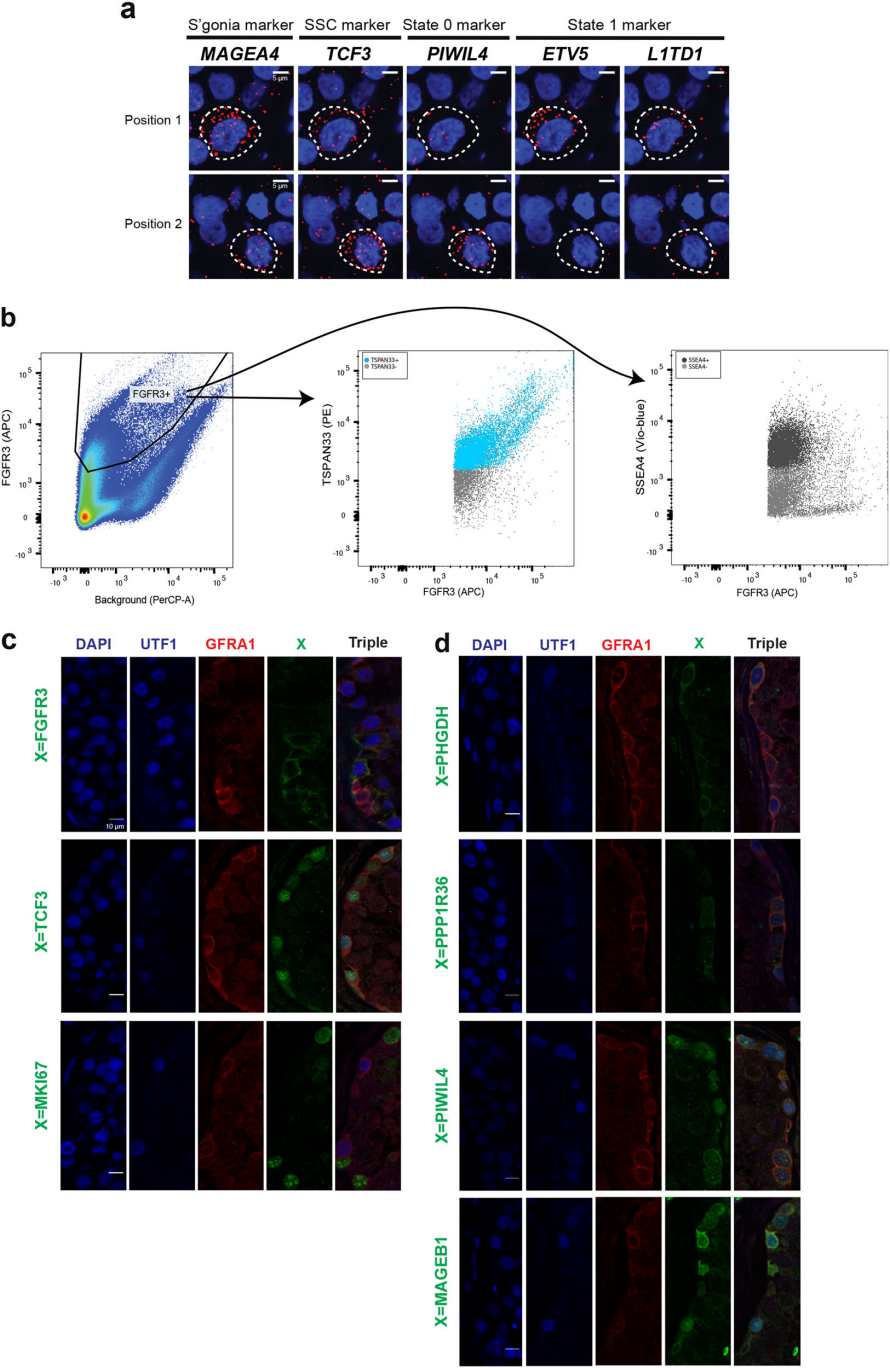
RNA and protein staining to validate state 0. **a** Sequential RNA FISH of SSC markers on tubular sections. Two SSCs are highlighted as representative examples. Blue is the DAPI signal; red detects RNA FISH signal for gene as indicated on the figure. White dashed line circles represent the cell boundaries. Scale bar: 5 μm. **b** Expression patterns of FGFR3 (marks State 0 and 1), TSPAN33 (marks State 0) and SSEA4 (marks State 1) in testicular cells via flow cytometry. Human single testicular cells were used for staining with the markers indicated; non-stained cells were used as control for gating purposes. Left: FGFR3^+^ cells were identified and used for analysis in the middle and right panels. Middle: co-staining pattern between FGFR3 (*x*-axis) and TSPAN33 (*y*-axis), with blue as TSPAN33^+^ and red and gray as TSPAN33^−^. Right: co-staining pattern between FGFR3 (*x*-axis) and SSEA4 (*y*-axis), with black as SSEA4^+^ and gray as SSEA4^−^. **c** Immunolocalization of UTF1 (State 0 marker, in blue), GFRA1 (State 1 marker, in red) and FGFR3 or TCF3 or MKI67 (in green). Each combination of single or triple antigen (named in green on the left side) is represented by 5 panels. Scale bar: 10 μm. **d** Immunolocalization of UTF1 (State 0 marker, in blue), GFRA1 (State 1 marker, in red) and 4 new candidate markers (in green). Each antigen (named in green on the left side) is represented by 5 panels. Scale bar: 10 μm

For validation, we performed sequential single molecule RNA fluorescence in situ hybridization (seqFISH)^32^ on 5 key genes (Fig. 8a; Supplementary information, Table S6). In cells expressing moderate/high *TCF3* (State 0 and State 1), only 27% (9/33) displayed high expression of *PIWIL4* (State 0 marker) and *ETV5*/*L1TD1* (State 1), while the remainder expressed either *PIWIL4* (27%) or *ETV5*/*L1TD1* (39%). This yielded significant non-overlap between State 0 and State 1 markers (hypergeometric test; *p* = 0.03), consistent with scRNA-seq data.

### The State 0 marker TSPAN33 shows co-localization with high FGFR3

We next sought to enrich State 0 cells using cell surface markers, and assess their SSEA4 status. Here, we used TSPAN-family receptors, which show a strong enrichment in State 0 (e.g., TSPAN33; Fig. 5f). Flow cytometry analysis of cells expressing high levels of the SSC marker FGFR3 (peaks in State 0, present in State 1; Fig. 5d), show that FGFR3^high^ cells have high TSPAN33 but low SSEA4 (Fig. 8b)—characterizing State 0 as FGFR3^high^ TSPAN33^high^ SSEA4^low^.

### In situ observation of early SSC States via protein immunofluorescence

We characterized the protein expression of early spermatogonial markers (UTF1, GFRA1, FGFR3, and TCF3)^33^ that exhibit differential expression across early States (0–1–2) by performing triple immunofluorescence (IF) staining. *UTF1* expression peaked at State 0, and only partially overlaped with *GFRA1*, which peaks in State 1 (Fig. 5d). We observed that ~66% of cells located at the periphery of the seminiferous tubules expressed either UTF1 and/or GFRA1, as previously reported.^34^ Here, we distinguished two broad spermatogonial phenotypes, characterized by either UTF1^high^/GFRA1^low^ or GFRA1^high^/UTF1^low^, that recapitulate the temporal progression from State 0 to State 1. Furthermore, the spermatogonial markers FGFR3 and TCF3 were expressed in a subset of cells expressing either UTF1 and/or GFRA1, but were never observed on their own (Fig. 8c). One simple explanation is that RNAs strongly expressed in State 0 (e.g., *UTF1*) produce a protein with a longer half-life than its RNA, and therefore the protein persists into the State 1 *GFRA1*-expressing cells—providing heterogeneity. As expected, the proliferating marker MKI67 showed almost no overlap with GFRA1 or UTF1 (Fig. 8c and data not shown), suggesting that transition to the proliferative State 2 occurs with the loss of SSC markers, which is consistent with our computational analyses (Figs. 5 and 6c).

Finally, we further characterized the expression of genes specific to State 0 or State 1. Using the Human Protein Atlas resource (http://www.proteinatlas.org/) to review the pattern of 490 State 0 genes,^35^ 16 candidate early SSC markers which displayed specific expression in cells located along the periphery of the seminiferous tubule were chosen. Triple IF staining with UTF1 and GFRA1 showed that each of these 16 markers is expressed in GFRA1-positive and/or UTF1-positive cells. Antibodies to PHGDH and PPP1R36 display strong staining in UTF1^high^ (State 0) cells, as predicted by the scRNA-seq analysis (Fig. 8d). Other State 0 markers showed expression in early spermatogonia (Fig. 8d; Supplementary information, Fig. S8b), an expected result for instances where the protein half-life is greater than that of the transcripts. In several instances, including PIWIL4 and MAGEB1, we observed differences in staining intensity and/or sub-nuclear localization in cells co-expressing either UTF1^high^ or GFRA1^high^ marker (Fig. 8d; Supplementary information, Fig. S8b). These observations are consistent with the proposal that although State 0 and State 1 define transcriptionally discrete states, they likely represent metastable/heterogeneous cellular phenotypes that afford SSCs the ability to adapt to a dynamic niche environment and ensure homeostatic regulation within the testis.

## Discussion

Human adult spermatogenesis is a complex process, and a full understanding will involve the integration of multiple data types—including those from rodents, where genetic tools and SSC culturing systems are already available—to determine both shared and unique mechanism in mice and men. Here, we aimed to provide foundational scRNA-seq data of all cells contained within the normal human young adult testis, complemented by computational analysis and validation studies—to offer new insights into the regulation of male gametogenesis in humans.

### Signaling features in the human testis niche

A major area of current interest involves communication between the niche and germline, and how these interactions mediate the changes observed during puberty and ageing. Here, we report new data that reveal potential differences between mice and men, and changes during development, for future functional investigation. First, for CXCR4-CXCL12 signaling (for SSC homing to the niche), *CXCL12* was primarily expressed at the RNA level by Leydig cells in humans rather than by Sertoli cells in mice. Furthermore, mouse SSCs express *Csf1r* for Csf1 response, while human *CSF1R* expression appears to be specific to macrophages. Moreover, we identified novel markers for niche cells, like *WFDC2* and *PRND* for Sertoli cells, and *VIT* for Leydig cells. Beyond these examples, our datasets provide a resource for additional analyses of niche cells and niche-germline communication. Furthermore, our transcriptome data on infant testes concur that major differences exist between infant somatic cells and their counterparts in the adult (Supplementary information, Fig. S7), and exploration of the mechanisms mediating these changes prior to and during puberty will be an important future research focus.

### Computational analysis of spermatogonial development and spermatogenesis

Here, we demonstrate that the dynamic and temporal trajectory of germline development could be reconstructed using scRNA-seq data and dedicated computational/analytical tools. The distribution of cell types for all three donors largely overlap during SSC stages and the early and mid stages of gametogenesis, establishing overall consistency between donors. However, modest differences were observed in late stages. Here, we note that the RNA content of maturing/mature sperm is both low and of poor integrity, a known and conserved property in mammals, conferring low number of genes/UMIs (unique molecular identifiers) in the maturing sperm datasets (Supplementary information, Fig. S1d). Therefore, although two distinct clusters of sperm were computationally obtained (Clusters 7 and 8), an alternative interpretation is that these sperm populations are similar, and simply contain different levels of RNA degradation/removal during this unique, transcriptionally inactive stage.

We also provide several new insights into spermatogonial development, most importantly the identification of a novel and early quiescent state, termed State 0. This interpretation is based on the properties/categories of the 490 differentially-regulated genes and the high similarities to the transcriptomic profile of infant germ cells. Moreover, State 0 cells (in contrast to State 1 cells) show low expression of the cell surface marker SSEA4, likely explaining why they escaped characterization in our prior study, which relied on SSEA4-mediated enrichment of testicular cells. Thus, we suggest that State 0 cells represent the undifferentiated and quiescent ‘reserve’ stem cell pool in the adult germline that is largely maintained from infants to adults.

Our data also call for a re-examination of prior work on human spermatogonial development. GFRA1 marks early undifferentiated spermatogonia,^36^ but exhibits a heterogeneous expression in human^34^ and mouse^37^ SSCs. Recently, using immunostaining, DiPersio et al.,^34^ proposed that ‘early’ (slow proliferating) GFRA1^high^ UTF1^−^ SSCs progress to GFRA1^low^ UTF1^*+*^, before committing to differentiation (c-KIT^+^). However, our scRNA-seq data and pseudotime analysis suggest an alternative sequence of events—where *UTF1* precedes the expression of *GFRA1*—which, interestingly aligns with recent work in the mouse.^37^ Beyond these two markers, we additionally provided data on hundreds of candidate spermatogonial markers (and validated 16 new protein markers) that define State 0 and State 1 SSCs in humans.

### Computational and molecular evidence for plasticity within early spermatogonia

Plasticity and stochastic behaviors within spermatogonial stem cell populations have been reported in several species including *Drosophila* and mice, in which differentiating spermatogonia can dedifferentiate and regain their early self-renewing properties.^38–41^ Our work provides two lines of evidence consistent with developmental plasticity in early human spermatogonia. First, RNA velocity analysis singled out a population of State 2 spermatogonia tending to ‘dedifferentiate’ into State 1-like cells. Notably, the transition from State 1 to State 2 is marked by an upregulation of proliferative markers—and thus might be a critical node for homeostasis by which developmental plasticity allows to balance early versus differentiating SSC populations.^42^ Second, we observe very limited changes in open chromatin and DNAme along the developmental trajectory of spermatogonia, which may enable transcriptional plasticity to take place by lowering epigenetic barriers to transcriptional changes, and dedifferentiation. Overall, we propose a spermatogonial developmental trajectory that involves 5 sequential transcriptional States, which generate moderately heterogeneous (metastable) proteomes, to enable State transitions and maintain a constant SSC pool; properties which might be essential to maintain life-long fertility, and critical for the germline replenishment in case of damage.

### A resource for future investigation of spermatogenesis

Recently, scRNA-seq has emerged as a highly useful approach for the study of human and mouse spermatogenesis.^29,43,44^ Here, our data reveals **>** 8000 genes that undergo significant differential regulation during male gametogenesis, and our results align well with a very recent scRNA-seq study of gametogenesis.^44^ Beyond coding genes, our work has uniquely explored transposable elements (TE) and long non-coding RNAs—which are shown to display remarkable stage-specific expression. Of particular interest are the expression of active LTR12C/D/E, SVA_D and AluYa5 elements during early spermatogenesis, LTR10A and LTR40c elements during spermatogonial stages. We also described *XIST* expression during spermatogonial stages—which we show coincides with the selective attenuation of genes near the XIC (Supplementary information, Fig. S4), suggesting an unexpected role for *XIST* spreading and silencing in this process. Taken together, our datasets and analyses provide a comprehensive resource for the study of both niche cells and germline cells—including coding genes, TEs and lncRNA expression dynamics—which will also serve as a useful reference dataset for comparisons to younger and older men, infertile men, and testicular cancer.

## Materials and methods

### Experimental model and subject details

Adult human testicular samples for scRNA-seq and immunostaining were from three healthy men (donor #1: 17 years old; donor #2: 24 years old; donor #3: 25 years old); sample for mRNA seqFISH was from a healthy man (donor #4: 23 years old). Infant testicular samples for scRNA-seq were from two infant donors (13 months old). All six samples were obtained through the University of Utah Andrology laboratory and Intermountain Donor Service. Those samples were removed from deceased individuals who consented to organ donation for transplantation and research. Sample used for ATAC-seq was obtained through the University of Utah Andrology laboratory consented for research (IRB approved protocol #00075836: understanding the transcriptional and epigenetic dynamics in human spermatogonial stem cell self-renewal, proliferation and differentiation).

### Sample storage by cryopreservation

Once collected, the pair of whole testis samples was transported to the research laboratory on ice in saline or Hank’s Balanced Salt Solution (HBSS; GIBCO cat # 24020117) and processed within 1 h of removal by surgery. Around 90% of each testis was divided into smaller portions (~0.5–1 g for each) using scissors and directly transferred into cryovials (Corning cat # 403659) in DMEM medium (Life Technologies cat # 11995073) containing 10% DMSO (Sigma-Aldrich cat # D8779), 15% fetal bovine serum/FBS (Gibco cat # 10082147) and cryopreserved in a Mr. Frosty Freezing container (Thermo Fisher Scientific cat # 5100–0001) ensuring a slow controlled freezing rate at −80 °C for overnight. Cryovials were then transferred to liquid nitrogen for long-term storage.

### Sample fixation for immunostainings

Around 10% of the remaining testis tissues were incubated in 40 mL of 1× PBS containing 4% paraformaldehyde/PFA (Thermo Fisher Scientific cat # 28908) overnight at 4 °C with agitation on a rotor (60 rpm). Fixed samples were then washed three times in cold PBS and stored in PBS at 4 °C until processing for immunostaining.

### Human adult testis sample preparation for single cell RNA sequencing

For each single cell sequencing experiment (technical replicate for one donor), ~5 cryovials were thawed in ~3 min. Tissues were washed twice in 1× PBS, and subjected to a standard two-step digestion procedure, as described previously.^10^ Briefly, testicular tissues were digested with collagenase type IV (Sigma Aldrich cat # C5138–500MG) for 5 min at 37 °C with gentle agitation (250 rpm), then shaken vigorously and incubated for another 3 min. The tubules were sedimented by centrifugation at 200× *g* for 5 min and washed with HBSS before digestion with 4.5 mL 0.25% trypsin/ethylenediaminetetraacetic acid (EDTA; Invitrogen cat # 25300054) and 4 kU DNase I (Sigma Aldrich cat # D4527–500ku). The suspension was triturated vigorously three to five times and incubated at 37 °C for 5 min. The process was repeated in 5 min increments for up to 15 min total. The digestion was stopped by adding 10% FBS (Gibco cat # 10082147). Single testicular cells were obtained by filtering through strainers with mesh size 70 µm (Fisher Scientific cat # 08–771–2) and 40 µm (Fisher Scientific cat # 08–771–1). The cells were pelleted by centrifugation at 600× *g* for 15 min, and washed twice with 1× PBS. Cell number was measured using hemocytometer, and cells were then re-suspended in 1× PBS + 0.4% BSA (Thermo Fisher Scientific cat # AM2616) at the concentration of ~1000 cells/uL, ready for single cell sequencing.

### Human infant testis sample preparation for single cell RNA sequencing

We performed two technical replicates for the infant donor. A quarter of the testis was thawed in ~5 min. Tissues were washed twice in 1× PBS, and minced into small pieces for better digestion outcome. Tissues were then treated with trypsin EDTA for ~25 min at 37 °C. The digestion was then stopped by adding 10% FBS (Gibco cat # 10082147). Single testicular cells were obtained by filtering through strainers with mesh size 70 µm (Fisher Scientific cat # 08–771–2) and 40 µm (Fisher Scientific cat # 08–771–1). The cells were pelleted by centrifugation at 600× *g* for 15 min, and washed twice with 1× PBS. Cell number was measured using hemocytometer, and cells were then re-suspended in 1× PBS + 0.4% BSA (Thermo Fisher Scientific cat # AM2616) at the concentration of ~1000 cells/uL, ready for single cell sequencing.

### Single cell RNA-seq performance, library preparation and sequencing

scRNA-Seq was performed using the 10× Genomics system. Briefly, each experiment captured ~1500 single cells, in order to obtain ~0.8% multiplex rate. Cells were diluted following manufacturer recommendations, and mixed with 33.8 µL of total mixed buffer before being loaded into 10× Chromium Controller using Chromium Single Cell 3’ v2 reagents. Each sequencing library was prepared following the manufacturer’s instructions, with 13 cycles used for cDNA amplification. Then ~100 ng of cDNA were used for library amplification by 12 cycles. The resulting libraries were then sequenced on a 26 × 100 cycle paired-end run on an Illumina HiSeq 2500 instrument.

### Process of single cell RNA-seq data

Raw sequencing data were demultiplexed using the mkfastq application (Cell Ranger v1.2.1). Three types of fastq files were generated: I1 contains 8 bp sample index; R1 contains 26 bp (10 bp cell-BC + 16 bp UMI) index and R2 contains 100 bp cDNA sequence. Fastq files were then run with the cellranger count application (Cell Ranger v1.2.1) using default settings, to perform alignment (using STAR v2.5.4a), filtering and cellular barcode and UMI counting. The UMI count tables of each cellular barcode were used for further analysis.

### Sequential RNA florescence in situ hybridization

Non-barcoded seqFISH (sequential FISH) probes were designed by targeting consensus of all constitutive exons (Supplementary information, Table S6) present in the masked hg38 human genome with 35-nucleotide (nt). All probes were blasted against the human transcriptome, and expected copy numbers of off-target probe hits were calculated using predicted RNA counts from RNA-seq dataset.^10^ Probes were then attached with one of the DNA hybridization chain reaction (HCR) initiator sequences (B1, B2, B3, B4 or B5) at 5’ end with 4-nt ‘ATAT’ space in between. Initiator sequences were specific to genes in each round of hybridizations.

Non-barcoded seqFISH was performed by following the previous protocol.^32^ Human testis tissues were first perfused with RNase-free PBS, and embedded in 30% RNase free sucrose (VWR cat # 97061–430). After the tissues sank, they were frozen using a dry ice/isopropanol bath in OCT media and stored at −80 °C. 15-µm sections were cut using cryotome and immediately placed on an aminosilane modified coverslip. The generated human testis sections mounted to coverslips (Thermo Scientific cat # 152450) were permeabilized at 4 °C in 70% EtOH for 12–18 h. Tissue sections were further permeabilized by adding RNase-free 8% SDS (10% SDS Ambion cat # AM9822) for 20 min. Samples were rinsed with 70% EtOH to remove SDS, and air-dried. The hybridization chambers (Grace Bio-Labs cat # 621505) were adhered around the tissue sections. Then samples were washed once with 2× SSC (Invitrogen cat # 15557–036) diluted in Ultrapure water (Invitrogen cat # 10977–015), and hybridized with 2.5 nM probes per incubation for overnight at 37 °C in Hybridization Buffer (50% HB: 2× SSC, 50% Formamide (v/v) (Ambion cat # AM9344), 10% Dextran Sulfate (w/v) (Sigma cat # D8906), in Ultrapure water). Samples were washed in 50% Wash Buffer (50% WB: 2× SSC, 50% Formamide (v/v), 0.1% Triton-X 100 (Sigma X-100) in Ultrapure water) for 30 min at room temperature. While washing, aliquoted HCR hairpins (Molecular Instruments Inc.) were heated to 95 °C for 1.5 min and allowed to cool to room temperature for 30 min in the dark. The HCR hairpins were diluted to a concentration of 120 nM per hairpin in amplification buffer (2× SSC, 10% Dextran Sulfate (w/v)), and incubated with the samples for 45 min at room temperature in the dark. Following amplification, samples were washed in the 30% Wash Buffer (30% WB: 2× SSC, 30% Formamide (v/v), 0.1% Triton-X 100 in Ultrapure water) for 30 min to remove non-specifically bound hairpins. Samples were then stained with 5 μg/mL DAPI (Sigma cat # D8417) in 2× SSC and imaged as described below. After imaging, samples were digested with DNase I (10 units of DNase I, 1× buffer (Roche cat # 04716728001) in Ultrapure water) for 2 h at 37 °C on the microscope using custom made heat pad. Following DNase I treatment, the samples were washed with 30% WBT at 37 °C for 30 min, and hybridized with the following round of probe set for overnight with 2.5 nM probes per each in 50% HB at 37 °C on the microscope. Samples were then washed, amplified with HCR hairpins and imaged as before. The above steps were iterated at each hybridization round.

Following the last non-barcoded seqFISH, immunofluorescence was performed. The samples were washed with 1× PBS (Ambion cat # AM9624) for a few times, blocked with 5% BSA blocking solution (5% BSA (Gemini cat # 700–106 P), 1× PBS, and 0.3% Triton-X 100 in Ultrapure water), and then incubated at room temperature for 1 h. The primary antibody, Anti-beta Catenin (Abcam cat # ab6301), was 100-fold diluted in 1% BSA solution (1% BSA, 1× PBS, 0.3% Triton-X), incubated with the samples at room temperature for 3 h. The samples were then washed with 1× PBS for three times for 15 min each. The secondary antibody (anti-mouse Alexa Fluor 647 Invitrogen cat # A31571) was 500-fold diluted in 1% BSA buffer, and incubated with the samples at room temperature for 1 h. Samples were washed with 1× PBS for three times, stained with DAPI and imaged as described below.

Samples were imaged in an anti-bleaching buffer (14 mM Tris-HCl, pH = 8.0, 35 mM NaCl, 0.8% D-Glucose (Sigma cat # G7528), 100-fold diluted Catalase (Sigma cat # C3155), Pyranose oxidase with OD_405_ of 0.05 (Sigma cat # P4234), and saturated amount of Trolox (Sigma cat # 238813)) with the microscope (Leica, DMi8) equipped with a confocal scanner unit (Yokogawa CSU-W1), a sCMOS camera (Andor Zyla 4.2 Plus), 63× oil objective lens (Leica 1.40 NA), and a motorized stage (ASI MS2000). Lasers from CNI and filter sets from Semrock were used. Snapshots were acquired with 0.5 μm z steps across 15 μm with more than 10 positions per sample.

seqFISH signals were visualized using ImageJ software. Firstly, all images were aligned manually in xy and z by using DAPI channel signals. Each channel of HCR signals was background subtracted using ImageJ’s subtract background function with rolling ball radius of 3 pixels. Images were applied by ImageJ’s mean integral image filter with block radius of 3 pixels, and then contrasted. Each image was visualized with an overlay of DAPI signals of the first round of hybridization.

### Immunostainings of testis tissues

The triple immunofluorescence stainings were performed on 5 µm formalin-fixed paraffin-embedded (FFPE) sections from portions of the testis from Donor 2 and 3 (24 and 25 years old respectively) following deparaffinisation, rehydratation and heat-mediated antigen retrieval in 10 mM sodium citrate buffer solution (pH 6). After treatment with Superblock (PBS) Blocking Buffer (Thermo Fisher Scientific, cat # 37515) for 30 min, individual sections were incubated overnight at 4 °C with a mix of three diluted antibodies (UTF1 (mouse monoclonal), GFRA1 (goat polyclonal) and a third rabbit polyclonal antibody (for antibodies details and dilutions, see the Table below). Antigen detection was conducted using the appropriate combination of Alexa Fluor 488, 555 and 647 secondary antibodies (all 1:500; Thermo Fisher Scientific, cat # A21202, cat # A21432, cat # A31573 respectively) for 2 h at room temperature in the dark. All primary/secondary antibodies were diluted in SignalBoost™ Immunoreaction Enhancer Kit (Calbiochem, cat # 407207–1KIT). After three washes in PBS, sections were incubated with DAPI (4’,6-Diamidine-2-phenylindole dihydrochloride) (Roche, cat # 10 236 276 001) to facilitate nuclear visualization (dilution: 1 µg/mL). Specificity of the antibody staining was confirmed using the same protocol but with omission of primary antibodies. Following multiple washes in PBS, slides were mounted using Vectashield mounting medium for fluorescence (Vector Laboratories, Inc., Burlingame, CA, cat # H-1000). Images were obtained under 25× objective (LD LCI PA 25× /0.8 DIC WD = 0.57 mm Imm Corr (UV)VIS-IR (Oil-Immersion) with a Zeiss LSM 780 Upright Multi-Photon Confocal Microscope and analyzed using Image J software (NIH, Bethesda, MD, USA).

**Table:** 

Ab name	Antibody host	Ab ID	Dilution	Company
UTF1	Mouse monoclonal	MAB4337 (5G10.2)	(1:1000)	Millipore
GFRα1	Goat polyclonal	AF560	(1:25)	R&D systems
FGFR3	Rabbit mAb	C51F2 (#4574)	(1:50)	Cell signaling technology
Ki67	Rabbit polyclonal	ab16667	(1:200)	Abcam
MAGEB1	Rabbit polyclonal	HPA001193	(1:300)	Human protein atlas
PPP1R36	Rabbit polyclonal	HA077492	(1:2000)	Human protein atlas
CAMK2B	Rabbit polyclonal	HPA051783	(1:275)	Human protein atlas
PHGDH	Rabbit polyclonal	HPA24031	(1:500)	Human protein atlas
ERICH5	Rabbit polyclonal	HPA025070	(1:500)	Human protein atlas
PIWIL4	Rabbit polyclonal	HPA036588	(1:100)	Human protein atlas
TCF3	Rabbit polyclonal	HPA062476	(1:150)	Human protein atlas
APBB1	Rabbit polyclonal	HPA038521	(1:300)	Human protein atlas
C19orf81	Rabbit polyclonal	HPA060238	(1:100)	Human protein atlas
GPRC5C	Rabbit polyclonal	HPA029776	(1:135)	Human protein atlas
ICA1L	Rabbit polyclonal	HPA042507	(1:100)	Human protein atlas
LMNTD2	Rabbit polyclonal	HPA058474	(1:300)	Human protein atlas
MAGEC1	Rabbit polyclonal	HPA004622	(1:500)	Human protein atlas
TUBA1A	Rabbit polyclonal	HPA043684	(1:100)	Human protein atlas
MAP2K5	Rabbit polyclonal	HPA027347	(1:400)	Human protein atlas
HLA-DPA1	Rabbit polyclonal	HPA017967	(1:35)	Human protein atlas
SLC25A22	Rabbit polyclonal	HPA014662	(1:300)	Human protein atlas

### Human c-KIT^+^ spermatogonia isolation using MACS

c-KIT^+^ cells were enriched using magnetic activated cell sorting (MACS) protocols (Miltenyi Biotec, Inc.). Single testicular cell suspensions were incubated with anti-c-KIT microbeads (Miltenyi Biotec cat # 130–098–571) at 4 °C. Following microbead binding, cells were re-suspended in autoMACS running buffer (Miltenyi Biotec cat # 130–091–221) and ran through LS columns (Miltenyi Biotec cat # 130–042–401) placed in a magnetic field. Columns were rinsed three times with buffer in autoMACS running buffer (Miltenyi Biotec cat # 130–091–221) before being removed from the magnetic field. MACS running/separation buffer (Miltenyi Biotec cat # 130–091–221) was then applied to the column before magnetically-labeled cells were flushed out by firmly pushing the plunger into the column. Cells were then centrifuged and re-suspended to a desired concentration.

### ATAC-seq library preparation and sequencing

The ATAC-seq libraries were prepared as previously described^45^ on ~30k sorted KIT^+^ spermatogonia, SSEA4^+^ SSCs or cultured ESCs.^10^ Briefly, collected cells were lysed in cold lysis buffer (10 mM Tris-HCl, pH 7.4, 10 mM NaCl, 3 mM MgCl_2_ and 0.1% IGEPAL CA-630) and the nuclei were pelleted and resuspended in Transposase buffer. The Tn5 enzyme was made in-house and the transposition reaction was carried out for 30 min at 37 °C. Following purification, the Nextera libraries were amplified for 12 cycles using the NEBnext PCR master mix (NEB cat # M0541L) and purified using the Agencourt AMPure XP–PCR Purication (Beckman Coulter cat # A63881). All libraries were sequenced on a 125-cycle paired-end run on an Illumina HiSeq 2500 instrument.

### Flow cytometry analysis

Cells were analyzed by flow cytometry using Aria Analyzer. For FGFR3 staining, cells were firstly incubated with anti-FGFR3 antibody (mouse monoclonal; Santa Cruz cat # sc-13121), washed and then incubated with Alexa Fluor-647 (anti-mouse; Thermo Fisher cat # Z25008); for TSPAN33 staining, cells were incubated with TSPAN33 PE-conjugated antibody (R&D Systems cat # FAB8405P-015); for SSEA4 staining, cells were incubated with SSEA4 VioBlue-conjugated antibody (Miltenyi Biotec cat # 130–098–366). Gating was based on unstained and single stained samples. FACS data were analyzed using FlowJo software (Ashland).

### Quantifications and statistical analysis

#### Cell type identification and clustering analysis using Seurat program

The Seurat program (http://satijalab.org/seurat/, R package, v.2.0.0) was firstly applied for analysis of RNA-Sequencing data. To start with, UMI count tables from each replicates and donors were loaded into R using Read10X function, and Seurat objects were built from each experiment. Each experiment was filtered and normalized with default settings. Specifically, cells were retained only when they had greater than 500 genes expressed, and less than 20% reads mapped to mitochondrial genome. We first ran t-SNE and the clustering analysis for each replicate, which resulted in similar t-SNE map. Next, to minimize variation between technical replicates, we normalized and combined technical replicates from the same donor using the 10× Genomics built-in application from Cell Ranger “cellrange aggr”. Data matrices from different donors were then loaded into R using Seurat. Next, cells were normalized to the total UMI read count as well as mitochondrial read percentage, as instructed in the manufacturer’s manual (http://satijalab.org/seurat/). Seurat objects (matrices from different donors) were then combined using RunCCA function. t-SNE and clustering analyses were then performed on the combined dataset using the top 5000 highly variable genes and PCs 1–15, which showed most significant *p*-values. Given the low number of Sertoli cells (underrepresented due to size filtering), the initial clustering analysis did not identify them as a separate cluster. We performed deeper clustering of somatic cells, identified the Sertoli cell cluster, and projected it back to the overall clusters, which resulted in 13 discrete cell clusters. Correlation of different replicates was calculated based on average expression (normalized UMIs by Seurat) in each experiment.

#### Pseudotime and clustering analysis

Germ cells (Clusters 1–8) from t-SNE plot were used for pseudotime analysis by slingshot (https://github.com/kstreet13/slingshot, R package, v0.1.2–3). Cluster 1 (SSCs) was used as start, cluster 4 (secondary spermatocytes) as middle, and cluster 8 (sperm) as end of pseudotime. After pseudotime time was determined, gene clustering analysis was performed to determine the fidelity of pseudotime. Here, cells (in columns) were ordered by their pseudotime, and genes (in rows) were clustered by k-means clustering using Cluster 3.0. Different k-mean numbers were used to reach the optimal clustering number. Genes within each gene clusters were then used to perform Gene Ontology analysis by David (v6.7).

#### Transposable element and lncRNA analysis

First, gtf files for TEs and lncRNAs were downloaded from UCSC and lncipedia, respectively. TE gtf was treated and filtered using the same approach as described.^46^ These gtf files were then used to replace the default gtf files (for genes) in Cell Ranger, and UMI count tables were generated using the same approach as described above. For downstream analysis, TE and lncRNA expression patterns were cast to the gene expression based clustering and pseudotime.

#### Reclustering of spermatogonia/SSCs (Cluster 1 and 2)

We parsed out cells in Cluster 1 and Cluster 2 (total number *n* = 614), and loaded their gene expression matrices into R through Seurat. Clustering and t-SNE analyses were performed, and a small cluster (containing 24 cells) was identified as outlier and excluded from further analysis. The remaining cells (*n* = 590) were re-clustered and analyzed using t-SNE using the top 5000 highly variable genes and PCs 1–5, which showed the most significant *p*-values. Pseudotime was performed as mentioned above using slingshot (v0.1.2–3).

#### Cell cycle analysis

Cell cycle analysis was performed using scran program (https://bioconductor.org/packages/3.7/bioc/vignettes/scran/inst/doc/scran.html, R Package; v1.6.5). Briefly, cell cycle genes were obtained from scran program, and their expression in States 0–4 were loaded into scran. Cell cycle phases (G1, S, and G2/M) were then assigned to each single cell.

#### Regulon analysis

Regulon analysis was performed using SCENIC program (https://github.com/aertslab/SCENIC, R Package; v0.1.7). State 0–4 cells were used to generate the regulon activity score of transcription factors as instructed by their manual. The regulon activity scores were then projected onto the t-SNE plot based on gene expression levels.

#### RNA velocity analysis

Cell velocity analysis was performed using Velocyto.R program (http://velocyto.org, v0.5), as instructed.^11^ Briefly, at first, Velocyto used raw data to count the spliced (mRNA) and unspliced (Intron) reads for each gene, and generated a.loom file. Those.loom files were then loaded intro R (v3.4) using read.loom.matrices function to generate count tables for splicing and unsplicing reads. To generate RNA velocity map for spermatogonia, splicing and unspliced reads from States 0–4 were further used, and coordinates of the cells in the t-SNE plot were also provided. Lastly, the RNA velocity map was projected onto the t-SNE plot.

#### ATAC-seq comparison analysis

SAM alignments were generated from the Illumina Fastq files aligned to human hg19 genome using Novocraft’s novoalign aligner (http://www.novocraft.com) with the following parameters: –o SMA –r ALL 50. Peak calling was performed using macs2 (https://github.com/taoliu/MACS, v2.1.2.20160309) using the following settings: –g 2.7e9–call-summit –f BAMPE –nomodel –B –SPMR –extsize 200. Generated bedgraph file was then transformed to bw format using UCSC bedGraphToBigWig application (v4). Correlation was generated using deepTools (v3) by firstly using multiBigwigSummary bins application (with default settings) and then plotCorrelation application (with the following parameters: --skipZeros --removeOutliers). Distance of peak summit was calculated using bedtool (http://bedtools.readthedocs.io/en/latest/content/tools/makewindows.html, v2.25.0) closestBed application.

### Data and software availability

The accession number for all sequencing data reported in this paper is GEO: GSE120508. Further information and requests for reagents should be directed to and will be fulfilled by the Lead Contact, Bradley R. Cairns (brad.cairns@hci.utah.edu).

## Electronic supplementary material

Supplementary information, Figure S1

Supplementary information, Figure S2

Supplementary information, Figure S3

Supplementary information, Figure S4

Supplementary information, Figure S5

Supplementary information, Figure S6

Supplementary information, Figure S7

Supplementary information, Figure S8

Supplementary information, Table S1

Supplementary information, Table S2

Supplementary information, Table S3

Supplementary information, Table S4

Supplementary information, Table S5

Supplementary information, Table S6
