# Supplementary material for: The adult human testis transcriptional cell atlas
Source: Cell Res. 2018 Oct 12;28(12):1141–57. doi: 10.1038/s41422-018-0099-2 (PMC6274646; doi:10.1038/s41422-018-0099-2)
Supplement: Supplementary file 1 — Supplementary information, Figure S1 [file 41422_2018_99_MOESM1_ESM.pdf]

Fig. S1

a

	Total reads	Mean reads/cell	Median gene/cell	Detected gene #	Captured cell # (after filtering)	Experiment ID	Sequencing Saturation	Correlation
Donor #1 Rep 1	298 M	249,159	2,140	26,575	1,000	#1	84.0%	0.969
Donor #1 Rep 2	300 M	196,631	2,489	27,652	1,248	#2	83.2%	
Donor #2 Rep 1	263 M	248,869	2,872	27,372	985	#3	83.9%	0.998
Donor #2 Rep 2	289 M	261,963	3,278	27,755	1,009	#3	83.3%	
Donor #3 Rep 1	270 M	212,559	2,453	27,451	1,116	#3	83.3%	0.997
Donor #3 Rep 2	401 M	315,596	2,311	27,521	1,132	#3	87.3%	

b

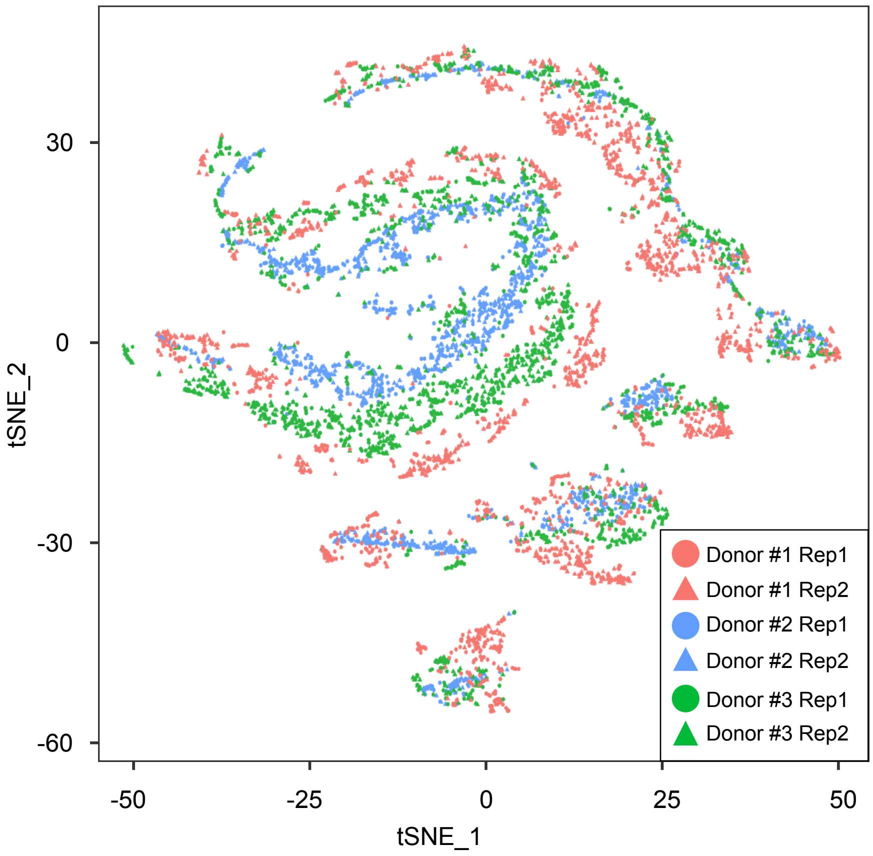

c

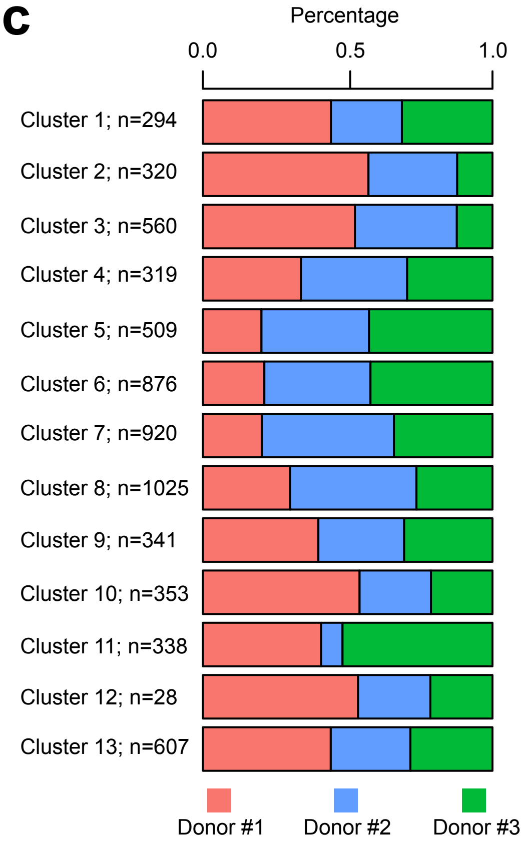

d

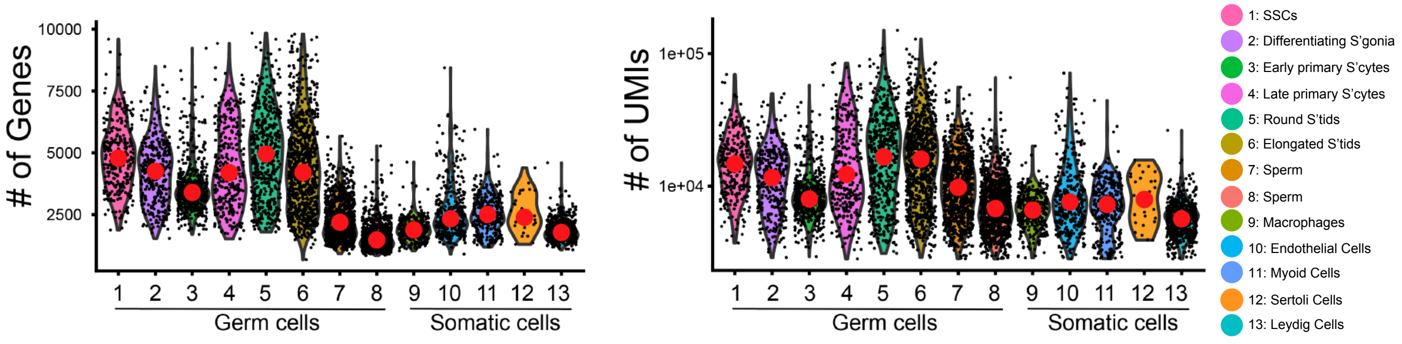

Fig. S1. Quality Control of Single Cell RNA-seq Datasets.

(a) Sequencing metrics of the six datasets (n=3 biological replicates; 2 technical duplicates) generated.

(b) tSNE plot of single cell transcriptome with cells colored based on their donors of origin and replicate.

(c) Bar graph showing the distribution of donor origin in different clusters.

(d) Violin plot of number of genes (left) or UMIs (right) for different clusters identified. Red dot represents the median. UMI: unique molecular identifier.
