# Supplementary material for: The adult human testis transcriptional cell atlas
Source: Cell Res. 2018 Oct 12;28(12):1141–57. doi: 10.1038/s41422-018-0099-2 (PMC6274646; doi:10.1038/s41422-018-0099-2)
Supplement: Supplementary file 2 — Supplementary information, Figure S2 [file 41422_2018_99_MOESM2_ESM.pdf]

Fig. S2

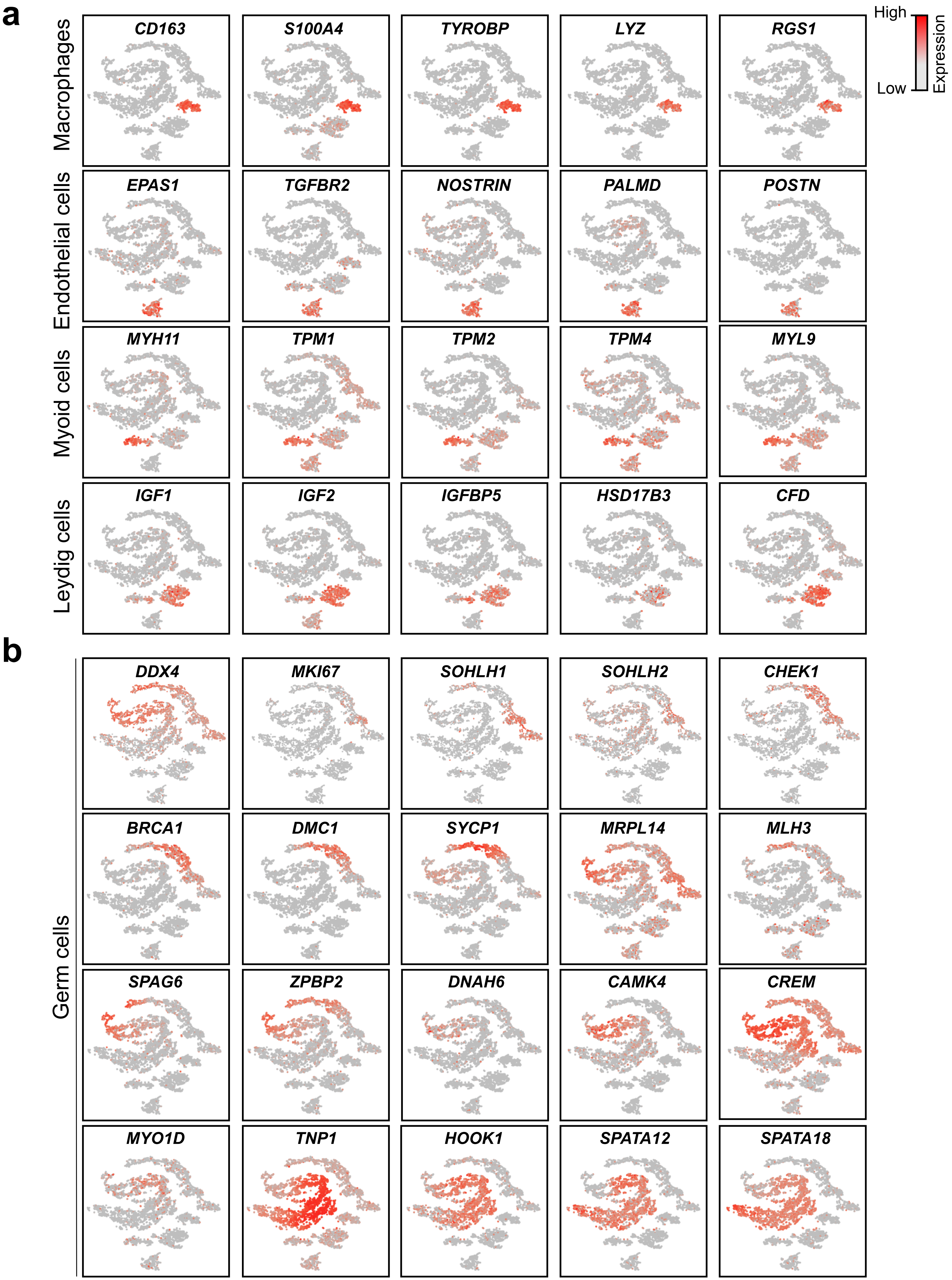
a
Macrophages
CD163
S100A4
TYROBP
LYZ
RGS1
High
Low
Expression
Endothelial cells
EPAS1
TGFBR2
NOSTRIN
PALMD
POSTN
Myoid cells
MYH11
TPM1
TPM2
TPM4
MYL9
Leydig cells
IGF1
IGF2
IGFBP5
HSD17B3
CFD
b
Germ cells
DDX4
MKI67
SOHLH1
SOHLH2
CHEK1
BRCA1
DMC1
SYCP1
MRPL14
MLH3
SPAG6
ZPBP2
DNAH6
CAMK4
CREM
MYO1D
TNP1
HOOK1
SPATA12
SPATA18

Fig. S2. Expression Patterns of Example Gene Markers in Different Clusters.

(a) Gene expression patterns of additional markers in niche cells. Top row: macrophages; second row: endothelial cells; third row: myoid cells; fourth row: Leydig cells.

(b) Gene expression pattern of additional markers in germ cells. Genes marked more differentiated germ cell population as they move from top to the bottom row.
