# Supplementary material for: The adult human testis transcriptional cell atlas
Source: Cell Res. 2018 Oct 12;28(12):1141–57. doi: 10.1038/s41422-018-0099-2 (PMC6274646; doi:10.1038/s41422-018-0099-2)
Supplement: Supplementary file 3 — Supplementary information, Figure S3 [file 41422_2018_99_MOESM3_ESM.pdf]

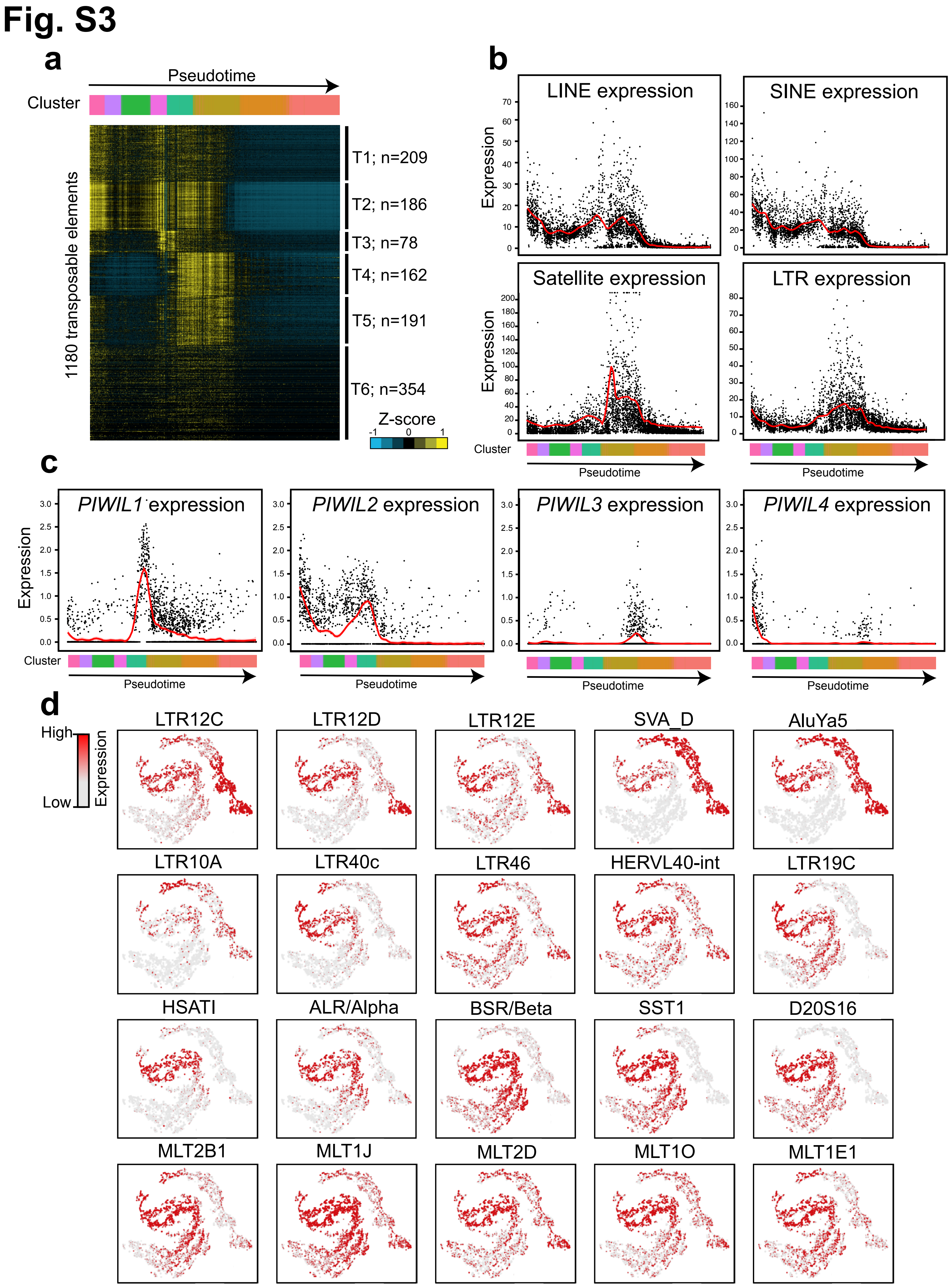
Fig. S3
a
Pseudotime
Cluster
1180 transposable elements
T1; n=209
T2; n=186
T3; n=78
T4; n=162
T5; n=191
T6; n=354
Z-score
-1 0 1
b
LINE expression
SINE expression
Satellite expression
LTR expression
Expression
Cluster
Pseudotime
c
PIWIL1 expression
PIWIL2 expression
PIWIL3 expression
PIWIL4 expression
Expression
Cluster
Pseudotime
d
High
Low
Expression
LTR12C
LTR12D
LTR12E
SVA_D
AluYa5
LTR10A
LTR40c
LTR46
HERVL40-int
LTR19C
HSATI
ALR/Alpha
BSR/Beta
SST1
D20S16
MLT2B1
MLT1J
MLT2D
MLT1O
MLT1E1

Fig. S3. Transposable Element (TE) Expression Dynamics during Spermatogenesis.

(a) K-means clustering (k=6) of transposable elements (TE) during spermatogenesis.

(b) Expression of LINE, SINE, Satellite and LTR during spermatogenesis.

(c) Expression of *PIWIL* genes during spermatogenesis.

(d) Relative expression levels of selected TEs projected on the tSNE plot. Top row shows examples of TEs highly expressed during early spermatogenic phase; second row shows TEs with expression during middle spermatogenesis; third and last rows show TEs expressed during late spermatogenesis.
