# Supplementary material for: The adult human testis transcriptional cell atlas
Source: Cell Res. 2018 Oct 12;28(12):1141–57. doi: 10.1038/s41422-018-0099-2 (PMC6274646; doi:10.1038/s41422-018-0099-2)
Supplement: Supplementary file 4 — Supplementary information, Figure S4 [file 41422_2018_99_MOESM4_ESM.pdf]

Fig. S4

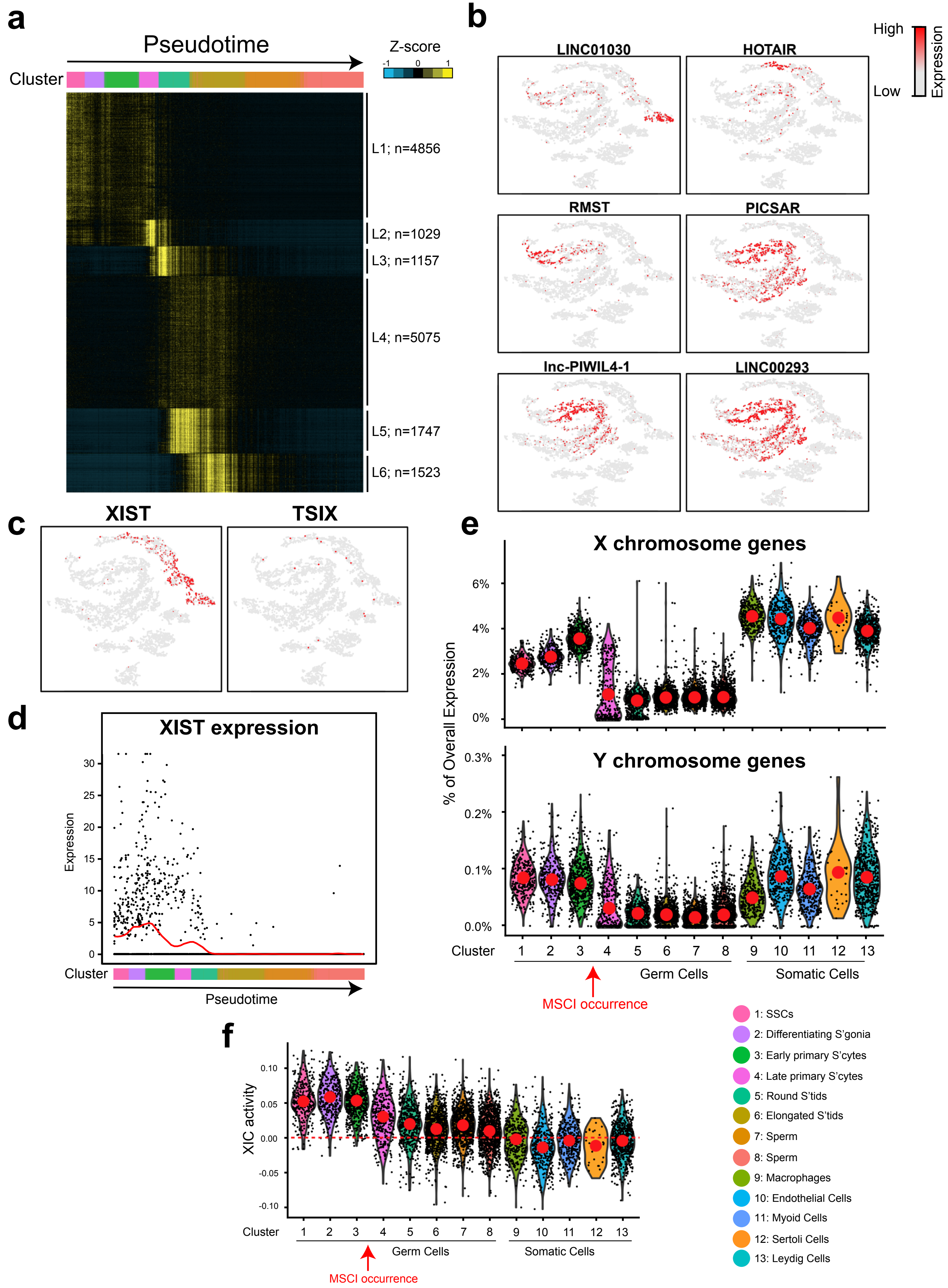

a
Pseudotime
Cluster
Z-score
-1
0
1
L1; n=4856
L2; n=1029
L3; n=1157
L4; n=5075
L5; n=1747
L6; n=1523
b
LINC01030
HOTAIR
RMST
PICSAR
lnc-PIWIL4-1
LINC00293
High
Low
Expression
c
XIST
TSIX
d
XIST expression
Expression
30
25
20
15
10
5
0
Cluster
Pseudotime
e
X chromosome genes
6%
4%
2%
0%
Y chromosome genes
0.3%
0.2%
0.1%
0.0%
% of Overall Expression
Cluster
1
2
3
4
5
6
7
8
9
10
11
12
13
Germ Cells
Somatic Cells
MSCI occurrence
f
XIC activity
0.10
0.05
0.00
-0.05
-0.10
Cluster
Germ Cells
Somatic Cells
MSCI occurrence
1: SSCs
2: Differentiating S'gonia
3: Early primary S'cytes
4: Late primary S'cytes
5: Round S'tids
6: Elongated S'tids
7: Sperm
8: Sperm
9: Macrophages
10: Endothelial Cells
11: Myoid Cells
12: Sertoli Cells
13: Leydig Cells

Fig. S4. Long non-coding RNA Expression Dynamic during Spermatogenesis.

(a) K-means clustering (k=6) of long non-coding RNA (lncRNA) during spermatogenesis.

(b) Relative expression levels of selected lncRNAs projected on the tSNE plot.

(c) Relative expression levels observed for *XIST* (left) and *TSIX* (right) projected on the tSNE plot.

(d) Expression of *XIST* during spermatogenesis. y-axis: normalized expression levels.

(e) Violin plot of relative expression of X (top) or Y (bottom) chromosome genes in different cell clusters. Red dot indicates the median. Note: meiotic sex chromosome inactivation (MSCI) starts at spermatocytes stage (Cluster 3-4 transition).

(f) Violin plot representing level of XCI activity in different cell clusters. Red dot indicates the median. XCI activity is measured as the correlation (spearman) between X chromosome gene expression levels and their distances from XCI, the X inactivation center.
