# Supplementary material for: The adult human testis transcriptional cell atlas
Source: Cell Res. 2018 Oct 12;28(12):1141–57. doi: 10.1038/s41422-018-0099-2 (PMC6274646; doi:10.1038/s41422-018-0099-2)
Supplement: Supplementary file 5 — Supplementary information, Figure S5 [file 41422_2018_99_MOESM5_ESM.pdf]

Fig. S5

a

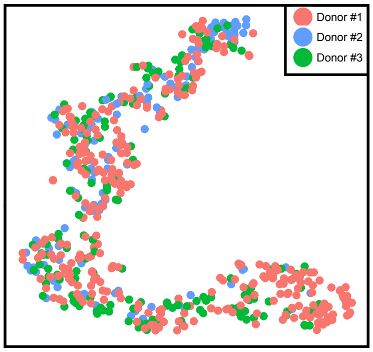

b

State / Donor	0	1	2	3	4	Total
#1	53(18%)	76(25%)	58(19%)	24(8%)	88(29%)	299
#2	48(38%)	41(32%)	24(19%)	5(5%)	6(6%)	128
#3	35(21%)	34(21%)	39(24%)	23(14%)	32(20%)	163
Total	136	151	121	54	128	590

c

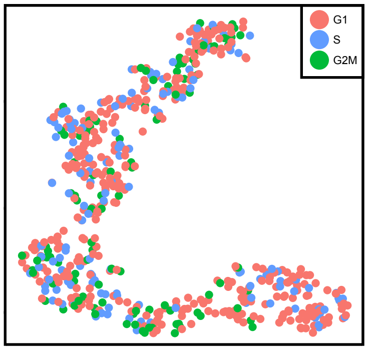

d

Phase / Donor	G1	S	G2/M	Total
#1	186(62%)	76(25%)	37(12%)	299
#2	68(53%)	29(23%)	31(24%)	128
#3	97(60%)	31(19%)	35(21%)	163
Total	351	136	103	590

e

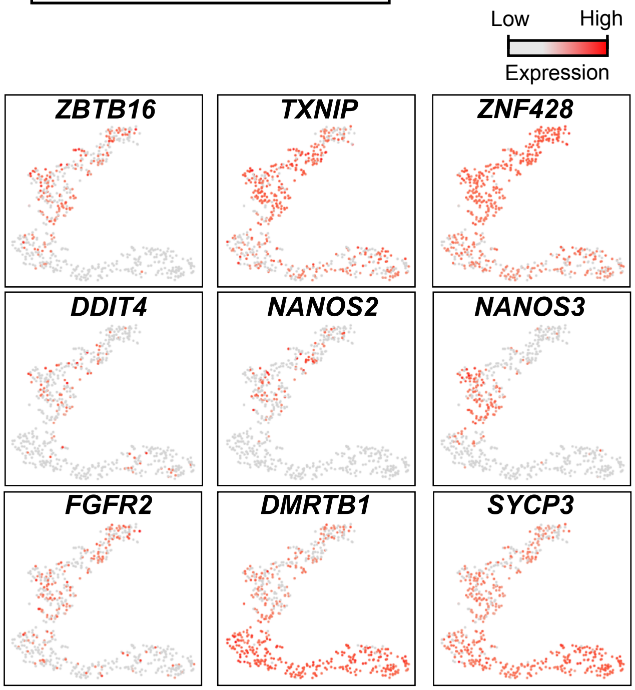

f

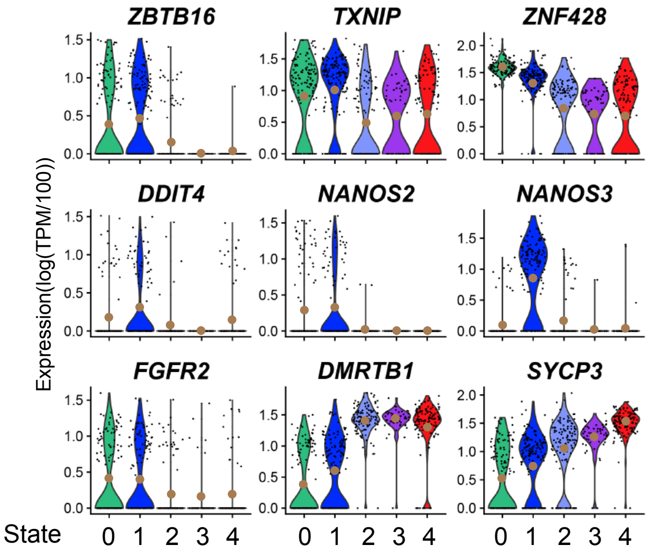

g

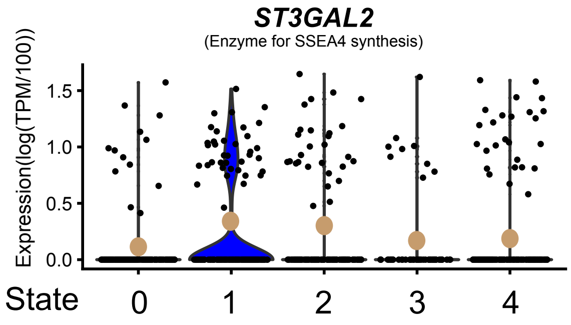

Fig. S5. Identification of a New SSC State.

(a) tSNE plot of spermatogonia (Clusters 1 and 2) with cells colored based on their donors of origin.

(b) Decomposition of states by donor of origin.

(c) tSNE plot of spermatogonia (Clusters 1 and 2) with cells colored based on their cell cycle phase.

(d) Decomposition of cell cycle phase by donor of origin.

(e) Cast expression of additional markers for self-renewing or differentiating markers.

(f) Violin plot of markers in Fig. S5e in different states. y-axis shows normalized expression.

(g) *ST3GAL2*, which encodes the enzyme that synthesizes SSEA4 shows higher expression in State 0.
