# Supplementary material for: The adult human testis transcriptional cell atlas
Source: Cell Res. 2018 Oct 12;28(12):1141–57. doi: 10.1038/s41422-018-0099-2 (PMC6274646; doi:10.1038/s41422-018-0099-2)
Supplement: Supplementary file 6 — Supplementary information, Figure S6 [file 41422_2018_99_MOESM6_ESM.pdf]

Fig. S6

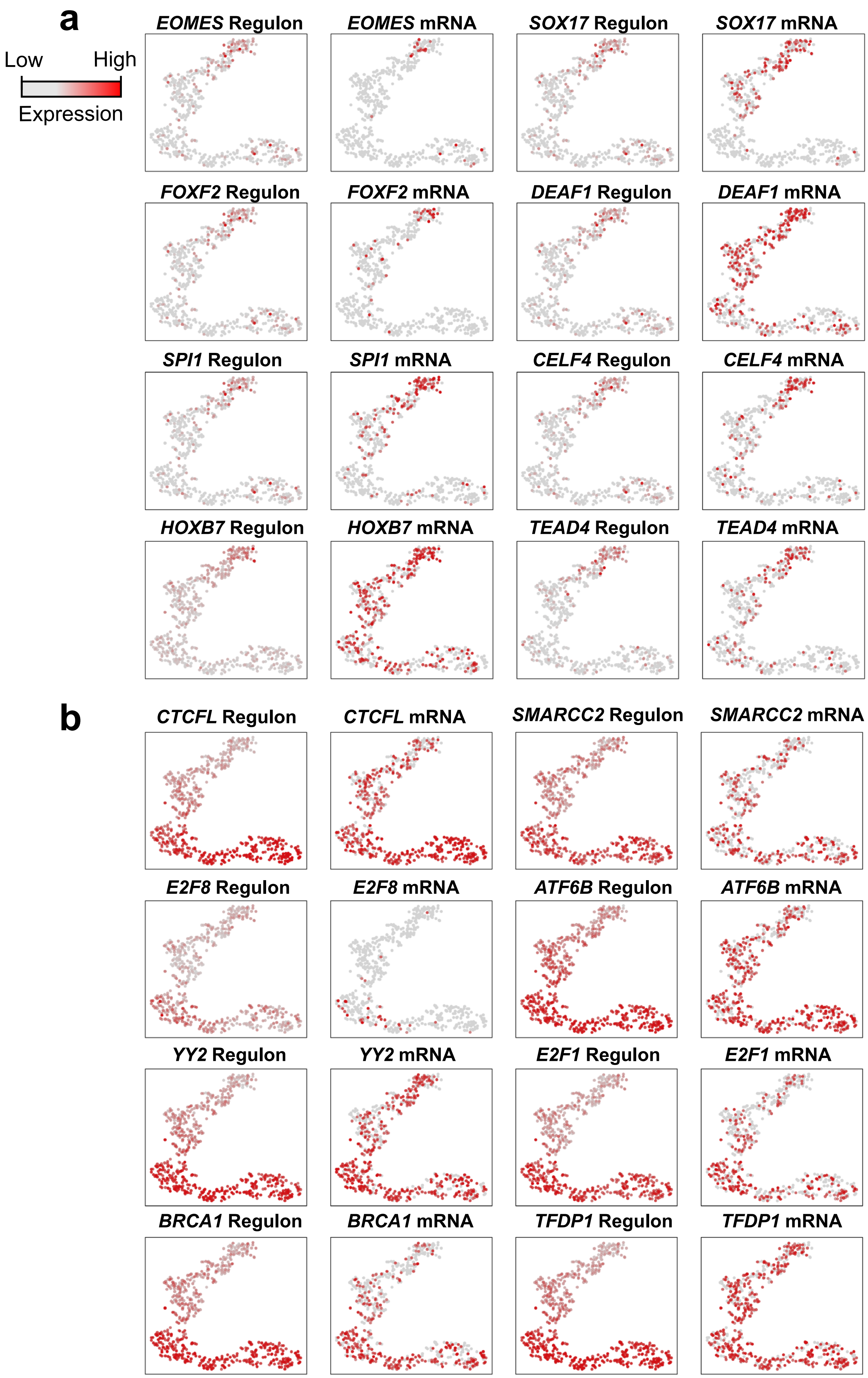
a
Low
High
Expression
EOMES Regulon
EOMES mRNA
SOX17 Regulon
SOX17 mRNA
FOXF2 Regulon
FOXF2 mRNA
DEAF1 Regulon
DEAF1 mRNA
SPI1 Regulon
SPI1 mRNA
CELF4 Regulon
CELF4 mRNA
HOXB7 Regulon
HOXB7 mRNA
TEAD4 Regulon
TEAD4 mRNA
b
CTCFL Regulon
CTCFL mRNA
SMARCC2 Regulon
SMARCC2 mRNA
E2F8 Regulon
E2F8 mRNA
ATF6B Regulon
ATF6B mRNA
YY2 Regulon
YY2 mRNA
E2F1 Regulon
E2F1 mRNA
BRCA1 Regulon
BRCA1 mRNA
TFDP1 Regulon
TFDP1 mRNA

Fig. S6. Regulon Analysis.

(a) Cast expression of self-renewing markers based on regulon activity (first and third columns) or mRNA expression (second and fourth columns).

(b) Cast expression of differentiating markers based on regulon activity (first and third columns) or mRNA expression (second and fourth columns).
