# Supplementary material for: The adult human testis transcriptional cell atlas
Source: Cell Res. 2018 Oct 12;28(12):1141–57. doi: 10.1038/s41422-018-0099-2 (PMC6274646; doi:10.1038/s41422-018-0099-2)
Supplement: Supplementary file 7 — Supplementary information, Figure S7 [file 41422_2018_99_MOESM7_ESM.pdf]

Fig. S7

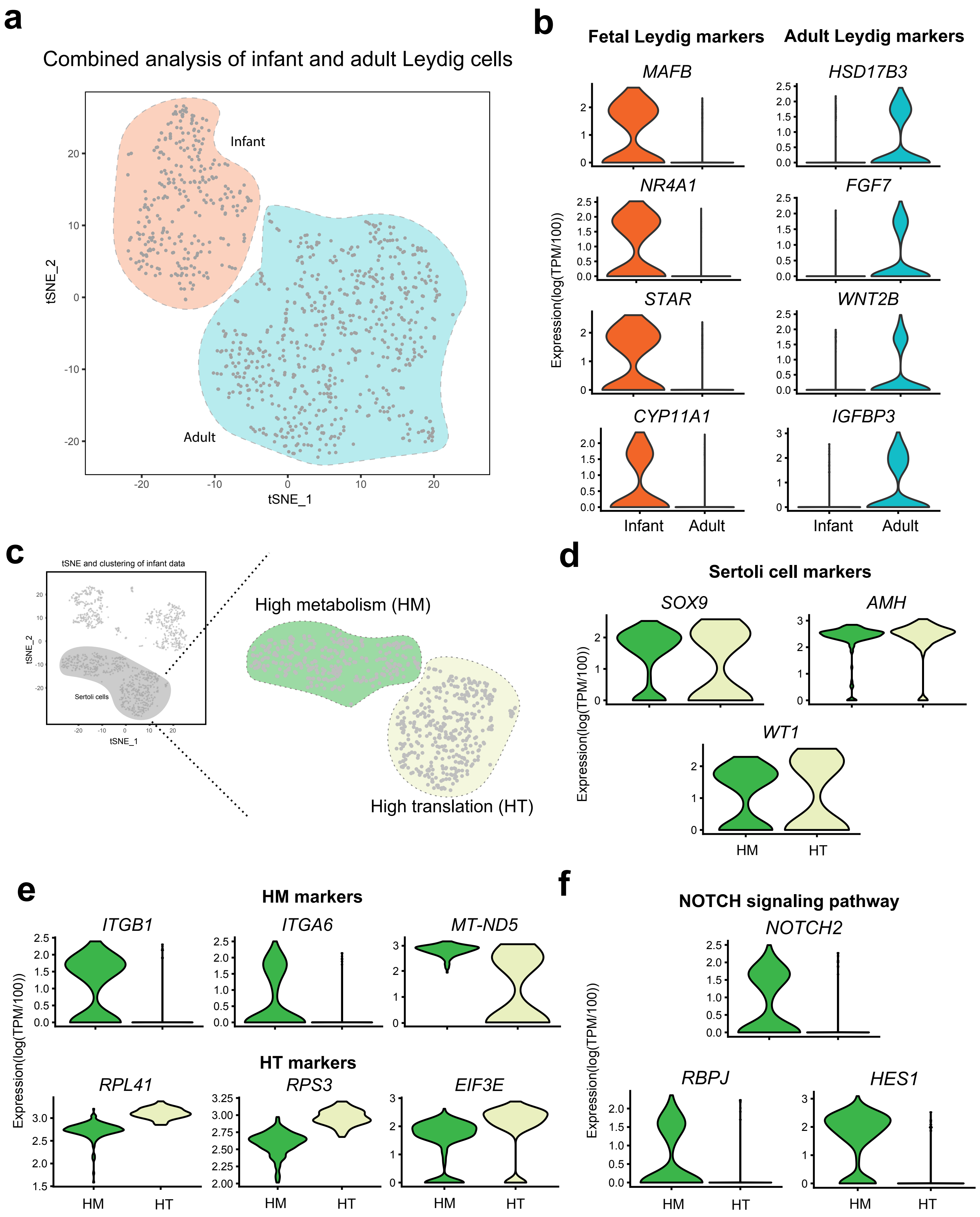
a
Combined analysis of infant and adult Leydig cells
Infant
Adult
tSNE_1
tSNE_2
b
Fetal Leydig markers
Adult Leydig markers
MAFB
HSD17B3
NR4A1
FGF7
STAR
WNT2B
CYP11A1
IGFBP3
Expression(log(TPM/100))
Infant
Adult
c
tSNE and clustering of infant data
Sertoli cells
High metabolism (HM)
High translation (HT)
d
Sertoli cell markers
SOX9
AMH
WT1
HM
HT
e
HM markers
ITGB1
ITGA6
MT-ND5
HT markers
RPL41
RPS3
EIF3E
f
NOTCH signaling pathway
NOTCH2
RBPJ
HES1

Fig. S7. Analysis of Infant Somatic Cells.

(a) tSNE and clustering analysis of infant and adult Leydig cells.

(b) Expression patterns of representative markers in infant and adult Leydig cell clusters in Fig. S7a. Left panel are fetal Leydig cell markers, and right panel are adult Leydig cell markers.

(c) Zoomed-in representation of infant Sertoli cells, which include two distinct clusters (high metabolism and high translation).

(d) Sertoli cell markers are evenly expressed in both Sertoli cell clusters.

(e) Expression of representative genes that are highly expressed in HM or HT Sertoli cells. Top row is HM markers, including different integrins and some mitochondria genes; bottom row is HT markers, including genes that are related to translation.

(f) Notch signaling pathways are active only in HM Sertoli cell clusters.
