# Supplementary material for: The adult human testis transcriptional cell atlas
Source: Cell Res. 2018 Oct 12;28(12):1141–57. doi: 10.1038/s41422-018-0099-2 (PMC6274646; doi:10.1038/s41422-018-0099-2)
Supplement: Supplementary file 8 — Supplementary information, Figure S8 [file 41422_2018_99_MOESM8_ESM.pdf]

Fig. S8

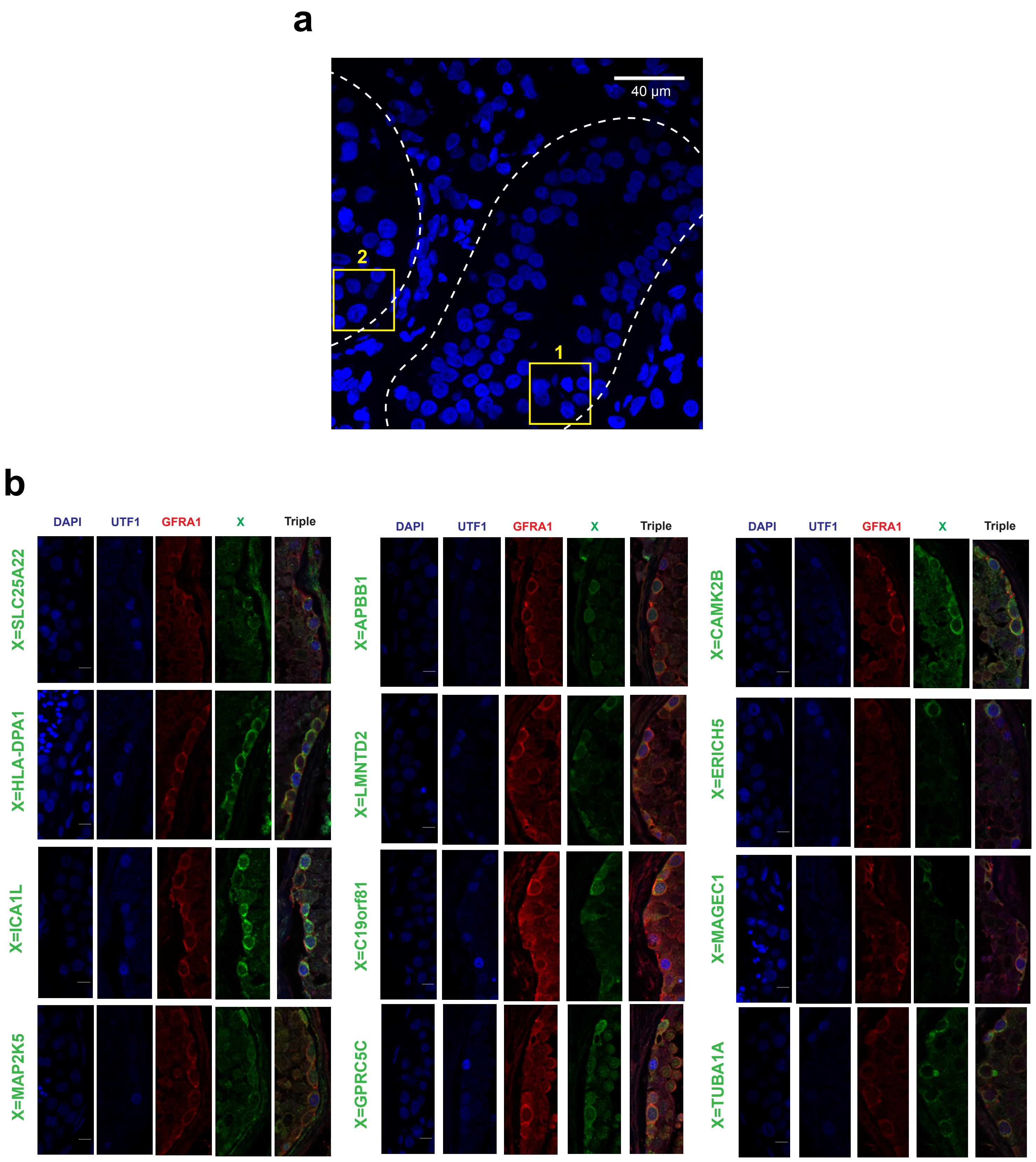
a
40 µm
2
1
b
DAPI
UTF1
GFRA1
X
Triple
X=SLC25A22
X=HLA-DPA1
X=ICA1L
X=MAP2K5
X=APBB1
X=LMNTD2
X=C19orf81
X=GPRC5C
X=CAMK2B
X=ERICH5
X=MAGEC1
X=TUBA1A

Fig. S8. Visualization of RNA FISH and Protein Staining.

(a) Low magnification of the DAPI staining of the testis tubular cross sections represented in Fig. 8a. Yellow boxes highlight the two positions presented in Fig. 8a.

(b) Immunolocalization of UTF1 (State 0 marker, in blue), GFRA1 (State 1 marker, in red) and 12 additional candidate markers (in green). Each antigen (named in green on the left side) is represented by 5 panels. All pictures are at the same magnification and the white bar in the bottom right indicates 10 um.
