# Supplementary material for: The adult human testis transcriptional cell atlas
Source: Cell Res. 2018 Oct 12;28(12):1141–57. doi: 10.1038/s41422-018-0099-2 (PMC6274646; doi:10.1038/s41422-018-0099-2)
Supplement: Supplementary file 9 — Supplementary information, Table S1 [file 41422_2018_99_MOESM9_ESM.pdf]

Supplementary information, Table S1: Summary of Single-cell Dataset and Clustering Information.

CellID	nGene	nUMI	Final_clusters
Donor2-AAACCTGGTGCCTTGG-1	4324	11455	6
Donor2-AAACCTGTCAACGGGA-1	1613	10149	8
Donor2-AAACCTGTCCTATGTT-1	2212	11672	8
Donor2-AAACCTGTCGGACAAG-1	1884	9140	8
Donor2-AAACGGGAGCTATGCT-1	1991	7849	7
Donor2-AAACGGGGTCTGGAGA-1	2094	7793	6
Donor2-AAACGGGTCTCTAAGG-1	2864	16431	7
Donor2-AAATGCCAGTGTTTGC-1	2428	8257	13
Donor2-AAATGCCGTTAGTGGG-1	3147	10459	11
Donor2-AAATGCCGTTCGTCTC-1	1569	5530	8
Donor2-AACACGTTCCACTCCA-1	5156	15884	6
Donor2-AACCATGGTAGCTAAA-1	2991	18074	7
Donor2-AACCGCGGTAAATGAC-1	6420	28397	6
Donor2-AACGTTGAGGACACCA-1	2449	9271	9
Donor2-AACTCCCAGGAGCGTT-1	2156	9099	9
Donor2-AACTCCCAGTGATCGG-1	1893	11301	8
Donor2-AACTCCCTCTGTACGA-1	1262	5587	8
Donor2-AACTCTTCAGTTAACC-1	5988	23018	5
Donor2-AACTCTTGTCAATGTC-1	2752	12092	6
Donor2-AACTGGTCATCACAAC-1	2049	6587	13
Donor2-AACTTTCAGACCTTTG-1	1873	5672	11
Donor2-AACTTTCGTGCCTGGT-1	1848	6664	7
Donor2-AACTTTCGTTCCCTTG-1	2220	8363	10
Donor2-AAGCCGCAGCGATTCT-1	2978	13935	10
Donor2-AAGCCGCCATACGCCG-1	2023	5707	11
Donor2-AAGCCGCGTCGAATCT-1	2744	9674	6
Donor2-AAGCCGCTCGGCGCTA-1	3216	12478	7
Donor2-AAGGAGCTCGCCTGTT-1	2579	7375	11
Donor2-AAGGCAGGTTGCGTTA-1	3884	11157	3
Donor2-AAGGCAGTCTACTTAC-1	2119	12746	8
Donor2-AAGGTTCAGCGATTCT-1	5815	20037	1
Donor2-AAGGTTCCACGAAATA-1	5525	19266	3
Donor2-AAGGTTCGTGGTCCGT-1	9081	70146	6
Donor2-AAGTCTGCAATACGCT-1	5116	15712	6
Donor2-AATCCAGCACTTCTGC-1	2418	7933	6
Donor2-AATCCAGCATCGGAAG-1	8141	48409	5
Donor2-AATCCAGTCTCCCTGA-1	7157	37640	1
Donor2-AATCCAGTCTTCTGGC-1	1545	5475	9
Donor2-AATCGGTAGGGTCTCC-1	1769	5713	13
Donor2-AATCGGTGTAGATTAG-1	3093	19462	7
Donor2-ACACCAACAATGGTCT-1	2782	6118	8
Donor2-ACACCAACACGCATCG-1	2073	12311	8
Donor2-ACACCCTCAGTATCTG-1	2201	8173	9
Donor2-ACACCCTTCAGCTCTC-1	2091	8157	9
Donor2-ACACCGGTCGGTGTTA-1	2418	14780	7
Donor2-ACACTGAAGGCATGTG-1	9232	57764	3
Donor2-ACACTGAAGGTAGCCA-1	5510	19524	6
Donor2-ACACTGACACCTCGTT-1	6473	26527	5
Donor2-ACACTGATCTTCTGGC-1	1845	6337	9
Donor2-ACAGCTAAGACAAAGG-1	4969	19823	6
Donor2-ACAGCTAGTTTGCATG-1	2209	13313	7
Donor2-ACATCAGGTAAAGGAG-1	3111	6701	1
Donor2-ACATCAGGTCAAACTC-1	4917	15474	5
Donor2-ACATCAGGTTAAGATG-1	2938	8546	11

Donor2-ACATCAGGTTCGTGAT-1 3207 10577 11
Donor2-ACATGGTAGCAGATCG-1 5246 16359 5
Donor2-ACATGGTGTGGGTCAA-1 2727 15510 7
Donor2-ACCAGTAAGATAGCAT-1 3623 12766 11
Donor2-ACCAGTAAGCTAACAA-1 5231 15382 3
Donor2-ACCAGTAAGTGTCCAT-1 6015 30589 6
Donor2-ACCAGTAGTGTCTGAT-1 2376 5699 8
Donor2-ACCCACTAGACCACGA-1 7690 44124 6
Donor2-ACCGTAAAGATAGGAG-1 1861 7529 7
Donor2-ACCGTAAAGGGTTCCC-1 1649 9052 8
Donor2-ACCGTAATCCAAACTG-1 4506 12183 6
Donor2-ACCGTAATCTAACTTC-1 7217 33176 6
Donor2-ACCTTTATCGGAAATA-1 2220 5967 11
Donor2-ACGAGCCGTGCATCTA-1 2230 9596 9
Donor2-ACGAGGAAGAAGAAGC-1 2558 10584 7
Donor2-ACGAGGAAGCTCAACT-1 1947 8009 13
Donor2-ACGAGGACAGCCTTTC-1 3290 17397 7
Donor2-ACGAGGACAGTCAGAG-1 2982 9968 6
Donor2-ACGAGGACAGTCGATT-1 2750 13498 6
Donor2-ACGAGGACATCCCATC-1 2784 10200 13
Donor2-ACGAGGAGTCGACTGC-1 4554 12487 5
Donor2-ACGAGGATCCGCATAA-1 6137 26017 5
Donor2-ACGATACAGCCAGTTT-1 1654 7153 7
Donor2-ACGATACAGGACACCA-1 4057 20929 6
Donor2-ACGATACTCAAACCGT-1 1872 8484 7
Donor2-ACGATGTAGCTAAACA-1 2856 11740 10
Donor2-ACGATGTCAAGTTCTG-1 3772 17158 9
Donor2-ACGCCAGTCTGAAAGA-1 3923 11199 5
Donor2-ACGCCGAAGCTAACAA-1 5388 23181 6
Donor2-ACGCCGAGTGATGATA-1 4037 23340 6
Donor2-ACGCCGAGTTACCAGT-1 1586 5519 7
Donor2-ACGCCGATCTTATCTG-1 7795 44545 6
Donor2-ACGGAGACAACGATGG-1 4190 23869 7
Donor2-ACGGAGAGTGTCTGAT-1 5169 15777 6
Donor2-ACGGCCATCCATGAGT-1 3014 6877 3
Donor2-ACGGCCATCCCAGGTG-1 4371 17730 6
Donor2-ACGGGCTAGTGGACGT-1 2609 11076 10
Donor2-ACGGGCTGTTCTGGTA-1 2081 10815 7
Donor2-ACGGGCTTCGATCCCT-1 2816 8080 5
Donor2-ACGGGCTTCTTACCTA-1 2511 13187 7
Donor2-ACGGGTCAGTGCAAGC-1 3548 9042 4
Donor2-ACGTCAACAACACCCG-1 1450 5859 13
Donor2-ACTATCTGTTATGTGC-1 2046 6331 11
Donor2-ACTGAACCATGCCACG-1 1756 7473 7
Donor2-ACTGAACTCACTCCTG-1 8734 64301 6
Donor2-ACTGAACTCGTTTAGG-1 4484 11962 6
Donor2-ACTGAGTTCAGTACGT-1 7743 42267 6
Donor2-ACTGAGTTCCAGAGGA-1 1266 6202 8
Donor2-ACTGAGTTCTCTGAGA-1 5681 30322 6
Donor2-ACTGATGAGTACACCT-1 3566 26645 7
Donor2-ACTGCTCTCGTCTGCT-1 6311 34957 4
Donor2-ACTGCTCTCTTTAGGG-1 4270 12483 4
Donor2-ACTGTCCAGAGTAATC-1 2693 11815 8
Donor2-ACTGTCCAGCGTGAGT-1 1715 7809 8
Donor2-ACTGTCCCAAGCCCAC-1 1371 6365 8
Donor2-ACTGTCCGTTCGGCAC-1 6701 31637 5

Donor2-ACTGTCCTCTTCCTTC-1 6326 23557 3
Donor2-ACTTACTGTGCGGTAA-1 1847 9218 8
Donor2-ACTTGTTAGGATGCGT-1 2309 6924 11
Donor2-ACTTGTTGTCGGCACT-1 1754 7316 7
Donor2-ACTTTCACAATCCAAC-1 4104 16110 8
Donor2-ACTTTCACATCGGGTC-1 1479 6439 8
Donor2-ACTTTCATCCGTACAA-1 6355 34537 5
Donor2-AGAATAGAGCCAGTTT-1 3389 13940 6
Donor2-AGAATAGTCAAAGTAG-1 1441 7834 8
Donor2-AGACGTTCAGCCTTGG-1 3594 12085 11
Donor2-AGACGTTCAGGTCCAC-1 3823 26454 8
Donor2-AGACGTTTCAGGCCCA-1 2237 11930 7
Donor2-AGACGTTTCGATCCCT-1 2201 8645 7
Donor2-AGAGCGAAGATGGGTC-1 6003 30982 1
Donor2-AGAGCGAAGCTGTTCA-1 3416 17836 7
Donor2-AGAGCGAAGTATGACA-1 2111 5835 11
Donor2-AGAGTGGAGAGGTTGC-1 4693 18024 6
Donor2-AGAGTGGAGTGCGATG-1 2975 10759 10
Donor2-AGATCTGAGCCCAGCT-1 5596 23044 5
Donor2-AGATCTGCAGCAGTTT-1 6239 41091 6
Donor2-AGATCTGGTTCACGGC-1 1855 5550 13
Donor2-AGATTGCAGGAGTACC-1 2649 5845 3
Donor2-AGATTGCAGGAGTTTA-1 3445 8826 3
Donor2-AGATTGCCACCGAATT-1 2327 6463 11
Donor2-AGATTGCTCATGCTCC-1 3269 7164 5
Donor2-AGATTGCTCCGTACAA-1 2075 12072 8
Donor2-AGCAGCCCAAAGCAAT-1 2526 7214 11
Donor2-AGCATACAGACGCACA-1 8550 55474 5
Donor2-AGCATACCACCTATCC-1 5416 28518 6
Donor2-AGCATACTCTAAGCCA-1 4888 13839 6
Donor2-AGCCTAAAGTTCCACA-1 2890 9636 11
Donor2-AGCCTAACAATAGCGG-1 3052 16640 7
Donor2-AGCGGTCAGAAACGAG-1 3009 12801 10
Donor2-AGCGTATCAAGCCCAC-1 2806 8357 13
Donor2-AGCGTATGTTTGTTTC-1 1712 6697 13
Donor2-AGCGTCGAGTGACATA-1 2202 5453 13
Donor2-AGCGTCGCATCACCCT-1 6607 34489 6
Donor2-AGCGTCGGTAGGACAC-1 4201 11976 5
Donor2-AGCGTCGTCTGGCGAC-1 6331 35076 5
Donor2-AGCTCCTCACTTAACG-1 3705 28889 7
Donor2-AGCTCTCTCGCGGATC-1 2912 5757 6
Donor2-AGCTCTCTCTACGAGT-1 3716 20130 6
Donor2-AGCTTGAAGTTACCCA-1 3086 22830 8
Donor2-AGGGAGTCAGTGAGTG-1 5277 16217 8
Donor2-AGGGAGTGTAGAGGAA-1 2056 8783 7
Donor2-AGGGATGCAAACCCAT-1 3165 10549 6
Donor2-AGGGATGCACGGCTAC-1 5868 21797 6
Donor2-AGGGATGGTTCGTGAT-1 1770 6174 13
Donor2-AGGGTGATCTACTCAT-1 7007 48986 4
Donor2-AGGGTGATCTGTCTCG-1 4370 13646 6
Donor2-AGGTCATAGTACTTGC-1 1274 6529 8
Donor2-AGGTCATGTGAGGGAG-1 2277 7958 10
Donor2-AGGTCATTCTCGAGTA-1 2127 7968 13
Donor2-AGTAGTCAGACGCAAC-1 5552 23191 5
Donor2-AGTAGTCAGCAGCCTC-1 2355 14845 8
Donor2-AGTAGTCCATCGGACC-1 6294 24951 5

Donor2-AGTAGTCTCCAAACAC-1 8765 61974 6
Donor2-AGTCTTTAGGCCCGTT-1 6098 45397 10
Donor2-AGTCTTTAGTCCATAC-1 7008 56303 6
Donor2-AGTCTTTCAGAGTGTG-1 6040 45392 10
Donor2-AGTCTTTCAGCTCCGA-1 2051 7503 9
Donor2-AGTCTTTTCCCACTTG-1 5771 43485 6
Donor2-AGTGAGGAGTACGTAA-1 2300 9683 7
Donor2-AGTGAGGCAGCTGTGC-1 6725 51062 6
Donor2-AGTGGGAAGACACGAC-1 5665 26943 6
Donor2-AGTGGGATCGCAAGCC-1 2755 8236 11
Donor2-AGTGTCAGTGAGTATA-1 2517 12579 7
Donor2-AGTTGGTAGTATCTCG-1 1553 5555 13
Donor2-ATAACGCAGGTAGCCA-1 4212 28522 7
Donor2-ATAACGCTCACGATGT-1 5674 55918 7
Donor2-ATAACGCTCTACCAGA-1 1977 10019 8
Donor2-ATAAGAGAGCGGATCA-1 6759 44010 6
Donor2-ATAAGAGGTAAATGAC-1 3168 17716 7
Donor2-ATAGACCAGGTACTCT-1 1503 5405 13
Donor2-ATAGACCCAAGCCATT-1 4536 12292 2
Donor2-ATAGACCCAATTCCTT-1 4035 9589 2
Donor2-ATCATCTAGTAGGTGC-1 1564 5823 7
Donor2-ATCATCTGTGCTAGCC-1 3475 8224 3
Donor2-ATCATGGTCACAATGC-1 4179 17603 8
Donor2-ATCCACCGTCGAACAG-1 2530 5561 5
Donor2-ATCCACCTCTTCAACT-1 1837 7489 9
Donor2-ATCCGAAAGTACACCT-1 7457 42029 5
Donor2-ATCCGAATCCACGTGG-1 4156 18847 6
Donor2-ATCGAGTAGAAACCTA-1 2768 11147 8
Donor2-ATCTACTCAGCTGTTA-1 5535 21179 1
Donor2-ATCTACTCAGTATCTG-1 6538 29045 6
Donor2-ATCTGCCAGATACACA-1 9679 83399 6
Donor2-ATCTGCCCAAAGTGCG-1 7638 41381 5
Donor2-ATCTGCCCACGTGAGA-1 2344 7937 10
Donor2-ATCTGCCCAGCTGGCT-1 1904 11063 8
Donor2-ATGAGGGCAAAGGCGT-1 1806 6304 9
Donor2-ATGAGGGGTAACGTTC-1 3600 14020 7
Donor2-ATGCGATCATGGTCTA-1 4723 42853 7
Donor2-ATGTGTGAGTGTTGAA-1 2299 8916 6
Donor2-ATTATCCGTTTGCATG-1 3896 23642 6
Donor2-ATTATCCTCAAGGTAA-1 6851 30420 5
Donor2-ATTCTACCAAGGACAC-1 3066 15813 7
Donor2-ATTGGACGTATAGGGC-1 2988 9677 11
Donor2-ATTGGTGTCAAGGCTT-1 1479 6650 8
Donor2-ATTTCTGCAATGTAAG-1 1905 6467 7
Donor2-ATTTCTGCATCGATTG-1 5360 19307 4
Donor2-ATTTCTGGTGTTCTTT-1 1535 8964 8
Donor2-CAACTAGAGAGGTACC-1 7450 35360 6
Donor2-CAAGAAACAGCTTAAC-1 4652 13591 5
Donor2-CAAGAAATCCCATTAT-1 3263 7818 6
Donor2-CAAGAAATCGATCCCT-1 2586 8229 13
Donor2-CAAGAAATCTAACTCT-1 3049 6331 2
Donor2-CAAGATCAGATAGTCA-1 3422 12733 5
Donor2-CAAGATCCATACTACG-1 3921 15525 8
Donor2-CAAGATCCATATGCTG-1 2100 6149 11
Donor2-CAAGGCCCAGTCACTA-1 4745 24485 6
Donor2-CAAGTTGAGAACTCGG-1 7112 35866 5

Donor2-CACAAACAGACTGTAA-1 9090 52641 6
Donor2-CACACAAAGCAGATCG-1 2196 5538 11
Donor2-CACACAAGTACAGACG-1 3902 19604 10
Donor2-CACACAAGTGGGTCAA-1 5424 14679 6
Donor2-CACACCTAGATGTAAC-1 3172 10652 11
Donor2-CACACCTAGATGTGTA-1 2664 11767 12
Donor2-CACACCTCAAGGTTTC-1 1274 7010 8
Donor2-CACAGGCTCCAAAGTC-1 4356 18310 7
Donor2-CACAGGCTCCACGCAG-1 1687 5871 9
Donor2-CACAGGCTCTAACTTC-1 5036 15564 6
Donor2-CACAGTAAGTCGTACT-1 1692 7407 8
Donor2-CACAGTAGTTCAGACT-1 2435 17896 8
Donor2-CACAGTATCCCGACTT-1 1340 5499 8
Donor2-CACAGTATCGAGCCCA-1 3914 13209 6
Donor2-CACATAGCATACTCTT-1 2093 7239 13
Donor2-CACATAGGTTATGTGC-1 3350 9786 2
Donor2-CACATAGTCGGTTAAC-1 5390 19452 4
Donor2-CACATTTAGCGATCCC-1 3148 7838 6
Donor2-CACCAGGAGGTTACCT-1 1429 7180 8
Donor2-CACCAGGCATGAGCGA-1 6567 47216 5
Donor2-CACCTTGCATCCCACT-1 4277 12694 5
Donor2-CACTCCATCATGCAAC-1 1781 6386 8
Donor2-CAGAATCAGACAGAGA-1 2007 7856 9
Donor2-CAGAATCAGCGGATCA-1 2729 12139 7
Donor2-CAGAATCAGTGGACGT-1 1335 5643 7
Donor2-CAGAATCAGTGGTCCC-1 2508 7210 11
Donor2-CAGAATCGTGAAAGAG-1 1788 6630 13
Donor2-CAGAGAGGTAAATACG-1 1671 6036 13
Donor2-CAGAGAGGTCTTGATG-1 4475 11945 6
Donor2-CAGATCAAGGGTCGAT-1 2425 8194 13
Donor2-CAGATCACACAACTGT-1 1762 5776 13
Donor2-CAGATCAGTCAGAAGC-1 2067 7087 10
Donor2-CAGATCATCCGTACAA-1 1677 9199 8
Donor2-CAGCAGCTCACAGTAC-1 3775 9516 3
Donor2-CAGCATAGTCGAAAGC-1 2292 9167 6
Donor2-CAGCATAGTCGCTTTC-1 1200 5940 8
Donor2-CAGCCGAAGAATAGGG-1 5640 50498 7
Donor2-CAGCCGAAGAATTCCC-1 2141 7718 13
Donor2-CAGCCGAAGCCTATGT-1 1721 5761 9
Donor2-CAGCCGAAGGCTAGCA-1 1757 9420 8
Donor2-CAGCCGAGTCACTGGC-1 3480 10097 5
Donor2-CAGCCGAGTCGTCTTC-1 3575 19767 6
Donor2-CAGCCGAGTTCCACGG-1 4344 15473 6
Donor2-CAGCGACAGACGACGT-1 2460 11323 10
Donor2-CAGCGACGTGTTGGGA-1 2825 12727 7
Donor2-CAGCTAAAGATGCCAG-1 1884 6238 10
Donor2-CAGCTGGGTATAGTAG-1 6692 41305 6
Donor2-CAGCTGGTCACAAACC-1 7169 29210 4
Donor2-CAGGTGCCAATCCAAC-1 5445 18813 5
Donor2-CAGGTGCCAGACACTT-1 1842 5667 13
Donor2-CAGGTGCTCAACGGGA-1 1897 6437 13
Donor2-CAGTAACGTACAAGTA-1 1757 10230 8
Donor2-CAGTAACGTAGGGACT-1 1919 7193 9
Donor2-CAGTAACGTCAACATC-1 1904 6527 10
Donor2-CAGTCCTAGAGTAAGG-1 2300 10595 7
Donor2-CAGTCCTTCAGTGTTG-1 6773 44777 5

Donor2-CATATGGCAATGGAGC-1 1575 7299 8
Donor2-CATATTCAGACCGGAT-1 5978 30805 6
Donor2-CATATTCTCAACGAAA-1 3148 12861 10
Donor2-CATATTCTCGGCGCAT-1 5981 26200 6
Donor2-CATCAAGCAAGTCTAC-1 1782 6431 9
Donor2-CATCAAGTCGTACCGG-1 1773 8592 7
Donor2-CATCAAGTCTGGTGTA-1 5476 18462 6
Donor2-CATCAGAAGTACGATA-1 1244 6242 8
Donor2-CATCAGACACAGATTC-1 6889 37462 5
Donor2-CATCAGAGTTACTGAC-1 2700 7849 11
Donor2-CATCAGAGTTTAAGCC-1 2780 10125 7
Donor2-CATCCACAGACGACGT-1 1713 6213 13
Donor2-CATCCACAGGTGCTAG-1 2699 8002 4
Donor2-CATCCACTCGGTCCGA-1 1719 6676 9
Donor2-CATCGGGCAACTGCTA-1 6117 23556 5
Donor2-CATCGGGTCTTGTATC-1 2914 6665 6
Donor2-CATGCCTGTATAAACG-1 2067 7496 13
Donor2-CATGGCGCACGGCGTT-1 2318 6548 11
Donor2-CATGGCGCATCTCGCT-1 2871 12357 7
Donor2-CATGGCGGTACCGCTG-1 2015 8346 7
Donor2-CATTATCAGAGTCTGG-1 2164 8961 6
Donor2-CATTATCAGCAGGTCA-1 4400 12258 4
Donor2-CATTCGCCAAGTCTAC-1 1868 6792 10
Donor2-CATTCGCGTTCGTCTC-1 4115 10225 3
Donor2-CATTCGCTCACTTACT-1 3032 27139 8
Donor2-CCAATCCAGCAGATCG-1 5480 17671 1
Donor2-CCAATCCAGTGGGTTG-1 5265 18550 1
Donor2-CCAATCCCATACGCCG-1 3185 10879 11
Donor2-CCAATCCGTCCATGAT-1 6153 24637 6
Donor2-CCAATCCTCGGAAATA-1 5853 23294 4
Donor2-CCACCTAAGAACAATC-1 7372 61223 6
Donor2-CCACCTAAGTAAGTAC-1 1395 6226 8
Donor2-CCACCTACAATCTGCA-1 4658 27810 6
Donor2-CCACCTAGTTACTGAC-1 9424 84458 4
Donor2-CCACGGACAATTGCTG-1 1953 5653 11
Donor2-CCACTACAGTTGTCGT-1 1852 7246 13
Donor2-CCACTACCACGGTTTA-1 2421 11290 7
Donor2-CCACTACTCTTGCAAG-1 2091 7094 13
Donor2-CCATTCGCAGACAAGC-1 4309 17493 6
Donor2-CCATTCGGTACTCTCC-1 6930 48656 4
Donor2-CCATTCGGTCTTTCAT-1 1946 7134 9
Donor2-CCATTCGTCCGAACGC-1 3331 7748 3
Donor2-CCCAATCAGTTCGATC-1 1816 6354 9
Donor2-CCCAATCCACATCCAA-1 3260 10079 11
Donor2-CCCAATCTCAGGTTCA-1 1567 5508 13
Donor2-CCCAGTTCATATACGC-1 1334 7025 8
Donor2-CCCAGTTGTGCAACGA-1 1506 7136 8
Donor2-CCCAGTTGTGCGCTTG-1 5582 20379 5
Donor2-CCCATACAGGTTACCT-1 2018 10173 8
Donor2-CCCATACGTACGCACC-1 2374 10137 7
Donor2-CCCTCCTAGCTATGCT-1 3028 6259 8
Donor2-CCCTCCTCATATACGC-1 2368 11228 7
Donor2-CCCTCCTGTACTTGAC-1 2875 10657 7
Donor2-CCCTCCTTCAGCTCGG-1 2196 7783 13
Donor2-CCGGGATCAGGGCATA-1 2872 13409 7
Donor2-CCGGGATGTAGCCTCG-1 1630 7908 8

Donor2-CCGGGATGTTTCCACC-1 2636 7786 11
Donor2-CCGGTAGCAGAGCCAA-1 1534 6075 8
Donor2-CCGGTAGCAGATGGGT-1 2178 12346 7
Donor2-CCGTACTAGCACAGGT-1 7848 41176 6
Donor2-CCGTACTGTAACGCGA-1 6608 24792 5
Donor2-CCGTACTGTTCAACCA-1 2591 7648 11
Donor2-CCGTACTTCAATCACG-1 1613 5674 7
Donor2-CCGTGGAAGTCATCCA-1 1738 9543 8
Donor2-CCGTGGAGTGTGTGCC-1 2241 8838 7
Donor2-CCGTTCAAGGAATTAC-1 5186 20084 6
Donor2-CCGTTCAAGGCGCTCT-1 2157 6009 11
Donor2-CCGTTCAAGGCTCAGA-1 4328 20950 7
Donor2-CCGTTCAAGGGAGTAA-1 5377 23851 5
Donor2-CCGTTCACATCCTTGC-1 6733 31192 6
Donor2-CCTAGCTAGAATTCCC-1 4071 10701 4
Donor2-CCTAGCTGTGGCAAAC-1 1506 7375 8
Donor2-CCTAGCTGTTTGCATG-1 2272 13768 8
Donor2-CCTAGCTTCACTTCAT-1 5256 26311 6
Donor2-CCTATTACACGGCGTT-1 1628 5780 9
Donor2-CCTATTAGTAGTAGTA-1 2492 9494 10
Donor2-CCTCAGTGTGATGTGG-1 8442 72362 4
Donor2-CCTCTGAGTGCACTTA-1 3698 16468 7
Donor2-CCTTACGCACGGTTTA-1 3185 10339 11
Donor2-CCTTACGGTCCAAGTT-1 5392 17163 1
Donor2-CCTTACGGTTAAGTAG-1 4096 14727 6
Donor2-CCTTACGGTTATGTGC-1 4735 14280 5
Donor2-CCTTCGATCTAGCACA-1 1695 5985 8
Donor2-CCTTTCTCACTGAAGG-1 3829 9691 3
Donor2-CCTTTCTGTCTGGAGA-1 1981 10360 8
Donor2-CCTTTCTGTTGCTCCT-1 4903 14854 6
Donor2-CCTTTCTTCCTGTAGA-1 4987 23720 6
Donor2-CGAACATCACCCTATC-1 4605 12545 2
Donor2-CGAACATCATCCGTGG-1 1330 5553 13
Donor2-CGAATGTAGAGACTTA-1 2677 6743 2
Donor2-CGAATGTAGGGTGTTG-1 4630 13025 6
Donor2-CGAATGTCAGACACTT-1 1805 10448 8
Donor2-CGAATGTGTGAGTATA-1 2467 6212 8
Donor2-CGAATGTTCGGCGCTA-1 2146 6487 11
Donor2-CGACCTTCAATGAATG-1 7013 35058 6
Donor2-CGACCTTCATGTAGTC-1 1824 6407 13
Donor2-CGACCTTTCATAGCAC-1 2246 9775 7
Donor2-CGAGAAGAGAAGCCCA-1 2560 8906 7
Donor2-CGAGAAGCAATTGCTG-1 1838 6375 13
Donor2-CGAGCACCACATTCGA-1 2410 9992 13
Donor2-CGAGCACGTGGCAAAC-1 1544 7672 8
Donor2-CGAGCCATCTTGTATC-1 2017 7978 13
Donor2-CGATCGGCACGACTCG-1 5250 24281 6
Donor2-CGATCGGGTTAGAACA-1 1405 6787 8
Donor2-CGATGTACAGCTCGCA-1 3183 9525 8
Donor2-CGATGTAGTATATGGA-1 4549 30189 6
Donor2-CGATGTATCGCCAGCA-1 2108 5400 10
Donor2-CGATTGAAGTACGACG-1 2995 5755 6
Donor2-CGATTGACACAAGCCC-1 3566 8866 8
Donor2-CGCCAAGAGCTCTCGG-1 1888 6302 9
Donor2-CGCCAAGAGGTGACCA-1 2446 11651 7
Donor2-CGCGGTACATACTACG-1 1735 5913 9

Donor2-CGCGGTAGTAAGCACG-1 2235 5855 11
Donor2-CGCGGTAGTAGTACCT-1 1819 9968 8
Donor2-CGCGGTATCACGAAGG-1 2272 17642 8
Donor2-CGCTATCCAGTTCCCT-1 1908 5897 7
Donor2-CGCTATCGTAGCTTGT-1 1658 5804 13
Donor2-CGCTATCTCCTAGGGC-1 1470 6234 13
Donor2-CGCTGGACAATCCGAT-1 2093 8399 7
Donor2-CGCTGGACATATGGTC-1 2013 6925 9
Donor2-CGCTGGAGTCGATTGT-1 2026 7651 9
Donor2-CGCTGGAGTTAAGTAG-1 2263 13665 7
Donor2-CGCTTCACAATCCGAT-1 1811 8338 7
Donor2-CGCTTCACATCACAAC-1 2224 8023 10
Donor2-CGGACACGTCTGCGGT-1 6472 31045 6
Donor2-CGGACACGTGATGCCC-1 2914 9398 11
Donor2-CGGACGTAGAAACCGC-1 1709 5798 13
Donor2-CGGACGTAGCCCAGCT-1 6400 32564 4
Donor2-CGGACGTTCACTTCAT-1 6229 27006 6
Donor2-CGGACTGAGAGTCGGT-1 7050 33908 5
Donor2-CGGACTGCAGGTGCCT-1 5950 44344 11
Donor2-CGGACTGGTTGCTCCT-1 4553 23351 6
Donor2-CGGAGCTCATTACCTT-1 3135 21382 8
Donor2-CGGAGCTGTTAGAACA-1 2849 5503 5
Donor2-CGGAGTCAGATAGCAT-1 3029 22093 7
Donor2-CGGAGTCGTCTTTCAT-1 3133 16931 7
Donor2-CGGAGTCGTGATGCCC-1 1755 7315 7
Donor2-CGGCTAGAGGGAACGG-1 1704 8509 7
Donor2-CGGCTAGAGTGACTCT-1 5727 20455 5
Donor2-CGGGTCAGTCATACTG-1 1751 8068 8
Donor2-CGGGTCATCGGACAAG-1 3172 11187 13
Donor2-CGGTTAAGTCAACTGT-1 1433 7080 8
Donor2-CGGTTAAGTTCCGTCT-1 1621 5716 13
Donor2-CGGTTAATCAGGTAAA-1 2499 8950 9
Donor2-CGTAGCGAGCAAATCA-1 7192 40764 4
Donor2-CGTAGCGAGCGTGAGT-1 2709 5388 6
Donor2-CGTAGGCAGCATGGCA-1 1719 8766 8
Donor2-CGTCAGGAGAGGTTAT-1 6581 21635 1
Donor2-CGTCAGGCAACACCCG-1 5192 25204 5
Donor2-CGTCAGGCAGACACTT-1 5770 40072 6
Donor2-CGTCCATGTCATGCAT-1 2189 7949 9
Donor2-CGTCTACCACCAACCG-1 2980 8750 11
Donor2-CGTCTACGTCAATGTC-1 2268 7070 13
Donor2-CGTCTACTCGTTACGA-1 3481 21356 7
Donor2-CGTGAGCCAGTAGAGC-1 3615 28261 7
Donor2-CGTGAGCGTACTCTCC-1 3432 7724 6
Donor2-CGTGTAAAGGCGTACA-1 3697 23834 7
Donor2-CGTGTAAGTATGGTTC-1 2726 7667 11
Donor2-CGTGTCTAGAGGTTAT-1 3812 17778 10
Donor2-CGTGTCTAGGCATTGG-1 2997 13893 10
Donor2-CGTGTCTGTACATGTC-1 2194 6366 11
Donor2-CGTTAGAAGTACGCCC-1 4692 13105 2
Donor2-CGTTCTGCACGAGAGT-1 6228 27361 6
Donor2-CGTTCTGCATGGAATA-1 6755 37946 4
Donor2-CGTTCTGGTTTCGCTC-1 2780 5689 4
Donor2-CGTTCTGTCCACGAAT-1 3269 8256 1
Donor2-CGTTCTGTCTTAACCT-1 6736 29564 5
Donor2-CGTTGGGGTCGCATAT-1 6420 28886 6

Donor2-CGTTGGGTCCGCAAGC-1 1885 6012 13
Donor2-CTAACTTGTTCCCGAG-1 3883 10471 2
Donor2-CTAAGACAGCGTAATA-1 2792 17008 7
Donor2-CTAAGACAGCTTATCG-1 6873 35113 6
Donor2-CTAAGACAGTCCAGGA-1 7509 60796 6
Donor2-CTAAGACTCTTAGAGC-1 1590 6874 8
Donor2-CTAATGGGTACTCTCC-1 1304 5823 8
Donor2-CTACACCAGAAGATTC-1 1688 10277 8
Donor2-CTACATTAGCCTATGT-1 2392 7874 13
Donor2-CTACATTAGTACGCGA-1 4969 26904 10
Donor2-CTACATTGTACGCTGC-1 1991 6027 11
Donor2-CTACATTGTGTGAATA-1 2190 5405 11
Donor2-CTACCCAAGGGTCTCC-1 2784 7700 8
Donor2-CTACCCATCACCAGGC-1 3621 10135 8
Donor2-CTACCCATCGGTTAAC-1 4986 14820 5
Donor2-CTACGTCAGTTAGGTA-1 1809 10354 8
Donor2-CTACGTCTCAAACCGT-1 3307 7566 3
Donor2-CTACGTCTCAACGAAA-1 3417 19419 7
Donor2-CTAGAGTAGGTGATAT-1 2980 9040 11
Donor2-CTAGAGTGTCCATCCT-1 3486 8570 4
Donor2-CTAGAGTTCATTTGGG-1 4003 10301 3
Donor2-CTAGAGTTCCTATGTT-1 5915 25227 6
Donor2-CTAGAGTTCGTACCGG-1 2953 11550 10
Donor2-CTAGCCTAGGCAGGTT-1 4658 14023 1
Donor2-CTAGCCTTCAAGGTAA-1 3632 19466 7
Donor2-CTAGTGAGTAAAGTCA-1 5249 17379 4
Donor2-CTAGTGATCAACGAAA-1 3048 13590 7
Donor2-CTCACACCATCTACGA-1 3862 35446 7
Donor2-CTCACACTCAACGCTA-1 2461 11843 7
Donor2-CTCAGAACAAACTGTC-1 5337 16619 6
Donor2-CTCAGAACATACGCCG-1 3845 10587 3
Donor2-CTCAGAATCTTCCTTC-1 2139 6933 13
Donor2-CTCATTATCTCCTATA-1 3306 11163 11
Donor2-CTCCTAGAGATGGCGT-1 3222 7637 3
Donor2-CTCCTAGCACGGTAGA-1 4415 35408 7
Donor2-CTCGAAATCTTTAGTC-1 2044 7488 9
Donor2-CTCGTACCACAAGACG-1 3175 6383 5
Donor2-CTCGTACGTAAGCACG-1 2423 8908 13
Donor2-CTCGTACTCGCCAAAT-1 1674 10321 8
Donor2-CTCGTACTCTCAACTT-1 3335 7483 6
Donor2-CTCGTCAGTTCTCATT-1 5041 16377 1
Donor2-CTCTAATAGTGGAGTC-1 1926 11069 8
Donor2-CTCTAATGTTGATTCG-1 5202 18910 6
Donor2-CTCTAATTCACGCGGT-1 3545 10259 4
Donor2-CTCTACGGTAAGGGAA-1 2735 8741 11
Donor2-CTCTGGTAGCGATCCC-1 4142 24285 6
Donor2-CTCTGGTAGGCTAGCA-1 4097 15968 6
Donor2-CTCTGGTCAAGCCCAC-1 1810 5622 9
Donor2-CTGAAACAGGCAATTA-1 6820 40146 4
Donor2-CTGAAACCACGCTTTC-1 2020 7631 7
Donor2-CTGAAGTAGAGACGAA-1 1283 6043 8
Donor2-CTGAAGTCAGGACGTA-1 3231 12056 6
Donor2-CTGATAGTCACCACCT-1 2345 9530 13
Donor2-CTGATCCCAAGGTGTG-1 4184 11269 6
Donor2-CTGCCTACACTACAGT-1 2166 8218 13
Donor2-CTGCCTAGTCTAGCGC-1 3443 8526 3

Donor2-CTGCGGATCAGGTTCA-1 1996 6774 13
Donor2-CTGCTGTAGACCACGA-1 1642 7102 8
Donor2-CTGCTGTCACCAACCG-1 4011 10627 3
Donor2-CTGCTGTCATCAGTCA-1 1742 10168 8
Donor2-CTGGTCTAGGATGGAA-1 3706 9462 2
Donor2-CTGGTCTGTCCGTCAG-1 2168 5732 12
Donor2-CTGTGCTAGTACGTTC-1 2706 11374 13
Donor2-CTGTTTATCCAGAGGA-1 1918 6626 13
Donor2-CTTAACTCATGCCTAA-1 2403 9584 9
Donor2-CTTACCGGTGACGGTA-1 3886 24024 7
Donor2-CTTACCGTCACCGGGT-1 9215 58990 6
Donor2-CTTACCGTCTGCGGCA-1 5332 21826 5
Donor2-CTTAGGAAGTGGGATC-1 1442 7441 8
Donor2-CTTGGCTGTAATCACC-1 6933 53938 5
Donor2-CTTTGCGAGGTAGCTG-1 4826 13904 5
Donor2-CTTTGCGAGGTGCACA-1 2892 11875 10
Donor2-CTTTGCGCACCCAGTG-1 2201 7637 13
Donor2-CTTTGCGCAGACGCTC-1 3362 10552 11
Donor2-CTTTGCGCAGTATCTG-1 4908 25869 6
Donor2-CTTTGCGTCGTTGCCT-1 3143 10502 11
Donor2-GAAACTCCAAGACACG-1 7820 52745 6
Donor2-GAAACTCCAATAGCGG-1 2468 9332 7
Donor2-GAAACTCTCTAACTCT-1 3049 7203 8
Donor2-GAAATGAAGCCCAACC-1 1883 5823 13
Donor2-GAAATGACAACACGCC-1 3530 12596 5
Donor2-GAACATCCAGTAAGCG-1 1888 12205 8
Donor2-GAACCTACAAGTACCT-1 7965 82574 4
Donor2-GAACGGACAAACCTAC-1 2062 5718 11
Donor2-GAACGGAGTAAACGCG-1 3244 6997 3
Donor2-GAACGGAGTCTAGCCG-1 1968 9221 7
Donor2-GAAGCAGCACTCAGGC-1 2013 11016 8
Donor2-GAATAAGAGAAGAAGC-1 2625 10030 10
Donor2-GAATGAAGTTGATTGC-1 3335 7787 8
Donor2-GAATGAATCAATCTCT-1 3627 11923 11
Donor2-GACACGCAGTGTTTGC-1 1913 5950 13
Donor2-GACACGCGTAAATACG-1 2593 11388 7
Donor2-GACAGAGCAAGGTTCT-1 1777 6874 8
Donor2-GACAGAGTCTCACATT-1 1971 7493 9
Donor2-GACCAATAGGGTATCG-1 3849 10878 5
Donor2-GACCAATAGTTGAGTA-1 2542 7954 8
Donor2-GACCAATCAACACGCC-1 1537 5422 7
Donor2-GACCAATCAATGGAAT-1 1479 5404 9
Donor2-GACCAATGTATATGAG-1 4711 21575 10
Donor2-GACCTGGCAAGCGTAG-1 6435 34776 6
Donor2-GACCTGGGTAAACCTC-1 2369 15261 8
Donor2-GACCTGGGTACAAGTA-1 1299 5547 8
Donor2-GACCTGGGTACTTCTT-1 3615 14361 9
Donor2-GACGCGTAGACGCTTT-1 1747 6961 7
Donor2-GACGCGTAGATGCCAG-1 2143 8898 7
Donor2-GACGGCTAGCTAGGCA-1 1320 5440 8
Donor2-GACGGCTAGGACAGAA-1 1819 8977 8
Donor2-GACGGCTCAATGCCAT-1 2961 8578 11
Donor2-GACGTGCAGGCATGGT-1 1727 5560 13
Donor2-GACGTGCCAAATTGCC-1 3420 14488 6
Donor2-GACGTTAAGGTAAACT-1 1704 7536 9
Donor2-GACGTTAGTAAGTGGC-1 2319 7655 13

Donor2-GACTAACAGGAGCGTT-1 6868 31599 5
Donor2-GACTACAGTTAGGGTG-1 2594 11529 7
Donor2-GACTACATCGAGAACG-1 2198 6829 11
Donor2-GACTGCGCAGAGTGTG-1 5645 18969 5
Donor2-GACTGCGTCCGCGTTT-1 1466 6027 8
Donor2-GACTGCGTCTCGGACG-1 2213 10027 7
Donor2-GAGCAGAAGGGCACTA-1 1861 6663 7
Donor2-GAGGTGACATGGGACA-1 3301 11735 6
Donor2-GATCAGTAGACAGACC-1 3455 23473 7
Donor2-GATCAGTAGCGTTGCC-1 3753 22456 7
Donor2-GATCAGTCAGCAGTTT-1 1442 8562 8
Donor2-GATCAGTCAGGGTTAG-1 3917 12352 6
Donor2-GATCGATGTCGGCATC-1 5603 21566 6
Donor2-GATCGCGAGCTGAAAT-1 4327 12201 3
Donor2-GATCGCGCATTTCACT-1 1774 5887 10
Donor2-GATGCTAGTTAGATGA-1 2099 8508 13
Donor2-GATGCTATCCGTAGTA-1 1884 11242 8
Donor2-GATTCAGTCATGTCCC-1 1349 5859 8
Donor2-GATTCAGTCGAACTGT-1 4300 10925 5
Donor2-GCAAACTAGGCGCTCT-1 3434 13949 11
Donor2-GCAAACTTCCAGATCA-1 2391 12905 7
Donor2-GCAAACTTCGCTTGTC-1 5929 25045 6
Donor2-GCAAACTTCTGTTTGT-1 9591 54473 5
Donor2-GCAATCACAGGCTGAA-1 4096 11033 4
Donor2-GCACATACAATAACGA-1 2554 10416 10
Donor2-GCACATATCAACTCTT-1 5102 24149 5
Donor2-GCACATATCTAAGCCA-1 2202 6651 11
Donor2-GCACTCTCAGCTGTGC-1 3973 17549 10
Donor2-GCACTCTCAGTCAGCC-1 2167 6935 13
Donor2-GCACTCTCATTCGACA-1 4074 14009 10
Donor2-GCACTCTGTGTGCGTC-1 2134 8750 9
Donor2-GCACTCTTCTGGGCCA-1 1901 6492 9
Donor2-GCAGCCATCTAACTGG-1 1900 7845 7
Donor2-GCAGCCATCTTCTGGC-1 4623 14956 4
Donor2-GCATACAAGGGTTTCT-1 2310 6599 11
Donor2-GCATGATGTAATCACC-1 5211 16610 6
Donor2-GCATGTACACGGTTTA-1 2438 6565 11
Donor2-GCATGTACATCGGACC-1 5402 20569 1
Donor2-GCATGTACATCTGGTA-1 8056 50776 6
Donor2-GCCAAATTCATCGATG-1 4462 12165 1
Donor2-GCCTCTAGTGTCCTCT-1 4148 15175 11
Donor2-GCGACCACACATCCAA-1 2044 12818 8
Donor2-GCGACCACATCCAACA-1 2180 5787 11
Donor2-GCGCAACAGGAGCGTT-1 9573 76949 6
Donor2-GCGCAACCAAGCCGTC-1 6323 32855 6
Donor2-GCGCAACCAATACGCT-1 1934 7592 9
Donor2-GCGCAACGTCTTCGTC-1 2003 10779 8
Donor2-GCGCAACTCAGCAACT-1 2342 7874 13
Donor2-GCGCAGTCACTGTGTA-1 5125 16408 4
Donor2-GCGCCAAAGCAGCGTA-1 3522 16634 6
Donor2-GCGCCAAAGCTGAAAT-1 2022 6860 13
Donor2-GCGCCAAAGGGCTTGA-1 2991 9039 6
Donor2-GCGCGATAGGTGCAAC-1 7524 40702 6
Donor2-GCGCGATCAAGCTGTT-1 2380 13823 7
Donor2-GCGCGATGTTCAGACT-1 5174 25467 5
Donor2-GCGGGTTCACGACGAA-1 7031 33716 6

Donor2-GCGGGTTGTCACTGGC-1 2934 17600 7
Donor2-GCTCCTACACATTCGA-1 5085 17506 2
Donor2-GCTCTGTAGATATGGT-1 2062 12537 8
Donor2-GCTGCAGAGAGTCTGG-1 1743 6469 9
Donor2-GCTGCAGAGATATGCA-1 2935 12411 13
Donor2-GCTGCAGAGCTGCAAG-1 2660 9258 7
Donor2-GCTGCAGAGTGGACGT-1 5413 23718 5
Donor2-GCTGCAGGTTGGTAAA-1 2334 10197 7
Donor2-GCTGCAGTCACCGTAA-1 7051 31759 1
Donor2-GCTGCGACAGTTCATG-1 4072 14693 11
Donor2-GCTGCGAGTAAACACA-1 2485 7221 11
Donor2-GCTGCGATCCGTTGCT-1 5496 18690 6
Donor2-GCTGCTTAGTCGAGTG-1 1925 7847 9
Donor2-GCTGCTTCAAGCCCAC-1 7819 49813 5
Donor2-GCTTCCACAGCGATCC-1 3311 7385 3
Donor2-GCTTGAACAGACAAAT-1 2579 12636 7
Donor2-GCTTGAACATTATCTC-1 2650 13923 7
Donor2-GCTTGAAGTAGGACAC-1 2376 10925 7
Donor2-GCTTGAATCTGGGCCA-1 2714 8135 11
Donor2-GGAAAGCAGCCATCGC-1 7872 69566 6
Donor2-GGAAAGCGTACGCTGC-1 3318 10408 11
Donor2-GGAAAGCTCATCACCC-1 5143 22095 6
Donor2-GGAACTTGTAGCCTAT-1 1601 6634 8
Donor2-GGAACTTTCACTCCTG-1 2404 8514 13
Donor2-GGAATAACATTCGACA-1 4388 12724 4
Donor2-GGAATAATCATTGCCC-1 6280 34723 5
Donor2-GGACAAGAGAGTACCG-1 4145 10720 5
Donor2-GGACAAGCACAGACAG-1 3756 11442 6
Donor2-GGACAAGCATATACGC-1 6580 29232 5
Donor2-GGACAAGTCCGTACAA-1 4259 11740 4
Donor2-GGACAGAAGACAGAGA-1 8207 65382 6
Donor2-GGACAGAAGTACGTAA-1 2381 9093 9
Donor2-GGACATTCATATACGC-1 1715 5530 13
Donor2-GGACATTGTAGAGCTG-1 6264 31216 5
Donor2-GGACATTGTCCGTCAG-1 4276 29545 7
Donor2-GGACATTTCTGGTGTA-1 2698 21926 8
Donor2-GGAGCAACACGGTAGA-1 2844 9470 11
Donor2-GGAGCAATCGCATGGC-1 1717 5651 9
Donor2-GGATGTTAGCGTTTAC-1 2222 6708 11
Donor2-GGATGTTCATGCCTAA-1 7349 44276 6
Donor2-GGATGTTTCAATAAGG-1 2006 12156 8
Donor2-GGATGTTTCCGCAAGC-1 2545 5619 3
Donor2-GGATTACAGCCACGTC-1 1672 5864 13
Donor2-GGATTACAGTGAAGAG-1 4316 11546 4
Donor2-GGATTACTCATTTGGG-1 4721 14100 1
Donor2-GGATTACTCCCGACTT-1 1398 6427 8
Donor2-GGCAATTGTACACCGC-1 3448 11388 13
Donor2-GGCCGATGTAGCGCTC-1 3483 7345 6
Donor2-GGCCGATGTCGAACAG-1 1981 7340 7
Donor2-GGCGACTCAAGCGCTC-1 2787 6678 1
Donor2-GGCGACTCATCGGAAG-1 8974 71391 6
Donor2-GGCGACTGTTCGTGAT-1 2129 8500 13
Donor2-GGCGTGTCATGTAGTC-1 2628 15453 8
Donor2-GGCGTGTGTAGCGTGA-1 1984 13928 8
Donor2-GGCGTGTGTGCTTCTC-1 1976 6470 7
Donor2-GGCTCGAAGTACGTAA-1 3226 14955 13

Donor2-GGCTCGAGTGGGTCAA-1 1788 5968 9
Donor2-GGCTCGATCATCGATG-1 7792 42762 5
Donor2-GGCTGGTAGCGTCTAT-1 4956 17078 6
Donor2-GGGAATGGTGCGAAAC-1 5463 20542 5
Donor2-GGGAATGGTTGGTAAA-1 1693 5385 13
Donor2-GGGAATGGTTGTACAC-1 1651 5388 13
Donor2-GGGAATGTCTTATCTG-1 2598 12786 7
Donor2-GGGACCTCAAACCCAT-1 5578 20393 5
Donor2-GGGACCTTCAACACTG-1 2212 6455 13
Donor2-GGGACCTTCTGTCTAT-1 3033 9116 11
Donor2-GGGAGATAGTTCGATC-1 3797 19620 6
Donor2-GGGAGATGTATCGCAT-1 4836 27312 6
Donor2-GGGAGATGTCTAGCCG-1 3123 16948 7
Donor2-GGGCATCCAATGCCAT-1 5838 22344 6
Donor2-GGGCATCCATCTCGCT-1 2422 10238 6
Donor2-GGGCATCTCCTTTCGG-1 4306 11820 4
Donor2-GGGTCTGTCCTAGGGC-1 4044 10469 4
Donor2-GGGTTGCTCGCCGTGA-1 3655 20091 9
Donor2-GGTATTGGTCTAACGT-1 5533 19231 2
Donor2-GGTGAAGAGGCCATAG-1 3314 20968 7
Donor2-GGTGCGTCACGAGAGT-1 2441 10283 9
Donor2-GTAACGTAGACCGGAT-1 2853 14510 7
Donor2-GTAACTGAGATCCGAG-1 5196 19111 5
Donor2-GTAACTGCACCGCTAG-1 1862 6566 13
Donor2-GTAACTGGTCCTGCTT-1 6272 24982 6
Donor2-GTACGTAAGCCCAATT-1 1674 5414 9
Donor2-GTACGTAAGGCAGTCA-1 2035 7220 10
Donor2-GTACTTTAGTCCATAC-1 9525 108016 6
Donor2-GTAGGCCGTAGCCTCG-1 2373 16794 8
Donor2-GTAGTCACAATGTTGC-1 4316 16513 6
Donor2-GTAGTCAGTTCTGGTA-1 3063 9966 11
Donor2-GTATTCTAGTCTTGCA-1 3413 14840 10
Donor2-GTCAAGTCAGCTTCGG-1 3074 11659 11
Donor2-GTCACGGAGCCGGTAA-1 1782 7715 7
Donor2-GTCATTTTCTAACTTC-1 5251 17326 6
Donor2-GTCCTCAGTAAGCACG-1 2784 15082 7
Donor2-GTCCTCAGTCCGCTGA-1 4324 16866 6
Donor2-GTCCTCATCACCACCT-1 3930 10193 3
Donor2-GTCGGGTCATGGATGG-1 4555 13775 5
Donor2-GTCGGGTGTCTCTTAT-1 1909 6831 9
Donor2-GTCGTAAGTTACGTCA-1 2152 6566 11
Donor2-GTCGTAATCACAGGCC-1 3654 7848 5
Donor2-GTCGTAATCACCACCT-1 2382 5555 8
Donor2-GTCTCGTAGGCTCTTA-1 2561 7843 11
Donor2-GTCTCGTTCGTCGTTC-1 6132 32114 5
Donor2-GTCTCGTTCTTGAGAC-1 1967 8256 8
Donor2-GTCTTCGTCTAACTTC-1 1795 6866 9
Donor2-GTCTTCGTCTTGGGTA-1 1840 8602 7
Donor2-GTGAAGGAGCCAGTAG-1 4663 21165 5
Donor2-GTGAAGGCAGCATACT-1 4993 15232 5
Donor2-GTGAAGGGTAATAGCA-1 3456 8724 3
Donor2-GTGCAGCGTCACCTAA-1 1720 5377 13
Donor2-GTGCATAAGCACCGTC-1 2973 9493 11
Donor2-GTGCATATCACCTTAT-1 5723 20355 5
Donor2-GTGCGGTGTAAACACA-1 3231 9864 6
Donor2-GTGCTTCAGAGGGATA-1 6293 23924 1

Donor2-GTGCTTCTCATGGTCA-1 6013 24013 6
Donor2-GTGGGTCCAGTACACT-1 6326 26841 4
Donor2-GTGGGTCCATGATCCA-1 1990 6292 13
Donor2-GTGGGTCGTATGAATG-1 8837 99628 5
Donor2-GTGGGTCTCACGCATA-1 1808 5932 13
Donor2-GTGTGCGAGATCCTGT-1 1917 7002 13
Donor2-GTGTGCGGTGTCTGAT-1 5512 18986 4
Donor2-GTGTGCGTCGTCCAGG-1 5073 19358 6
Donor2-GTGTTAGAGAGGACGG-1 3133 9578 4
Donor2-GTGTTAGAGCAATCTC-1 2395 7337 11
Donor2-GTGTTAGAGCCACCTG-1 2114 5855 11
Donor2-GTGTTAGTCTGGCGAC-1 1313 6205 8
Donor2-GTTAAGCAGATAGCAT-1 6454 48712 6
Donor2-GTTAAGCAGGCTATCT-1 7550 40025 5
Donor2-GTTAAGCGTTAAAGTG-1 4955 20736 5
Donor2-GTTAAGCTCAGTACGT-1 3218 18571 7
Donor2-GTTAAGCTCATCGGAT-1 2304 10961 7
Donor2-GTTAAGCTCCCGACTT-1 1777 6108 13
Donor2-GTTACAGCACCTCGGA-1 1860 9170 7
Donor2-GTTCATTGTGTGAAAT-1 3691 9521 2
Donor2-GTTCATTTCACTCTTA-1 2536 9253 8
Donor2-GTTCATTTCCCACTTG-1 2827 9021 13
Donor2-GTTCTCGCAACGATCT-1 3485 12121 11
Donor2-GTTCTCGCAATGGAAT-1 3152 8692 1
Donor2-GTTCTCGGTTTACTCT-1 6684 29619 6
Donor2-GTTTCTAGTCTCAACA-1 2284 7154 13
Donor2-GTTTCTATCTCGAGTA-1 2717 5757 5
Donor2-TAAACCGAGACAGACC-1 2326 13192 8
Donor2-TAAACCGAGGCAAAGA-1 5713 39155 6
Donor2-TAAACCGAGTGACATA-1 2832 13568 7
Donor2-TAAACCGCAGTGGAGT-1 4687 15159 8
Donor2-TAAACCGTCGTTTGCC-1 3399 11772 4
Donor2-TAAGAGAAGCATCATC-1 4634 27735 6
Donor2-TAAGAGACATGCTGGC-1 2429 6021 11
Donor2-TAAGCGTGTTGTACAC-1 2842 9806 11
Donor2-TAAGTGCAGGCATGTG-1 3053 7238 1
Donor2-TACACGAAGTGCGTGA-1 2343 10028 7
Donor2-TACAGTGAGGGCTCTC-1 5938 29058 1
Donor2-TACAGTGGTACGACCC-1 2239 6456 13
Donor2-TACAGTGGTTGTGGAG-1 2224 9476 9
Donor2-TACCTATAGAGTACAT-1 5140 16682 6
Donor2-TACCTATAGCCTATGT-1 2146 7433 13
Donor2-TACCTATTCCAAATGC-1 3213 7823 11
Donor2-TACCTATTCTGTGCAA-1 2269 6823 10
Donor2-TACCTTAGTGGTACAG-1 1732 7284 8
Donor2-TACTCATCAAAGTCAA-1 2189 6575 9
Donor2-TACTCGCCAGCTGCAC-1 4949 15052 5
Donor2-TACTTACAGTCGTACT-1 2320 7939 11
Donor2-TACTTACAGTGGGATC-1 5604 22462 6
Donor2-TACTTACCAAACTGTC-1 2110 8163 9
Donor2-TACTTACTCTTCTGGC-1 2050 10464 7
Donor2-TACTTGTCACGGCTAC-1 2148 5599 11
Donor2-TAGCCGGAGCTAACAA-1 1983 8451 13
Donor2-TAGCCGGCAACACGCC-1 7699 43168 6
Donor2-TAGCCGGCAGACGTAG-1 5680 31603 5
Donor2-TAGCCGGTCACAGGCC-1 2457 9376 9

Donor2-TAGGCATGTGGACGAT-1 2543 6900 4
Donor2-TAGGCATGTGTTCGAT-1 5232 16821 5
Donor2-TAGTTGGGTGGGTCAA-1 1750 5851 7
Donor2-TAGTTGGGTTGTCTTT-1 4852 17299 6
Donor2-TATCAGGGTCAACATC-1 4326 24439 6
Donor2-TATCTCACATTACGAC-1 5636 22242 6
Donor2-TATCTCAGTCCGACGT-1 2619 12841 7
Donor2-TATCTCAGTGCAACGA-1 1460 5488 13
Donor2-TATGCCCAGACTACAA-1 5271 15008 1
Donor2-TATGCCCCACCAGTTA-1 5079 22922 6
Donor2-TATGCCCGTTATTCTC-1 4257 24773 6
Donor2-TCAACGATCCAAACAC-1 3836 12432 11
Donor2-TCAATCTGTCACTTCC-1 1994 6643 13
Donor2-TCACAAGCAGTTTACG-1 2910 6025 4
Donor2-TCACGAAAGGTACTCT-1 3011 18220 7
Donor2-TCACGAAGTTGTCGCG-1 2585 10361 13
Donor2-TCACGAATCATCGATG-1 3367 19878 7
Donor2-TCAGATGAGGCATGGT-1 2509 7930 7
Donor2-TCAGATGAGGTTACCT-1 2403 6296 5
Donor2-TCAGCAAAGGGATGGG-1 3518 13057 4
Donor2-TCAGCAACATTACGAC-1 1886 6941 9
Donor2-TCAGCAAGTATGCTTG-1 4018 11178 4
Donor2-TCAGCAATCTCGGACG-1 3337 7298 3
Donor2-TCAGCTCCATGACATC-1 6251 44929 6
Donor2-TCAGGATAGACTTGAA-1 1397 5396 8
Donor2-TCAGGATCAGTTCCCT-1 6388 34245 6
Donor2-TCAGGATCATCGATTG-1 4076 10340 5
Donor2-TCAGGTAGTTACCAGT-1 1835 5591 13
Donor2-TCAGGTAGTTACGGAG-1 2617 6005 1
Donor2-TCAGGTATCTACCAGA-1 4505 14252 2
Donor2-TCATTACAGATCGGGT-1 1501 7093 8
Donor2-TCATTACCACGGTAAG-1 2099 6671 12
Donor2-TCATTTGAGCACCGTC-1 1870 5494 13
Donor2-TCATTTGAGGTCATCT-1 2441 9738 7
Donor2-TCATTTGCACGGTAAG-1 2386 7150 9
Donor2-TCATTTGCAGAAGCAC-1 3477 12441 11
Donor2-TCATTTGTCGCAAGCC-1 1839 10780 8
Donor2-TCCACACCAAAGAATC-1 2553 6876 5
Donor2-TCCACACGTCGAATCT-1 3319 8718 1
Donor2-TCCCGATAGCCAACAG-1 2067 6265 10
Donor2-TCCCGATGTAACGTTC-1 4108 15128 6
Donor2-TCGAGGCAGCCCGAAA-1 1796 10716 8
Donor2-TCGAGGCTCAACTCTT-1 2091 6454 13
Donor2-TCGCGAGGTCCGACGT-1 3138 7228 1
Donor2-TCGCGTTAGCTGTTCA-1 2194 13521 7
Donor2-TCGCGTTTCCGCGTTT-1 2341 15491 8
Donor2-TCGCGTTTCTGCTTGC-1 8876 58979 6
Donor2-TCGGGACAGTTCGCAT-1 1572 5913 8
Donor2-TCGGGACGTCCGTGAC-1 1925 11196 8
Donor2-TCGGGACGTGTTCTTT-1 2946 14027 7
Donor2-TCGGGACTCGCAAACT-1 6000 23683 5
Donor2-TCGTACCCAGGTGCCT-1 2584 8292 11
Donor2-TCGTACCGTAGGACAC-1 4896 18795 2
Donor2-TCGTACCGTTTCGCTC-1 2549 8188 11
Donor2-TCGTAGACATGCCTTC-1 2037 8168 9
Donor2-TCGTAGAGTCTAACGT-1 5532 19307 5

Donor2-TCGTAGAGTTTGTTGG-1 1841 7710 7
Donor2-TCTATTGGTCATGCAT-1 2692 6702 6
Donor2-TCTATTGTCGCGGATC-1 2202 10236 8
Donor2-TCTATTGTCTAACGGT-1 3094 12905 10
Donor2-TCTCATATCAGCACAT-1 6549 54674 10
Donor2-TCTCATATCTGGTGTA-1 4902 16380 5
Donor2-TCTCTAAAGCCGATTT-1 2674 5795 6
Donor2-TCTCTAAAGCTAGCCC-1 1741 9934 8
Donor2-TCTCTAAAGTGAACAT-1 1622 7604 8
Donor2-TCTCTAAGTTACGACT-1 5210 48791 7
Donor2-TCTGAGACACGCCAGT-1 2308 11097 9
Donor2-TCTGAGACATGACATC-1 1584 5593 8
Donor2-TCTGGAACATAAAGGT-1 2100 11300 8
Donor2-TCTGGAACATACTACG-1 3941 10837 3
Donor2-TCTTCGGAGACTAGAT-1 3586 9301 4
Donor2-TCTTCGGAGCAGCCTC-1 4286 15556 6
Donor2-TCTTCGGCATTCTTAC-1 2657 21362 8
Donor2-TCTTCGGTCGTCCAGG-1 4967 28089 7
Donor2-TCTTTCCAGATATGCA-1 1707 5419 13
Donor2-TCTTTCCTCTCGCTTG-1 2251 12201 8
Donor2-TGAAAGAAGCAGGTCA-1 2010 6362 13
Donor2-TGAAAGAAGGACAGAA-1 3909 9233 6
Donor2-TGACAACCAGTTCATG-1 2116 12399 7
Donor2-TGACGGCCAGCGTTCG-1 4248 11523 2
Donor2-TGACGGCTCAGTTGAC-1 2563 7024 11
Donor2-TGACGGCTCCACGTGG-1 2582 10813 13
Donor2-TGACTAGAGCCAACAG-1 1684 5517 13
Donor2-TGACTAGAGGCTAGGT-1 1619 5696 8
Donor2-TGACTAGCATTCCTCG-1 2099 7469 9
Donor2-TGACTAGGTAAGCACG-1 3025 9390 8
Donor2-TGACTAGGTACCGGCT-1 6912 28697 1
Donor2-TGACTAGTCTTTACAC-1 3142 6828 3
Donor2-TGACTTTAGGTTCCTA-1 3465 7904 3
Donor2-TGACTTTGTGATGATA-1 3562 23914 7
Donor2-TGAGAGGAGGAGCGAG-1 6613 28537 1
Donor2-TGAGAGGGTAAATACG-1 6350 32572 6
Donor2-TGAGAGGTCTTACCGC-1 4075 12304 4
Donor2-TGAGAGGTCTTCTGGC-1 5877 21912 5
Donor2-TGAGCATTCGCCAGCA-1 3485 8622 1
Donor2-TGAGGGAAGTGTTAGA-1 6560 36419 6
Donor2-TGAGGGACAGGTCTCG-1 2588 10504 9
Donor2-TGAGGGACATCCCACT-1 2391 6669 13
Donor2-TGAGGGATCCTATGTT-1 2121 6371 11
Donor2-TGATTTCCAATGAATG-1 7151 35459 6
Donor2-TGCACCTAGTTTCCTT-1 2727 9565 13
Donor2-TGCACCTCAAAGTCAA-1 2359 6959 8
Donor2-TGCACCTCACAGTCGC-1 1704 6942 7
Donor2-TGCACCTGTACAGTTC-1 4783 16971 5
Donor2-TGCACCTTCACTTACT-1 6435 27284 1
Donor2-TGCCAAACAGACAAAT-1 6607 28996 5
Donor2-TGCCAAACATCGATGT-1 1520 5574 8
Donor2-TGCCAAAGTCTTCGTC-1 1761 5764 13
Donor2-TGCCAAATCCGTTGTC-1 6050 47246 6
Donor2-TGCCCATCACAGACTT-1 1289 5921 8
Donor2-TGCCCATGTCCCTACT-1 1199 5633 8
Donor2-TGCCCATTCCGAGCCA-1 2801 5423 4

Donor2-TGCCCTAAGGATTCGG-1 4740 12817 5
Donor2-TGCCCTAAGTACACCT-1 2114 5427 9
Donor2-TGCGCAGCATCGATGT-1 2588 8627 11
Donor2-TGCGCAGGTCCTGCTT-1 6566 32145 6
Donor2-TGCGGGTCACATGGGA-1 4404 13638 2
Donor2-TGCGGGTGTCTCCCTA-1 2519 8557 7
Donor2-TGCGGGTTCCTTTCTC-1 5793 20295 5
Donor2-TGCGTGGGTCTCCCTA-1 6215 21476 5
Donor2-TGCGTGGGTGGTCTCG-1 1459 5445 7
Donor2-TGCGTGGGTTGAGTTC-1 1993 7481 13
Donor2-TGCGTGGTCTACTATC-1 1936 10742 8
Donor2-TGCTACCAGACATAAC-1 6183 22718 4
Donor2-TGCTACCAGCCGGTAA-1 7566 66795 5
Donor2-TGCTACCCAAACCTAC-1 5197 17128 4
Donor2-TGCTACCCAAACGTGG-1 3190 7824 2
Donor2-TGCTGCTTCGTTACAG-1 2537 8057 11
Donor2-TGCTGCTTCTAACTCT-1 4802 13262 1
Donor2-TGGCCAGAGGACTGGT-1 3686 13268 6
Donor2-TGGCCAGAGTCGCCGT-1 2561 9874 6
Donor2-TGGCGCAAGTCTCCTC-1 1839 6076 9
Donor2-TGGCGCACAAGGACTG-1 1669 8270 8
Donor2-TGGCGCATCACTCTTA-1 1983 7160 9
Donor2-TGGCTGGAGCGTAGTG-1 3638 8415 6
Donor2-TGGCTGGTCGGAAACG-1 4961 28357 6
Donor2-TGGGAAGAGGGATCTG-1 7564 40842 6
Donor2-TGGGAAGGTACATCCA-1 2355 8984 8
Donor2-TGGGAAGGTCCAGTGC-1 2526 7643 13
Donor2-TGGGAAGTCCGTCAAA-1 1763 8135 7
Donor2-TGGGCGTAGTACCGGA-1 1945 7570 10
Donor2-TGGGCGTGTCTCTCGT-1 2099 8907 9
Donor2-TGGGCGTGTTCGCTAA-1 2247 8924 6
Donor2-TGGTTAGGTACGCTGC-1 2697 9832 13
Donor2-TGGTTAGTCAGGTTCA-1 8746 64464 6
Donor2-TGGTTCCGTCATGCCG-1 2818 9455 13
Donor2-TGTATTCGTAAGGATT-1 2660 8888 11
Donor2-TGTCCCATCACGCATA-1 2233 9216 9
Donor2-TGTGGTATCGCAAGCC-1 2193 8674 7
Donor2-TTAACTCAGCTAGTGG-1 1692 5521 13
Donor2-TTAACTCTCATATCGG-1 3173 6938 3
Donor2-TTAGGACAGTCTTGCA-1 3908 9735 2
Donor2-TTAGGACTCTTCGGTC-1 1570 5726 13
Donor2-TTAGTTCAGAGCCTAG-1 1970 8078 8
Donor2-TTAGTTCAGGGAACGG-1 2310 7377 6
Donor2-TTAGTTCCACGGCTAC-1 2205 11410 7
Donor2-TTAGTTCCAGCCTTGG-1 5904 22095 5
Donor2-TTAGTTCTCATAGCAC-1 5120 16018 4
Donor2-TTATGCTGTAGGCATG-1 3040 15834 7
Donor2-TTCCCAGAGAGTCTGG-1 1605 8488 8
Donor2-TTCCCAGAGCAATCTC-1 2239 12322 7
Donor2-TTCCCAGCAATCTGCA-1 1702 6623 7
Donor2-TTCGAAGAGAAAGTGG-1 3103 8556 2
Donor2-TTCGGTCAGCTAACTC-1 1882 6615 9
Donor2-TTCGGTCAGGACATTA-1 2940 18125 7
Donor2-TTCGGTCGTCCAGTTA-1 1776 6560 13
Donor2-TTCGGTCGTTGTCGCG-1 1871 14322 8
Donor2-TTCTACAAGCCGCCTA-1 2643 7504 11

Donor2-TTCTACAGTCCAAGTT-1 2558 6677 11
Donor2-TTCTCAAAGTCCTCCT-1 2288 6857 11
Donor2-TTCTCAACAGACACTT-1 3600 8216 5
Donor2-TTCTCCTCATATACCG-1 5922 18784 3
Donor2-TTCTTAGGTACGCACC-1 7996 43520 5
Donor2-TTCTTAGGTTCCGGCA-1 1447 5957 8
Donor2-TTGAACGCACCAACCG-1 3924 20522 7
Donor2-TTGAACGCATACGCCG-1 3010 11903 6
Donor2-TTGAACGCATCAGTAC-1 2114 7463 13
Donor2-TTGAACGGTATGAATG-1 1892 7669 9
Donor2-TTGACTTCAAGTAATG-1 4852 25640 11
Donor2-TTGACTTTCACCATAG-1 6255 32788 6
Donor2-TTGACTTTCCCAACGG-1 4401 11525 2
Donor2-TTGCCGTCACCGAATT-1 2123 5877 11
Donor2-TTGCCGTCAGCTCCGA-1 6138 22121 5
Donor2-TTGCCGTTCCGGCACA-1 4108 10688 2
Donor2-TTGGAACGTTACGACT-1 1535 5588 8
Donor2-TTGTAGGTCAGTTTGG-1 3561 17724 7
Donor2-TTGTAGGTCCTTAATC-1 2272 16771 8
Donor2-TTTACTGGTCCCTACT-1 2433 7314 11
Donor2-TTTACTGTCGGACAAG-1 2269 6721 11
Donor2-TTTATGCGTCCGTCAG-1 3287 8269 8
Donor2-TTTATGCTCTGTGCAA-1 1421 5904 8
Donor2-TTTCCTCCAAGGTGTG-1 2434 10210 9
Donor2-TTTCCTCCACCCATTC-1 1669 5913 7
Donor2-TTTGCGCAGGTTACCT-1 4874 18279 5
Donor2-TTTGCGCAGTGGTAAT-1 1333 5649 8
Donor2-TTTGGTTCATGAAGTA-1 1707 9314 8
Donor2-TTTGTCAAGTCTTGCA-1 1905 7673 7
Donor2-TTTGTCACAAACGTGG-1 5333 21715 1
Donor2-TTTGTCACAGCCAGAA-1 2236 6542 11
Donor2-TTTGTCAGTTCGTCTC-1 5430 19233 1
Donor2-TTTGTCATCACCAGGC-1 3081 6924 4
Donor2-TTTGTCATCGTAGATC-1 1980 6620 13
Donor2-TTTGTCATCTTCAACT-1 2180 7194 13
Donor2-AAACGGGAGGTGATTA-2 1760 9586 8
Donor2-AAACGGGCACTCTGTC-2 4966 17736 6
Donor2-AAACGGGGTCATATGC-2 2043 6099 10
Donor2-AAACGGGGTTTACTCT-2 9465 85342 6
Donor2-AAAGATGCATGTCTCC-2 5822 22092 1
Donor2-AAAGATGTCAGCAACT-2 1709 6620 7
Donor2-AAAGATGTCGTTACGA-2 6729 30329 5
Donor2-AAAGCAAGTAAAGGAG-2 4165 22120 6
Donor2-AAAGCAAGTACCATCA-2 3657 9384 1
Donor2-AAAGCAATCACTTCAT-2 5050 15801 1
Donor2-AAAGTAGAGCTCCTCT-2 3652 13936 11
Donor2-AAAGTAGGTTCGTGAT-2 6115 23564 6
Donor2-AAAGTAGTCATGCTCC-2 2051 8276 9
Donor2-AAATGCCTCATGTCTT-2 4725 14366 3
Donor2-AACACGTCAGACTCGC-2 5481 18630 6
Donor2-AACCATGAGACTAGAT-2 3217 7584 3
Donor2-AACCATGAGCGATATA-2 3026 6862 4
Donor2-AACCATGAGGAATTAC-2 1878 6368 9
Donor2-AACCATGCAAATTGCC-2 1596 8319 8
Donor2-AACCGCGAGCTGTTCA-2 4655 27867 6
Donor2-AACCGCGGTTCACCTC-2 7017 54674 5

Donor2-AACCGCGTCGGTCCGA-2 4173 11290 6
Donor2-AACGTTGAGCGTCTAT-2 3484 18960 7
Donor2-AACTCAGAGGGCACTA-2 4056 23938 7
Donor2-AACTCAGAGGTAGCCA-2 4298 11466 3
Donor2-AACTCAGGTACAGACG-2 3691 21468 7
Donor2-AACTCAGTCCGAACGC-2 6651 51399 6
Donor2-AACTCCCAGGAGCGTT-2 6460 35105 4
Donor2-AACTCCCAGTAGCCGA-2 2182 6088 11
Donor2-AACTCCCGTTGTCGCG-2 3148 19220 7
Donor2-AACTCCCTCTTGCATT-2 7645 57323 6
Donor2-AACTCTTTCTGTTTGT-2 5359 34189 6
Donor2-AACTGGTCATACCATG-2 1991 8659 7
Donor2-AACTGGTGTAGCTTGT-2 1989 5744 13
Donor2-AACTGGTGTCTCTTAT-2 2282 14939 7
Donor2-AACTTTCAGTGGGCTA-2 1806 6300 9
Donor2-AACTTTCCAGACAAAT-2 6232 39604 6
Donor2-AAGACCTAGACCGGAT-2 2071 12318 7
Donor2-AAGACCTAGTGAATTG-2 6114 28365 6
Donor2-AAGACCTGTAAATACG-2 4931 28243 11
Donor2-AAGGAGCGTGCAGGTA-2 4369 13621 5
Donor2-AAGGCAGGTGTTTGTG-2 5876 20726 1
Donor2-AAGGTTCCACTGCCAG-2 1688 6667 13
Donor2-AAGTCTGAGCTGAACG-2 3794 10077 1
Donor2-AAGTCTGAGTCATGCT-2 5760 22686 6
Donor2-AAGTCTGTCCGCAAGC-2 3218 13084 11
Donor2-AATCCAGGTCGCGTGT-2 2777 5994 3
Donor2-AATCCAGTCCGCTGTT-2 5543 19413 1
Donor2-AATCCAGTCTGCGTAA-2 1571 8246 8
Donor2-AATCGGTAGTACGTAA-2 3611 7681 5
Donor2-AATCGGTTCTTCGAGA-2 5760 28366 6
Donor2-ACACCAAGTTTAAGCC-2 2272 9890 9
Donor2-ACACCCTAGATGCGAC-2 1544 8143 8
Donor2-ACACCCTCATGTAAGA-2 2334 6837 13
Donor2-ACACCCTGTAATTGGA-2 5277 18651 5
Donor2-ACACCGGCACATGACT-2 4186 27003 6
Donor2-ACACCGGGTCAAGCGA-2 2144 6970 11
Donor2-ACACCGGGTCCATCCT-2 3481 10920 12
Donor2-ACACCGGGTGCAACGA-2 2084 7114 13
Donor2-ACACCGGTCGAGAACG-2 1485 7931 8
Donor2-ACACCGGTCGCATGAT-2 4036 9387 3
Donor2-ACACTGAAGAGTAAGG-2 1295 5780 8
Donor2-ACACTGAAGCTCTCGG-2 3945 14207 11
Donor2-ACACTGATCAGTACGT-2 2086 7641 9
Donor2-ACAGCCGAGTGGCACA-2 7372 42439 6
Donor2-ACAGCCGGTCGCATCG-2 1272 6283 8
Donor2-ACAGCTAAGCTAGGCA-2 3714 22609 6
Donor2-ACAGCTATCCCTCAGT-2 3200 17299 7
Donor2-ACAGCTATCCTGCCAT-2 3977 9514 6
Donor2-ACATACGAGACTACAA-2 1807 8002 7
Donor2-ACATACGAGAGTGACC-2 4112 16021 6
Donor2-ACATACGAGCCACCTG-2 2456 16660 8
Donor2-ACATACGAGGCCCGTT-2 5427 17352 2
Donor2-ACATACGCAGCTCGCA-2 1765 8464 7
Donor2-ACATCAGCAGTGGGAT-2 6065 28785 4
Donor2-ACATCAGTCGTACCGG-2 3328 12174 11
Donor2-ACATCAGTCTGGTGTA-2 3012 16386 7

Donor2-ACATGGTGTCCGAAGA-2 1819 6813 9
Donor2-ACCAGTAAGACTGTAA-2 3313 10864 11
Donor2-ACCAGTATCGGTTCGG-2 3863 12582 11
Donor2-ACCCACTTCAGTTGAC-2 2063 6310 13
Donor2-ACCGTAAAGTGTACTC-2 1472 7752 8
Donor2-ACCGTAATCTCCCTGA-2 2920 18341 7
Donor2-ACCTTTAGTGTTCGAT-2 1776 5999 9
Donor2-ACGAGGACATGTCGAT-2 2374 18981 8
Donor2-ACGAGGAGTCAAAGAT-2 5787 25826 6
Donor2-ACGAGGAGTGATGCCC-2 2137 8779 7
Donor2-ACGAGGATCACGGTTA-2 8498 54651 5
Donor2-ACGAGGATCCTGTACC-2 1961 6028 11
Donor2-ACGATACGTAGGCTGA-2 1746 5710 13
Donor2-ACGATGTAGACCACGA-2 1666 5970 13
Donor2-ACGATGTCAGTTCATG-2 4205 16191 11
Donor2-ACGATGTGTAGCTAAA-2 1810 10588 8
Donor2-ACGATGTTCATGCTCC-2 3567 17030 6
Donor2-ACGCCAGAGAGCAATT-2 4574 20714 6
Donor2-ACGCCAGAGATCGGGT-2 1783 6405 9
Donor2-ACGCCAGCAGTCGTGC-2 2890 13813 7
Donor2-ACGCCAGCATTACGAC-2 3278 7957 3
Donor2-ACGCCAGTCAACCATG-2 3402 31820 8
Donor2-ACGCCGAAGGTGCAAC-2 2237 6109 10
Donor2-ACGGAGACATGCCACG-2 6393 26358 5
Donor2-ACGGAGACATGCTAGT-2 3859 26770 7
Donor2-ACGGAGATCAACACCA-2 5483 18303 6
Donor2-ACGGCCAAGCCACGCT-2 4412 12374 5
Donor2-ACGGGTCAGAGTAATC-2 5810 22345 2
Donor2-ACGTCAACATGGTCTA-2 3174 12841 8
Donor2-ACGTCAAGTTAGGGTG-2 4333 13181 1
Donor2-ACGTCAATCAACACCA-2 1359 6238 8
Donor2-ACGTCAATCCATGAGT-2 3414 18015 7
Donor2-ACTATCTGTAACGCGA-2 4998 15349 5
Donor2-ACTATCTGTTTCCACC-2 5158 16994 6
Donor2-ACTGAACCAAGCCGTC-2 1824 8431 7
Donor2-ACTGAACCACAGATTC-2 6653 28479 1
Donor2-ACTGAGTGTAGGCTGA-2 3467 9026 6
Donor2-ACTGAGTGTGCAACGA-2 3307 7496 5
Donor2-ACTGAGTTCAGAGCTT-2 5639 17674 5
Donor2-ACTGATGAGCACACAG-2 1656 7930 8
Donor2-ACTGATGAGCCAACAG-2 6800 30063 5
Donor2-ACTGCTCTCCATGAGT-2 2702 14613 7
Donor2-ACTTACTAGGAATTAC-2 5705 19457 1
Donor2-ACTTACTCAGGACCCT-2 3146 7480 3
Donor2-ACTTACTTCAGTTAGC-2 3739 16641 6
Donor2-ACTTACTTCTTACCGC-2 2998 9485 11
Donor2-ACTTGTTCAAGCGCTC-2 5164 18218 2
Donor2-ACTTGTTCATTAGGCT-2 7141 34599 1
Donor2-ACTTTCACAGACGCTC-2 3266 21173 7
Donor2-ACTTTCATCCTGCCAT-2 7048 44599 4
Donor2-ACTTTCATCTGCTGTC-2 2057 6101 13
Donor2-ACTTTCATCTTGACGA-2 2359 10059 9
Donor2-AGACGTTTCGTAGATC-2 7143 46189 6
Donor2-AGAGCGACATTGGCGC-2 3449 24856 7
Donor2-AGAGCTTAGCGATTCT-2 6456 50459 6
Donor2-AGAGCTTAGTCGTACT-2 4261 22222 10

Donor2-AGAGCTTTCAGATAAG-2 3606 23437 7
Donor2-AGAGTGGAGAGGTAGA-2 1715 9972 8
Donor2-AGAGTGGAGCGTCTAT-2 6700 29600 5
Donor2-AGAGTGGAGCTGTTCA-2 6559 29293 5
Donor2-AGAGTGGAGTCTCCTC-2 2690 5893 3
Donor2-AGAGTGGTCACTCCTG-2 3328 11576 11
Donor2-AGATCTGAGTTCGCGC-2 2207 5691 11
Donor2-AGATTGCCACCCAGTG-2 2486 9336 10
Donor2-AGATTGCCACTAGTAC-2 1735 9276 7
Donor2-AGATTGCCATTAGCCA-2 1995 6857 13
Donor2-AGATTGCGTACCCAAT-2 3976 21335 6
Donor2-AGATTGCTCACCACCT-2 6427 27400 5
Donor2-AGATTGCTCGGAATCT-2 2072 7562 9
Donor2-AGATTGCTCTGCGACG-2 2307 8990 9
Donor2-AGCAGCCCACCTGGTG-2 2062 7433 13
Donor2-AGCAGCCCATCACGTA-2 5258 16308 6
Donor2-AGCAGCCTCTAACTCT-2 2764 6046 2
Donor2-AGCATACAGAGGGCTT-2 2784 14589 7
Donor2-AGCATACGTATCACCA-2 6081 27918 6
Donor2-AGCGTATCATATGCTG-2 1327 5946 8
Donor2-AGCGTATTCAGTGCAT-2 3611 8611 3
Donor2-AGCGTATTCGCTTGTC-2 4774 27473 6
Donor2-AGCGTATTCTAACCGA-2 6927 38153 5
Donor2-AGCGTATTCTCAACTT-2 3150 6430 5
Donor2-AGCGTCGCAAGCTGTT-2 3691 21130 7
Donor2-AGCGTCGCAATGGATA-2 5478 20560 1
Donor2-AGCGTCGCAGCTGTTA-2 1692 5721 13
Donor2-AGCTCCTAGGATTCGG-2 2751 13442 7
Donor2-AGCTCCTCATGGTCAT-2 5834 49131 6
Donor2-AGCTCCTGTCACAAGG-2 5358 15194 1
Donor2-AGCTCCTGTCTGATCA-2 2024 5826 6
Donor2-AGCTCTCGTGTGTGCC-2 5824 25110 6
Donor2-AGCTCTCTCATCTGTT-2 4191 18524 6
Donor2-AGGCCACAGTCAAGCG-2 3204 6979 2
Donor2-AGGCCACCACGTGAGA-2 1746 6382 13
Donor2-AGGCCACCATCGGTTA-2 3966 10830 6
Donor2-AGGCCGTGTGTTGGGA-2 4006 10919 4
Donor2-AGGCCGTTCGCTTAGA-2 5781 36939 10
Donor2-AGGGAGTGTTCCTCCA-2 6380 27143 5
Donor2-AGGGATGAGATAGGAG-2 1916 12479 8
Donor2-AGGGATGCAGTATCTG-2 3253 7365 3
Donor2-AGGGATGTCGATGAGG-2 4665 15517 6
Donor2-AGGGTGAAGGGCTTGA-2 3946 9775 6
Donor2-AGGGTGAAGTGAAGAG-2 3823 9368 3
Donor2-AGGGTGACACTCAGGC-2 2250 6411 10
Donor2-AGGGTGATCCTCAACC-2 2575 11010 10
Donor2-AGGTCATCATTCACTT-2 4851 14458 6
Donor2-AGGTCCGAGAGCTGGT-2 6394 31255 1
Donor2-AGGTCCGCATTCTCAT-2 2464 11734 7
Donor2-AGGTCCGGTTACTGAC-2 1390 5766 8
Donor2-AGGTCCGTCAACACGT-2 1951 8188 10
Donor2-AGGTCCGTCCGAGCCA-2 3107 16762 7
Donor2-AGTGAGGAGAGGTAGA-2 2285 9739 12
Donor2-AGTGGGAAGACACGAC-2 2169 7845 13
Donor2-AGTGGGACAAACCTAC-2 3382 20533 7
Donor2-AGTGGGACATCCAACA-2 1802 8721 8

Donor2-AGTGTCAAGATAGCAT-2 5455 21190 6
Donor2-AGTGTCAAGTAAGTAC-2 2237 8773 7
Donor2-AGTGTCATCCAAATGC-2 7173 50494 6
Donor2-AGTGTCATCGTTGCCT-2 1786 6329 13
Donor2-AGTTGGTAGCACCGTC-2 5258 18805 2
Donor2-AGTTGGTGTGTAACGG-2 1766 7297 7
Donor2-ATAACGCAGTTGTAGA-2 3331 16950 6
Donor2-ATAAGAGTCCTTAATC-2 2972 8967 5
Donor2-ATAGACCAGAAGATTC-2 1667 7040 13
Donor2-ATCACGACAGGTGGAT-2 2279 9034 7
Donor2-ATCACGAGTATCAGTC-2 2471 7103 11
Donor2-ATCACGATCATCATTC-2 2294 13724 8
Donor2-ATCACGATCCAAGTAC-2 5418 34896 10
Donor2-ATCATCTCAAGGACAC-2 2386 14156 8
Donor2-ATCATCTGTCGTCTTC-2 5495 21115 6
Donor2-ATCATGGAGCACCGCT-2 4004 10021 5
Donor2-ATCATGGCATACGCCG-2 3720 17748 6
Donor2-ATCATGGGTCACTGGC-2 2457 7191 13
Donor2-ATCATGGTCTTGCAAG-2 2334 7123 11
Donor2-ATCCGAAGTGGTACAG-2 2611 9354 10
Donor2-ATCGAGTTCGGAAACG-2 1815 11029 8
Donor2-ATCTACTAGATCCGAG-2 3183 7199 3
Donor2-ATCTGCCAGATAGCAT-2 3908 9573 5
Donor2-ATGAGGGAGGGCTTGA-2 3257 18980 7
Donor2-ATGCGATAGCACCGCT-2 2842 13812 7
Donor2-ATGCGATAGGCCCTTG-2 6842 31484 5
Donor2-ATGCGATGTCACCCAG-2 6267 28221 5
Donor2-ATGGGAGTCACTCTTA-2 2338 7251 11
Donor2-ATGTGTGCATCCGTGG-2 1711 8830 7
Donor2-ATGTGTGGTCCAGTGC-2 1889 6863 9
Donor2-ATGTGTGGTGTATGGG-2 1997 7702 9
Donor2-ATGTGTGTCGATGAGG-2 9801 94670 6
Donor2-ATTACTCAGTACGACG-2 7042 35628 5
Donor2-ATTACTCCACGCCAGT-2 1368 5687 8
Donor2-ATTACTCCAGTAAGAT-2 1584 8968 8
Donor2-ATTACTCGTACGAAAT-2 5907 27144 4
Donor2-ATTATCCGTACCCAAT-2 5755 17334 4
Donor2-ATTATCCGTCAGATAA-2 4503 16258 6
Donor2-ATTATCCGTTCCGTCT-2 3252 12036 13
Donor2-ATTCTACAGATAGGAG-2 1313 7256 8
Donor2-ATTCTACCACTTAACG-2 3276 17523 6
Donor2-ATTGGACAGTGTCCCG-2 1973 6532 9
Donor2-ATTTCTGAGGTGTGGT-2 2031 10133 7
Donor2-ATTTCTGAGTCTTGCA-2 1899 6835 8
Donor2-ATTTCTGCACATGACT-2 4827 14545 4
Donor2-ATTTCTGTCTAACTGG-2 5322 17771 5
Donor2-CAACCAAAGTAGCGGT-2 1587 5873 13
Donor2-CAACCAAGTATAGTAG-2 2005 6389 13
Donor2-CAACCAAGTCGAAAGC-2 3224 16784 7
Donor2-CAACCTCCAGGTGGAT-2 2254 6903 6
Donor2-CAACTAGAGATCCCAT-2 2332 7231 6
Donor2-CAACTAGGTATAGGGC-2 3965 13766 11
Donor2-CAAGAAAGTCCGTTAA-2 8183 54694 5
Donor2-CAAGATCTCAGTTCGA-2 6916 30335 1
Donor2-CAAGTTGCAAGTTCTG-2 3791 31818 7
Donor2-CAAGTTGCAGGACGTA-2 1848 7343 8

Donor2-CAAGTTGGTCCGCTGA-2 3788 10646 2
Donor2-CAAGTTGTCCACGTGG-2 2491 8097 13
Donor2-CACAAACAGTGGAGTC-2 2889 11411 7
Donor2-CACAAACGTAGTAGTA-2 3470 7959 1
Donor2-CACACAAAGGATTCGG-2 2455 8079 8
Donor2-CACACAAAGTCGTACT-2 4342 12782 1
Donor2-CACACAAGTCCTAGCG-2 1821 7705 7
Donor2-CACACCTGTTAAGATG-2 6489 46629 10
Donor2-CACACTCGTACAAGTA-2 3186 14688 6
Donor2-CACAGGCAGCTCTCGG-2 4035 11063 3
Donor2-CACAGGCCATGGTCAT-2 3647 12608 11
Donor2-CACCAGGAGTGTGAAT-2 7423 37953 5
Donor2-CACCTTGCACATCCAA-2 2628 16210 7
Donor2-CACCTTGGTGATGATA-2 4891 31171 10
Donor2-CACTCCATCAACGGGA-2 4223 16648 6
Donor2-CACTCCATCAGAGCTT-2 4017 21313 10
Donor2-CACTCCATCGAATGCT-2 2948 9987 11
Donor2-CAGAATCGTGCTCTTC-2 7594 49183 6
Donor2-CAGAATCTCGGCCGAT-2 1416 5768 13
Donor2-CAGAGAGAGCTGCGAA-2 2969 15594 7
Donor2-CAGAGAGCACATGTGT-2 6191 33555 5
Donor2-CAGATCAAGCCAGGAT-2 6951 41285 4
Donor2-CAGATCAGTGTGGTTT-2 1501 7014 8
Donor2-CAGATCAGTTACCGAT-2 1863 5975 13
Donor2-CAGATCATCTACGAGT-2 3633 18327 10
Donor2-CAGCAGCCACAGAGGT-2 3185 7565 5
Donor2-CAGCAGCTCACCAGGC-2 9177 150014 5
Donor2-CAGCATAAGTCATGCT-2 5067 27020 6
Donor2-CAGCATACAGGCAGTA-2 2449 7125 13
Donor2-CAGCATATCCGTTGTC-2 2044 5755 13
Donor2-CAGCCGAAGGACAGAA-2 6019 25223 6
Donor2-CAGCCGATCGCGGATC-2 2407 8969 13
Donor2-CAGCGACAGCACCGTC-2 2784 8457 8
Donor2-CAGCGACCACTAAGTC-2 6561 27795 1
Donor2-CAGCGACGTCGCGTGT-2 5488 20103 6
Donor2-CAGCGACTCATAGCAC-2 3269 17117 7
Donor2-CAGCGACTCATTGCGA-2 2715 11360 9
Donor2-CAGCTAACAGTCGATT-2 1953 12282 8
Donor2-CAGCTAACATTTCAGG-2 1477 5927 13
Donor2-CAGCTGGCATATACCG-2 5241 17925 1
Donor2-CAGCTGGGTTATCCGA-2 5052 29440 10
Donor2-CAGCTGGTCCCAGGTG-2 1553 7756 8
Donor2-CAGGTGCAGCTGCCCA-2 6056 25952 6
Donor2-CAGGTGCAGGGCACTA-2 2106 6417 11
Donor2-CAGGTGCCACGCTTTC-2 2369 13105 8
Donor2-CAGGTGCTCTATCCCG-2 1923 10187 7
Donor2-CAGGTGCTCTGTTTGT-2 1997 11766 7
Donor2-CAGTAACCACCGCTAG-2 1728 5859 13
Donor2-CAGTCCTAGCGATGAC-2 4818 14256 6
Donor2-CAGTCCTAGTGCCATT-2 4225 12563 4
Donor2-CAGTCCTCACTTCTGC-2 3120 9457 4
Donor2-CAGTCCTGTGCGAAAC-2 2172 6201 13
Donor2-CATATGGCACGTCAGC-2 1799 11580 8
Donor2-CATATTCCATATACCG-2 1730 10197 8
Donor2-CATCAAGAGAACAACT-2 7979 58543 4
Donor2-CATCAAGCAAACCTAC-2 2908 10240 8

Donor2-CATCAGAAGGAGCGTT-2 5251 17172 5
Donor2-CATCAGATCAAACGGG-2 1333 5833 8
Donor2-CATCCACAGACAGACC-2 4196 11705 4
Donor2-CATCCACCAGGTCCAC-2 1831 9435 7
Donor2-CATCCACCATCACGTA-2 2913 15540 7
Donor2-CATCCACTCAGAGGTG-2 6673 44452 5
Donor2-CATCGAAAGGTGATAT-2 5706 22319 5
Donor2-CATCGAAAGTCACGCC-2 6358 38024 4
Donor2-CATCGAACAGCCTTTC-2 1784 10722 8
Donor2-CATCGAACAGCTGGCT-2 5578 21073 1
Donor2-CATCGAATCCGGGTGT-2 4428 23855 6
Donor2-CATCGAATCGTTGCCT-2 7681 42913 5
Donor2-CATCGAATCTGGGCCA-2 1904 9189 7
Donor2-CATCGGGCACTGCCAG-2 1600 6152 8
Donor2-CATCGGGGTTGATTGC-2 4442 13400 1
Donor2-CATCGGGTCATTTGGG-2 2986 15661 7
Donor2-CATGACAGTTTGACAC-2 6507 26130 1
Donor2-CATGCCTAGCTGCAAG-2 9152 71414 5
Donor2-CATGGCGCATTTGCCC-2 2129 6480 13
Donor2-CATGGCGTCACCACCT-2 2367 12099 7
Donor2-CATTATCAGCCTCGTG-2 5361 37084 6
Donor2-CATTATCCAGTTTACG-2 1682 7664 8
Donor2-CATTATCGTAAGCACG-2 2000 13493 8
Donor2-CATTCGCAGTCCATAC-2 1702 6275 13
Donor2-CATTCGCCAGTATCTG-2 1954 10292 7
Donor2-CCAATCCAGCCAACAG-2 3009 12125 6
Donor2-CCAATCCAGGTAGCTG-2 2100 7507 13
Donor2-CCAATCCAGTGGGCTA-2 2024 7163 6
Donor2-CCAATCCGTTAAAGAC-2 3350 14330 12
Donor2-CCACCTAAGAAGGACA-2 3009 14423 7
Donor2-CCACGGACAGACAAGC-2 3366 13397 11
Donor2-CCACGGATCTCCGGTT-2 3434 16768 7
Donor2-CCACGGATCTGGTATG-2 3852 16969 6
Donor2-CCACTACCAGTGGGAT-2 4451 18256 11
Donor2-CCAGCGAAGACATAAC-2 2215 7776 9
Donor2-CCAGCGAGTAATTGGA-2 6310 23276 5
Donor2-CCAGCGAGTACGCTGC-2 2196 6633 11
Donor2-CCAGCGATCGTCGTTC-2 6369 36788 4
Donor2-CCATGTCCAATGGTCT-2 4142 10809 1
Donor2-CCATTCGAGTACACCT-2 3058 8302 11
Donor2-CCATTCGTCAGTTGAC-2 6630 31464 6
Donor2-CCCAATCCACCGTTGG-2 3361 28588 7
Donor2-CCCAATCCAGACAAAT-2 3310 11965 5
Donor2-CCCAATCTCAAACGGG-2 1847 6492 9
Donor2-CCCAGTTGTCTCCATC-2 2683 5735 5
Donor2-CCCAGTTTCAACACGT-2 1775 6363 9
Donor2-CCCATACGTATAGTAG-2 2857 8703 11
Donor2-CCGGGATTCTGGTATG-2 5288 22446 6
Donor2-CCGGTAGAGCACCGCT-2 3089 16016 7
Donor2-CCGGTAGCAAGTTAAG-2 1998 6603 8
Donor2-CCGGTAGGTAAGTTCC-2 1786 7055 13
Donor2-CCGGTAGTCCCATTTA-2 1527 6274 13
Donor2-CCGTACTAGTGTCCCG-2 8919 71124 6
Donor2-CCGTACTAGTGTTTGC-2 2721 23266 8
Donor2-CCGTGGAAGCTCCTCT-2 4889 25354 6
Donor2-CCGTGGAGTTCTCATT-2 7513 46510 6

Donor2-CCGTGGATCTTGGGTA-2 2819 8463 10
Donor2-CCGTTCATCGTGACAT-2 8386 45614 5
Donor2-CCTAAAGCACCGATAT-2 5022 21691 6
Donor2-CCTACACAGGCATTGG-2 2372 15214 7
Donor2-CCTACACCACTTGGAT-2 5876 27436 6
Donor2-CCTACCACAAGCGCTC-2 6462 31815 4
Donor2-CCTACCATCAAAGACA-2 1754 8988 7
Donor2-CCTAGCTAGAAGAAGC-2 2116 8159 7
Donor2-CCTAGCTGTTTACTCT-2 2078 7136 9
Donor2-CCTATTACAATAGAGT-2 2084 9732 7
Donor2-CCTCAGTAGACTAGGC-2 5569 28519 5
Donor2-CCTCAGTCATCCTTGC-2 2683 8299 13
Donor2-CCTCAGTCATGTCCTC-2 3962 15853 5
Donor2-CCTCAGTGTCTAGTGT-2 5287 20132 6
Donor2-CCTCTGAAGAACAACT-2 2430 8816 6
Donor2-CCTCTGATCCTTTCGG-2 2418 5792 4
Donor2-CCTTACGGTCAAAGAT-2 2691 14091 8
Donor2-CCTTCCCAGGGTCGAT-2 2773 9057 11
Donor2-CCTTCCCCAGTAACGG-2 2719 8629 11
Donor2-CCTTCCCGTCGGGTCT-2 4245 15134 5
Donor2-CCTTCCCGTGTTCGAT-2 3720 9567 4
Donor2-CCTTCCCTCAGCAACT-2 2310 5951 4
Donor2-CCTTCGACACCGTTGG-2 2817 14844 7
Donor2-CCTTCGAGTCGAGATG-2 4097 14963 5
Donor2-CCTTCGAGTTCAGACT-2 3602 15858 6
Donor2-CCTTTCTAGATCCCAT-2 1557 8763 8
Donor2-CCTTTCTGTACATGTC-2 5546 18556 5
Donor2-CCTTTCTTCGCCTGTT-2 2281 14561 8
Donor2-CCTTTCTTCGTACGGC-2 6702 30803 6
Donor2-CGAACATAGAGGACGG-2 2344 9716 7
Donor2-CGACCTTAGATCTGAA-2 4076 13676 11
Donor2-CGACCTTGTTGTACAC-2 2041 8343 8
Donor2-CGACTTCTCCCATTTA-2 3400 7625 1
Donor2-CGACTTCTCGTGACAT-2 5339 30937 6
Donor2-CGAGAAGAGCAATCTC-2 1306 6286 8
Donor2-CGAGAAGCATCACGAT-2 2682 12439 7
Donor2-CGAGAAGGTGATGTCT-2 4177 22486 7
Donor2-CGAGCCAAGAAACGAG-2 2893 6796 6
Donor2-CGATCGGAGTGTTGAA-2 7388 38444 5
Donor2-CGATCGGCATCCTTGC-2 4039 10167 5
Donor2-CGATCGGTCGGTCCGA-2 3460 8769 4
Donor2-CGATGTAAGCCTATGT-2 3303 12713 10
Donor2-CGATGTATCCCTTGTG-2 1591 6757 7
Donor2-CGATTGAAGAATCTCC-2 3283 14747 7
Donor2-CGATTGAAGCCGCCTA-2 4661 14623 6
Donor2-CGATTGACAATCAGAA-2 2454 7110 11
Donor2-CGCCAAGAGCTCTCGG-2 1849 5655 7
Donor2-CGCCAAGAGTTACCCA-2 5346 16630 5
Donor2-CGCGGTAGTCATATGC-2 7985 55250 6
Donor2-CGCGTTTAGTGTCCCG-2 2612 13148 7
Donor2-CGCGTTTCAGTGGAGT-2 2100 12730 7
Donor2-CGCGTTTTCCTTAATC-2 5862 20592 5
Donor2-CGCTATCAGCTGCAAG-2 1422 7677 8
Donor2-CGCTATCCATGCCCGA-2 5140 17476 1
Donor2-CGCTATCGTCCTCTTG-2 6226 33543 4
Donor2-CGCTGGAAGTCGCCGT-2 2611 11106 10

Donor2-CGCTGGACAGGATTGG-2 3378 16901 6
Donor2-CGCTGGATCTATCCCG-2 2264 10456 7
Donor2-CGGACACAGTGGAGAA-2 1932 7535 13
Donor2-CGGACACGTGCAGTAG-2 3397 14196 6
Donor2-CGGACGTGTTGTACAC-2 2711 16084 7
Donor2-CGGACTGAGACAGGCT-2 4437 12390 6
Donor2-CGGACTGAGACCCACC-2 2667 8893 10
Donor2-CGGACTGGTATGAATG-2 2551 9890 10
Donor2-CGGACTGGTCCGCTGA-2 2721 10270 11
Donor2-CGGACTGGTCTAAAGA-2 2151 8722 7
Donor2-CGGACTGGTCTAGGTT-2 4431 12124 6
Donor2-CGGACTGTCCTGTAGA-2 5123 30882 5
Donor2-CGGAGCTAGTTAGGTA-2 4230 10453 5
Donor2-CGGAGTCTCTACCAGA-2 2667 6255 2
Donor2-CGGCTAGAGGGAACGG-2 7862 80128 5
Donor2-CGGCTAGAGTCACGCC-2 6403 28916 1
Donor2-CGGCTAGTCAGTACGT-2 3788 9239 1
Donor2-CGGGTCAAGAAACCGC-2 1729 7590 7
Donor2-CGGGTCAAGATGTGTA-2 3304 22621 7
Donor2-CGGGTCAAGGCCCGTT-2 3713 12638 11
Donor2-CGGGTCACATGCAACT-2 3312 9108 6
Donor2-CGGTTAATCCGCATCT-2 3350 20133 7
Donor2-CGTAGGCAGCGTTGCC-2 1470 6622 8
Donor2-CGTAGGCCACATTCGA-2 5099 16875 6
Donor2-CGTAGGCGTGTGAATA-2 2947 6666 3
Donor2-CGTAGGCGTTCCGGCA-2 5123 15962 5
Donor2-CGTAGGCTCGTCACGG-2 3293 7902 5
Donor2-CGTCACTAGAAACGCC-2 1882 8279 8
Donor2-CGTCACTAGGCGATAC-2 5270 18174 4
Donor2-CGTCACTCACAGATTC-2 5997 22736 5
Donor2-CGTCACTGTCCGAATT-2 1842 8130 8
Donor2-CGTCACTTCGCACTCT-2 7662 43157 2
Donor2-CGTCAGGCAATTGCTG-2 2250 11782 7
Donor2-CGTCAGGGTAGAGCTG-2 2456 10785 7
Donor2-CGTCCATAGTGCCAGA-2 1305 5821 8
Donor2-CGTCCATCACCAACCG-2 3240 13587 6
Donor2-CGTCCATGTATGAATG-2 3767 10040 3
Donor2-CGTCCATTCACATAGC-2 2074 5699 13
Donor2-CGTCCATTCCAATGGT-2 1856 6131 9
Donor2-CGTGAGCCAGCTCGCA-2 6481 34527 6
Donor2-CGTGAGCGTCCGAATT-2 2778 8076 11
Donor2-CGTGTAAAGCTCTCGG-2 1854 6513 8
Donor2-CGTGTAAGTTACGTCA-2 4884 16608 1
Donor2-CGTTAGAAGTGAACAT-2 3394 10339 11
Donor2-CGTTCTGAGAGGTACC-2 6026 38072 6
Donor2-CGTTCTGCAGATGAGC-2 4841 18198 5
Donor2-CGTTCTGTCCGCTGTT-2 2854 7746 6
Donor2-CGTTGGGAGCTGTCTA-2 2159 7241 9
Donor2-CGTTGGGCAAGCGTAG-2 3160 12100 11
Donor2-CTAACTTAGCGATATA-2 2821 6653 6
Donor2-CTAACTTAGGACAGCT-2 2884 8503 11
Donor2-CTAACTTCAATAGCAA-2 2027 9607 7
Donor2-CTAACTTCAGACTCGC-2 3501 11560 13
Donor2-CTAACTTCATGCGCAC-2 1605 7145 7
Donor2-CTAACTTGTTAAAGTG-2 4521 13711 1
Donor2-CTAAGACGTGAGCGAT-2 1948 10892 8

Donor2-CTAATGGAGTCCATAC-2 6566 36563 6
Donor2-CTAATGGAGTGGGATC-2 5082 15493 5
Donor2-CTAATGGCACCCATGG-2 2392 7823 10
Donor2-CTAATGGTCTGCTGTC-2 2144 8399 9
Donor2-CTACACCCAGTACACT-2 3639 21951 7
Donor2-CTACACCTCTTGACGA-2 1857 12571 8
Donor2-CTACATTAGGGTCTCC-2 3707 12962 11
Donor2-CTACATTTCCGATATG-2 9274 83675 5
Donor2-CTACCCAAGTCCTCCT-2 1581 8787 8
Donor2-CTACCCAGTACTCGCG-2 4470 24025 6
Donor2-CTACGTCAGTACTTGC-2 1808 7379 8
Donor2-CTAGAGTGTGAGTATA-2 5568 26184 6
Donor2-CTAGCCTAGCTGTTCA-2 3996 9327 5
Donor2-CTAGCCTCATGCCTAA-2 3366 22366 7
Donor2-CTCACACGTAATCACC-2 3815 22642 7
Donor2-CTCACACGTGGTCTCG-2 3547 8078 2
Donor2-CTCACACGTTCGTGAT-2 1545 8766 8
Donor2-CTCACACGTTGAACTC-2 1559 6458 7
Donor2-CTCACACTCCTGTACC-2 2477 7626 11
Donor2-CTCAGAAGTCAATGTC-2 2678 10377 7
Donor2-CTCAGAATCCTAGTGA-2 1807 6343 10
Donor2-CTCATTAGTCCTAGCG-2 3673 12259 6
Donor2-CTCATTAGTGACGCCT-2 3666 10175 6
Donor2-CTCCTAGCACCAGATT-2 6906 33036 4
Donor2-CTCGAAAAGACACGAC-2 2580 16032 8
Donor2-CTCGAAAAGGACAGCT-2 2416 7881 13
Donor2-CTCGAAACAATGGTCT-2 2059 13437 8
Donor2-CTCGAAACAGGTCCAC-2 2657 15788 7
Donor2-CTCGAGGAGGCAGGTT-2 1596 6125 10
Donor2-CTCGAGGCACGAAATA-2 2358 13442 7
Donor2-CTCGAGGGTCCTCTTG-2 2540 12367 7
Donor2-CTCGGAGAGCTGTTCA-2 2966 9466 11
Donor2-CTCGGGAAGAATGTTG-2 2003 9715 8
Donor2-CTCGGGATCCTAGAAC-2 5926 35758 6
Donor2-CTCGTACAGATCGATA-2 2263 10859 7
Donor2-CTCGTACAGATCTGAA-2 2285 16888 8
Donor2-CTCGTACCACCCTATC-2 6760 41708 4
Donor2-CTCGTACTCTCTGAGA-2 1802 9459 8
Donor2-CTCGTCAAGGAGTAGA-2 4467 29030 7
Donor2-CTCGTCATCCGTTGTC-2 1990 7077 11
Donor2-CTCTAATCACCGTTGG-2 4200 14828 11
Donor2-CTCTACGGTCTCTCGT-2 5377 22100 5
Donor2-CTCTACGTCCATTCTA-2 2481 7291 13
Donor2-CTGAAACTCACATACG-2 4807 12150 1
Donor2-CTGAAGTAGGCACATG-2 4821 12101 5
Donor2-CTGAAGTCACATGGGA-2 3031 11546 11
Donor2-CTGAAGTCAGCTGGCT-2 2499 9905 9
Donor2-CTGAAGTTCAAACGGG-2 7104 55608 5
Donor2-CTGATCCGTTAAAGTG-2 3374 11402 11
Donor2-CTGATCCTCAAGGCTT-2 9188 128577 6
Donor2-CTGATCCTCAGAGCTT-2 7114 60290 4
Donor2-CTGCCTAAGGACAGAA-2 3516 11197 6
Donor2-CTGCCTAAGTTACCCA-2 3775 8590 5
Donor2-CTGCCTAGTCCGTGAC-2 1594 6422 13
Donor2-CTGCCTATCGTCTGCT-2 5686 20680 5
Donor2-CTGCTGTTCTTACCTA-2 2607 7590 11

Donor2-CTGGTCTGTGGGTCAA-2 2354 8950 7
Donor2-CTGTGCTCAGCATGAG-2 2773 6040 6
Donor2-CTGTTTACATGGTAGG-2 2098 9716 7
Donor2-CTTAACTAGGAGTAGA-2 2128 6273 11
Donor2-CTTAACTGTCTAAACC-2 5906 21322 6
Donor2-CTTACCGCAACCGCCA-2 4307 14104 6
Donor2-CTTACCGCATACTCTT-2 3380 8869 5
Donor2-CTTACCGGTAGCGTGA-2 4728 14727 1
Donor2-CTTACCGGTGTTTGGT-2 2863 15559 7
Donor2-CTTACCGTCCCTTGCA-2 3471 13662 6
Donor2-CTTACCGTCTTGTACT-2 4583 13776 6
Donor2-CTTAGGACACCCATTC-2 3954 11090 4
Donor2-CTTAGGACAGTATAAG-2 4978 16235 6
Donor2-CTTAGGAGTTCCGGCA-2 5171 17057 4
Donor2-CTTAGGATCGGCCGAT-2 1671 7930 7
Donor2-CTTCTCTAGAAACCTA-2 8175 60844 1
Donor2-CTTCTCTAGATGTTAG-2 2169 7854 13
Donor2-CTTCTCTAGCTTTGGT-2 2558 9919 6
Donor2-CTTTGCGCACGAAAGC-2 3020 17991 7
Donor2-CTTTGCGCATCACAAC-2 3446 14094 6
Donor2-CTTTGCGGTGGGTATG-2 2026 9155 7
Donor2-CTTTGCGTCCTATGTT-2 1810 10100 8
Donor2-GAAACTCCAAGCCCAC-2 3098 10048 11
Donor2-GAAACTCCAAGCCTAT-2 6094 26394 6
Donor2-GAAACTCGTCACCTAA-2 4066 11187 1
Donor2-GAAACTCGTCTCTTTA-2 2021 7282 13
Donor2-GAAACTCGTTGCGTTA-2 7923 45230 5
Donor2-GAAACTCTCATGGTCA-2 1990 7409 9
Donor2-GAACATCCAGATCCAT-2 3278 10605 11
Donor2-GAACATCGTCTACCTC-2 1456 6055 8
Donor2-GAACATCGTGGTTTCA-2 2252 9271 9
Donor2-GAACATCGTTGTACAC-2 1659 5759 7
Donor2-GAACATCTCGGATGTT-2 3470 8749 1
Donor2-GAACCTACACGAAAGC-2 2783 15813 7
Donor2-GAACCTACAGCAGTTT-2 3331 7474 2
Donor2-GAACCTAGTCTAACGT-2 2229 6983 13
Donor2-GAACGGACAGCGTCCA-2 5640 19202 5
Donor2-GAACGGACAGCGTTCG-2 2064 6500 13
Donor2-GAACGGAGTGTGGTTT-2 2699 9638 10
Donor2-GAACGGAGTTGCGTTA-2 5723 21573 5
Donor2-GAACGGATCCGATATG-2 4884 30169 6
Donor2-GAACGGATCTTCGAGA-2 3514 12064 11
Donor2-GAAGCAGAGGTGTTAA-2 1486 7551 8
Donor2-GAAGCAGCAAGTAATG-2 5436 21339 3
Donor2-GAAGCAGGTACTTCTT-2 5042 24870 6
Donor2-GAAGCAGGTTCACCTC-2 4460 11620 5
Donor2-GAATAAGGTGTGCCTG-2 1878 7146 7
Donor2-GAATGAAAGTCGATAA-2 1198 6081 8
Donor2-GACACGCTCTGGAGCC-2 2511 12931 7
Donor2-GACCAATAGCTGAACG-2 2458 6616 11
Donor2-GACCAATTCGAGAACG-2 2594 8397 13
Donor2-GACCTGGAGTCGTTTG-2 3452 23342 7
Donor2-GACCTGGGTAAACGCG-2 2082 7263 13
Donor2-GACCTGGGTCATGCCG-2 2736 6154 3
Donor2-GACGCGTAGAAACCGC-2 5899 36150 5
Donor2-GACGCGTAGAGACTTA-2 1324 6915 8

Donor2-GACGCGTGTACCGAGA-2 3060 8904 11
Donor2-GACGCGTGTCTGATCA-2 1698 8858 8
Donor2-GACGCGTGTGCCTGCA-2 2440 8822 10
Donor2-GACGGCTAGGGTGTGT-2 2747 13401 7
Donor2-GACGTGCCAGTATAAG-2 2213 8448 6
Donor2-GACGTTAAGTACATGA-2 2349 9217 9
Donor2-GACGTTATCGCGATCG-2 6587 56405 5
Donor2-GACGTTATCGGACAAG-2 1678 7070 13
Donor2-GACTAACCACAAGTAA-2 4734 13111 1
Donor2-GACTAACCATGTCGAT-2 5625 22284 3
Donor2-GACTAACTCCTCAACC-2 2417 7892 11
Donor2-GACTAACTCTCAAGTG-2 1478 6660 8
Donor2-GACTAACTCTTTAGGG-2 2521 19141 8
Donor2-GACTACACAGTCGTGC-2 4846 16015 6
Donor2-GACTACACATGTAGTC-2 3996 22749 6
Donor2-GACTACATCCTTGGTC-2 4985 15277 1
Donor2-GACTGCGGTCTGATTG-2 3094 11970 10
Donor2-GACTGCGGTTCGTTGA-2 5058 18208 5
Donor2-GACTGCGTCTGGCGAC-2 6743 30749 6
Donor2-GAGGTGAAGCCCAATT-2 4022 13225 6
Donor2-GAGGTGACACAAGTAA-2 2356 8702 9
Donor2-GAGGTGAGTATAGTAG-2 6256 26669 5
Donor2-GAGTCCGAGGGCATGT-2 1804 5997 13
Donor2-GAGTCCGGTACGCTGC-2 2105 9047 7
Donor2-GAGTCCGTCAATAAGG-2 1687 9425 8
Donor2-GATCAGTGTCCGAAGA-2 5606 29359 5
Donor2-GATCGATAGGATGGAA-2 2168 7270 10
Donor2-GATCGATGTAGTGAAT-2 4650 21905 6
Donor2-GATCGTACAGCTCCGA-2 1968 5841 13
Donor2-GATCGTAGTTGATTGC-2 2328 9675 9
Donor2-GATCTAGGTCACTGGC-2 3155 10110 11
Donor2-GATCTAGTCGTCCGTT-2 3150 11154 6
Donor2-GATGAAACACCATGTA-2 3823 10040 3
Donor2-GATGAAACACTCGACG-2 5603 17200 6
Donor2-GATGAAACATGAAGTA-2 4016 28416 7
Donor2-GATGAGGAGAAGGTTT-2 4861 17631 6
Donor2-GATGAGGGTTGGTTTG-2 1572 7132 8
Donor2-GATGCTAAGTCCATAC-2 6966 34329 5
Donor2-GATTCAGTCTTACCTA-2 2607 9291 13
Donor2-GCAAACTAGCAGGTCA-2 2596 10750 7
Donor2-GCAAACTAGCGATTCT-2 9403 90690 6
Donor2-GCAAACTGTTCCATGA-2 3409 25159 7
Donor2-GCAAACTTCCTCCTAG-2 4496 65870 8
Donor2-GCAATCACAAGCGAGT-2 8540 56850 5
Donor2-GCAATCAGTAAACGCG-2 3860 10898 6
Donor2-GCACATATCACCCTCA-2 2091 8056 7
Donor2-GCACTCTAGCGTCTAT-2 1974 9980 7
Donor2-GCACTCTCACTGTGTA-2 2435 7038 11
Donor2-GCAGCCACAGGACCCT-2 5369 25874 5
Donor2-GCAGCCAGTCAACATC-2 2280 11834 8
Donor2-GCAGCCAGTTCTGGTA-2 2440 7534 11
Donor2-GCAGTTACATTGTGCA-2 8442 47726 1
Donor2-GCAGTTAGTAAATACG-2 2573 17152 7
Donor2-GCAGTTATCCGAGCCA-2 3599 8783 4
Donor2-GCATGATAGACTTGAA-2 3095 17484 7
Donor2-GCATGATAGTGCAAGC-2 7756 54668 4

Donor2-GCATGCGGTAACGTTC-2 4687 13106 2
Donor2-GCATGTATCTACCTGC-2 5338 34590 6
Donor2-GCCAAATTCACAAACC-2 5549 20321 6
Donor2-GCCTCTACAGTCGATT-2 1771 6876 7
Donor2-GCCTCTAGTGGGTATG-2 3492 18874 7
Donor2-GCGACCAAGATGAGAG-2 3286 7425 3
Donor2-GCGACCAGTTAAAGAC-2 2292 10550 7
Donor2-GCGCAACTCGAATGCT-2 3407 15173 6
Donor2-GCGCCAAAGCGACGTA-2 4905 15899 1
Donor2-GCGCCAAAGCTGGAAC-2 4488 14862 6
Donor2-GCGCCAACAGACACTT-2 6322 30446 6
Donor2-GCGCGATCAGGAATCG-2 5846 22842 5
Donor2-GCGGGTTCATTTGCCC-2 1553 6143 8
Donor2-GCTCCTAGTCGACTAT-2 8522 36188 6
Donor2-GCTCCTATCCGCGGTA-2 3992 10095 1
Donor2-GCTCCTATCTAACGGT-2 4039 11772 3
Donor2-GCTCTGTCACATCTTT-2 2397 13239 7
Donor2-GCTCTGTGTCTCATCC-2 6286 30388 6
Donor2-GCTCTGTTCCAAAGTC-2 2207 11777 8
Donor2-GCTGCAGAGAACAATC-2 5786 19540 3
Donor2-GCTGCAGAGTTCGCGC-2 4609 29955 7
Donor2-GCTGCGAAGTCGCCGT-2 4923 21810 6
Donor2-GCTGCTTAGCTGATAA-2 1888 6709 9
Donor2-GCTGCTTAGGAGTAGA-2 6418 32953 4
Donor2-GCTGCTTCACGAGAGT-2 2094 6896 13
Donor2-GCTGCTTTCACCTTAT-2 1929 10879 8
Donor2-GCTGCTTTCTAACTGG-2 4218 12704 5
Donor2-GCTGGGTCACGGTTTA-2 1755 9705 8
Donor2-GCTGGGTGTAAGTAGT-2 2094 11638 8
Donor2-GCTGGGTGTCTAAAGA-2 4387 12503 1
Donor2-GCTGGGTTCTTTAGGG-2 2588 7922 11
Donor2-GCTTGAATCATATCGG-2 5015 16301 1
Donor2-GCTTGAATCTGTGCAA-2 6657 50142 5
Donor2-GGAAAGCCACTAGTAC-2 1714 7488 7
Donor2-GGAAAGCCACTATCTT-2 2390 16273 7
Donor2-GGAAAGCTCGCGCCAA-2 4819 15820 1
Donor2-GGAAAGCTCTGCCCTA-2 2864 24715 8
Donor2-GGAACTTGTCCGTTAA-2 2315 6444 13
Donor2-GGAATAATCAGAGACG-2 2647 7708 11
Donor2-GGAATAATCTGTGCAA-2 2547 9877 6
Donor2-GGACAAGAGAGTCTGG-2 2091 7949 9
Donor2-GGACAGAAGCCACGTC-2 3659 12641 11
Donor2-GGACAGACAGTGAGTG-2 1453 6871 8
Donor2-GGACAGATCAACACCA-2 7215 47622 6
Donor2-GGACATTTCATAAAGG-2 4379 28276 7
Donor2-GGACATTTCCCTGACT-2 4924 15733 6
Donor2-GGACATTTCGAATGGG-2 2689 7161 5
Donor2-GGAGCAAAGCAGATCG-2 2622 8921 11
Donor2-GGAGCAAAGGCCCTTG-2 2456 13242 7
Donor2-GGAGCAATCCGCTGTT-2 1434 5951 8
Donor2-GGAGCAATCGAGAGCA-2 4037 23335 6
Donor2-GGATGTTTCTACCTGC-2 2073 6853 9
Donor2-GGATTACAGTTACGGG-2 3204 7949 3
Donor2-GGATTACGTATTACCG-2 2775 11974 6
Donor2-GGATTACGTGCAGTAG-2 3900 15635 5
Donor2-GGATTACGTGTGCCTG-2 9725 98811 6

Donor2-GGCAATTGTTGCGTTA-2 5730 22666 6
Donor2-GGCCGATGTAAATGAC-2 6470 25497 5
Donor2-GGCCGATGTATAGTAG-2 1546 7644 8
Donor2-GGCCGATGTCTCACCT-2 1663 6828 7
Donor2-GGCGACTAGAAACGAG-2 2270 12578 7
Donor2-GGCGACTGTAAATGTG-2 3794 8980 5
Donor2-GGCGACTTCTGCAGTA-2 2065 9434 7
Donor2-GGCGTGTAGGGCTCTC-2 2758 9173 10
Donor2-GGCGTGTAGGGTGTTG-2 1525 7192 8
Donor2-GGCGTGTGTAAGGGCT-2 1325 7199 8
Donor2-GGCGTGTGTGCACGAA-2 2199 9308 9
Donor2-GGCTGGTTCTAAGCCA-2 5532 20714 6
Donor2-GGGACCTAGATGTTAG-2 1812 9150 8
Donor2-GGGACCTGTAGCTGCC-2 2495 11296 9
Donor2-GGGACCTTCGAGCCCA-2 4400 24419 6
Donor2-GGGATGAAGTTGAGAT-2 6170 26313 6
Donor2-GGGCACTCATAACCTG-2 3582 28991 7
Donor2-GGGCACTTCCCATTAT-2 1723 8934 7
Donor2-GGGTCTGCAGACACTT-2 4698 15680 5
Donor2-GGTATTGTCCAGATCA-2 3602 8966 1
Donor2-GGTGAAGAGCTCCCAG-2 8747 75806 6
Donor2-GGTGAAGTCCTGTAGA-2 1731 6560 13
Donor2-GGTGCGTAGGTTACCT-2 1819 6929 7
Donor2-GGTGCGTGTCCGCTGA-2 1638 6277 7
Donor2-GGTGCGTGTCTACCTC-2 3009 10508 11
Donor2-GGTGTTAAGAGTACCG-2 1458 6002 7
Donor2-GGTGTTAAGGATCGCA-2 3289 7151 3
Donor2-GGTGTTAGTGGTCTCG-2 7112 50650 5
Donor2-GTAACGTAGTAGCGGT-2 2167 8421 10
Donor2-GTAACGTCAAGGACTG-2 2684 11868 7
Donor2-GTAACGTGTAGGACAC-2 5842 27621 6
Donor2-GTAACTGAGTACATGA-2 1979 7907 6
Donor2-GTAACTGGTCATGCCG-2 1541 9002 8
Donor2-GTAACTGGTCGAGTTT-2 2969 13823 6
Donor2-GTAACTGTCCAAGCCG-2 4970 30316 6
Donor2-GTACGTAAGTAGTGCG-2 3323 10315 11
Donor2-GTACTCCAGGATGGAA-2 3911 9539 3
Donor2-GTACTCCCACTGAAGG-2 2449 17232 8
Donor2-GTACTCCGTTCGTGAT-2 7685 58296 6
Donor2-GTACTCCTCAACCATG-2 5554 19277 6
Donor2-GTACTCCTCGGTCTAA-2 3800 12428 8
Donor2-GTACTTTAGATGCCTT-2 6880 36733 1
Donor2-GTACTTTAGTGGGCTA-2 1468 6753 8
Donor2-GTACTTTCACGACTCG-2 1926 6531 9
Donor2-GTACTTTTCTCGCTTG-2 8031 52484 5
Donor2-GTAGGCCAGACTAGAT-2 1807 12391 8
Donor2-GTAGGCCGTACCAGTT-2 5324 18221 4
Donor2-GTAGGCCTCAAGGCTT-2 4899 14721 2
Donor2-GTAGTCAAGATAGCAT-2 6450 38320 4
Donor2-GTAGTCACAATCAGAA-2 5429 18344 2
Donor2-GTAGTCAGTGGAAAGA-2 2016 7129 13
Donor2-GTAGTCAGTTAAGAAC-2 4561 13739 5
Donor2-GTATCTTAGAAACCAT-2 4534 16047 1
Donor2-GTATTCTAGTCACGCC-2 2580 7045 11
Donor2-GTATTCTTCCGCGCAA-2 1953 5716 11
Donor2-GTATTCTTCGTCACGG-2 2352 13819 8

Donor2-GTCAAGTGTCCGAAGA-2 1775 7545 7
Donor2-GTCACAAGTCGCGGTT-2 3189 7176 3
Donor2-GTCACAATCGGAATCT-2 9842 90410 5
Donor2-GTCACGGAGGCATGTG-2 5496 18115 5
Donor2-GTCACGGCATCTGGTA-2 3376 23080 7
Donor2-GTCACGGGTAAGGGAA-2 8829 67135 5
Donor2-GTCACGGGTAGGGTAC-2 4499 33977 7
Donor2-GTCACGGTCAGCACAT-2 2189 11979 7
Donor2-GTCACGGTCATAAAGG-2 2462 15946 8
Donor2-GTCATTTAGGAATTAC-2 6574 51271 6
Donor2-GTCATTTCACAGAGGT-2 2194 11928 7
Donor2-GTCATTTTCCTTTCTC-2 7377 41103 6
Donor2-GTCATTTTCGTCCGTT-2 3625 8998 3
Donor2-GTCATTTTCTGCCCTA-2 7732 42358 5
Donor2-GTCCTCAGTCCCTACT-2 3742 9593 1
Donor2-GTCCTCATCGCTAGCG-2 1717 6531 7
Donor2-GTCGGGTAGTGGTAAT-2 1421 6712 8
Donor2-GTCTCGTCACAGTCGC-2 1431 5783 7
Donor2-GTGAAGGCAGGTGCCT-2 3727 8955 2
Donor2-GTGCAGCCAGGGTACA-2 1771 7441 7
Donor2-GTGCATAAGCGATGAC-2 2782 10484 13
Donor2-GTGCATAAGGCAGTCA-2 3747 26119 7
Donor2-GTGCATAGTGACGGTA-2 3951 22131 6
Donor2-GTGCGGTGTGTAACGG-2 1704 5928 13
Donor2-GTGCGGTTCGAATGCT-2 7011 31992 5
Donor2-GTGCTTCAGCGCCTTG-2 1484 5751 8
Donor2-GTGCTTCTCTATGTGG-2 2904 9311 11
Donor2-GTGTTAGAGCAGGCTA-2 4167 11998 1
Donor2-GTGTTAGAGCTAAGAT-2 2942 8733 4
Donor2-GTGTTAGAGGACCACA-2 2484 7251 11
Donor2-GTGTTAGCACAGACTT-2 7479 59659 5
Donor2-GTTAAGCCACGGCGTT-2 4719 13045 5
Donor2-GTTAAGCCATTCCTGC-2 1477 6422 8
Donor2-GTTAAGCGTCCATCCT-2 1418 6767 8
Donor2-GTTCATTTCACAGGCC-2 3665 9354 4
Donor2-GTTCATTTCCACGTTC-2 1689 6238 13
Donor2-GTTCATTTCTACCAGA-2 2794 8439 11
Donor2-GTTCGGGTCACCACCT-2 4143 36901 7
Donor2-GTTCTCGTCGACCAGC-2 6251 27866 4
Donor2-GTTTCTAGTATAAACG-2 8318 39623 3
Donor2-GTTTCTATCGGGAGTA-2 6308 35592 6
Donor2-TAAACCGAGGAATTAC-2 3060 10451 11
Donor2-TAAACCGGTTGTTTGG-2 7156 47624 5
Donor2-TAAGAGAGTCATTAGC-2 6504 29246 5
Donor2-TAAGAGATCCAGGGCT-2 4761 13477 1
Donor2-TAAGAGATCCCTTGCA-2 2658 5812 5
Donor2-TAAGCGTAGAAGATTC-2 3959 23224 7
Donor2-TAAGCGTCAAGTACCT-2 3489 7971 4
Donor2-TAAGCGTGTCGCTTCT-2 2581 7225 11
Donor2-TAAGCGTTCAGGCGAA-2 1916 6227 13
Donor2-TAAGTGCAGCCGATTT-2 5461 20038 5
Donor2-TAAGTGCCAAACCTAC-2 1410 5731 8
Donor2-TAAGTGCGTGTGACGA-2 1956 12740 8
Donor2-TAAGTGCGTGTTGAGG-2 3282 18898 7
Donor2-TACACGACAGTATGCT-2 5282 26475 6
Donor2-TACACGATCATTTGGG-2 2147 5790 13

Donor2-TACACGATCGAATGCT-2 3786 19120 6
Donor2-TACAGTGAGGCACATG-2 2036 7217 11
Donor2-TACAGTGCATTCCTCG-2 4574 13118 4
Donor2-TACAGTGGTACTCAAC-2 2317 9735 7
Donor2-TACCTATAGTATGACA-2 5876 22843 1
Donor2-TACCTATGTCACCTAA-2 2690 7766 13
Donor2-TACCTTAGTAGCGCAA-2 7007 28173 3
Donor2-TACGGATCAGGATCGA-2 5345 35195 6
Donor2-TACGGGCGTCCAGTAT-2 3353 12029 11
Donor2-TACGGGCGTTCCCTTG-2 5689 18272 5
Donor2-TACGGTAAGCTTATCG-2 3138 13603 6
Donor2-TACGGTACAAGACGTG-2 2260 14259 8
Donor2-TACGGTACAATCGGTT-2 5331 24748 6
Donor2-TACGGTACAGGAATCG-2 2809 8506 5
Donor2-TACGGTATCGTGTAGT-2 5229 19073 4
Donor2-TACTCATAGCTCAACT-2 5184 16690 1
Donor2-TACTCATGTCTTCAAG-2 8000 50842 6
Donor2-TACTCATTCACGCATA-2 2950 14310 7
Donor2-TACTCGCAGTTAGCGG-2 2157 11279 8
Donor2-TACTTGTAGGAATTAC-2 5570 29804 6
Donor2-TACTTGTGTTCCCTTG-2 2567 10650 7
Donor2-TAGACCACACTAGTAC-2 3213 17535 7
Donor2-TAGACCATCCAATGGT-2 2517 7279 11
Donor2-TAGAGCTCAATGGATA-2 1590 7300 8
Donor2-TAGCCGGGTGCATCTA-2 5720 19990 5
Donor2-TAGGCATTCAAGCCTA-2 4412 12171 6
Donor2-TAGTGGTAGACAGAGA-2 3032 16682 7
Donor2-TAGTGGTAGGATGGAA-2 1871 7437 9
Donor2-TAGTGGTGTCAAAGAT-2 1526 8870 8
Donor2-TAGTGGTGTCCCTTGT-2 1921 10832 7
Donor2-TAGTGGTGTTGATTCG-2 7884 44871 6
Donor2-TAGTTGGAGCACAGGT-2 2958 6418 6
Donor2-TAGTTGGCATGGTCAT-2 6733 24804 1
Donor2-TAGTTGGGTCTGCAAT-2 2598 8804 10
Donor2-TAGTTGGTCACTATTC-2 4411 15731 6
Donor2-TAGTTGGTCCCTAACC-2 6028 29172 6
Donor2-TATCAGGTCCTTTCGG-2 4535 12470 5
Donor2-TATCTCACACTGAAGG-2 1782 5893 13
Donor2-TATCTCAGTATAGGGC-2 1921 6545 13
Donor2-TATCTCAGTCTGCCAG-2 1556 9433 8
Donor2-TATCTCATCTGCGTAA-2 5696 18811 5
Donor2-TATGCCCAGCAGATCG-2 5229 27814 6
Donor2-TCAACGACACCGGAAA-2 4904 20919 5
Donor2-TCAACGATCTGTTTGT-2 2829 8610 11
Donor2-TCAATCTGTACTCTCC-2 5242 16743 5
Donor2-TCACAAGTCGTTTGCC-2 1886 8106 9
Donor2-TCACGAATCACGGTTA-2 1927 6739 10
Donor2-TCAGATGTCGCTAGCG-2 6579 32852 6
Donor2-TCAGCAAAGGGTTTCT-2 2015 6135 13
Donor2-TCAGCAATCTGCTTGC-2 2255 10083 9
Donor2-TCAGCTCAGGAGCGTT-2 5331 20893 5
Donor2-TCATTACAGCCAGTAG-2 3337 8333 1
Donor2-TCATTACAGTTGTAGA-2 6926 49074 6
Donor2-TCATTACCACATTTCT-2 2973 13839 7
Donor2-TCATTACGTTGAGTTC-2 2360 9333 9
Donor2-TCATTTGAGCCCAGCT-2 3736 13632 11

Donor2-TCATTTGCATGTCCTC-2 6985 40557 6
Donor2-TCGAGGCAGAGTACAT-2 4083 10032 3
Donor2-TCGAGGCAGCGACGTA-2 5050 26758 5
Donor2-TCGCGAGCAGGGTTAG-2 3609 8910 6
Donor2-TCGCGAGGTACATCCA-2 5492 22051 4
Donor2-TCGCGAGGTGACTACT-2 3831 25984 7
Donor2-TCGCGAGGTTCGTGAT-2 2481 6006 11
Donor2-TCGCGTTAGCTACCTA-2 8076 48545 5
Donor2-TCGGGACAGTTGTAGA-2 2480 6133 11
Donor2-TCGGTAACACAGGAGT-2 4053 10565 1
Donor2-TCGGTAAGTCGCGAAA-2 9077 85275 6
Donor2-TCGTACCAGGAGCGAG-2 3848 9668 4
Donor2-TCTATTGGTATAGGGC-2 1934 7857 13
Donor2-TCTCTAAAGAGTGAGA-2 2210 6702 6
Donor2-TCTCTAAAGCACGCCT-2 7626 68329 6
Donor2-TCTCTAATCCCATTTA-2 5100 16291 1
Donor2-TCTGAGATCAAACCGT-2 1215 5812 8
Donor2-TCTGAGATCCATGAGT-2 3866 24905 7
Donor2-TCTTCGGCATATGCTG-2 1249 6303 8
Donor2-TCTTCGGCATGCGCAC-2 3950 9809 3
Donor2-TGAAAGAAGACTAGAT-2 2222 10468 7
Donor2-TGACAACAGTGTGGCA-2 3321 8224 3
Donor2-TGACAACCACACCGCA-2 4375 11547 6
Donor2-TGACAACGTAAGGATT-2 4238 31455 7
Donor2-TGACAACTCCCTTGCA-2 2313 9481 9
Donor2-TGACGGCCAAAGGCGT-2 6880 32998 1
Donor2-TGACTAGAGCCAACAG-2 6241 26062 5
Donor2-TGACTAGCAAGAGTCG-2 2379 11609 7
Donor2-TGACTAGGTATGAAAC-2 6807 29686 5
Donor2-TGACTTTAGATAGGAG-2 3933 9257 5
Donor2-TGACTTTAGTACGCCC-2 2614 6017 4
Donor2-TGACTTTGTAAGTTCC-2 2466 8678 10
Donor2-TGAGAGGCAGACAAAT-2 2262 6493 13
Donor2-TGAGAGGCAGCTTCGG-2 3833 10068 4
Donor2-TGAGAGGGTCTAGAGG-2 2931 19223 8
Donor2-TGAGAGGGTTAAGACA-2 3192 7595 3
Donor2-TGAGCATAGCGATAGC-2 1558 5732 7
Donor2-TGAGCATCAATGGTCT-2 2372 6993 11
Donor2-TGAGCATCACATGGGA-2 2264 10299 7
Donor2-TGAGCATGTTTGGCGC-2 2834 8861 11
Donor2-TGAGCCGTCTCAAACG-2 6270 28980 6
Donor2-TGAGCCGTCTTAGAGC-2 6953 46982 4
Donor2-TGAGGGAAGTGCTGCC-2 3328 13772 6
Donor2-TGAGGGACATCGGAAG-2 7972 66130 6
Donor2-TGAGGGACATGGTCTA-2 5149 16134 6
Donor2-TGAGGGAGTCTTCAAG-2 1447 7966 8
Donor2-TGATTTCAGCTAAACA-2 4626 14872 2
Donor2-TGCCAAACACCGATAT-2 1946 11167 8
Donor2-TGCCAAACACCTCGTT-2 1462 7007 8
Donor2-TGCCAAATCCAGATCA-2 1830 7897 13
Donor2-TGCCCATAGCCGTCGT-2 2431 6726 11
Donor2-TGCCCATAGGATGCGT-2 2517 7337 11
Donor2-TGCCCATCAAGCTGGA-2 2966 16497 7
Donor2-TGCCCATTCAGAGACG-2 3015 22233 7
Donor2-TGCCCTAAGAAGGGTA-2 8596 109574 5
Donor2-TGCCCTAAGCAGGTCA-2 3282 15765 13

Donor2-TGCCCTACATGGGAAC-2 2873 6251 3
Donor2-TGCGCAGAGTTCGCAT-2 3446 21356 7
Donor2-TGCGGGTAGAGTGAGA-2 2521 8184 6
Donor2-TGCGGGTCATCACAAC-2 2625 12150 7
Donor2-TGCGGGTGTAGGCATG-2 3237 18600 7
Donor2-TGCGTGGGTACCGTTA-2 2917 6762 3
Donor2-TGCTACCCACCGAATT-2 5089 17070 4
Donor2-TGCTACCTCACGAAGG-2 3323 28395 7
Donor2-TGCTACCTCAGGTAAA-2 4190 21097 6
Donor2-TGCTACCTCATCTGTT-2 2111 7037 6
Donor2-TGCTGCTAGGTTACCT-2 1637 6197 8
Donor2-TGCTGCTTCTCCCTGA-2 4617 13917 1
Donor2-TGGACGCCAAGTCTAC-2 2715 22030 8
Donor2-TGGACGCCAATGGATA-2 2768 9216 6
Donor2-TGGACGCTCTCGTATT-2 3396 17759 10
Donor2-TGGCGCAAGACGCACA-2 4017 10445 1
Donor2-TGGCGCAGTAACGCGA-2 8142 56852 6
Donor2-TGGCGCAGTCCAACTA-2 2869 8565 11
Donor2-TGGCGCAGTGTGCCTG-2 2852 11742 11
Donor2-TGGCTGGAGAATTCCC-2 7177 53369 6
Donor2-TGGCTGGAGACAGAGA-2 4553 13996 5
Donor2-TGGCTGGCATTTGCTT-2 2468 19786 8
Donor2-TGGGAAGGTCATATGC-2 1703 6258 13
Donor2-TGGGCGTCAAGGACAC-2 2739 9846 13
Donor2-TGGGCGTTCGCCAAAT-2 5628 31238 6
Donor2-TGGTTAGAGATGTAAC-2 3863 29058 7
Donor2-TGGTTCCCAGCGATCC-2 5321 18551 2
Donor2-TGGTTCCTCTTGAGAC-2 3113 7570 8
Donor2-TGGTTCCTCTTGTTTG-2 4292 11644 6
Donor2-TGTATTCTCCTTTCTC-2 3542 12992 5
Donor2-TGTATTCTCGCTGATA-2 6028 24460 6
Donor2-TGTCCCAAGCTAGGCA-2 1751 5834 6
Donor2-TGTCCCAGTGCCTGGT-2 6521 35256 6
Donor2-TGTCCCAGTTAAGTAG-2 2758 8559 10
Donor2-TGTGTTTCACAACGCC-2 6712 43622 4
Donor2-TGTGTTTGTGAGTATA-2 4360 12622 3
Donor2-TGTGTTTGTTGTCTTT-2 3846 11579 2
Donor2-TGTTCCGAGGAATGGA-2 4796 23134 6
Donor2-TGTTCCGTCAGTTCGA-2 5781 31755 5
Donor2-TTAACTCAGCTGAACG-2 4259 17458 5
Donor2-TTAACTCAGTAAGTAC-2 3128 8373 1
Donor2-TTAACTCGTAGAGGAA-2 4035 19743 6
Donor2-TTAACTCGTGTAAGTA-2 7544 41251 5
Donor2-TTAGTTCAGCTATGCT-2 5491 23573 6
Donor2-TTAGTTCAGGAATGGA-2 6350 28924 6
Donor2-TTAGTTCCACATCCGG-2 1906 7708 7
Donor2-TTAGTTCTCGAATGGG-2 2262 15621 8
Donor2-TTATGCTAGTCCATAC-2 2813 16629 7
Donor2-TTCCCAGCAGCTCCGA-2 6105 33563 10
Donor2-TTCCCAGCATCGACGC-2 3658 23347 7
Donor2-TTCCCAGTCATGGTCA-2 2839 8732 11
Donor2-TTCGAAGCATGCAATC-2 8381 54217 5
Donor2-TTCGAAGTCTAACGGT-2 2941 10209 11
Donor2-TTCGGTCAGGATGTAT-2 8127 44533 5
Donor2-TTCTACAGTCGCATCG-2 2199 10755 7
Donor2-TTCTCCTAGCGATATA-2 1223 5857 8

Donor2-TTCTCCTAGTGTCCAT-2 2563 11955 7
Donor2-TTCTCCTCACGAGAGT-2 6426 33449 4
Donor2-TTCTCCTGTCAAACTC-2 3203 18756 6
Donor2-TTCTCCTGTGTGCCTG-2 3822 17672 6
Donor2-TTCTTAGAGTATGACA-2 1635 6229 7
Donor2-TTCTTAGGTCAGCTAT-2 3844 9988 1
Donor2-TTCTTAGTCATAACCG-2 4098 12937 6
Donor2-TTCTTAGTCCGTACAA-2 2181 15220 8
Donor2-TTCTTAGTCTCGGACG-2 3088 7318 1
Donor2-TTGAACGAGTAGCGGT-2 6904 50809 5
Donor2-TTGACTTCACCGAAAG-2 3375 16789 10
Donor2-TTGACTTGTATGAATG-2 3963 9845 3
Donor2-TTGACTTGTCGGCTCA-2 6285 25851 5
Donor2-TTGACTTTCGTTTAGG-2 3932 10357 5
Donor2-TTGCCGTCATGCGCAC-2 1573 8437 8
Donor2-TTGCCGTTCACTTATC-2 2350 11450 7
Donor2-TTGCCGTTCGGCTACG-2 6274 27121 6
Donor2-TTGCGTCGTTAAGATG-2 3868 18532 6
Donor2-TTGGAACAGTTAGCGG-2 4468 10710 1
Donor2-TTGGAACGTTGCGTTA-2 2777 10836 13
Donor2-TTGGCAAAGCTCAACT-2 2632 13904 7
Donor2-TTGGCAACACTGTCGG-2 1713 11108 8
Donor2-TTGGCAAGTCCGTCAG-2 2361 10646 9
Donor2-TTGTAGGGTCTTGTCC-2 2601 8651 11
Donor2-TTTACTGAGCTGCCCA-2 4008 22955 6
Donor2-TTTACTGCAGTATAAG-2 1394 7055 8
Donor2-TTTATGCAGTGAAGAG-2 2412 12906 7
Donor2-TTTATGCCACAGCCCA-2 3482 14742 6
Donor2-TTTATGCGTAGCCTAT-2 3453 11818 3
Donor2-TTTCCTCCATCGACGC-2 2124 9327 7
Donor2-TTTCCTCGTACTCGCG-2 5825 26662 4
Donor2-TTTGCGCAGATACACA-2 2265 8588 11
Donor2-TTTGCGCAGGCAGGTT-2 2922 12100 7
Donor2-TTTGCGCAGGTGACCA-2 4144 16641 4
Donor2-TTTGCGCCACGACTCG-2 2045 11274 8
Donor2-TTTGTCAAGAGCTGGT-2 1524 5830 10
Donor3-AAACGGGGTAGTAGTA-1 3118 7062 3
Donor3-AAACGGGGTCTAAAGA-1 2056 8950 7
Donor3-AAACGGGGTGTAACGG-1 2224 12688 7
Donor3-AAACGGGTCTCACATT-1 1161 6779 8
Donor3-AAAGATGCAGCTGTTA-1 1331 8332 8
Donor3-AAAGATGGTCAGATAA-1 6068 24303 5
Donor3-AAAGCAATCACGACTA-1 2204 10997 7
Donor3-AAAGCAATCTCGGACG-1 1667 6021 9
Donor3-AAAGTAGCACCAGGTC-1 2532 12971 7
Donor3-AAAGTAGGTGGCGAAT-1 3062 13437 6
Donor3-AAAGTAGTCACAAACC-1 6785 33955 2
Donor3-AAAGTAGTCGGCGGTT-1 6058 35119 4
Donor3-AAAGTAGTCTGGGCCA-1 1711 10853 8
Donor3-AAATGCCCAAGTCTAC-1 3342 7590 3
Donor3-AAATGCCGTTCGTCTC-1 4191 10669 6
Donor3-AAATGCCTCACTTATC-1 1466 6026 7
Donor3-AACACGTCAAGTTAAG-1 3307 14951 6
Donor3-AACACGTCAGGTGCCT-1 6536 23774 1
Donor3-AACACGTGTTCTCATT-1 6728 44964 4
Donor3-AACACGTGTTGCCTCT-1 1611 7764 8

Donor3-AACCATGAGGTGCTTT-1 3865 10091 3
Donor3-AACCATGCATCAGTAC-1 1754 5097 13
Donor3-AACCATGGTATCAGTC-1 6048 28383 6
Donor3-AACCATGGTTAAGACA-1 3167 6898 3
Donor3-AACCGCGCACAGGAGT-1 2468 15212 7
Donor3-AACCGCGCACGCTTTC-1 1506 4658 9
Donor3-AACCGCGTCCTTTCTC-1 1685 8146 7
Donor3-AACGTTGAGCCCGAAA-1 1254 4932 8
Donor3-AACGTTGCAGAGTGTG-1 1768 11472 8
Donor3-AACGTTGGTGATAAGT-1 2723 6097 6
Donor3-AACGTTGTCCGATATG-1 2133 5332 4
Donor3-AACGTTGTCTCCAGGG-1 3542 8595 3
Donor3-AACTCAGCACATGTGT-1 3355 21106 7
Donor3-AACTCAGGTAGCAAAT-1 3975 17565 6
Donor3-AACTCAGGTGTAAGTA-1 3301 7255 3
Donor3-AACTCCCGTCATACTG-1 2034 10209 7
Donor3-AACTCCCTCCTGTAGA-1 1402 8639 8
Donor3-AACTCTTTCAGAGGTG-1 6314 23132 2
Donor3-AACTGGTGTCCGTGAC-1 1548 4413 10
Donor3-AACTGGTTCCACTCCA-1 3722 8829 3
Donor3-AACTTTCAGCTGAACG-1 4364 11725 1
Donor3-AACTTTCGTAGTAGTA-1 2041 9538 7
Donor3-AACTTTCTCCCGGATG-1 2529 4856 2
Donor3-AAGACCTCATTAACCG-1 2199 15533 8
Donor3-AAGACCTTCAGGATCT-1 1819 13552 8
Donor3-AAGACCTTCCACGTTC-1 5321 16617 6
Donor3-AAGCCGCAGACTTTCG-1 1262 7175 8
Donor3-AAGCCGCGTCGATTGT-1 4571 13023 5
Donor3-AAGGAGCCAAGTCTAC-1 2225 6698 13
Donor3-AAGGAGCGTCTTTCAT-1 2014 5799 11
Donor3-AAGGAGCTCTTCAACT-1 1347 8050 8
Donor3-AAGGCAGAGCTGTTCA-1 1039 5058 8
Donor3-AAGGCAGAGTCTCAAC-1 4550 12703 1
Donor3-AAGGCAGGTGTTTGGT-1 1346 6363 8
Donor3-AAGGCAGGTTGGAGGT-1 1599 5705 8
Donor3-AAGGCAGTCCTATGTT-1 2569 8614 6
Donor3-AAGGTTCCATTGTGCA-1 3848 9971 2
Donor3-AAGTCTGAGCAGGCTA-1 2481 5396 3
Donor3-AAGTCTGAGGCATGGT-1 2861 5491 6
Donor3-AAGTCTGCACAGGAGT-1 1487 4776 13
Donor3-AAGTCTGCATTCTCAT-1 3147 16368 7
Donor3-AAGTCTGGTACCCAAT-1 2777 15934 7
Donor3-AAGTCTGGTTTACTCT-1 3290 20418 7
Donor3-AAGTCTGTCATGTCTT-1 1985 7339 9
Donor3-AAGTCTGTCTGTTTGT-1 3764 9375 3
Donor3-AATCCAGGTCTGCAAT-1 1454 4991 9
Donor3-AATCCAGTCTGATACG-1 1735 4494 13
Donor3-ACACCGGGTCAGATAA-1 1837 6336 9
Donor3-ACAGCCGAGCGAAGGG-1 1866 12021 8
Donor3-ACAGCCGCAAGACACG-1 5177 18023 2
Donor3-ACAGCCGTCAACCAAC-1 1355 5035 7
Donor3-ACAGCTAAGCTCAACT-1 5418 19891 5
Donor3-ACATACGAGATGCCTT-1 1925 10213 7
Donor3-ACATACGGTAGCGTCC-1 5679 27949 5
Donor3-ACATACGGTCCGCTGA-1 1587 9890 8
Donor3-ACATACGGTGTGGTTT-1 3392 8276 3

Donor3-ACATACGTCGAACGGA-1 4545 52768 7
Donor3-ACATCAGAGCGTGAAC-1 2667 5723 6
Donor3-ACATCAGAGCTGAACG-1 2277 7359 13
Donor3-ACATCAGGTTCCCGAG-1 2761 12265 7
Donor3-ACATCAGTCCGCTGTT-1 1094 4846 8
Donor3-ACATGGTAGTCGATAA-1 6216 31037 4
Donor3-ACATGGTGTCTCCACT-1 1977 5815 6
Donor3-ACATGGTTCAGGATCT-1 1055 4352 8
Donor3-ACCAGTAAGAAGGTTT-1 2409 10895 7
Donor3-ACCAGTATCACTTATC-1 4947 19182 6
Donor3-ACCAGTATCTCTTGAT-1 1723 4628 13
Donor3-ACCCACTAGTTACGGG-1 6625 26117 2
Donor3-ACCCACTGTCAGAAGC-1 5069 22550 6
Donor3-ACCCACTTCCGTTGCT-1 1072 5507 8
Donor3-ACCGTAAGTGTGTGCC-1 1591 4963 13
Donor3-ACGAGCCCAATAACGA-1 2885 6300 3
Donor3-ACGAGCCCACACCGAC-1 2323 7657 13
Donor3-ACGAGCCCACCTTGTC-1 952 5077 8
Donor3-ACGAGGACACCAGGCT-1 6835 51730 5
Donor3-ACGAGGACATCACGAT-1 3071 12381 7
Donor3-ACGAGGATCAACACTG-1 1737 6107 9
Donor3-ACGAGGATCAGCGACC-1 1609 5226 13
Donor3-ACGATACAGGAGTTGC-1 2327 12350 7
Donor3-ACGATACAGGCTATCT-1 1327 4678 9
Donor3-ACGATACTCCCTGACT-1 6337 25198 1
Donor3-ACGATACTCTACCAGA-1 2862 17074 7
Donor3-ACGATGTAGACAGGCT-1 1668 8174 7
Donor3-ACGATGTAGGATCGCA-1 2199 7051 11
Donor3-ACGATGTCAGATCCAT-1 3537 8716 3
Donor3-ACGATGTGTTGTCTTT-1 1636 4379 13
Donor3-ACGCAGCAGCCAGTTT-1 4024 20749 6
Donor3-ACGCAGCAGGACTGGT-1 2094 9780 7
Donor3-ACGCAGCAGTGGACGT-1 1438 9577 8
Donor3-ACGCAGCCAATGGATA-1 4233 21653 6
Donor3-ACGCAGCTCTTACCTA-1 3614 8848 3
Donor3-ACGCCAGAGTCCGGTC-1 4056 10891 5
Donor3-ACGCCAGCAAGCCTAT-1 1073 4919 8
Donor3-ACGGAGAAGACTACAA-1 1031 4449 8
Donor3-ACGGAGAAGCTGATAA-1 1508 7044 8
Donor3-ACGGAGAAGGAGCGTT-1 2374 9710 7
Donor3-ACGGAGAAGGCTACGA-1 1643 7850 7
Donor3-ACGGAGAAGGGTATCG-1 4859 19190 6
Donor3-ACGGAGACACGCATCG-1 2560 6667 10
Donor3-ACGGAGATCCGCATAA-1 1538 8804 8
Donor3-ACGGCCAAGACTGTAA-1 4796 13380 2
Donor3-ACGGCCAAGCATCATC-1 3488 8556 4
Donor3-ACGGCCAAGGGCACTA-1 1634 10740 8
Donor3-ACGGCCACAGCTATTG-1 1495 4489 13
Donor3-ACGGCCAGTCCGAACC-1 1551 4953 9
Donor3-ACGGCCAGTGAACCTT-1 2095 7219 10
Donor3-ACGGCCATCTGTCTCG-1 2178 9615 9
Donor3-ACGGGCTTCAGTCAGT-1 1189 7911 8
Donor3-ACGGGCTTCCGTTGTC-1 3770 14054 6
Donor3-ACGGGTCCAAGTAATG-1 4537 13562 2
Donor3-ACGGGTCGTGTTAAGA-1 1333 5741 8
Donor3-ACGTCAAAGTACGCGA-1 1169 6213 8

Donor3-ACGTCAATCTCGGACG-1 5565 23414 6
Donor3-ACTATCTAGCACCGTC-1 3951 10843 3
Donor3-ACTATCTGTACATCCA-1 1821 6906 7
Donor3-ACTGAACAGAATCTCC-1 1685 5934 7
Donor3-ACTGAACAGTGTCCAT-1 2734 8941 4
Donor3-ACTGAACCAGCGTAAG-1 1466 5125 7
Donor3-ACTGAACGTCTCTCTG-1 6237 21113 3
Donor3-ACTGATGCAAGGCTCC-1 1488 5127 9
Donor3-ACTGTCCAGGGTATCG-1 1573 9007 8
Donor3-ACTTACTAGTGGGATC-1 5798 21388 5
Donor3-ACTTACTCACGCGAAA-1 2662 8592 10
Donor3-ACTTACTCACTTACGA-1 2083 7349 10
Donor3-ACTTACTGTCCCTTGT-1 2588 8017 10
Donor3-ACTTGTTTCGCCATAA-1 1741 7336 8
Donor3-ACTTTCACAGCTGGCT-1 1329 4304 9
Donor3-ACTTTCATCCAAGCCG-1 2099 6803 11
Donor3-AGACGTTCATGGGACA-1 2516 8025 10
Donor3-AGACGTTGTCTCGTTC-1 2559 5128 6
Donor3-AGAGCGAGTTTAAGCC-1 2581 12215 7
Donor3-AGAGCTTAGAGGGCTT-1 2260 6335 6
Donor3-AGAGCTTAGATATGCA-1 3149 10042 11
Donor3-AGAGTGGAGCGATCCC-1 3625 8809 3
Donor3-AGAGTGGTCAGAAATG-1 2332 10926 7
Donor3-AGAGTGGTCGAATCCA-1 4175 17003 6
Donor3-AGAGTGGTCGAATGGG-1 1368 7167 8
Donor3-AGATTGCAGTTAAGTG-1 1757 10359 8
Donor3-AGCAGCCAGAGACTTA-1 4247 16293 2
Donor3-AGCAGCCGTCCAACTA-1 6255 25698 5
Donor3-AGCATACAGTTATCGC-1 1700 4454 13
Donor3-AGCCTAAAGATGTGGC-1 2389 16302 7
Donor3-AGCCTAAAGGTAAACT-1 4431 22983 6
Donor3-AGCCTAACAATCCGAT-1 1165 4679 8
Donor3-AGCGGTCAGAATAGGG-1 2982 14696 7
Donor3-AGCGGTCGTTACGCGC-1 2455 5203 5
Donor3-AGCGGTCTCGTGTAGT-1 1672 4869 8
Donor3-AGCGTATCACCGTTGG-1 5662 19583 5
Donor3-AGCGTATTCTGCTGCT-1 6652 30492 6
Donor3-AGCGTCGAGCTAGCCC-1 3809 8881 3
Donor3-AGCGTCGCAATGGACG-1 1587 4600 13
Donor3-AGCTCCTAGTTAAGTG-1 1931 7907 9
Donor3-AGCTCCTCACAGTCGC-1 5885 24646 6
Donor3-AGCTCCTCACGGTTTA-1 1356 8015 8
Donor3-AGCTCCTCACTACAGT-1 2798 23023 8
Donor3-AGCTCCTGTTACCAGT-1 1374 5891 8
Donor3-AGCTCCTGTTCTGTTT-1 1519 8918 8
Donor3-AGCTCCTTCGAGAACG-1 2195 7945 10
Donor3-AGCTCTCAGCAGCGTA-1 4397 24017 4
Donor3-AGCTCTCTCGAATCCA-1 2259 6742 13
Donor3-AGCTCTCTCTGACCTC-1 1509 8586 8
Donor3-AGCTTGAAGGATGGAA-1 4460 14101 2
Donor3-AGGCCACTCCGAGCCA-1 1964 7728 9
Donor3-AGGCCACTCCTTAATC-1 5134 26951 6
Donor3-AGGCCGTCATGGGACA-1 1531 4344 13
Donor3-AGGGAGTGTACAGTTC-1 1241 6078 8
Donor3-AGGGAGTGTCTGCGGT-1 2832 11048 10
Donor3-AGGGATGGTAATTGGA-1 1922 5091 5

Donor3-AGGGATGTCAGAGCTT-1 1656 5452 9
Donor3-AGGGATGTCCTAGTGA-1 2584 7552 4
Donor3-AGGGTGAAGCTCCTTC-1 1692 7987 7
Donor3-AGGGTGACAATCAGAA-1 1611 5777 8
Donor3-AGGGTGACACTTAACG-1 5136 16592 1
Donor3-AGGTCATGTGAGCGAT-1 3748 9568 3
Donor3-AGGTCATTCGCGGATC-1 915 5197 7
Donor3-AGGTCCGAGGCCATAG-1 1513 5103 9
Donor3-AGGTCCGCAGTCAGCC-1 3349 7491 3
Donor3-AGGTCCGGTAGGGACT-1 2280 12180 7
Donor3-AGTAGTCCATTGAGCT-1 5162 16385 1
Donor3-AGTCTTTAGGACGAAA-1 3752 23154 7
Donor3-AGTCTTTGTCCTCTTG-1 1134 6382 8
Donor3-AGTCTTTGTCGGATCC-1 1711 11395 8
Donor3-AGTCTTTTCTGCAAGT-1 5469 18446 4
Donor3-AGTGAGGAGGGCACTA-1 3186 11825 11
Donor3-AGTGGGACACGGCTAC-1 5504 26806 6
Donor3-AGTGGGAGTGGGTCAA-1 2216 16449 8
Donor3-AGTGGGAGTTGCCTCT-1 1773 9055 7
Donor3-AGTGTCACAGGTCCAC-1 1374 7891 8
Donor3-AGTTGGTCACTATCTT-1 3682 17953 6
Donor3-AGTTGGTGTGGTACAG-1 1384 5400 8
Donor3-ATAACGCTCGGAAATA-1 1636 11003 8
Donor3-ATAAGAGCACTGTGTA-1 1281 4736 9
Donor3-ATAAGAGTCCAAATGC-1 1317 5761 8
Donor3-ATAGACCAGCCATCGC-1 3580 8655 4
Donor3-ATAGACCGTATTCTCT-1 1900 7389 9
Donor3-ATCACGAAGTTAGCGG-1 4029 31847 7
Donor3-ATCACGAGTCCTAGCG-1 1343 5409 7
Donor3-ATCATCTAGGGTTCCC-1 1707 10904 8
Donor3-ATCATCTGTCTAAAGA-1 2055 4796 3
Donor3-ATCATGGAGGTAGCTG-1 3853 18887 6
Donor3-ATCCACCAGAAACCGC-1 3026 6545 3
Donor3-ATCCACCGTAATCGTC-1 1641 4499 13
Donor3-ATCCACCGTTGGAGGT-1 2083 11802 7
Donor3-ATCCGAAAGGACCACA-1 1725 9239 8
Donor3-ATCCGAACAAATTGCC-1 1080 4346 8
Donor3-ATCCGAAGTTGCGCAC-1 2469 13811 7
Donor3-ATCGAGTTCTCCTATA-1 1837 5816 13
Donor3-ATCTACTGTGGCGAAT-1 4157 11252 3
Donor3-ATCTACTTCTTACCTA-1 3145 7246 5
Donor3-ATCTGCCAGAAACCGC-1 4168 10732 1
Donor3-ATCTGCCCACAGACAG-1 1182 4776 8
Donor3-ATGAGGGAGAGCTGGT-1 1214 6233 8
Donor3-ATGAGGGAGGCATGTG-1 1297 7246 8
Donor3-ATGAGGGGTTTAGCTG-1 1663 6388 7
Donor3-ATGAGGGTCATGCAAC-1 2806 12446 7
Donor3-ATGCGATAGCGCTTAT-1 1501 7338 8
Donor3-ATGCGATCATAGTAAG-1 1425 7612 8
Donor3-ATGCGATGTCTAGAGG-1 6624 34587 6
Donor3-ATGGGAGAGAAACCAT-1 4383 12954 4
Donor3-ATGGGAGCAGATGGGT-1 1571 5813 7
Donor3-ATGGGAGGTCTAGTGT-1 1547 7182 7
Donor3-ATGGGAGGTTACCAGT-1 1112 6443 8
Donor3-ATGGGAGTCACGCGGT-1 2091 6336 13
Donor3-ATGTGTGAGAATGTTG-1 1885 13050 8

Donor3-ATGTGTGAGGCTAGAC-1 1253 5278 7
Donor3-ATGTGTGAGTTGTAGA-1 2510 9107 9
Donor3-ATGTGTGTCGGCGGTT-1 1644 4400 13
Donor3-ATTACTCAGAATTGTG-1 1975 6535 13
Donor3-ATTACTCAGCGCCTCA-1 2057 5646 10
Donor3-ATTACTCAGGGTTTCT-1 3539 7813 2
Donor3-ATTATCCCAGGCAGTA-1 1900 13407 8
Donor3-ATTATCCGTGAGGCTA-1 1476 5334 7
Donor3-ATTATCCGTGGAAAGA-1 2223 8705 9
Donor3-ATTATCCGTTAAGACA-1 3171 7413 5
Donor3-ATTCTACCACATTCGA-1 8745 77185 6
Donor3-ATTCTACCATGCTGGC-1 1416 4897 7
Donor3-ATTGGACCAAACTGTC-1 4329 14256 5
Donor3-ATTGGACCATACAGCT-1 4215 14585 6
Donor3-ATTGGTGCATCACGTA-1 2120 9228 7
Donor3-ATTGGTGTCGTAGGAG-1 1722 5892 9
Donor3-ATTTCTGGTTAGAACA-1 1560 4287 6
Donor3-CAACCTCAGCTAGTCT-1 4467 19549 10
Donor3-CAACCTCCACTGTTAG-1 4179 26950 7
Donor3-CAACTAGAGTCCAGGA-1 1546 5296 13
Donor3-CAACTAGCACAACGCC-1 2623 8496 13
Donor3-CAACTAGGTTATCACG-1 3489 16881 6
Donor3-CAACTAGGTTATCGGT-1 5670 18346 1
Donor3-CAAGATCAGGCCCGTT-1 1593 7304 8
Donor3-CAAGATCCAAACTGCT-1 1797 9927 8
Donor3-CAAGATCCAATGACCT-1 4828 14631 1
Donor3-CAAGGCCAGAAACCAT-1 1903 4763 13
Donor3-CACAAACCACTACAGT-1 1435 6103 13
Donor3-CACACCTCAAGCTGAG-1 2881 13479 7
Donor3-CACACCTGTATAATGG-1 2046 10985 7
Donor3-CACACTCAGTCATGCT-1 2337 9551 9
Donor3-CACACTCCAACTTGAC-1 3804 14336 10
Donor3-CACACTCCACAACGTT-1 1309 7019 8
Donor3-CACACTCTCGTATCAG-1 3301 12595 10
Donor3-CACAGGCAGAGTACCG-1 4857 25228 5
Donor3-CACAGGCGTACTTCTT-1 6531 27004 5
Donor3-CACAGGCGTAGCACGA-1 1316 5918 8
Donor3-CACAGTACACTATCTT-1 1983 4523 4
Donor3-CACAGTATCCGAACGC-1 2330 6653 10
Donor3-CACAGTATCGCATGGC-1 3290 15912 7
Donor3-CACATAGCACGAAAGC-1 4147 22338 6
Donor3-CACATAGGTAGAGGAA-1 4768 15417 6
Donor3-CACATAGTCACTATTC-1 6558 29683 6
Donor3-CACATTTCACATGTGT-1 2655 5639 5
Donor3-CACATTTGTTGGGACA-1 6323 33770 4
Donor3-CACCACTCAGGGTACA-1 4906 12159 3
Donor3-CACCACTGTTAAGACA-1 5064 22192 6
Donor3-CACTCCAGTCTAACGT-1 1729 4425 13
Donor3-CAGAATCCAACTGCGC-1 2486 6544 4
Donor3-CAGAATCCACGCATCG-1 4869 14740 5
Donor3-CAGAATCTCACCACCT-1 947 4410 8
Donor3-CAGAGAGAGCCACGCT-1 1774 12316 8
Donor3-CAGAGAGTCCGTCATC-1 2773 12637 7
Donor3-CAGATCAAGTGGGTTG-1 2330 10716 7
Donor3-CAGATCAGTCTAGGTT-1 5008 15268 5
Donor3-CAGATCAGTCTCTTAT-1 2407 19043 8

Donor3-CAGATCAGTTCTGGTA-1 1704 6393 7
Donor3-CAGCAGCAGAGGTTGC-1 6205 21521 5
Donor3-CAGCAGCAGAGTAAGG-1 3028 14012 7
Donor3-CAGCAGCCAACTGCTA-1 1573 5616 9
Donor3-CAGCAGCCATAGAAAC-1 1238 4370 8
Donor3-CAGCAGCTCTAGAGTC-1 2039 8035 9
Donor3-CAGCATACACCACGTG-1 2722 13886 7
Donor3-CAGCATAGTTAAGACA-1 1533 9138 8
Donor3-CAGCCGACAAGCCGTC-1 1066 5766 8
Donor3-CAGCCGAGTCTAAAGA-1 1425 7488 8
Donor3-CAGCGACAGTCATGCT-1 5357 30008 6
Donor3-CAGCTAAAGTGGACGT-1 5604 28748 5
Donor3-CAGCTAACAGTATGCT-1 1389 4506 9
Donor3-CAGCTAATCCGCGCAA-1 7199 34905 5
Donor3-CAGCTGGAGGCAAAGA-1 1675 7677 7
Donor3-CAGCTGGCAGACGTAG-1 1463 5275 9
Donor3-CAGCTGGGTACGCACC-1 3817 9774 1
Donor3-CAGGTGCCACAGCGTC-1 2724 5772 3
Donor3-CAGTAACAGCACACAG-1 2446 7999 10
Donor3-CAGTAACAGCTAACAA-1 2281 15412 8
Donor3-CAGTAACAGTGTACTC-1 6906 33020 1
Donor3-CAGTAACCACTACAGT-1 1764 9686 8
Donor3-CATATGGAGATGCGAC-1 5582 20804 4
Donor3-CATCAAGCACATTAGC-1 3402 8314 5
Donor3-CATCAAGGTGATAAGT-1 3163 10437 4
Donor3-CATCAAGTCTTAACCT-1 4118 20087 6
Donor3-CATCAGACAGCTGTTA-1 1142 4605 8
Donor3-CATCAGAGTGTCCTCT-1 1365 8350 8
Donor3-CATCCACAGCCAGTAG-1 6033 31776 4
Donor3-CATCCACTCTCGATGA-1 7051 50323 6
Donor3-CATCGGGCATGATCCA-1 1802 7642 7
Donor3-CATCGGGGTGCACCAC-1 1537 8602 8
Donor3-CATCGGGGTGTCGCTG-1 4549 13173 5
Donor3-CATGCCTCAGACACTT-1 8488 46557 2
Donor3-CATGGCGAGCGTAATA-1 4102 11109 1
Donor3-CATGGCGCACGTAAGG-1 1183 4441 8
Donor3-CATGGCGGTATTCGTG-1 2814 10501 6
Donor3-CATGGCGGTCTAGCCG-1 2748 5928 4
Donor3-CATGGCGTCCCTCTTT-1 1703 6209 13
Donor3-CATTATCAGGATATAC-1 1631 9274 8
Donor3-CATTATCCAAAGCAAT-1 3628 16861 6
Donor3-CATTCGCAGTTCGCAT-1 5643 16785 5
Donor3-CATTCGCCAGATAATG-1 1615 6645 7
Donor3-CCAATCCAGAATCTCC-1 6120 28888 5
Donor3-CCAATCCCAAGTCTAC-1 1308 6755 8
Donor3-CCAATCCCAATAGCAA-1 3339 7758 3
Donor3-CCAATCCTCCAGATCA-1 1408 7083 8
Donor3-CCACCTAAGATAGTCA-1 1495 4322 13
Donor3-CCACCTAAGCTATGCT-1 1448 8542 8
Donor3-CCACCTACACTGTTAG-1 4324 10868 2
Donor3-CCACCTAGTCTAGGTT-1 1578 5744 7
Donor3-CCACCTATCTCTTATG-1 1691 5555 9
Donor3-CCACGGAAGCGCCTCA-1 1676 9264 8
Donor3-CCACGGATCCAATGGT-1 4770 15198 1
Donor3-CCAGCGAAGAATAGGG-1 1057 4576 8
Donor3-CCAGCGAAGGGAAACA-1 2234 10919 7

Donor3-CCATGTCAGTTTGCGT-1 3106 13038 6
Donor3-CCATTCGCAAGAAGAG-1 1847 4704 13
Donor3-CCATTCGGTATAGGTA-1 5559 26679 5
Donor3-CCCAATCAGACGACGT-1 3002 17054 7
Donor3-CCCAATCAGCCACGCT-1 1841 6213 13
Donor3-CCCAATCTCCAGAGGA-1 8733 59775 5
Donor3-CCCAGTTAGCGATAGC-1 4489 13462 2
Donor3-CCCAGTTAGGGAAACA-1 2596 9688 10
Donor3-CCCAGTTCACCGAAAG-1 2757 8419 10
Donor3-CCCAGTTTCAACACCA-1 4647 12203 4
Donor3-CCCATACGTCGCGGTT-1 5718 30378 5
Donor3-CCCATACTCTTGGGTA-1 3729 18617 6
Donor3-CCCTCCTCATTAGCCA-1 5188 19023 5
Donor3-CCCTCCTTCTTGACGA-1 1544 4649 13
Donor3-CCGTGGAAGACACTAA-1 1668 8096 8
Donor3-CCGTGGAAGATGCGAC-1 1789 6040 6
Donor3-CCGTGGAAGGACATTA-1 2760 11196 6
Donor3-CCGTGGACATGTAGTC-1 7675 43791 5
Donor3-CCGTGGAGTAACGACG-1 1700 5388 13
Donor3-CCGTGGAGTTAAAGTG-1 1449 5202 7
Donor3-CCGTGGAGTTGATTGC-1 5952 33415 6
Donor3-CCGTTCAAGGCTCTTA-1 4825 28951 6
Donor3-CCGTTCAGTTCGGCAC-1 1292 6105 8
Donor3-CCTAAAGAGCTAGGCA-1 1919 11168 7
Donor3-CCTAAAGAGTCCCACG-1 5801 28616 5
Donor3-CCTAAAGTCAGCCTAA-1 1725 12556 8
Donor3-CCTACACAGTCCGTAT-1 3405 7986 3
Donor3-CCTACACGTCATATGC-1 1285 4463 13
Donor3-CCTACACGTTCGCTAA-1 1413 4461 8
Donor3-CCTACCAAGGTTCCTA-1 2607 5993 2
Donor3-CCTAGCTGTCATGCCG-1 4594 26325 13
Donor3-CCTAGCTGTCTCATCC-1 2476 10533 9
Donor3-CCTAGCTGTGATGCCC-1 1283 6390 8
Donor3-CCTCAGTGTACTCGCG-1 1767 5674 13
Donor3-CCTCAGTGTGCCTGCA-1 2908 8979 10
Donor3-CCTCAGTTCCCTGACT-1 1193 5828 8
Donor3-CCTCTGAAGATGAGAG-1 2942 5979 3
Donor3-CCTCTGAAGGTCATCT-1 2643 5789 2
Donor3-CCTCTGAAGTTCGCAT-1 2367 5010 3
Donor3-CCTTACGAGAGGTACC-1 2106 6953 6
Donor3-CCTTACGAGTGGTCCC-1 1473 4707 9
Donor3-CCTTACGGTCTCTTAT-1 4201 13678 5
Donor3-CCTTCGAGTAATCACC-1 1671 5415 6
Donor3-CCTTCGAGTCCAACTA-1 2547 8627 13
Donor3-CCTTCGATCACTCCTG-1 1623 6912 7
Donor3-CCTTCGATCGAGAACG-1 1111 4703 8
Donor3-CCTTTCTCACTTACGA-1 1479 6849 7
Donor3-CCTTTCTGTCGGGTCT-1 1534 5667 9
Donor3-CCTTTCTGTTGTGGAG-1 3951 9717 3
Donor3-CCTTTCTTCACGAAGG-1 1443 9617 8
Donor3-CCTTTCTTCCTTCAAT-1 1219 5180 8
Donor3-CCTTTCTTCGAATGCT-1 3923 13807 6
Donor3-CGAACATAGTCAATAG-1 1787 9710 7
Donor3-CGAACATTCCGCATCT-1 1048 4499 8
Donor3-CGAATGTAGACGCAAC-1 4446 15637 2
Donor3-CGAATGTAGAGACTTA-1 1082 4464 7

Donor3-CGAATGTAGTTGTCGT-1 6207 24534 1
Donor3-CGAATGTTCAGGCGAA-1 2157 8603 7
Donor3-CGAATGTTCCACGACG-1 4825 14138 4
Donor3-CGAATGTTCCGTCATC-1 3878 20524 7
Donor3-CGACCTTAGAGCCCAA-1 8054 46803 5
Donor3-CGACCTTGTATTAGCC-1 2498 13607 7
Donor3-CGACCTTTCCAGAAGG-1 1358 7532 8
Donor3-CGACTTCAGGATATAC-1 5862 33096 4
Donor3-CGACTTCAGTGGACGT-1 1024 4977 8
Donor3-CGACTTCCACTTACGA-1 1311 8401 8
Donor3-CGACTTCCAGTACACT-1 3807 10488 5
Donor3-CGAGAAGAGATCCCGC-1 2205 4539 13
Donor3-CGAGAAGAGCGATTCT-1 2345 14114 7
Donor3-CGAGAAGGTAGAGTGC-1 2695 5561 3
Donor3-CGAGCACCAATGTTGC-1 1552 6952 7
Donor3-CGATCGGGTAGTACCT-1 1252 5121 7
Donor3-CGATCGGTCCCGACTT-1 1676 7568 7
Donor3-CGATCGGTCGCTGATA-1 7090 42539 6
Donor3-CGATCGGTCGTATCAG-1 3455 8831 3
Donor3-CGATGGCGTCTAAACC-1 2478 18139 8
Donor3-CGATGTACATCAGTCA-1 1666 7532 7
Donor3-CGATTGAAGCGTCAAG-1 2078 6815 9
Donor3-CGATTGAGTGTTGAGG-1 6067 24460 5
Donor3-CGCCAAGAGCCAGAAC-1 2330 6830 10
Donor3-CGCCAAGCATGTCTCC-1 1590 5283 9
Donor3-CGCCAAGTCCAGTAGT-1 6217 50635 6
Donor3-CGCGGTACAGCCAATT-1 2137 6319 13
Donor3-CGCGGTATCCGCTGTT-1 4873 15231 4
Donor3-CGCGTTTAGTAGTGCG-1 3976 10717 5
Donor3-CGCGTTTCAAGCTGTT-1 2152 11081 7
Donor3-CGCGTTTCATTTGCTT-1 3280 7615 3
Donor3-CGCTATCAGCGTTTAC-1 1613 6291 7
Donor3-CGCTGGAAGATCTGCT-1 4073 28724 7
Donor3-CGCTGGAGTCTAGCCG-1 2068 5931 13
Donor3-CGCTGGAGTGTTGAGG-1 1475 5144 6
Donor3-CGCTTCAGTAAACACA-1 1931 8577 7
Donor3-CGCTTCATCTAACCGA-1 3291 7126 3
Donor3-CGGACACTCAGGATCT-1 4208 10785 3
Donor3-CGGACGTAGGCAGTCA-1 2356 9958 9
Donor3-CGGACGTCAAGTTAAG-1 1324 6313 8
Donor3-CGGACGTTCGGATGGA-1 1649 4882 13
Donor3-CGGACTGAGTGCGTGA-1 1529 8894 8
Donor3-CGGACTGCAGAGTGTG-1 2935 19926 7
Donor3-CGGACTGTCTCGCATC-1 2334 6667 11
Donor3-CGGAGCTGTTAAGATG-1 3450 8453 3
Donor3-CGGAGCTGTTGGGACA-1 2330 16516 7
Donor3-CGGAGTCAGCTTCGCG-1 2885 6809 6
Donor3-CGGAGTCAGGTTACCT-1 1089 6286 8
Donor3-CGGCTAGAGCCACCTG-1 1969 11266 7
Donor3-CGGCTAGTCGGTGTCG-1 3281 7333 3
Donor3-CGGGTCAAGAAGGCCT-1 1706 6594 9
Donor3-CGGGTCATCACCAGGC-1 2576 5549 5
Donor3-CGGGTCATCCATGCTC-1 2901 9271 5
Donor3-CGGGTCATCCCGACTT-1 1257 4944 8
Donor3-CGGTTAAGTGCTAGCC-1 1957 17520 8
Donor3-CGGTTAATCCAAAGTC-1 3747 18985 6

Donor3-CGGTTAATCTGGCGAC-1 3026 6556 5
Donor3-CGTCACTAGGGATACC-1 1779 7063 9
Donor3-CGTCACTCACTATCTT-1 3793 9930 5
Donor3-CGTCACTCACTCGACG-1 2249 15724 7
Donor3-CGTCAGGAGCACAGGT-1 4031 10535 5
Donor3-CGTCAGGCAGTATGCT-1 1406 4285 13
Donor3-CGTCCATTCAGCGATT-1 1076 5437 8
Donor3-CGTCCATTCCAGAAGG-1 1237 5662 8
Donor3-CGTCTACGTATAGGTA-1 2044 6094 12
Donor3-CGTCTACGTGTCCTCT-1 2586 8780 11
Donor3-CGTGAGCAGCAGGTCA-1 2775 9917 10
Donor3-CGTGAGCGTGTATGGG-1 5710 32672 5
Donor3-CGTGAGCTCTGCGACG-1 4337 12151 6
Donor3-CGTGTAAAGAAGGCCT-1 1333 8215 8
Donor3-CGTGTAAGTAAACGCG-1 1124 5149 8
Donor3-CGTGTCTCAGTCAGAG-1 1139 6103 8
Donor3-CGTGTCTTCCGTACAA-1 3229 7535 3
Donor3-CGTTAGAAGTTTCCTT-1 2519 13868 7
Donor3-CGTTAGAGTCGCGGTT-1 1329 6940 8
Donor3-CGTTCTGCAATGTTGC-1 1095 4347 8
Donor3-CGTTCTGGTAAATGAC-1 1489 9527 8
Donor3-CGTTCTGGTATCTGCA-1 3248 9366 10
Donor3-CGTTCTGTCCAGTATG-1 1175 6882 8
Donor3-CGTTCTGTCTAACGGT-1 3287 11836 6
Donor3-CGTTGGGAGTTTGCGT-1 2991 6393 3
Donor3-CTAACTTTCCACGACG-1 7002 34430 5
Donor3-CTAACTTTCGATGAGG-1 2073 4540 3
Donor3-CTAAGACGTGCGAAAC-1 1745 6743 9
Donor3-CTAATGGAGAGTCTGG-1 3377 12869 4
Donor3-CTAATGGCAAAGCGGT-1 3102 26127 8
Donor3-CTAATGGCATTGGCGC-1 2592 13932 7
Donor3-CTACACCAGCTGAACG-1 1016 4545 8
Donor3-CTACACCTCTTTACGT-1 1330 5727 7
Donor3-CTACATTCATCGGAAG-1 2927 15653 7
Donor3-CTACATTTCAATAAGG-1 1189 5756 8
Donor3-CTACCCACAACACCCG-1 4611 14223 4
Donor3-CTACCCAGTGACCAAG-1 2651 10646 6
Donor3-CTACCCATCGATCCCT-1 2755 9552 10
Donor3-CTACGTCTCCCGACTT-1 1650 4907 10
Donor3-CTACGTCTCTATCCTA-1 7934 49205 5
Donor3-CTAGAGTAGACTGGGT-1 2031 6139 10
Donor3-CTAGAGTAGCCACCTG-1 1916 9302 7
Donor3-CTAGAGTCAACACCTA-1 4430 20957 6
Donor3-CTAGCCTCAGCGTTCG-1 2561 5536 3
Donor3-CTAGCCTTCGGTTAAC-1 3350 7897 3
Donor3-CTAGTGATCCCTCAGT-1 1636 13209 8
Donor3-CTCAGAAGTTACGGAG-1 1809 5915 6
Donor3-CTCATTAGTGCAGTAG-1 2192 5608 11
Donor3-CTCCTAGGTGAGTATA-1 4344 10886 6
Donor3-CTCCTAGTCGCGTTTC-1 3804 9487 3
Donor3-CTCGAAACACGTGAGA-1 3590 8098 6
Donor3-CTCGAAATCGCCAGCA-1 1096 4564 7
Donor3-CTCGAGGTCACCGGGT-1 5451 27922 6
Donor3-CTCGGAGGTTGGAGGT-1 1911 6515 8
Donor3-CTCGGAGTCGGATGTT-1 1100 4936 8
Donor3-CTCGGGATCGAACTGT-1 3556 9183 13

Donor3-CTCGTACTCGTTACGA-1 1340 4603 7
Donor3-CTCTAATCACATGACT-1 2027 5883 10
Donor3-CTCTAATGTTAAGATG-1 1372 4542 7
Donor3-CTCTAATTCCCAGGTG-1 1725 4771 13
Donor3-CTCTGGTAGAGGTTAT-1 1604 4680 13
Donor3-CTCTGGTGTACTTAGC-1 5032 15818 5
Donor3-CTCTGGTGTAGGGTAC-1 4828 15516 6
Donor3-CTGAAACAGCCACGCT-1 1421 7092 8
Donor3-CTGAAACAGTAGGCCA-1 1403 6785 7
Donor3-CTGAAACCACTTGGAT-1 2926 6950 6
Donor3-CTGAAACTCCCTAATT-1 3575 8425 4
Donor3-CTGAAGTTCTACTTAC-1 1711 8852 8
Donor3-CTGATAGGTAAGTGGC-1 7636 39484 6
Donor3-CTGATAGGTACTCGCG-1 2717 5412 5
Donor3-CTGATCCAGATGTCGG-1 1823 13147 8
Donor3-CTGATCCTCCGAACGC-1 4421 18852 6
Donor3-CTGCCTAGTATCACCA-1 2004 4719 6
Donor3-CTGCGGACAATTGCTG-1 4757 23817 6
Donor3-CTGCGGACAGGTCGTC-1 3614 8969 2
Donor3-CTGCGGAGTAGAGCTG-1 3194 7314 3
Donor3-CTGCGGAGTCGTTGTA-1 2317 10861 12
Donor3-CTGCGGATCCTCAACC-1 2090 4837 4
Donor3-CTGCGGATCGGATGTT-1 4354 40497 7
Donor3-CTGCTGTCAACACCTA-1 2366 4636 3
Donor3-CTGCTGTTCTTAGAGC-1 1109 4900 8
Donor3-CTGTGCTAGCCATCGC-1 3528 12123 10
Donor3-CTGTGCTCAAGCCATT-1 3911 9571 2
Donor3-CTGTGCTCAGACTCGC-1 2315 12078 7
Donor3-CTGTGCTCAGCGAACA-1 2589 14154 8
Donor3-CTGTGCTTCCACGTTC-1 1287 4730 9
Donor3-CTGTGCTTCCGCAGTG-1 6856 45648 5
Donor3-CTGTTTAAGAGGGATA-1 6656 26261 5
Donor3-CTGTTTAGTACAAGTA-1 2399 12428 7
Donor3-CTGTTTAGTATGAAAC-1 6176 26490 2
Donor3-CTGTTTAGTATTACCG-1 5807 21778 6
Donor3-CTTACCGAGGATATAC-1 1520 6179 9
Donor3-CTTACCGGTACAGACG-1 4935 16478 4
Donor3-CTTACCGTCAGCACAT-1 2684 8117 4
Donor3-CTTACCGTCAGCGACC-1 3174 15702 7
Donor3-CTTACCGTCATGCTCC-1 1271 5493 8
Donor3-CTTACCGTCATGTGGT-1 2701 13837 7
Donor3-CTTACCGTCCAGATCA-1 8716 85471 6
Donor3-CTTAGGAAGAAACCGC-1 6603 29120 6
Donor3-CTTAGGACACCCATGG-1 2221 13502 7
Donor3-CTTAGGAGTTGGACCC-1 2522 5965 5
Donor3-CTTAGGATCCAGGGCT-1 2021 7127 13
Donor3-CTTCTCTAGAGCTGCA-1 4025 10584 4
Donor3-CTTGGCTAGCGCCTTG-1 5613 19766 2
Donor3-CTTGGCTCAGGGAGAG-1 1359 5659 7
Donor3-CTTGGCTGTCGACTGC-1 8513 63748 6
Donor3-CTTTGCGGTCTAAAGA-1 5845 21606 5
Donor3-CTTTGCGTCATTTGGG-1 2187 13478 7
Donor3-GAAACTCCAGTTTACG-1 6203 36160 6
Donor3-GAAACTCTCTAACGGT-1 2566 5803 6
Donor3-GAAATGAAGGGCTTGA-1 7245 34199 5
Donor3-GAAATGACACCTGGTG-1 6365 55187 6

Donor3-GAAATGAGTGTCAATC-1 926 4309 8
Donor3-GAAATGATCATGCAAC-1 1183 5716 8
Donor3-GAACATCAGCTGAACG-1 1500 4378 9
Donor3-GAACCTACAAGCCGTC-1 1617 5470 9
Donor3-GAACCTATCACAATGC-1 6392 25301 2
Donor3-GAACCTATCGTTACGA-1 1189 6323 8
Donor3-GAACCTATCTCTGCTG-1 1471 5324 7
Donor3-GAACGGAAGGAATTAC-1 4169 17707 4
Donor3-GAACGGAGTCCATGAT-1 1602 7719 8
Donor3-GAACGGAGTGTTTGTG-1 3963 10043 1
Donor3-GAACGGATCAACTCTT-1 1753 4615 11
Donor3-GAAGCAGCAATAGAGT-1 1902 5055 13
Donor3-GAAGCAGCAGTCCTTC-1 4573 19337 6
Donor3-GAAGCAGGTTCACCTC-1 5402 20242 2
Donor3-GAAGCAGTCAGTACGT-1 1869 6156 13
Donor3-GAAGCAGTCCAAACTG-1 8020 96922 5
Donor3-GAAGCAGTCGGCTACG-1 1319 4372 7
Donor3-GAATAAGTCTCATTCA-1 2982 7607 5
Donor3-GAATGAAGTTCGGCAC-1 3048 15631 7
Donor3-GAATGAATCCCTTGTG-1 5112 17553 2
Donor3-GACACGCTCATCTGTT-1 2296 9804 7
Donor3-GACAGAGAGGGAAACA-1 6507 23709 5
Donor3-GACAGAGGTGGACGAT-1 3026 9775 10
Donor3-GACAGAGTCATATCGG-1 7722 78589 4
Donor3-GACCAATTCATGCTCC-1 2428 5236 3
Donor3-GACGCGTGTCAATACC-1 5856 22752 6
Donor3-GACGGCTAGAAGGGTA-1 2330 7389 12
Donor3-GACGGCTCAGACAAGC-1 1629 4462 13
Donor3-GACGGCTGTCCGAAGA-1 1080 4306 8
Donor3-GACGGCTGTCCGCTGA-1 3271 7907 3
Donor3-GACGTTAAGCACCGCT-1 5261 18438 1
Donor3-GACTAACAGGCCATAG-1 3755 9550 3
Donor3-GACTAACAGGCGATAC-1 2578 6977 11
Donor3-GACTAACCAATCGAAA-1 1199 5442 8
Donor3-GACTAACCAGCTTAAC-1 4032 20663 6
Donor3-GACTACAAGTGGACGT-1 1580 5870 9
Donor3-GACTACAAGTTCGCGC-1 1323 5149 8
Donor3-GACTGCGGTGTGGCTC-1 3172 17333 7
Donor3-GAGTCCGCATGTTGAC-1 2783 11960 7
Donor3-GAGTCCGTCTGTTTGT-1 2576 5056 2
Donor3-GATCAGTGTATAATGG-1 1990 4832 4
Donor3-GATCAGTTCACCAGGC-1 1447 8489 8
Donor3-GATCGATGTCTACCTC-1 1499 4383 13
Donor3-GATCGCGCATAACCTG-1 3050 6795 2
Donor3-GATCGCGTCGGTCTAA-1 1859 10582 7
Donor3-GATCGTACATCGATTG-1 1937 10571 7
Donor3-GATCGTATCAATCTCT-1 1640 7995 8
Donor3-GATCTAGCACAAGACG-1 1335 4616 7
Donor3-GATCTAGGTCTCTTAT-1 3473 9111 6
Donor3-GATCTAGTCTGTACGA-1 3796 9883 3
Donor3-GATGAAACAGAGTGTG-1 1400 4609 7
Donor3-GATGAAAGTCAACTGT-1 1977 10213 7
Donor3-GATGAAATCAACACGT-1 1515 6663 7
Donor3-GATGAGGAGTAGGTGC-1 2237 13433 7
Donor3-GATGAGGGTAAGTGTA-1 1498 4510 13
Donor3-GATGAGGGTCAGCTAT-1 3113 13843 6

Donor3-GATGAGGGTCCCTTGT-1 2094 6919 6
Donor3-GATGAGGGTCGGCACT-1 1899 9950 7
Donor3-GATGCTATCTGTCTAT-1 1470 4501 13
Donor3-GATTCAGGTAGCGATG-1 3757 10774 2
Donor3-GATTCAGGTGACTACT-1 2818 11646 6
Donor3-GCAAACTAGGGATACC-1 2752 5956 4
Donor3-GCAAACTGTCACTTCC-1 1957 7641 7
Donor3-GCAATCAAGACTAAGT-1 3139 16147 7
Donor3-GCAATCAGTAAACACA-1 2365 16267 8
Donor3-GCACATAGTTAGAACA-1 4712 15085 1
Donor3-GCACTCTGTCGAGTTT-1 1475 4421 13
Donor3-GCACTCTGTTTAGGAA-1 3793 10243 3
Donor3-GCACTCTTCAAGGTAA-1 2012 6216 13
Donor3-GCAGCCACAAATTGCC-1 4812 19326 6
Donor3-GCAGCCACAGGACGTA-1 2174 14081 8
Donor3-GCAGCCAGTCGCATAT-1 7015 60514 6
Donor3-GCAGCCAGTTAAGTAG-1 5323 16733 3
Donor3-GCAGTTAGTCTGATCA-1 1648 6248 8
Donor3-GCATACACACAAGTAA-1 3308 11756 5
Donor3-GCATACAGTGCCTGCA-1 3714 10251 2
Donor3-GCATGATAGTACGATA-1 5186 34263 6
Donor3-GCATGATCACTGTGTA-1 1974 7476 7
Donor3-GCATGATGTACGAAAT-1 3231 16774 7
Donor3-GCATGATTCGATAGAA-1 3582 7921 3
Donor3-GCATGCGCACGAAAGC-1 2073 8414 9
Donor3-GCATGCGGTAACGTTC-1 1755 4331 10
Donor3-GCATGCGGTCCGCTGA-1 4097 18506 6
Donor3-GCATGCGGTGCCTGCA-1 1397 6770 8
Donor3-GCATGCGGTGTGAAAT-1 1349 6793 8
Donor3-GCCAAATCACAAGCCC-1 4887 14315 6
Donor3-GCCAAATCACTAGTAC-1 4019 10579 3
Donor3-GCCAAATCAGGGTACA-1 1852 5774 9
Donor3-GCCAAATCAGTCGATT-1 4457 18953 6
Donor3-GCCAAATTCTCCAACC-1 5717 20588 6
Donor3-GCCTCTAGTAGCTCCG-1 1967 9263 7
Donor3-GCCTCTATCTAACTTC-1 3364 7168 2
Donor3-GCCTCTATCTATCGCC-1 3797 9091 3
Donor3-GCGACCAAGCCGCCTA-1 1563 10363 8
Donor3-GCGACCAAGTGAAGTT-1 1133 5584 8
Donor3-GCGACCAGTCGCCATG-1 9076 69585 1
Donor3-GCGAGAAGTGGACGAT-1 2573 11056 6
Donor3-GCGAGAATCGTCTGAA-1 6712 27828 1
Donor3-GCGCAACCAGATGGGT-1 1926 6556 9
Donor3-GCGCAACCATCCAACA-1 2439 6771 13
Donor3-GCGCAGTCAAATACAG-1 1456 4270 13
Donor3-GCGCCAACATTTCACT-1 2630 9169 10
Donor3-GCGCCAAGTCGGCATC-1 3941 9734 5
Donor3-GCGCGATAGGCGACAT-1 4743 14593 3
Donor3-GCGCGATGTCGTGGCT-1 1407 4783 13
Donor3-GCGCGATGTTGTACAC-1 3127 17083 7
Donor3-GCGCGATTCAGCTTAG-1 1029 4481 8
Donor3-GCGGGTTGTTCACGGC-1 3007 6555 3
Donor3-GCGGGTTTCCCGGATG-1 1708 5031 13
Donor3-GCTCCTACAGTCACTA-1 1789 5816 13
Donor3-GCTCCTAGTAGGCTGA-1 7473 44437 5
Donor3-GCTCCTATCATGCTCC-1 1761 5330 9

Donor3-GCTCTGTCAATGGTCT-1 2312 12103 7
Donor3-GCTCTGTGTCGTCTTC-1 3291 6904 6
Donor3-GCTCTGTTCTTGCAAG-1 7863 60544 6
Donor3-GCTGCAGGTACTTGAC-1 1746 5788 13
Donor3-GCTGCAGGTTCTGTTT-1 3387 8090 3
Donor3-GCTGCTTCATGTCGAT-1 2215 11987 7
Donor3-GCTGGGTCAAGGCTCC-1 2111 8342 7
Donor3-GCTGGGTGTCAAACTC-1 5324 17380 2
Donor3-GCTGGGTGTTCCACAA-1 1688 8143 7
Donor3-GCTTCCAAGTCGTTTG-1 3385 23721 8
Donor3-GCTTGAAAGAGCTGCA-1 2655 13598 7
Donor3-GCTTGAAAGCCGGTAA-1 1387 4796 13
Donor3-GCTTGAAAGGCTCAGA-1 6151 25512 5
Donor3-GCTTGAACAGCTCGCA-1 2600 9445 6
Donor3-GCTTGAATCTCTTATG-1 2120 11700 8
Donor3-GGAAAGCGTACCCAAT-1 1648 7304 7
Donor3-GGAAAGCGTGTGCGTC-1 1186 4930 7
Donor3-GGAAAGCGTTCCCGAG-1 1412 5506 7
Donor3-GGAACTTAGTACGTAA-1 1439 8372 8
Donor3-GGAACTTGTGGTCTCG-1 1524 9922 8
Donor3-GGAATAACATGGAATA-1 4441 20187 5
Donor3-GGAATAAGTACTCAAC-1 1161 4401 8
Donor3-GGAATAATCAGATAAG-1 3503 8264 3
Donor3-GGAATAATCCTAGTGA-1 963 4463 8
Donor3-GGACAGAAGTGAATTG-1 4904 16407 2
Donor3-GGACAGAGTGATGATA-1 2639 9888 6
Donor3-GGACAGAGTTCAGGCC-1 3401 12257 4
Donor3-GGACAGATCTTGTATC-1 3813 9260 3
Donor3-GGACATTAGTTTCCTT-1 2240 13586 7
Donor3-GGACGTCGTGTGCGTC-1 5139 16344 2
Donor3-GGACGTCTCAGGATCT-1 3578 8131 4
Donor3-GGACGTCTCAGTTGAC-1 3750 9746 3
Donor3-GGATGTTAGAGACTAT-1 1781 9086 7
Donor3-GGATTACTCGGCTTGG-1 5486 22654 5
Donor3-GGCAATTAGATGTTAG-1 1570 8509 8
Donor3-GGCAATTGTATTACCG-1 1249 4957 7
Donor3-GGCAATTGTCGCGTGT-1 1258 4836 7
Donor3-GGCAATTTCCTCAATT-1 1016 5539 8
Donor3-GGCAATTTCTAACTTC-1 2731 5883 5
Donor3-GGCCGATAGGAGTAGA-1 3143 6353 6
Donor3-GGCCGATGTACCTACA-1 3851 18301 4
Donor3-GGCCGATTCGTGGTCG-1 2279 10191 7
Donor3-GGCCGATTCTCCTATA-1 1451 4347 13
Donor3-GGCCGATTCTTCTGGC-1 2151 16528 8
Donor3-GGCGACTAGACTTTCG-1 1574 6512 7
Donor3-GGCGACTGTGGGTCAA-1 5366 45490 7
Donor3-GGCGTGTGTGTGAAAT-1 942 4433 8
Donor3-GGCTCGAAGCCTTGAT-1 2495 12512 7
Donor3-GGCTCGACATATGCTG-1 8001 46889 6
Donor3-GGCTCGAGTACAAGTA-1 2812 5908 3
Donor3-GGCTCGATCACTATTC-1 2596 9229 6
Donor3-GGGAATGAGAATTCCC-1 2577 8046 11
Donor3-GGGAATGCACGACGAA-1 2637 7240 10
Donor3-GGGAATGCACTGTGTA-1 1221 4710 8
Donor3-GGGAATGTCTTGAGGT-1 3965 12285 6
Donor3-GGGACCTCATTCTTAC-1 6214 24104 6

Donor3-GGGAGATCACCAGTTA-1 5008 34671 6
Donor3-GGGAGATGTCAAGCGA-1 1927 5209 13
Donor3-GGGAGATTCTGTCTCG-1 1757 6208 9
Donor3-GGGATGAAGAGCTATA-1 4313 13190 6
Donor3-GGGATGACATGGGAAC-1 1520 5498 7
Donor3-GGGCACTCAGGATCGA-1 2331 4752 5
Donor3-GGGCACTTCGATCCCT-1 1347 4422 9
Donor3-GGGCATCGTCCAGTGC-1 1440 6204 8
Donor3-GGGCATCGTTACGCGC-1 2074 14953 8
Donor3-GGGTCTGAGATCTGCT-1 7611 55848 6
Donor3-GGGTCTGCAAGGGTCA-1 6407 32814 5
Donor3-GGGTCTGCAGACTCGC-1 3588 9553 6
Donor3-GGGTCTGGTGTGAAAT-1 1670 5371 6
Donor3-GGGTCTGTCCGGCACA-1 6994 32512 6
Donor3-GGGTTGCAGAGTACAT-1 2296 15507 7
Donor3-GGGTTGCCACTACAGT-1 4571 14397 3
Donor3-GGGTTGCGTCGGCTCA-1 1654 4292 10
Donor3-GGTATTGCAGCCAATT-1 3489 8755 3
Donor3-GGTATTGTCCTCTAGC-1 4661 15543 1
Donor3-GGTGAAGCAGTAAGCG-1 1588 5299 10
Donor3-GGTGAAGCATCACGTA-1 3437 9955 5
Donor3-GGTGAAGTCAACACCA-1 2299 7658 11
Donor3-GGTGAAGTCAGCTCGG-1 956 4528 8
Donor3-GGTGCGTAGACGACGT-1 3005 17907 7
Donor3-GGTGCGTCAAAGGTGC-1 1947 14729 8
Donor3-GGTGCGTGTCTTCTCG-1 1462 5767 7
Donor3-GGTGTTAGTGATAAAC-1 2399 5032 6
Donor3-GGTGTTAGTGCACGAA-1 2403 7593 13
Donor3-GTAACGTTCCGTAGTA-1 2480 4596 3
Donor3-GTAACGTTCTGAAAGA-1 4143 11872 1
Donor3-GTAACTGAGACAGAGA-1 1732 8986 7
Donor3-GTAACTGAGGAGCGAG-1 1888 5747 10
Donor3-GTAACTGAGTGGACGT-1 1306 4852 7
Donor3-GTAACTGCACCACGTG-1 4746 14008 5
Donor3-GTAACTGGTCGAATCT-1 3431 10681 6
Donor3-GTAACTGGTCTCAACA-1 5317 19388 5
Donor3-GTACTCCCAGATAATG-1 1469 6275 7
Donor3-GTACTCCGTGCCTTGG-1 2777 10305 6
Donor3-GTACTCCTCGACCAGC-1 5531 20160 5
Donor3-GTACTTTAGGCGATAC-1 1850 5697 13
Donor3-GTACTTTTCTCGGACG-1 2214 8801 9
Donor3-GTAGGCCCACATCCAA-1 4316 12545 2
Donor3-GTAGGCCCAGCTGTAT-1 1243 6298 8
Donor3-GTAGGCCGTTCAGCGC-1 1654 5437 13
Donor3-GTAGGCCTCAAAGACA-1 6063 26673 4
Donor3-GTAGTCAGTCGAATCT-1 1741 5875 9
Donor3-GTATTCTAGCTGCAAG-1 1780 5198 7
Donor3-GTATTCTCACGTTGGC-1 1346 4510 9
Donor3-GTATTCTCAGCTGCTG-1 1552 5024 7
Donor3-GTATTCTGTGTTAAGA-1 1668 5625 13
Donor3-GTATTCTGTTCTGTTT-1 2393 4430 6
Donor3-GTATTCTTCGGAGCAA-1 5082 15989 5
Donor3-GTCAAGTAGGGCTCTC-1 2742 24178 8
Donor3-GTCAAGTAGGTGTGGT-1 1532 5118 13
Donor3-GTCAAGTCAGGATCGA-1 1837 6860 7
Donor3-GTCAAGTTCCGCGTTT-1 1343 6463 8

Donor3-GTCACAAAGCTAGTCT-1 2744 7340 12
Donor3-GTCACAACAGCTGTGC-1 5156 17340 2
Donor3-GTCACAATCAACCAAC-1 3585 7821 1
Donor3-GTCATTTAGGGATACC-1 1211 4912 7
Donor3-GTCATTTAGGGCTCTC-1 2345 15649 7
Donor3-GTCATTTGTCCAAGTT-1 7041 27514 1
Donor3-GTCCTCAAGACACGAC-1 4468 13915 5
Donor3-GTCCTCAGTGAGCGAT-1 2052 10999 7
Donor3-GTCGGGTTCCGCAAGC-1 6218 22796 3
Donor3-GTCGTAAAGCTAGTCT-1 4369 12120 1
Donor3-GTCGTAAAGGACAGAA-1 1910 5105 13
Donor3-GTCGTAACACCAGATT-1 5505 19901 6
Donor3-GTCGTAACATTGGTAC-1 3001 15899 7
Donor3-GTCGTAAGTCGACTAT-1 4969 15827 6
Donor3-GTCGTAAGTTGGTGGA-1 1393 6296 8
Donor3-GTCTCGTTCTTACCGC-1 1096 4535 8
Donor3-GTCTTCGCAAGTCATC-1 5623 21489 4
Donor3-GTCTTCGCATAAGACA-1 3698 9867 1
Donor3-GTCTTCGCATCTATGG-1 3989 9403 2
Donor3-GTCTTCGGTTCGGCAC-1 1549 7027 8
Donor3-GTCTTCGTCGTCTGCT-1 2500 5927 6
Donor3-GTGAAGGGTCTCTCTG-1 1520 9332 8
Donor3-GTGCAGCCAGAAGCAC-1 3623 9188 3
Donor3-GTGCAGCCATTCCTCG-1 7145 53582 6
Donor3-GTGCAGCTCGCCATAA-1 7863 41882 6
Donor3-GTGCAGCTCGTTACGA-1 1371 7014 8
Donor3-GTGCATAAGCTAAACA-1 3568 8799 3
Donor3-GTGCATACAGTAAGCG-1 2394 11518 7
Donor3-GTGCATACATCACGTA-1 1709 6956 7
Donor3-GTGCATACATTTCACT-1 1589 4914 9
Donor3-GTGCATACATTTGCTT-1 3911 9796 2
Donor3-GTGCGGTAGTACACCT-1 7602 42282 1
Donor3-GTGCGGTCAAGTTAAG-1 2091 11578 7
Donor3-GTGCGGTGTCATATGC-1 3641 16570 6
Donor3-GTGGGTCAGGGTTCCC-1 2255 9587 7
Donor3-GTGGGTCTCTTTACGT-1 1581 4322 9
Donor3-GTGTGCGAGGACAGAA-1 5259 16767 5
Donor3-GTGTTAGAGGCCATAG-1 3155 10638 4
Donor3-GTGTTAGGTGCAGGTA-1 9594 60381 1
Donor3-GTTAAGCTCCAGAAGG-1 1327 5430 7
Donor3-GTTACAGCACAGGCCT-1 1561 10635 8
Donor3-GTTACAGCAGGACGTA-1 912 4868 8
Donor3-GTTACAGCAGGGATTG-1 2725 11552 7
Donor3-GTTCATTCAATCACAC-1 2239 6809 13
Donor3-GTTCATTCACAGGCCT-1 1919 6696 9
Donor3-GTTCATTGTGAAGGCT-1 4641 13631 6
Donor3-GTTCGGGCAATACGCT-1 3725 9405 3
Donor3-GTTCGGGTCTGCTTGC-1 2230 9020 9
Donor3-GTTCTCGAGAGCTGGT-1 3235 6356 5
Donor3-GTTCTCGGTGGGTCAA-1 1429 4421 10
Donor3-GTTCTCGTCCCTCAGT-1 6120 26002 5
Donor3-GTTTCTACAAGCTGTT-1 5627 30926 5
Donor3-GTTTCTAGTACTCGCG-1 2151 5167 6
Donor3-TAAACCGAGCTCCCAG-1 2783 8591 10
Donor3-TAAACCGAGTGGTAGC-1 3319 7642 3
Donor3-TAAGAGATCCTGCCAT-1 6797 31933 5

Donor3-TAAGCGTCAGGTTTCA-1 3486 14849 10
Donor3-TAAGCGTGTAGAAGGA-1 6224 28529 2
Donor3-TAAGTGCCAAATACAG-1 1137 5643 8
Donor3-TACACGAGTGTCAATC-1 1555 7276 8
Donor3-TACAGTGAGCCGTCGT-1 4889 15472 5
Donor3-TACCTATCATTGGGCC-1 1362 8198 8
Donor3-TACCTATGTAAGGATT-1 2877 13860 7
Donor3-TACCTATTCATAACCG-1 2337 7063 13
Donor3-TACCTATTCGCGTAGC-1 7969 35743 3
Donor3-TACCTTAGTTCAGACT-1 3377 8053 3
Donor3-TACCTTATCTGGAGCC-1 1648 9300 8
Donor3-TACGGATGTCCTCCAT-1 1295 6743 8
Donor3-TACGGATGTTTGACTG-1 2948 14407 7
Donor3-TACGGTAAGTATCGAA-1 3807 10174 3
Donor3-TACGGTACAGATCCAT-1 841 4389 8
Donor3-TACGGTAGTCTGCGGT-1 1905 9806 7
Donor3-TACTCATCATGGATGG-1 1848 7164 9
Donor3-TACTCGCCAAGAAGAG-1 1575 8041 7
Donor3-TACTTACTCCCGACTT-1 2275 5976 13
Donor3-TACTTGTAGAGCTGGT-1 2817 10824 9
Donor3-TACTTGTAGTCCGGTC-1 3077 19325 7
Donor3-TACTTGTAGTGTACTC-1 1034 4804 8
Donor3-TACTTGTCAGCCTATA-1 2597 4426 6
Donor3-TACTTGTGTCGGATCC-1 1274 6406 8
Donor3-TAGACCAAGAGCTATA-1 4119 11866 4
Donor3-TAGACCAAGGCCCGTT-1 4859 19381 6
Donor3-TAGACCACACAGTCGC-1 1741 5321 13
Donor3-TAGAGCTAGATGTAAC-1 7007 53632 4
Donor3-TAGAGCTAGCGGCTTC-1 4879 14471 2
Donor3-TAGCCGGTCAGCGATT-1 4416 16126 5
Donor3-TAGGCATAGACAAGCC-1 1274 7869 8
Donor3-TAGGCATAGAGCTATA-1 1962 11918 8
Donor3-TAGGCATCATAGAAAC-1 1732 5443 10
Donor3-TAGGCATGTCATATGC-1 2149 13430 8
Donor3-TAGGCATGTTCCGTCT-1 1728 5361 13
Donor3-TAGTGGTGTAACGCGA-1 1601 4597 13
Donor3-TAGTGGTGTGCCTGCA-1 1350 6634 8
Donor3-TAGTTGGAGTAGCGGT-1 1606 4711 7
Donor3-TAGTTGGTCTCGCTTG-1 3644 10111 6
Donor3-TATCAGGAGGGTGTGT-1 5640 31758 6
Donor3-TATCAGGCACCCTATC-1 3128 19969 7
Donor3-TATCAGGGTGGGTATG-1 1779 7513 7
Donor3-TATCAGGTCAGCGACC-1 3413 14274 6
Donor3-TATCTCAAGTACCGGA-1 2261 6630 11
Donor3-TATCTCACACATCCAA-1 3288 7530 3
Donor3-TATCTCAGTACGCTGC-1 3422 8337 3
Donor3-TATCTCAGTGTAAGTA-1 3911 14475 6
Donor3-TATGCCCCACATTTCT-1 1675 6729 7
Donor3-TATTACCCAGTAACGG-1 1525 5719 7
Donor3-TCAACGAAGAAGCCCA-1 3217 17598 7
Donor3-TCAACGAAGCTAGTTC-1 1425 6573 8
Donor3-TCAACGATCCACGTTC-1 3602 12139 4
Donor3-TCAATCTAGGTGATTA-1 4455 22632 4
Donor3-TCAATCTAGTTAGCGG-1 4134 10603 2
Donor3-TCAATCTGTACCGTAT-1 2710 6006 5
Donor3-TCAATCTTCCTTAATC-1 3384 19942 7

Donor3-TCACAAGCACGAAGCA-1 1613 5884 7
Donor3-TCACAAGGTAGCGTCC-1 1649 5656 9
Donor3-TCACAAGTCAATCTCT-1 3651 8931 3
Donor3-TCACGAACACCTCGTT-1 1668 7230 7
Donor3-TCAGATGAGATATGGT-1 4952 27007 5
Donor3-TCAGATGTCAAGCCTA-1 3733 16024 6
Donor3-TCAGATGTCATAACCG-1 2054 6075 13
Donor3-TCAGATGTCTACGAGT-1 3030 7011 5
Donor3-TCAGCAAGTTTGACAC-1 1393 7801 8
Donor3-TCAGCAATCTCTGCTG-1 1378 7760 8
Donor3-TCAGCAATCTGCGGCA-1 4743 13579 2
Donor3-TCAGGATAGGCGTACA-1 1650 4378 10
Donor3-TCAGGATGTGAGCGAT-1 7950 50579 5
Donor3-TCAGGATTCGCTAGCG-1 4630 13503 5
Donor3-TCAGGATTCTAACGGT-1 3144 7163 3
Donor3-TCAGGTAAGGGATACC-1 1896 12594 8
Donor3-TCAGGTAAGGTGACCA-1 1086 5461 8
Donor3-TCATTACAGTGTACCT-1 2333 8105 10
Donor3-TCATTACGTACCGCTG-1 3820 9534 3
Donor3-TCATTTGAGCGAGAAA-1 4253 15476 6
Donor3-TCATTTGTCAGCTGGC-1 4669 22514 6
Donor3-TCATTTGTCAGTTGAC-1 5893 21179 2
Donor3-TCCACACGTCAGATAA-1 1758 6129 6
Donor3-TCCACACTCACAAACC-1 3094 21631 7
Donor3-TCCCGATCATACAGCT-1 1913 7074 9
Donor3-TCCCGATTCATCTGTT-1 1763 5365 13
Donor3-TCCCGATTCTTTCCTC-1 1051 4433 8
Donor3-TCGAGGCAGTGACTCT-1 4088 9907 5
Donor3-TCGAGGCAGTTTAGGA-1 2918 6623 4
Donor3-TCGAGGCCAGGATCGA-1 2031 4698 4
Donor3-TCGAGGCGTTAAAGAC-1 3373 7866 6
Donor3-TCGAGGCGTTTGACTG-1 2587 5241 3
Donor3-TCGAGGCTCGCGTTTC-1 2178 7263 10
Donor3-TCGAGGCTCTTAGCCC-1 3253 15972 7
Donor3-TCGCGAGAGCTCTCGG-1 1139 5656 8
Donor3-TCGCGAGGTTAAGTAG-1 2142 5252 11
Donor3-TCGCGAGTCCACTCCA-1 1543 7883 8
Donor3-TCGCGAGTCTGTCTAT-1 2391 7923 7
Donor3-TCGCGTTCAAAGTCAA-1 5333 17852 2
Donor3-TCGCGTTGTACTCGCG-1 4374 11827 5
Donor3-TCGCGTTGTGCACTTA-1 1631 10442 8
Donor3-TCGCGTTTCTAACCGA-1 1558 7491 7
Donor3-TCGGGACAGGCTAGAC-1 2702 5543 6
Donor3-TCGGGACTCATCGATG-1 1025 4411 8
Donor3-TCGGGACTCATTTGGG-1 3174 12792 6
Donor3-TCGGGACTCGCAGGCT-1 1131 4924 8
Donor3-TCGGTAAAGGATGGAA-1 3764 18362 6
Donor3-TCGTACCCAAGCCGTC-1 1732 4393 10
Donor3-TCGTAGAGTCTCGTTC-1 7421 30056 1
Donor3-TCGTAGATCTTCGAGA-1 3831 9577 5
Donor3-TCTCATACAATGCCAT-1 1843 9455 8
Donor3-TCTCATACAGACGTAG-1 3798 14659 6
Donor3-TCTCATATCATGCTCC-1 1083 5254 8
Donor3-TCTCATATCATTGCGA-1 2019 5536 10
Donor3-TCTCTAAAGGGTGTTG-1 2082 9554 7
Donor3-TCTCTAAAGTTATCGC-1 1643 7509 7

Donor3-TCTCTAAGTAGCTGCC-1 4652 14632 3
Donor3-TCTCTAATCAGCTCGG-1 5651 21856 4
Donor3-TCTCTAATCCAGATCA-1 1409 4567 13
Donor3-TCTGAGAAGTGGTAAT-1 1397 5374 7
Donor3-TCTGAGACAAACAACA-1 1078 5187 8
Donor3-TCTGAGACACTCAGGC-1 4593 14620 2
Donor3-TCTGAGACAGTTCATG-1 1218 7001 8
Donor3-TCTGAGAGTCGTGGCT-1 2715 6357 3
Donor3-TCTGGAACATCGATTG-1 3018 6427 3
Donor3-TCTTCGGAGCGGATCA-1 1012 4570 8
Donor3-TCTTCGGCATAGACTC-1 1943 10232 7
Donor3-TCTTCGGTCCTCCTAG-1 1929 6440 13
Donor3-TCTTTCCCAGGGTACA-1 2575 4999 6
Donor3-TCTTTCCCATTCCTGC-1 4274 19429 6
Donor3-TGAAAGACAAAGGCGT-1 5381 18913 6
Donor3-TGACAACAGAGGACGG-1 2159 5797 4
Donor3-TGACAACCACGCCAGT-1 1973 10917 8
Donor3-TGACAACGTATGGTTC-1 3899 11107 3
Donor3-TGACAACGTGTAATGA-1 2229 6373 11
Donor3-TGACGGCGTAAGTGTA-1 3776 8793 5
Donor3-TGACTTTCAATGTAAG-1 3638 20378 7
Donor3-TGACTTTGTTACAGAA-1 3519 8739 3
Donor3-TGAGAGGGTGCGCTTG-1 1545 4699 13
Donor3-TGAGCATAGTCTCAAC-1 6800 31536 5
Donor3-TGAGCATAGTGTTAGA-1 1169 5441 8
Donor3-TGAGCATGTGCCTGTG-1 2380 16541 8
Donor3-TGAGCATGTTACCAGT-1 2517 7281 4
Donor3-TGAGCCGCACGAGGTA-1 1537 5669 9
Donor3-TGAGGGAGTCCTCTTG-1 1440 5216 9
Donor3-TGATTTCCAATTCCTT-1 2529 8412 10
Donor3-TGCACCTTCATGTCCC-1 2738 9764 13
Donor3-TGCCAAAAGCGATGAC-1 1528 9332 8
Donor3-TGCCAAAAGTCGATAA-1 1765 4684 10
Donor3-TGCCCATAGCAGACTG-1 2018 8605 9
Donor3-TGCCCATCAGGGAGAG-1 2973 6431 3
Donor3-TGCCCATTCGTGACAT-1 4310 11227 3
Donor3-TGCCCTAGTACTTCTT-1 3279 16574 7
Donor3-TGCCCTAGTGGTTTCA-1 2490 9300 10
Donor3-TGCCCTATCTACTATC-1 1989 4573 4
Donor3-TGCGCAGAGGCCCGTT-1 1454 6881 8
Donor3-TGCGCAGGTAGGCATG-1 2124 5931 11
Donor3-TGCGCAGGTCGAGATG-1 5071 14717 2
Donor3-TGCGCAGGTTGGACCC-1 4094 9989 2
Donor3-TGCGGGTAGGACATTA-1 1887 7375 7
Donor3-TGCGGGTCATCGACGC-1 1294 6685 8
Donor3-TGCGGGTGTGCAACTT-1 2029 11284 8
Donor3-TGCGGGTTCTGATACG-1 2059 5723 10
Donor3-TGCGTGGCACACCGCA-1 3879 10590 6
Donor3-TGCGTGGTCACCAGGC-1 2520 10153 6
Donor3-TGCGTGGTCCCTCTTT-1 1459 5744 7
Donor3-TGCGTGGTCTCACATT-1 1912 7023 13
Donor3-TGCGTGGTCTTTAGTC-1 4720 13368 5
Donor3-TGCTACCAGCTGTCTA-1 4387 22002 6
Donor3-TGCTACCAGTCACGCC-1 4574 15165 2
Donor3-TGCTACCCATCCCACT-1 3965 17807 5
Donor3-TGCTACCTCCAAAGTC-1 5619 19392 5

Donor3-TGGACGCCATGTTGAC-1 1448 6214 7
Donor3-TGGACGCGTACACCGC-1 1954 4918 13
Donor3-TGGACGCGTGTTCTTT-1 5275 16740 1
Donor3-TGGACGCTCAAAGTAG-1 1744 5661 13
Donor3-TGGCCAGCAACTGGCC-1 2153 9712 7
Donor3-TGGCGCACAAACTGCT-1 3957 21059 6
Donor3-TGGCGCATCCAAACAC-1 5439 21503 6
Donor3-TGGCTGGAGGTTACCT-1 5864 37474 6
Donor3-TGGCTGGCATGAAGTA-1 1758 11127 8
Donor3-TGGGAAGCAAGGGTCA-1 2814 5863 3
Donor3-TGGGAAGTCGCGCCAA-1 2575 11374 7
Donor3-TGGGCGTAGCTAAGAT-1 4630 22593 6
Donor3-TGGGCGTGTAGCGCAA-1 3080 10164 11
Donor3-TGGGCGTTCAGTTAGC-1 1994 11587 7
Donor3-TGGTTAGAGAATTGTG-1 1712 5144 13
Donor3-TGGTTAGAGAGCCCAA-1 4165 11376 6
Donor3-TGGTTAGTCACATGCA-1 2720 9599 6
Donor3-TGGTTAGTCCCAAGTA-1 1395 5556 7
Donor3-TGGTTCCCAAGGACTG-1 2787 5852 5
Donor3-TGGTTCCCATATGGTC-1 1149 6686 8
Donor3-TGGTTCCGTCCCTACT-1 4875 14325 6
Donor3-TGTATTCAGGACCACA-1 2045 6121 13
Donor3-TGTATTCCACCTCGTT-1 4308 12403 4
Donor3-TGTATTCGTGTTGAGG-1 2280 6584 6
Donor3-TGTATTCTCGCAAACT-1 2919 5713 6
Donor3-TGTCCCAGTCTGGAGA-1 3399 7636 3
Donor3-TGTCCCAGTGTGGTTT-1 2451 11465 7
Donor3-TGTGGTACAAGCTGGA-1 4368 12484 1
Donor3-TGTGGTACATCATCCC-1 3240 19389 7
Donor3-TGTGGTATCAGGCCCA-1 2507 14058 7
Donor3-TGTGTTTAGCTCAACT-1 1286 6553 8
Donor3-TGTGTTTGTAAGTGGC-1 1619 5231 7
Donor3-TGTTCCGAGATCCCAT-1 4585 24582 6
Donor3-TGTTCCGGTATTACCG-1 988 4382 8
Donor3-TGTTCCGGTCTACCTC-1 4787 18099 5
Donor3-TGTTCCGGTTAGATGA-1 3027 10691 11
Donor3-TTAACTCAGCTAGTTC-1 6247 20803 1
Donor3-TTAACTCTCAAGAAGT-1 4619 13006 4
Donor3-TTAACTCTCCCGGATG-1 3493 8421 3
Donor3-TTAACTCTCTCGATGA-1 3098 7471 4
Donor3-TTAGGACCACTTCGAA-1 5951 22492 5
Donor3-TTAGGACGTAAATACG-1 1651 9802 8
Donor3-TTAGGACGTACCGTAT-1 1479 5419 13
Donor3-TTAGGCACACGACGAA-1 3849 24747 7
Donor3-TTAGTTCAGCTTATCG-1 5224 16464 3
Donor3-TTATGCTCAAATCCGT-1 5159 16121 4
Donor3-TTCCCAGAGCGCCTTG-1 1800 8931 7
Donor3-TTCCCAGTCAATCACG-1 6000 31123 5
Donor3-TTCCCAGTCGCTAGCG-1 1844 6267 13
Donor3-TTCGGTCAGCCCAGCT-1 4759 15172 5
Donor3-TTCGGTCCAGCTGTAT-1 1722 4980 6
Donor3-TTCTACAGTGGAAAGA-1 3952 9381 5
Donor3-TTCTACATCTGTACGA-1 1584 10028 8
Donor3-TTCTCAAAGATGCCTT-1 1397 5643 8
Donor3-TTCTCAAAGGCGACAT-1 3261 7938 3
Donor3-TTCTCAAGTATCGCAT-1 7267 38578 5

Donor3-TTCTCAATCACTCCTG-1 3234 7108 3
Donor3-TTCTCCTCACTTAACG-1 5410 28233 6
Donor3-TTCTCCTGTATATGGA-1 4124 11248 3
Donor3-TTCTCCTTCAGGCAAG-1 2651 9582 6
Donor3-TTGAACGAGGCCCTCA-1 2575 8095 4
Donor3-TTGAACGGTTCCCGAG-1 3427 14439 10
Donor3-TTGAACGTCCGAGCCA-1 3210 16678 7
Donor3-TTGAACGTCTCACATT-1 1793 8113 7
Donor3-TTGACTTAGTCATGCT-1 1585 5497 9
Donor3-TTGACTTGTCGCGGTT-1 5299 24190 6
Donor3-TTGACTTGTTAGTGGG-1 1653 6073 9
Donor3-TTGACTTTCGCCTGAG-1 2537 8802 6
Donor3-TTGCCGTCACTCGACG-1 3686 8594 3
Donor3-TTGCCGTCATGAACCT-1 5019 15594 5
Donor3-TTGCCGTTCGCGGATC-1 1646 10265 8
Donor3-TTGCGTCGTAAGGGAA-1 1034 4396 8
Donor3-TTGGAACAGGTGTTAA-1 3631 13670 10
Donor3-TTGGAACCAGTTTACG-1 1642 9684 8
Donor3-TTGGAACTCAGCTCGG-1 1627 4387 10
Donor3-TTGGAACTCTATCCCG-1 1693 4352 13
Donor3-TTGGCAAAGACCTAGG-1 982 4639 8
Donor3-TTGGCAACAATGAAAC-1 2684 13039 7
Donor3-TTTACTGGTGTGCCTG-1 1639 8369 8
Donor3-TTTACTGGTTGGGACA-1 1365 4346 7
Donor3-TTTACTGTCCTTTACA-1 4307 16802 4
Donor3-TTTACTGTCGCCTGTT-1 3312 14950 6
Donor3-TTTATGCAGGTAGCTG-1 2192 7279 10
Donor3-TTTATGCTCACAGGCC-1 1719 7683 7
Donor3-TTTCCTCCAGCCAATT-1 7058 30467 1
Donor3-TTTGCGCTCCCTCTTT-1 3135 6867 3
Donor3-TTTGGTTCAGCTGTAT-1 2888 19524 7
Donor3-TTTGTCACAAGTCATC-1 3533 7488 5
Donor3-AAACCTGAGGGTCGAT-2 1631 10126 8
Donor3-AAACCTGAGTAGCCGA-2 2249 12896 7
Donor3-AAACCTGGTGATGTGG-2 4409 11842 1
Donor3-AAACCTGTCTTCAACT-2 2231 5174 5
Donor3-AAACGGGAGAGACGAA-2 2553 4947 6
Donor3-AAACGGGCAGGTGCCT-2 2591 11293 6
Donor3-AAACGGGCATCTGGTA-2 2339 18118 8
Donor3-AAACGGGGTAATTGGA-2 1277 5533 8
Donor3-AAACGGGTCGATAGAA-2 4849 32669 6
Donor3-AAACGGGTCGCGGATC-2 4077 10879 3
Donor3-AAAGATGAGCTAACAA-2 2562 5467 3
Donor3-AAAGATGCATTCGACA-2 4483 17633 6
Donor3-AAAGATGGTAGAGCTG-2 4089 12362 2
Donor3-AAAGCAACACAGATTC-2 1792 7869 7
Donor3-AAAGCAAGTACTTAGC-2 3658 13284 6
Donor3-AAAGTAGCAATGACCT-2 3568 15856 10
Donor3-AAAGTAGGTACTTCTT-2 3689 8808 3
Donor3-AAATGCCCATTAGCCA-2 1097 4848 8
Donor3-AACACGTTCGTATCAG-2 1051 6182 8
Donor3-AACCATGCAGTGACAG-2 2297 11475 7
Donor3-AACCATGGTGCGATAG-2 1275 7490 8
Donor3-AACCGCGTCATGCAAC-2 5751 23620 6
Donor3-AACGTTGGTCTGATTG-2 3169 7620 3
Donor3-AACGTTGTCCGGCACA-2 5176 25141 6

Donor3-AACTCAGGTGACGCCT-2 1242 4582 7
Donor3-AACTCCCAGAAGAAGC-2 2287 4144 6
Donor3-AACTCCCAGGTACTCT-2 1729 9321 8
Donor3-AACTCCCAGTCCCACG-2 2210 15852 8
Donor3-AACTCCCCATTTCACT-2 1619 4518 13
Donor3-AACTCCCGTCTCCATC-2 1998 12582 8
Donor3-AACTCCCGTCTGGTCG-2 1153 5653 8
Donor3-AACTCCCTCCCTCAGT-2 4659 12988 2
Donor3-AACTCCCTCCGCGGTA-2 1785 12069 8
Donor3-AACTCTTCAGGAATGC-2 5341 17071 4
Donor3-AACTGGTAGCAGGTCA-2 4126 11346 6
Donor3-AACTTTCAGGTGCACA-2 1759 6103 9
Donor3-AACTTTCAGTTGTCGT-2 1465 6201 7
Donor3-AACTTTCCAACACCCG-2 1831 5866 13
Donor3-AACTTTCTCATATCGG-2 2094 5345 4
Donor3-AAGACCTCATTACGAC-2 5016 16217 6
Donor3-AAGACCTTCCCTCTTT-2 2403 9586 6
Donor3-AAGCCGCTCCTCTAGC-2 3615 19563 7
Donor3-AAGGAGCAGTGCGTGA-2 1205 5119 8
Donor3-AAGGAGCAGTTTGCGT-2 3580 25401 7
Donor3-AAGGAGCTCAAACCGT-2 5445 15533 1
Donor3-AAGGAGCTCACGCATA-2 3613 9274 3
Donor3-AAGGCAGAGCAGGTCA-2 2302 4822 3
Donor3-AAGGCAGCAGCAGTTT-2 894 4290 8
Donor3-AAGTCTGCATGCATGT-2 3630 9189 3
Donor3-AAGTCTGTCGTGACAT-2 1924 10589 7
Donor3-AATCCAGCATGCCTTC-2 1905 4445 5
Donor3-AATCCAGTCCATTCTA-2 2715 18012 8
Donor3-AATCCAGTCTTCGGTC-2 4078 10627 3
Donor3-AATCGGTAGAGGGCTT-2 1054 5013 8
Donor3-AATCGGTTCTCGTTTA-2 3515 8685 4
Donor3-ACACCAAAGAGTCGGT-2 1771 6595 9
Donor3-ACACCAAAGTCTTGCA-2 5488 35270 10
Donor3-ACACCAACAAACTGCT-2 3265 7397 4
Donor3-ACACCAAGTCAAACTC-2 1380 4723 7
Donor3-ACACCCTAGAAGATTC-2 2132 5426 6
Donor3-ACACCCTAGATGGGTC-2 1850 4947 8
Donor3-ACACCCTAGGTAGCCA-2 4740 14700 4
Donor3-ACACCCTGTAGAGTGC-2 5277 20066 4
Donor3-ACACCGGCAAGTTGTC-2 1571 5097 13
Donor3-ACACCGGTCGGAGGTA-2 5073 17069 1
Donor3-ACACTGAGTTGGGACA-2 2068 6430 10
Donor3-ACAGCCGAGGCACATG-2 1154 4242 7
Donor3-ACAGCTACATCCCATC-2 2091 8118 9
Donor3-ACAGCTAGTTAAAGAC-2 2832 13800 7
Donor3-ACATACGCATATGAGA-2 1502 8613 8
Donor3-ACATCAGAGATCCCGC-2 1313 6564 8
Donor3-ACATCAGCAGGGTATG-2 3447 8339 1
Donor3-ACATGGTAGTTGAGAT-2 4238 11835 2
Donor3-ACATGGTCAGCGTAAG-2 1601 5531 13
Donor3-ACATGGTTCTTTCCTC-2 2118 11336 7
Donor3-ACCAGTAAGATAGGAG-2 1889 5982 13
Donor3-ACCAGTACAAGGACAC-2 4140 10814 5
Donor3-ACCAGTAGTCCAGTAT-2 1227 6051 7
Donor3-ACCAGTATCCGCGGTA-2 3381 8473 6
Donor3-ACCAGTATCTGTCCGT-2 4280 13850 6

Donor3-ACCCACTAGGTGCTTT-2 4446 13376 2
Donor3-ACCCACTCAAACCTAC-2 4709 15736 2
Donor3-ACCCACTCAGTTCCCT-2 2257 4340 5
Donor3-ACCCACTGTCCAGTAT-2 6952 30636 3
Donor3-ACCCACTGTCTCACCT-2 3627 14621 6
Donor3-ACCCACTGTTCCATGA-2 1007 4302 8
Donor3-ACCCACTTCTCCTATA-2 3165 16638 7
Donor3-ACCGTAAAGGTGCTTT-2 1522 6424 8
Donor3-ACCGTAACATCTATGG-2 5778 23254 5
Donor3-ACCGTAAGTTGTCTTT-2 2302 8956 7
Donor3-ACCTTTACACTTAACG-2 4953 28139 5
Donor3-ACCTTTACAGATGGCA-2 3357 6921 3
Donor3-ACCTTTACATGAAGTA-2 1620 9030 8
Donor3-ACCTTTAGTGTTGAGG-2 3307 8728 6
Donor3-ACCTTTATCCTAGTGA-2 2295 4920 2
Donor3-ACCTTTATCGGCGCAT-2 2213 5621 10
Donor3-ACGAGCCCAATCACAC-2 2141 9483 7
Donor3-ACGAGCCGTATAGTAG-2 2976 7014 3
Donor3-ACGAGCCGTTTAGGAA-2 2387 10414 7
Donor3-ACGAGCCTCACGACTA-2 3708 8987 3
Donor3-ACGAGCCTCTTGCAAG-2 1495 6372 7
Donor3-ACGAGGACAGGGCATA-2 1919 9599 7
Donor3-ACGAGGATCACAAACC-2 4523 13437 2
Donor3-ACGATACAGATGCCAG-2 1399 6925 8
Donor3-ACGATGTAGGCTACGA-2 3968 11254 4
Donor3-ACGATGTTCCCAACGG-2 2178 18868 8
Donor3-ACGATGTTCTTCCTTC-2 1555 4602 8
Donor3-ACGCAGCCATGATCCA-2 1410 9243 8
Donor3-ACGCAGCGTTTGTTGG-2 1734 8521 7
Donor3-ACGCCAGTCTCGGACG-2 1368 7827 8
Donor3-ACGCCGAAGGTGATAT-2 2100 12544 8
Donor3-ACGCCGAAGTGTCCCG-2 1312 6072 8
Donor3-ACGCCGAGTAGAGGAA-2 3330 8422 5
Donor3-ACGCCGATCAGGATCT-2 2815 6170 3
Donor3-ACGCCGATCGCAAGCC-2 1827 4504 13
Donor3-ACGGAGAAGAATTGTG-2 3588 24219 7
Donor3-ACGGAGACATTTCAGG-2 1090 4900 8
Donor3-ACGGGCTAGATCGATA-2 2757 6155 3
Donor3-ACGGGCTCAATTGCTG-2 2020 15856 8
Donor3-ACGGGCTTCACCCTCA-2 1772 4353 10
Donor3-ACGGGTCAGGAGCGTT-2 1240 4601 7
Donor3-ACGGGTCAGTGCTGCC-2 2285 20772 8
Donor3-ACGGGTCGTAAGTGGC-2 1095 5258 8
Donor3-ACGGGTCGTTTACTCT-2 1043 5265 8
Donor3-ACGTCAAAGGTTCCTA-2 2596 6020 4
Donor3-ACGTCAAGTCGTGGCT-2 2831 13826 6
Donor3-ACTATCTGTCGCGAAA-2 3557 10542 2
Donor3-ACTGAACCACCTGGTG-2 3446 20020 7
Donor3-ACTGAGTCAAACTGTC-2 3304 19064 7
Donor3-ACTGAGTGTTTGTGTG-2 5878 20224 3
Donor3-ACTGATGCAGCGAACA-2 2808 13804 7
Donor3-ACTGATGTCGTACCGG-2 1763 5738 10
Donor3-ACTGCTCAGACAGAGA-2 1130 5539 8
Donor3-ACTGCTCCACACATGT-2 2226 14870 8
Donor3-ACTGTCCGTCGGATCC-2 1730 6190 7
Donor3-ACTGTCCTCTCGGACG-2 2791 5776 3

Donor3-ACTTACTCACGGTGTC-2 1411 6173 7
Donor3-ACTTACTGTTCAACCA-2 1999 5977 13
Donor3-ACTTGTTGTACAGTGG-2 1840 5512 6
Donor3-ACTTGTTTCACGGTTA-2 2074 8987 7
Donor3-ACTTGTTTCTCAACTT-2 4820 14750 6
Donor3-ACTTTCAAGGAGCGAG-2 1457 4546 13
Donor3-ACTTTCACACGAAGCA-2 3029 9748 4
Donor3-ACTTTCACAGCCAGAA-2 3729 22204 7
Donor3-ACTTTCATCAAAGACA-2 1012 5024 8
Donor3-AGAATAGAGATGTGGC-2 9031 64363 5
Donor3-AGAATAGCAGACAAAT-2 1615 5856 7
Donor3-AGACGTTCAGATAATG-2 3817 9929 3
Donor3-AGACGTTGTCCGAGTC-2 3107 24207 7
Donor3-AGACGTTTCAGTTGAC-2 1067 4621 8
Donor3-AGAGCTTCATCCGGGT-2 2144 5363 4
Donor3-AGAGCTTCATCTATGG-2 3359 7416 3
Donor3-AGAGCTTGTTACGACT-2 4243 12953 3
Donor3-AGAGTGGGTGGTACAG-2 1321 5185 9
Donor3-AGATCTGAGAGAACAG-2 2399 7389 6
Donor3-AGATCTGGTGCGAAAC-2 2483 11184 6
Donor3-AGATTGCGTCGAGATG-2 1668 9563 8
Donor3-AGATTGCGTCTTCGTC-2 3699 9844 1
Donor3-AGATTGCGTTTGCATG-2 2220 15610 8
Donor3-AGCAGCCCAGAGCCAA-2 1929 7133 9
Donor3-AGCAGCCGTCCGTCAG-2 2152 13560 7
Donor3-AGCAGCCTCCACTGGG-2 1547 8322 8
Donor3-AGCATACAGATAGGAG-2 7003 40938 2
Donor3-AGCATACAGATGTGGC-2 959 4151 8
Donor3-AGCCTAAAGGACACCA-2 2586 12589 7
Donor3-AGCCTAAGTAGCGATG-2 1806 7572 7
Donor3-AGCGGTCGTACCAGTT-2 2317 14474 7
Donor3-AGCGGTCTCATGCAAC-2 1246 4260 7
Donor3-AGCGTATCACAACGCC-2 1422 7839 8
Donor3-AGCTCCTAGATATGCA-2 1376 6593 8
Donor3-AGCTCCTGTGAGGCTA-2 2438 11537 7
Donor3-AGCTCTCGTAGAAAGG-2 3550 9014 3
Donor3-AGCTCTCTCGCATGAT-2 1570 9805 8
Donor3-AGCTTGAAGAAGGCCT-2 2497 11020 7
Donor3-AGCTTGAGTCGTGGCT-2 905 4327 8
Donor3-AGCTTGATCACAAACC-2 1401 6291 7
Donor3-AGCTTGATCTCGGACG-2 1235 6769 8
Donor3-AGGCCACAGATGAGAG-2 4508 20620 6
Donor3-AGGCCACCAGCTGTTA-2 2833 9679 10
Donor3-AGGCCACGTCGAGTTT-2 1268 4769 7
Donor3-AGGCCGTAGAGAACAG-2 1561 8558 7
Donor3-AGGCCGTAGTGGAGAA-2 3215 7094 3
Donor3-AGGCCGTTCCATGAAC-2 3643 9379 1
Donor3-AGGCCGTTCGAGAACG-2 2947 15092 7
Donor3-AGGGAGTCACGCTTTC-2 6480 38061 6
Donor3-AGGGAGTGTCTTGATG-2 1565 4171 6
Donor3-AGGGATGTCGTCGTTC-2 2694 12247 7
Donor3-AGGGTGACACCGATAT-2 6944 53598 5
Donor3-AGGGTGAGTCTGGTCG-2 4020 18990 6
Donor3-AGGTCATTCTCTTGAT-2 3855 10312 3
Donor3-AGGTCCGTCTGTGCAA-2 1819 5050 6
Donor3-AGGTCCGTCTTGTATC-2 1464 6985 7

Donor3-AGTCTTTGTCGTCTTC-2 7413 43000 5
Donor3-AGTGAGGCAGCATACT-2 1322 5816 7
Donor3-AGTGAGGTCACTCCTG-2 4253 14470 3
Donor3-AGTGAGGTCGTAGGTT-2 1818 8831 7
Donor3-AGTGGGACAATGGAAT-2 5118 16787 2
Donor3-AGTGGGACACGGTGTC-2 1324 6953 8
Donor3-AGTGGGAGTGGCAAAC-2 4196 22646 7
Donor3-AGTGGGATCCGCATAA-2 1421 8111 8
Donor3-AGTTGGTCATGCCTTC-2 3047 7384 3
Donor3-AGTTGGTGTCTGATTG-2 1982 4422 5
Donor3-ATAACGCTCCTTTACA-2 1951 4961 4
Donor3-ATAAGAGCAGATGGCA-2 4262 11237 5
Donor3-ATAGACCAGAAGGCCT-2 1639 9560 8
Donor3-ATAGACCAGATATGGT-2 2344 12796 7
Donor3-ATAGACCTCAGCAACT-2 1479 7653 8
Donor3-ATCACGAAGCAGCGTA-2 4718 13021 3
Donor3-ATCACGAAGTCGTACT-2 5952 30825 6
Donor3-ATCACGACAATCGGTT-2 4412 12214 6
Donor3-ATCATCTGTGTAATGA-2 1938 8321 7
Donor3-ATCATCTTCAACACCA-2 2540 9125 13
Donor3-ATCATGGAGAGCTTCT-2 8184 46821 5
Donor3-ATCATGGGTAAGGATT-2 5366 28275 5
Donor3-ATCATGGGTCTAAACC-2 5740 32041 6
Donor3-ATCATGGGTTTAGCTG-2 2884 16805 7
Donor3-ATCATGGTCGTTACAG-2 4540 13584 3
Donor3-ATCCACCGTAGGGACT-2 2329 10267 7
Donor3-ATCCACCTCAACACCA-2 3396 8328 6
Donor3-ATCCACCTCGCCTGTT-2 5071 16752 5
Donor3-ATCCGAAGTGCAACTT-2 3303 19532 7
Donor3-ATCGAGTAGCCACTAT-2 1849 12176 8
Donor3-ATCGAGTCAACTGCTA-2 2171 6355 13
Donor3-ATCGAGTTCGAGAACG-2 1203 4223 7
Donor3-ATCTACTCAATCTGCA-2 1761 8685 7
Donor3-ATCTACTCAGCTGCTG-2 2036 9048 7
Donor3-ATCTACTTCTCCAACC-2 4257 10171 2
Donor3-ATCTGCCAGTCACGCC-2 1587 8152 8
Donor3-ATCTGCCAGTGGAGAA-2 2220 12180 7
Donor3-ATCTGCCGTTGGTAAA-2 2223 9980 7
Donor3-ATGCGATAGACTCGGA-2 1911 4228 4
Donor3-ATGCGATAGCTCTCGG-2 1628 7605 8
Donor3-ATGCGATCAACAACCT-2 1947 4547 5
Donor3-ATGCGATCAACTGGCC-2 8211 57130 6
Donor3-ATGCGATCAGTATGCT-2 1762 4602 10
Donor3-ATGCGATGTCACCCAG-2 1705 4182 13
Donor3-ATGGGAGAGCAGGCTA-2 5862 24066 2
Donor3-ATGGGAGCAGCTCCGA-2 1159 5024 8
Donor3-ATGTGTGAGAGCTGGT-2 3652 8309 1
Donor3-ATGTGTGCATTGGCGC-2 3019 11636 10
Donor3-ATGTGTGTCCCTAACC-2 1895 5746 13
Donor3-ATTACTCAGCCCAGCT-2 4732 16137 4
Donor3-ATTACTCCACCAGGCT-2 3266 7988 5
Donor3-ATTATCCAGCGATGAC-2 3298 17151 7
Donor3-ATTCTACAGCCCAACC-2 5804 18405 2
Donor3-ATTCTACCAGGGTTAG-2 4140 17819 6
Donor3-ATTGGACCAACGCACC-2 3820 9924 1
Donor3-ATTGGACCAGGGAGAG-2 1613 4536 13

Donor3-ATTGGTGGTGTTCGAT-2 8820 76967 5
Donor3-ATTGGTGGTTATGCGT-2 2264 7916 8
Donor3-ATTTCTGAGCATGGCA-2 3110 18429 7
Donor3-ATTTCTGCACACTGCG-2 1363 8076 8
Donor3-ATTTCTGCAGCCAATT-2 1212 6250 8
Donor3-ATTTCTGGTACGACCC-2 2678 5541 3
Donor3-ATTTCTGTCCTGCAGG-2 4496 14054 1
Donor3-CAACCAAAGGCCCTTG-2 1191 5519 8
Donor3-CAACCAATCAGAGACG-2 3714 8590 4
Donor3-CAACCAATCGGACAAG-2 1994 6334 13
Donor3-CAACCTCCAGCAGTTT-2 2687 10047 6
Donor3-CAACCTCTCAGGTAAA-2 4748 14222 6
Donor3-CAACCTCTCTGTCAAG-2 1272 5263 7
Donor3-CAAGAAAGTGAGGCTA-2 1196 5541 8
Donor3-CAAGAAATCAGTTGAC-2 1404 4169 13
Donor3-CAAGATCCATTGGGCC-2 2535 5581 6
Donor3-CAAGATCGTGTTTGGT-2 3647 25179 7
Donor3-CAAGATCTCAACACGT-2 1166 5192 8
Donor3-CAAGGCCAGTGGTAGC-2 3100 12959 7
Donor3-CAAGGCCCAAGGTGTG-2 2089 6699 13
Donor3-CAAGGCCCATCGATTG-2 2194 9468 7
Donor3-CAAGGCCTCCCTCAGT-2 4783 14649 3
Donor3-CAAGGCCTCTAGAGTC-2 1443 5454 7
Donor3-CAAGTTGCACACGCTG-2 4778 14667 5
Donor3-CAAGTTGCAGCGTAAG-2 5390 18933 1
Donor3-CACAAACAGGGCTTGA-2 2883 13061 6
Donor3-CACACAACAACACGCC-2 2437 8056 6
Donor3-CACACCTCAAAGTGCG-2 2299 4455 3
Donor3-CACACCTGTTTCGCTC-2 1376 7433 8
Donor3-CACACCTTCTTGAGAC-2 2142 7173 9
Donor3-CACACTCAGAGTAAGG-2 3452 7642 3
Donor3-CACACTCAGGTGATAT-2 1983 9991 7
Donor3-CACACTCCAACTTGAC-2 8442 58168 6
Donor3-CACACTCGTATTACCG-2 2097 6059 13
Donor3-CACACTCTCGAATGGG-2 6022 25474 1
Donor3-CACAGGCAGGGTGTGT-2 1663 9315 8
Donor3-CACAGGCCATTTGCCC-2 1778 8430 8
Donor3-CACAGGCTCTTTACAC-2 2616 5545 6
Donor3-CACAGTAGTTCCACAA-2 1204 4690 7
Donor3-CACAGTAGTTCCCTTG-2 3755 22639 7
Donor3-CACATAGAGTGACTCT-2 2698 8204 13
Donor3-CACATAGGTGCCTGTG-2 1498 6749 7
Donor3-CACATTTAGAAGGGTA-2 1000 4161 8
Donor3-CACATTTCACTTAAGC-2 2010 4694 4
Donor3-CACATTTTCTAACTCT-2 1972 6358 12
Donor3-CACATTTTCTAAGCCA-2 2327 5787 4
Donor3-CACCACTCACTAGTAC-2 1931 6752 9
Donor3-CACCACTCATCTACGA-2 1037 5622 8
Donor3-CACCACTCATTATCTC-2 6904 30175 1
Donor3-CACCACTGTATGAATG-2 3317 7402 3
Donor3-CACCTTGTCGGACAAG-2 3203 7177 3
Donor3-CACTCCACAAGTCTAC-2 1271 4431 13
Donor3-CACTCCACATAAAGGT-2 3815 8862 5
Donor3-CACTCCAGTATATGGA-2 1717 5587 9
Donor3-CACTCCAGTCATACTG-2 2111 8429 8
Donor3-CACTCCATCCTGTACC-2 1366 6754 8

Donor3-CAGAATCCAAGTCTGT-2 1331 4436 9
Donor3-CAGAATCGTTTGTGTG-2 3798 17682 6
Donor3-CAGAATCTCTTCAACT-2 3682 9166 4
Donor3-CAGAGAGCACGGTAAG-2 2963 6674 3
Donor3-CAGAGAGGTGAGCGAT-2 4340 15044 5
Donor3-CAGAGAGTCAGCATGT-2 2609 6959 4
Donor3-CAGAGAGTCGGACAAG-2 1831 7741 7
Donor3-CAGATCAGTAATCGTC-2 2332 4912 3
Donor3-CAGATCATCAGCATGT-2 1999 6085 13
Donor3-CAGCAGCGTCTTTCAT-2 2285 4897 2
Donor3-CAGCATAGTAGATTAG-2 1384 7287 8
Donor3-CAGCATAGTCTTGATG-2 2513 5275 3
Donor3-CAGCCGAAGACTACAA-2 2219 12069 7
Donor3-CAGCCGAAGTCTTGCA-2 990 4144 8
Donor3-CAGCCGAAGTGTACCT-2 4319 19636 6
Donor3-CAGCCGAGTCGAGATG-2 1210 7245 8
Donor3-CAGCCGAGTGGCCCTA-2 1866 6294 6
Donor3-CAGCGACCACTACAGT-2 1654 4756 13
Donor3-CAGCTAAAGATCCCAT-2 1490 8575 8
Donor3-CAGCTAACAAGGACTG-2 7170 62461 5
Donor3-CAGCTGGCAATCACAC-2 3221 6995 3
Donor3-CAGCTGGCACATTAGC-2 1846 5030 13
Donor3-CAGCTGGTCACGACTA-2 3461 8677 3
Donor3-CAGCTGGTCAGTTAGC-2 5378 17269 2
Donor3-CAGTAACAGAGTAATC-2 3567 26313 7
Donor3-CAGTAACCAGCCTTGG-2 1778 5612 9
Donor3-CAGTAACTCGCAAACT-2 1890 12244 8
Donor3-CAGTAACTCTTGAGAC-2 4250 11475 6
Donor3-CAGTCCTAGACAGAGA-2 3554 8232 3
Donor3-CAGTCCTCACCAACCG-2 3243 8097 4
Donor3-CAGTCCTGTCCTCCAT-2 1791 4470 13
Donor3-CATATGGCAACTGCTA-2 1062 4503 8
Donor3-CATATTCGTCTCGTTC-2 2926 11514 6
Donor3-CATATTCGTGACTACT-2 5113 16464 5
Donor3-CATATTCTCTAGAGTC-2 1376 5620 8
Donor3-CATCAAGAGGCATGTG-2 6727 29113 5
Donor3-CATCAAGCAACACCCG-2 1937 8762 7
Donor3-CATCAAGGTCAACATC-2 2022 4350 5
Donor3-CATCAGACAGGGTTAG-2 3101 19132 7
Donor3-CATCAGAGTAACGCGA-2 1219 4950 7
Donor3-CATCAGAGTTAAGATG-2 1637 7012 7
Donor3-CATCCACCAGCGTTCG-2 1063 4655 8
Donor3-CATCCACCAGGACCCT-2 3792 12918 6
Donor3-CATCGAACACGTCTCT-2 8145 80806 4
Donor3-CATCGGGAGCCCTAAT-2 2496 5797 3
Donor3-CATCGGGCATGTTGAC-2 3667 9386 2
Donor3-CATGACACAGGTGCCT-2 4558 19686 4
Donor3-CATGACATCTCACATT-2 1518 6934 8
Donor3-CATGCCTAGGACACCA-2 1899 4934 13
Donor3-CATGCCTAGGATTCGG-2 1718 7021 7
Donor3-CATGCCTCAAGCGCTC-2 1253 6435 8
Donor3-CATGCCTTCAGTTGAC-2 1462 7205 8
Donor3-CATTATCCATGTTGAC-2 1816 6848 7
Donor3-CATTATCTCAGTTAGC-2 2513 4589 5
Donor3-CATTCGCTCCAGGGCT-2 1758 6257 9
Donor3-CATTCGCTCCTATGTT-2 3904 9669 3

Donor3-CATTCGCTCTGCCCTA-2 5262 17841 2
Donor3-CCAATCCCACGAGGTA-2 3325 8066 4
Donor3-CCAATCCGTTTGACTG-2 1102 5547 8
Donor3-CCAATCCTCAGTGTTG-2 3266 12915 10
Donor3-CCACCTAAGAACAACT-2 1714 9622 8
Donor3-CCACCTACAGTCCTTC-2 1335 5967 7
Donor3-CCACGGATCCGAGCCA-2 1360 4572 9
Donor3-CCACTACGTGAGGGTT-2 6500 29067 2
Donor3-CCAGCGAAGAGGGATA-2 1198 4732 7
Donor3-CCAGCGAAGATCTGAA-2 1603 4722 13
Donor3-CCAGCGAGTTCATGGT-2 2070 7805 9
Donor3-CCATGTCAGGATATAC-2 4587 20027 6
Donor3-CCATGTCGTAATCACC-2 1811 4215 4
Donor3-CCATGTCGTCCGTGAC-2 1740 5268 13
Donor3-CCATTCGAGGCATTGG-2 1484 4213 7
Donor3-CCCAATCAGAATGTGT-2 1776 6577 7
Donor3-CCCAATCAGCCAGTTT-2 1403 5339 8
Donor3-CCCAATCGTTGAGGTG-2 5148 19105 1
Donor3-CCCAGTTAGAAACGCC-2 3290 11459 11
Donor3-CCCAGTTAGCTGTTCA-2 1125 5689 8
Donor3-CCCAGTTAGGGCATGT-2 1219 5837 8
Donor3-CCCAGTTAGTAGTGCG-2 1163 4829 7
Donor3-CCCAGTTTCTCACATT-2 6986 36404 5
Donor3-CCGGGATAGCGTAATA-2 3217 17138 7
Donor3-CCGGGATAGTACGACG-2 3408 26458 7
Donor3-CCGGGATGTGATGTCT-2 1862 7035 13
Donor3-CCGGTAGAGATGGGTC-2 1178 4567 8
Donor3-CCGGTAGAGGAGTTGC-2 5168 17542 1
Donor3-CCGGTAGAGGATATAC-2 5749 26981 4
Donor3-CCGGTAGGTCAAACTC-2 3387 12178 6
Donor3-CCGGTAGTCACATGCA-2 2583 7123 4
Donor3-CCGGTAGTCTGTACGA-2 1032 4112 8
Donor3-CCGTGGAAGATGAGAG-2 3408 7779 3
Donor3-CCGTGGAAGCTAGTGG-2 1483 8736 8
Donor3-CCTAAAGAGTACGATA-2 4776 15231 4
Donor3-CCTAAAGCATGTAGTC-2 1194 5400 8
Donor3-CCTAAAGCATTGCGGC-2 4674 14302 6
Donor3-CCTAAAGGTAAAGTCA-2 1636 5450 13
Donor3-CCTAAAGTCATGGTCA-2 1503 5109 9
Donor3-CCTACACGTATCAGTC-2 3172 16821 7
Donor3-CCTACACGTTGATTCG-2 1416 5974 7
Donor3-CCTACACTCGATGAGG-2 1485 5935 8
Donor3-CCTACCAAGATCTGCT-2 6736 41522 6
Donor3-CCTACCAAGCTAAGAT-2 1313 6500 8
Donor3-CCTACCAGTTCACCTC-2 7487 39966 2
Donor3-CCTACCATCCCATTTA-2 6645 32072 6
Donor3-CCTAGCTAGCTCAACT-2 1310 9034 8
Donor3-CCTATTATCTACTATC-2 3274 7213 3
Donor3-CCTATTATCTTTACAC-2 1705 11353 8
Donor3-CCTCAGTTCGAGAACG-2 3525 8668 3
Donor3-CCTCTGAAGACTGGGT-2 1596 6131 7
Donor3-CCTCTGACAATGGAAT-2 1004 4619 8
Donor3-CCTCTGACAGTCTTCC-2 2023 13473 8
Donor3-CCTCTGAGTGTTTGGT-2 1908 6281 7
Donor3-CCTCTGATCAAGATCC-2 4322 11784 2
Donor3-CCTCTGATCCTATTCA-2 2241 9688 9

Donor3-CCTCTGATCCTTGACC-2 1403 6010 7
Donor3-CCTTACGCACAACGCC-2 1471 6572 8
Donor3-CCTTACGCACGTCAGC-2 1087 4610 8
Donor3-CCTTCCCAGTACGTAA-2 2733 5874 3
Donor3-CCTTCCCCAGGATCGA-2 1367 4737 7
Donor3-CCTTCCCGTCTCAACA-2 1329 4346 7
Donor3-CCTTCCCGTTCGCGAC-2 1746 7458 7
Donor3-CCTTCGACACCCATTC-2 7378 55982 4
Donor3-CCTTCGAGTAGCTGCC-2 5333 21338 5
Donor3-CCTTTCTAGTGTTGAA-2 4623 11953 1
Donor3-CCTTTCTCACGGTGTC-2 1379 7494 8
Donor3-CCTTTCTGTTACTGAC-2 1598 7534 7
Donor3-CGAACATAGCGTTCCG-2 1506 10363 8
Donor3-CGAACATGTACCAGTT-2 1020 5396 8
Donor3-CGAACATGTAGCGTCC-2 1821 12331 8
Donor3-CGAACATGTCACTTCC-2 1537 4215 10
Donor3-CGAATGTAGCAACGGT-2 1781 6463 10
Donor3-CGAATGTCACCTATCC-2 2313 11036 7
Donor3-CGAATGTTCATCTGCC-2 4848 14931 5
Donor3-CGACCTTAGGCACATG-2 3844 9846 4
Donor3-CGACCTTGTTTCCACC-2 1600 4510 13
Donor3-CGACTTCCACAACGTT-2 2632 14977 7
Donor3-CGACTTCGTAAGGGCT-2 5886 24945 4
Donor3-CGACTTCGTACAGACG-2 1221 4116 7
Donor3-CGACTTCGTTACGTCA-2 1672 4645 13
Donor3-CGACTTCTCATAGCAC-2 2072 9211 7
Donor3-CGAGAAGAGACCTAGG-2 1683 7952 7
Donor3-CGAGAAGCATACTACG-2 6343 42518 6
Donor3-CGAGCACTCCGCGGTA-2 2617 5567 5
Donor3-CGAGCCAGTTTCGCTC-2 4500 11880 2
Donor3-CGATCGGAGAATGTGT-2 1761 11153 8
Donor3-CGATGGCCAATTCCTT-2 3692 17913 6
Donor3-CGATGGCCATCTCGCT-2 4047 9501 1
Donor3-CGATGGCCATTGGTAC-2 3820 9862 3
Donor3-CGATGGCTCTCAACTT-2 6105 38513 6
Donor3-CGATGTAGTAAGTTCC-2 3004 6951 3
Donor3-CGATTGATCAGAGGTG-2 2260 13615 7
Donor3-CGATTGATCCGCATCT-2 1792 4146 6
Donor3-CGCCAAGTCAGTTCGA-2 2363 4768 5
Donor3-CGCGGTAAGGTACTCT-2 3763 8820 1
Donor3-CGCGGTACACAGTCGC-2 3516 9221 2
Donor3-CGCTATCAGGTGCTAG-2 4629 14146 5
Donor3-CGCTATCTCAGATAAG-2 6925 33937 6
Donor3-CGCTATCTCTTTAGTC-2 1043 4734 8
Donor3-CGCTGGAAGCCTCGTG-2 1952 5329 10
Donor3-CGCTGGAGTACCGTTA-2 3098 19211 7
Donor3-CGCTGGAGTTAAGAAC-2 3363 8938 1
Donor3-CGCTTCAAGACCCACC-2 1331 6821 8
Donor3-CGCTTCAAGCGCTTAT-2 1385 5593 7
Donor3-CGCTTCAGTAAGAGGA-2 5801 33650 6
Donor3-CGGACACAGCAATCTC-2 2935 6660 3
Donor3-CGGACACTCACCCTCA-2 5716 36813 6
Donor3-CGGACACTCGTAGATC-2 3261 18301 7
Donor3-CGGACGTAGATAGTCA-2 4493 19963 6
Donor3-CGGACGTCAATGGTCT-2 2217 8953 7
Donor3-CGGACGTGTCCCGACA-2 3599 20685 7

Donor3-CGGACGTGTCTCGTTC-2 2488 9388 10
Donor3-CGGACGTTCACATAGC-2 679 4823 6
Donor3-CGGACTGCACTTAACG-2 931 4427 8
Donor3-CGGACTGGTAAGTAGT-2 2578 8056 4
Donor3-CGGACTGGTGTTTGGT-2 1865 6282 13
Donor3-CGGACTGTCTTACCGC-2 1685 8254 7
Donor3-CGGAGCTGTACAGTGG-2 2196 4472 2
Donor3-CGGAGCTTCGTAGGTT-2 4953 16105 6
Donor3-CGGAGTCAGTGTCCAT-2 3557 15925 10
Donor3-CGGAGTCTCTGAAAGA-2 1438 6456 8
Donor3-CGGCTAGCAGACGCCT-2 5481 18005 2
Donor3-CGGCTAGGTAGTACCT-2 6265 34095 6
Donor3-CGGCTAGTCGGTTCGG-2 4410 12540 2
Donor3-CGGGTCAAGCTGAACG-2 3803 9187 6
Donor3-CGGGTCACAATGGAAT-2 3776 9634 5
Donor3-CGGGTCAGTCTCTTAT-2 3195 7332 3
Donor3-CGGGTCATCAGGCAAG-2 854 6145 8
Donor3-CGGGTCATCATTGCGA-2 4683 22248 10
Donor3-CGGTTAAAGCCACGCT-2 5253 24674 5
Donor3-CGGTTAAAGTAGCCGA-2 1391 7192 8
Donor3-CGGTTAAGTGGCTCCA-2 4745 19341 6
Donor3-CGGTTAATCCTAGGGC-2 5426 18586 1
Donor3-CGTAGCGCAAAGCGGT-2 1035 4429 8
Donor3-CGTAGCGCACATAACC-2 5837 25974 6
Donor3-CGTAGCGCATAGACTC-2 1724 5521 8
Donor3-CGTAGCGCATCAGTCA-2 1968 7935 7
Donor3-CGTAGGCGTCCCTTGT-2 7453 40589 5
Donor3-CGTAGGCGTGAGGGTT-2 1697 4852 13
Donor3-CGTAGGCGTGTTGGGA-2 3981 13978 5
Donor3-CGTCCATAGAGCCCAA-2 1386 5996 8
Donor3-CGTCCATAGTAGCCGA-2 2506 5209 11
Donor3-CGTCCATGTCAGCTAT-2 2383 8784 13
Donor3-CGTCCATGTTAAGTAG-2 2872 15870 7
Donor3-CGTCTACCAAGAAGAG-2 1971 5141 10
Donor3-CGTCTACCAAGACACG-2 3531 8786 3
Donor3-CGTCTACTCTGATACG-2 3788 10007 4
Donor3-CGTGAGCAGACGACGT-2 1456 8511 8
Donor3-CGTGAGCAGGACGAAA-2 4935 17160 6
Donor3-CGTGAGCGTCTGCAAT-2 5022 23383 6
Donor3-CGTGTCTAGGCAAAGA-2 1416 5768 8
Donor3-CGTTAGAAGCGTCAAG-2 4369 23965 6
Donor3-CGTTAGAAGGAGTTTA-2 3427 8373 3
Donor3-CGTTAGACAAGTAGTA-2 3114 7146 3
Donor3-CGTTAGAGTCGCATAT-2 1844 7990 7
Donor3-CGTTAGATCCCAGGTG-2 3298 9404 6
Donor3-CGTTAGATCCTAGGGC-2 1997 12305 7
Donor3-CGTTAGATCTGTTTGT-2 2138 12867 7
Donor3-CGTTCTGGTCGGATCC-2 2966 17164 7
Donor3-CGTTGGGCAGCCTATA-2 5034 15961 3
Donor3-CGTTGGGTCCGTTGCT-2 4171 11600 5
Donor3-CTAAGACCAGGGAGAG-2 3273 7386 3
Donor3-CTAAGACGTAAACACA-2 1041 5519 8
Donor3-CTAAGACTCGAGAACG-2 2506 8598 13
Donor3-CTAAGACTCTACCAGA-2 2341 7348 8
Donor3-CTAAGACTCTGAAAGA-2 2034 6399 13
Donor3-CTAATGGCAGTTCATG-2 3177 6741 3

Donor3-CTAATGGTCTTCAACT-2 1251 4376 7
Donor3-CTACACCGTACTTGAC-2 1304 4876 8
Donor3-CTACACCGTCAGAATA-2 2469 11781 7
Donor3-CTACACCTCATCGGAT-2 4223 13062 5
Donor3-CTACATTAGAAACCTA-2 5319 16933 1
Donor3-CTACATTAGTTTCCTT-2 3643 9323 2
Donor3-CTACCCAGTTGAGGTG-2 2213 8074 6
Donor3-CTACCCAGTTGGAGGT-2 3587 9106 3
Donor3-CTACGTCAGTACACCT-2 2202 12608 7
Donor3-CTACGTCCAAACGTGG-2 4439 35214 7
Donor3-CTACGTCCAAGCTGAG-2 2016 5483 13
Donor3-CTACGTCCATACTCTT-2 1357 6354 8
Donor3-CTAGAGTAGATCGGGT-2 1819 11282 8
Donor3-CTAGAGTCATGTAAGA-2 2223 9611 7
Donor3-CTAGAGTGTTTAGCTG-2 4923 14977 1
Donor3-CTAGAGTTCCGCATCT-2 5716 19872 5
Donor3-CTAGCCTAGCAAATCA-2 2603 5788 1
Donor3-CTAGCCTAGGCTCAGA-2 1846 4270 4
Donor3-CTAGCCTCAATGCCAT-2 2226 7219 13
Donor3-CTAGCCTTCAGTACGT-2 6674 29373 1
Donor3-CTAGTGAAGCCAACAG-2 1913 5718 13
Donor3-CTAGTGAAGGGCTCTC-2 5240 22367 6
Donor3-CTAGTGAAGTCAAGGC-2 3978 14905 6
Donor3-CTAGTGAGTAGCGATG-2 1833 6078 13
Donor3-CTAGTGAGTTATCCGA-2 1759 9547 8
Donor3-CTAGTGAGTTGATTCG-2 2961 7061 5
Donor3-CTCACACAGTACACCT-2 1154 5383 8
Donor3-CTCACACCACACATGT-2 2087 6449 9
Donor3-CTCACACCAGTCAGCC-2 1019 4413 8
Donor3-CTCACACTCCACTGGG-2 1100 4626 8
Donor3-CTCACACTCTCTGAGA-2 2449 15275 7
Donor3-CTCAGAAAGAGGTTGC-2 3266 7992 3
Donor3-CTCAGAACAGACAAGC-2 1518 7618 8
Donor3-CTCATTAGTCATGCCG-2 1300 5473 7
Donor3-CTCCTAGGTTACCGAT-2 2331 8837 9
Donor3-CTCGAAAAGTCTTGCA-2 2488 4427 5
Donor3-CTCGAAACATGCGCAC-2 2659 5853 3
Donor3-CTCGAAACATTTCAGG-2 1486 4853 9
Donor3-CTCGAAATCGACGGAA-2 4372 24425 6
Donor3-CTCGAAATCTACTTAC-2 4649 12052 2
Donor3-CTCGAGGTCAGAAATG-2 5001 15477 5
Donor3-CTCGGAGAGTTAGGTA-2 1020 4719 8
Donor3-CTCGGAGGTACACCGC-2 2453 6919 6
Donor3-CTCGGAGGTCCAACTA-2 2088 7035 6
Donor3-CTCGGGATCGGATGGA-2 1617 6135 7
Donor3-CTCGGGATCTAACTTC-2 3553 8678 3
Donor3-CTCGTACAGCCAGTAG-2 5412 17290 1
Donor3-CTCGTCAAGCAACGGT-2 1151 6048 8
Donor3-CTCGTCAGTACAAGTA-2 5431 17270 1
Donor3-CTCTGGTGTCTCCACT-2 1587 9711 8
Donor3-CTGAAGTGTCCGTGAC-2 3574 15856 6
Donor3-CTGATAGCATGTTCCC-2 2986 6499 2
Donor3-CTGATAGGTATATGAG-2 1236 6322 8
Donor3-CTGATAGTCCGGGTGT-2 1567 9257 8
Donor3-CTGATAGTCCTATTCA-2 1079 4416 8
Donor3-CTGATCCAGTTCGCGC-2 2213 7034 13

Donor3-CTGATCCCAAAGCGGT-2 7342 40222 6
Donor3-CTGATCCCACAACTGT-2 1897 4145 6
Donor3-CTGATCCGTACCGGCT-2 3827 9830 3
Donor3-CTGATCCTCCCATTTA-2 1099 4675 7
Donor3-CTGCCTAAGCGTTCCG-2 2022 5815 13
Donor3-CTGCCTATCCAAATGC-2 1629 6990 8
Donor3-CTGCTGTGTAAGAGGA-2 5598 23919 5
Donor3-CTGCTGTGTAGTAGTA-2 6044 23494 3
Donor3-CTGCTGTTCGCAGGCT-2 4759 14942 1
Donor3-CTGCTGTTCTTAGAGC-2 5359 28395 6
Donor3-CTGGTCTAGAGCTATA-2 1635 6053 7
Donor3-CTGGTCTAGATCCCGC-2 1293 4466 12
Donor3-CTGGTCTCACACATGT-2 1730 8103 8
Donor3-CTGGTCTCAGGTCCAC-2 1258 4887 8
Donor3-CTGGTCTTCAAACAAG-2 1587 9315 8
Donor3-CTGTGCTAGCACCGCT-2 5568 31526 4
Donor3-CTGTGCTCAGGGCATA-2 3461 9743 3
Donor3-CTGTTTACAAGGTTCT-2 1671 4858 6
Donor3-CTGTTTAGTAGAGTGC-2 2108 6831 9
Donor3-CTGTTTATCGCGCCAA-2 1885 7860 7
Donor3-CTTAACTCAGGACCCT-2 1603 4526 10
Donor3-CTTACCGCACGAGAGT-2 4268 26238 7
Donor3-CTTACCGCATTACCTT-2 5049 15638 2
Donor3-CTTACCGGTCCGACGT-2 2272 5910 5
Donor3-CTTACCGTCACCACCT-2 1002 4679 8
Donor3-CTTACCGTCACTATTC-2 1201 5459 8
Donor3-CTTACCGTCAGTGTTG-2 4676 14939 2
Donor3-CTTACCGTCTATGTGG-2 2833 17459 7
Donor3-CTTAGGAAGTCCCACG-2 1611 4246 6
Donor3-CTTCTCTAGACCCACC-2 3356 8498 6
Donor3-CTTCTCTCAGCGTCCA-2 3804 9188 3
Donor3-CTTCTCTCATGTAAGA-2 3238 11089 7
Donor3-CTTCTCTGTCGGCACT-2 1653 6501 7
Donor3-CTTCTCTGTTTGCATG-2 2045 8379 7
Donor3-CTTCTCTTCGTCCGTT-2 1436 7633 8
Donor3-CTTCTCTTCTGATTCT-2 2466 5974 6
Donor3-CTTTGCGAGCTTTGGT-2 3645 9033 3
Donor3-CTTTGCGCAGTTCATG-2 1015 4586 8
Donor3-CTTTGCGCATTATCTC-2 2010 8986 7
Donor3-GAAATGAGTCGGCTCA-2 2090 7579 13
Donor3-GAAATGATCACTGGGC-2 1303 4531 13
Donor3-GAACATCCAGACGTAG-2 6460 35394 5
Donor3-GAACATCGTTCCGGCA-2 4754 15554 3
Donor3-GAACCTAGTTTAAGCC-2 1049 5358 8
Donor3-GAACGGAAGACTCGGA-2 2951 6600 6
Donor3-GAACGGAAGGCTCATT-2 1531 6165 8
Donor3-GAACGGATCCTACAGA-2 1504 4492 13
Donor3-GAACGGATCGAGGTAG-2 1500 4256 13
Donor3-GAACGGATCTTACCTA-2 8816 59489 6
Donor3-GAAGCAGGTCCAAGTT-2 1084 5385 8
Donor3-GAAGCAGTCAGGCCCA-2 6080 26768 5
Donor3-GAAGCAGTCGGCTTGG-2 4076 27260 7
Donor3-GAATAAGAGCGCTTAT-2 3298 16931 7
Donor3-GAATAAGCATGGTCAT-2 4076 10993 1
Donor3-GACACGCTCCGAACGC-2 3588 8893 3
Donor3-GACAGAGCATAGACTC-2 1106 4332 7

Donor3-GACAGAGTCACAATGC-2 2022 4266 4
Donor3-GACCAATAGGGTATCG-2 1499 5646 8
Donor3-GACCAATCAAATACAG-2 3567 8533 3
Donor3-GACCAATCAAGAAAGG-2 1865 14603 8
Donor3-GACCAATCACAGACAG-2 1957 5009 10
Donor3-GACCAATCAGTATAAG-2 2755 10853 6
Donor3-GACGCGTAGAAAGTGG-2 1627 4110 13
Donor3-GACGCGTGTTCTCATT-2 4048 10810 6
Donor3-GACGCGTTCTTTACGT-2 2703 11880 6
Donor3-GACGGCTTCACGATGT-2 6623 40829 6
Donor3-GACGGCTTCGAATCCA-2 3261 17327 7
Donor3-GACGTGCAGACTGGGT-2 3735 7861 6
Donor3-GACGTGCCATTTGCTT-2 2835 13308 7
Donor3-GACGTGCTCTGCTGCT-2 2844 4973 5
Donor3-GACGTTAAGCCACCTG-2 3522 8599 4
Donor3-GACGTTACACAGGCCT-2 2336 7873 6
Donor3-GACTAACGTACAAGTA-2 2072 6107 10
Donor3-GACTAACGTATATGGA-2 1881 6230 9
Donor3-GACTACACATGTAGTC-2 2090 9618 7
Donor3-GACTACAGTCCATGAT-2 4470 13001 4
Donor3-GACTGCGAGACTGTAA-2 1715 6892 7
Donor3-GACTGCGGTTTCGCTC-2 961 4527 8
Donor3-GAGCAGACAGGCGATA-2 3434 7877 3
Donor3-GAGCAGACAGTCAGCC-2 2642 5547 3
Donor3-GAGGTGACAAAGAATC-2 2071 8128 9
Donor3-GAGGTGATCAACACCA-2 3653 9882 6
Donor3-GAGGTGATCCGTAGTA-2 1390 8616 8
Donor3-GAGTCCGAGGCTATCT-2 2642 5766 5
Donor3-GAGTCCGGTTCCCGAG-2 2216 6834 9
Donor3-GAGTCCGTCCCACTTG-2 1945 5268 13
Donor3-GAGTCCGTCCGCGGTA-2 1710 6478 9
Donor3-GATCAGTAGATGTGGC-2 1876 6877 9
Donor3-GATCAGTCAACTGGCC-2 1384 4724 9
Donor3-GATCGATGTTCCACGG-2 2569 13083 7
Donor3-GATCGATTCAAGATCC-2 3403 7987 3
Donor3-GATCGATTCTGAGTGT-2 2083 8963 7
Donor3-GATCGCGGTGCAGGTA-2 1571 5474 7
Donor3-GATCGTACAGCCAATT-2 7307 36588 5
Donor3-GATCGTACAGCTTCGG-2 2964 6564 3
Donor3-GATCGTATCAATAAGG-2 6308 26814 4
Donor3-GATCTAGAGTACGTTC-2 7089 37247 5
Donor3-GATCTAGTCGCTGATA-2 1282 8092 8
Donor3-GATGAGGAGCAATATG-2 4145 9988 5
Donor3-GATGCTACACATCTTT-2 1041 4322 8
Donor3-GATGCTAGTAAATACG-2 2405 10057 7
Donor3-GATTCAGCAAGAAGAG-2 7157 30023 1
Donor3-GATTCAGCACAGATTC-2 4717 14116 5
Donor3-GCAAACTCAATCCAAC-2 4268 16557 6
Donor3-GCAAACTTCACAGGCC-2 1374 4384 13
Donor3-GCAAACTTCCGTTGCT-2 2143 12171 8
Donor3-GCAAACTTCGTTACGA-2 4471 12764 4
Donor3-GCAATCAAGATGCCAG-2 1755 10120 8
Donor3-GCAATCAAGTGACTCT-2 3995 10165 3
Donor3-GCAATCACATTAACCG-2 1543 6100 9
Donor3-GCAATCATCAGTTAGC-2 5314 34702 6
Donor3-GCAATCATCGTCTGCT-2 4827 21368 6

Donor3-GCACATAAGTGGTAAT-2 3663 14074 6
Donor3-GCACATACAGACGTAG-2 4987 16596 6
Donor3-GCACTCTAGCCACGCT-2 2548 11295 7
Donor3-GCACTCTAGCGAAGGG-2 2335 12933 7
Donor3-GCACTCTAGGTGATAT-2 6336 45414 5
Donor3-GCAGCCAAGAACTCGG-2 3211 7431 3
Donor3-GCAGCCATCACCTTAT-2 1587 5302 6
Donor3-GCAGTTATCGAATGCT-2 2223 7394 6
Donor3-GCAGTTATCGCTTAGA-2 3499 8583 3
Donor3-GCATACAAGAATTCCC-2 4988 16284 2
Donor3-GCATACAGTAAGAGGA-2 1275 5207 7
Donor3-GCATACAGTAGCACGA-2 1686 10105 8
Donor3-GCATGATAGTCCTCCT-2 4210 10918 3
Donor3-GCATGATCAAAGGTGC-2 3067 7505 6
Donor3-GCATGATCATTGGTAC-2 5335 32073 6
Donor3-GCATGCGAGAACTGTA-2 1574 10486 8
Donor3-GCATGCGCAAGAGTCG-2 3118 7343 3
Donor3-GCATGCGTCGTGACAT-2 1464 7456 8
Donor3-GCATGTAGTCTTCGTC-2 4529 12255 1
Donor3-GCATGTAGTTCGTCTC-2 6701 24895 2
Donor3-GCATGTATCAGCAACT-2 1830 8178 7
Donor3-GCCAAATCAACTGGCC-2 1189 5454 8
Donor3-GCCAAATTCAGTACGT-2 1737 4887 13
Donor3-GCCAAATTCTTTACGT-2 3973 14341 5
Donor3-GCCTCTAAGGTGTGGT-2 3000 6230 6
Donor3-GCCTCTAGTTAGTGGG-2 6278 23051 1
Donor3-GCCTCTAGTTCAGGCC-2 1548 5947 7
Donor3-GCCTCTATCCCATTAT-2 5479 22621 5
Donor3-GCCTCTATCTTCAACT-2 1573 8839 8
Donor3-GCGACCAAGCAGCGTA-2 1073 4519 8
Donor3-GCGACCAGTTCGGCAC-2 1245 6755 8
Donor3-GCGAGAACATCACCCT-2 2289 19566 8
Donor3-GCGAGAAGTCTAGTGT-2 4916 16076 1
Donor3-GCGCAACCAAACCTAC-2 3901 19094 10
Donor3-GCGCAACCACGGCTAC-2 2486 4968 3
Donor3-GCGCAACGTGACTCAT-2 5165 21867 5
Donor3-GCGCAGTAGCCGATTT-2 1373 7299 8
Donor3-GCGCAGTGTCTCCACT-2 3360 7043 2
Donor3-GCGCAGTGTGATAAGT-2 1557 7546 7
Donor3-GCGCCAACACCAGCAC-2 6129 26366 4
Donor3-GCGCCAATCCATTCTA-2 4800 14342 5
Donor3-GCGCGATAGATATGCA-2 1258 6183 8
Donor3-GCGCGATGTTCCACTC-2 3863 10570 3
Donor3-GCGCGATTCTACGAGT-2 1221 7296 8
Donor3-GCTCCTAAGGGTATCG-2 3836 8876 6
Donor3-GCTCCTATCAAGCCTA-2 4779 14896 4
Donor3-GCTCTGTTCCTTGCCA-2 3203 8300 3
Donor3-GCTGCAGAGCCGCCTA-2 1423 6711 8
Donor3-GCTGCAGTCAACACCA-2 1723 8319 8
Donor3-GCTGCGACAGCGAACA-2 2059 9911 7
Donor3-GCTGCTTAGATCCGAG-2 3116 6785 3
Donor3-GCTGCTTTCGAACGGA-2 5715 24556 6
Donor3-GCTGGGTAGCGATTCT-2 3617 9227 3
Donor3-GCTTCCAAGAATTCCC-2 2115 6042 10
Donor3-GCTTCCAAGGTGATTA-2 4018 20668 6
Donor3-GCTTGAACAAGCCGCT-2 2141 7696 9

Donor3-GCTTGAACATGCAATC-2 2213 19033 8
Donor3-GCTTGAATCGTCGTTC-2 1361 6099 7
Donor3-GGAAAGCAGCTTTGGT-2 3510 19113 6
Donor3-GGAAAGCAGTGTCCAT-2 4328 12364 2
Donor3-GGAAAGCGTGAAGGCT-2 1311 6453 8
Donor3-GGAAAGCTCCACGACG-2 4887 20953 6
Donor3-GGAAAGCTCCTTAATC-2 2925 11581 6
Donor3-GGAACTTCATCACAAC-2 1989 8435 7
Donor3-GGAACTTGTAATTGGA-2 1150 4343 7
Donor3-GGAACTTGTACATGTC-2 1702 4925 7
Donor3-GGAACTTTCAGTCAGT-2 3443 18941 7
Donor3-GGAACTTTCGCGTTTC-2 2018 5363 11
Donor3-GGACAAGCACCGAAAG-2 1430 4175 13
Donor3-GGACAAGCATCGACGC-2 1377 8468 8
Donor3-GGACAAGTCTGCTTGC-2 1915 11593 7
Donor3-GGACAGACAAGCGATG-2 7263 45203 5
Donor3-GGACAGATCCGCGGTA-2 1731 5825 13
Donor3-GGACATTCAGTAAGAT-2 3440 7840 3
Donor3-GGACATTTCAACGGCC-2 2163 5183 11
Donor3-GGACGTCTCAGGCGAA-2 1801 4881 13
Donor3-GGATGTTAGCGTAATA-2 1604 4789 9
Donor3-GGATGTTGTATAGTAG-2 1083 5734 8
Donor3-GGATTACTCCCGACTT-2 6453 39121 6
Donor3-GGATTACTCTGGCGTG-2 1939 13311 8
Donor3-GGCAATTTCCAGGGCT-2 2798 6912 5
Donor3-GGCCGATTCGCTTGTC-2 1213 5590 8
Donor3-GGCCGATTCTTCCTTC-2 1658 4880 13
Donor3-GGCGACTAGAATGTTG-2 2577 15566 7
Donor3-GGCGACTAGCGTTTAC-2 3184 14343 6
Donor3-GGCGACTCAATCTACG-2 1976 5312 13
Donor3-GGCGACTCAGAAGCAC-2 1607 6132 7
Donor3-GGCGTGTCAATCAGAA-2 1746 5157 6
Donor3-GGCTCGACAAGCCGTC-2 1603 6184 9
Donor3-GGCTCGACATCCTTGC-2 4759 23405 6
Donor3-GGCTGGTAGGGCTTCC-2 1927 6442 7
Donor3-GGCTGGTCAAGACACG-2 1590 8252 8
Donor3-GGCTGGTCACTTCGAA-2 4022 10028 5
Donor3-GGCTGGTGTACAGTGG-2 1145 5991 8
Donor3-GGGAATGAGCGTTCCG-2 1646 6226 9
Donor3-GGGAATGGTTGCTCCT-2 1293 4187 9
Donor3-GGGACCTCAGAGTGTG-2 1487 4609 13
Donor3-GGGACCTCAGATCGGA-2 5738 30393 4
Donor3-GGGAGATAGGGCACTA-2 1817 8580 7
Donor3-GGGAGATTCCTTGGTC-2 6148 24934 2
Donor3-GGGATGAAGTTACCCA-2 4083 10830 5
Donor3-GGGATGACATTACCTT-2 1567 4606 13
Donor3-GGGATGAGTCTCTTTA-2 1144 4751 8
Donor3-GGGCACTCAAAGCAAT-2 3205 7492 4
Donor3-GGGCACTCAAGTTGTC-2 1925 12134 8
Donor3-GGGCACTTCGTGTAGT-2 4141 12674 5
Donor3-GGGCATCAGGCTAGAC-2 2231 7204 6
Donor3-GGGCATCAGTGTACCT-2 2114 6057 6
Donor3-GGGCATCCAAAGCAAT-2 4234 12042 6
Donor3-GGGTCTGAGTAAGTAC-2 4683 14023 2
Donor3-GGGTCTGGTGACTACT-2 1094 4536 8
Donor3-GGGTTGCCACCAACCG-2 3532 8466 2

Donor3-GGGTTGCTCCAGAAGG-2 1233 5497 8
Donor3-GGGTTGCTCGTGACAT-2 3000 6944 3
Donor3-GGGTTGCTCTACTCAT-2 2544 10471 7
Donor3-GGTATTGAGCGCCTTG-2 1881 5015 9
Donor3-GGTATTGCATGAAGTA-2 5910 46051 6
Donor3-GGTATTGTCTCGGACG-2 1902 10316 7
Donor3-GGTGAAGAGAGAACAG-2 8998 66224 5
Donor3-GGTGAAGAGCCTTGAT-2 2111 10168 7
Donor3-GGTGAAGCATTCTCAT-2 1726 6317 9
Donor3-GGTGAAGTCGTTGCCT-2 5634 22063 6
Donor3-GGTGCGTAGAGTACCG-2 1172 4787 7
Donor3-GGTGCGTAGCTGCAAG-2 4950 16267 2
Donor3-GGTGCGTTCGAACGGA-2 1546 6012 7
Donor3-GGTGCGTTCGGTTAAC-2 5163 16259 1
Donor3-GGTGCGTTCGTCGTTC-2 3154 16104 7
Donor3-GTAACGTCAACAACCT-2 1674 4668 7
Donor3-GTAACTGTCCTTTCGG-2 4843 14871 6
Donor3-GTAACTGTCTTCTGGC-2 4803 25728 4
Donor3-GTACGTATCTCGAGTA-2 5769 25455 2
Donor3-GTACGTATCTGGTGTA-2 2716 8183 6
Donor3-GTACGTATCTTGCCGT-2 1553 8504 8
Donor3-GTACTCCGTGTGACGA-2 1551 9266 8
Donor3-GTACTCCTCTTGCAAG-2 1381 4887 7
Donor3-GTACTTTAGTAGCCGA-2 2914 9702 4
Donor3-GTACTTTAGTCTCGGC-2 1440 6050 7
Donor3-GTACTTTGTTGGTTTG-2 4372 11241 1
Donor3-GTACTTTTCTTCAACT-2 1937 11421 7
Donor3-GTAGTCAGTGCCTGGT-2 4444 12040 2
Donor3-GTATCTTCATCTATGG-2 2509 5171 3
Donor3-GTATCTTGTCTGCGGT-2 2950 6510 3
Donor3-GTATTCTAGAGAACAG-2 1245 4098 7
Donor3-GTATTCTGTGGCTCCA-2 5229 15675 2
Donor3-GTATTCTGTTATCACG-2 1119 5683 8
Donor3-GTCAAGTAGCACCGTC-2 5055 16017 3
Donor3-GTCAAGTAGTACCGGA-2 1345 6394 8
Donor3-GTCAAGTCACACTGCG-2 2948 6604 3
Donor3-GTCACAAAGTTCGATC-2 1517 10430 8
Donor3-GTCACGGAGATCGATA-2 1790 6440 7
Donor3-GTCACGGCACCTCGGA-2 2706 22479 7
Donor3-GTCACGGGTCGCTTTC-2 1264 7885 8
Donor3-GTCACGGTCGAATGCT-2 2421 10230 7
Donor3-GTCATTTCAGTCCTTC-2 1634 4871 13
Donor3-GTCCTCAAGTTAAGTG-2 4025 15482 4
Donor3-GTCCTCACACAGCCCA-2 2095 6336 13
Donor3-GTCCTCACATTATCTC-2 1316 7148 8
Donor3-GTCGGGTAGCCTATGT-2 1616 5498 7
Donor3-GTCGGGTGTCACTGGC-2 1320 5234 7
Donor3-GTCTTCGCAGGCGATA-2 2140 12924 8
Donor3-GTCTTCGCATTCACTT-2 2116 7066 7
Donor3-GTGCAGCAGAGTCGGT-2 5377 20291 6
Donor3-GTGCATAAGTCGATAA-2 2518 5490 5
Donor3-GTGCATAAGTGTCCAT-2 1850 6577 6
Donor3-GTGCATACAGCGTTCG-2 3001 6696 5
Donor3-GTGCATAGTGTTGGGA-2 3873 9988 2
Donor3-GTGCGGTCATGTAGTC-2 2445 5909 6
Donor3-GTGCGGTGTCCGACGT-2 1090 4548 8

Donor3-GTGCGGTTCAATACCG-2 1399 4295 9
Donor3-GTGCTTCGTAGTAGTA-2 1995 5687 13
Donor3-GTGTGCGAGACGCTTT-2 1808 11532 8
Donor3-GTGTGCGAGATATGGT-2 5240 22120 5
Donor3-GTGTGCGAGGCTCAGA-2 2259 8238 10
Donor3-GTGTGCGTCTGGGCCA-2 1481 4744 6
Donor3-GTGTTAGAGGAGTAGA-2 3431 15371 6
Donor3-GTGTTAGAGGGAAACA-2 1092 4656 8
Donor3-GTGTTAGAGTGACATA-2 6236 23787 6
Donor3-GTGTTAGGTCCGAGTC-2 2166 9663 7
Donor3-GTTACAGGTTGTGGCC-2 1518 4710 13
Donor3-GTTCGGGGTTACGACT-2 1777 6411 7
Donor3-GTTCTCGAGACCGGAT-2 2058 6041 13
Donor3-GTTCTCGCAGGATCGA-2 6030 24079 5
Donor3-GTTCTCGGTAGGACAC-2 1427 4592 13
Donor3-GTTCTCGGTCAACATC-2 4171 11456 5
Donor3-GTTCTCGTCTTCCTTC-2 2392 6981 13
Donor3-GTTTCTACAATGGACG-2 1896 8526 7
Donor3-TAAACCGAGGTAAACT-2 5060 16063 6
Donor3-TAAACCGTCTCTTATG-2 1207 5634 8
Donor3-TAAGAGAAGACAAGCC-2 1149 5816 8
Donor3-TAAGAGAGTAGAAGGA-2 4092 23514 7
Donor3-TAAGCGTAGGCTAGCA-2 1958 7239 10
Donor3-TAAGCGTTCGCCAAAT-2 3666 9245 3
Donor3-TAAGTGCCAAACCCAT-2 5573 21842 4
Donor3-TACACGATCCTCTAGC-2 8428 53870 6
Donor3-TACCTATAGGAGTACC-2 4297 11558 4
Donor3-TACCTATCATCCGGGT-2 3873 18742 6
Donor3-TACGGATCAGCGTCCA-2 5651 21591 5
Donor3-TACGGATCATCCCATC-2 6736 30558 2
Donor3-TACGGATGTCAAAGCG-2 2094 6054 4
Donor3-TACGGGCAGAGGGATA-2 3623 8946 4
Donor3-TACGGGCAGCCCTAAT-2 1890 9580 7
Donor3-TACGGGCCACAGAGGT-2 2853 6764 3
Donor3-TACGGGCGTCAGTGGA-2 3841 8728 5
Donor3-TACGGGCTCGTTGCCT-2 3706 10216 4
Donor3-TACGGGCTCTCTTGAT-2 2261 10403 7
Donor3-TACGGTACAATACGCT-2 1411 8559 8
Donor3-TACGGTAGTCCATGAT-2 2629 4697 6
Donor3-TACGGTAGTGATAAGT-2 3059 7326 1
Donor3-TACGGTATCTCATTCA-2 4066 10965 5
Donor3-TACTCATAGATAGGAG-2 1530 10261 8
Donor3-TACTCATAGTGGTAAT-2 2298 7165 11
Donor3-TACTCGCAGTGGTCCC-2 1712 7040 9
Donor3-TACTCGCGTATATCCG-2 1509 4091 13
Donor3-TACTCGCGTATCACCA-2 1035 4557 8
Donor3-TACTTACAGATCCCAT-2 5971 20258 3
Donor3-TACTTACAGGAATGGA-2 984 4404 8
Donor3-TACTTACGTCTTGTCC-2 2533 11981 7
Donor3-TACTTACTCTGGAGCC-2 2856 19677 8
Donor3-TACTTGTGTCGCGTGT-2 2366 5457 2
Donor3-TAGACCAGTAATCGTC-2 2972 6721 3
Donor3-TAGACCATCAGGCAAG-2 1855 7257 9
Donor3-TAGAGCTCAGGTCCAC-2 2378 7893 10
Donor3-TAGAGCTGTTACGGAG-2 1859 5742 6
Donor3-TAGAGCTGTTTAGGAA-2 1621 4359 10

Donor3-TAGAGCTTCAGGTAAA-2 1741 7737 7
Donor3-TAGAGCTTCATGCTCC-2 5579 23488 2
Donor3-TAGCCGGAGTATTGGA-2 5341 20367 4
Donor3-TAGCCGGAGTCCATAC-2 903 4240 8
Donor3-TAGCCGGCACACCGCA-2 2051 8758 7
Donor3-TAGGCATAGTGTCCCG-2 1183 4965 8
Donor3-TAGGCATGTGTGACCC-2 6392 24189 1
Donor3-TAGGCATGTTCCGGCA-2 2308 14068 7
Donor3-TAGGCATTCCTGTACC-2 1684 7446 7
Donor3-TAGTGGTCATGAGCGA-2 7331 37436 5
Donor3-TAGTTGGCATACTACG-2 1702 10568 8
Donor3-TAGTTGGTCACATACG-2 4046 10208 3
Donor3-TATCAGGGTGTGACCC-2 3933 16211 6
Donor3-TATCTCAAGTAATCCC-2 1033 5225 8
Donor3-TATCTCAGTTAAAGTG-2 918 4394 8
Donor3-TATCTCATCCGCTGTT-2 1906 5723 13
Donor3-TATGCCCAGAAACGCC-2 3286 13231 6
Donor3-TATTACCAGTGTCTCA-2 1646 4066 11
Donor3-TATTACCCAATAGCGG-2 2585 8449 6
Donor3-TATTACCCAATGAAAC-2 1797 11948 8
Donor3-TCAACGAAGGAGTCTG-2 1691 11500 8
Donor3-TCAACGACATGACGGA-2 3158 7831 4
Donor3-TCAACGACATGTCCTC-2 1237 4545 8
Donor3-TCAATCTCACAACGCC-2 1413 4192 13
Donor3-TCAATCTGTATCAGTC-2 8139 52663 5
Donor3-TCACAAGTCACCACCT-2 2292 6445 4
Donor3-TCACGAAGTCTTGATG-2 5229 17156 1
Donor3-TCAGATGAGGTGCAAC-2 5121 19039 2
Donor3-TCAGATGTCCAAGTAC-2 2955 10140 11
Donor3-TCAGATGTCTACGAGT-2 1294 4940 7
Donor3-TCAGCAACACCCATTC-2 3425 21385 7
Donor3-TCAGCAACATTTGCTT-2 2121 6426 10
Donor3-TCAGCTCCACGAGAGT-2 1466 6150 7
Donor3-TCAGCTCCATACCATG-2 3760 9451 3
Donor3-TCAGCTCGTGAAATCA-2 2917 6484 6
Donor3-TCAGCTCTCCGCGGTA-2 1197 6919 8
Donor3-TCAGGATAGCTAAACA-2 1431 9378 8
Donor3-TCAGGTACATCGATGT-2 2879 5859 6
Donor3-TCAGGTATCCTGCAGG-2 5985 26652 5
Donor3-TCAGGTATCCTTTCTC-2 3942 9850 4
Donor3-TCATTACCAAGGTTCT-2 2049 5478 10
Donor3-TCATTACTCATGCTCC-2 1592 6304 7
Donor3-TCATTTGAGCTAGTCT-2 2514 4980 5
Donor3-TCATTTGCAGCTCCGA-2 6021 24480 6
Donor3-TCATTTGGTCGCTTCT-2 1819 4433 10
Donor3-TCATTTGGTGACGCCT-2 4674 14327 5
Donor3-TCATTTGTCTTGTTTG-2 4425 13350 5
Donor3-TCCACACCATATACGC-2 1484 4410 13
Donor3-TCCACACCATTACGAC-2 3960 9661 5
Donor3-TCCACACGTCGACTAT-2 6770 34150 4
Donor3-TCCCGATAGAACTGTA-2 1307 6175 8
Donor3-TCCCGATAGCTGGAAC-2 1822 8844 8
Donor3-TCCCGATAGGTTACCT-2 1557 7854 7
Donor3-TCCCGATCATGCTAGT-2 6597 31451 5
Donor3-TCCCGATTCTAACCGA-2 4243 12218 2
Donor3-TCCCGATTCTTAGAGC-2 1631 8095 7

Donor3-TCGAGGCCATAGACTC-2 5217 20877 5
Donor3-TCGAGGCTCAACGCTA-2 3404 7975 2
Donor3-TCGAGGCTCTCGCTTG-2 1538 4271 13
Donor3-TCGCGAGAGGCCATAG-2 1520 7127 8
Donor3-TCGCGAGCAACTGCGC-2 3118 17676 7
Donor3-TCGCGAGTCTATCCCG-2 2255 19978 8
Donor3-TCGCGTTCAGAGTGTG-2 3115 15626 7
Donor3-TCGCGTTGTCGCGAAA-2 1823 9480 7
Donor3-TCGGGACTCGTCACGG-2 3201 7710 2
Donor3-TCGGTAAAGGAGTAGA-2 1862 6090 13
Donor3-TCGTACCAGCTGGAAC-2 2098 6112 13
Donor3-TCGTACCGTATTCTCT-2 2517 8157 10
Donor3-TCGTACCTCTTAGCCC-2 1152 5696 8
Donor3-TCGTAGAAGCAGATCG-2 4932 19663 6
Donor3-TCGTAGAGTTCAGCGC-2 1181 5186 8
Donor3-TCGTAGATCGTACGGC-2 2218 8465 7
Donor3-TCTATTGTCATAAAGG-2 5181 31653 6
Donor3-TCTCATATCACATACG-2 1453 6680 8
Donor3-TCTCTAACACGTAAGG-2 3565 8775 3
Donor3-TCTGAGACACAGACAG-2 2119 5090 5
Donor3-TCTGAGAGTGACTACT-2 1695 6795 7
Donor3-TCTGAGAGTGTAACGG-2 2982 6886 6
Donor3-TCTGGAAGTGTGCCTG-2 972 4255 8
Donor3-TCTGGAAGTTGGTGGA-2 1249 5501 8
Donor3-TCTTCGGAGGCTAGCA-2 1112 4525 8
Donor3-TGAAAGAAGATGGCGT-2 5543 24990 6
Donor3-TGAAAGACAGTAACGG-2 1146 5898 8
Donor3-TGACAACCAAGCGTAG-2 2740 17186 7
Donor3-TGACAACCACGTTGGC-2 6802 60176 6
Donor3-TGACGGCAGTCCAGGA-2 1332 4927 7
Donor3-TGACTTTTCATAGCAC-2 4221 8542 1
Donor3-TGAGAGGAGCGTAATA-2 1371 4518 8
Donor3-TGAGAGGTCACGACTA-2 1769 4943 10
Donor3-TGAGCATAGAGCTTCT-2 1632 5531 9
Donor3-TGAGCATAGCGTCAAG-2 2950 6275 3
Donor3-TGAGCATCAAGCCGCT-2 2192 4125 6
Donor3-TGAGCATCACAAGTAA-2 4401 11903 4
Donor3-TGAGCCGAGAGGGATA-2 4079 12856 1
Donor3-TGAGCCGGTCGACTGC-2 9456 78364 5
Donor3-TGAGGGACAATCCGAT-2 1124 4422 8
Donor3-TGAGGGAGTGACTACT-2 1906 4260 5
Donor3-TGATTTCAGAGGACGG-2 1554 5172 7
Donor3-TGATTTCTCAGTCCCT-2 3276 7235 3
Donor3-TGATTTCTCGGACAAG-2 1087 5874 8
Donor3-TGCCAAAAGCACAGGT-2 3297 7742 1
Donor3-TGCCAAACAGCATGAG-2 2563 5850 4
Donor3-TGCCAAAGTGGCAAAC-2 3006 6711 3
Donor3-TGCCCATCAATGTTGC-2 1184 5205 8
Donor3-TGCCCTAAGCGATTCT-2 1599 4856 13
Donor3-TGCCCTACATTAGCCA-2 6258 48670 6
Donor3-TGCCCTATCAGCTCGG-2 1871 11848 8
Donor3-TGCCCTATCATATCGG-2 1341 6346 7
Donor3-TGCCCTATCGCCAAAT-2 1557 4620 13
Donor3-TGCGCAGCACTTCGAA-2 2649 5977 3
Donor3-TGCGCAGGTGATGATA-2 1798 5008 13
Donor3-TGCGCAGTCCCATTAT-2 2178 4319 6

Donor3-TGCGGGTAGCGAGAAA-2 2527 5512 2
Donor3-TGCGGGTCATCTACGA-2 4001 11466 6
Donor3-TGCGTGGAGCGATCCC-2 1140 5590 8
Donor3-TGCGTGGAGGCAGTCA-2 1722 9399 8
Donor3-TGCGTGGCACCACGTG-2 2522 4420 5
Donor3-TGCGTGGCATTTGCCC-2 1501 6609 7
Donor3-TGCTACCAGACACGAC-2 2805 8609 10
Donor3-TGCTACCGTTCTCATT-2 6687 30000 6
Donor3-TGCTACCTCCTTTCGG-2 1048 5016 8
Donor3-TGCTGCTAGCACAGGT-2 1429 5862 8
Donor3-TGCTGCTAGCCACGCT-2 4495 15444 5
Donor3-TGGACGCCATGCAATC-2 3089 6905 3
Donor3-TGGACGCTCATCTGCC-2 1477 8896 8
Donor3-TGGCGCATCATGCAAC-2 1376 7245 8
Donor3-TGGCGCATCTTACCTA-2 3528 19169 7
Donor3-TGGCTGGAGCCCAGCT-2 1068 4674 8
Donor3-TGGCTGGAGCCCGAAA-2 1264 4210 7
Donor3-TGGCTGGTCCATGAAC-2 1948 9148 7
Donor3-TGGGCGTAGAAACGCC-2 2989 16381 7
Donor3-TGGGCGTTCTAAGCCA-2 1562 8009 8
Donor3-TGGTTAGCACTGCCAG-2 5627 19832 5
Donor3-TGGTTAGTCCTTTCGG-2 1903 5493 6
Donor3-TGGTTCCCAATCAGAA-2 5337 18797 6
Donor3-TGGTTCCCAGCCACCA-2 2302 11545 7
Donor3-TGTATTCAGAAGCCCA-2 1434 4229 13
Donor3-TGTATTCAGAAGGTTT-2 2572 6326 4
Donor3-TGTATTCAGAGACTTA-2 1771 9413 7
Donor3-TGTATTCAGTATCGAA-2 2702 6004 6
Donor3-TGTATTCAGTTCGATC-2 1585 4272 8
Donor3-TGTATTCCAAAGCGGT-2 3317 7710 6
Donor3-TGTATTCCAGGGCATA-2 2914 15957 7
Donor3-TGTATTCTCATGTCCC-2 1947 11215 7
Donor3-TGTCCCAAGGGATCTG-2 3739 16933 6
Donor3-TGTCCCAGTCACACGC-2 2454 5691 6
Donor3-TGTGGTACAAATTGCC-2 1774 10649 8
Donor3-TGTGGTACAATGCCAT-2 1923 7717 7
Donor3-TGTGGTAGTCAAAGCG-2 4675 14693 6
Donor3-TGTGTTTAGTGCCAGA-2 4744 17399 6
Donor3-TGTTCCGAGCCCAACC-2 1740 5935 9
Donor3-TGTTCCGTCGAACTGT-2 1536 6559 7
Donor3-TGTTCCGTCGACGGAA-2 1855 5388 10
Donor3-TTAACTCAGTGTTTGC-2 1415 5759 8
Donor3-TTAACTCAGTTCGCGC-2 6118 24942 5
Donor3-TTAACTCGTCAGCTAT-2 4251 25932 7
Donor3-TTAGGACGTGATGATA-2 2375 7780 10
Donor3-TTAGGACTCACTTATC-2 5054 15773 2
Donor3-TTAGGCATCCTTTACA-2 7056 46612 4
Donor3-TTAGTTCCAGCGTAAG-2 1567 4142 13
Donor3-TTAGTTCGTCGAACAG-2 1191 5689 8
Donor3-TTAGTTCGTCTCGTTC-2 2198 5733 6
Donor3-TTATGCTGTTCGTTGA-2 1318 6488 8
Donor3-TTATGCTTCATGTCCC-2 3764 18983 6
Donor3-TTCCCAGAGAGTACCG-2 1642 4912 13
Donor3-TTCCCAGGTAGTGAAT-2 1761 4436 10
Donor3-TTCGGTCCATTCCTGC-2 3172 16432 7
Donor3-TTCGGTCGTCAGTGGA-2 1082 5140 8

Donor3-TTCGGTCGTCTCCACT-2 3533 8545 3
Donor3-TTCGGTCTCCGCATCT-2 7742 52125 6
Donor3-TTCTACAAGTATCGAA-2 3765 9859 3
Donor3-TTCTACACAATCGGTT-2 3030 12713 6
Donor3-TTCTCAAAGTCAATAG-2 2941 8324 4
Donor3-TTCTCAACAAGGACAC-2 4212 24735 6
Donor3-TTCTCCTAGATATGGT-2 1810 5321 13
Donor3-TTCTCCTAGGCGACAT-2 3202 19690 7
Donor3-TTCTCCTTCACTCCTG-2 5153 43422 7
Donor3-TTCTCCTTCCGAACGC-2 5282 16885 3
Donor3-TTCTCCTTCTCTGTCG-2 7053 36249 6
Donor3-TTCTTAGGTAATTGGA-2 2199 11254 7
Donor3-TTCTTAGGTCGAGATG-2 3427 11916 10
Donor3-TTGACTTAGTTCCACA-2 3626 7763 6
Donor3-TTGCCGTAGCTGAACG-2 1826 5046 10
Donor3-TTGCCGTCACGAGGTA-2 2437 7681 13
Donor3-TTGCCGTCAGACACTT-2 2593 11361 7
Donor3-TTGCGTCAGCGTGAGT-2 1037 5419 8
Donor3-TTGGAACCAATGTTGC-2 5306 18138 3
Donor3-TTGGAACCACCGAAAG-2 7622 45145 6
Donor3-TTGGAACGTTATCGGT-2 1290 4297 13
Donor3-TTGGAACTCCAAATGC-2 4978 14842 6
Donor3-TTGGAACTCTTGCATT-2 1147 5548 8
Donor3-TTGGCAAAGGGAAACA-2 1752 5050 12
Donor3-TTGGCAACACATCTTT-2 1143 5541 8
Donor3-TTGGCAATCACGAAGG-2 2518 11501 7
Donor3-TTGGCAATCCCGGATG-2 5505 17212 2
Donor3-TTGGCAATCTGAAAGA-2 1691 5236 13
Donor3-TTGGCAATCTTCGAGA-2 2058 16191 8
Donor3-TTGTAGGAGACAAAGG-2 1456 8311 8
Donor3-TTGTAGGAGCTCTCGG-2 2101 7619 9
Donor3-TTGTAGGCAGTATGCT-2 2150 4957 5
Donor3-TTTACTGAGCTCCTTC-2 2377 11672 7
Donor3-TTTACTGCAGGTCTCG-2 4144 19565 6
Donor3-TTTATGCCATATGAGA-2 4399 12977 6
Donor3-TTTATGCGTGTGTGCC-2 2764 5514 3
Donor3-TTTCCTCGTGCACTTA-2 3534 8733 3
Donor3-TTTCCTCTCAAACCAC-2 1082 4467 8
Donor3-TTTCCTCTCCCTTGCA-2 4152 20094 5
Donor3-TTTGCGCCACGGCTAC-2 6110 24350 4
Donor3-TTTGCGCGTAGGCATG-2 4690 14982 4
Donor3-TTTGCGCGTTCCACTC-2 3317 7642 6
Donor3-TTTGGTTAGGGTGTGT-2 5773 20964 5
Donor3-TTTGGTTAGTGAACGC-2 3801 10178 1
Donor1-AAACCTGAGAAACCTA-1 4078 10563 3
Donor1-AAACCTGAGAAAGTGG-1 2191 7587 10
Donor1-AAACGGGAGGTTCCTA-1 5032 25297 6
Donor1-AAACGGGCACTGTGTA-1 3282 7254 4
Donor1-AAACGGGGTGAACCTT-1 1126 2980 9
Donor1-AAAGATGAGGACACCA-1 1330 3130 13
Donor1-AAAGATGCATTGCGGC-1 1861 5201 10
Donor1-AAAGATGCATTGGGCC-1 1777 4349 13
Donor1-AAAGATGTCAAGATCC-1 3081 6653 6
Donor1-AAAGCAATCTCGGACG-1 6710 33040 6
Donor1-AAAGTAGCATGTCCTC-1 2398 8321 9
Donor1-AAATGCCCATTGTGCA-1 3342 11500 11

Donor1-AACACGTAGTCGCCGT-1 3188 7060 2
Donor1-AACACGTCATTCCTCG-1 1537 5531 8
Donor1-AACACGTGTCCATGAT-1 2814 5328 6
Donor1-AACCATGAGGAGTACC-1 1351 4203 13
Donor1-AACCATGAGGCGACAT-1 2678 5950 3
Donor1-AACCATGCATGGGACA-1 970 3234 8
Donor1-AACCATGGTAAGGGAA-1 1352 5312 8
Donor1-AACCGCGTCCCAAGAT-1 2562 8900 10
Donor1-AACCGCGTCGCTTAGA-1 1219 3006 7
Donor1-AACGTTGAGAAACCAT-1 2961 7040 3
Donor1-AACGTTGCAACACCCG-1 2212 5976 11
Donor1-AACTCAGGTGACCAAG-1 5067 28337 10
Donor1-AACTCCCAACTGCTA-1 2371 8409 9
Donor1-AACTCCCCACTCTGTC-1 5443 18249 2
Donor1-AACTCCCGTACCGGCT-1 3237 7714 3
Donor1-AACTCTTAGGAATCGC-1 1429 3093 11
Donor1-AACTCTTCACCGTTGG-1 1980 4428 6
Donor1-AACTCTTGTCTACCTC-1 4772 19399 6
Donor1-AACTCTTGTGATAAGT-1 2676 5714 3
Donor1-AACTGGTCATTGGCGC-1 2727 11666 10
Donor1-AACTGGTGTATAGTAG-1 2483 7578 11
Donor1-AACTTTCCAGGACGTA-1 1254 4070 8
Donor1-AAGACCTAGAATGTTG-1 6483 30106 5
Donor1-AAGACCTAGGTTACCT-1 2095 10619 8
Donor1-AAGCCGCAGTAGGTGC-1 2718 8554 6
Donor1-AAGCCGCAGTATCGAA-1 1542 5206 13
Donor1-AAGGAGCGTAAGAGGA-1 1772 4468 10
Donor1-AAGGCAGCACACATGT-1 2328 8610 10
Donor1-AAGGCAGCACGGACAA-1 2730 5896 3
Donor1-AAGGCAGCACTTAAGC-1 2765 15220 7
Donor1-AAGGCAGTCCCGGATG-1 1876 3317 4
Donor1-AAGGTTCAGACCGGAT-1 3267 7716 2
Donor1-AAGGTTCAGGAGTCTG-1 2851 5618 2
Donor1-AAGGTTCTCAAACCGT-1 1234 3108 13
Donor1-AAGTCTGAGAGCTATA-1 1690 5074 10
Donor1-AAGTCTGCACCTATCC-1 1405 3829 13
Donor1-AAGTCTGGTTGCGTTA-1 4485 12493 4
Donor1-AATCCAGGTAAATGAC-1 1460 3251 11
Donor1-AATCCAGTCTCGGACG-1 4894 14365 2
Donor1-AATCGGTCATCGATGT-1 1146 3606 8
Donor1-AATCGGTTCCAGAAGG-1 2113 5336 6
Donor1-ACACCAAAGAAACCGC-1 2066 4858 13
Donor1-ACACCAATCCGATATG-1 1276 2845 11
Donor1-ACACCCTAGTTGTAGA-1 2811 5900 3
Donor1-ACACCCTCAGATCGGA-1 1688 4340 10
Donor1-ACACCCTGTACATCCA-1 1927 3448 3
Donor1-ACACCCTGTACTCGCG-1 1668 3719 11
Donor1-ACACCCTTCAATCACG-1 3534 10008 6
Donor1-ACACCGGAGAAACCTA-1 5133 15932 1
Donor1-ACACCGGAGAACTCGG-1 2271 4589 2
Donor1-ACACTGACACCGTTGG-1 1665 5376 9
Donor1-ACAGCCGCAGCGAACA-1 2322 8455 6
Donor1-ACAGCCGGTACCGTTA-1 5911 27184 4
Donor1-ACAGCCGGTTCCGGCA-1 1616 4264 12
Donor1-ACAGCTACAGTGGGAT-1 2486 7979 10
Donor1-ACAGCTAGTCCCTACT-1 2007 5148 8

Donor1-ACAGCTAGTGTGCCTG-1 3055 6977 2
Donor1-ACAGCTAGTTCCACGG-1 1313 4151 9
Donor1-ACATACGAGCAGCCTC-1 1558 4445 9
Donor1-ACATACGCAAATACAG-1 2024 5845 9
Donor1-ACATACGGTAGATTAG-1 1031 2944 9
Donor1-ACATCAGGTTAGGGTG-1 3927 12638 6
Donor1-ACCGTAAGTTAAGAAC-1 3303 7817 3
Donor1-ACCGTAATCCCAACGG-1 1870 6156 13
Donor1-ACCTTTATCGTTGCCT-1 1755 5745 9
Donor1-ACGAGCCTCAGTTTGG-1 1341 3454 13
Donor1-ACGAGGATCATCGGAT-1 4110 10345 2
Donor1-ACGATACGTGCATCTA-1 7542 38740 5
Donor1-ACGATGTAGGGTATCG-1 2577 8460 11
Donor1-ACGATGTTCACCCGAG-1 3896 9560 1
Donor1-ACGCAGCTCATGTCTT-1 1400 3904 13
Donor1-ACGCCAGCAAGTCTGT-1 2619 10190 10
Donor1-ACGCCGATCCTTGGTC-1 3058 6933 3
Donor1-ACGGAGATCCAAAGTC-1 2677 7353 11
Donor1-ACGGAGATCGCTTAGA-1 3382 8208 3
Donor1-ACGGAGATCGTCCGTT-1 3785 9077 2
Donor1-ACGGCCAAGGGCACTA-1 3057 10114 11
Donor1-ACGGCCACAGGTGCCT-1 2217 5552 11
Donor1-ACGGGCTAGAGTAATC-1 3162 7655 1
Donor1-ACGGGCTCAGAGCCAA-1 3448 8233 6
Donor1-ACGGGCTCAGCCAGAA-1 1360 3783 13
Donor1-ACGGGCTCATGCTAGT-1 1665 4079 6
Donor1-ACGGGCTGTTCGTGAT-1 3997 10225 5
Donor1-ACGGGCTTCGACAGCC-1 2472 6921 13
Donor1-ACGGGCTTCTCCCTGA-1 1369 3653 13
Donor1-ACGGGTCCAAGCCCAC-1 2454 11513 7
Donor1-ACGGGTCCAATGGAAT-1 1105 3564 8
Donor1-ACGGGTCCACTGTCGG-1 3532 8918 3
Donor1-ACGGGTCTCATCATTC-1 1288 3944 7
Donor1-ACGGGTCTCGTCTGAA-1 2198 4378 5
Donor1-ACTATCTGTTCACGGC-1 1591 3770 11
Donor1-ACTGAACAGATGGGTC-1 5951 19850 1
Donor1-ACTGAACGTAAGGATT-1 1556 5172 9
Donor1-ACTGAACGTCATACTG-1 1477 4321 13
Donor1-ACTGAACGTCGCTTTC-1 3212 7973 3
Donor1-ACTGAACGTGTCGCTG-1 3987 9689 3
Donor1-ACTGAGTGTGTGGCTC-1 3291 11377 11
Donor1-ACTGCTCCATGTAGTC-1 5840 20558 1
Donor1-ACTGCTCGTATTCGTG-1 2041 3923 2
Donor1-ACTGCTCTCGCATGAT-1 1858 3480 3
Donor1-ACTGTCCAGATTACCC-1 3417 7979 3
Donor1-ACTGTCCCACCTTGTC-1 4497 13374 6
Donor1-ACTGTCCCACTGCCAG-1 2413 8594 10
Donor1-ACTGTCCCATACTCTT-1 2070 6078 13
Donor1-ACTGTCCTCGCGCCAA-1 1435 3438 13
Donor1-ACTTTCAAGGACGAAA-1 4621 15017 9
Donor1-ACTTTCAAGTACGACG-1 1206 3656 7
Donor1-ACTTTCAAGTATTGGA-1 2108 5847 10
Donor1-ACTTTCAGTTGCTCCT-1 5568 16205 2
Donor1-ACTTTCATCTTCTGGC-1 2858 6924 6
Donor1-AGAATAGAGTGGTAAT-1 3714 17217 10
Donor1-AGAATAGGTCTTCGTC-1 3881 35773 8

Donor1-AGACGTTAGGACCACA-1 4764 15556 6
Donor1-AGACGTTAGGCATGGT-1 2001 5458 10
Donor1-AGACGTTGTCCGAAGA-1 1210 2980 7
Donor1-AGACGTTTCATTTGGG-1 3305 7176 1
Donor1-AGAGCGAAGCAGACTG-1 1590 6713 9
Donor1-AGAGCGAAGTCGTACT-1 1288 2940 11
Donor1-AGAGCGACACAACGCC-1 3828 9849 2
Donor1-AGAGCTTAGTGGGATC-1 2190 6240 6
Donor1-AGAGCTTCACGACGAA-1 1662 3813 10
Donor1-AGAGCTTGTCGGGTCT-1 3973 10397 2
Donor1-AGAGCTTTCTTGTATC-1 980 3631 8
Donor1-AGATCTGGTTACTGAC-1 1208 4315 8
Donor1-AGATCTGTCTCCAACC-1 2919 5754 5
Donor1-AGCAGCCAGACAGGCT-1 1558 3465 8
Donor1-AGCAGCCCATCACGAT-1 4217 10811 3
Donor1-AGCAGCCTCCCTCTTT-1 4788 17136 10
Donor1-AGCATACAGAGTGACC-1 1584 3772 11
Donor1-AGCATACCAGCCTTTC-1 2954 6359 3
Donor1-AGCATACGTTCGCGAC-1 1604 3614 11
Donor1-AGCCTAACAAACTGTC-1 9112 90976 6
Donor1-AGCGTATAGAGTAAGG-1 2532 16775 8
Donor1-AGCGTATAGGCTCAGA-1 1713 4468 10
Donor1-AGCGTATCACATTCGA-1 7187 29696 1
Donor1-AGCGTATGTCGTCTTC-1 1412 3158 13
Donor1-AGCGTATTCCGTCATC-1 1964 10304 8
Donor1-AGCGTCGAGCCGCCTA-1 2231 6111 11
Donor1-AGCGTCGAGTATCTCG-1 1503 4726 13
Donor1-AGCGTCGAGTGAACGC-1 1229 3608 8
Donor1-AGCGTCGAGTTAGCGG-1 1105 3227 13
Donor1-AGCGTCGCACTCGACG-1 2356 6643 11
Donor1-AGCGTCGGTGAGGGTT-1 2471 13179 7
Donor1-AGCTCCTCAAAGGTGC-1 2034 7128 7
Donor1-AGCTCCTGTGCAACGA-1 4431 13468 4
Donor1-AGCTCCTTCCTTAATC-1 4262 10909 2
Donor1-AGCTCTCCAATGGTCT-1 3734 8163 5
Donor1-AGCTTGAAGCCATCGC-1 1076 3233 13
Donor1-AGCTTGATCGTCGTTC-1 2242 6579 11
Donor1-AGGCCACCATGACGGA-1 1547 4269 9
Donor1-AGGCCGTAGAGTTGGC-1 1922 5133 11
Donor1-AGGCCGTTCCCTTGTG-1 5574 18028 2
Donor1-AGGGAGTCATGCTAGT-1 3073 6514 3
Donor1-AGGGAGTCATTGGGCC-1 1307 2969 10
Donor1-AGGGAGTTCAAACGGG-1 1737 4363 11
Donor1-AGGGATGAGGGCTTGA-1 5256 18399 2
Donor1-AGGGATGCATTCTCAT-1 2328 9012 7
Donor1-AGGGATGGTCTCTTAT-1 1812 8598 8
Donor1-AGGGATGGTGGTGTAG-1 1472 2874 11
Donor1-AGGGATGGTGTCGCTG-1 2106 4442 2
Donor1-AGGGTGAAGATATGCA-1 3309 7641 2
Donor1-AGGGTGAGTAAGTAGT-1 2461 6983 13
Donor1-AGGGTGAGTGCGGTAA-1 2851 6187 3
Donor1-AGGTCATGTAGGACAC-1 2933 6664 2
Donor1-AGGTCATTCACCACCT-1 2776 5390 5
Donor1-AGGTCCGAGGAGTCTG-1 1399 3324 13
Donor1-AGGTCCGAGGGTTTCT-1 1230 3487 9
Donor1-AGGTCCGCAGGTGGAT-1 1450 3189 11

Donor1-AGTAGTCCAACAACCT-1 4328 12801 4
Donor1-AGTGGGACACGAAATA-1 3074 7031 3
Donor1-AGTGGGAGTACCGAGA-1 3130 10465 6
Donor1-AGTGGGATCTAACTGG-1 2130 7529 13
Donor1-AGTGTCAAGATGCGAC-1 1200 3788 9
Donor1-AGTTGGTGTAGCCTAT-1 2243 5394 5
Donor1-ATAACGCAGCCAGAAC-1 2855 5985 3
Donor1-ATAACGCTCAGAGACG-1 1097 3127 8
Donor1-ATAAGAGCATCTACGA-1 3343 11209 11
Donor1-ATAAGAGCATGCATGT-1 3304 8164 3
Donor1-ATAAGAGGTGTTCGAT-1 2942 6985 3
Donor1-ATAAGAGTCTTCATGT-1 2788 9156 11
Donor1-ATAAGAGTCTTGAGAC-1 2801 5649 3
Donor1-ATAGACCAGTTCGATC-1 3309 8316 3
Donor1-ATAGACCGTTAAAGTG-1 3206 7583 3
Donor1-ATCACGATCGACCAGC-1 1729 6569 13
Donor1-ATCATCTAGGATTCGG-1 1361 3533 11
Donor1-ATCATCTCACCGTTGG-1 1649 4221 13
Donor1-ATCATCTGTTGCGCAC-1 4251 21399 10
Donor1-ATCATCTTCGCACTCT-1 1123 3434 8
Donor1-ATCATGGGTTTACTCT-1 1467 3149 11
Donor1-ATCATGGGTTTGACTG-1 2130 3578 6
Donor1-ATCCACCCAACCGCCA-1 1576 5218 13
Donor1-ATCCACCCACATCTTT-1 1631 4922 13
Donor1-ATCCACCCACGAAGCA-1 4886 15033 2
Donor1-ATCGAGTAGGAGTCTG-1 1880 6164 13
Donor1-ATCGAGTCAGGTGCCT-1 1282 3172 13
Donor1-ATCGAGTCAGGTTTCA-1 3228 7572 3
Donor1-ATCGAGTGTTACCGAT-1 1743 3801 11
Donor1-ATGCGATAGGTCGGAT-1 1380 3671 7
Donor1-ATGCGATGTCGGGTCT-1 1151 2855 7
Donor1-ATGGGAGGTTGGGACA-1 2192 5880 11
Donor1-ATTACTCAGTTAGCGG-1 1085 2936 7
Donor1-ATTACTCCACCAGGTC-1 6122 20547 2
Donor1-ATTACTCGTCCAACTA-1 4341 12316 6
Donor1-ATTATCCGTCGCATCG-1 3506 7550 3
Donor1-ATTCTACAGGTGCACA-1 1016 3457 8
Donor1-ATTGGACAGAGATGAG-1 3072 6685 3
Donor1-ATTGGTGAGACCTAGG-1 1867 5993 13
Donor1-ATTGGTGAGCGATTCT-1 3229 7809 3
Donor1-ATTGGTGAGTTATCGC-1 1367 3027 13
Donor1-ATTGGTGGTCATACTG-1 2831 9400 10
Donor1-ATTGGTGGTTTACTCT-1 1243 3652 8
Donor1-ATTGGTGTCGGATGGA-1 1137 3474 9
Donor1-ATTTCTGAGAACAACT-1 2055 3780 2
Donor1-ATTTCTGAGGCTCAGA-1 5534 19126 2
Donor1-ATTTCTGTCCAAAGTC-1 2754 5768 3
Donor1-CAACCAATCAGTTAGC-1 1423 3170 11
Donor1-CAACCAATCTCTTGAT-1 2801 6277 4
Donor1-CAACCTCAGAATGTGT-1 1795 6757 7
Donor1-CAACCTCGTAAGTGTA-1 4414 21604 10
Donor1-CAACTAGGTTTACTCT-1 1664 3414 11
Donor1-CAAGAAAGTCAATGTC-1 1736 4562 10
Donor1-CAAGAAATCCGAATGT-1 2697 12168 10
Donor1-CAAGATCGTAGCTTGT-1 3002 6837 3
Donor1-CAAGTTGCACCTTGTC-1 1219 4357 8

Donor1-CAAGTTGTCACAAACC-1 1595 4823 7
Donor1-CACAAACGTCACACGC-1 1108 3140 8
Donor1-CACAAACGTGCAGACA-1 2632 5211 5
Donor1-CACACAAAGGAGTCTG-1 6109 22119 5
Donor1-CACACCTGTCTGCAAT-1 2028 4334 13
Donor1-CACACCTGTTACGACT-1 1974 10984 8
Donor1-CACACCTTCAGCGACC-1 1107 2995 7
Donor1-CACACTCCAAGGTGTG-1 2334 6396 10
Donor1-CACAGGCCAAGTCATC-1 1308 4917 8
Donor1-CACAGTATCTCAAACG-1 1511 3396 11
Donor1-CACATTTAGCAAATCA-1 1937 5303 13
Donor1-CACATTTGTAGGCTGA-1 3045 7142 3
Donor1-CACCACTTCTGCTGCT-1 3537 7933 3
Donor1-CACCAGGGTCCGAAGA-1 2245 7180 9
Donor1-CACCAGGTCCTGTAGA-1 2574 5275 3
Donor1-CACCTTGAGAGATGAG-1 2286 10035 8
Donor1-CACCTTGAGTAATCCC-1 4672 25836 10
Donor1-CACCTTGCAGGACCCT-1 1937 5865 13
Donor1-CACCTTGTCTATCCCG-1 4430 12324 6
Donor1-CACTCCACATTAACCG-1 3653 8284 3
Donor1-CACTCCATCCTTTCTC-1 1429 4058 7
Donor1-CAGAATCAGCTGCAAG-1 1461 4212 13
Donor1-CAGAATCCATCCTAGA-1 2256 6704 13
Donor1-CAGAATCTCCAACCAA-1 1154 4050 8
Donor1-CAGATCACAATGGAAT-1 1794 4386 10
Donor1-CAGCAGCAGGTCATCT-1 2000 7141 9
Donor1-CAGCAGCGTATTCTCT-1 2947 6125 2
Donor1-CAGCATAAGACTTTCG-1 1237 3493 7
Donor1-CAGCATAGTTAAAGTG-1 2183 6120 10
Donor1-CAGCATATCATAAAGG-1 1245 2854 8
Donor1-CAGCGACAGACTTGAA-1 2080 3931 3
Donor1-CAGCTAAAGCGATCCC-1 2961 6894 3
Donor1-CAGCTAACACCATCCT-1 2801 10999 10
Donor1-CAGCTAAGTCTGATTG-1 1059 3685 8
Donor1-CAGCTAAGTTCGCGAC-1 3525 8118 3
Donor1-CAGCTAAGTTTCGCTC-1 1535 5528 13
Donor1-CAGCTAATCAAACAAG-1 5557 17617 1
Donor1-CAGCTAATCAGCTGGC-1 1573 2905 6
Donor1-CAGCTGGCATGGATGG-1 2116 3396 5
Donor1-CAGTAACAGAGAGCTC-1 2206 8113 9
Donor1-CAGTAACAGGATGCGT-1 1551 7499 8
Donor1-CAGTAACAGTGGGATC-1 1277 3038 10
Donor1-CAGTAACCAGGTCGTC-1 1296 4772 8
Donor1-CAGTAACCATGTCTCC-1 2556 8998 9
Donor1-CAGTCCTAGACGCTTT-1 1875 6020 13
Donor1-CAGTCCTTCACGCGGT-1 1299 3256 6
Donor1-CATATGGGTGCTCTTC-1 5381 16573 1
Donor1-CATATTCGTAGCACGA-1 2239 8917 7
Donor1-CATATTCTCCTATGTT-1 950 3196 8
Donor1-CATCAAGCATTTCAGG-1 1357 3908 12
Donor1-CATCAGACACGCTTTC-1 2179 3734 5
Donor1-CATCAGAGTGGTACAG-1 1179 4519 8
Donor1-CATCAGATCTCGTATT-1 4902 22081 10
Donor1-CATCCACCATACAGCT-1 1638 5062 7
Donor1-CATCCACGTGACGGTA-1 1918 3186 2
Donor1-CATCGAACATGTCCTC-1 1537 5097 9

Donor1-CATCGAAGTCTACCTC-1 8980 47163 1
Donor1-CATCGGGCATATGGTC-1 1708 4175 10
Donor1-CATCGGGGTACCGTTA-1 2046 5407 10
Donor1-CATGACACAAACGTGG-1 2889 6506 3
Donor1-CATGCCTCAGCTGCAC-1 2083 6792 10
Donor1-CATGCCTGTCAGGACA-1 1915 3113 5
Donor1-CATGGCGAGTGATCGG-1 1653 5734 7
Donor1-CATTATCCATGGTCAT-1 1934 9105 8
Donor1-CATTATCTCCTGCTTG-1 2674 5507 3
Donor1-CATTCGCCACCCTATC-1 1538 5658 13
Donor1-CATTCGCCATTCTTAC-1 3363 7862 3
Donor1-CCAATCCAGAATCTCC-1 1457 5146 7
Donor1-CCAATCCCAATAGCAA-1 983 3186 8
Donor1-CCAATCCCACGGTAGA-1 1316 3305 11
Donor1-CCAATCCGTGTTCTTT-1 2493 7012 6
Donor1-CCAATCCTCCACGTTC-1 2039 6239 13
Donor1-CCACCTACAGCTCCGA-1 3191 10324 10
Donor1-CCACCTAGTGCTGTAT-1 4696 13739 2
Donor1-CCACGGAAGCTCCTCT-1 7281 31751 2
Donor1-CCAGCGACAGCTGTGC-1 2645 4971 1
Donor1-CCAGCGATCAACTCTT-1 2664 8486 9
Donor1-CCAGCGATCAATCACG-1 4089 10852 2
Donor1-CCATGTCAGAAACCAT-1 2143 7741 9
Donor1-CCATTCGTCGATCCCT-1 2269 9208 13
Donor1-CCCAATCGTAGCCTAT-1 3393 14144 10
Donor1-CCCAATCGTGTGACGA-1 6347 22713 1
Donor1-CCCAATCTCCAGGGCT-1 1862 5172 11
Donor1-CCCAATCTCTGTCAAG-1 1915 6397 13
Donor1-CCCATACGTTCCACTC-1 1596 4330 9
Donor1-CCGGGATAGGCTAGCA-1 3885 9501 3
Donor1-CCGGGATCAGCAGTTT-1 3121 7309 3
Donor1-CCGGGATTCCTCGCAT-1 3447 8417 3
Donor1-CCGGTAGAGCCCAACC-1 4896 17811 10
Donor1-CCGGTAGCATCTACGA-1 6571 32039 6
Donor1-CCGGTAGGTTCTCATT-1 3533 7379 2
Donor1-CCGTACTAGGTAGCTG-1 3346 11612 10
Donor1-CCGTACTCAACTGCTA-1 3711 9192 3
Donor1-CCGTGGAGTCTAGCCG-1 1637 4906 10
Donor1-CCGTTCACAGCATGAG-1 1701 7507 8
Donor1-CCGTTCAGTTTGTTGG-1 2596 7939 10
Donor1-CCTAAAGCACCTCGTT-1 1631 4803 13
Donor1-CCTAAAGCAGCTGCTG-1 1561 3550 11
Donor1-CCTACACAGCGCCTCA-1 1687 4225 10
Donor1-CCTACACGTATAGGTA-1 3561 8103 5
Donor1-CCTACACGTCACACGC-1 1182 2839 11
Donor1-CCTACCACAGGGTTAG-1 1523 2828 4
Donor1-CCTACCAGTACCCAAT-1 3523 8919 3
Donor1-CCTACCAGTCCCTACT-1 2641 5578 3
Donor1-CCTATTACACAACTGT-1 1344 4281 8
Donor1-CCTCAGTCAAACTGCT-1 4639 12553 5
Donor1-CCTCAGTTCTCTAGGA-1 3255 7949 3
Donor1-CCTCTGAGTGGGTATG-1 3554 8897 3
Donor1-CCTCTGAGTTTGCATG-1 1340 3960 13
Donor1-CCTTACGGTGCAACGA-1 1609 6917 8
Donor1-CCTTACGGTGCAGTAG-1 3037 6033 5
Donor1-CCTTCCCAGATCCCGC-1 2977 9596 11

Donor1-CCTTCCCGTCTCTCGT-1 1727 4136 13
Donor1-CCTTCGAGTGCCTGTG-1 4796 14033 5
Donor1-CCTTCGAGTGTAAGTA-1 1503 3640 10
Donor1-CGAACATCATTTGCCC-1 1566 5186 7
Donor1-CGAATGTAGAGTGACC-1 2736 5850 3
Donor1-CGAATGTAGTACACCT-1 2572 4730 3
Donor1-CGAATGTCACTTGGAT-1 1819 7521 7
Donor1-CGACCTTAGTGTACCT-1 1607 4540 9
Donor1-CGACCTTCACTGCCAG-1 1318 3110 13
Donor1-CGACCTTCATATGGTC-1 2980 6446 3
Donor1-CGACCTTGTGCATCTA-1 1810 4271 11
Donor1-CGACTTCCAGCTGGCT-1 4548 13174 4
Donor1-CGACTTCCATAGTAAG-1 1097 3192 8
Donor1-CGACTTCTCCACGTGG-1 940 3044 8
Donor1-CGACTTCTCTTGTTTG-1 1964 5975 13
Donor1-CGAGAAGGTAAATGAC-1 932 2846 8
Donor1-CGAGCACCAGCTGCTG-1 2555 10499 9
Donor1-CGAGCACTCCTATTCA-1 1612 5216 9
Donor1-CGAGCCACAAGCGATG-1 3230 8081 3
Donor1-CGAGCCACACCGTTGG-1 1778 4642 13
Donor1-CGAGCCACAGGTGGAT-1 1844 4669 11
Donor1-CGAGCCAGTAATCGTC-1 2526 7912 11
Donor1-CGATCGGAGCCGGTAA-1 1799 4885 11
Donor1-CGATCGGTCTACTCAT-1 1192 2993 13
Donor1-CGATGTAAGAGACTTA-1 3083 9807 10
Donor1-CGATTGAGTCTTCTCG-1 3989 14093 10
Donor1-CGCCAAGAGTATTGGA-1 2046 5254 10
Donor1-CGCCAAGGTGATGTGG-1 1890 4872 11
Donor1-CGCGTTTTCTCTTATG-1 5568 25200 10
Donor1-CGCTATCAGCCCTAAT-1 3493 12293 11
Donor1-CGCTATCTCCTATGTT-1 1350 2914 11
Donor1-CGCTATCTCTTACCTA-1 2400 7930 10
Donor1-CGCTGGACATAAGACA-1 1816 5975 13
Donor1-CGCTTCAGTAAGGGAA-1 1906 7062 7
Donor1-CGCTTCATCTAACTGG-1 1316 3721 8
Donor1-CGGACACCAGACAGGT-1 2301 6968 10
Donor1-CGGACACTCAGCAACT-1 1258 4240 8
Donor1-CGGACGTCACCAACCG-1 5757 37781 10
Donor1-CGGAGCTCATACAGCT-1 4016 16196 10
Donor1-CGGAGCTGTATCTGCA-1 2895 5735 2
Donor1-CGGAGTCAGAGCTGGT-1 2294 12534 8
Donor1-CGGAGTCGTCCCTTGT-1 2107 4058 3
Donor1-CGGAGTCTCCTTGGTC-1 3707 9385 3
Donor1-CGGCTAGCAGGGCATA-1 1522 5146 13
Donor1-CGGCTAGTCCTAGAAC-1 1536 6303 8
Donor1-CGGGTCAGTCTAGTCA-1 2406 4881 4
Donor1-CGGTTAACAAGGACAC-1 4348 10952 5
Donor1-CGGTTAACAGATGGGT-1 1775 5510 9
Donor1-CGTAGCGAGAAGGGTA-1 1605 3330 11
Donor1-CGTAGCGAGTGAACAT-1 1724 4464 11
Donor1-CGTAGCGCAGCTGTTA-1 1536 2892 2
Donor1-CGTAGCGGTAATCACC-1 5791 17399 2
Donor1-CGTAGCGGTAGAGGAA-1 1641 3833 11
Donor1-CGTAGCGGTCCAGTAT-1 969 2887 8
Donor1-CGTAGCGGTGTTGGGA-1 3666 15282 10
Donor1-CGTAGCGTCCAGAGGA-1 1166 4202 9

Donor1-CGTAGCGTCCTCAACC-1 2084 5985 10
Donor1-CGTAGGCAGGCTAGGT-1 2637 10027 10
Donor1-CGTAGGCGTTTGTGTG-1 1670 4099 10
Donor1-CGTAGGCTCACTTATC-1 3473 8084 3
Donor1-CGTAGGCTCTAACTCT-1 1449 5955 8
Donor1-CGTCACTCAAACGCGA-1 2617 5770 3
Donor1-CGTCAGGTCGCTTGTC-1 3414 12396 12
Donor1-CGTCTACAGGCCGAAT-1 2510 4769 2
Donor1-CGTCTACCAGCCTTGG-1 1590 6517 7
Donor1-CGTCTACGTCCGCTGA-1 1472 4304 9
Donor1-CGTGAGCGTTACGGAG-1 2466 6739 11
Donor1-CGTGTCTGTTCTGGTA-1 1333 4118 9
Donor1-CGTGTCTTCAGCGACC-1 2804 6231 3
Donor1-CGTTAGAAGCATGGCA-1 3000 5933 5
Donor1-CGTTCTGGTAGCAAAT-1 3658 8709 3
Donor1-CGTTCTGTCATGTCTT-1 1568 5616 8
Donor1-CGTTGGGAGGGTGTGT-1 1896 6227 9
Donor1-CGTTGGGCAATCAGAA-1 3297 12085 11
Donor1-CGTTGGGCACTCAGGC-1 4219 11894 3
Donor1-CGTTGGGTCCCAGGTG-1 1264 3070 13
Donor1-CTAACTTGTCGTCTTC-1 3372 7828 2
Donor1-CTAAGACCACAGTCGC-1 1801 3215 5
Donor1-CTAAGACGTCAAAGCG-1 2531 5605 3
Donor1-CTAAGACGTTTCCACC-1 1763 4948 9
Donor1-CTAAGACTCATCTGTT-1 2004 6519 9
Donor1-CTAATGGCAATGGATA-1 2008 6861 9
Donor1-CTAATGGGTGTCAATC-1 1599 4456 10
Donor1-CTAATGGTCACCCTCA-1 1387 2893 13
Donor1-CTACACCAGACGCTTT-1 1370 3746 8
Donor1-CTACATTCAGTACACT-1 3887 9111 2
Donor1-CTACATTCATGAACCT-1 1756 5959 9
Donor1-CTACGTCGTAGCTAAA-1 1788 4753 10
Donor1-CTACGTCGTTGCTCCT-1 951 3001 8
Donor1-CTACGTCTCATTATCC-1 5078 15839 1
Donor1-CTAGAGTCACCACGTG-1 7428 33472 2
Donor1-CTAGAGTTCTATCCCG-1 1472 3163 11
Donor1-CTAGCCTCAAGCGAGT-1 2074 5979 6
Donor1-CTAGCCTCAGCTGCAC-1 2781 7749 10
Donor1-CTAGCCTGTGGCTCCA-1 1246 3399 13
Donor1-CTAGTGATCAGCAACT-1 2835 10438 6
Donor1-CTAGTGATCGCTGATA-1 1186 4240 8
Donor1-CTCACACCACAGAGGT-1 1581 4259 13
Donor1-CTCACACCAGTCAGCC-1 1374 4188 9
Donor1-CTCAGAAAGCCAGAAC-1 2958 6474 5
Donor1-CTCAGAATCTAACCGA-1 1616 4656 10
Donor1-CTCATTAAGTGTCCAT-1 2230 3750 5
Donor1-CTCATTACACTGCCAG-1 2506 7867 11
Donor1-CTCATTATCGGCGCTA-1 1134 3463 8
Donor1-CTCATTATCTACCAGA-1 2322 5556 6
Donor1-CTCCTAGAGCTCCCAG-1 2982 6383 3
Donor1-CTCGAAAAGAAGAAGC-1 1157 2832 10
Donor1-CTCGAAACACCAACCG-1 7266 32479 4
Donor1-CTCGAAAGTTATCCGA-1 1664 5492 13
Donor1-CTCGAGGAGACGACGT-1 1696 2939 3
Donor1-CTCGAGGAGCGTTGCC-1 1939 3790 3
Donor1-CTCGAGGGTGGTCCGT-1 1519 4342 10

Donor1-CTCGGGAAGGCCCTCA-1 1887 9344 8
Donor1-CTCGTACGTTACCGAT-1 4034 20448 10
Donor1-CTCGTCAAGCTGCGAA-1 2944 7204 3
Donor1-CTCGTCACAGCGATCC-1 1441 3366 11
Donor1-CTCGTCACATCCTAGA-1 2478 13741 8
Donor1-CTCTAATAGACCTAGG-1 1911 10049 8
Donor1-CTCTAATAGTAAGTAC-1 1762 3351 2
Donor1-CTCTAATAGTACGCCC-1 2462 7390 10
Donor1-CTCTAATAGTCACGCC-1 2879 14067 7
Donor1-CTCTAATCACTACAGT-1 1204 3338 9
Donor1-CTCTAATGTCCTCCAT-1 1862 4244 6
Donor1-CTCTAATTCGAATCCA-1 1867 6320 13
Donor1-CTCTACGAGCTACCGC-1 1074 3705 8
Donor1-CTCTACGAGGACATTA-1 2097 8180 8
Donor1-CTCTACGGTATGAATG-1 1907 4194 11
Donor1-CTCTGGTGTATGAATG-1 2199 7270 13
Donor1-CTCTGGTGTCTCTCGT-1 2394 6944 13
Donor1-CTGAAACTCATGCAAC-1 1666 3603 11
Donor1-CTGAAGTGTCCAGTAT-1 3001 14305 7
Donor1-CTGAAGTTCCAGAGGA-1 1386 3455 13
Donor1-CTGAAGTTCGAATGGG-1 1422 2922 11
Donor1-CTGATAGTCTTTAGTC-1 1277 3951 8
Donor1-CTGATCCAGAAACCTA-1 4199 13954 10
Donor1-CTGATCCCAAGTCTAC-1 2724 6926 11
Donor1-CTGCCTAAGTGTGAAT-1 1381 3186 11
Donor1-CTGCGGATCGTCCGTT-1 3192 7907 3
Donor1-CTGGTCTCAAGTTGTC-1 2050 3781 3
Donor1-CTGGTCTGTCAACTGT-1 1541 4096 7
Donor1-CTGGTCTGTGGTAACG-1 4574 17383 10
Donor1-CTGTGCTAGAAACGAG-1 1575 3566 11
Donor1-CTGTGCTAGTTCGATC-1 1192 3289 7
Donor1-CTGTTTATCGCTAGCG-1 2659 8236 13
Donor1-CTTAACTAGAATAGGG-1 2213 5895 6
Donor1-CTTAACTCAAACGTGG-1 1466 3813 13
Donor1-CTTAACTCAGGCTGAA-1 7741 42863 2
Donor1-CTTACCGAGAGTTGGC-1 1185 3142 10
Donor1-CTTACCGCACGCGAAA-1 6137 23527 1
Donor1-CTTACCGCAGCGTAAG-1 3674 16139 10
Donor1-CTTACCGTCGAGCCCA-1 1781 9269 8
Donor1-CTTACCGTCTTAACCT-1 4965 25279 10
Donor1-CTTAGGAGTGATAAAC-1 3937 9956 1
Donor1-CTTAGGAGTTGCTCCT-1 1065 2832 7
Donor1-CTTAGGATCAGTGCAT-1 1554 4319 13
Donor1-CTTCTCTCAGCGTAAG-1 5605 18407 2
Donor1-CTTCTCTGTTCACGGC-1 1871 5120 13
Donor1-CTTTGCGAGGCTCATT-1 2959 6804 6
Donor1-CTTTGCGAGTGCTGCC-1 3015 9805 11
Donor1-GAAACTCAGGACATTA-1 1129 3180 7
Donor1-GAAACTCAGGCCCTTG-1 4337 13206 1
Donor1-GAAACTCCAACTGCGC-1 2426 7399 13
Donor1-GAAACTCGTCATCCCT-1 1542 3426 13
Donor1-GAAACTCGTCTCATCC-1 3235 18658 7
Donor1-GAAACTCTCCTGTACC-1 1143 3348 9
Donor1-GAAACTCTCTATCCTA-1 2029 8171 7
Donor1-GAAATGACACGCATCG-1 1610 7003 8
Donor1-GAAATGAGTGCGAAAC-1 3669 8833 4

Donor1-GAAATGATCATGCAAC-1 1756 5415 9
Donor1-GAACATCAGTGGGCTA-1 1378 3465 10
Donor1-GAACATCCAAGCCGCT-1 3441 8022 3
Donor1-GAACATCGTACATCCA-1 1927 5341 13
Donor1-GAACATCGTTTCCACC-1 1109 3265 8
Donor1-GAACATCGTTTGACTG-1 2526 4600 6
Donor1-GAACCTATCCTAGTGA-1 1407 4000 9
Donor1-GAACGGAAGTGGAGTC-1 1211 3529 7
Donor1-GAAGCAGTCGTGGACC-1 2301 8366 10
Donor1-GAATAAGAGAGGACGG-1 3108 8275 6
Donor1-GAATAAGAGTCAAGGC-1 6352 21145 2
Donor1-GAATAAGGTCTGATCA-1 1923 4923 13
Donor1-GAATGAAAGTTTGCGT-1 3011 10170 11
Donor1-GACACGCCAATCTGCA-1 1429 4383 7
Donor1-GACCAATTCTTGCAAG-1 1727 4958 13
Donor1-GACCTGGAGAGACTTA-1 3528 8685 3
Donor1-GACCTGGAGCATGGCA-1 1684 4427 13
Donor1-GACCTGGCAGGGTATG-1 1230 3876 8
Donor1-GACCTGGCAGTCAGCC-1 3499 8687 3
Donor1-GACGCGTGTTATCGGT-1 6412 24719 2
Donor1-GACGTGCAGTGTCCCG-1 1579 5003 9
Donor1-GACGTGCCAAAGGTGC-1 5311 19933 1
Donor1-GACGTTAAGAATTCCC-1 5190 17089 1
Donor1-GACGTTACAGCCAATT-1 8155 48781 1
Donor1-GACGTTACAGCGTCCA-1 1907 3733 6
Donor1-GACTAACAGTCACGCC-1 2208 8611 7
Donor1-GACTAACGTACTCGCG-1 4360 19643 4
Donor1-GACTAACTCCACGTTC-1 1642 4239 10
Donor1-GACTACAAGCGTCTAT-1 1408 3499 13
Donor1-GACTACAAGCGTGTCC-1 2075 5951 10
Donor1-GACTACAAGTTCGCAT-1 2116 6867 13
Donor1-GACTACAGTGCTAGCC-1 1400 2955 13
Donor1-GACTGCGAGGATTCGG-1 2244 7258 10
Donor1-GAGCAGACACAACGTT-1 2214 7338 10
Donor1-GAGCAGACAGTTCCCT-1 2930 6189 3
Donor1-GAGTCCGAGTACATGA-1 1475 3745 13
Donor1-GAGTCCGTCGCCTGAG-1 1278 3030 11
Donor1-GATCAGTAGCCAGGAT-1 1324 4351 9
Donor1-GATCAGTAGGGAGTAA-1 2281 6584 11
Donor1-GATCAGTAGTGCGATG-1 1251 4460 7
Donor1-GATCAGTCATATGCTG-1 1679 4130 10
Donor1-GATCGATCAGCTATTG-1 1746 6293 7
Donor1-GATCGATGTCCATGAT-1 3456 8342 3
Donor1-GATCGATGTGTTGAGG-1 4119 11991 3
Donor1-GATCGCGTCGACAGCC-1 7270 34622 3
Donor1-GATCGTACAGTCTTCC-1 3072 16912 7
Donor1-GATGAAAAGCACAGGT-1 2004 5591 10
Donor1-GATGAAAAGCTGGAAC-1 1614 4791 9
Donor1-GATGAAAGTGTGAATA-1 1121 2866 13
Donor1-GATGAGGAGGCCCTCA-1 3201 11604 11
Donor1-GATGAGGTCAGGCGAA-1 1420 3100 11
Donor1-GATGAGGTCGCCTGTT-1 1898 6175 10
Donor1-GATGCTAAGGCTAGCA-1 1200 2969 11
Donor1-GATTCAGCATGAGCGA-1 2180 7347 10
Donor1-GATTCAGGTCAGGACA-1 3530 7881 6
Donor1-GCAAACTAGGTTACCT-1 1341 3334 10

Donor1-GCAAACTTCTCAAACG-1 2457 8924 9
Donor1-GCAATCAAGATCCTGT-1 1985 3668 2
Donor1-GCAATCACACAAGCCC-1 1516 3632 13
Donor1-GCAATCACAGAGTGTG-1 3125 6567 1
Donor1-GCAATCACATTGCGGC-1 3575 11628 11
Donor1-GCACATAGTCAACTGT-1 2231 6547 11
Donor1-GCACTCTAGGTACTCT-1 5855 18674 2
Donor1-GCACTCTTCCCAAGTA-1 2520 13532 8
Donor1-GCACTCTTCGCTTAGA-1 2293 8055 13
Donor1-GCAGCCACAGCTCGCA-1 1540 3407 10
Donor1-GCAGTTAAGATGCCAG-1 4153 11647 6
Donor1-GCAGTTAAGTGTACGG-1 1023 3086 8
Donor1-GCATACAGTCGCGGTT-1 2293 10440 8
Donor1-GCATACATCTCCTATA-1 1276 3947 7
Donor1-GCATGATAGAGCTATA-1 2017 4617 11
Donor1-GCATGATAGCTTCGCG-1 2500 6475 6
Donor1-GCATGATAGTATCGAA-1 1716 5154 13
Donor1-GCATGATCATCCGGGT-1 1621 4771 13
Donor1-GCATGATGTCATACTG-1 2559 8172 6
Donor1-GCCAAATAGCGATAGC-1 1683 6212 8
Donor1-GCCAAATCAAGCCTAT-1 3260 7368 3
Donor1-GCCTCTAGTCTGGTCG-1 1933 7745 7
Donor1-GCCTCTATCAAAGACA-1 2723 5374 2
Donor1-GCGACCATCGTAGGTT-1 1561 4672 8
Donor1-GCGAGAAAGCGACGTA-1 2219 8399 7
Donor1-GCGAGAACATTGTGCA-1 4028 10700 1
Donor1-GCGAGAAGTTCGCGAC-1 2129 6935 10
Donor1-GCGAGAATCATGCATG-1 6177 23465 5
Donor1-GCGCAACAGCGTCTAT-1 6468 23893 3
Donor1-GCGCAACGTGGCAAAC-1 1489 5813 8
Donor1-GCGCCAAAGACTCGGA-1 2057 5254 6
Donor1-GCGCCAAAGTATGACA-1 1076 3604 8
Donor1-GCGCCAACAATGAAAC-1 2002 5324 10
Donor1-GCGCCAAGTAGGCATG-1 1586 4309 13
Donor1-GCGCCAAGTGCAGGTA-1 2451 7367 13
Donor1-GCGCCAATCTCATTCA-1 2490 7765 10
Donor1-GCGCGATCAAGGACAC-1 3690 8742 3
Donor1-GCGGGTTGTGATGCCC-1 3310 11456 11
Donor1-GCGGGTTGTTCCGTCT-1 1765 5083 13
Donor1-GCTCCTAAGCGAGAAA-1 1973 7806 9
Donor1-GCTCCTATCATCGCTC-1 5125 16811 3
Donor1-GCTCTGTAGCGGATCA-1 2441 7931 10
Donor1-GCTCTGTGTACTCGCG-1 1823 4488 7
Donor1-GCTCTGTGTGAACCTT-1 3131 7198 3
Donor1-GCTCTGTGTGCACTTA-1 2540 8443 10
Donor1-GCTCTGTGTTACGACT-1 2154 7202 10
Donor1-GCTCTGTGTTTACTCT-1 1629 5807 8
Donor1-GCTGCGAAGATGCGAC-1 6527 28357 6
Donor1-GCTGCGACAGCTGTGC-1 2968 15185 7
Donor1-GCTGCGATCGCAAACT-1 1261 3363 9
Donor1-GCTGCTTAGTTTCCTT-1 2392 6188 11
Donor1-GCTGCTTGTAAGCACG-1 1664 6050 13
Donor1-GCTGCTTTCCATTCTA-1 4604 12359 5
Donor1-GCTGCTTTCGTTTGCC-1 1165 3558 8
Donor1-GCTGCTTTCTGGAGCC-1 1471 3690 10
Donor1-GCTGGGTGTTCCGTCT-1 1376 5327 8

Donor1-GCTGGGTTCCAGGGCT-1 4116 11074 1
Donor1-GCTTCCAAGGACAGAA-1 2643 5476 3
Donor1-GCTTGAACACACCGCA-1 1404 5538 8
Donor1-GCTTGAATCGAGCCCA-1 3837 8659 3
Donor1-GCTTGAATCTTTACAC-1 2221 6476 9
Donor1-GGAAAGCCAGACGCAA-1 3534 8538 6
Donor1-GGAAAGCCATCTCCCA-1 3552 9236 3
Donor1-GGAAAGCTCGAATGGG-1 1900 5402 10
Donor1-GGAACTTTCAGTGTTG-1 1861 6704 7
Donor1-GGAATAACAGATCGGA-1 1116 3810 8
Donor1-GGAATAAGTTGGGACA-1 2605 12801 7
Donor1-GGAATAATCACTCCTG-1 1654 4204 13
Donor1-GGACAAGAGGACAGCT-1 1042 3891 8
Donor1-GGACAGACAAGTCTAC-1 6403 22967 3
Donor1-GGACATTAGACTAGAT-1 2299 5890 10
Donor1-GGACATTAGCTCTCGG-1 2709 7462 4
Donor1-GGACATTAGGAGTCTG-1 1774 7183 7
Donor1-GGACATTCATTATCTC-1 1742 6600 7
Donor1-GGACATTTCCACGACG-1 1500 5088 7
Donor1-GGACGTCCAATGGTCT-1 1129 3693 8
Donor1-GGACGTCGTAAACGCG-1 1263 3842 13
Donor1-GGACGTCTCGAACTGT-1 2110 7698 13
Donor1-GGACGTCTCGGAAACG-1 1643 5275 9
Donor1-GGAGCAATCTGAAAGA-1 3389 11795 11
Donor1-GGATGTTAGTTGTAGA-1 1998 7147 7
Donor1-GGATGTTTCCCAACGG-1 1704 5003 10
Donor1-GGATTACCAGCTGTAT-1 4617 13263 2
Donor1-GGATTACCAGTTCATG-1 2038 3951 2
Donor1-GGCAATTAGAGGTTGC-1 2942 7085 5
Donor1-GGCAATTAGCTGTCTA-1 2811 8696 11
Donor1-GGCAATTGTGCAACTT-1 1470 5280 13
Donor1-GGCAATTGTGTGAAAT-1 2748 6165 3
Donor1-GGCCGATGTCGGCACT-1 3132 7177 3
Donor1-GGCCGATTCCTCAACC-1 1739 4798 11
Donor1-GGCCGATTCGGAATCT-1 2597 8814 6
Donor1-GGCGACTAGGCTCATT-1 1541 3499 11
Donor1-GGCGACTTCTTGTTTG-1 2695 9928 10
Donor1-GGCGTGTAGCCCAACC-1 1470 7312 8
Donor1-GGCGTGTAGTGGGCTA-1 3295 11751 6
Donor1-GGCGTGTAGTTCGATC-1 2850 6554 3
Donor1-GGCGTGTGTCGAGTTT-1 1355 3606 10
Donor1-GGCTCGAGTCACTGGC-1 2257 5093 5
Donor1-GGCTCGAGTGAGGCTA-1 4143 10197 3
Donor1-GGCTCGATCCTAGAAC-1 1513 4220 8
Donor1-GGCTCGATCGATGAGG-1 4636 13275 3
Donor1-GGCTGGTGTCAGCTAT-1 2713 5955 3
Donor1-GGCTGGTTCAAGAAGT-1 2785 9281 10
Donor1-GGGAATGAGACCTTTG-1 1522 3633 7
Donor1-GGGAATGCAATTCCTT-1 3451 8281 3
Donor1-GGGAATGCAGCTCGAC-1 1299 4735 8
Donor1-GGGAATGTCACTTCAT-1 1908 5601 13
Donor1-GGGAATGTCTGAAAGA-1 3952 15622 6
Donor1-GGGACCTAGCGTGAAC-1 3305 9843 6
Donor1-GGGACCTCAGGGTTAG-1 3172 10057 6
Donor1-GGGAGATTCGGTTAAC-1 4482 13283 1
Donor1-GGGATGAAGCTATGCT-1 882 3010 8

Donor1-GGGATGAAGGCATGTG-1 2044 8057 9
Donor1-GGGATGAGTGAGCGAT-1 2008 5573 10
Donor1-GGGATGATCCTCAATT-1 1518 5396 8
Donor1-GGGCACTGTCTTGTCC-1 4251 21116 10
Donor1-GGGCATCTCAGCTCGG-1 2443 6916 11
Donor1-GGGTTGCAGTAATCCC-1 2250 7284 6
Donor1-GGGTTGCCACGTCAGC-1 1184 2827 13
Donor1-GGTATTGAGAGACTTA-1 1295 2900 10
Donor1-GGTATTGCAAGAGGCT-1 1874 3307 6
Donor1-GGTATTGGTGCCTGGT-1 3045 13544 7
Donor1-GGTGCGTAGCGTAATA-1 1370 2853 11
Donor1-GGTGTTAGTCATTAGC-1 2594 5221 2
Donor1-GGTGTTAGTCTTTCAT-1 3797 23836 7
Donor1-GTAACGTCACAGATTC-1 2227 7041 9
Donor1-GTAACTGCAGTGGGAT-1 2795 5780 3
Donor1-GTAACTGGTTGGTGGA-1 4648 12671 1
Donor1-GTAACTGTCTTGAGGT-1 5990 41729 10
Donor1-GTACGTAAGTTAACGA-1 2472 5082 5
Donor1-GTACGTACAGATGAGC-1 1973 7666 9
Donor1-GTACGTATCCAAGCCG-1 1836 5575 13
Donor1-GTACGTATCTGAGGGA-1 1241 3151 9
Donor1-GTACTTTGTTCCACAA-1 2870 5912 2
Donor1-GTACTTTTCGGAAATA-1 1636 4511 13
Donor1-GTAGGCCAGCGCCTCA-1 8534 51533 6
Donor1-GTAGGCCCAGCTGTTA-1 4876 12597 2
Donor1-GTAGGCCGTGCAACGA-1 6887 34228 4
Donor1-GTAGTCACAAGTCTGT-1 1606 4465 13
Donor1-GTAGTCAGTATAGTAG-1 2215 4985 11
Donor1-GTAGTCATCAAAGACA-1 6683 26628 2
Donor1-GTAGTCATCCGAACGC-1 5621 20329 3
Donor1-GTATCTTAGCTGTTCA-1 1532 3692 13
Donor1-GTATCTTAGGCATGGT-1 2381 7647 6
Donor1-GTATCTTGTCAGCTAT-1 4522 11916 1
Donor1-GTATTCTAGAGATGAG-1 7423 35414 5
Donor1-GTATTCTCAGTTAACC-1 4856 26124 10
Donor1-GTATTCTGTCGATTGT-1 5943 22392 1
Donor1-GTATTCTGTGTTTGGT-1 896 3003 8
Donor1-GTATTCTTCGACGGAA-1 2320 7299 10
Donor1-GTCACAAAGTGTTAGA-1 3819 12629 10
Donor1-GTCACGGGTCCTCTTG-1 2156 3777 5
Donor1-GTCATTTAGTGTTAGA-1 3834 14850 10
Donor1-GTCATTTCACAGACAG-1 1194 3822 8
Donor1-GTCATTTGTTCCAACA-1 1096 3809 8
Donor1-GTCCTCACACAGCCCA-1 2688 5996 6
Donor1-GTCCTCACAGTAGAGC-1 2606 9226 13
Donor1-GTCCTCATCTTGAGAC-1 2711 10902 7
Donor1-GTCTCGTCAGACGCTC-1 1905 3462 2
Donor1-GTCTCGTTCACCTTAT-1 5179 16504 4
Donor1-GTCTTCGCATACTACG-1 4311 20182 10
Donor1-GTCTTCGTCAGTTCGA-1 1326 3364 10
Donor1-GTGAAGGGTACCGAGA-1 3459 7752 5
Donor1-GTGAAGGGTCTCCATC-1 2137 7291 13
Donor1-GTGAAGGTCTGAGTGT-1 1662 4365 10
Donor1-GTGCAGCGTCCATCCT-1 1231 3513 13
Donor1-GTGCAGCTCCGAATGT-1 1515 5352 8
Donor1-GTGCAGCTCTGGTATG-1 1394 3451 11

Donor1-GTGCATAAGATGCCAG-1 1979 3739 5
Donor1-GTGCATAGTACAGCAG-1 1007 3353 8
Donor1-GTGCATATCCACTGGG-1 1547 7034 8
Donor1-GTGCTTCCACAGCGTC-1 2736 5475 6
Donor1-GTGGGTCGTATAATGG-1 1355 4751 8
Donor1-GTGGGTCTCTGACCTC-1 1432 3765 13
Donor1-GTGTGCGAGATTACCC-1 1103 2919 13
Donor1-GTGTGCGGTGGAAAGA-1 1013 3027 8
Donor1-GTGTTAGCAGTACACT-1 3399 11358 11
Donor1-GTGTTAGCATACAGCT-1 1845 5152 13
Donor1-GTGTTAGCATTGCGGC-1 5031 16605 1
Donor1-GTGTTAGGTAGAAGGA-1 1834 6571 9
Donor1-GTGTTAGGTCATGCCG-1 1217 3780 9
Donor1-GTGTTAGGTGGGTATG-1 3911 28301 7
Donor1-GTGTTAGTCGCCTGTT-1 1294 3170 11
Donor1-GTTAAGCGTAGCACGA-1 1660 5816 7
Donor1-GTTAAGCGTTGTTTGG-1 2085 8141 9
Donor1-GTTAAGCGTTTGACTG-1 1022 2901 8
Donor1-GTTACAGCAAAGGTGC-1 2349 11343 8
Donor1-GTTACAGCAGCTTAAC-1 1868 5432 13
Donor1-GTTACAGTCTTGGGTA-1 2650 8923 10
Donor1-GTTCATTGTGGCAAAC-1 2158 6390 10
Donor1-GTTCGGGAGCTAAGAT-1 1251 4352 8
Donor1-GTTCGGGAGGCGTACA-1 1516 4193 9
Donor1-GTTCGGGCACTCTGTC-1 3183 7405 3
Donor1-GTTCGGGCATGGATGG-1 2779 6093 3
Donor1-GTTCGGGGTCTCTTTA-1 4327 10851 3
Donor1-GTTCGGGGTGAACCTT-1 4157 11271 6
Donor1-GTTTCTAAGGTTACCT-1 2279 8307 9
Donor1-TAAACCGTCACATACG-1 1549 3199 11
Donor1-TAAGAGAGTAAGTTCC-1 1870 6938 9
Donor1-TAAGAGATCTCAAACG-1 1145 2985 9
Donor1-TAAGCGTCACGTCAGC-1 3131 11307 6
Donor1-TAAGCGTGTGGACGAT-1 1963 5970 13
Donor1-TAAGCGTTCCTATTCA-1 3117 12684 10
Donor1-TACACGAAGCGGCTTC-1 2334 6286 6
Donor1-TACACGACATGAACCT-1 2725 8230 10
Donor1-TACACGAGTGGACGAT-1 4930 16188 2
Donor1-TACACGATCTAACCGA-1 3644 8317 3
Donor1-TACCTATCATGGGACA-1 3384 7965 2
Donor1-TACCTATTCCAGATCA-1 2315 3776 5
Donor1-TACCTATTCGCTGATA-1 2526 8490 10
Donor1-TACCTTAGTAAACCTC-1 2536 7915 10
Donor1-TACCTTATCTAACTGG-1 5190 21026 6
Donor1-TACCTTATCTGGCGAC-1 1358 3380 13
Donor1-TACGGGCCAATCAGAA-1 1269 4447 7
Donor1-TACGGGCCATCGGACC-1 2609 5166 2
Donor1-TACGGGCGTTCGCGAC-1 4197 10678 5
Donor1-TACGGGCTCTCTGCTG-1 6764 25398 2
Donor1-TACGGTAAGTAGCCGA-1 6866 34719 5
Donor1-TACGGTAGTAAGGGCT-1 4920 14939 2
Donor1-TACTCATCAAGAGTCG-1 1514 4515 13
Donor1-TACTCATTCCGAACGC-1 1377 5032 8
Donor1-TACTCGCAGCACCGCT-1 1462 2857 11
Donor1-TACTCGCGTTCTGGTA-1 1048 3622 8
Donor1-TACTTACCATGAGCGA-1 3459 7851 2

Donor1-TACTTACTCCACGACG-1 4702 14821 6
Donor1-TACTTACTCCCATTAT-1 4184 12544 6
Donor1-TACTTGTAGCTTTGGT-1 5910 24056 1
Donor1-TACTTGTTCACGCGGT-1 6413 21713 3
Donor1-TAGACCACATGGGAAC-1 2637 11248 7
Donor1-TAGACCAGTGACGGTA-1 2075 5202 10
Donor1-TAGAGCTGTGCTCTTC-1 1473 3162 11
Donor1-TAGAGCTTCGCTTAGA-1 1794 4978 13
Donor1-TAGAGCTTCTGGGCCA-1 1850 5348 10
Donor1-TAGCCGGCACTAGTAC-1 3603 9587 3
Donor1-TAGCCGGGTACTTGAC-1 1789 7865 8
Donor1-TAGCCGGGTTTAAGCC-1 1630 3832 11
Donor1-TAGTTGGAGGCGTACA-1 3712 11770 11
Donor1-TAGTTGGAGGTGTGGT-1 1422 5332 8
Donor1-TAGTTGGCACATTTCT-1 1900 5935 13
Donor1-TAGTTGGTCACCATAG-1 4970 14205 2
Donor1-TATGCCCCAACTTGAC-1 1313 5714 8
Donor1-TATGCCCTCGTTGACA-1 1547 3664 11
Donor1-TATTACCAGCTGAACG-1 1645 5187 9
Donor1-TCAACGATCGCGTTTC-1 1718 3976 11
Donor1-TCAATCTCACACGCTG-1 3576 12627 11
Donor1-TCAATCTTCACCGTAA-1 1791 4784 13
Donor1-TCACAAGAGAGTAAGG-1 1496 3184 10
Donor1-TCACAAGGTGTAAGTA-1 1624 2826 8
Donor1-TCACGAAAGACTAGAT-1 1778 5861 13
Donor1-TCACGAATCAGCTCGG-1 1363 3299 11
Donor1-TCAGATGGTCCGAACC-1 3116 11989 10
Donor1-TCAGATGGTTTCGCTC-1 2817 6345 3
Donor1-TCAGATGTCGTCCGTT-1 1526 6904 8
Donor1-TCAGATGTCTTGAGGT-1 3247 12066 10
Donor1-TCAGCAAGTCGCATCG-1 3003 13006 9
Donor1-TCAGCAATCAGCACAT-1 2246 6323 13
Donor1-TCAGCAATCATAGCAC-1 1430 3034 11
Donor1-TCAGCTCGTAAGGGAA-1 3427 8923 3
Donor1-TCAGGATGTCAGAGGT-1 1826 5697 13
Donor1-TCAGGATGTTCTGTTT-1 4262 12708 3
Donor1-TCAGGTAAGAAGCCCA-1 1405 4193 11
Donor1-TCAGGTATCTGACCTC-1 3246 10682 11
Donor1-TCATTTGCAGAAGCAC-1 1702 5610 9
Donor1-TCATTTGTCTTGGGTA-1 1299 3719 8
Donor1-TCCACACCAAGTAATG-1 1184 3625 9
Donor1-TCCACACCAATGCCAT-1 2622 11067 10
Donor1-TCCACACTCCGTAGTA-1 8210 52299 4
Donor1-TCCCGATAGCGCTCCA-1 1579 3748 11
Donor1-TCCCGATGTACAGCAG-1 1477 4379 8
Donor1-TCCCGATGTTAAGAAC-1 3233 7275 3
Donor1-TCCCGATTCGAACGGA-1 3067 7204 3
Donor1-TCGAGGCAGTCATCCA-1 1158 3002 6
Donor1-TCGAGGCCAGTTTACG-1 1410 2926 11
Donor1-TCGAGGCGTAAATACG-1 1792 5095 13
Donor1-TCGAGGCGTTCGTCTC-1 3456 7381 5
Donor1-TCGAGGCTCTCAAGTG-1 4833 28569 10
Donor1-TCGAGGCTCTGCTGTC-1 1962 5410 10
Donor1-TCGCGAGGTCTGCCAG-1 1377 2839 11
Donor1-TCGCGAGTCTTCATGT-1 1669 3791 11
Donor1-TCGCGTTAGCATCATC-1 1883 6741 13

Donor1-TCGCGTTTCACGCATA-1 5162 14395 2
Donor1-TCGCGTTTCATGTGGT-1 2774 6190 3
Donor1-TCGGGACGTCTAGAGG-1 1408 4280 7
Donor1-TCGGGACTCGCCGTGA-1 1315 4303 7
Donor1-TCGGTAAAGCACGCCT-1 2448 4921 3
Donor1-TCGTACCAGGAGTTTA-1 1375 4407 8
Donor1-TCGTACCCAGCTGCTG-1 2782 7688 11
Donor1-TCGTACCCATTACCTT-1 2221 8532 9
Donor1-TCGTACCCATTATCTC-1 1633 4438 13
Donor1-TCGTACCGTAAATGAC-1 2691 11035 10
Donor1-TCGTACCTCACGACTA-1 1016 3174 8
Donor1-TCGTAGATCATCGCTC-1 1529 4036 10
Donor1-TCTATTGAGTACATGA-1 8438 71267 10
Donor1-TCTATTGGTATTCGTG-1 2910 6679 3
Donor1-TCTATTGGTTCTGTTT-1 2230 6990 10
Donor1-TCTCATAAGGCGACAT-1 1188 4108 8
Donor1-TCTCATAGTAGCTCCG-1 1526 4900 13
Donor1-TCTCTAAAGATGAGAG-1 1813 5498 13
Donor1-TCTGAGAAGCTAGCCC-1 3255 8115 3
Donor1-TCTGAGAAGGGTATCG-1 1953 5203 10
Donor1-TCTGAGAAGTTCGATC-1 2310 4572 5
Donor1-TCTGGAAGTAGCGCTC-1 5872 30193 10
Donor1-TCTTCGGAGACTTTCG-1 2307 6283 13
Donor1-TCTTTCCAGCACCGCT-1 1727 5375 13
Donor1-TGAAAGACAGCATGAG-1 4758 20951 10
Donor1-TGACAACAGCCAGTTT-1 2071 7024 7
Donor1-TGACAACAGCTAACTC-1 1332 5074 8
Donor1-TGACAACCACATGTGT-1 1336 3884 9
Donor1-TGACGGCCAGCTGTAT-1 5400 16133 1
Donor1-TGACTAGGTCTTGTCC-1 1329 2909 13
Donor1-TGACTTTCACCTTGTC-1 1324 4199 9
Donor1-TGACTTTGTGCACGAA-1 3114 9744 11
Donor1-TGACTTTGTTTACTCT-1 4238 10476 3
Donor1-TGACTTTTCAGTTCGA-1 2610 17175 8
Donor1-TGACTTTTCATTGCCC-1 1437 3744 13
Donor1-TGAGCATCAAACAACA-1 2638 5935 3
Donor1-TGAGCATTCCAAGCCG-1 1153 3375 8
Donor1-TGAGCATTCCAGGGCT-1 1956 6061 10
Donor1-TGAGCCGAGTGCCATT-1 1167 3699 8
Donor1-TGAGCCGGTACATGTC-1 2121 6606 11
Donor1-TGAGGGAAGAGGGATA-1 3301 10044 11
Donor1-TGAGGGAAGGACAGCT-1 3696 9482 3
Donor1-TGAGGGAAGTGTACGG-1 1396 3689 10
Donor1-TGAGGGACAACGCACC-1 3803 31921 8
Donor1-TGAGGGAGTCAGAATA-1 1885 4975 13
Donor1-TGAGGGATCTACCTGC-1 2196 5291 12
Donor1-TGATTTCCAAGGACAC-1 1515 5059 7
Donor1-TGATTTCGTATCACCA-1 3378 6915 2
Donor1-TGCACCTGTATTACCG-1 2173 6880 10
Donor1-TGCACCTGTCTGGTCG-1 1708 4989 9
Donor1-TGCCAAACACGTCTCT-1 1319 3261 10
Donor1-TGCCAAAGTCAAAGCG-1 2146 7655 9
Donor1-TGCCAAAGTTCTGTTT-1 1816 6298 13
Donor1-TGCCAAAGTTTGACAC-1 1029 2981 8
Donor1-TGCCAAATCATCGCTC-1 5207 16167 1
Donor1-TGCCCATAGCGAAGGG-1 4625 16193 3

Donor1-TGCCCATCATCACGAT-1 3524 7595 2
Donor1-TGCCCTAAGTGAAGTT-1 1795 4802 13
Donor1-TGCCCTACACGCTTTC-1 5502 26177 6
Donor1-TGCGCAGAGATCACGG-1 2008 5750 10
Donor1-TGCGCAGAGCTTCGCG-1 1055 2906 8
Donor1-TGCGCAGCACAGGCCT-1 1498 5459 7
Donor1-TGCGCAGGTAGCTCCG-1 2654 8173 11
Donor1-TGCGTGGAGACAAAGG-1 3102 7805 5
Donor1-TGCTACCGTGGTGTAG-1 2369 7903 10
Donor1-TGCTACCTCGGTCCGA-1 1714 5618 9
Donor1-TGCTGCTAGGGTTCCC-1 2013 4964 6
Donor1-TGCTGCTTCGCGCCAA-1 1276 3335 13
Donor1-TGGACGCCAACGCACC-1 2123 5934 11
Donor1-TGGACGCGTCGCGGTT-1 1503 3744 13
Donor1-TGGACGCTCAGCTCTC-1 1900 3885 3
Donor1-TGGCCAGCACGGACAA-1 2621 5560 3
Donor1-TGGCGCACATCCGCGA-1 2680 7740 11
Donor1-TGGGAAGAGAAGGTGA-1 1227 3107 7
Donor1-TGGGAAGAGAGGTTGC-1 1831 5849 13
Donor1-TGGGAAGTCACAGTAC-1 1900 4633 11
Donor1-TGGTTAGGTTCGTTGA-1 2238 8735 10
Donor1-TGGTTCCAGATCGATA-1 1018 4088 8
Donor1-TGGTTCCAGCCCAATT-1 1739 6137 8
Donor1-TGGTTCCAGGGATCTG-1 1078 3623 7
Donor1-TGGTTCCAGTGAATTG-1 2819 5924 3
Donor1-TGGTTCCGTGGGTATG-1 1852 3332 4
Donor1-TGTATTCAGGAGCGAG-1 3024 13742 10
Donor1-TGTATTCGTACAGTTC-1 3080 10913 11
Donor1-TGTATTCGTCGAATCT-1 2834 5855 2
Donor1-TGTATTCTCGAATGGG-1 3430 8308 3
Donor1-TGTGGTACAGATTGCT-1 1508 3736 11
Donor1-TGTGGTACAGCCTTTC-1 1362 4855 7
Donor1-TGTGGTACATTCCTCG-1 3085 6921 4
Donor1-TGTGTTTAGTACGATA-1 1697 3995 11
Donor1-TGTTCCGTCACCCTCA-1 1561 3514 10
Donor1-TTAACTCGTTCATGGT-1 4012 10432 4
Donor1-TTAACTCTCAATCTCT-1 3386 15675 10
Donor1-TTAGGACAGCGCCTCA-1 6597 23427 1
Donor1-TTAGGCAGTCCTCCAT-1 1750 6035 10
Donor1-TTAGTTCCATCCCATC-1 5995 26691 4
Donor1-TTAGTTCGTATCTGCA-1 4792 14723 1
Donor1-TTAGTTCTCGCCAGCA-1 1640 4726 9
Donor1-TTATGCTGTGACTACT-1 1411 4281 13
Donor1-TTATGCTGTGCGAAAC-1 4622 15147 4
Donor1-TTATGCTGTTCCACGG-1 1610 5078 13
Donor1-TTCCCAGGTGAGGGTT-1 1931 9527 8
Donor1-TTCCCAGTCAGTTAGC-1 5235 18333 6
Donor1-TTCCCAGTCGAATCCA-1 3253 7321 2
Donor1-TTCGAAGAGTGGGCTA-1 2159 8234 7
Donor1-TTCGGTCAGTACGTTC-1 2033 6111 10
Donor1-TTCGGTCCAAGCGATG-1 2662 5517 3
Donor1-TTCGGTCCACAACTGT-1 1156 3417 9
Donor1-TTCGGTCTCAGGTTCA-1 3620 8075 2
Donor1-TTCTCAACAGCCTATA-1 1846 5370 13
Donor1-TTCTCAAGTCGGCATC-1 1591 4181 6
Donor1-TTCTCAAGTGCCTGTG-1 3275 11031 11

Donor1-TTCTCAATCACGCATA-1 3300 10802 11
Donor1-TTCTCCTAGGACTGGT-1 1378 5365 8
Donor1-TTCTCCTTCCGCATAA-1 3880 8759 2
Donor1-TTCTCCTTCTTCTGGC-1 1598 4935 9
Donor1-TTGAACGCAACGCACC-1 1542 4734 9
Donor1-TTGAACGCAGCGAACA-1 3470 11488 12
Donor1-TTGACTTCACACCGAC-1 1174 3482 8
Donor1-TTGACTTGTATAGGGC-1 2781 7061 11
Donor1-TTGACTTGTCGACTGC-1 3223 7177 3
Donor1-TTGACTTTCGCCATAA-1 1549 3456 11
Donor1-TTGCCGTCAATAAGCA-1 2046 4082 4
Donor1-TTGCGTCAGATGTGGC-1 2525 7585 11
Donor1-TTGCGTCGTGACTCAT-1 6834 31158 1
Donor1-TTGGAACAGCTCCTTC-1 1627 5786 13
Donor1-TTGGAACAGTCGATAA-1 2361 9335 7
Donor1-TTGGCAACACGAGAGT-1 2861 6464 4
Donor1-TTGGCAAGTGATAAGT-1 5523 17362 2
Donor1-TTGTAGGAGGCATTGG-1 1486 4473 13
Donor1-TTGTAGGTCACTCCTG-1 2508 6346 11
Donor1-TTGTAGGTCCAGATCA-1 906 2863 8
Donor1-TTTACTGAGAAACGCC-1 3227 8098 3
Donor1-TTTACTGGTGGCGAAT-1 4898 21436 6
Donor1-TTTATGCAGGGCACTA-1 3528 9114 3
Donor1-TTTATGCAGTATCGAA-1 2419 7824 7
Donor1-TTTATGCCATGACATC-1 1165 3794 8
Donor1-TTTATGCGTTAGTGGG-1 1732 5701 9
Donor1-TTTCCTCAGATATGGT-1 1256 5295 8
Donor1-TTTCCTCTCACATAGC-1 3907 10304 3
Donor1-TTTCCTCTCGCCAAAT-1 1482 5351 8
Donor1-TTTGCGCAGCGCCTCA-1 1984 6647 13
Donor1-TTTGGTTCAGTAAGCG-1 1685 4049 13
Donor1-TTTGGTTGTCATGCCG-1 1118 2878 9
Donor1-TTTGGTTGTGCAACGA-1 1469 3778 13
Donor1-TTTGTCAAGGACTGGT-1 5943 21537 1
Donor1-TTTGTCACATGGTAGG-1 4807 14378 1
Donor1-TTTGTCACATTGGGCC-1 2375 4587 2
Donor1-AAACCTGCAAAGTCAA-2 1721 4392 10
Donor1-AAACCTGTCATGCATG-2 3835 10035 3
Donor1-AAACGGGCAGCTTAAC-2 1250 4070 8
Donor1-AAAGATGAGACAGACC-2 4763 14004 1
Donor1-AAAGATGAGGGCATGT-2 2329 9269 9
Donor1-AAAGATGAGTGATCGG-2 2339 5078 6
Donor1-AAAGATGCATCCGTGG-2 2384 13424 8
Donor1-AAAGCAACATCCGTGG-2 1218 3719 9
Donor1-AAAGCAAGTTCATGGT-2 2151 7681 13
Donor1-AAAGTAGAGAGCAATT-2 2120 4655 5
Donor1-AAAGTAGGTCGCCATG-2 7440 41611 1
Donor1-AAATGCCAGCAGACTG-2 5393 17335 2
Donor1-AAATGCCTCTCGAGTA-2 1237 3592 13
Donor1-AAATGCCTCTTCGAGA-2 6019 23650 4
Donor1-AACACGTGTGTTGAGG-2 4091 11580 6
Donor1-AACACGTTCTACTCAT-2 4940 27152 6
Donor1-AACCATGCAATGTAAG-2 1268 3610 7
Donor1-AACCATGCAGCAGTTT-2 7188 42862 3
Donor1-AACCATGTCTCCCTGA-2 6092 22021 4
Donor1-AACCGCGAGGCGTACA-2 5428 20025 2

Donor1-AACGTTGCACTACAGT-2 3210 7212 3
Donor1-AACGTTGGTACTTAGC-2 2098 8072 9
Donor1-AACTCAGAGGGCTTCC-2 2767 5127 5
Donor1-AACTCCCAGAGTACAT-2 3463 7905 3
Donor1-AACTCCCAGGCAATTA-2 1624 5279 9
Donor1-AACTCCCTCATACGGT-2 1569 3853 7
Donor1-AACTCTTCACACCGAC-2 3568 7391 5
Donor1-AACTCTTTCGGCGGTT-2 1968 3794 6
Donor1-AACTGGTGTAGCTGCC-2 1459 6064 8
Donor1-AACTGGTGTGTGAAAT-2 3496 7977 4
Donor1-AACTTTCAGGTGCTTT-2 2704 5345 4
Donor1-AAGACCTAGTACGCCC-2 1586 3706 10
Donor1-AAGACCTAGTCAAGGC-2 2206 6396 10
Donor1-AAGACCTGTCAAACTC-2 1499 5292 8
Donor1-AAGCCGCAGCAAATCA-2 2554 4556 2
Donor1-AAGGAGCAGAGAGCTC-2 3024 6805 3
Donor1-AAGGCAGAGTCATGCT-2 4443 13078 1
Donor1-AAGGCAGGTGAGCGAT-2 1434 3986 13
Donor1-AAGGCAGTCGTCACGG-2 2345 6922 13
Donor1-AAGGTTCCATGAACCT-2 1887 5921 13
Donor1-AAGGTTCGTCAGAGGT-2 1925 5050 13
Donor1-AAGTCTGAGTTTCCTT-2 4000 10293 4
Donor1-AAGTCTGCACAACTGT-2 3103 7154 3
Donor1-AAGTCTGCAGGCAGTA-2 3627 8360 2
Donor1-AATCCAGCATCGGTTA-2 2381 8060 10
Donor1-AATCCAGGTGCTGTAT-2 1710 5852 9
Donor1-AATCCAGGTTCCTCCA-2 1345 3767 7
Donor1-ACACCAAGTAATCACC-2 2325 7058 7
Donor1-ACACCAAGTCTTGCGG-2 4630 12749 2
Donor1-ACACCCTCAGGTCCAC-2 2218 7370 13
Donor1-ACACCCTCATGACATC-2 1602 4512 13
Donor1-ACACCGGAGGTGCTTT-2 2897 10354 13
Donor1-ACACCGGCAATCTGCA-2 3155 16169 7
Donor1-ACACCGGCAGCTCGAC-2 1338 3922 7
Donor1-ACACCGGGTCGCGTGT-2 3712 9472 3
Donor1-ACACCGGTCGCCATAA-2 1218 3964 8
Donor1-ACAGCCGAGTGTTTGC-2 2486 4727 4
Donor1-ACAGCCGCAATAACGA-2 1307 4972 8
Donor1-ACAGCCGCACCGCTAG-2 1253 3485 8
Donor1-ACAGCCGTCAACGAAA-2 2125 6837 6
Donor1-ACAGCCGTCCTAAGTG-2 1230 4335 8
Donor1-ACAGCTAAGGATGTAT-2 3520 8170 2
Donor1-ACAGCTAGTGCAACGA-2 1952 3722 1
Donor1-ACATACGAGTAACCCT-2 3363 7668 1
Donor1-ACATACGTCTGCCCTA-2 1545 5005 7
Donor1-ACATCAGCAAGGTTCT-2 2492 5182 4
Donor1-ACATCAGGTACTTCTT-2 1502 4058 13
Donor1-ACATCAGGTTACGTCA-2 2296 12746 8
Donor1-ACATCAGGTTACTGAC-2 1939 6126 13
Donor1-ACATGGTGTCTCGTTC-2 2813 5621 5
Donor1-ACATGGTTCCCTTGCA-2 7528 65994 4
Donor1-ACCAGTACAACACCTA-2 1351 4009 13
Donor1-ACCAGTACAAGACGTG-2 1985 10916 8
Donor1-ACCAGTACAGATCCAT-2 1550 4567 13
Donor1-ACCAGTAGTATAGGGC-2 4648 14433 1
Donor1-ACCCACTAGACAAAGG-2 2167 5970 9

Donor1-ACCCACTAGTGGACGT-2 1362 4813 13
Donor1-ACCCACTGTACGACCC-2 2022 4723 4
Donor1-ACCGTAAAGTTCGCAT-2 4057 11340 1
Donor1-ACCGTAACATCACAAC-2 1377 4748 8
Donor1-ACCTTTACACCGAATT-2 1896 8448 7
Donor1-ACCTTTAGTACAGCAG-2 1089 3670 8
Donor1-ACGAGCCAGCAGGTCA-2 3750 10039 1
Donor1-ACGAGCCAGGGAACGG-2 1503 4347 13
Donor1-ACGAGCCAGTGCAAGC-2 4146 22626 7
Donor1-ACGAGCCGTTCTGAAC-2 4523 14640 3
Donor1-ACGAGGACAGGGATTG-2 1785 8154 8
Donor1-ACGAGGAGTCTCTCTG-2 3486 8582 3
Donor1-ACGAGGAGTCTGCAAT-2 4143 11074 3
Donor1-ACGAGGAGTTGTCGCG-2 1533 3718 13
Donor1-ACGAGGATCCCACTTG-2 2255 7203 10
Donor1-ACGAGGATCGGTCCGA-2 4827 14435 3
Donor1-ACGATACCAATCTGCA-2 2834 5798 3
Donor1-ACGATACTCATGTGGT-2 5390 18599 1
Donor1-ACGATGTAGTGTGGCA-2 1256 4524 8
Donor1-ACGATGTGTACTTAGC-2 3283 7888 3
Donor1-ACGCAGCAGTGGAGAA-2 8092 50385 6
Donor1-ACGCAGCCATTCTTAC-2 5706 18861 4
Donor1-ACGCAGCTCCCTCAGT-2 1781 6944 8
Donor1-ACGCAGCTCTGATTCT-2 4946 15683 1
Donor1-ACGCCAGGTCGCCATG-2 2096 7846 9
Donor1-ACGCCAGGTGCCTGGT-2 2233 6971 7
Donor1-ACGCCAGTCCCAAGAT-2 6547 34250 4
Donor1-ACGCCGAAGTGACTCT-2 1680 7734 8
Donor1-ACGCCGACAGATAATG-2 3419 7835 3
Donor1-ACGCCGAGTCGAATCT-2 1710 6577 9
Donor1-ACGCCGAGTTCCCGAG-2 3595 7979 3
Donor1-ACGGAGAGTCTCATCC-2 4167 9925 2
Donor1-ACGGCCAAGGCTCTTA-2 1347 3562 7
Donor1-ACGGCCAGTCAGAAGC-2 3169 7050 2
Donor1-ACGGGCTCAAGTTCTG-2 3582 9389 10
Donor1-ACGGGCTCACTTAAGC-2 1277 4119 8
Donor1-ACGGGCTGTCTAAAGA-2 1757 3863 6
Donor1-ACGGGCTTCAGTTAGC-2 1511 4478 7
Donor1-ACGGGCTTCCTAGAAC-2 3833 10615 2
Donor1-ACGGGCTTCGGTGTTA-2 3645 9212 3
Donor1-ACGGGTCCACGAAGCA-2 3923 10378 2
Donor1-ACGGGTCGTGCCTGCA-2 1420 5755 8
Donor1-ACGGGTCTCGTATCAG-2 1991 8466 8
Donor1-ACGTCAAAGAAGCCCA-2 3761 9057 5
Donor1-ACGTCAAAGTAGGTGC-2 1444 4295 13
Donor1-ACGTCAACATGGAATA-2 3901 9918 2
Donor1-ACGTCAAGTAAACCTC-2 3034 6861 3
Donor1-ACGTCAAGTAGCTAAA-2 3141 7162 3
Donor1-ACGTCAAGTCTAACGT-2 4329 11615 2
Donor1-ACTATCTGTAAATGTG-2 3063 6683 4
Donor1-ACTGAACAGAAGATTC-2 4390 12409 5
Donor1-ACTGAACGTCTGGAGA-2 3155 7522 6
Donor1-ACTGAACGTTGCTCCT-2 2512 11114 7
Donor1-ACTGAACTCACGGTTA-2 2678 6075 3
Donor1-ACTGAGTAGCACGCCT-2 1529 4690 13
Donor1-ACTGAGTAGGAATGGA-2 3638 7724 5

Donor1-ACTGAGTAGGATGCGT-2 2568 4547 2
Donor1-ACTGATGAGTGATCGG-2 3161 7503 2
Donor1-ACTGATGAGTGTCCCG-2 2575 5482 2
Donor1-ACTGATGCACTTACGA-2 2454 7349 10
Donor1-ACTGATGTCACTTACT-2 1870 6283 9
Donor1-ACTGATGTCCAAACAC-2 1383 4677 8
Donor1-ACTGCTCGTCCCTACT-2 6763 25848 1
Donor1-ACTGTCCGTACCGAGA-2 3246 8224 3
Donor1-ACTGTCCTCAGCAACT-2 1484 4912 8
Donor1-ACTGTCCTCATATCGG-2 4506 14079 1
Donor1-ACTGTCCTCTCTGTCG-2 2884 6163 6
Donor1-ACTTACTAGGGATGGG-2 2901 6399 2
Donor1-ACTTACTCACTTCGAA-2 2228 5717 11
Donor1-ACTTACTCAGTAAGCG-2 2030 7209 9
Donor1-ACTTACTGTCTCAACA-2 3088 6825 3
Donor1-ACTTACTTCAGCTCGG-2 2999 9397 10
Donor1-ACTTACTTCCCTCTTT-2 2317 6038 6
Donor1-ACTTACTTCCGAACGC-2 2612 9348 10
Donor1-ACTTGTTAGTCCGTAT-2 2355 12284 8
Donor1-ACTTGTTGTCCCGACA-2 6229 22499 3
Donor1-ACTTGTTTCCCTAATT-2 2008 6561 13
Donor1-ACTTGTTTCGAATGGG-2 1713 4991 13
Donor1-ACTTTCACAAGGTTTC-2 2675 5207 3
Donor1-ACTTTCAGTCTTCAAG-2 1839 7726 8
Donor1-ACTTTCATCTGCAAGT-2 5637 20269 2
Donor1-AGAATAGCAGTATAAG-2 3057 6786 2
Donor1-AGAATAGCAGTCTTCC-2 1762 5735 7
Donor1-AGAATAGCAGTTCATG-2 2705 13495 7
Donor1-AGAATAGGTCACCCAG-2 3463 14387 7
Donor1-AGAATAGTCACCTCGT-2 2432 9082 10
Donor1-AGAATAGTCCCAAGAT-2 4928 16940 2
Donor1-AGACGTTGTAAACACA-2 4427 20571 6
Donor1-AGAGCGAAGTACGTTC-2 5258 16629 3
Donor1-AGAGCGACAAAGTCAA-2 3076 8005 3
Donor1-AGAGCGACACAGCCCA-2 2481 13945 8
Donor1-AGAGCGACATCGATTG-2 2435 12327 8
Donor1-AGAGCTTCAATAGAGT-2 3459 12783 6
Donor1-AGAGCTTGTAGAGCTG-2 4065 10317 1
Donor1-AGAGCTTTCCACTGGG-2 2092 8287 9
Donor1-AGAGCTTTCCGTCATC-2 1419 5366 8
Donor1-AGAGTGGAGGGATCTG-2 1487 5361 8
Donor1-AGATCTGCATACGCTA-2 1632 3958 13
Donor1-AGATCTGCATGAACCT-2 2405 9117 7
Donor1-AGATCTGGTTCATGGT-2 1933 5677 13
Donor1-AGATCTGTCCTATGTT-2 4246 9781 5
Donor1-AGATTGCAGGATGCGT-2 1869 4062 11
Donor1-AGATTGCCACTGAAGG-2 3020 6423 6
Donor1-AGATTGCTCAAACAAG-2 2067 9038 8
Donor1-AGCAGCCAGTCCTCCT-2 1788 7425 8
Donor1-AGCAGCCCATGTTGAC-2 3743 10286 1
Donor1-AGCATACCAAGGACAC-2 5707 31374 5
Donor1-AGCATACTCCATGCTC-2 2341 7472 13
Donor1-AGCATACTCTCACATT-2 5692 22265 6
Donor1-AGCCTAACATGCTAGT-2 2828 5557 2
Donor1-AGCGGTCAGAGCTATA-2 1908 5621 7
Donor1-AGCGGTCTCTCGTATT-2 2410 13166 8

Donor1-AGCGTATAGAGTACAT-2 3101 7163 3
Donor1-AGCGTATAGCGGATCA-2 1208 4264 8
Donor1-AGCGTATTCGCATGAT-2 2149 10479 8
Donor1-AGCGTCGAGATGGGTC-2 5404 18529 1
Donor1-AGCGTCGGTTCCGTCT-2 2035 3740 3
Donor1-AGCGTCGTCAGAGCTT-2 2328 4095 6
Donor1-AGCGTCGTCTTGTATC-2 2048 4636 3
Donor1-AGCTCCTCAACACCCG-2 4799 32187 7
Donor1-AGCTCCTCACCAGGCT-2 1289 3654 8
Donor1-AGCTCCTCAGGCTCAC-2 3036 6643 3
Donor1-AGCTCCTGTGGGTATG-2 3545 7837 4
Donor1-AGCTCCTTCATATCGG-2 1539 4744 7
Donor1-AGCTCTCCATGAGCGA-2 4115 10751 6
Donor1-AGCTCTCCATGCAACT-2 1784 5859 7
Donor1-AGCTCTCGTGAACCTT-2 2938 8512 12
Donor1-AGGCCACTCGGTCCGA-2 1645 7210 8
Donor1-AGGCCGTCACTTAACG-2 1433 4055 7
Donor1-AGGCCGTGTATTCGTG-2 1076 3540 8
Donor1-AGGCCGTTCCTTTCGG-2 1359 3906 13
Donor1-AGGGAGTAGATAGGAG-2 1098 3465 7
Donor1-AGGGAGTCAATGGAAT-2 1303 5275 8
Donor1-AGGGAGTGTCACTTCC-2 2187 6364 10
Donor1-AGGGAGTGTTTAAGCC-2 3394 22909 7
Donor1-AGGGAGTTCTTGAGGT-2 2102 4532 6
Donor1-AGGGATGCACGTGAGA-2 2033 5505 10
Donor1-AGGGATGTCGAACTGT-2 3524 11760 9
Donor1-AGGGTGAAGGGAAACA-2 2669 6510 6
Donor1-AGGTCATGTGATGTGG-2 3875 10238 3
Donor1-AGGTCATGTTACGCGC-2 2793 8013 5
Donor1-AGGTCCGCAAAGCGGT-2 3308 8026 1
Donor1-AGGTCCGGTCTAAACC-2 2151 5768 13
Donor1-AGGTCCGGTGCCTGCA-2 1874 8839 8
Donor1-AGTAGTCCAGACAAGC-2 1756 6311 13
Donor1-AGTAGTCCATAGAAAC-2 1554 4618 8
Donor1-AGTCTTTAGGAGTACC-2 2060 6772 7
Donor1-AGTCTTTCATGCATGT-2 3590 10695 6
Donor1-AGTCTTTTCTATCGCC-2 3052 6669 3
Donor1-AGTCTTTTCTGGTATG-2 1439 3711 13
Donor1-AGTCTTTTCTTCGGTC-2 2624 5559 1
Donor1-AGTGAGGCACTGCCAG-2 3354 7397 3
Donor1-AGTGAGGTCTGATTCT-2 2468 7482 10
Donor1-AGTGGGATCGGCTTGG-2 1859 4976 13
Donor1-AGTGGGATCGGTCTAA-2 2914 6139 2
Donor1-AGTGTCAGTCTAGCCG-2 2947 6476 3
Donor1-AGTGTCATCCCTAACC-2 2799 9202 10
Donor1-AGTTGGTAGACAATAC-2 2333 4672 5
Donor1-AGTTGGTCACGAGAGT-2 1436 4184 13
Donor1-ATAAGAGCATGCGCAC-2 4153 12780 6
Donor1-ATAGACCAGGTAGCTG-2 4646 13764 5
Donor1-ATAGACCCATGGTCAT-2 4435 15373 6
Donor1-ATAGACCTCGCTTAGA-2 3479 12298 4
Donor1-ATCACGAAGAAGGCCT-2 6058 27752 4
Donor1-ATCATCTAGAACAATC-2 1596 5709 13
Donor1-ATCATCTAGGGATACC-2 2266 4318 5
Donor1-ATCATCTCATGAGCGA-2 4914 45026 7
Donor1-ATCATCTGTTATCACG-2 3548 8755 3

Donor1-ATCATGGCACCACGTG-2 5299 19495 1
Donor1-ATCATGGCACCTATCC-2 3529 8364 3
Donor1-ATCATGGCATAAGACA-2 6608 25303 1
Donor1-ATCCACCGTAAGGATT-2 2404 6105 6
Donor1-ATCCACCGTAGCTCCG-2 1648 4110 7
Donor1-ATCCGAACAGACGCAA-2 1270 4564 8
Donor1-ATCCGAAGTTAAGATG-2 1473 3902 13
Donor1-ATCCGAATCATTCACT-2 2447 5392 12
Donor1-ATCCGAATCCCACTTG-2 3733 10001 2
Donor1-ATCGAGTAGCGATCCC-2 5320 22724 4
Donor1-ATCGAGTAGGCGTACA-2 1900 5720 13
Donor1-ATCGAGTCACTTAACG-2 4409 12661 3
Donor1-ATCTACTAGACGCTTT-2 1481 4342 13
Donor1-ATCTACTGTCCGAGTC-2 1486 4375 8
Donor1-ATCTACTTCCTTGCCA-2 3724 8789 1
Donor1-ATCTGCCAGACAAGCC-2 1666 5432 13
Donor1-ATCTGCCCAAGAGGCT-2 2256 3758 6
Donor1-ATCTGCCCACATTAGC-2 2730 5577 1
Donor1-ATGAGGGCATAGGATA-2 1732 7669 8
Donor1-ATGAGGGGTTAGATGA-2 2041 4077 6
Donor1-ATGCGATCACACATGT-2 1884 5525 6
Donor1-ATGGGAGAGACATAAC-2 3627 13438 6
Donor1-ATGTGTGAGGATGTAT-2 1909 6959 9
Donor1-ATGTGTGAGTACCGGA-2 4913 16279 1
Donor1-ATTACTCCAAGGACAC-2 3059 7141 3
Donor1-ATTACTCGTAAATGAC-2 2704 5297 2
Donor1-ATTACTCGTGATGTCT-2 3123 7054 3
Donor1-ATTATCCCATCATCCC-2 5184 16051 3
Donor1-ATTATCCGTCTCGTTC-2 6328 33794 6
Donor1-ATTATCCGTGCGAAAC-2 2423 5367 3
Donor1-ATTATCCTCCTTTACA-2 2012 9843 8
Donor1-ATTGGACAGGTAAACT-2 1306 4168 8
Donor1-ATTGGACGTCGAGATG-2 2139 4193 3
Donor1-ATTGGTGTCATGCAAC-2 2865 9204 11
Donor1-ATTGGTGTCTTGTTTG-2 1535 5614 8
Donor1-ATTTCTGAGGCGACAT-2 6479 28345 3
Donor1-CAACCAAAGCGTCAAG-2 1721 7027 8
Donor1-CAACCAAGTTGAGTTC-2 1124 3649 8
Donor1-CAACCTCAGATGTCGG-2 1986 7258 9
Donor1-CAACCTCCAGGACCCT-2 4101 9229 1
Donor1-CAACCTCCATACGCTA-2 3440 10611 6
Donor1-CAACCTCGTCGACTGC-2 2058 4265 10
Donor1-CAACCTCTCCTCAACC-2 2039 5427 10
Donor1-CAACTAGCAATGAATG-2 4412 13298 4
Donor1-CAACTAGGTCTGGTCG-2 9312 79594 6
Donor1-CAAGAAACACGCTTTC-2 2325 4038 6
Donor1-CAAGAAACAGCTATTG-2 2890 12027 6
Donor1-CAAGAAACATCCCATC-2 2687 9528 10
Donor1-CAAGAAATCACTCCTG-2 2545 5511 3
Donor1-CAAGAAATCAGTCCCT-2 4376 12450 3
Donor1-CAAGATCCAAGTACCT-2 5541 18008 1
Donor1-CAAGATCCACCACCAG-2 1568 4921 7
Donor1-CAAGATCGTCTTCTCG-2 2137 5801 6
Donor1-CAAGATCGTTTACTCT-2 3792 8341 3
Donor1-CAAGATCTCGGATGTT-2 2452 7114 13
Donor1-CAAGATCTCTCGGACG-2 4122 11439 3

Donor1-CAAGGCCAGCCCAATT-2 6996 33161 1
Donor1-CAAGGCCGTACCAGTT-2 2464 9849 7
Donor1-CAAGGCCGTTCCGGCA-2 1495 4714 8
Donor1-CAAGTTGAGTAAGTAC-2 3457 11616 11
Donor1-CAAGTTGCATAAAGGT-2 5690 19831 3
Donor1-CAAGTTGTCGCTTAGA-2 4968 16999 6
Donor1-CACACAACAAGTAATG-2 4870 12347 2
Donor1-CACACAACACATCCAA-2 2395 6334 10
Donor1-CACACCTCAAACAACA-2 1378 3783 13
Donor1-CACACCTGTCCGAACC-2 2243 7203 7
Donor1-CACACCTTCCACGACG-2 2529 4049 5
Donor1-CACACTCCATCGGACC-2 2504 4963 1
Donor1-CACACTCGTCATGCCG-2 5578 18052 5
Donor1-CACACTCGTCTCTTTA-2 6211 22673 1
Donor1-CACACTCGTCTTCAAG-2 2021 4059 4
Donor1-CACAGGCTCCGCGCAA-2 5124 14398 3
Donor1-CACAGTAAGCTCAACT-2 2398 10465 9
Donor1-CACAGTAGTTAAGACA-2 1571 4733 7
Donor1-CACAGTATCAGAGACG-2 1179 3657 8
Donor1-CACAGTATCATTGCGA-2 4129 10592 4
Donor1-CACATAGAGTGGGATC-2 5827 23967 4
Donor1-CACATTTAGGAATCGC-2 3547 9070 6
Donor1-CACATTTGTCTCCACT-2 1607 4409 9
Donor1-CACCACTAGACTAGAT-2 3334 8038 3
Donor1-CACCACTAGTCTCCTC-2 8960 64516 6
Donor1-CACCACTCAGGTCGTC-2 3621 8907 3
Donor1-CACCACTGTAGCGCAA-2 4459 13682 4
Donor1-CACCACTGTATGAATG-2 1390 4557 8
Donor1-CACCACTTCAGTTTGG-2 3042 6870 2
Donor1-CACCAGGAGGTCGGAT-2 4732 15713 2
Donor1-CACCAGGCAAGCGATG-2 1427 4355 13
Donor1-CACCAGGCAATGGAAT-2 7091 47663 4
Donor1-CACCAGGCAGCTTCGG-2 3597 9364 1
Donor1-CACCAGGGTATTAGCC-2 1879 7022 7
Donor1-CACCTTGAGCGTTGCC-2 4400 13394 2
Donor1-CACCTTGTCGGTCCGA-2 1402 4950 7
Donor1-CACCTTGTCTAACTGG-2 2566 14007 7
Donor1-CACTCCAAGCCGTCGT-2 3098 6591 2
Donor1-CACTCCAGTCGCCATG-2 3284 7382 2
Donor1-CACTCCAGTCTCACCT-2 2972 5531 5
Donor1-CACTCCAGTGGTAACG-2 3347 8845 3
Donor1-CACTCCAGTGTAATGA-2 3057 18241 7
Donor1-CACTCCATCATAGCAC-2 3046 6524 2
Donor1-CACTCCATCATGTCTT-2 4743 17446 2
Donor1-CAGAATCCAATGTAAG-2 2827 10646 8
Donor1-CAGAATCCATTAGCCA-2 2413 13689 8
Donor1-CAGAATCGTGTTTGTG-2 3698 9208 4
Donor1-CAGAGAGAGTTTCCTT-2 5017 15466 2
Donor1-CAGAGAGCAAGACGTG-2 4591 13168 6
Donor1-CAGAGAGTCATCGCTC-2 3549 9379 3
Donor1-CAGATCAAGACGCAAC-2 4914 14361 4
Donor1-CAGATCATCTCATTCA-2 2474 7416 13
Donor1-CAGCAGCCAATGGACG-2 993 3813 8
Donor1-CAGCAGCTCACTTATC-2 1212 4049 8
Donor1-CAGCATACACGAGGTA-2 1250 3869 8
Donor1-CAGCCGAAGGGATACC-2 1507 4932 7

Donor1-CAGCCGACAATTCCTT-2 1589 5758 8
Donor1-CAGCCGAGTCACCCAG-2 3375 8337 3
Donor1-CAGCCGATCCTTGACC-2 1962 6614 7
Donor1-CAGCCGATCTTGCAAG-2 3137 5954 5
Donor1-CAGCGACGTAGGCTGA-2 1670 4830 6
Donor1-CAGCTAACAGACAGGT-2 6313 27780 1
Donor1-CAGCTAAGTCCCTACT-2 2926 6507 3
Donor1-CAGCTGGGTTTGTTGG-2 7796 39180 5
Donor1-CAGCTGGTCACAGGCC-2 2331 4665 3
Donor1-CAGCTGGTCGTCACGG-2 2815 5532 2
Donor1-CAGGTGCAGACCGGAT-2 3389 7978 1
Donor1-CAGTAACCAACAACCT-2 2469 4692 3
Donor1-CAGTAACCAGACGCCT-2 6845 38306 4
Donor1-CATATGGCAGCGAACA-2 3433 8761 1
Donor1-CATATTCAGTTTCCTT-2 2120 4048 3
Donor1-CATATTCCACCCATGG-2 2275 8995 7
Donor1-CATATTCGTCTTCAAG-2 5984 22772 1
Donor1-CATATTCGTGCACGAA-2 5698 17908 2
Donor1-CATCAAGAGCCGGTAA-2 2775 5838 4
Donor1-CATCAAGCAATGGTCT-2 1677 6144 8
Donor1-CATCAAGTCATCTGCC-2 1480 4799 8
Donor1-CATCAGAGTCTCGTTC-2 2465 5279 1
Donor1-CATCAGAGTTGGAGGT-2 1402 4364 13
Donor1-CATCCACCATGGGAAC-2 3408 8244 3
Donor1-CATCCACTCACCGTAA-2 2466 10698 9
Donor1-CATCCACTCGCCGTGA-2 2361 6507 10
Donor1-CATCGAAAGCTTATCG-2 1185 4192 8
Donor1-CATCGAATCCCAAGAT-2 3766 9807 3
Donor1-CATCGGGAGCAGACTG-2 2279 10559 8
Donor1-CATCGGGTCGTTTATC-2 5425 18726 2
Donor1-CATCGGGTCTTGAGAC-2 3289 7320 1
Donor1-CATGACAAGAATTGTG-2 3121 6732 4
Donor1-CATGACATCAGAGGTG-2 3776 14071 12
Donor1-CATGCCTAGATCTGCT-2 2085 7601 9
Donor1-CATGCCTAGGTGACCA-2 3931 10842 3
Donor1-CATGCCTCAATAGCAA-2 1650 4764 7
Donor1-CATGCCTGTGGCGAAT-2 4390 12870 12
Donor1-CATGGCGCAGACAAAT-2 6618 36433 4
Donor1-CATGGCGGTACAGCAG-2 4489 12941 1
Donor1-CATGGCGTCCACTGGG-2 1624 6488 8
Donor1-CATTATCAGCGGCTTC-2 3001 7252 4
Donor1-CATTATCAGTGTTAGA-2 5422 19981 2
Donor1-CATTATCCAGGAACGT-2 3541 6675 5
Donor1-CATTATCCAGTCTTCC-2 6667 37501 4
Donor1-CATTCGCAGCTCTCGG-2 6261 24191 3
Donor1-CATTCGCAGTACGCCC-2 1707 4871 13
Donor1-CATTCGCCAGTCACTA-2 3606 8187 2
Donor1-CATTCGCGTCTGATCA-2 4187 11881 4
Donor1-CATTCGCGTTAGTGGG-2 1598 4461 13
Donor1-CATTCGCGTTGCGCAC-2 2468 4832 2
Donor1-CCAATCCAGCTGAACG-2 2870 5780 6
Donor1-CCAATCCAGTAATCCC-2 3828 8772 3
Donor1-CCAATCCGTATGAAAC-2 2664 4495 6
Donor1-CCAATCCTCTGGTTCC-2 1159 3511 8
Donor1-CCACCTAAGATCACGG-2 1866 7658 7
Donor1-CCACCTAGTTCCAACA-2 3078 6166 2

Donor1-CCACCTATCGGAAACG-2 2801 9062 11
Donor1-CCACGGACACTCGACG-2 2242 3727 5
Donor1-CCACTACAGTACGCCC-2 3538 8888 3
Donor1-CCACTACGTAGAGCTG-2 1510 4081 13
Donor1-CCACTACGTCCTCTTG-2 3767 8650 3
Donor1-CCACTACTCATCGATG-2 2205 4328 1
Donor1-CCACTACTCCCTCAGT-2 1246 3916 8
Donor1-CCAGCGAAGGGTTCCC-2 3027 5870 5
Donor1-CCAGCGAGTCGATTGT-2 1531 4654 7
Donor1-CCAGCGATCCAAATGC-2 7006 34181 4
Donor1-CCAGCGATCTGTCAAG-2 4794 13081 3
Donor1-CCATGTCAGAAAGTGG-2 4438 11580 2
Donor1-CCATGTCCAAGCGCTC-2 2016 3783 2
Donor1-CCATGTCGTATCAGTC-2 3001 6618 4
Donor1-CCATGTCGTTGCGCAC-2 7452 49921 2
Donor1-CCATTCGCACATGACT-2 2322 10768 8
Donor1-CCATTCGCACGTCTCT-2 2565 10249 9
Donor1-CCATTCGTCATTATCC-2 1944 5764 6
Donor1-CCATTCGTCCATTCTA-2 5349 19567 5
Donor1-CCCAATCGTCTAAACC-2 1403 3479 7
Donor1-CCCAATCTCGAGCCCA-2 1172 3915 8
Donor1-CCCAGTTAGGTGATAT-2 3089 7066 3
Donor1-CCCAGTTAGTTAAGTG-2 3265 8338 1
Donor1-CCCAGTTGTCTCCACT-2 1221 3861 8
Donor1-CCCATACAGTCAAGCG-2 4257 13906 6
Donor1-CCCATACCACATTTCT-2 4268 11007 6
Donor1-CCCATACTCCACTCCA-2 3795 9286 3
Donor1-CCCTCCTAGGAATGGA-2 2162 7911 7
Donor1-CCGGGATCATGCCACG-2 2127 8111 7
Donor1-CCGGGATGTGTTTGTG-2 1559 6946 8
Donor1-CCGGTAGAGGCCATAG-2 3424 9676 1
Donor1-CCGGTAGCATGCCACG-2 3026 13299 7
Donor1-CCGTACTGTACGCTGC-2 2592 4519 6
Donor1-CCGTGGACAAGTCATC-2 3546 15278 7
Donor1-CCGTGGAGTAAGTTCC-2 4304 10612 6
Donor1-CCGTGGAGTATTAGCC-2 3356 8233 4
Donor1-CCGTTCAAGATGAGAG-2 1193 4423 8
Donor1-CCGTTCAAGCTGTTCA-2 1045 3487 8
Donor1-CCGTTCAAGGAGTTTA-2 3818 8268 1
Donor1-CCGTTCAAGTGCTGCC-2 5733 19046 1
Donor1-CCGTTCACAATTGCTG-2 2906 14909 7
Donor1-CCGTTCACATCGACGC-2 2602 9291 13
Donor1-CCGTTCAGTATAGGTA-2 2129 3874 5
Donor1-CCTAAAGTCAAACCGT-2 4605 11798 5
Donor1-CCTAAAGTCAGGTTCA-2 1215 4071 8
Donor1-CCTAAAGTCGTGGTCG-2 3752 8487 2
Donor1-CCTACACCAGTACACT-2 1871 5305 7
Donor1-CCTAGCTAGAGTCGGT-2 1560 4835 13
Donor1-CCTAGCTAGTGGAGTC-2 4246 12022 2
Donor1-CCTAGCTCACATCCGG-2 1986 4688 10
Donor1-CCTAGCTCATTACCTT-2 2088 9913 8
Donor1-CCTAGCTGTAGAAAGG-2 3046 6654 3
Donor1-CCTAGCTGTATTACCG-2 2035 3506 6
Donor1-CCTAGCTGTTGCGCAC-2 4579 14350 3
Donor1-CCTATTAAGAAAGTGG-2 4895 16017 2
Donor1-CCTATTAAGTACACCT-2 1390 5636 8

Donor1-CCTATTAGTAAATACG-2 1669 4865 13
Donor1-CCTATTAGTCCAGTAT-2 3322 7587 4
Donor1-CCTCAGTTCCTTTCGG-2 4228 10990 4
Donor1-CCTCAGTTCGGCGGTT-2 5473 20826 1
Donor1-CCTCTGAGTCCGCTGA-2 2280 4605 2
Donor1-CCTCTGATCCAAACAC-2 6355 29594 6
Donor1-CCTCTGATCCTTTCTC-2 3858 10051 3
Donor1-CCTTACGAGCGTGTCC-2 1321 3889 7
Donor1-CCTTACGCATCCAACA-2 2088 7352 9
Donor1-CCTTACGGTAGGGACT-2 1543 6136 8
Donor1-CCTTACGTCTGGTATG-2 1467 4037 13
Donor1-CCTTCCCAGGTGCTTT-2 2261 6435 4
Donor1-CCTTCCCCAAGACGTG-2 1624 3754 10
Donor1-CCTTCCCCAGCCAATT-2 1940 11155 8
Donor1-CCTTCGAGTACCTACA-2 1977 7749 9
Donor1-CCTTTCTTCAGTTGAC-2 2200 4055 4
Donor1-CGAACATTCTGATTCT-2 6774 25458 1
Donor1-CGACCTTAGAGACGAA-2 1761 8133 8
Donor1-CGACCTTCAAGTCTAC-2 2888 8333 11
Donor1-CGACCTTGTAGCGTCC-2 1520 6231 8
Donor1-CGACCTTGTTCATGGT-2 2139 10679 8
Donor1-CGACCTTGTTCTCATT-2 6874 29806 5
Donor1-CGACCTTTCCAGATCA-2 1873 3894 1
Donor1-CGACTTCCACTGTCGG-2 6722 33950 4
Donor1-CGACTTCGTGGCTCCA-2 1366 3570 7
Donor1-CGACTTCGTGGTCTCG-2 4582 18166 6
Donor1-CGAGAAGAGAAGATTC-2 5848 25678 4
Donor1-CGAGAAGAGCGCTTAT-2 1520 4905 13
Donor1-CGAGAAGCAACTGGCC-2 3411 7229 5
Donor1-CGAGAAGCAATGAAAC-2 4655 15244 5
Donor1-CGAGAAGCACCTCGTT-2 1293 4043 13
Donor1-CGAGAAGGTGAAAGAG-2 1139 3601 8
Donor1-CGAGAAGGTTCCCGAG-2 1333 5244 8
Donor1-CGAGAAGTCACCTCGT-2 1734 8811 8
Donor1-CGAGCACAGATGGGTC-2 1545 5438 13
Donor1-CGAGCACCAATCCAAC-2 4746 14187 1
Donor1-CGAGCCAAGATGCGAC-2 3059 6614 3
Donor1-CGAGCCAGTCGCGAAA-2 4169 12588 1
Donor1-CGAGCCAGTGGTCCGT-2 1932 8848 8
Donor1-CGAGCCATCCTGCCAT-2 2362 10068 9
Donor1-CGATCGGCACGGCCAT-2 2769 6518 3
Donor1-CGATCGGGTAAATGAC-2 1587 6397 8
Donor1-CGATGGCAGGGATCTG-2 6536 29864 5
Donor1-CGATGGCCAGACAAAT-2 2018 7455 9
Donor1-CGATGTAAGGACACCA-2 2301 7195 10
Donor1-CGATGTACAAGCTGAG-2 4636 13909 2
Donor1-CGATTGAGTTCAACCA-2 4422 10395 3
Donor1-CGATTGATCCACTCCA-2 5397 17095 3
Donor1-CGCCAAGCAAACGCGA-2 1254 4084 8
Donor1-CGCCAAGCAATAAGCA-2 7104 41095 4
Donor1-CGCGGTACACTGCCAG-2 3198 7878 2
Donor1-CGCGGTAGTCTGGAGA-2 1318 4510 8
Donor1-CGCGGTAGTTGCTCCT-2 3401 7338 3
Donor1-CGCGTTTAGATATGCA-2 4392 14046 2
Donor1-CGCGTTTCACATGGGA-2 5237 16670 2
Donor1-CGCGTTTTCACCACCT-2 5028 16354 3

Donor1-CGCTATCAGGGCTCTC-2 4871 15074 1
Donor1-CGCTATCCATCTCGCT-2 1692 4502 10
Donor1-CGCTATCTCTGCCCTA-2 2409 4707 2
Donor1-CGCTGGAAGTACGTAA-2 1508 5365 13
Donor1-CGCTGGAGTCGCATCG-2 3973 10996 2
Donor1-CGCTTCAAGCAGATCG-2 4355 20026 6
Donor1-CGCTTCAAGCTGCCCA-2 2233 4307 3
Donor1-CGGACACAGATCCCGC-2 2754 7330 6
Donor1-CGGACACAGCAGGTCA-2 1200 3700 8
Donor1-CGGACACTCTTGAGGT-2 1289 3697 9
Donor1-CGGACGTCAAGCCCAC-2 2314 3731 5
Donor1-CGGACGTGTGTATGGG-2 2433 4995 3
Donor1-CGGACGTTCTATCCCG-2 3279 7470 4
Donor1-CGGACTGAGTGGGTTG-2 5160 18891 6
Donor1-CGGAGCTCAATGGTCT-2 4444 13119 2
Donor1-CGGAGTCAGAAGAAGC-2 1322 3763 7
Donor1-CGGAGTCAGTATGACA-2 1442 5148 8
Donor1-CGGAGTCGTAGGCATG-2 1606 5904 13
Donor1-CGGAGTCTCGTCCGTT-2 2194 7096 10
Donor1-CGGCTAGAGAGCTTCT-2 5360 21037 2
Donor1-CGGCTAGAGTCGCCGT-2 3669 9201 6
Donor1-CGGCTAGGTATAGGGC-2 2638 5246 2
Donor1-CGGCTAGGTCGGCACT-2 3343 8582 3
Donor1-CGGGTCACACGCGAAA-2 3486 8579 3
Donor1-CGGTTAAAGGTGATTA-2 2337 4493 3
Donor1-CGGTTAACAAACCTAC-2 1279 3585 13
Donor1-CGGTTAAGTGAGGGTT-2 5054 17297 5
Donor1-CGGTTAATCACCTCGT-2 2545 10713 7
Donor1-CGTAGCGGTCCTAGCG-2 2094 5735 10
Donor1-CGTAGCGTCACGGTTA-2 1246 4050 8
Donor1-CGTAGCGTCCCTTGTG-2 4893 13318 2
Donor1-CGTAGGCGTAATCACC-2 1826 5852 7
Donor1-CGTAGGCGTCGATTGT-2 1466 5631 8
Donor1-CGTAGGCTCTGCAGTA-2 1462 4453 13
Donor1-CGTCACTAGAGACTTA-2 5260 16814 4
Donor1-CGTCACTGTAGCTCCG-2 3971 10688 1
Donor1-CGTCAGGAGATGGCGT-2 1312 3792 8
Donor1-CGTCAGGAGTAGCGGT-2 3443 8737 1
Donor1-CGTCAGGAGTCTCCTC-2 1386 3516 10
Donor1-CGTCAGGGTAAACACA-2 1258 4995 8
Donor1-CGTCAGGGTCCGTGAC-2 2571 9580 7
Donor1-CGTCCATCATTCTCAT-2 4103 10353 3
Donor1-CGTCCATTCGGAGCAA-2 2900 5942 4
Donor1-CGTCCATTCTTGCAAG-2 1393 4954 8
Donor1-CGTCTACCACAACGTT-2 3605 8849 1
Donor1-CGTCTACTCCTTTCGG-2 2257 4777 6
Donor1-CGTGAGCGTCCGTGAC-2 6003 21351 2
Donor1-CGTGAGCTCATGGTCA-2 3156 9056 6
Donor1-CGTGTAAAGCCTATGT-2 3329 7968 2
Donor1-CGTGTAACATACTCTT-2 2915 10966 10
Donor1-CGTGTCTCAATGACCT-2 2423 7145 6
Donor1-CGTGTCTCATTTCAGG-2 6749 30722 6
Donor1-CGTGTCTGTAGAAAGG-2 4302 20922 7
Donor1-CGTGTCTGTGTGAAAT-2 6778 42389 4
Donor1-CGTTAGAAGTTGAGTA-2 2611 5404 3
Donor1-CGTTCTGCATCAGTAC-2 1825 5730 7

Donor1-CGTTCTGGTGATAAAC-2 7194 29606 1
Donor1-CGTTCTGTCGCGTTTC-2 1412 4553 7
Donor1-CGTTGGGAGCCAGAAC-2 1872 6310 13
Donor1-CGTTGGGAGCTAGCCC-2 2962 6922 3
Donor1-CGTTGGGAGTGTTGAA-2 3265 8389 1
Donor1-CGTTGGGGTCTCTCTG-2 1454 5484 8
Donor1-CGTTGGGTCCCAGGTG-2 1219 3738 13
Donor1-CTAACTTAGCACAGGT-2 1321 4270 8
Donor1-CTAACTTAGTGAATTG-2 2108 4417 11
Donor1-CTAACTTCAGGGTATG-2 1531 3457 10
Donor1-CTAACTTCATGGATGG-2 1790 4905 13
Donor1-CTAAGACAGGAGTCTG-2 2352 10496 8
Donor1-CTAAGACAGTATCTCG-2 1623 5547 7
Donor1-CTAAGACGTATCAGTC-2 6373 22940 2
Donor1-CTAAGACGTCTACCTC-2 3757 8418 5
Donor1-CTAATGGAGCCTTGAT-2 1942 5067 8
Donor1-CTAATGGCACTGTGTA-2 1741 4649 13
Donor1-CTAATGGCAGTCACTA-2 3867 9891 1
Donor1-CTACACCCAATGGTCT-2 3220 8469 6
Donor1-CTACACCCAGGTCCAC-2 1652 7032 8
Donor1-CTACACCGTCAGATAA-2 1647 4527 13
Donor1-CTACACCTCATCATTC-2 2169 7467 9
Donor1-CTACACCTCTAACGGT-2 5427 17494 1
Donor1-CTACACCTCTGCGGCA-2 2456 8987 10
Donor1-CTACATTAGTCAAGGC-2 1302 3690 7
Donor1-CTACATTCATTTCAGG-2 2194 7031 10
Donor1-CTACATTGTGCCTGCA-2 1496 3968 13
Donor1-CTACCCAAGTACGACG-2 3178 7796 3
Donor1-CTACCCAGTATTAGCC-2 1545 3900 13
Donor1-CTACCCATCAGTTCGA-2 3062 6910 3
Donor1-CTACGTCAGCTGGAAC-2 2933 6665 3
Donor1-CTACGTCAGGGTCTCC-2 2093 6669 13
Donor1-CTACGTCCACAGATTC-2 2438 4794 2
Donor1-CTACGTCCACTGTCGG-2 1650 5538 13
Donor1-CTACGTCGTAGCTCCG-2 3419 7220 5
Donor1-CTACGTCTCCGCATCT-2 1910 6069 13
Donor1-CTAGAGTCAAGTACCT-2 1671 5295 7
Donor1-CTAGAGTCATACAGCT-2 1697 5504 9
Donor1-CTAGCCTCAACGATGG-2 3163 7136 3
Donor1-CTAGCCTTCCTTGGTC-2 3946 9882 3
Donor1-CTAGTGAAGCTAGGCA-2 5257 15668 2
Donor1-CTAGTGACACAGCCCA-2 3938 14188 6
Donor1-CTAGTGATCCTGTACC-2 1992 9073 8
Donor1-CTCACACAGCGATCCC-2 5978 21055 4
Donor1-CTCACACAGTGAATTG-2 1521 6251 8
Donor1-CTCACACCACTGCCAG-2 4174 11142 1
Donor1-CTCACACGTTCCGTCT-2 1761 4500 6
Donor1-CTCACACTCGTTACGA-2 1854 9895 8
Donor1-CTCAGAAAGCTGATAA-2 1330 5089 8
Donor1-CTCATTACACTACAGT-2 2406 4643 2
Donor1-CTCCTAGAGGCTCAGA-2 3410 7931 3
Donor1-CTCCTAGAGTCCTCCT-2 1207 3778 8
Donor1-CTCGAAACAACTTGAC-2 1383 4282 8
Donor1-CTCGAAAGTTTGTTGG-2 1647 4392 7
Donor1-CTCGAGGCACTAAGTC-2 3498 8692 2
Donor1-CTCGAGGGTACAGTGG-2 3776 8720 3

Donor1-CTCGAGGGTACCGTAT-2 1631 5677 13
Donor1-CTCGGAGCATACGCCG-2 1384 5032 8
Donor1-CTCGGAGGTCCCTACT-2 4988 13821 2
Donor1-CTCGGGACACATTAGC-2 1389 4010 9
Donor1-CTCGGGATCCTAGAAC-2 1189 3541 7
Donor1-CTCGTACAGTATGACA-2 6341 22143 1
Donor1-CTCGTACTCAGTCCCT-2 3271 7431 3
Donor1-CTCGTACTCCGCATAA-2 2815 6093 3
Donor1-CTCGTACTCTAACTCT-2 2174 7998 9
Donor1-CTCGTACTCTGTCAAG-2 2221 4237 3
Donor1-CTCGTCAAGCTAAGAT-2 4697 15368 2
Donor1-CTCGTCATCCAGATCA-2 5916 21047 1
Donor1-CTCGTCATCCGAAGAG-2 3179 7662 2
Donor1-CTCTAATCACCAGATT-2 3115 5611 5
Donor1-CTCTAATGTCAAGCGA-2 1653 4252 7
Donor1-CTCTAATGTTTCGCTC-2 2027 4267 10
Donor1-CTCTACGGTTAAAGAC-2 1670 3567 11
Donor1-CTCTGGTCATTTCACT-2 3292 6607 4
Donor1-CTCTGGTGTCTGCAAT-2 1102 3487 8
Donor1-CTGAAACAGATGTGTA-2 2732 5924 3
Donor1-CTGAAACGTCTCGTTC-2 5977 22361 1
Donor1-CTGAAGTAGACAGGCT-2 1479 3722 13
Donor1-CTGAAGTAGACGCTTT-2 5395 18847 3
Donor1-CTGAAGTAGCCAGTTT-2 1885 6486 13
Donor1-CTGAAGTCACGTCTCT-2 2422 9466 9
Donor1-CTGAAGTGTCTTTCAT-2 3021 9357 11
Donor1-CTGAAGTTCAATACCG-2 4377 12429 2
Donor1-CTGAAGTTCAATCACG-2 1900 3469 6
Donor1-CTGATAGCAATCGAAA-2 2226 5414 13
Donor1-CTGATAGGTATAAACG-2 2109 3944 2
Donor1-CTGATAGTCTTGAGGT-2 2160 9586 7
Donor1-CTGATCCAGAGACTTA-2 1584 5067 7
Donor1-CTGATCCAGTGGAGAA-2 1977 5051 13
Donor1-CTGCCTAAGGAGTTGC-2 2996 6164 2
Donor1-CTGCCTACATACTACG-2 2511 4738 2
Donor1-CTGCCTAGTAAGGATT-2 5572 19836 1
Donor1-CTGCCTATCTCTGAGA-2 2221 8365 7
Donor1-CTGCGGACACTGCCAG-2 4390 14987 2
Donor1-CTGCGGATCGACCAGC-2 2202 4147 4
Donor1-CTGCGGATCGTGACAT-2 2403 5231 1
Donor1-CTGCTGTGTCCCTACT-2 2496 4757 3
Donor1-CTGGTCTAGCTGAAAT-2 1580 3572 6
Donor1-CTGTGCTCAGGTCCAC-2 3747 9852 1
Donor1-CTGTGCTTCACATGCA-2 2109 6351 13
Donor1-CTGTGCTTCCTACAGA-2 2126 8588 8
Donor1-CTGTGCTTCCTTGCCA-2 3594 8425 4
Donor1-CTGTTTATCCACGAAT-2 5230 19210 2
Donor1-CTTAACTAGCCTTGAT-2 1747 7624 7
Donor1-CTTACCGAGGAGTTGC-2 5171 19563 6
Donor1-CTTACCGCAATGACCT-2 5641 19283 1
Donor1-CTTACCGGTGTGAAAT-2 2162 6699 13
Donor1-CTTACCGTCAGCAACT-2 2792 5822 5
Donor1-CTTACCGTCTACCAGA-2 2131 6846 9
Donor1-CTTAGGAAGATACACA-2 2970 7003 2
Donor1-CTTAGGACATCCAACA-2 2224 8077 9
Donor1-CTTAGGAGTCCCTACT-2 1653 5463 9

Donor1-CTTAGGAGTTCCCTTG-2 2793 5762 3
Donor1-CTTAGGATCTGTTTGT-2 6913 38497 4
Donor1-CTTCTCTAGACACGAC-2 3793 9839 3
Donor1-CTTCTCTCATTGTGCA-2 1699 4593 7
Donor1-CTTCTCTTCTGCAAGT-2 2115 6139 7
Donor1-CTTGGCTAGGCATGGT-2 2600 10009 7
Donor1-CTTGGCTAGTCGATAA-2 2990 19719 8
Donor1-CTTGGCTCATCCAACA-2 3812 9250 3
Donor1-GAAATGACATTTGCTT-2 5018 19763 6
Donor1-GAACATCAGCTAGTGG-2 1512 3983 13
Donor1-GAACCTAAGGTGCACA-2 2257 3608 6
Donor1-GAACCTAAGGTGGGTT-2 1993 10688 8
Donor1-GAACCTACAAAGTGCG-2 3387 8213 3
Donor1-GAACCTAGTGTATGGG-2 1255 4458 8
Donor1-GAACCTATCTGGCGTG-2 3389 7456 3
Donor1-GAACGGACACAAGCCC-2 6014 35012 6
Donor1-GAAGCAGGTTTGGGCC-2 2165 11014 8
Donor1-GAAGCAGTCACCCGAG-2 2150 3874 5
Donor1-GAATGAACAGTCCTTC-2 1145 3526 8
Donor1-GAATGAAGTAAATGAC-2 1213 3702 13
Donor1-GAATGAAGTACCTACA-2 1173 3632 8
Donor1-GAATGAATCAACCAAC-2 1413 4862 8
Donor1-GACACGCAGCTACCTA-2 5700 18071 2
Donor1-GACACGCCAGCCTTTC-2 3033 6648 5
Donor1-GACACGCCATATGGTC-2 3685 12240 11
Donor1-GACAGAGCATCACGAT-2 1726 3687 4
Donor1-GACAGAGGTACTCAAC-2 3627 7839 3
Donor1-GACAGAGTCGAGAACG-2 4002 9755 2
Donor1-GACCAATAGCGCTTAT-2 3720 12631 11
Donor1-GACCAATCACAACGTT-2 2166 4462 6
Donor1-GACCAATGTTTGGCGC-2 7078 23043 2
Donor1-GACCAATTCGCTTGTC-2 895 3498 10
Donor1-GACCTGGGTAAATACG-2 1617 4310 13
Donor1-GACCTGGTCGAATCCA-2 2430 7551 10
Donor1-GACGCGTAGAACTCGG-2 1825 6070 9
Donor1-GACGCGTAGAATGTTG-2 2308 6887 10
Donor1-GACGCGTGTAGATTAG-2 1389 4680 13
Donor1-GACGTGCAGCGTGAGT-2 2670 5437 3
Donor1-GACGTGCAGGATTCGG-2 3626 8353 3
Donor1-GACGTGCAGTTTCCTT-2 1185 4119 8
Donor1-GACGTGCCAGACACTT-2 3151 7022 4
Donor1-GACGTGCCAGTAAGAT-2 5560 24018 1
Donor1-GACGTTAAGTCTCAAC-2 3376 8007 6
Donor1-GACTAACGTAGCGTGA-2 2981 6690 3
Donor1-GACTAACGTGGTACAG-2 2158 10021 8
Donor1-GACTAACTCGGCGCAT-2 5719 19728 6
Donor1-GACTACAGTGCCTTGG-2 6500 26101 2
Donor1-GACTACAGTTCCGTCT-2 2053 7791 9
Donor1-GACTGCGGTAGTGAAT-2 3088 7415 3
Donor1-GAGCAGAGTCTAGGTT-2 2389 5050 3
Donor1-GAGCAGATCTCGATGA-2 2407 9359 9
Donor1-GAGGTGAAGTCAAGCG-2 1985 5215 6
Donor1-GAGGTGACACACGCTG-2 1376 4058 13
Donor1-GAGGTGACAGACAAGC-2 1888 6607 7
Donor1-GAGGTGAGTTCTGTTT-2 1643 5959 8
Donor1-GAGTCCGAGCAATATG-2 1682 5075 13

Donor1-GAGTCCGCAAGGTTCT-2 1735 5872 9
Donor1-GAGTCCGTCTAGCACA-2 1633 5975 13
Donor1-GATCAGTTCAACGGCC-2 2149 6961 13
Donor1-GATCAGTTCTACTATC-2 1319 3736 8
Donor1-GATCGATAGGGTCGAT-2 2408 10769 6
Donor1-GATCGATGTAATCGTC-2 6454 24495 4
Donor1-GATCGCGAGAAGGCCT-2 4100 11703 3
Donor1-GATCGTATCGCTGATA-2 1569 4435 13
Donor1-GATCTAGAGTTGAGAT-2 4320 11308 2
Donor1-GATGAAAAGCTGATAA-2 6332 29104 4
Donor1-GATGAAACACATCCAA-2 6079 21138 1
Donor1-GATGAGGTCTACGAGT-2 3015 6606 3
Donor1-GATGCTACATCTGGTA-2 2519 10534 7
Donor1-GATGCTATCATGTCTT-2 2380 4211 1
Donor1-GATGCTATCTAACCGA-2 3160 7312 6
Donor1-GATTCAGAGGCATGTG-2 3518 9092 12
Donor1-GATTCAGCACACTGCG-2 1587 5472 8
Donor1-GCAAACTCAAGTACCT-2 1296 3702 9
Donor1-GCAAACTTCATGTGGT-2 1498 3608 7
Donor1-GCAATCAGTAGCGATG-2 1725 5820 13
Donor1-GCAATCATCCAAACAC-2 4818 17423 6
Donor1-GCACATAAGATCGATA-2 2078 4613 6
Donor1-GCACTCTAGGGTTTCT-2 4979 14405 2
Donor1-GCACTCTGTGTCAATC-2 1936 5965 13
Donor1-GCAGCCAAGTCCCACG-2 1460 5037 8
Donor1-GCAGCCAGTGTGACCC-2 2289 4063 2
Donor1-GCAGTTAAGAGTAAGG-2 3516 8287 4
Donor1-GCATACAAGCTGAAAT-2 1975 6615 13
Donor1-GCATACACAATAGCGG-2 2185 7534 7
Donor1-GCATACAGTTCCGTCT-2 4213 10709 2
Donor1-GCATACATCTGAGTGT-2 1697 7311 8
Donor1-GCATGATTCCGTAGGC-2 1715 6667 9
Donor1-GCATGCGAGACCGGAT-2 4061 9856 3
Donor1-GCATGCGAGATCTGAA-2 4194 17696 6
Donor1-GCATGCGCATCCTTGC-2 4467 11293 5
Donor1-GCATGTAAGTAATCCC-2 1185 3496 8
Donor1-GCATGTAGTGTGCCTG-2 3130 7241 3
Donor1-GCATGTATCCTGCTTG-2 3630 10431 6
Donor1-GCATGTATCTAACGGT-2 2008 7164 13
Donor1-GCCAAATAGCGAGAAA-2 2607 5110 2
Donor1-GCCAAATAGTGGCACA-2 5086 20763 4
Donor1-GCCTCTAAGCTACCGC-2 3680 8788 4
Donor1-GCCTCTATCATTTGGG-2 3362 8418 3
Donor1-GCGACCAAGCCCAGCT-2 4353 11957 3
Donor1-GCGAGAAAGACCCACC-2 4680 13824 1
Donor1-GCGAGAAGTCACACGC-2 3482 9255 2
Donor1-GCGCAACGTATCTGCA-2 3594 8813 3
Donor1-GCGCAACGTGAGCGAT-2 2503 4563 6
Donor1-GCGCCAAAGCCAACAG-2 1527 4724 13
Donor1-GCGCGATCACTGTGTA-2 1162 3986 8
Donor1-GCGCGATCATGCAACT-2 1259 3717 7
Donor1-GCGCGATGTGTTGAGG-2 1365 4952 8
Donor1-GCGCGATTCAGTTCGA-2 1745 6032 7
Donor1-GCGGGTTAGCGTGTCC-2 3554 7551 5
Donor1-GCGGGTTCAAGCGTAG-2 2300 3608 5
Donor1-GCGGGTTGTTATTCTC-2 3444 22081 7

Donor1-GCGGGTTTCGATGAGG-2 7020 37401 4
Donor1-GCTCTGTCAGGATCGA-2 3860 10124 4
Donor1-GCTGCAGAGACATAAC-2 2280 6339 13
Donor1-GCTGCAGTCCGTTGTC-2 1675 4585 7
Donor1-GCTGCGAAGAGAACAG-2 2914 10859 6
Donor1-GCTGCGACACCTTGTC-2 2904 8529 11
Donor1-GCTGCGACATCGATTG-2 2078 7831 9
Donor1-GCTGCGAGTTCAGTAC-2 4146 11302 5
Donor1-GCTGCTTAGATCCTGT-2 1851 5989 10
Donor1-GCTGCTTAGCTGCCCA-2 1394 4445 8
Donor1-GCTGCTTCACTTCGAA-2 2259 7340 11
Donor1-GCTGCTTCATTGCGGC-2 2204 7701 13
Donor1-GCTGCTTGTTCCCTTG-2 1949 9375 8
Donor1-GCTGGGTAGGGTCTCC-2 1246 3798 13
Donor1-GCTGGGTGTCTTGTCC-2 1203 3834 8
Donor1-GCTTCCACAGCCAATT-2 2147 6250 7
Donor1-GCTTCCACAGTGAGTG-2 1695 4826 13
Donor1-GCTTCCAGTCGGCACT-2 1692 6137 7
Donor1-GCTTCCAGTGTGAAAT-2 1487 3972 13
Donor1-GCTTCCATCAGCGACC-2 1761 5375 7
Donor1-GCTTGAAAGACAAAGG-2 5201 17955 2
Donor1-GCTTGAAAGAGGGATA-2 6793 34642 5
Donor1-GCTTGAAGTGACGCCT-2 3645 9148 3
Donor1-GCTTGAAGTGTCAATC-2 1507 5229 9
Donor1-GCTTGAAGTTACGTCA-2 1347 3534 7
Donor1-GCTTGAATCGGTCTAA-2 1215 3787 8
Donor1-GGAAAGCCAGCGTTCG-2 3497 12798 12
Donor1-GGAAAGCCAGTCAGCC-2 2204 6562 10
Donor1-GGAAAGCGTTGACGTT-2 1320 4704 8
Donor1-GGAAAGCGTTGCCTCT-2 2357 5180 3
Donor1-GGAAAGCTCAACACTG-2 2709 5164 6
Donor1-GGAAAGCTCCCAAGAT-2 1429 4998 8
Donor1-GGAACTTAGATCCTGT-2 2685 5353 1
Donor1-GGAACTTCAATGACCT-2 4106 11566 4
Donor1-GGAACTTCACTGAAGG-2 1769 3618 6
Donor1-GGAACTTTCACAGGCC-2 2464 8084 10
Donor1-GGAATAACATAGACTC-2 1453 3549 13
Donor1-GGAATAAGTCGAAAGC-2 4643 14393 1
Donor1-GGAATAAGTCTAGCCG-2 6857 31446 2
Donor1-GGACAAGAGAGCTGCA-2 1317 4135 8
Donor1-GGACAAGCACGTTGGC-2 4071 11480 3
Donor1-GGACAAGCATCAGTAC-2 5545 19536 3
Donor1-GGACAAGGTCGCTTCT-2 2347 4718 1
Donor1-GGACAAGTCACAATGC-2 1640 3934 11
Donor1-GGACAAGTCAGCATGT-2 1865 7315 9
Donor1-GGACAGAAGAATAGGG-2 1180 3728 8
Donor1-GGACATTAGTATTGGA-2 2710 5453 6
Donor1-GGACATTTCAAACCAC-2 4925 14706 6
Donor1-GGAGCAAGTTCCGGCA-2 2041 5240 10
Donor1-GGAGCAAGTTCTGAAC-2 3228 26381 8
Donor1-GGAGCAATCACATGCA-2 2032 4705 2
Donor1-GGAGCAATCCTAGGGC-2 1548 4520 13
Donor1-GGATGTTAGGACGAAA-2 4675 14044 2
Donor1-GGATGTTAGTCCGGTC-2 2817 5606 2
Donor1-GGATGTTGTGCAGTAG-2 2949 7072 3
Donor1-GGATGTTGTGTCCTCT-2 1382 4237 8

Donor1-GGATGTTGTGTGAAAT-2 6509 24029 3
Donor1-GGATTACAGTCAATAG-2 1170 3572 8
Donor1-GGATTACCATCGGTTA-2 2956 6910 6
Donor1-GGCAATTCATCCCATC-2 2103 5958 10
Donor1-GGCAATTGTCATGCCG-2 1420 3746 13
Donor1-GGCAATTGTTACGGAG-2 1350 4859 9
Donor1-GGCAATTTCACTCTTA-2 6407 22569 3
Donor1-GGCAATTTCAGAGACG-2 3398 16629 7
Donor1-GGCAATTTCGCCTGAG-2 3635 7556 2
Donor1-GGCCGATAGATTACCC-2 5217 20291 3
Donor1-GGCCGATAGCAGGTCA-2 1139 4082 8
Donor1-GGCCGATCAAAGCAAT-2 1292 4110 8
Donor1-GGCCGATTCGCTTGTC-2 1432 4262 7
Donor1-GGCCGATTCTCGTTTA-2 4049 12727 6
Donor1-GGCGACTAGTAGATGT-2 2785 13488 7
Donor1-GGCGACTCACTCGACG-2 1820 3794 6
Donor1-GGCGACTGTTACGTCA-2 1389 4103 13
Donor1-GGCTCGACATGTCTCC-2 6288 24133 1
Donor1-GGCTCGAGTGACGGTA-2 2627 6564 6
Donor1-GGCTGGTGTTCAGTAC-2 1765 4606 7
Donor1-GGGAATGAGGTGCACA-2 2265 9135 8
Donor1-GGGAATGAGTAACCCT-2 2805 10728 7
Donor1-GGGAATGTCCGCATCT-2 1282 4728 8
Donor1-GGGACCTCAACGATCT-2 3116 6735 1
Donor1-GGGAGATTCTTCCTTC-2 5620 19849 4
Donor1-GGGATGAAGAGGACGG-2 5200 16273 2
Donor1-GGGATGACACACGCTG-2 6492 32590 4
Donor1-GGGATGACAGGCTCAC-2 2447 4766 3
Donor1-GGGATGACATTCTCAT-2 1394 4510 8
Donor1-GGGATGATCATGTAGC-2 2152 3773 4
Donor1-GGGCACTGTCCGAAGA-2 3448 7837 3
Donor1-GGGCATCCAACGCACC-2 1945 8504 8
Donor1-GGGCATCGTCACCCAG-2 2036 7177 7
Donor1-GGGTCTGAGCAATCTC-2 2048 3547 6
Donor1-GGGTCTGAGCTGATAA-2 1324 3650 8
Donor1-GGGTCTGGTCGACTAT-2 1565 4130 7
Donor1-GGGTCTGTCTTCGAGA-2 2142 4236 6
Donor1-GGGTTGCGTACGAAAT-2 1526 6463 8
Donor1-GGTATTGAGCTCAACT-2 3064 6774 1
Donor1-GGTATTGAGGGCATGT-2 2354 5588 6
Donor1-GGTATTGGTCATTAGC-2 5452 17461 1
Donor1-GGTATTGTCCTAGGGC-2 2947 5054 5
Donor1-GGTGAAGTCAGAGACG-2 2760 6874 2
Donor1-GGTGAAGTCCGAACGC-2 2166 4657 11
Donor1-GGTGAAGTCCTAGGGC-2 2536 5566 3
Donor1-GGTGCGTTCTTACCTA-2 2913 15757 12
Donor1-GTAACGTAGGACAGCT-2 1655 6249 7
Donor1-GTAACGTCAAAGGTGC-2 4608 12459 6
Donor1-GTAACGTCACTTACGA-2 3228 7701 3
Donor1-GTAACGTCATTGAGCT-2 1432 3474 13
Donor1-GTAACGTGTCGGATCC-2 1594 6671 8
Donor1-GTAACGTTCACATAGC-2 5451 18234 1
Donor1-GTAACGTTCCATGCTC-2 1727 5429 7
Donor1-GTAACGTTCGGAGGTA-2 1309 3484 7
Donor1-GTAACGTTCGGATGTT-2 3003 6298 6
Donor1-GTAACTGAGACGCACA-2 6793 32499 1

Donor1-GTACGTAAGTACACCT-2 3970 10628 4
Donor1-GTACGTATCTTGTATC-2 4493 12384 2
Donor1-GTACTCCGTCGAGTTT-2 1209 4226 9
Donor1-GTACTCCTCACTCCTG-2 4217 10875 1
Donor1-GTACTCCTCGAACTGT-2 1861 5919 7
Donor1-GTACTTTAGGCCCGTT-2 1445 3567 11
Donor1-GTACTTTCACTTCTGC-2 1903 7648 8
Donor1-GTACTTTCAGCGATCC-2 4707 13822 1
Donor1-GTACTTTGTAGGGTAC-2 4048 15690 10
Donor1-GTAGGCCAGCCGTCGT-2 2169 4168 2
Donor1-GTAGGCCTCCCTAACC-2 4706 14083 1
Donor1-GTAGTCAGTCCGAACC-2 3062 10776 12
Donor1-GTATCTTCAACTGCGC-2 3458 12727 11
Donor1-GTATCTTTCCTAAGTG-2 2803 10121 9
Donor1-GTATTCTGTTATCGGT-2 2257 5850 6
Donor1-GTATTCTTCGTAGGTT-2 1275 4403 8
Donor1-GTCAAGTGTGCCTGGT-2 2997 6800 2
Donor1-GTCAAGTTCCAGAGGA-2 2132 5941 12
Donor1-GTCACAACATGCTAGT-2 1525 4459 13
Donor1-GTCACAAGTAATAGCA-2 1166 3524 8
Donor1-GTCACAAGTTTGCATG-2 3764 12919 6
Donor1-GTCACAATCTGGTTCC-2 1802 7126 8
Donor1-GTCACGGAGCGAGAAA-2 2267 5672 11
Donor1-GTCACGGAGTGGGTTG-2 2808 6022 4
Donor1-GTCACGGAGTGTTTGC-2 2737 5895 3
Donor1-GTCACGGCAAAGTGCG-2 2342 6185 6
Donor1-GTCATTTCATCTATGG-2 1670 4097 10
Donor1-GTCATTTTCCTAAGTG-2 2526 8736 7
Donor1-GTCCTCAAGACGACGT-2 3484 8777 3
Donor1-GTCCTCAAGCTCCCAG-2 2545 4346 5
Donor1-GTCCTCAAGCTGCCCA-2 2881 10752 10
Donor1-GTCCTCAAGTAACCCT-2 2283 4643 3
Donor1-GTCGGGTAGATCGATA-2 1722 5113 13
Donor1-GTCGGGTGTACGCACC-2 2008 4986 13
Donor1-GTCGGGTGTATGGTTC-2 1545 5575 8
Donor1-GTCGGGTGTCGGATCC-2 2310 12297 8
Donor1-GTCGGGTGTTTGGCGC-2 1741 3496 4
Donor1-GTCGGGTTCCTCAATT-2 2072 4977 10
Donor1-GTCGTAAAGTTGCAGG-2 1633 5414 9
Donor1-GTCGTAACAATCCAAC-2 1483 4340 13
Donor1-GTCGTAACAGTTCATG-2 2625 4315 5
Donor1-GTCGTAAGTAGAAAGG-2 3222 9718 11
Donor1-GTCGTAAGTGTGGTTT-2 2023 7987 7
Donor1-GTCGTAATCTCGCATC-2 4033 10912 1
Donor1-GTCTTCGCAGATCCAT-2 2409 5925 6
Donor1-GTCTTCGCATATGCTG-2 5632 18701 2
Donor1-GTCTTCGGTAGCGTAG-2 1376 5520 8
Donor1-GTGAAGGCAGATCCAT-2 2882 13002 7
Donor1-GTGCAGCTCGTCGTTC-2 5383 30662 5
Donor1-GTGCATAAGTACATGA-2 3389 16290 7
Donor1-GTGCATATCTTACCTA-2 2319 5787 6
Donor1-GTGCGGTAGCTGTTCA-2 1586 4968 13
Donor1-GTGCGGTCAAGTTAAG-2 1097 3609 8
Donor1-GTGCGGTCACCTATCC-2 2008 7384 9
Donor1-GTGCGGTGTACGACCC-2 2970 6271 3
Donor1-GTGCGGTTCAGCATGT-2 1662 4425 13

Donor1-GTGCTTCAGTTAACGA-2 2059 7188 9
Donor1-GTGCTTCAGTTGCAGG-2 2582 5497 3
Donor1-GTGCTTCCAGTAACGG-2 3967 10412 6
Donor1-GTGGGTCTCCCTAATT-2 4564 11889 2
Donor1-GTGTTAGCACCGAATT-2 3321 8494 3
Donor1-GTTAAGCAGACAATAC-2 2834 6659 3
Donor1-GTTAAGCAGCTGCCCA-2 1626 6572 8
Donor1-GTTAAGCCAAGCGATG-2 1814 6097 9
Donor1-GTTAAGCCACGCTTTC-2 1366 4021 7
Donor1-GTTAAGCGTATCACCA-2 4172 10233 5
Donor1-GTTACAGAGAACAATC-2 1535 6336 8
Donor1-GTTACAGTCAGCCTAA-2 2264 6848 10
Donor1-GTTCATTAGTGTACGG-2 1407 3955 13
Donor1-GTTCATTCAACAACCT-2 3670 11476 11
Donor1-GTTCATTCACCAGGTC-2 3030 6644 3
Donor1-GTTCATTCAGCCTGTG-2 3565 7718 3
Donor1-GTTCATTCATGTCCTC-2 3267 8244 3
Donor1-GTTCATTGTCAGGACA-2 1600 3895 10
Donor1-GTTCGGGAGAATTCCC-2 3627 9844 4
Donor1-GTTCGGGCAATCGGTT-2 3091 9303 6
Donor1-GTTCGGGGTGCTTCTC-2 1987 5010 6
Donor1-GTTCGGGGTGTCGCTG-2 4412 13785 3
Donor1-GTTCTCGTCAGGCGAA-2 1624 3941 12
Donor1-GTTCTCGTCTCTGTCG-2 3243 7509 4
Donor1-TAAACCGCATTCACTT-2 3074 6954 3
Donor1-TAAACCGGTCCAGTGC-2 2788 6128 2
Donor1-TAAGAGACATGGTTGT-2 2798 6484 4
Donor1-TAAGAGATCAAGCCTA-2 3064 7524 5
Donor1-TAAGAGATCCTTCAAT-2 7140 25657 3
Donor1-TAAGCGTGTAAGAGGA-2 4241 12724 6
Donor1-TAAGCGTGTACCAGTT-2 1938 9817 8
Donor1-TAAGCGTTCGTCTGAA-2 1796 5649 13
Donor1-TAAGTGCCATCGGAAG-2 1341 3924 8
Donor1-TACACGAAGCACGCCT-2 1159 3663 8
Donor1-TACACGAGTACCGTTA-2 1569 4436 7
Donor1-TACAGTGAGCGTGAAC-2 1838 7954 8
Donor1-TACCTATCAAACCTAC-2 5649 18635 1
Donor1-TACCTATTCGCTAGCG-2 2069 5319 10
Donor1-TACCTTAGTTCACCTC-2 1614 3969 8
Donor1-TACCTTATCCAGGGCT-2 1666 5835 7
Donor1-TACCTTATCCCTAATT-2 2872 6477 1
Donor1-TACGGATAGTTTAGGA-2 3655 9810 1
Donor1-TACGGATGTAAGTTCC-2 2138 11521 8
Donor1-TACGGATTCGCGATCG-2 7015 55070 4
Donor1-TACGGGCCAGGATTGG-2 2192 6861 11
Donor1-TACGGGCCATTGGGCC-2 3535 7915 2
Donor1-TACTCATAGTACCGGA-2 2681 5416 5
Donor1-TACTCATCAAACTGCT-2 1812 5825 13
Donor1-TACTCATTCACGATGT-2 7713 39229 5
Donor1-TACTCATTCACTTCAT-2 3282 7091 1
Donor1-TACTCGCAGACACGAC-2 2594 9811 9
Donor1-TACTCGCAGCGTAGTG-2 1391 3764 13
Donor1-TACTCGCAGGTGATTA-2 1273 4248 8
Donor1-TACTCGCCAGTCAGCC-2 1891 8104 8
Donor1-TACTCGCTCTCTGTCG-2 3862 17347 6
Donor1-TACTTACAGGTGCACA-2 1735 5692 13

Donor1-TACTTACGTCCAACTA-2 5156 15848 1
Donor1-TACTTACGTTGTCGCG-2 1409 5013 8
Donor1-TAGACCACATCCCACT-2 2005 4903 10
Donor1-TAGACCAGTACCAGTT-2 1556 3956 13
Donor1-TAGACCAGTTACGGAG-2 2866 9536 6
Donor1-TAGACCAGTTATTCTC-2 1743 5250 13
Donor1-TAGACCATCCATGAAC-2 1231 3472 7
Donor1-TAGAGCTCAGTGACAG-2 1919 6139 9
Donor1-TAGAGCTTCCACTCCA-2 2613 9648 9
Donor1-TAGCCGGCACCACCAG-2 1038 3951 8
Donor1-TAGCCGGTCCTGTAGA-2 5089 24169 5
Donor1-TAGCCGGTCGCTGATA-2 4257 9755 3
Donor1-TAGGCATTCTCCAACC-2 1477 4400 7
Donor1-TAGTGGTCAATGACCT-2 3615 8728 4
Donor1-TAGTGGTCATTAGCCA-2 3434 7933 3
Donor1-TAGTTGGAGCGCTTAT-2 2208 3857 11
Donor1-TAGTTGGCAGCCTTTC-2 4075 16713 6
Donor1-TAGTTGGCAGGGAGAG-2 2089 3663 6
Donor1-TAGTTGGGTCTTTCAT-2 1249 4614 8
Donor1-TAGTTGGTCTTGTTTG-2 4444 12122 3
Donor1-TATCAGGAGGACACCA-2 1560 4130 13
Donor1-TATCAGGCATAAGACA-2 3506 9221 6
Donor1-TATCAGGCATACAGCT-2 1692 4767 11
Donor1-TATCAGGGTTGACGTT-2 2317 8645 13
Donor1-TATTACCAGAGAGCTC-2 6999 28688 1
Donor1-TCAACGAAGTGGGTTG-2 3419 7916 4
Donor1-TCAACGAGTACACCGC-2 1234 3939 8
Donor1-TCAATCTAGAGGTTAT-2 1733 4026 10
Donor1-TCAATCTAGGACACCA-2 4192 10456 5
Donor1-TCAATCTAGTAGTGCG-2 1281 4043 13
Donor1-TCAATCTCACCAGATT-2 3155 6496 3
Donor1-TCACAAGTCCCAGGTG-2 2289 8710 13
Donor1-TCACGAAAGCAGGCTA-2 1205 4536 8
Donor1-TCACGAACAGTCAGCC-2 3101 7146 3
Donor1-TCACGAAGTCTCCCTA-2 1867 5147 13
Donor1-TCAGCAAAGAGGGATA-2 4063 11689 3
Donor1-TCAGCAAAGTGTACTC-2 1912 6129 13
Donor1-TCAGCAACACAACGTT-2 7223 38949 5
Donor1-TCAGCAACAGGAACGT-2 3635 9128 2
Donor1-TCAGCAAGTGTGAAAT-2 2089 10736 8
Donor1-TCAGCTCCATCCTAGA-2 3624 8204 3
Donor1-TCAGGATCAACACGCC-2 1262 3570 8
Donor1-TCAGGATGTAAACACA-2 1746 4853 13
Donor1-TCAGGTAAGTGTACGG-2 2259 4293 6
Donor1-TCATTACAGTAGCGGT-2 4090 14584 6
Donor1-TCATTACCAATAACGA-2 2428 7869 10
Donor1-TCATTACCACTGTGTA-2 3580 12957 11
Donor1-TCATTTGCATGTAGTC-2 3555 7606 2
Donor1-TCATTTGGTTGTGGAG-2 1290 4971 8
Donor1-TCATTTGTCATCGGAT-2 1087 3531 8
Donor1-TCATTTGTCTCTTATG-2 1420 3511 13
Donor1-TCCACACTCGGAATCT-2 1502 3858 13
Donor1-TCCCGATAGAGTGAGA-2 3618 8257 5
Donor1-TCCCGATAGTGTACGG-2 1372 4368 9
Donor1-TCCCGATCACGGCCAT-2 1549 6927 8
Donor1-TCCCGATTCACATGCA-2 1252 4009 8

Donor1-TCGAGGCGTGGTCTCG-2 2061 5256 10
Donor1-TCGAGGCTCTTAGAGC-2 2349 3893 5
Donor1-TCGCGAGGTCAGCTAT-2 4787 13170 2
Donor1-TCGCGAGTCACCACCT-2 5404 19245 3
Donor1-TCGCGAGTCAGCACAT-2 5051 14574 3
Donor1-TCGCGAGTCCCACTTG-2 1962 4999 11
Donor1-TCGCGAGTCGTACCGG-2 1701 7835 8
Donor1-TCGCGTTGTACCGAGA-2 1758 4662 13
Donor1-TCGCGTTGTGCACCAC-2 1422 4924 8
Donor1-TCGGTAAAGGCGATAC-2 1758 6544 13
Donor1-TCGGTAAGTCGGATCC-2 2865 6914 3
Donor1-TCGGTAAGTCGGCACT-2 7804 47685 4
Donor1-TCGGTAAGTCTGATCA-2 2989 6018 5
Donor1-TCGGTAATCACAACGT-2 2891 6030 5
Donor1-TCGTACCCAGCCACCA-2 1260 4182 13
Donor1-TCGTAGACAAACAACA-2 4131 10325 3
Donor1-TCGTAGAGTGCAGACA-2 2073 5881 6
Donor1-TCTCATACAAGCGATG-2 1626 4433 7
Donor1-TCTCATAGTAAAGTCA-2 1191 3848 8
Donor1-TCTCATAGTCAAAGCG-2 2437 5210 3
Donor1-TCTCTAATCTAACCGA-2 2631 5037 6
Donor1-TCTGAGAAGTTGAGAT-2 7345 39546 4
Donor1-TCTGAGAAGTTGTAGA-2 3914 16858 6
Donor1-TCTGAGAGTAGCTAAA-2 4417 24761 7
Donor1-TCTGGAAAGGGTGTTG-2 3775 9299 4
Donor1-TCTGGAACACTGTGTA-2 2886 10403 9
Donor1-TCTGGAACAGCCTTGG-2 2934 6892 3
Donor1-TCTGGAATCATTATCC-2 6112 20175 2
Donor1-TCTGGAATCCTTGGTC-2 2071 4049 6
Donor1-TCTGGAATCTGTCTAT-2 3218 6863 2
Donor1-TCTTCGGTCCTTGGTC-2 2737 5949 3
Donor1-TCTTTCCGTGGACGAT-2 4935 14885 4
Donor1-TCTTTCCTCTCTAGGA-2 5864 22525 5
Donor1-TGAAAGACAAGGACAC-2 2855 10271 7
Donor1-TGAAAGACAGTCGTGC-2 4719 53512 8
Donor1-TGAAAGACATGGTCAT-2 3029 7254 4
Donor1-TGAAAGATCGTCGTTC-2 1264 4446 8
Donor1-TGACAACAGTTAGCGG-2 3124 15873 7
Donor1-TGACAACCATGGAATA-2 4337 13253 3
Donor1-TGACAACGTATAAACG-2 8452 74828 6
Donor1-TGACAACGTCAAACTC-2 2563 5372 1
Donor1-TGACAACGTCCGAAGA-2 1847 6541 9
Donor1-TGACGGCAGGTGTTAA-2 1937 3880 5
Donor1-TGACGGCAGTGGGATC-2 3988 10845 3
Donor1-TGACGGCTCTTGAGGT-2 2685 18295 8
Donor1-TGACTAGAGGTGACCA-2 1519 4670 13
Donor1-TGACTAGGTCATACTG-2 2413 4845 5
Donor1-TGACTAGGTCTAAAGA-2 1600 5003 13
Donor1-TGACTAGGTGTGACGA-2 2502 4546 2
Donor1-TGACTTTGTAGAGTGC-2 1840 4903 10
Donor1-TGAGAGGCACGAAAGC-2 2860 11062 7
Donor1-TGAGAGGTCGTCACGG-2 1455 3731 9
Donor1-TGAGCATAGCATCATC-2 5045 15102 3
Donor1-TGAGCCGGTACAAGTA-2 3542 6981 6
Donor1-TGAGCCGGTCCCTTGT-2 1346 4512 13
Donor1-TGAGCCGTCGTAGGAG-2 2605 4739 6

Donor1-TGAGGGAAGATCCCGC-2 1462 4320 13
Donor1-TGAGGGAAGCACGCCT-2 4422 12961 1
Donor1-TGAGGGAAGCTGCCCA-2 7141 59894 4
Donor1-TGATTTCTCCAGAAGG-2 1473 4792 9
Donor1-TGCACCTAGCACCGCT-2 3669 9499 3
Donor1-TGCACCTGTCGCTTCT-2 3618 8152 3
Donor1-TGCACCTTCCATGAAC-2 1373 3839 13
Donor1-TGCCAAATCGCACTCT-2 3566 19125 7
Donor1-TGCCCATAGACTACAA-2 2423 4441 2
Donor1-TGCCCATAGCAGATCG-2 5421 19686 1
Donor1-TGCCCATGTGACGCCT-2 1555 4034 7
Donor1-TGCCCATTCAAGGTAA-2 3752 8517 5
Donor1-TGCCCTACACATCCAA-2 2840 5840 2
Donor1-TGCCCTAGTGCACCAC-2 4751 13918 1
Donor1-TGCCCTATCACAACGT-2 2184 8553 7
Donor1-TGCGCAGGTATGAATG-2 3696 9970 3
Donor1-TGCGGGTGTACCCAAT-2 3751 9473 3
Donor1-TGCGGGTTCCAGATCA-2 1906 6392 9
Donor1-TGCGTGGAGCTGCGAA-2 2588 5961 6
Donor1-TGCGTGGGTCCGTCAG-2 2960 11350 7
Donor1-TGCGTGGGTTGGTAAA-2 2037 3775 5
Donor1-TGCGTGGTCGGATGGA-2 4497 13528 1
Donor1-TGCTACCCACGAAGCA-2 1830 6192 8
Donor1-TGCTACCCATTCCTGC-2 1863 5535 13
Donor1-TGCTGCTCAGTTAACC-2 1427 5701 8
Donor1-TGGACGCTCACATGCA-2 1493 4014 13
Donor1-TGGACGCTCAGCGATT-2 3056 6726 4
Donor1-TGGACGCTCTCGAGTA-2 1952 8200 9
Donor1-TGGCCAGAGCTACCGC-2 5003 16133 2
Donor1-TGGCCAGCACCACCAG-2 5345 17081 2
Donor1-TGGCCAGGTGACGCCT-2 1639 6494 8
Donor1-TGGCCAGGTTGCTCCT-2 5189 17151 2
Donor1-TGGCCAGTCGGCGCTA-2 1318 4267 8
Donor1-TGGCTGGGTACTTAGC-2 5819 20020 3
Donor1-TGGCTGGGTGACAAAT-2 1545 5984 8
Donor1-TGGCTGGGTTCAGCGC-2 1666 7749 8
Donor1-TGGGAAGAGCTCCCAG-2 1639 4535 13
Donor1-TGGGAAGTCGTCCAGG-2 2013 4426 4
Donor1-TGGGCGTGTCTCAACA-2 7662 36736 3
Donor1-TGGTTCCCAACACGCC-2 1237 3783 8
Donor1-TGGTTCCGTTGTCTTT-2 2986 10329 6
Donor1-TGGTTCCTCCTGCCAT-2 1920 6423 13
Donor1-TGTATTCAGGCCATAG-2 4975 15338 2
Donor1-TGTATTCAGTTAGCGG-2 5359 18240 1
Donor1-TGTATTCGTCTCCACT-2 2047 5878 10
Donor1-TGTCCCAAGACTTTCG-2 4308 12191 2
Donor1-TGTCCCACATACAGCT-2 1909 4167 3
Donor1-TGTCCCAGTCGTGGCT-2 3613 8920 3
Donor1-TGTTCCGCACCAGTTA-2 2579 7234 4
Donor1-TTAACTCAGAGTGAGA-2 2736 4633 5
Donor1-TTAACTCAGCCATCGC-2 1702 4846 10
Donor1-TTAACTCCAATGACCT-2 1321 3853 13
Donor1-TTAGGACAGTCAAGCG-2 2627 13733 7
Donor1-TTAGGACCACGCTTTC-2 3049 6919 1
Donor1-TTAGGCAAGCACGCCT-2 2099 6395 13
Donor1-TTAGGCAGTCACACGC-2 1689 5716 7

Donor1-TTAGGCATCTCCAGGG-2 2912 6526 3
Donor1-TTAGTTCGTATGAAAC-2 3842 9848 3
Donor1-TTAGTTCTCATTGCGA-2 2180 4130 2
Donor1-TTATGCTGTTATCACG-2 4541 12349 3
Donor1-TTCGAAGGTCCAGTGC-2 3279 9060 1
Donor1-TTCGAAGGTTACGACT-2 2256 7911 13
Donor1-TTCTACAAGTCCAGGA-2 4752 12532 3
Donor1-TTCTTAGAGCCCGAAA-2 1478 5811 8
Donor1-TTGAACGCAATGCCAT-2 1912 4267 6
Donor1-TTGAACGGTAGGCTGA-2 3407 11834 6
Donor1-TTGACTTAGATGCCAG-2 1986 6954 7
Donor1-TTGACTTAGCACCGCT-2 2465 4314 5
Donor1-TTGACTTAGTTAACGA-2 2670 9494 7
Donor1-TTGACTTGTTGGACCC-2 2311 9181 9
Donor1-TTGACTTTCATGTCCC-2 1510 3710 13
Donor1-TTGCCGTGTCCTCTTG-2 4231 11660 2
Donor1-TTGCCGTTCTGTACGA-2 1488 6096 8
Donor1-TTGCGTCAGCGCCTCA-2 1621 6422 8
Donor1-TTGCGTCGTAGCAAAT-2 1666 3869 6
Donor1-TTGCGTCTCCCTTGCA-2 6668 26276 3
Donor1-TTGGAACAGCACCGTC-2 2117 6208 13
Donor1-TTGGAACCAGACAAAT-2 2011 7158 13
Donor1-TTGGAACCATAGAAAC-2 6441 26046 4
Donor1-TTGGAACGTCGAGATG-2 1426 5543 8
Donor1-TTGGCAAAGATAGGAG-2 1341 4410 7
Donor1-TTGGCAACACGACGAA-2 1107 4018 8
Donor1-TTGGCAACAGACAAGC-2 1828 8983 8
Donor1-TTGGCAAGTAAGAGGA-2 3185 7381 3
Donor1-TTGTAGGCAAGCCATT-2 5277 18571 2
Donor1-TTGTAGGGTAGCCTCG-2 1873 7549 8
Donor1-TTGTAGGGTTGGACCC-2 1952 3760 3
Donor1-TTGTAGGTCTTAGCCC-2 2933 9981 10
Donor1-TTTACTGGTGTTGAGG-2 5184 25802 6
Donor1-TTTACTGTCCGAGCCA-2 3612 8628 4
Donor1-TTTATGCAGTTGCAGG-2 2282 8485 7
Donor1-TTTCCTCAGATGCGAC-2 1975 3860 6
Donor1-TTTCCTCAGTAGGCCA-2 5239 14761 1
Donor1-TTTCCTCGTACCGGCT-2 2241 7209 7
Donor1-TTTCCTCGTCGGCTCA-2 2070 4188 5
Donor1-TTTCCTCGTTGGTGGA-2 4244 10530 1
Donor1-TTTCCTCTCCGTTGTC-2 3086 6311 2
Donor1-TTTCCTCTCGTACGGC-2 2178 5796 6
Donor1-TTTGCGCAGGCTCTTA-2 3088 10971 10
Donor1-TTTGCGCTCTCAAACG-2 2391 9978 9
Donor1-TTTGGTTCACCCATGG-2 4501 11400 5
Donor1-TTTGGTTGTTAAGAAC-2 2601 5872 1
Donor1-TTTGGTTTCTGCGGCA-2 2113 6163 13
Donor1-TTTGTCAAGAACTCGG-2 2734 5652 6
Donor1-TTTGTCAAGGATGGTC-2 3099 5943 2
Donor1-TTTGTCAGTAAGGGAA-2 7531 59912 4
Donor1-TTTGTCAGTGTGCGTC-2 1720 6499 13
Donor1-TTTGTCATCCAAACTG-2 3112 5913 5
