# Supplementary material for: The adult human testis transcriptional cell atlas
Source: Cell Res. 2018 Oct 12;28(12):1141–57. doi: 10.1038/s41422-018-0099-2 (PMC6274646; doi:10.1038/s41422-018-0099-2)
Supplement: Supplementary file 10 — Supplementary information, Table S2 [file 41422_2018_99_MOESM10_ESM.pdf]

Supplementary information, Table S2: K-means Clustering of Genes that Show Differential Expression along Germ Cell Development.

Gene	Cluster
SELM	Cluster1
PPDPF	Cluster1
PHPT1	Cluster1
GAPDH	Cluster1
FKBP8	Cluster1
FTL	Cluster1
AK5	Cluster1
MIF	Cluster1
PSMA7	Cluster1
DCAF12	Cluster1
KANSL1	Cluster1
TMEM14C	Cluster1
TUBB	Cluster1
GRN	Cluster1
CRIP2	Cluster1
CCDC109B	Cluster1
AKR1B1	Cluster1
TJP1	Cluster1
RARS	Cluster1
CPEB1	Cluster1
NT5DC1	Cluster1
MTPN	Cluster1
RALY	Cluster1
FMR1	Cluster1
THAP4	Cluster1
AKIRIN2	Cluster1
TANC2	Cluster1
LIN7B	Cluster1
TKT	Cluster1
ARID4A	Cluster1
CDC123	Cluster1
ZBTB43	Cluster1
PTRHD1	Cluster1
RAB11B	Cluster1
WWP1	Cluster1
MGMT	Cluster1
RPL22L1	Cluster1
TADA3	Cluster1
PIAS4	Cluster1
CDC42BPA	Cluster1
FAM150B	Cluster1
SMS	Cluster1
RPL36	Cluster1
RPLP0	Cluster1
RPS18	Cluster1
RPS19	Cluster1
RPS16	Cluster1
SOX4	Cluster1
RHOXF1	Cluster1
RPL13A	Cluster1
SOD1	Cluster1
ASB9	Cluster1
PTGES2	Cluster1
RPS23	Cluster1

MARCKSL1 Cluster1
GTF3A Cluster1
ELAVL2 Cluster1
RPL39 Cluster1
RPL12 Cluster1
HSPB1 Cluster1
RPL14 Cluster1
YWHAB Cluster1
TBCB Cluster1
PSIP1 Cluster1
PDLIM1 Cluster1
GSTP1 Cluster1
GADD45GIP1 Cluster1
TUBA1B Cluster1
APEX1 Cluster1
MAGEB2 Cluster1
ATIC Cluster1
RPS29 Cluster1
PABPC4 Cluster1
ATP1B1 Cluster1
RPS11 Cluster1
IGFBP2 Cluster1
CDCA7L Cluster1
BEND4 Cluster1
COPS6 Cluster1
AKR1A1 Cluster1
NME4 Cluster1
MTCH1 Cluster1
MPG Cluster1
LPIN2 Cluster1
RPL26 Cluster1
SLC25A6 Cluster1
NAB1 Cluster1
BOP1 Cluster1
NME3 Cluster1
DSCC1 Cluster1
TEAD1 Cluster1
ITGB1 Cluster1
EEF1B2 Cluster1
POLRMT Cluster1
CCDC12 Cluster1
PLPP2 Cluster1
TAF6 Cluster1
NFIC Cluster1
RPL27 Cluster1
RBMX2 Cluster1
BARD1 Cluster1
TGIF2 Cluster1
NEIL2 Cluster1
PPP2R1A Cluster1
LRRFIP1 Cluster1
HCFC1R1 Cluster1
TWF2 Cluster1
ZFP36L2 Cluster1
RASSF3 Cluster1
RPS5 Cluster1

POGZ Cluster1
GMDS Cluster1
BNIP3 Cluster1
GAMT Cluster1
PDCL3 Cluster1
HEBP2 Cluster1
INSIG1 Cluster1
GOLIM4 Cluster1
C12orf49 Cluster1
TXNIP Cluster1
APOO Cluster1
CNFN Cluster1
BRMS1 Cluster1
JARID2 Cluster1
LLGL2 Cluster1
DLK1 Cluster1
ANXA11 Cluster1
LRRC42 Cluster1
TSR3 Cluster1
RNF219 Cluster1
SSRP1 Cluster1
HDAC5 Cluster1
SRP72 Cluster1
PTMS Cluster1
NUDT14 Cluster1
RPL4 Cluster1
LUZP4 Cluster1
ADGRL1 Cluster1
TMEM123 Cluster1
CAPG Cluster1
MCUR1 Cluster1
EIF4EBP1 Cluster1
UXS1 Cluster1
PHF13 Cluster1
MCRS1 Cluster1
AC013461.1 Cluster1
HOXB7 Cluster1
BIRC2 Cluster1
ARPC4 Cluster1
TNNT1 Cluster1
PELI2 Cluster1
COX6B1 Cluster1
STRN4 Cluster1
CCDC88C Cluster1
TCEAL4 Cluster1
THAP1 Cluster1
RGMA Cluster1
TOP1MT Cluster1
SH3BGRL3 Cluster1
AGAP1 Cluster1
ZMYND8 Cluster1
RPL38 Cluster1
MAP2K7 Cluster1
ACTR2 Cluster1
SNF8 Cluster1
ATAD3A Cluster1

MAP7D3 Cluster1
RYK Cluster1
ZFHX3 Cluster1
SDC2 Cluster1
NUDT15 Cluster1
DND1 Cluster1
JRK Cluster1
CHTF8 Cluster1
MIER2 Cluster1
LSM10 Cluster1
C12orf45 Cluster1
GNB2 Cluster1
UROS Cluster1
GTDC1 Cluster1
TATDN1 Cluster1
ENAH Cluster1
RCE1 Cluster1
NTHL1 Cluster1
ZFAS1 Cluster1
ATE1 Cluster1
KLHL35 Cluster1
REST Cluster1
PDLIM7 Cluster1
OTUB1 Cluster1
C19orf24 Cluster1
CCDC127 Cluster1
BRD2 Cluster1
SPTAN1 Cluster1
HPS4 Cluster1
SCO2 Cluster1
MYCBP Cluster1
RIT1 Cluster1
STAMBP Cluster1
MIIP Cluster1
FAM195B Cluster1
PPP2R2A Cluster1
NDUFAF6 Cluster1
SSU72 Cluster1
ENO1 Cluster1
ARMC6 Cluster1
GTF2I Cluster1
ECHS1 Cluster1
ATP5D Cluster1
INTS10 Cluster1
TNRC6B Cluster1
CCDC6 Cluster1
SLC41A3 Cluster1
C19orf53 Cluster1
SMAD2 Cluster1
XAB2 Cluster1
ATP1A1 Cluster1
TUFM Cluster1
ASPSCR1 Cluster1
BLOC1S1 Cluster1
MIB2 Cluster1
TPM3 Cluster1

POLD2 Cluster1
PIWIL2 Cluster1
DDX43 Cluster1
EPC1 Cluster1
LBR Cluster1
GALNS Cluster1
KAT6B Cluster1
TDRD1 Cluster1
NUTM2B-AS1 Cluster1
DAZAP2 Cluster1
UACA Cluster1
CBL Cluster1
PABPN1 Cluster1
NDUFS4 Cluster1
PCSK1N Cluster1
ERCC1 Cluster1
AP2S1 Cluster1
RPS4X Cluster1
GNB2L1 Cluster1
ZNF428 Cluster1
LMO4 Cluster1
TPT1 Cluster1
GLTSCR2 Cluster1
SERPINE2 Cluster1
TCF3 Cluster1
SIX1 Cluster1
UTF1 Cluster1
ID4 Cluster1
L1TD1 Cluster1
ID1 Cluster1
USP11 Cluster1
LY6E Cluster1
AES Cluster1
RPL11 Cluster1
THRA Cluster1
IRF2BPL Cluster1
COTL1 Cluster1
KRT18 Cluster1
STK24 Cluster1
RAC3 Cluster1
PMAIP1 Cluster1
NOC4L Cluster1
PALM3 Cluster1
ID2 Cluster1
FAM26F Cluster1
MYO6 Cluster1
RHOC Cluster1
TMEM14A Cluster1
TOMM34 Cluster1
PKN1 Cluster1
FGFR3 Cluster1
TLK1 Cluster1
ADAR Cluster1
DPPA2 Cluster1
SSBP3 Cluster1
C1QTNF4 Cluster1

YPEL2 Cluster1
SUPT5H Cluster1
SERPINI1 Cluster1
MEF2C Cluster1
FST Cluster1
RBFOX3 Cluster1
NFKBIA Cluster1
CCDC106 Cluster1
RPL37 Cluster1
FSD1 Cluster1
NPB Cluster1
BLVRA Cluster1
RABGGTA Cluster1
KLHL23 Cluster1
PRR5 Cluster1
C19orf84 Cluster1
DTNB Cluster1
DUSP5 Cluster1
LY6H Cluster1
CITED2 Cluster1
GCNT2 Cluster1
C12orf75 Cluster1
PODXL2 Cluster1
RAB6B Cluster1
BMPR1B Cluster1
FAM43A Cluster1
SMARCA1 Cluster1
IDH2 Cluster1
GABRB3 Cluster1
MXD4 Cluster1
TCF7L2 Cluster1
KDM1B Cluster1
LINC01511 Cluster1
TSPAN13 Cluster1
DPPA4 Cluster1
CLN6 Cluster1
SMAD9 Cluster1
KCNQ2 Cluster1
TUBA1A Cluster1
SNAPC2 Cluster1
ENHO Cluster1
NPW Cluster1
MFHAS1 Cluster1
ZFAND2B Cluster1
TMEM55A Cluster1
CDK17 Cluster1
STX3 Cluster1
CRTAP Cluster1
HEY1 Cluster1
SPOCD1 Cluster1
EYA2 Cluster1
HACD4 Cluster1
MDFI Cluster1
PIWIL4 Cluster1
ELMSAN1 Cluster1
PARD3 Cluster1

LRIG1 Cluster1
VIPR2 Cluster1
RAB5C Cluster1
RNF187 Cluster1
BNC2 Cluster1
ST3GAL4 Cluster1
BFSP1 Cluster1
ATP9A Cluster1
PCMTD1 Cluster1
SIAH2 Cluster1
IFI16 Cluster1
NFIB Cluster1
EGR4 Cluster1
BAIAP2 Cluster1
CEBPD Cluster1
UPP1 Cluster1
NEDD4L Cluster1
CAPRIN2 Cluster1
TMEM251 Cluster1
RALA Cluster1
CNTNAP2 Cluster1
CCNJL Cluster1
BOK Cluster1
IGLON5 Cluster1
EIF3L Cluster1
HOXC9 Cluster1
KCTD13 Cluster1
RPS10 Cluster1
CLTB Cluster1
ASCL2 Cluster1
RNF130 Cluster1
DMAP1 Cluster1
FAM25G Cluster1
FHL3 Cluster1
DPYSL2 Cluster1
HNRNPH2 Cluster1
SCT Cluster1
SAMD11 Cluster1
RP11-395G23.3 Cluster1
BLMH Cluster1
TRIM71 Cluster1
CHEK2 Cluster1
SERTAD4-AS1 Cluster1
RAB20 Cluster1
NANOS3 Cluster1
ANKRD37 Cluster1
SMARCD3 Cluster1
CAMK2G Cluster1
MB21D2 Cluster1
STX7 Cluster1
MRPL37 Cluster1
RP11-70C1.1 Cluster1
UBA1 Cluster1
RP11-307P5.1 Cluster1
BAMBI Cluster1
ZNF462 Cluster1

LTBP1 Cluster1
TPM2 Cluster1
ADCK3 Cluster1
MROH6 Cluster1
AC002454.1 Cluster1
SSBP2 Cluster1
WIPF3 Cluster1
PFKL Cluster1
FAM21A Cluster1
PAK4 Cluster1
ELK3 Cluster1
PHF6 Cluster1
SHKBP1 Cluster1
RGS10 Cluster1
PLPPR5 Cluster1
RGS14 Cluster1
ROR2 Cluster1
RP11-161M6.2 Cluster1
TBL1X Cluster1
HES1 Cluster1
ZDHHC21 Cluster1
ANXA5 Cluster1
PALM Cluster1
CEBPB Cluster1
LMO2 Cluster1
LINC01582 Cluster1
GPC1 Cluster1
DEAF1 Cluster1
VGLL4 Cluster1
NREP Cluster1
PRKAG2 Cluster1
LINC01030 Cluster1
RGS17 Cluster1
SF3A2 Cluster1
AGAP3 Cluster1
LINC01481 Cluster1
NRG2 Cluster1
TCL1B Cluster1
FAT1 Cluster1
SESN2 Cluster1
AIP Cluster1
SCARB2 Cluster1
PRDX3 Cluster1
REEP2 Cluster1
RNASET2 Cluster1
GAN Cluster1
IMPDH1 Cluster1
PSME1 Cluster1
SPTLC2 Cluster1
DCTN4 Cluster1
SERINC5 Cluster1
B3GALNT1 Cluster1
ARRDC4 Cluster1
IFNAR2 Cluster1
NR1D1 Cluster1
AC018755.17 Cluster1

AK4 Cluster1
SSFA2 Cluster1
PTPRS Cluster1
PLS3 Cluster1
SEPP1 Cluster1
PTPN14 Cluster1
CXCL9 Cluster1
TNFAIP8 Cluster1
MLLT3 Cluster1
TTLL1 Cluster1
CHD7 Cluster1
CERS6 Cluster1
NAB2 Cluster1
ATXN1 Cluster1
GPM6B Cluster1
ERI1 Cluster1
ATP6V0E2 Cluster1
TCN2 Cluster1
SMIM10L1 Cluster1
GSDMD Cluster1
NEK6 Cluster1
PQBP1 Cluster1
ZNF134 Cluster1
CLEC11A Cluster1
GFRA1 Cluster1
TMSB15A Cluster1
BCAM Cluster1
PDZD11 Cluster1
ZNF581 Cluster1
DRAM2 Cluster1
SUSD4 Cluster1
SNX18 Cluster1
TLN2 Cluster1
PCLO Cluster1
AZU1 Cluster1
SNHG19 Cluster1
SERINC2 Cluster1
MAL2 Cluster1
PRKX Cluster1
APBB1 Cluster1
OGFR Cluster1
COBL Cluster1
LIMK1 Cluster1
EPHX2 Cluster1
UNC119 Cluster1
CAMK2B Cluster1
TACC2 Cluster1
TRAF7 Cluster1
TRPM4 Cluster1
DLX5 Cluster1
FAM89A Cluster1
KLF7 Cluster1
RP11-506F3.1 Cluster1
SFXN1 Cluster1
ZNF787 Cluster1
SYNE2 Cluster1

CADM2 Cluster1
ANKRD27 Cluster1
FAM50A Cluster1
CMTM7 Cluster1
DBN1 Cluster1
YPEL3 Cluster1
RPP40 Cluster1
C1orf233 Cluster1
GTPBP6 Cluster1
MT-ATP8 Cluster1
ELOVL4 Cluster1
SATB1 Cluster1
ZGLP1 Cluster1
RBBP5 Cluster1
THAP3 Cluster1
IL27RA Cluster1
RBP4 Cluster1
SCPEP1 Cluster1
SERPING1 Cluster1
KIAA1958 Cluster1
FGFR1 Cluster1
NBL1 Cluster1
TJP2 Cluster1
PPCS Cluster1
DSCR3 Cluster1
ZNF701 Cluster1
ANP32A Cluster1
VPS37D Cluster1
ZEB2 Cluster1
GNAL Cluster1
PDCL Cluster1
C17orf49 Cluster1
MINK1 Cluster1
RP11-973H7.1 Cluster1
SOX17 Cluster1
DGKD Cluster1
STXBP3 Cluster1
SERTAD3 Cluster1
CBR1 Cluster1
MGST3 Cluster1
PPP1R1A Cluster1
CAB39 Cluster1
KCTD2 Cluster1
TMEM181 Cluster1
MARK4 Cluster1
USP12 Cluster1
SP3 Cluster1
HSD17B11 Cluster1
SPR Cluster1
MIR22HG Cluster1
ACAP3 Cluster1
CD151 Cluster1
CRIM1 Cluster1
TSPAN14 Cluster1
CNTNAP3B Cluster1
NANOS2 Cluster1

RFXANK Cluster1
JAK1 Cluster1
MAGEB16 Cluster1
TNIP1 Cluster1
SLC9A3R2 Cluster1
NINJ1 Cluster1
RGS2 Cluster1
PPP2R2D Cluster1
FGFR2 Cluster1
DYRK1A Cluster1
TTC9C Cluster1
RPL17 Cluster1
SFRP1 Cluster1
TFAP2A Cluster1
IRF2BP2 Cluster1
NUDT7 Cluster1
RASL11B Cluster1
SMARCD2 Cluster1
PCAT7 Cluster1
JADE1 Cluster1
CACFD1 Cluster1
SPSB3 Cluster1
NUDT11 Cluster1
STAT1 Cluster1
RPUSD2 Cluster1
ECI2 Cluster1
ZNF516 Cluster1
APBA2 Cluster1
WDR13 Cluster1
KAT2A Cluster1
SPRY1 Cluster1
SETD1A Cluster1
SH2B2 Cluster1
MPDZ Cluster1
BAG6 Cluster1
TMEM39B Cluster1
TMEM219 Cluster1
TPBG Cluster1
RP5-1085F17.3 Cluster1
CTC-241N9.1 Cluster1
11-Sep Cluster1
GNB4 Cluster1
ARPC1B Cluster1
MLYCD Cluster1
COMMD5 Cluster1
CHN1 Cluster1
KLHL42 Cluster1
NIT2 Cluster1
RAB4B Cluster1
VPS26B Cluster1
BRMS1L Cluster1
FYN Cluster1
ASPH Cluster1
RBM38 Cluster1
ZNF322 Cluster1
SKI Cluster1

PREX1 Cluster1
RUNX1T1 Cluster1
RP11-11N9.4 Cluster1
ZRSR2 Cluster1
DEDD2 Cluster1
FN1 Cluster1
GNAQ Cluster1
GNG12 Cluster1
ZDHHC14 Cluster1
AIG1 Cluster1
FAM178B Cluster1
DAB2IP Cluster1
LSR Cluster1
RNF213 Cluster1
PHF21A Cluster1
NFIA Cluster1
CTXN1 Cluster1
ACTN1 Cluster1
DUSP23 Cluster1
IRX2 Cluster1
MYO10 Cluster1
DYRK2 Cluster1
LEPROT Cluster1
AATF Cluster1
DLST Cluster1
PDK2 Cluster1
CYFIP1 Cluster1
ZNF16 Cluster1
SGK3 Cluster1
AFF1 Cluster1
UGCG Cluster1
PRMT7 Cluster1
SWSAP1 Cluster1
MIPEP Cluster1
CCDC94 Cluster1
UBE2L6 Cluster1
FOXP4 Cluster1
PPP2R5D Cluster1
ADNP Cluster1
WASL Cluster1
PEX2 Cluster1
ASUN Cluster1
CUL4A Cluster1
PYM1 Cluster1
LCMT1 Cluster1
FAM13A Cluster1
9-Sep Cluster1
RHOBTB3 Cluster1
CLASP1 Cluster1
VAT1 Cluster1
PPP2R3A Cluster1
ZBTB16 Cluster1
CIART Cluster1
CSNK1E Cluster1
MRAS Cluster1
HIP1R Cluster1

TIAM1 Cluster1
IL13RA1 Cluster1
DOCK8 Cluster1
MBNL1 Cluster1
PRKCI Cluster1
AP001372.2 Cluster1
IRF3 Cluster1
PRR14L Cluster1
BTBD3 Cluster1
RECQL Cluster1
MED27 Cluster1
RABL6 Cluster1
LZTS2 Cluster1
XPOT Cluster1
ADI1 Cluster1
KLC3 Cluster1
LCA5 Cluster1
SIRT5 Cluster1
CEP131 Cluster1
FAM21C Cluster1
SLC25A26 Cluster1
TRIM27 Cluster1
AKT1S1 Cluster1
RNF146 Cluster1
STXBP6 Cluster1
ST20 Cluster1
PRRC2B Cluster1
B4GALT2 Cluster1
YARS2 Cluster1
BAG3 Cluster1
PQLC3 Cluster1
LIG1 Cluster1
ASCC1 Cluster1
ISG15 Cluster1
HRSP12 Cluster1
DUSP4 Cluster1
USP19 Cluster1
ISL1 Cluster1
GUK1 Cluster1
NAA60 Cluster1
SMIM1 Cluster1
BBS2 Cluster1
USP36 Cluster1
RP11-128M1.1 Cluster1
SLC25A1 Cluster1
OSTF1 Cluster1
ZNF414 Cluster1
GPT2 Cluster1
ZNF444 Cluster1
KRT8 Cluster1
C11orf68 Cluster1
TXNRD1 Cluster1
SLC25A13 Cluster1
KBTBD4 Cluster1
2-Mar Cluster1
PHC1 Cluster1

SLC35A1 Cluster1
AGL Cluster1
RP11-693J15.6 Cluster1
ADAMTS6 Cluster1
CYBA Cluster1
NCK2 Cluster1
ZNF90 Cluster1
TPTEP1 Cluster1
FKBP5 Cluster1
CDYL2 Cluster1
ZNF217 Cluster1
CDC34 Cluster1
DCP1A Cluster1
CCDC71 Cluster1
POMGNT2 Cluster1
PHGDH Cluster1
SAMM50 Cluster1
CCDC144NL-AS1 Cluster1
ZC3H3 Cluster1
PIK3C3 Cluster1
A2M Cluster1
GMPR Cluster1
GSN Cluster1
BCAR1 Cluster1
STK38L Cluster1
SAP30BP Cluster1
CPNE1 Cluster1
BIN1 Cluster1
C8orf82 Cluster1
RALB Cluster1
WDR43 Cluster1
FLCN Cluster1
RBKS Cluster1
SPI1 Cluster1
BSDC1 Cluster1
MIF4GD Cluster1
GTF2A1 Cluster1
SYT14 Cluster1
LANCL2 Cluster1
FMN1 Cluster1
HOPX Cluster1
ARID3B Cluster1
MAP3K11 Cluster1
RAB1B Cluster1
C1orf122 Cluster1
LAMTOR2 Cluster1
CD74 Cluster1
JUND Cluster1
PGLS Cluster1
KIAA0232 Cluster1
MIR4458HG Cluster1
CD81 Cluster1
UHMK1 Cluster1
PRDX6 Cluster1
PINX1 Cluster1
HEY2 Cluster1

ITGA6 Cluster1
RBCK1 Cluster1
AHCY Cluster1
BRD4 Cluster1
UQCRC1 Cluster1
MGAT1 Cluster1
KDM2B Cluster1
MLST8 Cluster1
VIM Cluster1
TMEM189 Cluster1
DLGAP4 Cluster1
ETV5 Cluster1
LAMP1 Cluster1
FBXO7 Cluster1
AP2A1 Cluster1
PTGDS cluster10
LYPLA1 cluster10
PSPH cluster10
CCDC168 cluster10
DNAJB7 cluster10
WDR74 cluster10
C10orf120 cluster10
TP53TG5 cluster10
CCDC185 cluster10
G2E3 cluster10
RP11-166P13.4 cluster10
TEKT5 cluster10
C20orf173 cluster10
PTK7 cluster10
SLPI cluster10
C19orf71 cluster10
8-Mar cluster10
CD55 cluster10
ARID2 cluster10
PCMT1 cluster10
GABARAPL1 cluster10
CREB1 cluster10
ZNF677 cluster10
C21orf91 cluster10
COLCA1 cluster10
CTD-2647E9.3 cluster10
RP11-665J16.1 cluster10
RP11-470L19.6 cluster10
CA9 cluster10
SRRM5 cluster10
RP11-544A12.4 cluster10
AC016912.3 cluster10
RP5-1022J11.2 cluster10
RP11-385M4.1 cluster10
CTD-3035K23.7 cluster10
LINC01377 cluster10
PCCB cluster10
ZNF32-AS3 cluster10
THAP2 cluster10
RP11-467L13.7 cluster10
SLC25A41 cluster10

NDUFB2 cluster10
SH3BP5 cluster10
DCDC2C cluster10
LPGAT1 cluster10
RP11-313E19.2 cluster10
CD46 cluster10
RP11-557J10.5 cluster10
CTB-33O18.3 cluster10
SPDYE3 cluster10
ASGR1 cluster10
LRRC72 cluster10
CST8 cluster10
CTA-243E7.1 cluster10
WISP3 cluster10
PEX5 cluster10
RP11-87C7.3 cluster10
IRF1 cluster10
FAM170B cluster10
MIR4307HG cluster10
AC105339.1 cluster10
SIRT2 cluster10
GABBR2 cluster10
DPY19L1 cluster10
TUBB6 cluster10
DXO cluster10
LINC01467 cluster10
CRLS1 cluster10
AGAP4 cluster10
RP11-478K15.7 cluster10
LINC00358 cluster10
LINC01523 cluster10
RP11-499O7.7 cluster10
RP11-164O23.7 cluster10
AKNAD1 cluster10
AC116609.1 cluster10
PCSK2 cluster10
LINC01001 cluster10
RP11-839G9.1 cluster10
CLIP4 cluster10
TMEM150A cluster10
SCAND1 cluster10
ADGRG1 cluster10
LINGO1-AS1 cluster10
PALMD cluster10
CHD5 cluster10
AFAP1L1 cluster10
RP11-862G15.1 cluster10
TANGO6 cluster10
MOXD1 cluster10
RP11-644K8.1 cluster10
KRT15 cluster10
AP006216.5 cluster10
DNAJB8-AS1 cluster10
VGLL3 cluster10
LMO1 cluster10
CTB-91J4.1 cluster10

SPATA31D4 cluster10
ACSS1 cluster10
RNF125 cluster10
LNX1-AS1 cluster10
SMIM14 cluster10
RP3-335N17.2 cluster10
CEP76 cluster10
RP11-480I12.10 cluster10
MCF2L2 cluster10
CFAP20 cluster10
JRKL cluster10
SORCS3-AS1 cluster10
HECW1 cluster10
SNX13 cluster10
NPIPB4 cluster10
CAHM cluster10
WDR26 cluster10
RP11-14O19.1 cluster10
PJA1 cluster10
RP11-514F8.2 cluster10
TRBV7-1 cluster10
AP000345.1 cluster10
TLR3 cluster10
EFCAB14-AS1 cluster10
SNX31 cluster10
AC064834.3 cluster10
CKB cluster10
AC005808.3 cluster10
ARAP1 cluster10
GMCL1 cluster10
TRIM11 cluster10
SLC35A5 cluster10
FANCC cluster10
TROVE2 cluster10
RP11-666O2.2 cluster10
DYRK4 cluster10
KLHL11 cluster10
BAZ1A cluster10
NPIPB7 cluster10
ZNF790 cluster10
IQCE cluster10
DDA1 cluster10
AGBL1-AS1 cluster10
ZFP42 cluster10
RNF139-AS1 cluster10
SHOX2 cluster10
COQ6 cluster10
AFF4 cluster10
WDR37 cluster10
ZNF330 cluster10
LTN1 cluster10
RP11-334J6.6 cluster10
DKK3 cluster10
TCF20 cluster10
LAPTM4A cluster10
AC004862.6 cluster10

RP11-552D8.1 cluster10
RP11-62H20.1 cluster10
C2orf16 cluster10
CYP17A1 cluster10
PLPP6 cluster10
KB-1183D5.13 cluster10
MEX3B cluster10
PPP2R5E cluster10
RP11-478H13.1 cluster10
POPDC3 cluster10
S100A10 cluster10
CDADC1 cluster10
PACRG-AS3 cluster10
TOR1AIP1 cluster10
ARHGEF39 cluster10
MFSD12 cluster10
IQCJ-SCHIP1-AS1 cluster10
PARD3B cluster10
AGAP9 cluster10
ADRA1A cluster10
RP5-1030M6.3 cluster10
RP11-295M18.6 cluster10
FDPS cluster10
RP11-359G22.2 cluster10
AGAP5 cluster10
ASNS cluster10
ADAMTS17 cluster10
ZBTB1 cluster10
AC099342.1 cluster10
RP11-359G22.4 cluster10
DDAH2 cluster10
LINC01580 cluster10
FAM153A cluster10
RP11-114H24.3 cluster10
RP11-179A9.3 cluster10
MAMDC2 cluster10
WFDC11 cluster10
NLGN1 cluster10
RP11-779O18.3 cluster10
CLIP1-AS1 cluster10
ORMDL3 cluster10
C15orf59-AS1 cluster10
TGM7 cluster10
AC012456.3 cluster10
RP11-73O6.3 cluster10
MYO1D cluster10
LINC01490 cluster10
EIF1AX cluster10
RP11-166B2.1 cluster10
RP11-51J9.5 cluster10
CDK6 cluster10
RP11-307O10.1 cluster10
SLC36A1 cluster10
ST7 cluster10
RP11-696N14.1 cluster10
KXD1 cluster10

CTC-499B15.7 cluster10
FRMD3 cluster10
HK2 cluster10
RARA-AS1 cluster10
KIZ-AS1 cluster10
RP1-127D3.4 cluster10
RP11-20B24.4 cluster10
RBL2 cluster10
RP3-453P22.2 cluster10
EDRF1 cluster10
MFSD6 cluster10
LINC00442 cluster10
AC010907.5 cluster10
TBC1D3I cluster10
FAM134C cluster10
SCRT1 cluster10
ARHGAP42 cluster10
PPP1CB cluster10
AC069363.1 cluster10
RP5-1027O15.1 cluster10
PRICKLE1 cluster10
MRRF cluster10
SEMA3D cluster10
DLGAP2-AS1 cluster10
ITIH4 cluster10
PSMD3 cluster10
EMC10 cluster10
RP11-340I6.7 cluster10
RP11-108M9.3 cluster10
RP1-111D6.2 cluster10
LINC01082 cluster10
RP3-333B15.4 cluster10
LINC00906 cluster10
C22orf31 cluster10
FAM60A cluster10
GCC1 cluster10
CWC27 cluster10
CLEC2D cluster10
GORASP1 cluster10
EVX1-AS cluster10
WDR20 cluster10
GGH cluster10
ANKS1B cluster10
RP11-168P8.3 cluster10
CYB5D1 cluster10
CAP1 cluster10
CDC42SE1 cluster10
ZSCAN29 cluster10
CEP164 cluster10
PTPN11 cluster10
RP11-1017G21.4 cluster10
AGAP6 cluster10
RP11-56L13.1 cluster10
DSCR9 cluster10
PICK1 cluster10
MAK cluster10

VPS53 cluster10
TDP2 cluster10
STK3 cluster10
MFSD11 cluster10
PKNOX2 cluster10
RP11-141M1.1 cluster10
BCL2L11 cluster10
WBP2 cluster10
GPD2 cluster10
SPAG4 cluster10
WSCD1 cluster10
ST7-AS2 cluster10
HIPK1-AS1 cluster10
LRRC9 cluster10
SPDYE1 cluster10
WRN cluster10
SLC25A44 cluster10
UGT1A6 cluster10
CSRNP1 cluster10
PSME3 cluster10
IDI1 cluster10
FAHD1 cluster10
NF1 cluster10
CYP51A1-AS1 cluster10
COLCA2 cluster10
VEPH1 cluster10
HAS2-AS1 cluster10
CACNA2D3 cluster10
GFOD2 cluster10
VASH1 cluster10
LINC01374 cluster10
FAM109A cluster10
SLC16A1-AS1 cluster10
CYP51A1 cluster10
CCNT1 cluster10
DES cluster10
SULT1A1 cluster10
GSTCD cluster10
RP11-247C2.2 cluster10
MOCS3 cluster10
C22orf46 cluster10
HDAC4 cluster10
ACAT2 cluster10
RP11-587H10.2 cluster10
RP11-356J5.12 cluster10
PITRM1 cluster10
NMNAT2 cluster10
KLF11 cluster10
KPNA3 cluster10
AC073342.12 cluster10
RP11-396F22.1 cluster10
IFIH1 cluster10
DDX52 cluster10
AHCYL2 cluster10
DNAJB4 cluster10
RP11-522N14.2 cluster10

DGAT2 cluster10
C1orf43 cluster10
HEMK1 cluster10
EVX1 cluster10
TOPORS cluster10
MIMT1 cluster10
NSUN3 cluster10
IGF2BP2 cluster10
UFD1L cluster10
LINC01315 cluster10
FRMPD2 cluster10
FAM63B cluster10
AC011718.2 cluster10
AMFR cluster10
WASF3 cluster10
RP5-864K19.4 cluster10
MT2A cluster10
FUT11 cluster10
TUBG1 cluster10
DHX33 cluster10
BCL10 cluster10
FAM212B cluster10
ZCCHC6 cluster10
MRC2 cluster10
UBXN8 cluster10
SUCLA2 cluster10
RAD21L1 cluster10
ARF4-AS1 cluster10
PTRH1 cluster10
REXO1 cluster10
LRRC8B cluster10
CLIP1 cluster10
KCNV2 cluster10
GS1-124K5.3 cluster10
YWHAG cluster10
FOXP1 cluster10
RDH10 cluster10
UBE2D3 cluster10
RP11-383M4.6 cluster10
FAM200A cluster10
TXNDC17 cluster10
ZDHHC6 cluster10
AKR1E2 cluster10
AP1AR cluster10
EIF4G1 cluster10
RRP1B cluster10
RGMB cluster10
NAPEPLD cluster10
AMOTL1 cluster10
CYB561A3 cluster10
MIPOL1 cluster10
SESN1 cluster10
ALDH3A2 cluster10
RAD51D cluster10
TGFBR1 cluster10
MYH11 cluster10

MIEF1 cluster10
GFER cluster10
CENPJ cluster10
C10orf35 cluster10
KIAA1715 cluster10
CDH23 cluster10
HOOK3 cluster10
THAP6 cluster10
CCDC113 cluster10
CLEC16A cluster10
C1orf174 cluster10
ABLIM1 cluster10
RP11-665C16.6 cluster10
HSDL2 cluster10
PPP1R3E cluster10
LRRC36 cluster10
OTUD4 cluster10
CDC5L cluster10
KIZ cluster10
ZFP36L1 cluster10
OSER1-AS1 cluster10
KLHL20 cluster10
MYO15A cluster10
MAPK8 cluster10
CDIP1 cluster10
AP5M1 cluster10
EPS15L1 cluster10
KBTBD7 cluster10
NFX1 cluster10
TNKS cluster10
DHDDS cluster10
ATMIN cluster10
LMBR1L cluster10
NPLOC4 cluster10
MAFG cluster10
ATPAF1 cluster10
ECHDC2 cluster10
FAM76B cluster10
CAPZA1 cluster10
ZSCAN5A cluster10
ABHD2 cluster10
UBE2R2 cluster10
LINC00982 cluster10
ZNF44 cluster10
MKL1 cluster10
TOB1 cluster10
CSNK2A2 cluster10
FBXO34 cluster10
HYOU1 cluster10
USP4 cluster10
XPNPEP3 cluster10
PLBD1 cluster10
KIAA1586 cluster10
ZCCHC2 cluster10
ANKRD28 cluster10
RP11-242D8.1 cluster10

CTDSPL2 cluster10
KIF1C cluster10
MYO19 cluster10
SMYD2 cluster10
NPIPA1 cluster10
TRMT2A cluster10
CLK3 cluster10
GPATCH2L cluster10
BMI1 cluster10
SNHG9 cluster10
MRPL57 cluster10
ZNF451 cluster10
ERCC4 cluster10
C12orf73 cluster10
RAD21 cluster10
MCL1 cluster10
ROCK1 cluster10
SMAP1 cluster10
RPRD1A cluster10
CCDC28A cluster10
GIGYF2 cluster10
ZNF331 cluster10
EFCAB13 cluster10
FAM76A cluster10
RIMKLB cluster10
SLC16A1 cluster10
ACACA cluster10
DGCR8 cluster10
ZCCHC8 cluster10
ITFG2 cluster10
G3BP2 cluster10
SPIN1 cluster10
PTPN20 cluster10
MGEA5 cluster10
NAALAD2 cluster10
GALNT2 cluster10
TMEM63B cluster10
ATP1B3 cluster10
PBX1 cluster10
ANXA2 cluster10
COL4A3BP cluster10
KLF6 cluster10
AC007557.1 Cluster11
OTUB2 Cluster11
TEX37 Cluster11
ACTL9 Cluster11
BAG1 Cluster11
CAPZA3 Cluster11
FUZ Cluster11
FAM166A Cluster11
YWHAZ Cluster11
PFN3 Cluster11
AC006942.4 Cluster11
TEX36-AS1 Cluster11
OXCT2 Cluster11
SPANXD Cluster11

CTD-2015G9.1 Cluster11
CCDC54 Cluster11
SPATA3-AS1 Cluster11
IQCF5 Cluster11
SPANXC Cluster11
GTF3C2-AS1 Cluster11
SPANXB1 Cluster11
ARMC12 Cluster11
TNP2 Cluster11
FHL1 Cluster11
CXorf65 Cluster11
ZNF295-AS1 Cluster11
TNP1 Cluster11
CCDC179 Cluster11
MIR193BHG Cluster11
IQCF2 Cluster11
TEX35 Cluster11
IQCF1 Cluster11
FAM71E1 Cluster11
SPATA24 Cluster11
RP4-584D14.6 Cluster11
C10orf82 Cluster11
SLIT1-AS1 Cluster11
SPATA19 Cluster11
RP5-1051H14.2 Cluster11
SPERT Cluster11
UBE2J1 Cluster11
HMGB4 Cluster11
SPATA33 Cluster11
FAM229A Cluster11
ROPN1L Cluster11
NFKBIB Cluster11
HEMGN Cluster11
RP11-168L7.3 Cluster11
GNG2 Cluster11
C9orf9 Cluster11
SMIM6 Cluster11
SBDS Cluster11
TIMD4 Cluster11
RP11-286O18.1 Cluster11
TCP10L Cluster11
RAB21 Cluster11
IRGC Cluster11
FGF14-AS1 Cluster11
C2orf88 Cluster11
MRVI1-AS1 Cluster11
XXyac-YX155B6.5 Cluster11
RP11-619L12.4 Cluster11
ROPN1B Cluster11
C17orf105 Cluster11
TUBA4A Cluster11
FLJ27354 Cluster11
SCP2D1 Cluster11
TBX18-AS1 Cluster11
LINC01350 Cluster11
CALM3 Cluster11

RP11-597D13.8 Cluster11
CNN1 Cluster11
CEP112 Cluster11
FBL Cluster11
FAM81B Cluster11
EDRF1-AS1 Cluster11
GSTK1 Cluster11
GTSF1L Cluster11
RP11-768G7.2 Cluster11
IFT172 Cluster11
ACP1 Cluster11
CYLC2 Cluster11
KB-1083B1.1 Cluster11
FBXO39 Cluster11
DNAJA4 Cluster11
FHL5 Cluster11
MIR7515HG Cluster11
PRKAR2A Cluster11
DNALI1 Cluster11
MSMP Cluster11
EIF5A2 Cluster11
RP11-251P6.1 Cluster11
CEP295NL Cluster11
TFAM Cluster11
ATAD1 Cluster11
LINC00226 Cluster11
IFT57 Cluster11
APOPT1 Cluster11
POLR2I Cluster11
S100A11 Cluster11
COX8C Cluster11
RP5-971N18.3 Cluster11
SPATA32 Cluster11
MS4A5 Cluster11
FAM71F1 Cluster11
RNF151 Cluster11
RP11-197N18.7 Cluster11
SPA17 Cluster11
FAM205C Cluster11
C1orf100 Cluster11
ZNF345 Cluster11
KIF2B Cluster11
ARHGAP26-AS1 Cluster11
HOOK1 Cluster11
DYNLL2 Cluster11
SPATA7 Cluster11
PRRX1 Cluster11
RP11-758P17.3 Cluster11
TSKS Cluster11
GID4 Cluster11
ZNF91 Cluster11
FANCD2OS Cluster11
GAS8 Cluster11
ARPC5L Cluster11
IPO5 Cluster11
FAM104A Cluster11

RP11-958J22.3 Cluster11
EFCAB1 Cluster11
RP11-215E13.1 Cluster11
AKIRIN1 Cluster11
RP11-80B9.4 Cluster11
CBY3 Cluster11
MEA1 Cluster11
LINC01333 Cluster11
SOCS7 Cluster11
TSC22D1 Cluster11
RP11-38C18.3 Cluster11
LKAAEAR1 Cluster11
WDR1 Cluster11
AC007163.6 Cluster11
SIRPD Cluster11
GSTO2 Cluster11
RP11-232C2.2 Cluster11
GOSR2 Cluster11
BAG5 Cluster11
CCSER1 Cluster11
ZNF683 Cluster11
RP11-152O14.4 Cluster11
RNF133 Cluster11
TIAM2 Cluster11
LINC01255 Cluster11
EWSAT1 Cluster11
POLR3C Cluster11
SLC25A37 Cluster11
VPS45 Cluster11
EEF2KMT Cluster11
RP13-436F16.1 Cluster11
RP11-369E15.4 Cluster11
CLK4 Cluster11
YPEL1 Cluster11
TTLL2 Cluster11
ZBTB20-AS1 Cluster11
KIF5C Cluster11
RP11-102C16.3 Cluster11
C3orf30 Cluster11
NAA38 Cluster11
RFPL3S Cluster11
RP11-180C1.1 Cluster11
GPRC5B Cluster11
INPP1 Cluster11
RP11-420J11.2 Cluster11
GOLM1 Cluster11
GRID2 Cluster11
RP11-12M5.1 Cluster11
VPS13A-AS1 Cluster11
RP11-834C11.5 Cluster11
ATP6V1A Cluster11
CELA3B Cluster11
LINC00927 Cluster11
RP13-401N8.1 Cluster11
RP5-1002M8.4 Cluster11
AC079779.5 Cluster11

OVOL1-AS1 Cluster11
BPI Cluster11
CCDC159 Cluster11
C8orf74 Cluster11
TXNDC8 Cluster11
MAPT Cluster11
LPAR6 Cluster11
BCAT1 Cluster11
CDV3 Cluster11
ANKEF1 Cluster11
SPANXN4 Cluster11
FABP12 Cluster11
RASSF8 Cluster11
RP11-165M1.3 Cluster11
FAM71C Cluster11
RP11-27M9.1 Cluster11
CTD-2118P12.1 Cluster11
LINC01548 Cluster11
CATSPERD Cluster11
RP11-664D1.1 Cluster11
RP11-374M1.3 Cluster11
C1QTNF3 Cluster11
PPP1R7 Cluster11
AC007131.2 Cluster11
CAPN2 Cluster11
LINC01430 Cluster11
TLE4 Cluster11
SSX2IP Cluster11
CEP350 Cluster11
RP11-34D15.2 Cluster11
RP11-158I23.1 Cluster11
PRRX2-AS1 Cluster11
ISCA1 Cluster11
ASTN2 Cluster11
FAM71D Cluster11
RP11-369E15.3 Cluster11
FOXP1-AS1 Cluster11
RP11-456H18.1 Cluster11
RP11-21B23.3 Cluster11
LINC01233 Cluster11
RP11-17A4.3 Cluster11
EHBP1 Cluster11
TTC39A Cluster11
RP3-331H24.5 Cluster11
PCAT6 Cluster11
TEPP Cluster11
FAM182B Cluster11
RNF139 Cluster11
LINC01277 Cluster11
RP11-399E6.1 Cluster11
ABCC3 Cluster11
CTD-2666L21.1 Cluster11
LINC00929 Cluster11
CDC14A Cluster11
CFAP44-AS1 Cluster11
S100A8 Cluster11

LINC01539 Cluster11
KNSTRN Cluster11
PDE1A Cluster11
C12orf42 Cluster11
SNAP23 Cluster11
RP1-140J1.1 Cluster11
C17orf112 Cluster11
GSKIP Cluster11
PRKCE Cluster11
PLAC8L1 Cluster11
AC016738.3 Cluster11
C20orf85 Cluster11
RP11-319E16.1 Cluster11
ARL2BP Cluster11
RP11-734K2.4 Cluster11
ARHGAP28 Cluster11
MKRN2 Cluster11
CELA3A Cluster11
NLK Cluster11
AC008694.3 Cluster11
AC002465.2 Cluster11
LINC00944 Cluster11
LINC00919 Cluster11
SERPINB6 Cluster11
C18orf32 Cluster11
PAGE3 Cluster11
DNPEP Cluster11
MTFR1 Cluster11
RP3-399L15.3 Cluster11
RP11-547D23.1 Cluster11
CCDC180 Cluster11
TRIM42 Cluster11
RP11-411H5.1 Cluster11
PKIB Cluster11
RAB3IL1 Cluster11
PLCE1-AS2 Cluster11
MAPRE3 Cluster11
RP11-778H2.1 Cluster11
RP1-17K7.3 Cluster11
LINC00293 Cluster11
GPR182 Cluster11
AGPAT2 Cluster11
LINC00935 Cluster11
CTD-2143L24.1 Cluster11
CES1 Cluster11
HERPUD2 Cluster11
ARAP2 Cluster11
FREM1 Cluster11
CTNNA3 Cluster11
SCLY Cluster11
RGS6 Cluster11
SERGEF Cluster11
RP11-17A4.2 Cluster11
ELL Cluster11
AGPAT3 Cluster11
MICAL2 Cluster11

CSNK1G2-AS1 Cluster11
FAM183A Cluster11
LSM12 Cluster11
CTD-2071N1.1 Cluster11
RP11-281P23.2 Cluster11
C6orf89 Cluster11
RP11-347D21.1 Cluster11
ZNF75D Cluster11
SFXN5 Cluster11
KIFC3 Cluster11
RP5-845O24.8 Cluster11
NTMT1 Cluster11
ROR1-AS1 Cluster11
CPEB3 Cluster11
C6orf163 Cluster11
ENOX1 Cluster11
RP11-24D15.1 Cluster11
RP11-201M22.1 Cluster11
RP11-85L21.4 Cluster11
LINC01364 Cluster11
FAM181A-AS1 Cluster11
CTC-360P9.4 Cluster11
DCAF6 Cluster11
VARS Cluster11
RP11-292B1.2 Cluster11
LARP1B Cluster11
PPM1J Cluster11
PDXDC1 Cluster11
LINC00162 Cluster11
RP11-421P23.1 Cluster11
FAM71A Cluster11
FAM71B Cluster11
PRSS58 Cluster11
PRSS37 Cluster11
C20orf144 Cluster11
CCDC80 Cluster11
IQCF6 Cluster11
CMTM2 Cluster11
RP11-140K17.3 Cluster11
DKKL1 Cluster11
MEX3C Cluster11
FBXO24 Cluster11
TMEM191B Cluster11
H2AFJ Cluster11
TBC1D26 Cluster11
NDNF Cluster11
FAM134B Cluster11
WDR64 Cluster11
MAPK3 Cluster11
ACSL1 Cluster11
RORA Cluster11
RP11-526P6.1 Cluster11
RP11-519M16.1 Cluster11
RP11-367J7.3 Cluster11
RP11-76E17.3 Cluster11
LINC00448 Cluster11

AC113607.2 Cluster11
RP11-483P21.6 Cluster11
RP11-404L6.2 Cluster11
DRICH1 Cluster11
DCC Cluster11
CTD-2020K17.4 Cluster11
RP11-415D17.3 Cluster11
RP11-543H12.1 Cluster11
TMEM205 Cluster11
LINC00494 Cluster11
RP11-190D6.2 Cluster11
RP11-73E17.2 Cluster11
RP11-6F2.5 Cluster11
GPR18 Cluster11
RP1-27K12.4 Cluster11
LINC00616 Cluster11
ZNRD1-AS1 Cluster11
RP11-152O14.1 Cluster11
RP4-737E23.2 Cluster11
HPCA Cluster11
RP11-112L6.3 Cluster11
AC009158.1 Cluster11
CTB-174D11.1 Cluster11
SLC37A1 Cluster11
SPATA45 Cluster11
RP11-188C12.3 Cluster11
ITPR3 Cluster11
AGT Cluster11
KIAA1211 Cluster11
RP11-161D15.1 Cluster11
MIR3976HG Cluster11
CDKN1C Cluster11
RP11-846C15.2 Cluster11
LINC01214 Cluster11
SRD5A2 Cluster11
SUFU Cluster11
BMP8B Cluster11
TP53INP2 Cluster11
MIR762HG Cluster11
PIFO Cluster11
SLC1A2 Cluster11
EAF1 Cluster11
OSBPL10-AS1 Cluster11
ADGRL3-AS1 Cluster11
C10orf90 Cluster11
THAP5 Cluster11
CTD-2144E22.8 Cluster11
ABHD16B Cluster11
INPP4B Cluster11
MATN2 Cluster11
RP11-101E7.2 Cluster11
DPY19L2 Cluster11
RAI14 Cluster11
GRB10 Cluster11
EPB41L3 Cluster11
IGSF11 Cluster11

EFCAB6 Cluster11
RP11-379H18.1 Cluster11
RP11-63A1.2 Cluster11
RP11-774D14.1 Cluster11
VSTM4 Cluster11
RP11-883G14.2 Cluster11
DNAAF3 Cluster11
APLF Cluster11
LDLRAD4 Cluster11
AL157902.3 Cluster11
FER Cluster11
FAM53C Cluster11
GNAI2 Cluster11
SUN1 Cluster11
SGF29 Cluster11
ZNF487 Cluster11
SYAP1 Cluster11
SPIRE1 Cluster11
NDUFV2-AS1 Cluster11
PRKAA1 Cluster11
C12orf10 Cluster11
RP11-755O11.2 Cluster11
SURF2 Cluster11
SPATA3 Cluster12
DCUN1D1 Cluster12
LELP1 Cluster12
C10orf62 Cluster12
TSSK6 Cluster12
OAZ3 Cluster12
GLUL Cluster12
ACAP1 Cluster12
GSG1 Cluster12
TSPAN16 Cluster12
C16orf82 Cluster12
FSCN3 Cluster12
TPPP2 Cluster12
DNAJC4 Cluster12
SPEM1 Cluster12
CRISP2 Cluster12
SH3RF2 Cluster12
NUPR2 Cluster12
C17orf74 Cluster12
C3orf22 Cluster12
REEP6 Cluster12
UBL4B Cluster12
MORN3 Cluster12
CCNY Cluster12
SMCP Cluster12
RP11-322N21.2 Cluster12
BPIFA3 Cluster12
CLPB Cluster12
NDUFA13 Cluster12
SPATA42 Cluster12
PHOSPHO1 Cluster12
CTD-2568A17.1 Cluster12
RANGAP1 Cluster12

PROCA1 Cluster12
CCDC91 Cluster12
PCP2 Cluster12
CXCL16 Cluster12
GAPDHS Cluster12
PTP4A1 Cluster12
C19orf70 Cluster12
GPX4 Cluster12
LEMD1 Cluster12
PAQR7 Cluster12
SPATA18 Cluster12
LINC00608 Cluster12
DGCR6L Cluster12
LPIN1 Cluster12
RNF138 Cluster12
CARHSP1 Cluster12
CCDC37-AS1 Cluster12
SPTY2D1-AS1 Cluster12
TCP11 Cluster12
ODF3L2 Cluster12
ISG20L2 Cluster12
SMKR1 Cluster12
ODF2 Cluster12
C2orf57 Cluster12
PCYT2 Cluster12
LRTOMT Cluster12
C5orf52 Cluster12
CDIPT-AS1 Cluster12
DNAJB8 Cluster12
METAP1 Cluster12
C9orf173 Cluster12
STARD10 Cluster12
MOSPD3 Cluster12
MFAP3L Cluster12
PHKG2 Cluster12
ZFAND3 Cluster12
MYL6B Cluster12
GABRG3-AS1 Cluster12
HIP1 Cluster12
TEX38 Cluster12
PSMF1 Cluster12
TMCO5A Cluster12
AKAP1 Cluster12
BZW1 Cluster12
BOD1L2 Cluster12
PKM Cluster12
NDUFA3 Cluster12
C12orf54 Cluster12
RP11-402G3.5 Cluster12
ERI3 Cluster12
CCDC57 Cluster12
ACTL7A Cluster12
CAPZB Cluster12
C16orf78 Cluster12
RP11-496I9.1 Cluster12
KIF2C Cluster12

TMEM31 Cluster12
RP11-73G16.3 Cluster12
UBXN6 Cluster12
ABHD1 Cluster12
AK1 Cluster12
RP11-195F19.5 Cluster12
TSPAN1 Cluster12
IQCF3 Cluster12
MRPS25 Cluster12
ALKBH7 Cluster12
TMEM53 Cluster12
ACSBG2 Cluster12
FAM46C Cluster12
CABS1 Cluster12
CEP170 Cluster12
CIB1 Cluster12
AQP5 Cluster12
C1orf234 Cluster12
FAM71F2 Cluster12
EIF4H Cluster12
ALDOA Cluster12
PDXK Cluster12
LASP1 Cluster12
C11orf71 Cluster12
C20orf141 Cluster12
MAP1LC3A Cluster12
RCC1 Cluster12
AKAP4 Cluster12
RP11-217B7.3 Cluster12
FUNDC2 Cluster12
LINC01191 Cluster12
THEG Cluster12
TSSK3 Cluster12
FBXW5 Cluster12
NBR1 Cluster12
ZNF649-AS1 Cluster12
NDUFA9 Cluster12
LINC00347 Cluster12
ACTRT2 Cluster12
VRK3 Cluster12
STX8 Cluster12
RAB11FIP4 Cluster12
ETNK2 Cluster12
AZIN2 Cluster12
TSPAN6 Cluster12
CCSER2 Cluster12
PRM3 Cluster12
ADIG Cluster12
SLFNL1 Cluster12
ARPP19 Cluster12
DUSP15 Cluster12
SHARPIN Cluster12
SLC39A13 Cluster12
MS4A14 Cluster12
PRKCZ Cluster12
PRH1 Cluster12

C9orf16 Cluster12
RP11-304L19.4 Cluster12
TRIM36 Cluster12
CCDC70 Cluster12
AC012594.1 Cluster12
PGAM2 Cluster12
OSER1 Cluster12
PSORS1C1 Cluster12
C11orf42 Cluster12
TMEM120A Cluster12
RP5-956O18.2 Cluster12
FAM187B Cluster12
UBQLN3 Cluster12
LPP Cluster12
PAFAH1B1 Cluster12
SEC14L1 Cluster12
FNDC8 Cluster12
RP11-109E10.1 Cluster12
HAGLR Cluster12
ARL4A Cluster12
AC009487.4 Cluster12
TMEM210 Cluster12
ARHGAP35 Cluster12
STK35 Cluster12
C9orf24 Cluster12
GUCA1A Cluster12
LINC00851 Cluster12
AC010649.1 Cluster12
SPATA6 Cluster12
KCNIP2-AS1 Cluster12
RNF38 Cluster12
PGK2 Cluster12
SGCA Cluster12
NRDC Cluster12
RP11-146F11.1 Cluster12
RAD23B Cluster12
DPP7 Cluster12
AKAP14 Cluster12
NTRK3-AS1 Cluster12
MACROD2-AS1 Cluster12
NSUN4 Cluster12
GSTM3 Cluster12
RND2 Cluster12
HBZ Cluster12
CABYR Cluster12
ST6GALNAC2 Cluster12
PLBD2 Cluster12
MAP2K2 Cluster12
PHACTR2-AS1 Cluster12
C10orf53 Cluster12
SLC38A7 Cluster12
HABP4 Cluster12
ODF3B Cluster12
WBP2NL Cluster12
AC002115.5 Cluster12
UBQLNL Cluster12

LRRD1 Cluster12
BRWD1-AS2 Cluster12
YJEFN3 Cluster12
MAD2L2 Cluster12
OPTN Cluster12
AC007325.4 Cluster12
MSTO1 Cluster12
TSSK2 Cluster12
FAM229B Cluster12
TREH Cluster12
ELOF1 Cluster12
DYRK1B Cluster12
CUL9 Cluster12
ZDHHC19 Cluster12
RGS22 Cluster12
LINC00521 Cluster12
LSMEM2 Cluster12
ZNF571-AS1 Cluster12
CCDC136 Cluster12
TP53I11 Cluster12
TTLL10 Cluster12
SPATC1 Cluster12
SORBS3 Cluster12
PSMA6 Cluster12
OGFOD2 Cluster12
SH3GL3 Cluster12
KLHDC3 Cluster12
PGP Cluster12
PPP1R11 Cluster12
PHF2 Cluster12
ATXN1L Cluster12
RABIF Cluster12
TSACC Cluster12
EIF4E Cluster12
NDUFS8 Cluster12
WI2-2373I1.2 Cluster12
NDUFV3 Cluster12
NUP35 Cluster12
FAM120B Cluster12
RP11-400L8.2 Cluster12
ACE Cluster12
RNF44 Cluster12
SPATA12 Cluster12
CCDC187 Cluster12
BCL2L14 Cluster12
NELFE Cluster12
PRPH Cluster12
SMG9 Cluster12
RP11-162N7.1 Cluster12
PARD6A Cluster12
PIK3R3.1 Cluster12
LINC00535 Cluster12
HK1 Cluster12
ACPT Cluster12
TPI1 Cluster12
BCAS3 Cluster12

PHF7 Cluster12
DNAJC5B Cluster12
CT83 Cluster12
METTL22 Cluster12
MROH7 Cluster12
DGCR14 Cluster12
RANGRF Cluster12
MEX3D Cluster12
INPP5K Cluster12
SLC27A5 Cluster12
SPATA20 Cluster12
GPI Cluster12
NEURL1 Cluster12
ZNF688 Cluster12
CTB-35F21.2 Cluster12
ANKRD53 Cluster12
RP11-1277A3.1 Cluster12
CLCNKA Cluster12
RP11-364B14.1 Cluster12
PAOX Cluster12
LINC00445 Cluster12
TNFAIP8L1 Cluster12
RP11-520D19.1 Cluster12
C16orf13 Cluster12
CDHR2 Cluster12
TSSK1B Cluster12
PPP2R5A Cluster12
GNAS Cluster12
H1FNT Cluster12
BANF2 Cluster12
IGSF11-AS1 Cluster12
OSGIN1 Cluster12
SAMD4A Cluster12
SLC22A18 Cluster12
ZMIZ1 Cluster12
FAM195A Cluster12
C1orf194 Cluster12
ASB5 Cluster12
ETV2 Cluster12
ZNF124 Cluster12
RP11-345J4.3 Cluster12
MAP3K14-AS1 Cluster12
CRAT Cluster12
DUSP18 Cluster12
SENP8 Cluster12
GSTZ1 Cluster12
DKFZP434K028 Cluster12
CARS2 Cluster12
ZNF706 Cluster12
DNAJC18 Cluster12
PWRN1 Cluster12
AC004070.1 Cluster12
TESC Cluster12
PDE4A Cluster12
MAPT-AS1 Cluster12
RP11-347C12.3 Cluster12

ANO2 Cluster12
AC092155.4 Cluster12
LYPD4 Cluster12
RP11-30L15.6 Cluster12
LINC00901 Cluster12
GPX1 Cluster12
DDX3X Cluster12
RP11-126O1.2 Cluster12
HYPM Cluster12
COL9A3 Cluster12
DUSP21 Cluster12
UBA5 Cluster12
FLJ36000 Cluster12
SNX29 Cluster12
10-Mar Cluster12
C16orf72 Cluster12
ZDHHC3 Cluster12
DHX57 Cluster12
AVPI1 Cluster12
TTLL6 Cluster12
BMP7-AS1 Cluster12
KLK13 Cluster12
SLC25A28 Cluster12
CYSTM1 Cluster12
CYP17A1-AS1 Cluster12
CCDC169 Cluster12
USPL1 Cluster12
TBX1 Cluster12
NRAV Cluster12
PDZD8 Cluster12
GLRX2 Cluster12
RP11-100N20.1 Cluster12
FRMD6 Cluster12
TEX26 Cluster12
SYN3 Cluster12
RP11-763K15.1 Cluster12
C17orf50 Cluster12
QARS Cluster12
THBS3 Cluster12
CIB2 Cluster12
MRPS15 Cluster12
STPG1 Cluster12
AZIN1-AS1 Cluster12
CSNK1D Cluster12
SLC22A23 Cluster12
PCSK4 Cluster12
VWA3B Cluster12
RP11-14J7.7 Cluster12
LYPD6 Cluster12
FNDC4 Cluster12
C17orf97 Cluster12
RP4-712E4.1 Cluster12
SPZ1 Cluster12
CYB5R2 Cluster12
MAD1L1 Cluster12
DDI1 Cluster12

TBATA Cluster12
EPHA10 Cluster12
RP11-536N17.1 Cluster12
CTD-3074O7.5 Cluster12
RP11-191N8.2 Cluster12
RP11-932O9.4 Cluster12
BACE1 Cluster12
EGLN2 Cluster12
C9orf50 Cluster12
RFTN2 Cluster12
HYAL3 Cluster12
AC004623.2 Cluster12
PPP2R2B Cluster12
SERPINA5 Cluster12
SPATC1L Cluster12
RP11-46O21.2 Cluster12
RP11-783K16.5 Cluster12
RP11-156L14.1 Cluster12
SMPD1 Cluster12
CBLN1 Cluster12
FSCB Cluster12
RP11-319G6.1 Cluster12
HRASLS5 Cluster12
TOLLIP Cluster12
RP11-168O16.2 Cluster12
ITPKA Cluster12
CCDC188 Cluster12
SAG Cluster12
EHD1 Cluster12
CDNF Cluster12
CFAP100 Cluster12
NAT6 Cluster12
CSMD2-AS1 Cluster12
LINC00635 Cluster12
LINC00943 Cluster12
NT5C1B Cluster12
ZNF350-AS1 Cluster12
C17orf107 Cluster12
TMEM91 Cluster12
MGC39584 Cluster12
CTD-2330K9.3 Cluster12
AC007557.4 Cluster12
CTD-2530N21.4 Cluster12
LSM14B Cluster12
RP11-834C11.4 Cluster12
RP11-286M16.1 Cluster12
LCP1 Cluster12
GNG3 Cluster12
RP11-770E5.3 Cluster12
TBC1D21 Cluster12
HDAC11 Cluster12
RP11-109P11.1 Cluster12
TH2LCRR Cluster12
B4GALT1-AS1 Cluster12
GDPD3 Cluster12
CSNK1G2 Cluster12

RP11-805I24.1 Cluster12
BRI3BP Cluster12
TMEM215 Cluster12
RP11-630C16.1 Cluster12
RP11-106M3.3 Cluster12
RP11-90C4.1 Cluster12
C19orf45 Cluster12
RP1-93H18.1 Cluster12
KIAA0513 Cluster12
RP11-1338A24.1 Cluster12
RP11-493E12.1 Cluster12
ODF1 Cluster12
USP2 Cluster12
C6orf132 Cluster12
TSGA13 Cluster12
GABRG3 Cluster12
SRPK2 Cluster12
MIR9-3HG Cluster12
OSBPL10 Cluster12
EXOSC4 Cluster12
TMEM239 Cluster12
RP11-214D15.2 Cluster12
CERS1 Cluster12
ZNHIT2 Cluster12
SLC25A46 Cluster12
BMP8A Cluster12
RP11-588G21.2 Cluster12
HSPA4L Cluster12
RIBC1 Cluster12
C4orf36 Cluster12
RP11-219E7.2 Cluster12
ZCCHC13 Cluster12
RP1-4G17.2 Cluster12
AC012370.3 Cluster12
PPP1R36 Cluster12
RP11-363E6.3 Cluster12
RP11-61J19.2 Cluster12
FBXL13 Cluster12
TAF10 Cluster12
TIMP2 Cluster12
OSBP2 Cluster12
TTC16 Cluster12
CTD-2520I13.1 Cluster12
HSPB9 Cluster12
ESPN Cluster12
RP11-402G3.3 Cluster12
RP11-881M11.1 Cluster12
SELO Cluster12
RP11-1078H9.5 Cluster12
TRIM29 Cluster12
SSR4 Cluster12
MYL6 Cluster12
PRC1-AS1 Cluster12
DGCR6 Cluster12
KLK5 Cluster12
CSE1L-AS1 Cluster12

RP11-415J8.5 Cluster12
C11orf1 Cluster12
RP11-370P15.2 Cluster12
RP1-92O14.6 Cluster12
NACC2 Cluster12
TMX4 Cluster12
CTD-2231E14.4 Cluster12
KIAA0895L Cluster12
RP11-624L4.2 Cluster12
PRR30 Cluster12
CHRNE Cluster12
FAM20A Cluster12
MOGS Cluster12
MTX1 Cluster12
PMFBP1 Cluster12
MAPK13 Cluster12
DHRS3 Cluster12
SELV Cluster12
LINC00326 Cluster12
C16orf90 Cluster12
SLC10A6 Cluster12
LPIN3 Cluster12
LRRC27 Cluster12
TPD52L3 Cluster12
PRR7-AS1 Cluster12
APOE Cluster12
FAM217A Cluster12
CCDC7 Cluster12
MFF Cluster12
LINGO1-AS2 Cluster12
C7orf31 Cluster12
KLK11 Cluster12
LYL1 Cluster12
CEP85L Cluster12
C12orf50 Cluster12
C2orf70 Cluster12
RP11-147C23.1 Cluster12
CLU Cluster12
LRRC71 Cluster12
CYLC1 Cluster12
EEF1D Cluster12
SPPL2C Cluster12
HSPA1L Cluster12
MAPK10 Cluster12
CD37 Cluster12
AMZ2 Cluster12
LINC01227 Cluster12
ZFAND4 Cluster12
KLHL10 Cluster12
LINC00202-1 Cluster12
RP11-705O24.1 Cluster12
RP11-7M8.2 Cluster12
PPP1R32 Cluster12
CDKN2A Cluster12
LOXHD1 Cluster12
CAMLG Cluster12

C6orf201 Cluster12
GALNTL5 Cluster12
TEX40 Cluster12
LINC00301 Cluster12
MORN2 Cluster12
BRK1 Cluster12
GHITM Cluster12
ARL2 Cluster12
ATL3 Cluster12
CNTFR-AS1 Cluster12
HMOX2 Cluster12
AC006946.15 Cluster12
MCM8-AS1 Cluster12
UQCC2 Cluster12
GFRA2 Cluster12
TCTE1 Cluster12
TMEM191C Cluster12
ICA1 Cluster12
IZUMO4 Cluster12
GGN Cluster12
S1PR2 Cluster12
NGLY1 Cluster12
LINC00658 Cluster12
FXR1 Cluster12
FAM220A Cluster12
TEX22 Cluster12
LURAP1 Cluster12
RP11-496H1.2 Cluster12
MRPL41 Cluster12
RAB8B Cluster12
RP11-38F22.1 Cluster12
MZT2B Cluster12
PTOV1 Cluster12
ADRM1 Cluster12
MPLKIP cluster2
CST3 cluster2
RPLP1 cluster2
HIST1H1A cluster2
MAGEA4 cluster2
PFN1 cluster2
RBM3 cluster2
LSM7 cluster2
RAMP2 cluster2
PNMA5 cluster2
PRDX1 cluster2
SLC35B2 cluster2
C1QBP cluster2
RDX cluster2
TNIP2 cluster2
EGFL7 cluster2
ZNF559 cluster2
C14orf2 cluster2
RASGRP2 cluster2
TSR2 cluster2
BSG cluster2
PARP1 cluster2

GTF2E2 cluster2
MPP5 cluster2
RBFA cluster2
CTSA cluster2
HSD17B10 cluster2
CT45A10 cluster2
GAGE1 cluster2
MTHFD2 cluster2
STARD7 cluster2
YY2 cluster2
CCDC167 cluster2
UXT cluster2
CTSD cluster2
PRAME cluster2
CETN2 cluster2
ATP5G2 cluster2
PIM1 cluster2
NAMPT cluster2
NAA50 cluster2
TSFM cluster2
OS9 cluster2
NOP56 cluster2
H2AFY cluster2
TMEM14B cluster2
BRD9 cluster2
PDLIM5 cluster2
DTYMK cluster2
STEAP1 cluster2
MCM6 cluster2
UBE2E3 cluster2
NAPG cluster2
LAMC1 cluster2
ADSL cluster2
IQGAP1 cluster2
CAMSAP1 cluster2
PIK3IP1 cluster2
GLMN cluster2
HTATSF1 cluster2
SFSWAP cluster2
NPAT cluster2
FAM96A cluster2
RNF2 cluster2
CENPM cluster2
HECTD1 cluster2
WHAMM cluster2
MFAP1 cluster2
GUCD1 cluster2
EIF4EBP2 cluster2
ERVK3-1 cluster2
C19orf43 cluster2
GRB2 cluster2
POP5 cluster2
TRIAP1 cluster2
AGA cluster2
HIST1H1C cluster2
PCNT cluster2

COPS7B cluster2
NPC1 cluster2
VPS25 cluster2
CHD3 cluster2
MCM3 cluster2
HDHD2 cluster2
FNBP1 cluster2
GANAB cluster2
POLE4 cluster2
MAPKAPK5 cluster2
TPM1 cluster2
MPHOSPH6 cluster2
BAX cluster2
SLC25A14 cluster2
NUP85 cluster2
KMT5B cluster2
CDK10 cluster2
TFDP3 cluster2
ERAL1 cluster2
EIF2AK1 cluster2
FXYD1 cluster2
SLC3A2 cluster2
RBM22 cluster2
PLD3 cluster2
CAPZA2 cluster2
ATG5 cluster2
C8orf33 cluster2
PARL cluster2
PPAT cluster2
TULP3 cluster2
PPIB cluster2
CHRNA5 cluster2
OGG1 cluster2
PAFAH1B2 cluster2
TWISTNB cluster2
NPM3 cluster2
NIPA2 cluster2
TUBGCP3 cluster2
MOCS2 cluster2
LRRC47 cluster2
AKAP10 cluster2
MECP2 cluster2
TMEM145 cluster2
UROD cluster2
CNTFR cluster2
CSTB cluster2
PRPF19 cluster2
NDUFB7 cluster2
FOXR1 cluster2
MRPS6 cluster2
NOC2L cluster2
CTU2 cluster2
ATRX cluster2
PGAM1 cluster2
KIAA0355 cluster2
RSBN1L cluster2

FDX1 cluster2
SMUG1 cluster2
MOB2 cluster2
PSMD13 cluster2
ANKRD17 cluster2
WDHD1 cluster2
JTB cluster2
TRAF2 cluster2
RAP1B cluster2
NUDT3 cluster2
ABHD14A cluster2
GGA2 cluster2
ARHGAP21 cluster2
SNRNP25 cluster2
RBM4B cluster2
PCYOX1 cluster2
TMX3 cluster2
CDCA7 cluster2
SESTD1 cluster2
CENPA cluster2
RBMX cluster2
CHCHD10 cluster2
SARNP cluster2
UBAP2 cluster2
NELFCD cluster2
GJC1 cluster2
NKIRAS2 cluster2
KANSL1-AS1 cluster2
HAUS8 cluster2
NXT2 cluster2
ZBTB11 cluster2
GNPTG cluster2
REXO4 cluster2
CPVL cluster2
COQ4 cluster2
ARHGAP12 cluster2
TMEM126B cluster2
MEF2A cluster2
TCOF1 cluster2
C9orf142 cluster2
RNASEH2C cluster2
LRRC40 cluster2
SPOP cluster2
FUBP1 cluster2
CTAG2 cluster2
POLR2A cluster2
CHCHD4 cluster2
HEATR1 cluster2
MRPS23 cluster2
CUL5 cluster2
MRPL44 cluster2
CRK cluster2
VMA21 cluster2
TSEN34 cluster2
MTRNR2L12 cluster2
COX5B cluster2

C9orf72 cluster2
MTMR12 cluster2
PPP2CB cluster2
PDPK1 cluster2
SPPL3 cluster2
TMEM245 cluster2
MRPL50 cluster2
RNF4 cluster2
TYW3 cluster2
LDHB cluster2
CD63 cluster2
RPL36A cluster2
MDM2 cluster2
HSPA1A cluster2
EED cluster2
RERE cluster2
RPS3 cluster2
PURA cluster2
PRKAG1 cluster2
MRPL48 cluster2
SNHG7 cluster2
EP300 cluster2
BORCS8 cluster2
CENPC cluster2
ANXA7 cluster2
PI4KB cluster2
TMEM199 cluster2
MBD4 cluster2
SGPL1 cluster2
PRDX5 cluster2
APLP1 cluster2
LIMS1 cluster2
PSMD12 cluster2
MAGOHB cluster2
COA3 cluster2
ZMYND19 cluster2
IFT46 cluster2
FAM136A cluster2
ANAPC13 cluster2
THUMPD1 cluster2
NOLC1 cluster2
E2F1 cluster2
CPSF2 cluster2
B2M cluster2
C7orf50 cluster2
UBA3 cluster2
SDF2 cluster2
WDR12 cluster2
RRAS2 cluster2
LCOR cluster2
HTT cluster2
LSG1 cluster2
HCFC1 cluster2
URM1 cluster2
CD2BP2 cluster2
CCZ1 cluster2

LTBP3 cluster2
TAF3 cluster2
SLF2 cluster2
EIF3I cluster2
RP9 cluster2
ACAP2 cluster2
SPG21 cluster2
MAP4K4 cluster2
ZNF711 cluster2
LRP6 cluster2
UBE2E2 cluster2
PFDN1 cluster2
DCPS cluster2
USP34 cluster2
TRNAU1AP cluster2
PACSIN2 cluster2
NR6A1 cluster2
PPP2R4 cluster2
FUNDC1 cluster2
CALU cluster2
APP cluster2
SMARCC1 cluster2
MPDU1 cluster2
SEC13 cluster2
JAM3 cluster2
CCAR2 cluster2
MORC4 cluster2
C16orf91 cluster2
PET117 cluster2
AAK1 cluster2
SSR1 cluster2
PASD1 cluster2
POLD1 cluster2
NAT10 cluster2
PPP3CA cluster2
LINC00969 cluster2
TCTN3 cluster2
ZFX cluster2
PHF10 cluster2
PPIL1 cluster2
PSMB4 cluster2
RNF13 cluster2
CDS2 cluster2
ABT1 cluster2
PMF1 cluster2
MAN1B1 cluster2
UMPS cluster2
PEX19 cluster2
ANKS3 cluster2
PPP1R8 cluster2
MRPL27 cluster2
SMARCAD1 cluster2
ICMT cluster2
NFRKB cluster2
ZNF644 cluster2
APEH cluster2

PSAP cluster2
TUBGCP5 cluster2
LETMD1 cluster2
PIN4 cluster2
VPS33A cluster2
ADPGK cluster2
ARHGAP11A cluster2
MACROD1 cluster2
GNAI1 cluster2
ARF3 cluster2
MRPL24 cluster2
PER3 cluster2
U2AF2 cluster2
UBE2I cluster2
SMARCE1 cluster2
TMEM141 cluster2
COPZ1 cluster2
SCO1 cluster2
SIKE1 cluster2
GATAD2A cluster2
ACTB cluster2
C4orf27 cluster2
NCOA1 cluster2
HBQ1 cluster2
RPS6KA5 cluster2
MRPL1 cluster2
YWHAH cluster2
GOLGA5 cluster2
PSMG4 cluster2
PAK1IP1 cluster2
DLEU1 cluster2
HYAL2 cluster2
PPP1R37 cluster2
SLC37A4 cluster2
ASPM cluster2
XPNPEP1 cluster2
UBE2Z cluster2
LRRC58 cluster2
PREB cluster2
NUP58 cluster2
PUS7L cluster2
NDUFB8 cluster2
BORCS7 cluster2
SRSF6 cluster2
ATG4B cluster2
MAP3K7 cluster2
SKA3 cluster2
NEO1 cluster2
TOMM22 cluster2
PRPSAP2 cluster2
RWDD4 cluster2
FUT8 cluster2
MTRNR2L8 cluster2
NENF cluster2
MT-ND6 cluster2
R3HDM2 cluster2

LRRC14 cluster2
EMG1 cluster2
NOM1 cluster2
MZT2A cluster2
GARS cluster2
B3GAT2 cluster2
ATOX1 cluster2
WDR45B cluster2
VTA1 cluster2
F12 cluster2
ZNF593 cluster2
TTC5 cluster2
ZNF236 cluster2
PMPCA cluster2
VPS4A cluster2
SFI1 cluster2
MIA3 cluster2
FBXO18 cluster2
WDR47 cluster2
PITPNA cluster2
SPIDR cluster2
FAM3C cluster2
TSR1 cluster2
PRKCSH cluster2
DUSP11 cluster2
GXYLT1 cluster2
ORC5 cluster2
PLEKHA5 cluster2
SRFBP1 cluster2
C9orf78 cluster2
MKLN1 cluster2
ARFIP1 cluster2
PIK3R3 cluster2
DYNLRB1 cluster2
USP31 cluster2
TRAPPC1 cluster2
PPIE cluster2
PRKRIR cluster2
ZFAND2A cluster2
CENPQ cluster2
ERICH1 cluster2
NBEAL1 cluster2
CMPK1 cluster2
STIM2 cluster2
PRDM2 cluster2
RNASEH2A cluster2
TMEM9 cluster2
MTMR3 cluster2
CKAP2L cluster2
XPC cluster2
WASF2 cluster2
TMPO cluster2
SMIM7 cluster2
CCDC137 cluster2
UBE2Q1 cluster2
FAM103A1 cluster2

NT5C cluster2
CLN5 cluster2
ICE2 cluster2
EFTUD2 cluster2
C19orf48 cluster2
CTSH cluster2
FBXW2 cluster2
EVI5 cluster2
LSM4 cluster2
EPN1 cluster2
NIFK cluster2
SNRPN cluster2
CCS cluster2
INPP5F cluster2
PTPN2 cluster2
EIF3B cluster2
FTSJ2 cluster2
HINT2 cluster2
LRPPRC cluster2
FAM50B cluster2
SQSTM1 cluster2
TFDP1 cluster2
MORC1 cluster2
USP14 cluster2
MRPL32 cluster2
MCM7 cluster2
STRA13 cluster2
UNG cluster2
NFYC cluster2
DNAJC9 cluster2
IARS2 cluster2
UCHL1 cluster2
HSPA1B cluster2
SREK1 cluster2
IWS1 cluster2
TMED4 cluster2
FKBP3 cluster2
DCAF4L2 cluster2
PELP1 cluster2
GTF2F2 cluster2
UBTF cluster2
ENDOV cluster2
ZFYVE21 cluster2
CSNK1G3 cluster2
ASAP1 cluster2
FIS1 cluster2
CFL1 cluster2
CALM1 cluster2
UBE2C cluster2
C20orf27 cluster2
RPS2 cluster2
APRT cluster2
DMRT1 cluster2
RPS28 cluster2
PAFAH1B3 cluster2
ACTG1 cluster2

ESX1 cluster2
SAP25 cluster2
COX20 cluster2
EI24 cluster2
NOP10 cluster2
RPS8 cluster2
NME1 cluster2
HMGA1 cluster2
TXN cluster2
GCSH cluster2
RPSA cluster2
TUBA1C cluster2
C4orf48 cluster2
TMA7 cluster2
TIMM13 cluster2
RPL18A cluster2
TMSB10 cluster2
MYL12A cluster2
RPL10A cluster2
PTS cluster2
TLE1 cluster2
CYC1 cluster2
GNL3 cluster2
ACTL8 cluster2
ATP5I cluster2
RPS19BP1 cluster2
ACRC cluster2
ASAH1 cluster2
OAZ1 cluster2
SRM cluster2
RPL10 cluster2
PLEKHJ1 cluster2
SOHLH1 cluster2
ISOC1 cluster2
NMT2 cluster2
SIGIRR cluster2
HMBS cluster2
COMMD6 cluster2
QDPR cluster2
SPATS2L cluster2
CTBP2 cluster2
NMRAL1 cluster2
PGK1 cluster2
SLC25A5 cluster2
CECR5 cluster2
WDR34 cluster2
PPP1CA cluster2
DCTPP1 cluster2
POLE3 cluster2
CD3EAP cluster2
TBL1XR1 cluster2
CD9 cluster2
APOA1BP cluster2
DNPH1 cluster2
EXOSC5 cluster2
AP1S1 cluster2

IMPDH2 cluster2
CCND1 cluster2
CHD6 cluster2
RPL6 cluster2
MRPL15 cluster2
EDF1 cluster2
CD99 cluster2
POP7 cluster2
PDCD2 cluster2
DRAP1 cluster2
AGTRAP cluster2
MCM5 cluster2
MRPS34 cluster2
PPP1R14A cluster2
NHP2 cluster2
KRTCAP3 cluster2
CDT1 cluster2
ENOPH1 cluster2
B4GALT3 cluster2
KCTD20 cluster2
LAMTOR4 cluster2
C16orf59 cluster2
TTF1 cluster2
MLX cluster2
ARHGEF26 cluster2
E2F6 cluster2
POLR3K cluster2
MKI67 cluster2
TMEM256 cluster2
FEZ2 cluster2
RCAN3 cluster2
ARMC10 cluster2
ALKBH2 cluster2
SLC39A4 cluster2
TFB2M cluster2
ASNA1 cluster2
POLR1C cluster2
COPS7A cluster2
DPH2 cluster2
KLHL15 cluster2
NEFH cluster2
TSEN54 cluster2
USO1 cluster2
SYNC cluster2
KIT cluster2
SORL1 cluster2
SNAPIN cluster2
TSEN2 cluster2
AP3S1 cluster2
FXN cluster2
DCAF4 cluster2
RPL27A cluster2
GRSF1 cluster2
FARSA cluster2
DLL3 cluster2
STIP1 cluster2

AFMID cluster2
CLPTM1L cluster2
CCDC107 cluster2
RPIA cluster2
GPATCH4 cluster2
CLOCK cluster2
ANP32E cluster2
GPC4 cluster2
FAM213A cluster2
STUB1 cluster2
HOXB4 cluster2
IMP3 cluster2
YIF1A cluster2
COPE cluster2
YRDC cluster2
NARS cluster2
CISD3 cluster2
UQCC3 cluster2
MCM2 cluster2
COX4I1 cluster2
SAMD1 cluster2
SDAD1 cluster2
ATG14 cluster2
FGFR1OP2 cluster2
NUDT16L1 cluster2
ARHGDIA cluster2
ZNF296 cluster2
PARP2 cluster2
ABCF1 cluster2
PSMG3 cluster2
PTPRF cluster2
PNO1 cluster2
GCAT cluster2
ADH5 cluster2
TXLNG cluster2
NUCKS1 cluster2
SLC25A11 cluster2
MIER1 cluster2
PLIN2 cluster2
ENOSF1 cluster2
FPGS cluster2
QTRTD1 cluster2
TRAPPC6A cluster2
HACD2 cluster2
SSBP4 cluster2
DVL2 cluster2
MRTO4 cluster2
RAB22A cluster2
NT5DC2 cluster2
SS18L2 cluster2
PHB cluster2
PIM3 cluster2
ADPRHL2 cluster2
RILP cluster2
ASF1A cluster2
TSNAX cluster2

HOMER2 cluster2
MBTPS1 cluster2
NDUFA1 cluster2
LARP1 cluster2
MYBBP1A cluster2
POLE2 cluster2
CDK2AP2 cluster2
POLR3G cluster2
IDH1 cluster2
NOP16 cluster2
NR2C2AP cluster2
TAF9B cluster2
QTRT1 cluster2
SURF1 cluster2
TMEM106C cluster2
PEPD cluster2
MAPK6 cluster2
PPAN cluster2
C1GALT1 cluster2
MSH6 cluster2
THOC6 cluster2
GOLT1B cluster2
BPHL cluster2
LAPTM4B cluster2
NBN cluster2
TAF1D cluster2
UQCR11 cluster2
TGOLN2 cluster2
FAM219B cluster2
UBE2E1 cluster2
RRS1 cluster2
MYO1B cluster2
C1orf131 cluster2
GRWD1 cluster2
CCNA2 cluster2
WDR77 cluster2
BRD3 cluster2
KCTD5 cluster2
NOB1 cluster2
G3BP1 cluster2
MAEA cluster2
POLR2G cluster2
LYRM4 cluster2
TBC1D7 cluster2
MRPL4 cluster2
ATXN3 cluster2
NDUFB11 cluster2
AIFM1 cluster2
CCDC85B cluster2
ECI1 cluster2
PIGX cluster2
FOXRED2 cluster2
NDUFB9 cluster2
THAP11 cluster2
PTBP1 cluster2
CPNE7 cluster2

SMARCD1 cluster2
APC cluster2
DCXR cluster2
GTF3C5 cluster2
TRAF3IP1 cluster2
DNAAF5 cluster2
RP11-366H4.1 cluster2
COX19 cluster2
MRPL12 cluster2
MANBAL cluster2
PRKD2 cluster2
NDUFA11 cluster2
GIGYF1 cluster2
DUSP22 cluster2
PARP16 cluster2
SLC20A1 cluster2
SNX10 cluster2
POLR3H cluster2
UBE2O cluster2
EIF2S3 cluster2
TRAPPC4 cluster2
FDX1L cluster2
AEBP2 cluster2
DARS cluster2
RP4-594I10.3 cluster2
ULK3 cluster2
DHPS cluster2
SRPRA cluster2
SRD5A3 cluster2
FAM32A cluster2
CTTN cluster2
GNPTAB cluster2
HES4 cluster2
WIPF2 cluster2
UHRF1 cluster2
FBLN2 cluster2
NCOA7 cluster2
NAA10 cluster2
ARFGAP2 cluster2
SNX11 cluster2
COQ10A cluster2
NUS1 cluster2
BCL2L12 cluster2
C19orf25 cluster2
RRP9 cluster2
MORF4L2 cluster2
CTPS1 cluster2
ARRB2 cluster2
GAGE13 cluster2
CHAMP1 cluster2
MYSM1 cluster2
THAP9-AS1 cluster2
MYBL2 cluster2
ARL6IP6 cluster2
ZBTB24 cluster2
C22orf39 cluster2

TMTC4 cluster2
MPZL1 cluster2
TMED1 cluster2
JAG2 cluster2
G6PD cluster2
MAP1A cluster2
CEP68 cluster2
CASP7 cluster2
TRAP1 cluster2
ZCCHC10 cluster2
PDCD2L cluster2
BABAM1 cluster2
RSAD1 cluster2
XIST cluster2
SURF6 cluster2
PAK2 cluster2
ATG12 cluster2
ETV7 cluster2
MRPL49 cluster2
TIMELESS cluster2
MAP3K5 cluster2
TMEM223 cluster2
SHTN1 cluster2
ARL6IP4 cluster2
MRPS18B cluster2
FAM98A cluster2
RPE cluster2
BYSL cluster2
HIBADH cluster2
RMND5A cluster2
RRP12 cluster2
RRP1 cluster2
PREPL cluster2
GSE1 cluster2
ELK1 cluster2
SLC52A2 cluster2
EPB41L5 cluster2
TSPAN4 cluster2
OSGEP cluster2
SPOPL cluster2
MED6 cluster2
CNP cluster2
YIF1B cluster2
RP11-12G12.7 cluster2
BIK cluster2
NELFA cluster2
MED16 cluster2
RFXAP cluster2
ITPA cluster2
B3GAT3 cluster2
EMC1 cluster2
DDX54 cluster2
SLC25A4 cluster2
NRDE2 cluster2
ITM2C cluster2
MYCBP2 cluster2

CGREF1 cluster2
SLC35C2 cluster2
TRAF3 cluster2
SMARCA4 cluster2
PNKD cluster2
WDYHV1 cluster2
KIAA1551 cluster2
COQ10B cluster2
SEPHS1 cluster2
NARS2 cluster2
SEMA4D cluster2
IQCA1 cluster2
USP3 cluster2
HRAS cluster2
SIRT7 cluster2
BNIP2 cluster2
TPD52L2 cluster2
GFM1 cluster2
RNF5 cluster2
TK1 cluster2
CST6 cluster2
TNKS2 cluster2
NDFIP1 cluster2
TSPY2 cluster2
EPB41L4B cluster2
MAPRE1 cluster2
RPUSD4 cluster2
ATP6V1H cluster2
BAHCC1 cluster2
RP11-10A14.5 cluster2
C6orf1 cluster2
PRR13 cluster2
TOR1AIP2 cluster2
GIPC1 cluster2
SNHG15 cluster2
CYB5R3 cluster2
CKAP4 cluster2
NAT9 cluster2
RBPMS2 cluster2
EIF2AK4 cluster2
RUFY2 cluster2
CXADR cluster2
OXA1L cluster2
RRAGD cluster2
COA6 cluster2
CAD cluster2
UBE3D cluster2
PPARG cluster2
G6PC3 cluster2
SCRN2 cluster2
IFI27L2 cluster2
EGLN1 cluster2
PUS1 cluster2
POLR3D cluster2
TYW1 cluster2
CNPPD1 cluster2

CCM2 cluster2
RP11-294J22.6 cluster2
SNX1 cluster2
CIAO1 cluster2
SECISBP2L cluster2
CDC23 cluster2
RCC2 cluster2
FGD6 cluster2
DUSP14 cluster2
ACLY cluster2
MBOAT2 cluster2
UTP4 cluster2
ACP2 cluster2
C17orf89 cluster2
NIP7 cluster2
VPS37C cluster2
MTERF3 cluster2
C3orf17 cluster2
MRPS35 cluster2
TIGAR cluster2
CCDC115 cluster2
RAB9A cluster2
BCAT2 cluster2
TMEM43 cluster2
GALK1 cluster2
MRPS28 cluster2
TSC2 cluster2
IMP4 cluster2
CHMP4B cluster2
CREG1 cluster2
TXN2 cluster2
NOP2 cluster2
ST3GAL5 cluster2
APPL2 cluster2
NRAS cluster2
DIMT1 cluster2
KMT2B cluster2
METTL1 cluster2
CLUH cluster2
INTU cluster2
PRMT2 cluster2
SLC35B4 cluster2
KPTN cluster2
SEPW1 cluster2
CCNT2 cluster2
ZNF212 cluster2
PPRC1 cluster2
NCBP2-AS2 cluster2
SOHLH2 cluster2
TPRA1 cluster2
PPIF cluster2
COX16 cluster2
ALKBH4 cluster2
PNKP cluster2
ANHX cluster2
POLR3A cluster2

ZFP90 cluster2
EXOSC1 cluster2
GCN1 cluster2
C3orf58 cluster2
N4BP1 cluster2
MAP3K1 cluster2
PPP1R14B cluster2
DDX19A cluster2
GPATCH3 cluster2
SHQ1 cluster2
IBTK cluster2
RHBDD3 cluster2
ENSA cluster2
MAZ cluster2
MTA3 cluster2
CDK9 cluster2
ZMAT5 cluster2
TLE3 cluster2
FBXO21 cluster2
ZSWIM6 cluster2
ZNF626 cluster2
RNF145 cluster2
KRI1 cluster2
MRPL23 cluster2
STX10 cluster2
CTCF cluster2
SOCS5 cluster2
TFRC cluster2
POP1 cluster2
MARK2 cluster2
ANGEL2 cluster2
NELFB cluster2
PCGF2 cluster2
GNPDA1 cluster2
ACVR1B cluster2
TMEM55B cluster2
TIMM44 cluster2
SNX25 cluster2
LINC00998 cluster2
MRPS2 cluster2
TP53BP1 cluster2
IREB2 cluster2
PPIH cluster2
SMAGP cluster2
RELA cluster2
AK3 cluster2
TRPC4AP cluster2
QSOX2 cluster2
NOP14 cluster2
CROCC cluster2
SCAF4 cluster2
POU2F1 cluster2
CYB5A cluster2
TMEM203 cluster2
EHMT2 cluster2
PNRC2 cluster2

MED9 cluster2
FHL2 cluster2
MRPL55 cluster2
VOPP1 cluster2
TULP4 cluster2
SIPA1L2 cluster2
DIP2B cluster2
BRAT1 cluster2
MRPL35 cluster2
RNF220 cluster2
KIAA0907 cluster2
ACTR1B cluster2
ZNF84 cluster2
JOSD1 cluster2
CPSF4 cluster2
PNMAL1 cluster2
METTL2B cluster2
ANKRD40 cluster2
AKAP8 cluster2
PRR14 cluster2
SPTY2D1 cluster2
NADK cluster2
ABCC5 cluster2
ABL2 cluster2
SNX5 cluster2
SEL1L cluster2
TMED8 cluster2
MLXIP cluster2
ANKRD39 cluster2
DPM2 cluster2
DDIAS cluster2
AKAP11 cluster2
PHC3 cluster2
SLC7A6OS cluster2
LCLAT1 cluster2
RAB11FIP2 cluster2
TARS cluster2
HILPDA cluster2
WDR46 cluster2
ZNF791 cluster2
TDG cluster2
RNASEH1 cluster2
SCAP cluster2
SEC31A cluster2
INIP cluster2
FAM83D cluster2
ZYG11B cluster2
MAP3K4 cluster2
SMIM4 cluster2
ZNF502 cluster2
CTDNEP1 cluster2
ATP5SL cluster2
RCCD1 cluster2
OSBPL11 cluster2
ZNF511 cluster2
KCTD3 cluster2

GLOD4 cluster2
TP53BP2 cluster2
MRPL40 cluster2
XPO5 cluster2
NUDT1 cluster2
FAM133A cluster2
PTP4A3 cluster2
TMSB4X cluster2
ZDHHC17 cluster2
U2AF1 cluster2
C12orf57 cluster2
GTF2E1 cluster2
NUP50 cluster2
PARPBP cluster2
TMEM138 cluster2
CTSB cluster2
COPG2 cluster2
RPP14 cluster2
MRPS18A cluster2
CHTF18 cluster2
RHOB cluster2
SCAMP4 cluster2
TSTD2 cluster2
QSER1 cluster2
SETD6 cluster2
HS2ST1 cluster2
MLLT6 cluster2
IFNGR1 cluster2
HOXB3 cluster2
YTHDF3 cluster2
DDX59 cluster2
ACO2 cluster2
IKZF5 cluster2
GLYR1 cluster2
RRN3 cluster2
TWF1 cluster2
MEST cluster2
FBXO45 cluster2
GTF2B cluster2
TRIT1 cluster2
ECSIT cluster2
CHERP cluster2
OTUD3 cluster2
TMEM11 cluster2
TPD52 cluster2
VAPB cluster2
TBC1D9B cluster2
TRUB2 cluster2
DLG1 cluster2
CDC16 cluster2
FAM173B cluster2
TMEM131 cluster2
NCOA6 cluster2
EVL cluster2
ACTR3B cluster2
ELAC2 cluster2

PIKFYVE cluster2
MSH3 cluster2
AAAS cluster2
MPV17 cluster2
MPRIP cluster2
DAG1 cluster2
YIPF3 cluster2
UBE3C cluster2
CIC cluster2
CDC37 cluster2
ACTR5 cluster2
ABHD17A cluster2
CCDC124 cluster2
MAT2B cluster2
STX12 cluster2
MRPL10 cluster2
EML4 cluster2
ANO10 cluster2
ZNF3 cluster2
TOB2 cluster2
POR cluster2
GBAS cluster2
MED1 cluster2
CDKN2AIPNL cluster2
PUSL1 cluster2
ACO1 cluster2
FNBP1L cluster2
KIAA1841 cluster2
COX14 cluster2
MTR cluster2
FBXO28 cluster2
NECAP1 cluster2
FAM210A cluster2
BTBD7 cluster2
TEX10 cluster2
SLC30A5 cluster2
ILVBL cluster2
NUP160 cluster2
PPP4R3B cluster2
APIP cluster2
EBP cluster2
MRE11A cluster2
SNX4 cluster2
PPTC7 cluster2
MKNK2 cluster2
PPT1 cluster2
MMS19 cluster2
TMEM167B cluster2
ABCD3 cluster2
ASNSD1 cluster2
ETFDH cluster2
COMMD9 cluster2
NCAPH2 cluster2
ERC1 cluster2
GRPEL1 cluster2
REPS1 cluster2

RPRD2 cluster2
OSTM1 cluster2
PSMD9 cluster2
DFFA cluster2
RABL2B cluster2
DIAPH1 cluster2
HDAC3 cluster2
TRAF4 cluster2
GLTP cluster2
KLF10 cluster2
HDLBP cluster2
ZNF267 cluster2
MKNK1 cluster2
TCEA1 cluster2
MYH10 cluster2
RP11-255M2.3 cluster2
LMLN cluster2
RPA1 cluster2
SDHAF2 cluster2
DCAF8 cluster2
CTNNA1 cluster2
HDGF cluster2
CARKD cluster2
AUTS2 cluster2
C5orf45 cluster2
MRPS12 cluster2
TAOK2 cluster2
H1FX cluster2
SUMO3 cluster2
RNF157 cluster2
AP3M2 cluster2
FAM168B cluster2
VPS72 cluster2
ALG2 cluster2
NAA16 cluster2
RRAGC cluster2
NECAP2 cluster2
HM13 cluster2
GPS1 cluster2
COASY cluster2
EHMT1 cluster2
ATL2 cluster2
RBM14 cluster2
NUFIP1 cluster2
TLN1 cluster2
NGFRAP1 cluster2
RAB5A cluster2
CASP2 cluster2
LRBA cluster2
EPN2 cluster2
SIPA1L1 cluster2
AC090498.1 cluster2
ARHGEF33 cluster2
RUNX1 cluster2
MIOS cluster2
IDH3B cluster2

GOLGA3 cluster2
TMED3 cluster2
CREBBP cluster2
ANKRA2 cluster2
BPGM cluster2
XPA cluster2
WIZ cluster2
NDN cluster2
RGS12 cluster2
TMEM69 cluster2
RUNDC3B cluster2
CWC22 cluster2
ALG14 cluster2
YAF2 cluster2
NDUFS2 cluster2
HNRNPL cluster2
PCNX cluster2
NIT1 cluster2
GPKOW cluster2
TUBD1 cluster2
RHOG cluster2
USB1 cluster2
RBMXL1 cluster2
VHL cluster2
MAGEA12 cluster2
METTL9 cluster2
SMIM8 cluster2
NUDT5 cluster2
ATG16L1 cluster2
WDSUB1 cluster2
GOLPH3 cluster2
SART1 cluster2
DCBLD2 cluster2
ZUFSP cluster2
HEXA cluster2
SPAST cluster2
MTFMT cluster2
MADD cluster2
KCTD6 cluster2
INO80 cluster2
SCAF8 cluster2
METTL8 cluster2
BROX cluster2
OXSR1 cluster2
HIC2 cluster2
THUMPD2 cluster2
PBDC1 cluster2
AC069277.2 cluster2
ZNF226 cluster2
DPY19L3 cluster2
UVRAG cluster2
COG2 cluster2
ERCC3 cluster2
CBFB cluster2
PRRC1 cluster2
RPP21 cluster2

AKT2 cluster2
TBCD cluster2
KIAA0368 cluster2
PAN3 cluster2
ZNF532 cluster2
DDX41 cluster2
EXOC2 cluster2
GABPB1 cluster2
HNRNPU cluster2
C18orf8 cluster2
FNIP2 cluster2
SBF1 cluster2
AKAP17A cluster2
TCHP cluster2
ZMYND11 cluster2
MED25 cluster2
SNRPD2 cluster3
LUZP1 cluster3
OAZ2 cluster3
NRBP1 cluster3
KIF21A cluster3
ERGIC3 cluster3
TEX19 cluster3
SCML1 cluster3
CLSPN cluster3
DPH7 cluster3
TEX101 cluster3
MEIOB cluster3
DNAJC1 cluster3
KCNQ1OT1 cluster3
GPAA1 cluster3
HORMAD1 cluster3
GINS2 cluster3
SYCP2 cluster3
VPS29 cluster3
SYCE2 cluster3
ZCCHC17 cluster3
ABCA5 cluster3
CCDC73 cluster3
SYCE1 cluster3
PRAP1 cluster3
CT47B1 cluster3
NPTX2 cluster3
PRSS21 cluster3
PRIM1 cluster3
TCTEX1D2 cluster3
TMEM59 cluster3
C11orf85 cluster3
SFR1 cluster3
FAM9B cluster3
FAM9C cluster3
C20orf24 cluster3
FAM105A cluster3
SMCHD1 cluster3
TAOK3 cluster3
INCA1 cluster3

RNF34 cluster3
WNK1 cluster3
MRPL18 cluster3
CDC6 cluster3
STAG3 cluster3
MPC1 cluster3
BRCA1 cluster3
DMC1 cluster3
CYP20A1 cluster3
RP13-347D8.7 cluster3
C19orf52 cluster3
CCNDBP1 cluster3
VPS35 cluster3
RNF212B cluster3
CT55 cluster3
BEND2 cluster3
CMC2 cluster3
STRIP2 cluster3
GPAT2 cluster3
YME1L1 cluster3
CHID1 cluster3
GGCT cluster3
LLNLR-245B6.1 cluster3
HLA-B cluster3
C19orf57 cluster3
FLYWCH2 cluster3
HIF1A cluster3
TMEM147 cluster3
KIF1A cluster3
AAMDC cluster3
HDAC6 cluster3
PGRMC1 cluster3
RRM1 cluster3
RNF216 cluster3
ARL1 cluster3
TEX12 cluster3
TOP2B cluster3
RAD51AP2 cluster3
CCDC79 cluster3
EIF2S1 cluster3
LPCAT2 cluster3
OTUD6A cluster3
PEG10 cluster3
C12orf65 cluster3
PSMA8 cluster3
EYA3 cluster3
YIPF4 cluster3
ESCO2 cluster3
SGSM3 cluster3
TRAFD1 cluster3
CNTRL cluster3
UBE2F cluster3
RAB6A cluster3
HAUS5 cluster3
IQSEC3 cluster3
HSPA14 cluster3

TEX15 cluster3
ASF1B cluster3
MAGEA10 cluster3
RBM6 cluster3
ANKRD31 cluster3
CCNB1IP1 cluster3
ATP6AP2 cluster3
JADE3 cluster3
NFAT5 cluster3
POU2F2 cluster3
CCNE2 cluster3
AC005324.6 cluster3
RAD9B cluster3
CENPU cluster3
SYNGR4 cluster3
RHNO1 cluster3
PKMYT1 cluster3
CCDC152 cluster3
C21orf58 cluster3
CTNNAL1 cluster3
ATP11C cluster3
PRDM9 cluster3
RAD51C cluster3
PIGS cluster3
CCDC47 cluster3
GOLGA2 cluster3
ARHGAP5 cluster3
GTF2H2 cluster3
NUP107 cluster3
LTBP4 cluster3
PDHA1 cluster3
ZNF280B cluster3
FZD3 cluster3
TIPIN cluster3
FBXL5 cluster3
MCM3AP-AS1 cluster3
DMRTC2 cluster3
FAM9A cluster3
WDR6 cluster3
CHGB cluster3
FEN1 cluster3
FANCI cluster3
MCM8 cluster3
BBS4 cluster3
EEA1 cluster3
EP400 cluster3
MEIOC cluster3
ATAD5 cluster3
XX-CR54.1 cluster3
CPT2 cluster3
C1orf146 cluster3
BHMG1 cluster3
MARK3 cluster3
ISY1 cluster3
ERP44 cluster3
DHX40 cluster3

PCGF6 cluster3
SLC39A1 cluster3
NUSAP1 cluster3
FANCA cluster3
TMEM116 cluster3
PPIC cluster3
FBXO47 cluster3
SDHD cluster3
CKAP5 cluster3
STXBP1 cluster3
RIC8A cluster3
CHD9 cluster3
PIGK cluster3
FBXO11 cluster3
CHEK1 cluster3
NOL8 cluster3
ITFG1 cluster3
TSPAN3 cluster3
GDE1 cluster3
PHIP cluster3
C14orf39 cluster3
STK25 cluster3
MAGEA11 cluster3
VMP1 cluster3
COCH cluster3
CNOT6L cluster3
CCNB3 cluster3
SGK494 cluster3
TTC32 cluster3
DCK cluster3
C15orf40 cluster3
HIST1H2AA cluster3
MAGEA9B cluster3
GOLGB1 cluster3
ARHGEF10 cluster3
TPST1 cluster3
ACSS2 cluster3
U82695.5 cluster3
7-Sep cluster3
PTK2 cluster3
FAM208B cluster3
PEG3 cluster3
CNTLN cluster3
C6orf203 cluster3
ACTR3 cluster3
TIPRL cluster3
CDC45 cluster3
ETFA cluster3
RFC4 cluster3
HDDC3 cluster3
ACBD3 cluster3
NDUFS1 cluster3
CABIN1 cluster3
LINC01024 cluster3
SAMD4B cluster3
POLE cluster3

MRPL11 cluster3
PAGE4 cluster3
SMC1A cluster3
CRY1 cluster3
ZW10 cluster3
MACF1 cluster3
LARS cluster3
MSH2 cluster3
EPB41 cluster3
FBXO5 cluster3
PAK1 cluster3
LAMTOR3 cluster3
PHTF2 cluster3
CDKN2C cluster3
HLA-C cluster3
KMT5C cluster3
XKR9 cluster3
ANKHD1 cluster3
BMPR2 cluster3
GALNT11 cluster3
SNCA cluster3
AP1S2 cluster3
NDUFA10 cluster3
MMP25-AS1 cluster3
RRM2 cluster3
SERPINH1 cluster3
HAUS3 cluster3
HINFP cluster3
SLMAP cluster3
ATP6AP1 cluster3
C18orf63 cluster3
ZNF714 cluster3
TEX11 cluster3
ZWINT cluster3
TMEM97 cluster3
MLEC cluster3
PROCR cluster3
BBS7 cluster3
PXMP2 cluster3
CRELD1 cluster3
SYCP1 cluster3
TIGD3 cluster3
C16orf70 cluster3
PRDX4 cluster3
KDELR1 cluster3
GDPD1 cluster3
PRPF18 cluster3
SPO11 cluster3
CLN8 cluster3
MAGEA1 cluster3
GABPB2 cluster3
PSMC3IP cluster3
GLCE cluster3
NSD1 cluster3
SENP5 cluster3
TUSC3 cluster3

SUCO cluster3
SH3KBP1 cluster3
HFM1 cluster3
CCP110 cluster3
RACGAP1 cluster3
TCHH cluster3
PRSS38 cluster3
LRRC41 cluster3
AADAT cluster3
SPCS3 cluster3
IFT81 cluster3
RHOT1 cluster3
P4HA1 cluster3
DENND2C cluster3
RP11-160H22.5 cluster3
CSPP1 cluster3
KREMEN2 cluster3
ZNF446 cluster3
PSMD5-AS1 cluster3
SYTL1 cluster3
BOD1L1 cluster3
CEP78 cluster3
DSEL cluster3
NCAPG cluster3
FAM157C cluster3
PAX6 cluster3
CCDC122 cluster3
NCOA3 cluster3
LETM1 cluster3
USP47 cluster3
NUMB cluster3
HESX1 cluster3
PBX3 cluster3
TRIP13 cluster3
BFAR cluster3
EXOC5 cluster3
PKN2 cluster3
SSX1 cluster3
H2AFX cluster3
BUB1B cluster3
THOC2 cluster3
FHOD1 cluster3
MED8 cluster3
IPO9 cluster3
RP11-522I20.3 cluster3
BLOC1S2 cluster3
MYT1L cluster3
C6orf136 cluster3
REL cluster3
CENPP cluster3
HADHA cluster3
TNKS2-AS1 cluster3
CLASRP cluster3
ANAPC7 cluster3
RP11-422P24.12 cluster3
DLEU2 cluster3

COPB2 cluster3
PTGR2 cluster3
KNTC1 cluster3
CES2 cluster3
AP006222.2 cluster3
GBA cluster3
ACTN4 cluster3
WEE1 cluster3
PPIP5K2 cluster3
APPL1 cluster3
PJA2 cluster3
CUTA cluster3
SLC35B1 cluster3
WDR3 cluster3
DONSON cluster3
FAM98C cluster3
SPTBN1 cluster3
CCDC18 cluster3
PCMTD2 cluster3
BET1 cluster3
C9orf84 cluster3
CCDC15 cluster3
DIP2A cluster3
PRDM7 cluster3
SLC31A1 cluster3
CLDN18 cluster3
SPAG7 cluster3
MMD cluster3
SLC25A17 cluster3
RAPH1 cluster3
CCDC64 cluster3
MYOM2 cluster3
NDC80 cluster3
TMUB2 cluster3
ARFGEF1 cluster3
C3orf14 cluster3
KLHL22 cluster3
COQ2 cluster3
ANKRD12 cluster3
STOM cluster3
FBXL20 cluster3
CALY cluster3
CCDC93 cluster3
CCDC58 cluster3
TAF4B cluster3
PLA2G12A cluster3
RFWD2 cluster3
VAMP7 cluster3
LINC01140 cluster3
TRAPPC8 cluster3
VWA9 cluster3
CHST12 cluster3
NPRL3 cluster3
HORMAD2 cluster3
MAPKAPK5-AS1 cluster3
ZNF280C cluster3

GBF1 cluster3
ADCK5 cluster3
KLHL8 cluster3
CISD2 cluster3
KIF18A cluster3
FN3KRP cluster3
PIP5K1A cluster3
SGCE cluster3
SRR cluster3
MNT cluster3
LPCAT3 cluster3
AUNIP cluster3
ALG13 cluster3
UBL7-AS1 cluster3
CTSV cluster3
OAT cluster3
SMARCC2 cluster3
TOP3A cluster3
CNPY2 cluster3
PHF8 cluster3
C5orf42 cluster3
POLA2 cluster3
TMEM52 cluster3
DCTN5 cluster3
MROH1 cluster3
MTMR9 cluster3
ZNF318 cluster3
GMEB1 cluster3
RTCA cluster3
NDUFS5 cluster3
CENPN cluster3
CERCAM cluster3
ERBB3 cluster3
UHRF2 cluster3
SLC39A10 cluster3
KANSL2 cluster3
AMN cluster3
DDHD2 cluster3
COG4 cluster3
PRPF38A cluster3
ZFYVE16 cluster3
AASDH cluster3
TRAF3IP2-AS1 cluster3
ZNF672 cluster3
EXT2 cluster3
WDR76 cluster3
GAL3ST1 cluster3
ASZ1 cluster3
KIAA0100 cluster3
YTHDC2 cluster3
N4BP2L2 cluster3
B3GALNT2 cluster3
FAM41C cluster3
ARFRP1 cluster3
SLC9A5 cluster3
EPB41L2 cluster3

TRA2A cluster3
MAGEB6 cluster3
SETD5 cluster3
PPP5C cluster3
MXI1 cluster3
NT5C3A cluster3
HAUS6 cluster3
P3H1 cluster3
SRBD1 cluster3
LCMT2 cluster3
ZNHIT6 cluster3
EPS15 cluster3
MTMR14 cluster3
KMT2A cluster3
MICU1 cluster3
CLSTN1 cluster3
EIF2A cluster3
TEX13D cluster3
CRNKL1 cluster3
RPP25L cluster3
FANCD2 cluster3
SPECC1 cluster3
MUM1 cluster3
CCDC144A cluster3
FAM114A2 cluster3
TMEM242 cluster3
PIK3C2A cluster3
PIDD1 cluster3
PTPN4 cluster3
FIGNL1 cluster3
ECT2 cluster3
RBM26-AS1 cluster3
CCDC155 cluster3
STMND1 cluster3
RBMXL3 cluster3
PARP12 cluster3
IL6ST cluster3
SNX16 cluster3
YTHDF1 cluster3
PXDN cluster3
SUMF2 cluster3
HTRA2 cluster3
CENPL cluster3
SF3B3 cluster3
SLC35F5 cluster3
AAGAB cluster3
MOAP1 cluster3
CS cluster3
CDC27 cluster3
TSC22D2 cluster3
C17orf75 cluster3
AREG cluster3
PALB2 cluster3
ASCC3 cluster3
LSS cluster3
MEI1 cluster3

RNASEH2B cluster3
RAD51 cluster3
CEP192 cluster3
HAT1 cluster3
RAF1 cluster3
KIF11 cluster3
PGD cluster3
C1orf112 cluster3
NFE2L1 cluster3
CEP290 cluster3
MDC1 cluster3
VEZT cluster3
VKORC1 cluster3
M1AP cluster3
DAAM1 cluster3
ROCK2 cluster3
PRPF3 cluster3
ORC4 cluster3
NRXN2 cluster3
MAP3K3 cluster3
ME2 cluster3
SETD2 cluster3
HUWE1 cluster3
NFYB cluster3
TAOK1 cluster3
CBWD1 cluster3
UFL1 cluster3
MCM10 cluster3
SENP1 cluster3
ATP9B cluster3
ZNF257 cluster3
GTF2H2C cluster3
IGF2BP1 cluster3
SLC24A1 cluster3
LINC01572 cluster3
RASAL2 cluster3
TRAPPC6B cluster3
XBP1 cluster3
ZNF326 cluster3
CTNND1 cluster3
BAIAP2L1 cluster3
SPG11 cluster3
CD2AP cluster3
PITPNB cluster3
PLGRKT cluster3
C1orf109 cluster3
CBWD5 cluster3
PLAC1 cluster3
RP11-192H23.6 cluster3
STK17B cluster3
P4HA2 cluster3
ARID4B cluster3
AGTPBP1 cluster3
EAF2 cluster3
MMS22L cluster3
MED14 cluster3

SBF2-AS1 cluster3
ZHX1 cluster3
SHCBP1 cluster3
PANK3 cluster3
IKBIP cluster3
MAP2K1 cluster3
CTNNB1 cluster3
HUS1B cluster3
PHRF1 cluster3
PDCD6IP cluster3
PGM2L1 cluster3
IFIT5 cluster3
MESP1 cluster3
AGFG2 cluster3
CLDN10 cluster3
GART cluster3
ZNF99 cluster3
MAPKAP1 cluster3
RP11-486G15.2 cluster3
NCAPG2 cluster3
RABGAP1 cluster3
LYST cluster3
RECK cluster3
C11orf24 cluster3
AC002480.2 cluster3
TMEM128 cluster3
SNAPC5 cluster3
P4HA3 cluster3
JPX cluster3
TBCCD1 cluster3
GNPDA2 cluster3
RP11-1C8.7 cluster3
VPS13A cluster3
CCDC172 cluster3
ZDHHC13 cluster3
PARP10 cluster3
SDR39U1 cluster3
CPEB4 cluster3
FXR2 cluster3
PDE3B cluster3
LIMK2 cluster3
ZNF136 cluster3
UGT8 cluster3
PEMT cluster3
GTF3C3 cluster3
ATR cluster3
TMEM30A cluster3
NDUFV1 cluster3
ZNF324 cluster3
KLHL24 cluster3
DLAT cluster3
FBXO4 cluster3
TMEM206 cluster3
CXXC1 cluster3
NEK8 cluster3
RDH11 cluster3

HOOK2 cluster3
TCEANC2 cluster3
EXOG cluster3
LNPEP cluster3
ROBO3 cluster3
ZNF622 cluster3
DLGAP5 cluster3
ZNF800 cluster3
DMXL1 cluster3
SMIM13 cluster3
KDM2A cluster3
TSPYL1 cluster3
H1F0 cluster3
FNDC3A cluster3
ISCU cluster3
PWWP2A cluster3
FBXO22 cluster3
VASP cluster3
PRPF40B cluster3
SLK cluster3
CRLF3 cluster3
TSC22D3 cluster3
TMEM259 cluster3
PDP2 cluster3
TIMM10B cluster3
PSME2 cluster3
NCKAP1 cluster3
AARS cluster3
GTF2H3 cluster3
DMXL2 cluster3
RFWD3 cluster3
ABCD4 cluster3
RPA4 cluster3
GPRASP2 cluster3
EBAG9 cluster3
USP37 cluster3
RAB41 cluster3
USP33 cluster3
RBL1 cluster3
ATM cluster3
MAGEC1 cluster3
TMEM101 cluster3
DDX23 cluster3
HCCS cluster3
UTP20 cluster3
KHDC1 cluster3
EMD cluster3
LMBRD2 cluster3
ZCCHC9 cluster3
RSPRY1 cluster3
NPAP1 cluster3
LINC00936 cluster3
NMD3 cluster3
MFN1 cluster3
RIMS2 cluster3
NUCB1 cluster3

ANKIB1 cluster3
TCTN1 cluster3
MBD6 cluster3
HSD17B14 cluster3
MTBP cluster3
LMF1 cluster3
FOXN2 cluster3
TMEM237 cluster3
ZNF394 cluster3
HSD17B4 cluster3
PTPDC1 cluster3
PIK3CB cluster3
MSH4 cluster3
SAP30 cluster3
ZNF655 cluster3
DBF4B cluster3
API5 cluster3
C11orf54 cluster3
PPP1R16A cluster3
RP11-78J21.4 cluster3
OGFOD1 cluster3
USP6 cluster3
MAP4 cluster3
MELK cluster3
BRPF3 cluster3
MPP2 cluster3
CTD-3193K9.4 cluster3
FOXRED1 cluster3
TESK2 cluster3
BOD1 cluster3
RBM4 cluster3
RQCD1 cluster3
C20orf194 cluster3
DNASE1 cluster3
SNX27 cluster3
RALGAPB cluster3
XRN1 cluster3
SDCCAG8 cluster3
COQ9 cluster3
RC3H2 cluster3
ZNF232 cluster3
PAPOLG cluster3
METTL4 cluster3
VPS36 cluster3
CES4A cluster3
ZNF280D cluster3
KIAA1211L cluster3
PPM1B cluster3
UBR5-AS1 cluster3
ZMYM6 cluster3
RB1 cluster3
KIF1BP cluster3
POLR1E cluster3
PEX1 cluster3
ZSWIM8 cluster3
TXLNA cluster3

RANBP2 cluster3
ZNFX1 cluster3
AC020956.3 cluster3
ZBED5 cluster3
E2F4 cluster3
DPEP2 cluster3
STAG2 cluster3
ZNF92 cluster3
ERLIN2 cluster3
RP4-669L17.10 cluster3
TDRKH cluster3
ZNF454 cluster3
KCTD9 cluster3
XRCC2 cluster3
KMT2C cluster3
UCHL5 cluster3
YDJC cluster3
PDIK1L cluster3
TMEM175 cluster3
RELT cluster3
RP11-398K22.12 cluster3
LMBR1 cluster3
POLM cluster3
FARP2 cluster3
IQCH cluster3
DCP2 cluster3
RECQL4 cluster3
N4BP2 cluster3
OTULIN cluster3
HEATR5A cluster3
PSMD5 cluster3
CXorf67 cluster3
UNK cluster3
WDR44 cluster3
STIM1 cluster3
ZCCHC7 cluster3
USP48 cluster3
TMEM98 cluster3
VCL cluster3
CDK5RAP1 cluster3
ZGRF1 cluster3
METTL14 cluster3
MTOR cluster3
EMSY cluster3
BLOC1S6 cluster3
MDK cluster3
ADARB1 cluster3
RP11-983P16.4 cluster3
ZC3HC1 cluster3
PTPRK cluster3
KLC2 cluster3
CHD1 cluster3
MAPK7 cluster3
EPM2AIP1 cluster3
ZNF738 cluster3
FAM193A cluster3

TRPM7 cluster3
MAT2A cluster3
PCBP3 cluster3
PDE8A cluster3
CDK13 cluster3
DNM1L cluster3
GPALPP1 cluster3
RNF24 cluster3
TMEM33 cluster3
C19orf54 cluster3
CDK18 cluster3
FBXO48 cluster3
TRABD cluster3
XAGE3 cluster3
NFXL1 cluster3
FANCG cluster3
USP51 cluster3
RAD18 cluster3
PTPN23 cluster3
TMCC1 cluster3
JMY cluster3
STAM cluster3
ARHGAP5-AS1 cluster3
NBPF1 cluster3
ACAD8 cluster3
ZNF557 cluster3
TMEM17 cluster3
SKA2 cluster3
C2orf44 cluster3
UCK1 cluster3
UBAP2L cluster3
AJ011932.1 cluster3
ADIPOR2 cluster3
CSGALNACT2 cluster3
ZNF430 cluster3
RBFOX2 cluster3
UBXN2A cluster3
RAB3GAP1 cluster3
SIL1 cluster3
CCNL2 cluster3
DHRS13 cluster3
FNTA cluster3
STT3A cluster3
ZNF292 cluster3
LACC1 cluster3
PPP2R3B cluster3
FCHO2 cluster3
ZFC3H1 cluster3
HMGXB3 cluster3
PHLPP2 cluster3
HIPK1 cluster3
GPR89A cluster3
CRTC2 cluster3
PBX4 cluster3
EXOC3 cluster3
TUBB8 cluster3

FBXL12 cluster3
PDE12 cluster3
NUFIP2 cluster3
RPS6KL1 cluster3
DNAJC25 cluster3
PAPD5 cluster3
ZNF652 cluster3
CMTM6 cluster3
PEX11B cluster3
CSTF2 cluster3
PPP4R4 cluster3
ARFGEF2 cluster3
GATAD2B cluster3
OXCT1 cluster3
EIF3J-AS1 cluster3
FGF14-AS2 cluster3
TM9SF3 cluster3
ZNF17 cluster3
MCMDC2 cluster3
CLK1 cluster3
SNTB2 cluster3
KIF14 cluster3
ALG5 cluster3
FAM188A cluster3
ATPAF2 cluster3
ACAA2 cluster3
MASTL cluster3
TMEM216 cluster3
ZNF541 cluster3
PAWR cluster3
TTC33 cluster3
PLEKHA1 cluster3
NARF cluster3
PIGO cluster3
ZNF98 cluster3
ABHD17B cluster3
ELMOD2 cluster3
INTS4 cluster3
POLR3B cluster3
AC136616.1 cluster3
MAP3K13 cluster3
ARHGAP20 cluster3
GGT7 cluster3
PEX12 cluster3
PNPLA4 cluster3
MAN2A1 cluster3
SOWAHC cluster3
EXOC6 cluster3
HERC1 cluster3
DIP2C cluster3
ZC3H8 cluster3
AF186192.6 cluster3
GGCX cluster3
FAM20B cluster3
AGBL4 cluster3
CEPT1 cluster3

UQCRQ cluster3
TRIM41 cluster3
TMUB1 cluster3
RRM2B cluster3
KDM6A cluster3
SELK cluster3
RAB5B cluster3
BANP cluster3
SENP7 cluster3
SIAH1 cluster3
ZNF37A cluster3
SCAI cluster3
YIPF6 cluster3
CHUK cluster3
TINF2 cluster3
SCMH1 cluster3
DDX28 cluster3
STXBP5L cluster3
TMEM39A cluster3
TAF7L cluster3
IPO7 cluster3
USP28 cluster3
POLN cluster3
CSTF2T cluster3
AP3M1 cluster3
MDM4 cluster3
FAM45A cluster3
QSOX1 cluster3
JOSD2 cluster3
SLC35A3 cluster3
C7orf49 cluster3
NFATC3 cluster3
RP11-83A24.2 cluster3
NETO2 cluster3
STIL cluster3
TMEM161B cluster3
RP11-160E2.6 cluster3
TEFM cluster3
ARFIP2 cluster3
CEP72 cluster3
SNRNP48 cluster3
TRIM32 cluster3
SEC16A cluster3
MYBL1 cluster3
HSPA13 cluster3
SUPT7L cluster3
DHRSX cluster3
DHX34 cluster3
ZNF250 cluster3
PPM1F cluster3
CENPBD1 cluster3
UTP23 cluster3
CEP250 cluster3
FAM189B cluster3
RNF8 cluster3
C19orf38 cluster3

ERCC2 cluster3
IQCC cluster3
LINC00667 cluster3
USP40 cluster3
ZBTB21 cluster3
LIN9 cluster3
PIGM cluster3
CHD8 cluster3
SMG1 cluster3
MFN2 cluster3
GIT2 cluster3
CFAP70 cluster3
PBRM1 cluster3
UBE2D1 cluster3
SEC22B cluster3
PLEKHG4 cluster3
RASA1 cluster3
TRIM52 cluster3
ATRN cluster3
PUDP cluster3
UBAC2 cluster3
FO538757.2 cluster3
NRSN2 cluster3
BIN2 cluster3
MORC2 cluster3
GDAP2 cluster3
ANKRD46 cluster3
PI4KA cluster3
ACADVL cluster3
LRP11 cluster3
PHF23 cluster3
KIDINS220 cluster3
PAXBP1 cluster3
TRIM23 cluster3
IVNS1ABP cluster3
SLC39A6 cluster3
10-Sep cluster3
ZNF34 cluster3
SEPSECS cluster3
ADD1 cluster3
FOXJ3 cluster3
FAM126B cluster3
TMEM68 cluster3
NEK9 cluster3
RC3H1 cluster3
UGGT2 cluster3
PAXIP1 cluster3
PLEKHF2 cluster3
PRIMPOL cluster3
ENTPD5 cluster3
RNF121 cluster3
NIFK-AS1 cluster3
AC010524.2 cluster3
GML cluster3
IDH3A cluster3
VCPIP1 cluster3

NAA25 cluster3
RAB40C cluster3
NSF cluster3
TC2N cluster3
SERINC1 cluster3
KATNBL1 cluster3
CRCP cluster3
RP11-332H14.2 cluster3
DNAJC3-AS1 cluster3
VPS13C cluster3
BEX4 cluster3
MED13 cluster3
RP11-412D9.4 cluster3
LGMN cluster3
ITGB1BP1 cluster3
GGACT cluster3
RP11-446F3.2 cluster3
ADAM17 cluster3
KIAA0319L cluster3
SUPV3L1 cluster3
ZFP82 cluster3
ZSCAN32 cluster3
FBXO8 cluster3
IPPK cluster3
CCNC cluster3
VPS16 cluster3
SLC35B3 cluster3
ATF6 cluster3
COMMD7 cluster3
RCHY1 cluster3
KIF1B cluster3
ZNF398 cluster3
FUOM cluster3
MYEF2 cluster3
ZMYM2 cluster3
ZNF721 cluster3
WDR90 cluster3
PRKD3 cluster3
COG6 cluster3
BOLA1 cluster3
RGL2 cluster3
PPME1 cluster3
SMURF2 cluster3
NSUN6 cluster3
ATP6V0A1 cluster3
C5orf63 cluster3
RCOR1 cluster3
SPICE1 cluster3
GINM1 cluster3
PRPF4 cluster3
BTAF1 cluster3
ZMYM1 cluster3
PIK3R4 cluster3
NXF1 cluster3
ZNF431 cluster3
MED30 cluster3

FAM234A cluster3
CBWD2 cluster3
TSPYL6 cluster3
ZNF639 cluster3
XIAP cluster3
PUM1 cluster3
ERI2 cluster3
ST8SIA6-AS1 cluster3
C18orf25 cluster3
CASC4 cluster3
GANC cluster3
APTR cluster3
MUT cluster3
AP4M1 cluster3
SPPL2A cluster3
CEP95 cluster3
PPP6R3 cluster3
PCOLCE cluster3
CAMSAP2 cluster3
POLDIP2 cluster3
DIS3L cluster3
STX16 cluster3
TNRC6A cluster3
TTC19 cluster3
NUP153 cluster3
SPC25 cluster3
CYHR1 cluster3
UBR4 cluster3
CLTC cluster3
SLC5A6 cluster3
PEF1 cluster3
CORO1C cluster3
PAXIP1-AS1 cluster3
RCOR3 cluster3
HN1L cluster3
RAD51AP1 cluster3
WDFY2 cluster3
ATAD2B cluster3
RLF cluster3
MORC3 cluster3
THOC5 cluster3
COPA cluster3
RP11-544A12.8 cluster3
TM7SF2 cluster3
ERRFI1 cluster3
OFD1 cluster3
MACROD2 cluster3
ITCH cluster3
TARSL2 cluster3
ASB8 cluster3
UBE2G2 cluster3
CHORDC1 cluster3
MOV10L1 cluster3
RAB1A cluster3
KIFAP3 cluster3
KRTDAP cluster3

WDR73 cluster3
FLJ37453 cluster3
DDRGK1 cluster3
FAR1 cluster3
MSL3 cluster3
SPG7 cluster3
NEAT1 cluster3
RP11-3B7.1 cluster3
HOTAIR cluster3
RGS5.1 cluster3
HOXC6 cluster3
NET1 cluster3
ZNF662 cluster3
MAU2 cluster3
WDR11 cluster3
KIN cluster3
FAM98B cluster3
TNFAIP8L3 cluster3
DMRT3 cluster3
FTHL17 cluster3
PHKB cluster3
MAGEB1 cluster3
HIST1H4C cluster4
ZCWPW1 cluster4
VCX3B cluster4
SMC1B cluster4
VCX cluster4
SMC3 cluster4
SYCP3 cluster4
VCX3A cluster4
TOP2A cluster4
PRSS50 cluster4
FMR1NB cluster4
DUT cluster4
HMGB1 cluster4
VCX2 cluster4
HMGB2 cluster4
BCAP31 cluster4
DPEP3 cluster4
MALAT1 cluster4
NASP cluster4
HERC5 cluster4
PIGP cluster4
PTMA cluster4
PSMD1 cluster4
CBX1 cluster4
BTG3 cluster4
SDF2L1 cluster4
HPRT1 cluster4
HELLS cluster4
TAF12 cluster4
NCL cluster4
SNRPB cluster4
DSTN cluster4
PAGE1 cluster4
TEX30 cluster4

WBSCR22 cluster4
CENPH cluster4
CASC5 cluster4
DDX24 cluster4
C5orf47 cluster4
YEATS4 cluster4
NPM1 cluster4
TTC3 cluster4
U2SURP cluster4
IQCB1 cluster4
TMEM258 cluster4
MT-CYB cluster4
PPIA cluster4
EIF5B cluster4
RBM17 cluster4
IER3IP1 cluster4
POLR2F cluster4
RHEB cluster4
PPIG cluster4
ROMO1 cluster4
DNAJB11 cluster4
CFDP1 cluster4
THAP7 cluster4
NIPBL cluster4
ARPC5 cluster4
C11orf58 cluster4
SAE1 cluster4
NPC2 cluster4
MT-ATP6 cluster4
DENR cluster4
PTGES3 cluster4
MYDGF cluster4
SEC11A cluster4
GPBP1 cluster4
SUB1 cluster4
MT-ND3 cluster4
HLTF cluster4
C1D cluster4
PRPF31 cluster4
HMCES cluster4
RTN3 cluster4
CHCHD2 cluster4
CALM2 cluster4
SRP14 cluster4
HSP90AA1 cluster4
MAP1LC3B cluster4
RPL21 cluster4
APLP2 cluster4
PSMB2 cluster4
LRPAP1 cluster4
DDX46 cluster4
TKTL1 cluster4
SRSF9 cluster4
TOPAZ1 cluster4
ORMDL1 cluster4
TMEM50B cluster4

ORC6 cluster4
VRK1 cluster4
VAPA cluster4
TMED2 cluster4
HIGD1A cluster4
GOLGA7 cluster4
SLBP cluster4
ADAL cluster4
RPA2 cluster4
ATP6V0E1 cluster4
CHAF1A cluster4
TSPYL2 cluster4
HNRNPH3 cluster4
COA1 cluster4
TAF11 cluster4
EIF1B cluster4
CLNS1A cluster4
UQCRH cluster4
GAGE2A cluster4
UPF2 cluster4
SLF1 cluster4
CNBP cluster4
BRCA2 cluster4
SIVA1 cluster4
TMEM179B cluster4
TALDO1 cluster4
SUMO2 cluster4
SNRNP40 cluster4
UBE2T cluster4
HNRNPC cluster4
SON cluster4
PAGE2B cluster4
APMAP cluster4
FAM175A cluster4
DAZL cluster4
SNRPG cluster4
TERF1 cluster4
XRCC6 cluster4
CDK12 cluster4
SCAF11 cluster4
TEX264 cluster4
NFATC2IP cluster4
GDI2 cluster4
PCBP2 cluster4
HNRNPR cluster4
S100PBP cluster4
ERP29 cluster4
SDHC cluster4
ARIH2 cluster4
CTCFL cluster4
LAMTOR5 cluster4
COX17 cluster4
PSPC1 cluster4
BAZ2A cluster4
HNRNPA2B1 cluster4
CALR cluster4

ZC3H13 cluster4
ESF1 cluster4
SRSF2 cluster4
MAGEC2 cluster4
PDCD7 cluster4
TPR cluster4
SSX3 cluster4
TGS1 cluster4
MT-ND5 cluster4
DZIP3 cluster4
ATF7IP2 cluster4
GPATCH8 cluster4
USP9X cluster4
SNRPD1 cluster4
KIAA1429 cluster4
CKS2 cluster4
DSN1 cluster4
LY6K cluster4
TMED10 cluster4
PRPF8 cluster4
HNRNPK cluster4
NKAP cluster4
MRFAP1 cluster4
VBP1 cluster4
RBM39 cluster4
SMC4 cluster4
SRRM2 cluster4
PARN cluster4
TMED9 cluster4
ANP32B cluster4
THRAP3 cluster4
NAE1 cluster4
CENPV cluster4
PNISR cluster4
OCIAD1 cluster4
COX7B cluster4
RBBP8 cluster4
PAGE2 cluster4
C19orf60 cluster4
DNMT1 cluster4
NKTR cluster4
TMEM80 cluster4
LMAN2 cluster4
XRCC5 cluster4
SELT cluster4
RIF1 cluster4
MAD2L1 cluster4
PPA1 cluster4
SEC62 cluster4
ITM2B cluster4
UBL5 cluster4
ATAD2 cluster4
TERF2IP cluster4
SLC4A1AP cluster4
LSM14A cluster4
ATP6V1G1 cluster4

KIF5B cluster4
PAGE5 cluster4
KRIT1 cluster4
CDC42 cluster4
ITSN2 cluster4
PITHD1 cluster4
CCT3 cluster4
ESCO1 cluster4
SNX6 cluster4
MED11 cluster4
ARPC1A cluster4
HP1BP3 cluster4
YTHDF2 cluster4
CNIH4 cluster4
BANF1 cluster4
PTPN12 cluster4
ZDHHC4 cluster4
HDAC2 cluster4
DTD1 cluster4
RPN2 cluster4
RPS17 cluster4
RPS21 cluster4
SNW1 cluster4
RSRP1 cluster4
JMJD1C cluster4
TIMM8B cluster4
SPCS1 cluster4
RAB7A cluster4
SRP9 cluster4
DDX1 cluster4
RBBP7 cluster4
USP1 cluster4
ARGLU1 cluster4
CTSL cluster4
HDAC1 cluster4
TPTE cluster4
PNN cluster4
M6PR cluster4
MRPS26 cluster4
HSPB11 cluster4
CLTA cluster4
METAP2 cluster4
PDAP1 cluster4
DDOST cluster4
SNRPF cluster4
RCN2 cluster4
POLR2K cluster4
YWHAE cluster4
HSPA5 cluster4
TPX2 cluster4
EPCAM cluster4
SUZ12 cluster4
UTP6 cluster4
DDB1 cluster4
FASTK cluster4
LSM6 cluster4

TAF15 cluster4
TRIM44 cluster4
DDX42 cluster4
CMAS cluster4
RAC1 cluster4
CCAR1 cluster4
CKAP2 cluster4
UBE2B cluster4
SPINT2 cluster4
CENPK cluster4
ARL5A cluster4
TAX1BP1 cluster4
TM2D1 cluster4
RAB14 cluster4
ISCA2 cluster4
FABP5 cluster4
HNRNPA3 cluster4
MTF2 cluster4
RNPS1 cluster4
ATP6V0B cluster4
RBM44 cluster4
NXT1 cluster4
SRSF3 cluster4
NSFL1C cluster4
SRSF7 cluster4
RNF167 cluster4
DNAJB6 cluster4
FKBP2 cluster4
STAG1 cluster4
EBLN3 cluster4
ATP5E cluster4
OSTC cluster4
RAB18 cluster4
ILF2 cluster4
RPS4Y1 cluster4
SNRPE cluster4
SKP1 cluster4
PARK7 cluster4
ZMYM5 cluster4
PDIA3 cluster4
RBX1 cluster4
PTPRA cluster4
KLC1 cluster4
COX7A2L cluster4
NRBF2 cluster4
RHOA cluster4
RAN cluster4
SLC25A36 cluster4
VCP cluster4
BPTF cluster4
TSG101 cluster4
EIF3J cluster4
ARPC3 cluster4
UBA6 cluster4
SNRNP70 cluster4
SAFB cluster4

DDX18 cluster4
TRMT6 cluster4
KNOP1 cluster4
JAGN1 cluster4
VIMP cluster4
CSE1L cluster4
RABEP1 cluster4
CHTOP cluster4
ZNF770 cluster4
ZMAT2 cluster4
AKAP8L cluster4
DCUN1D5 cluster4
CCNI cluster4
TMEM41B cluster4
KIF20B cluster4
HSP90B1 cluster4
FAM208A cluster4
STRADB cluster4
SLC25A3 cluster4
HNRNPAB cluster4
NAA15 cluster4
CIAPIN1 cluster4
TMX1 cluster4
PHF14 cluster4
ANAPC15 cluster4
SQLE cluster4
NDUFA4 cluster4
ZNF75A cluster4
HNRNPUL1 cluster4
PPP4R3A cluster4
UIMC1 cluster4
RBM25 cluster4
MED31 cluster4
CCT8 cluster4
HNRNPA0 cluster4
NEDD8 cluster4
RPL18 cluster4
RBPJ cluster4
UBA2 cluster4
CAND1 cluster4
MLLT4 cluster4
TCL1A cluster4
CASP8AP2 cluster4
GPANK1 cluster4
ZFAND5 cluster4
HSPE1 cluster4
MPHOSPH8 cluster4
DNAJA2 cluster4
CIRBP cluster4
KDM1A cluster4
TMX2 cluster4
LAMTOR1 cluster4
STAU1 cluster4
RP11-452F19.3 cluster4
SRSF10 cluster4
KDM5A cluster4

NOVA1 cluster4
THUMPD3-AS1 cluster4
DEK cluster4
ATP2B1 cluster4
ERH cluster4
MOB4 cluster4
SAR1A cluster4
PRELID1 cluster4
PPP2CA cluster4
ZFR cluster4
EIF4G2 cluster4
BCCIP cluster4
MAGEA3 cluster4
RPS26 cluster4
TOP1 cluster4
DNAJC19 cluster4
CMSS1 cluster4
CHD4 cluster4
MPHOSPH10 cluster4
PFDN2 cluster4
DHX29 cluster4
SEC11C cluster4
SSB cluster4
WTAP cluster4
TACC1 cluster4
CHIC2 cluster4
PDIA6 cluster4
LAGE3 cluster4
FAM133B cluster4
HSPA8 cluster4
SF3A3 cluster4
TMEM87A cluster4
AUP1 cluster4
PDS5A cluster4
CTR9 cluster4
CHRAC1 cluster4
HMGN2 cluster4
DDX21 cluster4
AP3D1 cluster4
HNRNPM cluster4
ARFGAP3 cluster4
WDR83OS cluster4
ANAPC5 cluster4
GABARAPL2 cluster4
SMG6 cluster4
POLR2L cluster4
CNOT4 cluster4
UBXN1 cluster4
MTX2 cluster4
CHMP2B cluster4
ANKRD54 cluster4
ZNF638 cluster4
POLR2J cluster4
MIS18BP1 cluster4
C16orf87 cluster4
RHOT2 cluster4

ICE1 cluster4
SMARCA5 cluster4
SHPRH cluster4
PPP4C cluster4
MIS18A cluster4
LUC7L3 cluster4
RAB10 cluster4
SFT2D1 cluster4
ZFYVE9 cluster4
ACTR10 cluster4
GOLGA4 cluster4
SNX2 cluster4
NUTF2 cluster4
HNRNPDL cluster4
SPEN cluster4
PIGT cluster4
ZSWIM7 cluster4
ASH1L cluster4
FBLN1 cluster4
HSPD1 cluster4
COMMD2 cluster4
LSM2 cluster4
KIAA2026 cluster4
RFC3 cluster4
NONO cluster4
H2AFZ cluster4
HERC2 cluster4
ZNF207 cluster4
MAGEA6 cluster4
OSBPL8 cluster4
OIP5 cluster4
AAMP cluster4
PDCD4 cluster4
FBXW7 cluster4
GNB1 cluster4
RP11-382A20.3 cluster4
YY1 cluster4
DOCK7 cluster4
TAF5 cluster4
MRPL20 cluster4
CDK4 cluster4
RNF212 cluster4
CBX3 cluster4
SMC2 cluster4
CRELD2 cluster4
CNIH1 cluster4
MAPK1IP1L cluster4
NGDN cluster4
FBXL3 cluster4
NHLRC3 cluster4
SEC61B cluster4
SRRM1 cluster4
CDCA8 cluster4
HNRNPA1 cluster4
TBCA cluster4
PRC1 cluster4

NAP1L1 cluster4
TRMT112 cluster4
ANKRD26 cluster4
MEAF6 cluster4
KHDRBS1 cluster4
RPL7L1 cluster4
PSMC6 cluster4
GOPC cluster4
CPSF6 cluster4
RFC2 cluster4
CDKN2D cluster4
VAMP3 cluster4
PRPF6 cluster4
PRMT5 cluster4
TUBGCP2 cluster4
SNRNP27 cluster4
PSMA3 cluster4
RALBP1 cluster4
WBP4 cluster4
TXNDC15 cluster4
BOLA3 cluster4
LSM5 cluster4
ARHGAP29 cluster4
TRIP11 cluster4
ATXN2 cluster4
SPDL1 cluster4
TMEM57 cluster4
CSTF3 cluster4
SUMO1 cluster4
RPS27L cluster4
GLG1 cluster4
PCOLCE2 cluster4
GCC2 cluster4
ZC3H15 cluster4
FRA10AC1 cluster4
SENP6 cluster4
MND1 cluster4
AC074117.10 cluster4
DNAJC7 cluster4
PDCD6 cluster4
TVP23B cluster4
KPNB1 cluster4
BAZ1B cluster4
FKBP1A cluster4
DGUOK cluster4
KRR1 cluster4
DYNC1LI1 cluster4
PSMA5 cluster4
MOB1A cluster4
POLR2J3 cluster4
RNMT cluster4
TIMM22 cluster4
HMGXB4 cluster4
LSM1 cluster4
TM2D3 cluster4
TMEM167A cluster4

NEMF cluster4
SRSF5 cluster4
AFG3L2 cluster4
TMEM18 cluster4
PPIL4 cluster4
MRPL13 cluster4
PRKDC cluster4
MRPS10 cluster4
CREBRF cluster4
CDK11A cluster4
MCMBP cluster4
RNF115 cluster4
SNRPD3 cluster4
HNRNPD cluster4
P4HB cluster4
YIPF5 cluster4
LRIF1 cluster4
DCTN1 cluster4
DCTN3 cluster4
TRMT10A cluster4
PPP1R12A cluster4
COX8A cluster4
TMCO1 cluster4
TMEM50A cluster4
AKAP9 cluster4
ABCE1 cluster4
XPO1 cluster4
CDCA4 cluster4
REV3L cluster4
MT-ND4L cluster4
MGME1 cluster4
CDK11B cluster4
POLR2H cluster4
ILKAP cluster4
CYB5B cluster4
RBAK-RBAKDN cluster4
SCAPER cluster4
MSL1 cluster4
POLR2B cluster4
FTH1 cluster4
ATG101 cluster4
GABPB1-AS1 cluster4
MESDC2 cluster4
EIF1AY cluster4
RAD1 cluster4
VAMP2 cluster4
DNAJC8 cluster4
ATF6B cluster4
RAB28 cluster4
FGD5-AS1 cluster4
PSMD10 cluster4
ALKBH5 cluster4
FAM49B cluster4
KMT2E cluster4
ILF3 cluster4
MNAT1 cluster4

PSMA2 cluster4
PHACTR4 cluster4
HSF2 cluster4
ATP6V1E1 cluster4
MAP7D2 cluster4
TMEM134 cluster4
DDX6 cluster4
BRD7 cluster4
PUF60 cluster4
HENMT1 cluster4
CDC37L1 cluster4
NCSTN cluster4
C5orf24 cluster4
IMPACT cluster4
IARS cluster4
TSEN15 cluster4
TCERG1 cluster4
HACD3 cluster4
EIF2B1 cluster4
CKS1B cluster4
MBIP cluster4
PHF5A cluster4
PRCC cluster4
CNOT7 cluster4
PHAX cluster4
ATP5C1 cluster4
ATP5A1 cluster4
BRWD1 cluster4
GCLM cluster4
GID8 cluster4
WSB1 cluster4
CENPE cluster4
CEP85 cluster4
BMS1 cluster4
RBM28 cluster4
SNRNP200 cluster4
SET cluster4
EZR cluster4
SMARCB1 cluster4
PRRC2C cluster4
6-Mar cluster4
OST4 cluster4
LEMD3 cluster4
PDS5B cluster4
WDR33 cluster4
NAP1L4 cluster4
C11orf31 cluster4
AIMP1 cluster4
COPB1 cluster4
MDH1 cluster4
METTL5 cluster4
MED28 cluster4
MYL12B cluster4
EIF5 cluster4
RBBP4 cluster4
RMDN3 cluster4

TIMM17B cluster4
MRFAP1L1 cluster4
RALGAPA1 cluster4
TAF2 cluster4
SFPQ cluster4
MDN1 cluster4
TMEM60 cluster4
RBM26 cluster4
ARF6 cluster4
ITSN1 cluster4
EIF4A1 cluster4
ZC3H6 cluster4
SRSF11 cluster4
SPCS2 cluster4
PDCD10 cluster4
MAP3K2 cluster4
GDI1 cluster4
BCLAF1 cluster4
DICER1 cluster4
LYRM9 cluster4
ABHD5 cluster4
SPATS2 cluster4
BDP1 cluster4
ZNF22 cluster4
DKC1 cluster4
GNAI3 cluster4
WNK3 cluster4
MGA cluster4
TIMMDC1 cluster4
STOML2 cluster4
RBM8A cluster4
STK4 cluster4
FYTTD1 cluster4
YTHDC1 cluster4
DLD cluster4
ZCCHC11 cluster4
PMVK cluster4
PRPSAP1 cluster4
ADD3 cluster4
HSP90AB1 cluster4
FUS cluster4
HAUS1 cluster4
CNOT6 cluster4
TAF1B cluster4
CD164 cluster4
FANCL cluster4
WHSC1L1 cluster4
RSL1D1 cluster4
WAPL cluster4
SMC5 cluster4
PPP4R2 cluster4
RMI2 cluster4
ZNF518A cluster4
FNIP1 cluster4
IST1 cluster4
RRP15 cluster4

MALT1 cluster4
MED19 cluster4
PYCR2 cluster4
VPS26A cluster4
BRIX1 cluster4
C1orf35 cluster4
EIF4A2 cluster4
SNRPA cluster4
CLINT1 cluster4
DMRTB1 cluster4
TRA2B cluster4
NOC3L cluster4
EIF4A3 cluster4
PHB2 cluster4
ATP5G3 cluster4
RUVBL1 cluster4
DYNC1H1 cluster4
KRAS cluster4
RAD50 cluster4
CDC42SE2 cluster4
CCNK cluster4
RPL22 cluster4
SF3B1 cluster4
SPNS1 cluster4
NDUFAF4 cluster4
FRG1 cluster4
KIF22 cluster4
TMEM107 cluster4
RRBP1 cluster4
SDF4 cluster4
SUPT16H cluster4
FAF2 cluster4
RB1CC1 cluster4
NSUN5 cluster4
SMNDC1 cluster4
SNX17 cluster4
CCT6A cluster4
TAF7 cluster4
PTBP2 cluster4
SIN3A cluster4
ARCN1 cluster4
STRAP cluster4
DAXX cluster4
NUP54 cluster4
RNF214 cluster4
PDIA4 cluster4
CAMTA1 cluster4
ATXN10 cluster4
CNOT3 cluster4
PPP3CB cluster4
MAGOH cluster4
NSL1 cluster4
PHF3 cluster4
BCL7B cluster4
MED4 cluster4
RAD54L2 cluster4

NOL11 cluster4
EID1 cluster4
SRSF1 cluster4
MAN1A2 cluster4
CSAD cluster4
PIGH cluster4
ATP2A2 cluster4
DCTN6 cluster4
TDRD9 cluster4
RBM23 cluster4
RIC3 cluster4
LARP4 cluster4
MED10 cluster4
WDR75 cluster4
PCNP cluster4
NUP188 cluster4
PRPF4B cluster4
ARMC8 cluster4
MZT1 cluster4
EIF3A cluster4
PRMT1 cluster4
UBQLN1 cluster4
ATF4 cluster4
NUDC cluster4
LSM8 cluster4
MRPS5 cluster4
ARF5 cluster4
DPF2 cluster4
DHX9 cluster4
RSF1 cluster4
POLR2C cluster4
HSPA9 cluster4
RICTOR cluster4
DCAF4L1 cluster4
STRN3 cluster4
SASS6 cluster4
CASC3 cluster4
SYF2 cluster4
UBN1 cluster4
SMIM20 cluster4
NCBP3 cluster4
MLF2 cluster4
SRSF4 cluster4
DTWD1 cluster4
CFL2 cluster4
PTCD3 cluster4
ABHD10 cluster4
LMAN1 cluster4
CELF1 cluster4
NUP93 cluster4
TMEM106B cluster4
GPBP1L1 cluster4
TMEM9B cluster4
PTP4A2 cluster4
ZNF830 cluster4
DDX17 cluster4

UBLCP1 cluster4
RPAP3 cluster4
LARP7 cluster4
NUBP1 cluster4
GAR1 cluster4
HNRNPH1 cluster4
SGOL1 cluster4
SAFB2 cluster4
BIRC6 cluster4
EXOSC7 cluster4
THYN1 cluster4
RPN1 cluster4
MRPL16 cluster4
AMD1 cluster4
RTCB cluster4
REV1 cluster4
TMEM208 cluster4
ZNF131 cluster4
POLR2E cluster4
SHOC2 cluster4
ARID1A cluster4
PWP1 cluster4
ADNP2 cluster4
NCOR1 cluster4
SCML2 cluster4
KAT6A cluster4
COPS8 cluster4
NUP37 cluster4
TIAL1 cluster4
FIP1L1 cluster4
ZNF302 cluster4
LUC7L2 cluster4
AZI2 cluster4
NAA35 cluster4
DHX15 cluster4
PUM3 cluster4
GTPBP4 cluster4
SEC63 cluster4
CWF19L2 cluster4
MINOS1 cluster4
ILF3-AS1 cluster4
CDC73 cluster4
ZNF281 cluster4
NCK1 cluster4
LTV1 cluster4
FAM96B cluster4
COQ7 cluster4
TOMM40 cluster4
HELZ cluster4
PET100 cluster4
ARL16 cluster4
INO80E cluster4
SCFD1 cluster4
RBM42 cluster4
TARDBP cluster4
ACADM cluster4

CBR4 cluster4
YLPM1 cluster4
PCBP1 cluster4
PPID cluster4
TDRD12 cluster4
SKIV2L2 cluster4
GON4L cluster4
FNBP4 cluster4
OARD1 cluster4
PRPF38B cluster4
SEC61G cluster4
SBNO1 cluster4
RBM46 cluster4
RPL34 Cluster5
RPL7 Cluster5
CWC15 Cluster5
MAEL Cluster5
POMP Cluster5
UQCRB Cluster5
GLRX5 Cluster5
CHMP5 Cluster5
DDX4 Cluster5
H3F3B Cluster5
PSMA4 Cluster5
COX5A Cluster5
DHX36 Cluster5
EMC3 Cluster5
SF3B5 Cluster5
CACUL1 Cluster5
NUDT21 Cluster5
ATG3 Cluster5
SAP18 Cluster5
H2AFV Cluster5
PPFIA1 Cluster5
SNU13 Cluster5
HNRNPF Cluster5
CCT2 Cluster5
UBXN4 Cluster5
VPS4B Cluster5
THOC3 Cluster5
DNAJB9 Cluster5
TUBA3D Cluster5
EIF2S2 Cluster5
SERBP1 Cluster5
GLRX3 Cluster5
SYCE3 Cluster5
COX6C Cluster5
VDAC3 Cluster5
ZRANB2 Cluster5
BRD8 Cluster5
SHFM1 Cluster5
GPN3 Cluster5
NSRP1 Cluster5
CCDC59 Cluster5
HSF2BP Cluster5
RAD23A Cluster5

SECISBP2 Cluster5
BTBD10 Cluster5
EMC7 Cluster5
HAX1 Cluster5
TTC1 Cluster5
KDELR2 Cluster5
KLHDC2 Cluster5
PRPF40A Cluster5
CEBPZOS Cluster5
SF3B6 Cluster5
ATRAID Cluster5
BUD31 Cluster5
PDCD5 Cluster5
TUBA3E Cluster5
DDX5 Cluster5
CCDC90B Cluster5
ACTL6A Cluster5
RSRC2 Cluster5
SPESP1 Cluster5
C1orf52 Cluster5
NDUFB5 Cluster5
RPS27A Cluster5
KRBOX1 Cluster5
PSMD11 Cluster5
TPRKB Cluster5
ENY2 Cluster5
EMC4 Cluster5
DYNC1I2 Cluster5
FAM204A Cluster5
MORF4L1 Cluster5
ANKLE2 Cluster5
NAPA Cluster5
NDUFC2 Cluster5
MANF Cluster5
DNAJC2 Cluster5
PCID2 Cluster5
HSBP1 Cluster5
PSMA1 Cluster5
PDE6D Cluster5
ZCRB1 Cluster5
TUBA3C Cluster5
RPL26L1 Cluster5
SIMC1 Cluster5
ATP5L Cluster5
NDUFA6 Cluster5
BUB3 Cluster5
C8orf59 Cluster5
EXOSC8 Cluster5
NKIRAS1 Cluster5
CSNK2B Cluster5
HIRIP3 Cluster5
WBP11 Cluster5
ACER3 Cluster5
WHSC1 Cluster5
EMC2 Cluster5
COX6A1 Cluster5

DYNLT1 Cluster5
YWHAQ Cluster5
ING2 Cluster5
UBE3A Cluster5
PPP6R2 Cluster5
FUCA1 Cluster5
LZIC Cluster5
CNOT1 Cluster5
CSNK1A1 Cluster5
NUP88 Cluster5
DEGS1 Cluster5
VPS37A Cluster5
CBX5 Cluster5
SEC22C Cluster5
CCDC171 Cluster5
NFU1 Cluster5
ATP6V1D Cluster5
NDUFC1 Cluster5
HINT1 Cluster5
FBXO25 Cluster5
TMEM170A Cluster5
PCM1 Cluster5
LLPH Cluster5
RAB11A Cluster5
VDAC2 Cluster5
ANAPC10 Cluster5
DSCR8 Cluster5
SNRPB2 Cluster5
RPL39L Cluster5
UBE2Q2 Cluster5
GPATCH11 Cluster5
UPF3A Cluster5
RWDD1 Cluster5
DDX50 Cluster5
DPM1 Cluster5
ATP5F1 Cluster5
TXNL1 Cluster5
ATXN2L Cluster5
CLIC4 Cluster5
LUC7L Cluster5
COPS3 Cluster5
15-Sep Cluster5
LSM3 Cluster5
INTS6 Cluster5
ARF4 Cluster5
ATP6V0D1 Cluster5
CPSF3 Cluster5
SUPT20H Cluster5
ZNF200 Cluster5
EWSR1 Cluster5
CYCS Cluster5
PSMD7 Cluster5
RANBP1 Cluster5
IFT20 Cluster5
RPA3 Cluster5
TM9SF2 Cluster5

GTF2H5 Cluster5
SAMD8 Cluster5
SPRYD7 Cluster5
XRN2 Cluster5
PCNA Cluster5
SSBP1 Cluster5
TOX4 Cluster5
ANKRD11 Cluster5
KTN1 Cluster5
GSPT1 Cluster5
C2orf69 Cluster5
COX7C Cluster5
COX11 Cluster5
MRPL33 Cluster5
PPP6C Cluster5
CHURC1 Cluster5
PSMB1 Cluster5
SAAL1 Cluster5
C5orf15 Cluster5
IMMT Cluster5
2-Sep Cluster5
TMEM5 Cluster5
ELF2 Cluster5
EXOSC9 Cluster5
MTCH2 Cluster5
PLPP5 Cluster5
KHSRP Cluster5
UAP1 Cluster5
FAM122A Cluster5
EFCAB7 Cluster5
C11orf73 Cluster5
STK17A Cluster5
UBE2L3 Cluster5
IP6K2 Cluster5
DAD1 Cluster5
R3HCC1L Cluster5
UQCRFS1 Cluster5
GAPVD1 Cluster5
MYEOV2 Cluster5
NDUFAB1 Cluster5
DDX10 Cluster5
C11orf70 Cluster5
CTNNBIP1 Cluster5
RAB30-AS1 Cluster5
ITGB3BP Cluster5
EXOSC3 Cluster5
C12orf29 Cluster5
MRPS11 Cluster5
NSMCE2 Cluster5
TM2D2 Cluster5
CHCHD7 Cluster5
MICU2 Cluster5
KIF23 Cluster5
ZNF7 Cluster5
SLC30A1 Cluster5
OOEP Cluster5

MMADHC Cluster5
MRPS16 Cluster5
GLCCI1 Cluster5
TMEM230 Cluster5
MTERF4 Cluster5
UGP2 Cluster5
USP42 Cluster5
SURF4 Cluster5
RANBP3 Cluster5
ATG4C Cluster5
PDHB Cluster5
WASH1 Cluster5
CRBN Cluster5
NDUFB3 Cluster5
SYNCRIP Cluster5
UBE2V2 Cluster5
PDHX Cluster5
CEP135 Cluster5
MAPK1 Cluster5
PAPOLA Cluster5
TIMM23 Cluster5
SRI Cluster5
REEP5 Cluster5
ZNF143 Cluster5
COA5 Cluster5
PSMD14 Cluster5
TRMT13 Cluster5
NUDCD1 Cluster5
ODF2L Cluster5
GTF3C6 Cluster5
SSR3 Cluster5
TRMT10C Cluster5
THOC1 Cluster5
SDHA Cluster5
GFPT1 Cluster5
BNIP3L Cluster5
UBE2S Cluster5
ORC3 Cluster5
HSPA4 Cluster5
CLUAP1 Cluster5
FARSB Cluster5
TRIM28 Cluster5
GOSR1 Cluster5
SEC22A Cluster5
NR2C1 Cluster5
SRP19 Cluster5
SLC25A33 Cluster5
ZC2HC1A Cluster5
NTPCR Cluster5
NDUFB1 Cluster5
ORC2 Cluster5
TRIM37 Cluster5
NOP58 Cluster5
MRPL52 Cluster5
ZNHIT3 Cluster5
TSN Cluster5

GGNBP2 Cluster5
AKIP1 Cluster5
COPS2 Cluster5
ARF1 Cluster5
UFM1 Cluster5
MRPL3 Cluster5
RAE1 Cluster5
CCT5 Cluster5
QRICH1 Cluster5
PSMD2 Cluster5
TRAM1 Cluster5
DNTTIP1 Cluster5
ORMDL2 Cluster5
TCP1 Cluster5
PSMA3-AS1 Cluster5
DNAJC3 Cluster5
EIF6 Cluster5
RRAGA Cluster5
NUDCD2 Cluster5
PRELID3B Cluster5
TRIP12 Cluster5
GLT8D1 Cluster5
MLH1 Cluster5
TMEM263 Cluster5
SCAMP1 Cluster5
MRPS33 Cluster5
PAICS Cluster5
HADHB Cluster5
ARMC1 Cluster5
PPP1R2 Cluster5
PTPRN2 Cluster5
RFK Cluster5
TRMT1L Cluster5
RPAIN Cluster5
SUGT1 Cluster5
ABI2 Cluster5
POLI Cluster5
UBE2D2 Cluster5
ZC3H18 Cluster5
PAIP1 Cluster5
PSMB3 Cluster5
UTP18 Cluster5
DNTTIP2 Cluster5
NDUFA5 Cluster5
PGM3 Cluster5
SVBP Cluster5
RSRC1 Cluster5
INTS8 Cluster5
EPRS Cluster5
KIF3A Cluster5
PFDN4 Cluster5
CNPY3 Cluster5
CEP55 Cluster5
TSSC4 Cluster5
ACBD6 Cluster5
DERL1 Cluster5

EZH2 Cluster5
DNAJC10 Cluster5
MRPS22 Cluster5
UBE2J2 Cluster5
DR1 Cluster5
AHSA1 Cluster5
ELAVL1 Cluster5
ZFAND6 Cluster5
ACSL3 Cluster5
ANAPC16 Cluster5
DNAJC21 Cluster5
ERLEC1 Cluster5
PMPCB Cluster5
EMC6 Cluster5
TRAPPC3 Cluster5
ZC3H11A Cluster5
RIOK1 Cluster5
MED21 Cluster5
IFT43 Cluster5
SMC6 Cluster5
PABPC1 Cluster5
NDUFA8 Cluster5
KPNA2 Cluster5
PA2G4 Cluster5
MRPL51 Cluster5
SLIRP Cluster5
RPL36AL Cluster5
SNRPC Cluster5
CENPW Cluster5
DNAJA1 Cluster5
CIR1 Cluster5
MRPL14 Cluster5
ESD Cluster5
LZTFL1 Cluster5
PSMG2 Cluster5
CEBPZ Cluster5
DNAJC15 Cluster5
IK Cluster5
SYPL1 Cluster5
SERF2 Cluster5
CCT7 Cluster5
METTL23 Cluster5
RTN4 Cluster5
RAB2A Cluster5
CDC20 Cluster5
USP8 Cluster5
C14orf166 Cluster5
DCAF7 Cluster5
CCDC181 Cluster5
DERL2 Cluster5
MRPL43 Cluster5
ATP5B Cluster5
RPS7 Cluster5
CCT4 Cluster5
PSMB6 Cluster5
SDHB Cluster5

KRTCAP2 Cluster5
NSA2 Cluster5
KRT10 Cluster5
CENPF Cluster5
PSMB7 Cluster5
SNRPA1 Cluster5
UCHL3 Cluster5
UFSP2 Cluster5
PEX13 Cluster5
NOL7 Cluster5
RER1 Cluster5
COMMD8 Cluster5
RPL5 Cluster5
HNRNPLL Cluster5
NPHP1 Cluster5
CCDC34 Cluster5
TFIP11 Cluster5
WDR5 Cluster5
SETD3 Cluster5
CDK7 Cluster5
LRRC59 Cluster5
CCNB1 Cluster5
RPL23A Cluster5
TCEB1 Cluster5
CD59 Cluster5
RP11-390E23.6 Cluster5
RPL35A Cluster5
SLU7 Cluster5
MRPS9 Cluster5
CGRRF1 Cluster5
MIEN1 Cluster5
NDUFS6 Cluster5
HAGH Cluster5
RPF2 Cluster5
EIF3E Cluster5
SF3B2 Cluster5
CARD19 Cluster5
CEP19 Cluster5
RPS15A Cluster5
ARL14EP Cluster5
CFAP97 Cluster5
RPS20 Cluster5
PHTF1 Cluster5
RPL24 Cluster5
NDUFAF2 Cluster5
ATP5J Cluster5
TTC26 Cluster5
TTK Cluster5
SNX3 Cluster5
PPHLN1 Cluster5
MAF1 Cluster5
GMNN Cluster5
NAA30 Cluster5
COPS4 Cluster5
ERO1B Cluster5
CDK5RAP3 Cluster5

ZNF839 Cluster5
NME7 Cluster5
MRPL46 Cluster5
BTF3 Cluster5
MRPL22 Cluster5
TBRG1 Cluster5
CSTF1 Cluster5
OXSM Cluster5
PSMC2 Cluster5
NDUFAF1 Cluster5
KIAA1524 Cluster5
UBE2K Cluster5
GPATCH2 Cluster5
BTF3L4 Cluster5
APITD1 Cluster5
CHCHD1 Cluster5
ZNF473 Cluster5
RPF1 Cluster5
SETD4 Cluster5
MRPS18C Cluster5
PPIL3 Cluster5
PSMC3 Cluster5
SPAG9 Cluster5
TTI2 Cluster5
ELP4 Cluster5
PKNOX1 Cluster5
TOMM7 Cluster5
PHF20L1 Cluster5
GAA Cluster5
MRPL54 Cluster5
CHCHD6 Cluster5
DHRS7B Cluster5
RPS25 Cluster5
PANK2 Cluster5
TRAPPC2L Cluster5
C20orf96 Cluster5
MRPS31 Cluster5
EFCAB14 Cluster5
SSSCA1 Cluster5
PSMD6 Cluster5
SF3B4 Cluster5
NACA Cluster5
NFE2L2 Cluster5
RNF126 Cluster5
TFG Cluster5
GTPBP8 Cluster5
DRC3 Cluster5
VPS28 Cluster5
FASTKD2 Cluster5
TACC3 Cluster5
RARS2 Cluster5
FKBP4 Cluster5
ITGAE Cluster5
JKAMP Cluster5
CLDND1 Cluster5
EIF3H Cluster5

HES6 Cluster5
CDR2 Cluster5
CSRP2 Cluster5
PSEN1 Cluster5
IFT52 Cluster5
YAE1D1 Cluster5
EIF3D Cluster5
CREBL2 Cluster5
RSL24D1 Cluster5
SUCLG1 Cluster5
UHRF1BP1L Cluster5
DPY30 Cluster5
TBCC Cluster5
TXNDC9 Cluster5
LINC00493 Cluster5
DDX39A Cluster5
PSTK Cluster5
AGGF1 Cluster5
STYX Cluster5
LYRM2 Cluster5
PPM1D Cluster5
EIF3M Cluster5
SPSB2 Cluster5
CEP83 Cluster5
CNOT2 Cluster5
DESI2 Cluster5
OSBPL2 Cluster5
CLIC1 Cluster5
CEP63 Cluster5
FAM162A Cluster5
CDCA2 Cluster5
IQCK Cluster5
ATP5J2 Cluster5
ARPC2 Cluster5
RPL30 Cluster5
TMEM183A Cluster5
EXTL2 Cluster5
NDUFS7 Cluster5
RANBP9 Cluster5
MRPL2 Cluster5
VPS8 Cluster5
FAM160A2 Cluster5
DHX30 Cluster5
PNMA1 Cluster5
ERLIN1 Cluster5
C12orf76 Cluster5
LYPLAL1 Cluster5
HMGN3 Cluster5
PLRG1 Cluster5
COA4 Cluster5
CCDC174 Cluster5
ATP6V1B2 Cluster5
DHX16 Cluster5
EXOC1 Cluster5
PNRC1 Cluster5
ZNF277 Cluster5

CTB-50L17.10 Cluster5
HAUS2 Cluster5
WAC-AS1 Cluster5
CSDE1 Cluster5
NDUFS3 Cluster5
DYNC1LI2 Cluster5
PSMB5 Cluster5
NDUFA12 Cluster5
MYNN Cluster5
LEPROTL1 Cluster5
RMDN1 Cluster5
CWC25 Cluster5
RPP30 Cluster5
POLR3F Cluster5
EBNA1BP2 Cluster5
ALDH9A1 Cluster5
UTP11L Cluster5
MRPS14 Cluster5
GOLGA8B Cluster5
TMEM126A Cluster5
ATF7IP Cluster5
PRRC2A Cluster5
AC006277.2 Cluster5
TBRG4 Cluster5
CCDC130 Cluster5
RFC1 Cluster5
TRIOBP Cluster5
UBE2Q2L Cluster5
CNOT10 Cluster5
MRPL17 Cluster5
TEX9 Cluster5
MRPS36 Cluster5
CCDC117 Cluster5
GTF2H1 Cluster5
PLEKHB2 Cluster5
WDR61 Cluster5
MCPH1 Cluster5
IMMP1L Cluster5
REXO2 Cluster5
CEBPG Cluster5
TMEM70 Cluster5
TIMM17A Cluster5
CHMP2A Cluster5
NDUFA2 Cluster5
C14orf119 Cluster5
SSNA1 Cluster5
DPH3 Cluster5
MCM4 Cluster5
AP2A2 Cluster5
CACYBP Cluster5
TNPO1 Cluster5
ATP5H Cluster5
MRPL21 Cluster5
EIF2B2 Cluster5
AHI1 Cluster5
SF1 Cluster5

RNF10 Cluster5
C2orf49 Cluster5
SUPT4H1 Cluster5
KIF2A Cluster5
HIGD2A Cluster5
NDUFV2 Cluster5
PIGF Cluster5
STX18 Cluster5
USP5 Cluster5
PEBP1 Cluster5
RTFDC1 Cluster5
BCAS2 Cluster5
NCBP2 Cluster5
ASH2L Cluster5
PRKRIP1 Cluster5
MTDH Cluster5
KARS Cluster5
RNF149 Cluster5
TMEM248 Cluster5
EIF4G3 Cluster5
STT3B Cluster5
RPL23 Cluster5
RNH1 Cluster5
RPS14 Cluster5
DCAF13 Cluster5
ECH1 Cluster5
CRIPT Cluster5
MAP9 Cluster5
GNL2 Cluster5
POLD3 Cluster5
HSPH1 Cluster5
DIDO1 Cluster5
C2orf76 Cluster5
ASB1 Cluster5
ACIN1 Cluster5
NDUFA7 Cluster5
SRPRB Cluster5
RYBP Cluster5
CCDC25 Cluster5
WDR18 Cluster5
NMT1 Cluster5
RNGTT Cluster5
MARS Cluster5
HSDL1 Cluster5
PNPLA2 Cluster5
POLR3GL Cluster5
GOLGA8A Cluster5
MALSU1 Cluster5
ADSS Cluster5
ZBTB8OS Cluster5
DAP3 Cluster5
HBS1L Cluster5
SMU1 Cluster5
DNAJA3 Cluster5
POLR2M Cluster5
C6orf62 Cluster5

NUDT9 Cluster5
SART3 Cluster5
ZNF24 Cluster5
TTC17 Cluster5
HARS Cluster5
ZNHIT1 Cluster5
DDX27 Cluster5
NEK3 Cluster5
C11orf57 Cluster5
KIAA1328 Cluster5
UBE2A Cluster5
KRCC1 Cluster5
CCNL1 Cluster5
FLII Cluster5
CDK5RAP2 Cluster5
PCNXL4 Cluster5
WAC Cluster5
SARS Cluster5
PIAS1 Cluster5
NEK1 Cluster5
RPL9 Cluster5
KAT5 Cluster5
NR1H2 Cluster5
UQCRC2 Cluster5
RUBCN Cluster5
CSNK2A1 Cluster5
SAV1 Cluster5
ECHDC1 Cluster5
SNRNP35 Cluster5
PDCD11 Cluster5
PLAA Cluster5
MRPL9 Cluster5
FAM222B Cluster5
GPR137 cluster6
BCAP29 cluster6
PHF1 cluster6
CCDC88A cluster6
HSPA2 cluster6
CETN3 cluster6
RP11-620J15.3 cluster6
IFT88 cluster6
C15orf48 cluster6
UNC50 cluster6
CUTC cluster6
SGOL2 cluster6
CNTD1 cluster6
C15orf43 cluster6
EID3 cluster6
SSR2 cluster6
SPATA22 cluster6
SUGP2 cluster6
CKLF cluster6
AC240274.1 cluster6
METTL10 cluster6
BOLL cluster6
NUF2 cluster6

PEX3 cluster6
LMNA cluster6
CCDC36 cluster6
ART3 cluster6
AC159540.1 cluster6
CEP57L1 cluster6
LACTB cluster6
DHFR cluster6
CCDC14 cluster6
THOC7 cluster6
DEPDC1B cluster6
PLPP1 cluster6
SPDYA cluster6
TXNRD3 cluster6
RSBN1 cluster6
NCAPH cluster6
ANKRD36C cluster6
C4orf32 cluster6
CATSPER2 cluster6
GULP1 cluster6
FAM216A cluster6
WIPI2 cluster6
RAD17 cluster6
ANKRD36 cluster6
DEPDC1 cluster6
LRRC49 cluster6
AK9 cluster6
ANKRD62 cluster6
MPP6 cluster6
MGAT2 cluster6
SOS2 cluster6
BUD13 cluster6
SNAPC1 cluster6
TNPO3 cluster6
PYURF cluster6
PFDN6 cluster6
TOPBP1 cluster6
PCSK7 cluster6
XRRA1 cluster6
TCEB3 cluster6
KDM5B cluster6
RDH14 cluster6
AHCTF1 cluster6
RWDD2B cluster6
MKKS cluster6
ALG8 cluster6
TBC1D12 cluster6
EAPP cluster6
FKBP6 cluster6
ACD cluster6
HLA-A cluster6
FAM107B cluster6
CDK1 cluster6
WDR70 cluster6
MLLT10 cluster6
SLC4A7 cluster6

CCNO cluster6
RNFT1 cluster6
KIAA1109 cluster6
ANKRD36B cluster6
ZNF544 cluster6
CDCA3 cluster6
FAM207A cluster6
CYB5D2 cluster6
ATRNL1 cluster6
GGA1 cluster6
GOLGA1 cluster6
IMPA1 cluster6
BIRC5 cluster6
NSMCE3 cluster6
CENPT cluster6
GTSE1 cluster6
AK2 cluster6
PDRG1 cluster6
TANK cluster6
MBD1 cluster6
MYO9A cluster6
DYNC2H1 cluster6
C10orf67 cluster6
NEK4 cluster6
WRB cluster6
EIF5A cluster6
SNX14 cluster6
C21orf59 cluster6
IFT74 cluster6
GLB1L cluster6
KCTD19 cluster6
ARV1 cluster6
PLA2G16 cluster6
PENK cluster6
NEK2 cluster6
WDR87 cluster6
IFT27 cluster6
OVOS2 cluster6
RBMXL2 cluster6
SFMBT1 cluster6
PIWIL1 cluster6
TCFL5 cluster6
COX6B2 cluster6
FAM174A cluster6
ADAD2 cluster6
TBP cluster6
USP32 cluster6
TXNDC12 cluster6
ZNF793-AS1 cluster6
TCF25 cluster6
TCEA2 cluster6
TYMS cluster6
POP4 cluster6
SMDT1 cluster6
STK33 cluster6
LRRC23 cluster6

ENO1-AS1 cluster6
DNAJC27 cluster6
REEP4 cluster6
CHAC2 cluster6
ARL3 cluster6
TMEM261 cluster6
PAF1 cluster6
NBPF14 cluster6
RP13-942N8.1 cluster6
TIMM9 cluster6
PMM1 cluster6
BTBD1 cluster6
NT5C3B cluster6
TBK1 cluster6
CYLD cluster6
FAM184A cluster6
SAC3D1 cluster6
PFKM cluster6
ANAPC11 cluster6
TOE1 cluster6
PLIN3 cluster6
AC005076.5 cluster6
FAM134A cluster6
RP11-293A21.1 cluster6
UBR5 cluster6
FAAH cluster6
WDR54 cluster6
CEP104 cluster6
AQP11 cluster6
EFCAB2 cluster6
MTL5 cluster6
MRPL28 cluster6
C9orf43 cluster6
3-Mar cluster6
PINK1 cluster6
SNHG8 cluster6
WDR66 cluster6
DNAJC5G cluster6
EIF5AL1 cluster6
CCHCR1 cluster6
ZBED5-AS1 cluster6
DNAJB2 cluster6
CD320 cluster6
CCDC112 cluster6
COMMD4 cluster6
EIF3K cluster6
ATP5G1 cluster6
SETX cluster6
TP53I13 cluster6
ZBTB44 cluster6
ARL6IP1 cluster6
CGGBP1 cluster6
WDR48 cluster6
TTC14 cluster6
C4orf3 cluster6
EFR3A cluster6

POLR2D cluster6
DYX1C1 cluster6
HDDC2 cluster6
CCDC24 cluster6
EEF1E1 cluster6
FAM221A cluster6
PIH1D1 cluster6
PAAF1 cluster6
MDM1 cluster6
CUEDC2 cluster6
CEP126 cluster6
C2orf68 cluster6
FAM118B cluster6
MBTD1 cluster6
PTTG1IP cluster6
CDCA5 cluster6
TIMM10 cluster6
TDRG1 cluster6
RP11-390F4.3 cluster6
TMEM99 cluster6
SLC25A31 cluster6
LINC01206 cluster6
STMN1 cluster6
SLTM cluster6
LDHAL6B cluster6
LINC01120 cluster6
LINC00668 cluster6
BUB1 cluster6
RFX4 cluster6
ANKRD30BL cluster6
STK31 cluster6
ZWILCH cluster6
STRBP cluster6
LINC00865 cluster6
PPP1R35 cluster6
EP400NL cluster6
CTC-338M12.5 cluster6
SLC4A8 cluster6
MLH3 cluster6
HOXB-AS1 cluster6
DDIT3 cluster6
CTD-2015H6.3 cluster6
FBXO43 cluster6
AC005082.12 cluster6
SLC2A14 cluster6
C3orf62 cluster6
KB-1592A4.15 cluster6
ANKRD30A cluster6
TDRD10 cluster6
RP13-735L24.1 cluster6
ACYP1 cluster6
DAZAP1 cluster6
LINC00837 cluster6
ZSCAN16-AS1 cluster6
LINC01481.1 cluster6
C12orf40 cluster6

RNF17 cluster6
FAM225A cluster6
TCP10 cluster6
PILRB cluster6
C4orf46 cluster6
IFT122 cluster6
FAM225B cluster6
CTD-2036P10.3 cluster6
CTC-523E23.1 cluster6
TEX14 cluster6
RP11-403N16.3 cluster6
MYADM cluster6
CNTROB cluster6
LINC00930 cluster6
PLK2 cluster6
IL23A cluster6
CFAP46 cluster6
RP3-468B3.2 cluster6
TCP10L2 cluster6
RP11-482D24.2 cluster6
SMURF1 cluster6
COL20A1 cluster6
CCDC191 cluster6
IL5 cluster6
PAN2 cluster6
RP11-844P9.2 cluster6
WDR35 cluster6
LINC01015 cluster6
SPRTN cluster6
ZNF546 cluster6
NEU3 cluster6
ANKRD18A cluster6
POTEC cluster6
TSTD1 cluster6
PTAR1 cluster6
PRNP cluster6
ENTHD1 cluster6
TDRD15 cluster6
RASSF1 cluster6
RP11-219A15.1 cluster6
RALGPS2 cluster6
IQCH-AS1 cluster6
DIXDC1 cluster6
RPGRIP1L cluster6
MAGEF1 cluster6
RP11-325N19.3 cluster6
KAT7 cluster6
ENKD1 cluster6
LINC00657 cluster6
WDR62 cluster6
PLD6 cluster6
BCKDK cluster6
PMS2 cluster6
C9orf40 cluster6
FAM214A cluster6
POLG2 cluster6

SGK1 cluster6
ZNF609 cluster6
CTD-2270L9.4 cluster6
AC023491.2 cluster6
RP11-363J20.1 cluster6
RFESD cluster6
HYLS1 cluster6
ANKRD20A4 cluster6
FAM95B1 cluster6
INSIG2 cluster6
LINC01006 cluster6
AC002117.1 cluster6
C1GALT1C1L cluster6
LCORL cluster6
ARMT1 cluster6
LINC00342 cluster6
AC008088.4 cluster6
HOXB-AS3 cluster6
LINC00964 cluster6
EXD2 cluster6
SCYL1 cluster6
RABAC1 cluster6
LEO1 cluster6
VPS13B cluster6
FLOT1 cluster6
RP11-181C3.1 cluster6
ST13 cluster6
ARHGAP33 cluster6
SEC61A2 cluster6
PRKAB1 cluster6
CEP44 cluster6
CARF cluster6
TNRC6C cluster6
TMEM67 cluster6
MKL2 cluster6
KDM4B cluster6
SPEF1 cluster6
MTFR2 cluster6
MRPL19 cluster6
KDM7A cluster6
CCDC144NL cluster6
HECTD4 cluster6
ZCCHC14 cluster6
SAMD13 cluster6
DAP cluster6
MIB1 cluster6
U2AF1L4 cluster6
ELP6 cluster6
PPP1R9A cluster6
FAM206A cluster6
BRD1 cluster6
RP11-152N13.5 cluster6
ANKRD20A3 cluster6
PPM1K cluster6
TK2 cluster6
RNF103 cluster6

RP11-714M23.2 cluster6
MYLIP cluster6
RNF6 cluster6
MTMR6 cluster6
FAM179B cluster6
DMTF1 cluster6
THOP1 cluster6
MSRB1 cluster6
FAM135A cluster6
PIGV cluster6
TEX261 cluster6
SEPSECS-AS1 cluster6
ARL6 cluster6
KANSL1L cluster6
CNOT11 cluster6
LINC00116 cluster6
AP4S1 cluster6
SHE cluster6
ATP8B3 cluster6
ELOVL2 cluster6
CAAP1 cluster6
NISCH cluster6
FOXD3-AS1 cluster6
POMT1 cluster6
RSPH1 cluster6
ARL13B cluster6
ACBD5 cluster6
PNPT1 cluster6
WDR19 cluster6
TDRD5 cluster6
SYNGR1 cluster6
BAD cluster6
NFS1 cluster6
ZNF608 cluster6
CEP295 cluster6
CCNYL1 cluster6
ZNF76 cluster6
RP5-1073O3.2 cluster6
CSRNP2 cluster6
SNAPC3 cluster6
ULK2 cluster6
CPSF1 cluster6
AC009133.12 cluster6
TOMM70A cluster6
FBXO3 cluster6
PRR19 cluster6
ERBB2IP cluster6
FBXO15 cluster6
TKFC cluster6
CRY2 cluster6
ANKRD18B cluster6
LINC01606 cluster6
WWC2 cluster6
MCAT cluster6
PEX7 cluster6
RING1 cluster6

IFT80 cluster6
FAM217B cluster6
PIGBOS1 cluster6
EBPL cluster6
DCTN2 cluster6
DCAF16 cluster6
JMJD6 cluster6
PPP1R10 cluster6
KPNA4 cluster6
DUSP28 cluster6
CALCOCO1 cluster6
DHRS11 cluster6
EIF2B3 cluster6
LENG8 cluster6
TDRD3 cluster6
YY1AP1 cluster6
RALGPS1 cluster6
SYNRG cluster6
HMG20B cluster6
DPP8 cluster6
RASSF8-AS1 cluster6
ACTR1A cluster6
EFEMP2 cluster6
BRE cluster6
FBRS cluster6
FAAP20 cluster6
HACL1 cluster6
SERINC3 cluster6
AKR7A2 cluster6
AP1M1 cluster6
TLK2 cluster6
OXR1 cluster6
FAM118A cluster6
GNL1 cluster6
HSPBP1 cluster6
NPEPPS cluster6
NUTM2A-AS1 cluster6
MRGBP cluster7
C1orf61 cluster7
RPL7A cluster7
RPL31 cluster7
PPP1CC cluster7
PFKP cluster7
EEF2 cluster7
MFI2-AS1 cluster7
PSMC1 cluster7
ITFG1-AS1 cluster7
PLEKHO1 cluster7
YBX1 cluster7
COX7A2 cluster7
SPATA4 cluster7
DBI cluster7
YBX3 cluster7
RNF114 cluster7
GK2 cluster7
AC005754.7 cluster7

12-Sep cluster7
ANKRD7 cluster7
HRASLS cluster7
RGMB-AS1 cluster7
PFN4 cluster7
ATPIF1 cluster7
BRDT cluster7
CETN1 cluster7
TMIGD3 cluster7
CH17-140K24.2 cluster7
RP11-1223D19.1 cluster7
NDUFB4 cluster7
CT62 cluster7
REEP3 cluster7
LRWD1 cluster7
ACAT1 cluster7
APH1B cluster7
GKAP1 cluster7
RPL10L cluster7
TRIM69 cluster7
CH17-140K24.7 cluster7
SPATA17 cluster7
EFCAB10 cluster7
C7orf62 cluster7
TTC25 cluster7
ACR cluster7
PDHA2 cluster7
PIH1D2 cluster7
LCA5L cluster7
C1orf56 cluster7
AHRR cluster7
NACA2 cluster7
ANKRD55 cluster7
XRCC6BP1 cluster7
MFSD6L cluster7
C6orf99 cluster7
CCDC74B cluster7
H3F3C cluster7
WDR53 cluster7
RPGRIP1 cluster7
CCDC74A cluster7
TRIM13 cluster7
RNF181 cluster7
SCCPDH cluster7
LARP6 cluster7
DDX19B cluster7
RSPH9 cluster7
CFAP206 cluster7
ADAM32 cluster7
ALS2CR11 cluster7
TP53RK cluster7
NME5 cluster7
CFAP53 cluster7
OSCP1 cluster7
C8orf88 cluster7
CHKA cluster7

PPP2R3C cluster7
HSF1 cluster7
KLHDC9 cluster7
MTIF3 cluster7
MTO1 cluster7
TSGA10 cluster7
HCFC2 cluster7
PPP2R1B cluster7
AMN1 cluster7
IQUB cluster7
AC062017.1 cluster7
SHCBP1L cluster7
KIF9 cluster7
RBAKDN cluster7
NRIP3 cluster7
ZPBP cluster7
CCDC173 cluster7
KHDRBS3 cluster7
BAZ2B cluster7
MRPL36 cluster7
CAPRIN1 cluster7
C1orf94 cluster7
KATNAL1 cluster7
DUS2 cluster7
FGFR1OP cluster7
PRKAR1A cluster7
MED7 cluster7
WDR60 cluster7
CDC25C cluster7
ZPBP2 cluster7
WARS cluster7
RP5-1023B21.1 cluster7
LDHC cluster7
CCDC110 cluster7
PTTG1 cluster7
C7orf55 cluster7
MCHR2-AS1 cluster7
TBPL1 cluster7
MNS1 cluster7
PSMG1 cluster7
LINC01016 cluster7
CCT6B cluster7
DBF4 cluster7
PPP3R2 cluster7
CCNB2 cluster7
NKAPL cluster7
AC004381.6 cluster7
PRKCDBP cluster7
GLIPR1L1 cluster7
GS1-259H13.2 cluster7
CCDC146 cluster7
PBK cluster7
NAA20 cluster7
SPINK2 cluster7
LYRM5 cluster7
GTF2A2 cluster7

SPAG6 cluster7
DYDC1 cluster7
SPATA16 cluster7
CCNA1 cluster7
CCDC62 cluster7
COX7B2 cluster7
MRPL47 cluster7
CCNH cluster7
RP5-942I16.1 cluster7
GLIPR1L2 cluster7
DDX25 cluster7
C6orf48 cluster7
SH3GLB1 cluster7
GOT1 cluster7
TCTE3 cluster7
YBEY cluster7
DYRK3 cluster7
PABPC3 cluster7
RP4-665J23.1 cluster7
OBSL1 cluster7
HN1 cluster7
DRG1 cluster7
GALNT3 cluster7
NUDT4 cluster7
CHST13 cluster7
CCDC65 cluster7
DNAL1 cluster7
MKRN1 cluster7
ZC2HC1C cluster7
MDH1B cluster7
LRRC34 cluster7
LYAR cluster7
SLCO6A1 cluster7
ZMYND10 cluster7
LRRC6 cluster7
ZNF846 cluster7
CCDC83 cluster7
C1orf158 cluster7
TMBIM4 cluster7
NUB1 cluster7
C16orf86 cluster7
LACTB2 cluster7
C15orf61 cluster7
COPRS cluster7
LRRIQ3 cluster7
INO80C cluster7
C14orf1 cluster7
CCDC42 cluster7
MRPL42 cluster7
RP11-705C15.4 cluster7
RIBC2 cluster7
LINC01210 cluster7
RP6-91H8.2 cluster7
CLGN cluster7
CHST1 cluster7
NDUFAF3 cluster7

KPNA5 cluster7
ADAM30 cluster7
GYG1 cluster7
ASRGL1 cluster7
BBOF1 cluster7
ATP6V1F cluster7
PPP3CC cluster7
MPST cluster7
RGCC cluster7
C4orf22 cluster7
NDUFAF7 cluster7
TMEM225 cluster7
PSMD4 cluster7
ISOC2 cluster7
LRR1 cluster7
SLC9B1 cluster7
CD47 cluster7
BBIP1 cluster7
PLK1 cluster7
RP11-231D20.2 cluster7
C9orf135-AS1 cluster7
CRNDE cluster7
BBS12 cluster7
AURKC cluster7
LRRC46 cluster7
ZMYND12 cluster7
BAG2 cluster7
LNP1 cluster7
CTC-338M12.2 cluster7
DECR1 cluster7
DNAJB1 cluster7
AC002467.7 cluster7
KDM4D cluster7
PIP5K1B cluster7
PPP1R42 cluster7
PSENEN cluster7
RP11-80F22.15 cluster7
RSPH6A cluster7
SNUPN cluster7
PRPS1L1 cluster7
ARHGAP9 cluster7
TMPRSS12 cluster7
GMPS cluster7
C22orf23 cluster7
LINC01096 cluster7
C1QTNF9B-AS1 cluster7
TMEM232 cluster7
BRAP cluster7
ARHGAP15 cluster7
LAP3 cluster7
ACSL6 cluster7
CDKL2 cluster7
C16orf95 cluster7
DPCD cluster7
CCDC38 cluster7
CTD-2538C1.2 cluster7

TOMM20 cluster7
SLC26A8 cluster7
PPP1R1C cluster7
RP13-270P17.1 cluster7
DNAAF1 cluster7
LDHA cluster7
UQCR10 cluster7
ZCWPW2 cluster7
ATG4D cluster7
IL4I1 cluster7
RUVBL2 cluster7
AURKA cluster7
RP11-180O5.2 cluster7
CARTPT cluster7
REC114 cluster7
RTF1 cluster7
LINC01249 cluster7
TBL2 cluster7
ZNF596 cluster7
AC010127.3 cluster7
TRIM61 cluster7
MIR646HG cluster7
SOX18 cluster7
FAM228A cluster7
SMIM19 cluster7
FAM170A cluster7
OTUD6B-AS1 cluster7
CISD1 cluster7
SPAG1 cluster7
AC007391.2 cluster7
KATNAL2 cluster7
RPS13 cluster7
TMEM220 cluster7
ELP5 cluster7
C10orf107 cluster7
CTD-3187F8.11 cluster7
HDGFL1 cluster7
SLC39A3 cluster7
LRRCC1 cluster7
GNPNAT1 cluster7
ENKUR cluster7
SLC6A16 cluster7
SAYSD1 cluster7
MDH2 cluster7
PALLD cluster7
TUSC1 cluster7
LVCAT1 cluster7
GS1-18A18.2 cluster7
PSMC4 cluster7
ACTR6 cluster7
ZNF233 cluster7
LINC01568 cluster7
NAGK cluster7
EPB41L4A-AS1 cluster7
PKD2L2 cluster7
TKTL2 cluster7

TTC30A cluster7
LINC01486 cluster7
ANKRD45 cluster7
DPM3 cluster7
YIPF2 cluster7
ALDH1A2 cluster7
GTSF1 cluster7
MEIG1 cluster7
PCDH17 cluster7
C11orf74 cluster7
SOX5 cluster7
RP11-253M7.1 cluster7
PXYLP1 cluster7
RNF32 cluster7
THUMPD3 cluster7
G0S2 cluster7
AMDHD2 cluster7
FAR2 cluster7
MRPL34 cluster7
LIN7A cluster7
RNF113B cluster7
STAM2 cluster7
AC022007.5 cluster7
ADAM18 cluster7
LETM2 cluster7
AC003075.4 cluster7
RNF20 cluster7
SPATA8 cluster7
FBXO36 cluster7
LEKR1 cluster7
GJA8 cluster7
RP11-446F17.3 cluster7
POLR1D cluster7
AC009948.5 cluster7
CRYZL1 cluster7
KRT23 cluster7
RP11-358H12.1 cluster7
SOBP cluster7
SYNJ2BP cluster7
ADAM2 cluster7
INAFM2 cluster7
AC007966.1 cluster7
HSF5 cluster7
FDFT1 cluster7
SLC2A5 cluster7
PXDNL cluster7
FAM173A cluster7
SLC1A5 cluster7
RWDD2A cluster7
C17orf80 cluster7
HOXA7 cluster7
C1orf101 cluster7
OVOL1 cluster7
SLC51B cluster7
DUS3L cluster7
PKIG cluster7

ATP5O cluster7
CCDC39 cluster7
TYSND1 cluster7
AIMP2 cluster7
RP11-973N13.4 cluster7
RP1-138B7.7 cluster7
SLCO3A1 cluster7
PRCP cluster7
AKAP12 cluster7
KATNB1 cluster7
C11orf63 cluster7
CASC2 cluster7
C18orf21 cluster7
RP11-417O18.2 cluster7
AC026471.6 cluster7
SPAG8 cluster7
RP11-524O1.4 cluster7
LINC01360 cluster7
B9D1 cluster7
OXLD1 cluster7
CTD-2553L13.10 cluster7
SLC25A2 cluster7
HSPBAP1 cluster7
WDR63 cluster7
TMPO-AS1 cluster7
LINC01424 cluster7
DCDC2 cluster7
CCDC158 cluster7
IQCD cluster7
DNAL4 cluster7
RP11-676F20.4 cluster7
RP5-828H9.1 cluster7
ADNP-AS1 cluster7
CFAP52 cluster7
GRK4 cluster7
FAM132B cluster7
CCDC148 cluster7
CTA-407F11.8 cluster7
ZNF821 cluster7
JHDM1D-AS1 cluster7
LINC00654 cluster7
CHCHD5 cluster7
PDZD9 cluster7
DRC7 cluster7
FAM57B cluster7
MOK cluster7
YBX2 cluster7
RP11-245D16.4 cluster7
UBE2E2-AS1 cluster7
MALL cluster7
SPAG16 cluster7
RP11-389G6.5 cluster7
RP11-121G22.3 cluster7
ZBTB32 cluster7
PIAS2 cluster7
FLJ35934 cluster7

DNAI2 cluster7
RP11-517B11.4 cluster7
C16orf71 cluster7
TMEM202 cluster7
AC079354.5 cluster7
RP11-607P23.1 cluster7
ODF4 cluster7
DGCR5 cluster7
AGBL5 cluster7
COMMD1 cluster7
GS1-24F4.2 cluster7
BLZF1 cluster7
MORC2-AS1 cluster7
LYRM7 cluster7
RP11-148B18.1 cluster7
ITGB6 cluster7
OVCH1-AS1 cluster7
LINC00882 cluster7
ART5 cluster7
LRRC37B cluster7
SLC35E4 cluster7
HVCN1 cluster7
TOMM5 cluster7
RP11-626G11.5 cluster7
CEP70 cluster7
CFAP43 cluster7
WDR88 cluster7
RP1-90J20.8 cluster7
FAM72A cluster7
CAPN11 cluster7
IAH1 cluster7
CBARP cluster7
RP11-760D2.5 cluster7
RP11-203B9.4 cluster7
AKAP6 cluster7
RP11-52L5.6 cluster7
BORA cluster7
NUDT16 cluster7
SLC15A4 cluster7
ADO cluster7
KLHDC10 cluster7
NOL4L cluster7
STAMBPL1 cluster7
STARD6 cluster7
TTC12 cluster7
TMEM217 cluster7
SNHG10 cluster7
BVES-AS1 cluster7
NME8 cluster7
C10orf25 cluster7
B3GALT4 cluster7
PHYHIPL cluster7
AC113617.1 cluster7
LINC00911 cluster7
HOXA-AS2 cluster7
ODC1 cluster7

NEFM cluster7
SOD3 cluster7
RIIAD1 cluster7
HIST1H2BC cluster7
CCDC87 cluster7
PLA2G6 cluster7
NUDT6 cluster7
SPAG5 cluster7
ATXN7L1 cluster7
C11orf49 cluster7
IDE cluster7
ATG9A cluster7
KIAA1324 cluster7
ZNF689 cluster7
DAW1 cluster7
DAPK3 cluster7
PAPOLB cluster7
APOM cluster7
RP11-410L14.2 cluster7
DHRS4 cluster7
CTC-203F4.2 cluster7
PCDHB7 cluster7
NUDCD3 cluster7
SMIM2-AS1 cluster7
TRPT1 cluster7
RFX2 cluster7
AP2B1 cluster7
RP11-568N6.1 cluster7
SRA1 cluster7
BMPR1B-AS1 cluster7
DNAH8 cluster7
KIAA1257 cluster7
MTERF2 cluster7
WFDC3 cluster7
AC093901.1 cluster7
FAM126A cluster7
CCDC50 cluster7
RABGAP1L cluster7
TRIM24 cluster7
DNAI1 cluster7
RP11-235C23.5 cluster7
SIGLECL1 cluster7
RORA-AS1 cluster7
RP1-63G5.8 cluster7
OVOL2 cluster7
CAMKMT cluster7
GOLGA8G cluster7
SMARCA2 cluster7
AC009473.1 cluster7
ZNF667-AS1 cluster7
RP11-554D20.1 cluster7
C2orf61 cluster7
RAB32 cluster7
RP11-462G2.2 cluster7
FAM186B cluster7
HMMR cluster7

PPA2 cluster7
FTCD cluster7
TAF9 cluster7
RP11-295G20.2 cluster7
RP11-573D15.9 cluster7
RP11-68L18.1 cluster7
C15orf39 cluster7
CINP cluster7
CPTP cluster7
RPUSD3 cluster7
MTRF1 cluster7
COQ5 cluster7
SSH2 cluster7
UBXN10 cluster7
IGHMBP2 cluster7
MRGPRG-AS1 cluster7
RP5-908M14.9 cluster7
MED26 cluster7
DACH1 cluster7
LINC00221 cluster7
ABHD8 cluster7
GGPS1 cluster7
PRKACG cluster7
LINC00879 cluster7
CILP2 cluster7
STYXL1 cluster7
BIRC8 cluster7
RP11-758H9.2 cluster7
GALNT15 cluster7
TP53I3 cluster7
LACTB2-AS1 cluster7
RP4-561L24.3 cluster7
IL13 cluster7
CAPNS1 cluster7
TRAPPC12 cluster7
SLC25A19 cluster7
RP11-628E19.2 cluster7
KLB cluster7
CTD-3028N15.1 cluster7
ADRBK2 cluster7
FAM227B cluster7
RP11-67L3.4 cluster7
CCDC170 cluster7
ERICH1-AS1 cluster7
RP3-496C20.1 cluster7
DCAF10 cluster7
ARMC2 cluster7
NAPRT cluster7
RP4-816N1.6 cluster7
KB-1410C5.2 cluster7
GEMIN4 cluster7
RAB13 cluster7
KRT72 cluster7
PPP2R5C cluster7
CCDC86 cluster7
LINC00305 cluster7

RP11-535A19.2 cluster7
THTPA cluster7
RABL3 cluster7
NMNAT3 cluster7
MYCNOS cluster7
CCDC178 cluster7
TPM4 cluster7
PITPNA-AS1 cluster7
ATL1 cluster7
UBE2U cluster7
RP11-89K11.1 cluster7
SLC22A16 cluster7
LINC01431 cluster7
RP11-862L9.3 cluster7
RP11-434E6.2 cluster7
CLHC1 cluster7
AC015922.6 cluster7
DNAH12 cluster7
CTSF cluster7
RP11-402J6.1 cluster7
RAB4A cluster7
CCDC96 cluster7
SVIL-AS1 cluster7
C12orf56 cluster7
MOBP cluster7
ESR2 cluster7
NOSIP cluster7
ANKRD13A cluster7
RP11-539L10.3 cluster7
BTD cluster7
CTD-2162K18.4 cluster7
JMJD1C-AS1 cluster7
MUC12 cluster7
CCDC92 cluster7
ZNF385D cluster7
LINC00662 cluster7
DDT cluster7
C2orf81 cluster7
RP11-697B24.1 cluster7
HEXIM2 cluster7
GIT1 cluster7
SRPK1 cluster7
TMA16 cluster7
TTC21A cluster7
HTATIP2 cluster7
UBAP1 cluster7
IFT22 cluster7
PRADC1 cluster7
DEF6 cluster7
ZDHHC12 cluster7
DUS1L cluster7
SLC5A2 cluster7
DCAF5 cluster7
CFAP221 cluster7
USP3-AS1 cluster7
DZIP1 cluster7

SLC25A51 cluster7
FAM181A cluster7
SMIM22 cluster7
SAMD15 cluster7
CMTM1 cluster7
IPO4 cluster7
FEZ1 cluster7
RP4-613A2.1 cluster7
DPP10-AS1 cluster7
MVD cluster7
SRRT cluster7
MTRF1L cluster7
TROAP cluster7
EFCAB5 cluster7
EIF3F cluster7
PTPMT1 cluster7
LIPT2 cluster7
ZFP91 cluster7
SERP1 cluster7
RHBDD2 cluster7
ADORA2A-AS1 cluster7
SCG5 cluster7
RNMTL1 cluster7
STK11 cluster7
SMPD2 cluster7
PLK4 cluster7
STK16 cluster7
CEP83-AS1 cluster7
CCDC7.1 cluster7
APITD1-CORT cluster7
SAXO2 cluster7
CCDC157 cluster7
ZNF675 cluster7
SLC2A8 cluster7
RAB11FIP5 cluster7
NSMCE1 cluster7
SRSF12 cluster7
DALRD3 cluster7
FZR1 cluster7
CIT cluster7
ACADS cluster7
PNPLA6 cluster7
LRRC1 cluster7
COPS5 cluster7
RMND5B cluster7
KCMF1 cluster7
ST7L cluster7
C21orf2 cluster7
SPPL2B cluster7
TECR cluster7
AARD cluster7
FAM161A cluster7
AGFG1 cluster7
SLAIN2 cluster7
MTFR1L cluster7
DNAJC30 cluster7

PSMD8 cluster7
MGAT4D cluster7
ENDOG cluster7
RP11-1006G14.2 cluster7
KYNU cluster7
CHPT1 cluster7
AC138647.1 Cluster8
CLDND2 Cluster8
GOLGA6L2 Cluster8
LRRC73 Cluster8
TRAPPC2P1 Cluster8
SVIP Cluster8
RP11-547C13.1 Cluster8
C11orf65 Cluster8
C15orf65 Cluster8
CIB4 Cluster8
C11orf97 Cluster8
C6orf118 Cluster8
GOT1L1 Cluster8
GOLGA6L10 Cluster8
TPT1-AS1 Cluster8
NDUFB6 Cluster8
PRR22 Cluster8
ZNF563 Cluster8
CXorf66 Cluster8
USMG5 Cluster8
GPR85 Cluster8
CYTH2 Cluster8
IGSF10 Cluster8
TFB1M Cluster8
TIPARP Cluster8
C4orf17 Cluster8
RP11-10A14.3 Cluster8
PIH1D3 Cluster8
RBBP6 Cluster8
MRPS21 Cluster8
XXbac-BCX196D17.5 Cluster8
C14orf142 Cluster8
CTNNBL1 Cluster8
GOLGA6L4 Cluster8
KBTBD3 Cluster8
SUV39H2 Cluster8
KIAA1143 Cluster8
USP7 Cluster8
REC8 Cluster8
PYROXD1 Cluster8
ERGIC2 Cluster8
SH3BGR Cluster8
TOMM20L Cluster8
MAP7 Cluster8
7-Mar Cluster8
FSIP2 Cluster8
AGPS Cluster8
KATNA1 Cluster8
SOD2 Cluster8
FARP1 Cluster8

TPRG1 Cluster8
GNG5 Cluster8
DEPDC7 Cluster8
RBM7 Cluster8
USP16 Cluster8
ATP5S Cluster8
HINT3 Cluster8
C19orf18 Cluster8
DDHD1 Cluster8
EML2 Cluster8
PPIL6 Cluster8
RP11-202D1.2 Cluster8
ZNF165 Cluster8
SUDS3 Cluster8
SPG20 Cluster8
EFHC1 Cluster8
GPCPD1 Cluster8
KPNA6 Cluster8
METTL6 Cluster8
TDRD6 Cluster8
METTL3 Cluster8
ZNF569 Cluster8
GOLGA6L9 Cluster8
FANCF Cluster8
AKAP13 Cluster8
INSL6 Cluster8
OLA1 Cluster8
SNAP29 Cluster8
C4orf47 Cluster8
RBPMS Cluster8
UBE2W Cluster8
11-Mar Cluster8
BTG1 Cluster8
TUBE1 Cluster8
SENP2 Cluster8
PACRGL Cluster8
SREK1IP1 Cluster8
SAR1B Cluster8
HIBCH Cluster8
EMC8 Cluster8
ANKRD42 Cluster8
LRRFIP2 Cluster8
CCDC82 Cluster8
CDKN2AIP Cluster8
TP53TG3D Cluster8
CEP162 Cluster8
ZNF106 Cluster8
MRPL39 Cluster8
HMGB3 Cluster8
HMGN1 Cluster8
5-Mar Cluster8
FAM177A1 Cluster8
NEDD1 Cluster8
LRCH4 Cluster8
POLK Cluster8
MAGI1 Cluster8

UPF3B Cluster8
SLC35G2 Cluster8
TTLL7 Cluster8
FH Cluster8
DNAH14 Cluster8
HOXD8 Cluster8
CPD Cluster8
SKIL Cluster8
RASA2 Cluster8
C11orf88 Cluster8
WDR78 Cluster8
C9orf135 Cluster8
LRRC39 Cluster8
CDRT15L2 Cluster8
C1orf105 Cluster8
H1FOO Cluster8
CFAP126 Cluster8
GEMIN6 Cluster8
LRRIQ1 Cluster8
BBS5 Cluster8
DYNC2LI1 Cluster8
LINC01351 Cluster8
FAM24A Cluster8
CCDC67 Cluster8
PAIP2B Cluster8
AC092687.5 Cluster8
PHOSPHO2 Cluster8
OLFML2B Cluster8
ZNRD1 Cluster8
CTAGE1 Cluster8
LINC00694 Cluster8
TBC1D15 Cluster8
GCA Cluster8
CDRT15 Cluster8
ABRA Cluster8
CTB-181F24.1 Cluster8
DPP10-AS3 Cluster8
RP11-264K23.1 Cluster8
SWT1 Cluster8
C1orf87 Cluster8
TMEM243 Cluster8
BCKDHB Cluster8
CHMP3 Cluster8
RP11-156K13.3 Cluster8
CCDC138 Cluster8
ODF3L1 Cluster8
XKR3 Cluster8
RP11-700N1.1 Cluster8
ZNF85 Cluster8
LHFP Cluster8
SPEF2 Cluster8
NUTM1 Cluster8
ENPP2 Cluster8
RP11-90C4.3 Cluster8
CCDC81 Cluster8
RP11-272J7.4 Cluster8

LINC01628 Cluster8
GOLGA8N Cluster8
GOLGA8R Cluster8
SOS1 Cluster8
NBPF19 Cluster8
NKX6-1 Cluster8
C8orf89 Cluster8
AC015849.2 Cluster8
C6orf229 Cluster8
ALS2CR12 Cluster8
FAM24B Cluster8
EFHB Cluster8
DUSP10 Cluster8
RP11-495K9.3 Cluster8
RGL4 Cluster8
RP11-411B10.2 Cluster8
CTD-2588C8.8 Cluster8
LINC00703 Cluster8
TSPAN19 Cluster8
ARL5B Cluster8
PRSS46 Cluster8
RP11-202D18.2 Cluster8
HIPK4 Cluster8
RP11-252M21.6 Cluster8
MRPS30 Cluster8
LRRC3B Cluster8
DHX32 Cluster8
RP11-495P10.1 Cluster8
AC007740.1 Cluster8
AY269186.2 Cluster8
LIPJ Cluster8
ZNF222 Cluster8
PLN Cluster8
GOLGA8S Cluster8
CMC1 Cluster8
CCDC30 Cluster8
RCN1 Cluster8
CDH18 Cluster8
PPP1R27 Cluster8
TRAC Cluster8
ARFGEF3 Cluster8
CTC-459M5.2 Cluster8
XXbac-BPG308J9.3 Cluster8
RP11-128B16.3 Cluster8
RP11-317P15.3 Cluster8
MLIP-AS1 Cluster8
LRRC69 Cluster8
TMTC1 Cluster8
RP11-532M24.1 Cluster8
IL12A-AS1 Cluster8
AP005530.1 Cluster8
CFAP77 Cluster8
GOLGA8M Cluster8
DCLK3 Cluster8
SEC23B Cluster8
CATSPER4 Cluster8

C11orf53 Cluster8
HEATR4 Cluster8
SLAIN1 Cluster8
RP11-171N4.3 Cluster8
LINC00869 Cluster8
TIGD4 Cluster8
LIMCH1 Cluster8
TWSG1 Cluster8
POC5 Cluster8
NRXN1 Cluster8
CFAP58 Cluster8
MBD5 Cluster8
RP11-785F11.1 Cluster8
RP11-789F5.1 Cluster8
RP11-297L17.2 Cluster8
SPATA41 Cluster8
LENG1 Cluster8
IPO8 Cluster8
TRIM33 Cluster8
RP11-872J21.3 Cluster8
RP1-198K11.5 Cluster8
SIRPG Cluster8
TFPI Cluster8
KCNMA1-AS1 Cluster8
DST Cluster8
RP11-2C7.1 Cluster8
DNAJB13 Cluster8
ZSWIM2 Cluster8
SLC35D1 Cluster8
ZBTB48 Cluster8
CCL20 Cluster8
CTD-2014E2.6 Cluster8
C2orf78 Cluster8
DNAH6 Cluster8
RP11-637A17.2 Cluster8
RP11-421L21.3 Cluster8
RP11-254I22.3 Cluster8
C1orf189 Cluster8
SLC9A2 Cluster8
RP11-99L13.1 Cluster8
DTHD1 Cluster8
LINC01534 Cluster8
RP11-295G12.1 Cluster8
C10orf88 Cluster8
RP11-16N2.1 Cluster8
RP11-395P13.4 Cluster8
SDE2 Cluster8
RP11-734K21.3 Cluster8
CARS Cluster8
RSPH3 Cluster8
C7orf72 Cluster8
CPXCR1 Cluster8
LINC01040 Cluster8
ERICH6 Cluster8
AC078842.4 Cluster8
FBXO3-AS1 Cluster8

RP5-1024G6.5 Cluster8
RP13-81N3.2 Cluster8
SDHAF3 Cluster8
RP11-173A6.3 Cluster8
WDFY4 Cluster8
ZNF560 Cluster8
MEI4 Cluster8
RP11-627G18.4 Cluster8
SNX9 Cluster8
CCDC150 Cluster8
RP11-157B13.7 Cluster8
EPSTI1 Cluster8
NCBP2L Cluster8
DYDC2 Cluster8
CTD-2170G1.2 Cluster8
RP11-24P14.1 Cluster8
AGBL3 Cluster8
ZNF789 Cluster8
CTD-2001C12.1 Cluster8
ZNF436-AS1 Cluster8
RABEPK Cluster8
GK Cluster8
NBPF3 Cluster8
SIRT4 Cluster8
KB-1043D8.6 Cluster8
LINC00449 Cluster8
BRINP3 Cluster8
RP11-47P18.2 Cluster8
ANKMY2 Cluster8
ZNF461 Cluster8
RP11-70F11.8 Cluster8
FAM47E Cluster8
ACSS3 Cluster8
ERVW-1 Cluster8
SAMD3 Cluster8
RP11-537A6.9 Cluster8
LINC00877 Cluster8
RP11-219B4.5 Cluster8
LINC01445 Cluster8
RSPH4A Cluster8
RP11-70F11.11 Cluster8
UBE2H Cluster8
GOLGA8Q Cluster8
CHRDL2 Cluster8
RHOQ Cluster8
SLC35G3 Cluster8
GPAT3 Cluster8
FOXB1 Cluster8
LINC01505 Cluster8
EFTUD1 Cluster8
IGF2BP3 Cluster8
RP13-210D15.4 Cluster8
TRDN Cluster8
MIR7-3HG Cluster8
LTA4H Cluster8
FSIP1 Cluster8

MTHFD2L Cluster8
CDIPT Cluster8
DNAH7 Cluster8
RP11-626P14.2 Cluster8
RP11-95M15.1 Cluster8
CFAP57 Cluster8
C4orf51 Cluster8
DACT1 Cluster8
SGMS2 Cluster8
IQCA1L Cluster8
TGIF1 Cluster8
RNF175 Cluster8
DHFRL1 Cluster8
RP11-393I2.4 Cluster8
LINC01066 Cluster8
CAPS2 Cluster8
DPF3 Cluster8
CD19 Cluster8
RP4-738P15.1 Cluster8
KIAA1107 Cluster8
RP11-169N13.4 Cluster8
ZFHX4 Cluster8
RP11-171N4.2 Cluster8
DLX6 Cluster8
LINC01553 Cluster8
RBMS1 Cluster8
CCDC154 Cluster8
AGBL2 Cluster8
SUMF1 Cluster8
PRDM5 Cluster8
EFCAB9 Cluster8
UNC80 Cluster8
P4HTM Cluster8
CATSPER1 Cluster8
ZNF239 Cluster8
VWA5A Cluster8
RFTN1 Cluster8
FLJ40288 Cluster8
IAPP Cluster8
APOBEC2 Cluster8
RP11-223P11.3 Cluster8
FOXN3 Cluster8
AP001048.4 Cluster8
RP4-753D10.5 Cluster8
AC096669.3 Cluster8
KIF27 Cluster8
MTAP Cluster8
GRAMD1C Cluster8
NOL10 Cluster8
AP2M1 Cluster8
SARAF Cluster8
ADCK2 Cluster8
TMEM95 Cluster8
TNIK Cluster8
TRPM1 Cluster8
THBS4 Cluster8

RP11-202D1.3 Cluster8
LAIR1 Cluster8
CASC22 Cluster8
RP11-123O10.3 Cluster8
GOLGA6A Cluster8
ADK Cluster8
KDM3A Cluster8
PVRL3-AS1 Cluster8
CABLES2 Cluster8
AC104781.1 Cluster8
ERICH6B Cluster8
RP11-202G11.2 Cluster8
KISS1 Cluster8
RP11-876F14.1 Cluster8
ZNF891 Cluster8
MDGA2 Cluster8
ATG9B Cluster8
AP003774.5 Cluster8
SNCAIP Cluster8
RP11-756P10.2 Cluster8
RP11-610J23.1 Cluster8
CFLAR Cluster8
GNA11 Cluster8
MTG2 Cluster8
AC012462.2 Cluster8
TPPP3 Cluster8
PELO Cluster8
AC007405.6 Cluster8
SCAMP2 Cluster8
AC114814.3 Cluster8
DTX2 Cluster8
NXPE1 Cluster8
ASTE1 Cluster8
FAM228B Cluster8
KCNU1 Cluster8
RP11-244B22.6 Cluster8
EXOC6B Cluster8
RP11-417F21.1 Cluster8
AC003973.4 Cluster8
RNPC3 Cluster8
RPAP2 Cluster8
RP1-313I6.12 Cluster8
OR4M1 Cluster8
EFHC2 Cluster8
FAM13C Cluster8
ZNF814 Cluster8
FAM122C Cluster8
RP11-15F12.3 Cluster8
SMIM2 Cluster8
ATAT1 Cluster8
JAZF1 Cluster8
HSPB7 Cluster8
ZNF385B Cluster8
EMC9 Cluster8
PRMT5-AS1 Cluster8
IL20RB Cluster8

FAM47B Cluster8
ZNF397 Cluster8
PSME4 Cluster8
NOS3 Cluster8
SYMPK Cluster8
NPAS2 Cluster8
TMED5 Cluster8
SCIN Cluster8
RP11-548M13.1 Cluster8
RDH8 Cluster8
CTDP1 Cluster8
CBY1 Cluster8
STX17 Cluster8
TOR2A Cluster8
TMEM108 Cluster8
GSK3B Cluster8
RP4-625H18.2 Cluster8
ANTXRL Cluster8
DDX53 Cluster8
TGIF2LX Cluster8
AC009365.4 Cluster8
MCOLN2 Cluster8
ARID3A Cluster8
TRAPPC11 Cluster8
SCLT1 Cluster8
CCDC189 Cluster8
LANCL1 Cluster8
TDP1 Cluster8
POMGNT1 Cluster8
TXNL4A Cluster8
ASXL1 Cluster8
RP11-29B9.2 Cluster8
RANBP17 Cluster8
CTD-2291D10.2 Cluster8
CNOT8 Cluster8
MAGI3 Cluster8
HMBOX1 Cluster8
LINC01495 Cluster8
LINC01224 Cluster8
ZNF263 Cluster8
PGGT1B Cluster8
ADIPOR1 Cluster8
ANKAR Cluster8
C18orf54 Cluster8
CADM1 Cluster8
CCDC66 Cluster8
USP15 Cluster8
KPNA1 Cluster8
CDH2 Cluster8
MCFD2 Cluster8
CPA5 Cluster8
WDR41 Cluster8
RFX1 Cluster8
EPS8 Cluster8
CMTR2 Cluster8
STX2 Cluster8

CNN3 Cluster8
KLHL12 Cluster8
CUL3 Cluster8
ZNF766 Cluster8
DIS3 Cluster8
MEG3 Cluster8
NR3C1 Cluster8
RCL1 Cluster8
NOL12 Cluster8
FERMT2 Cluster8
TRUB1 Cluster8
CHCHD3 Cluster8
JUN Cluster8
GRINA Cluster9
ACTL10 Cluster9
C9orf116 Cluster9
C2orf73 Cluster9
TEKT3 Cluster9
PP2D1 Cluster9
CAPSL Cluster9
JARID2-AS1 Cluster9
C5orf49 Cluster9
FAM209A Cluster9
ERICH2 Cluster9
FAM209B Cluster9
MS4A6E Cluster9
TMCO2 Cluster9
C2orf40 Cluster9
ACTL7B Cluster9
SPACA4 Cluster9
TEX29 Cluster9
CCIN Cluster9
ASB17 Cluster9
RP11-502N13.2 Cluster9
C20orf195 Cluster9
CAST Cluster9
IQGAP2 Cluster9
ACTRT3 Cluster9
WBSCR28 Cluster9
SUN5 Cluster9
FAM186A Cluster9
FAM8A1 Cluster9
TEX33 Cluster9
ACOT13 Cluster9
CALCOCO2 Cluster9
SAXO1 Cluster9
AKAP3 Cluster9
CDKN3 Cluster9
NDRG3 Cluster9
RP4-586O15.1 Cluster9
TEX43 Cluster9
PDCL2 Cluster9
RP11-498B4.5 Cluster9
SERP2 Cluster9
FEM1B Cluster9
C1orf111 Cluster9

AC015971.2 Cluster9
DAB1-AS1 Cluster9
SPACA7 Cluster9
PLCZ1 Cluster9
RP11-983G14.1 Cluster9
DYNLT3 Cluster9
SUN3 Cluster9
RP11-561E1.1 Cluster9
TNFAIP6 Cluster9
PSD3 Cluster9
PKHD1 Cluster9
CXXC5 Cluster9
C2orf74 Cluster9
RNF141 Cluster9
IZUMO2 Cluster9
CFAP36 Cluster9
PRSS54 Cluster9
DYNLRB2 Cluster9
LYZL2 Cluster9
FAM92A1 Cluster9
XYLT2 Cluster9
TES Cluster9
DESI1 Cluster9
ROPN1 Cluster9
TXNDC2 Cluster9
TRIM17 Cluster9
LINC01095 Cluster9
SPACA3 Cluster9
DUSP13 Cluster9
CREM Cluster9
CAPN3 Cluster9
RP11-309L24.4 Cluster9
LACE1 Cluster9
AC004510.3 Cluster9
MAN1A1 Cluster9
CTNNA2 Cluster9
TMEM190 Cluster9
C1orf185 Cluster9
ZNF829 Cluster9
SLC44A2 Cluster9
PEX5L-AS2 Cluster9
RP11-413G15.1 Cluster9
C5orf46 Cluster9
TIPARP-AS1 Cluster9
FILIP1L Cluster9
SMIM23 Cluster9
FAM153B Cluster9
ARRDC5 Cluster9
RNF148 Cluster9
C12orf60 Cluster9
ZBTB20 Cluster9
AC109829.1 Cluster9
TRABD2A Cluster9
KLHL7 Cluster9
SULF1 Cluster9
SPATA25 Cluster9

ADSSL1 Cluster9
CCDC89 Cluster9
USP2-AS1 Cluster9
TMEM144 Cluster9
OLAH Cluster9
BBX Cluster9
RP11-545G3.1 Cluster9
RP11-360D2.1 Cluster9
FTMT Cluster9
ACRV1 Cluster9
ZBTB38 Cluster9
SOX6 Cluster9
TRIML1 Cluster9
RP11-779O18.1 Cluster9
CCDC126 Cluster9
KLF17 Cluster9
LINC01125 Cluster9
RP1-202O8.3 Cluster9
TEKT2 Cluster9
RP11-349E4.1 Cluster9
EQTN Cluster9
AC004448.5 Cluster9
ANKRD9 Cluster9
SOX30 Cluster9
ETF1 Cluster9
BRI3 Cluster9
AC116614.1 Cluster9
C17orf64 Cluster9
SCOC Cluster9
ZC3H14 Cluster9
COMMD3 Cluster9
LRRC18 Cluster9
UTRN Cluster9
LINC00643 Cluster9
USP46 Cluster9
CCER1 Cluster9
SWI5 Cluster9
RP11-206L10.9 Cluster9
DIAPH3-AS2 Cluster9
TTC23L Cluster9
PDZD2 Cluster9
TEKT1 Cluster9
EFCAB11 Cluster9
FAM135B Cluster9
NEBL Cluster9
UNC13C Cluster9
RP11-101C21.4 Cluster9
LYZL6 Cluster9
LINC01198 Cluster9
MBD3L1 Cluster9
AC009299.2 Cluster9
AC104654.1 Cluster9
SPANXN5 Cluster9
FAM221B Cluster9
RP11-639B1.1 Cluster9
NLRP1 Cluster9

RP11-684B21.1 Cluster9
ZNRF4 Cluster9
LINC01487 Cluster9
RP4-569M23.5 Cluster9
FKBP7 Cluster9
LYPD6B Cluster9
HOMEZ Cluster9
RP11-85O21.5 Cluster9
AASDHPPT Cluster9
ACRBP Cluster9
CTC-439O9.3 Cluster9
LINC00642 Cluster9
SETD9 Cluster9
SESN3 Cluster9
NPIPB6 Cluster9
LYZL1 Cluster9
MYH3 Cluster9
APPBP2 Cluster9
SMAD1-AS2 Cluster9
RP11-1136G4.1 Cluster9
RP11-407A16.3 Cluster9
CCT8L2 Cluster9
DOC2A Cluster9
FAM205A Cluster9
RP11-219J21.1 Cluster9
ATF5 Cluster9
CCDC183 Cluster9
FAM27C Cluster9
CAND2 Cluster9
RP11-552I14.1 Cluster9
NUPL2 Cluster9
C7orf34 Cluster9
CEP152 Cluster9
STXBP5-AS1 Cluster9
RP11-462P6.1 Cluster9
C4orf45 Cluster9
PDILT Cluster9
DRAM1 Cluster9
MMEL1 Cluster9
C7orf61 Cluster9
NOL4 Cluster9
RUNX2 Cluster9
LRRC52 Cluster9
RP11-58O3.2 Cluster9
RTKN2 Cluster9
LINC01052 Cluster9
PLCH1 Cluster9
RP11-452K12.4 Cluster9
LMNB2 Cluster9
RP11-375O18.2 Cluster9
SPAM1 Cluster9
ADAM29 Cluster9
NPIPB15 Cluster9
TSSK4 Cluster9
SMAD1 Cluster9
GRID1-AS1 Cluster9

KLF5 Cluster9
RP11-100F15.2 Cluster9
TMEM45A Cluster9
GS1-124K5.4 Cluster9
CNTN4 Cluster9
RP11-355E10.1 Cluster9
C6orf10 Cluster9
SLC38A9 Cluster9
LINC01183 Cluster9
AC006019.3 Cluster9
DDX20 Cluster9
LINC01304 Cluster9
RP11-326A19.3 Cluster9
LINC00254 Cluster9
RP11-781A6.1 Cluster9
CCDC105 Cluster9
AC096570.1 Cluster9
HDAC9 Cluster9
CLDN12 Cluster9
USP44 Cluster9
LYSMD2 Cluster9
SPATA9 Cluster9
RP11-560G2.1 Cluster9
LNX1 Cluster9
STAT4 Cluster9
AC079354.3 Cluster9
RP11-550H2.1 Cluster9
EFCAB3 Cluster9
CEP57 Cluster9
LYZL4 Cluster9
LAMA4 Cluster9
RPP38 Cluster9
ARL4D Cluster9
TRMT5 Cluster9
ALLC Cluster9
STK32C Cluster9
CDC40 Cluster9
LINC01589 Cluster9
RP11-22M7.2 Cluster9
SPANXN3 Cluster9
ABCA10 Cluster9
EPHA6 Cluster9
RP11-20B24.5 Cluster9
IZUMO1 Cluster9
ZMYND15 Cluster9
RFX3 Cluster9
RP11-457M11.7 Cluster9
CCDC121 Cluster9
RGPD4-AS1 Cluster9
ARMC3 Cluster9
ZBBX Cluster9
LATS2 Cluster9
C17orf98 Cluster9
RP3-325F22.3 Cluster9
SPACA1 Cluster9
CASC16 Cluster9

SSMEM1 Cluster9
RP11-1080G15.1 Cluster9
RP11-746B8.1 Cluster9
MTPAP Cluster9
C11orf94 Cluster9
C9orf153 Cluster9
AC016831.7 Cluster9
IZUMO3 Cluster9
RP11-829H16.3 Cluster9
RP5-937E21.8 Cluster9
AC019055.1 Cluster9
RP11-614F17.2 Cluster9
TMEM247 Cluster9
C22orf42 Cluster9
AQP7 Cluster9
CTD-2296D1.5 Cluster9
MAATS1 Cluster9
ING1 Cluster9
C9orf131 Cluster9
PCF11 Cluster9
TMEM262 Cluster9
CRADD Cluster9
DIAPH3 Cluster9
NABP1 Cluster9
ODF3 Cluster9
C16orf92 Cluster9
AC097467.2 Cluster9
ARMC4 Cluster9
CFAP45 Cluster9
RP11-456O19.2 Cluster9
ANK2 Cluster9
TUSC7 Cluster9
RMDN2 Cluster9
CARNMT1 Cluster9
RP5-928E24.2 Cluster9
CTD-2135D7.2 Cluster9
ADAD1 Cluster9
CCDC63 Cluster9
PACRG-AS2 Cluster9
LINC01192 Cluster9
ADGB Cluster9
GRM7-AS3 Cluster9
SLC16A12-AS1 Cluster9
RP11-1109M24.5 Cluster9
ETNK1 Cluster9
RP4-726F1.2 Cluster9
RP13-60M5.2 Cluster9
CASC23 Cluster9
RGSL1 Cluster9
IL13RA2 Cluster9
TEX36 Cluster9
LINC01492 Cluster9
RP1-17K7.2 Cluster9
CCDC182 Cluster9
CCDC27 Cluster9
CA10 Cluster9

SIRT1 Cluster9
RP11-195L15.2 Cluster9
RP11-157E14.1 Cluster9
FANK1 Cluster9
RP11-557H15.3 Cluster9
CTD-3185P2.1 Cluster9
TTC29 Cluster9
RAB27B Cluster9
LINC00917 Cluster9
RP11-45L9.1 Cluster9
LINC01440 Cluster9
RP11-61G19.2 Cluster9
DHRS9 Cluster9
FAM19A2 Cluster9
PRSS55 Cluster9
LECT2 Cluster9
NUCB2 Cluster9
ATP1A4 Cluster9
RP11-69E11.8 Cluster9
OSGIN2 Cluster9
ITPRIPL1 Cluster9
MEGF11 Cluster9
PPP6R1 Cluster9
RP11-111A21.1 Cluster9
KLF4 Cluster9
AP003025.2 Cluster9
PDP1 Cluster9
CCDC60 Cluster9
C12orf80 Cluster9
UBL3 Cluster9
CUL2 Cluster9
RP11-318C2.1 Cluster9
COL25A1 Cluster9
PIBF1 Cluster9
STK32B Cluster9
RP11-778J15.1 Cluster9
RP11-739N10.1 Cluster9
OR2H1 Cluster9
THCAT158 Cluster9
PRRX2 Cluster9
CCDC116 Cluster9
FAIM2 Cluster9
CAMK4 Cluster9
CTD-2010I22.2 Cluster9
TBC1D28 Cluster9
CATSPER3 Cluster9
RP11-358H18.2 Cluster9
POLR1B Cluster9
ADTRP Cluster9
SLC44A5 Cluster9
RP11-690C23.2 Cluster9
CTD-2542C24.8 Cluster9
MGAT4C Cluster9
TFDP2 Cluster9
C15orf41 Cluster9
APOBEC4 Cluster9

RP11-503L19.1 Cluster9
RP11-712B9.2 Cluster9
TPP2 Cluster9
CNBD1 Cluster9
PCCA-AS1 Cluster9
TEX26-AS1 Cluster9
DIAPH3-AS1 Cluster9
LINC01491 Cluster9
TJP3 Cluster9
POLB Cluster9
TOPORS-AS1 Cluster9
CAB39L Cluster9
PPFIA2 Cluster9
ISPD-AS1 Cluster9
SCN3A Cluster9
CEP41 Cluster9
CASC1 Cluster9
SRGAP1 Cluster9
RP11-168P8.5 Cluster9
RP5-1022P6.4 Cluster9
RP3-417L20.4 Cluster9
LINC00158 Cluster9
RNF217 Cluster9
ADAM20 Cluster9
CCDC102B Cluster9
SPATA5 Cluster9
UGT3A1 Cluster9
ZNF479 Cluster9
RP11-864J10.4 Cluster9
ZNF433 Cluster9
LINC01603 Cluster9
NAALADL2 Cluster9
RP11-268P4.4 Cluster9
TMC7 Cluster9
NOXRED1 Cluster9
ALKBH3-AS1 Cluster9
PCDH9-AS3 Cluster9
HADH Cluster9
ADAM7 Cluster9
CNNM1 Cluster9
RHOBTB1 Cluster9
RP3-388M5.9 Cluster9
RP11-379C10.1 Cluster9
AC011752.1 Cluster9
RP11-474D1.2 Cluster9
RP11-3L23.2 Cluster9
DFNB59 Cluster9
CUL1 Cluster9
RP11-300J18.2 Cluster9
CTD-2201G3.1 Cluster9
SCD5 Cluster9
CTD-2184C24.2 Cluster9
PPARGC1A Cluster9
IFI27L1 Cluster9
AL450226.2 Cluster9
CAGE1 Cluster9

LINC00202-2 Cluster9
CNBD2 Cluster9
ERICH3 Cluster9
RNF14 Cluster9
PDK1 Cluster9
ZSCAN31 Cluster9
PREX2 Cluster9
BX255923.3 Cluster9
RP13-39P12.3 Cluster9
SPATA6L Cluster9
KIAA1683 Cluster9
MMP20 Cluster9
CELF2 Cluster9
MYCBPAP Cluster9
FHAD1 Cluster9
CELF2-AS1 Cluster9
LRRTM3 Cluster9
BTBD16 Cluster9
C2orf42 Cluster9
OPN3 Cluster9
TEAD2 Cluster9
AC104777.4 Cluster9
RP11-394A14.4 Cluster9
PPM1A Cluster9
PLEKHB1 Cluster9
RAP1GDS1 Cluster9
RP11-461O7.1 Cluster9
CFAP65 Cluster9
C14orf28 Cluster9
RP11-6N13.1 Cluster9
NADK2 Cluster9
TENM4 Cluster9
PWRN2 Cluster9
RP11-526A4.1 Cluster9
ACADL Cluster9
FUT10 Cluster9
C3orf84 Cluster9
SEPT4-AS1 Cluster9
FAHD2B Cluster9
PLSCR2 Cluster9
LGALS8 Cluster9
RP11-1060J15.4 Cluster9
PRICKLE2-AS1 Cluster9
PMS1 Cluster9
TMEM89 Cluster9
TAT-AS1 Cluster9
NEK11 Cluster9
GOLGA6L22 Cluster9
MYO3A Cluster9
KIF15 Cluster9
FLT1 Cluster9
KIAA0753 Cluster9
PACRG Cluster9
RP11-213G2.2 Cluster9
ATP6V1E2 Cluster9
FGD4 Cluster9

AC073628.1 Cluster9
TULP2 Cluster9
MFSD14A Cluster9
ANLN Cluster9
RP11-262H14.3 Cluster9
CDKL3 Cluster9
LINC01010 Cluster9
AP1G1 Cluster9
C21orf62-AS1 Cluster9
DYM Cluster9
GOLGA6L1 Cluster9
ULK4 Cluster9
CYP2R1 Cluster9
RP5-827L5.1 Cluster9
NTRK3 Cluster9
UBR7 Cluster9
ELAVL4 Cluster9
ANKMY1 Cluster9
PBXIP1 Cluster9
AXDND1 Cluster9
PEX11G Cluster9
TMF1 Cluster9
C3orf38 Cluster9
RIOK3 Cluster9
SKAP2 Cluster9
SLC38A6 Cluster9
ATP6V1C1 Cluster9
DNAJC24 Cluster9
PHF20 Cluster9
URI1 Cluster9
MORN5 Cluster9
NEK10 Cluster9
SEL1L2 Cluster9
RP11-2O17.2 Cluster9
RP11-79C6.3 Cluster9
CCDC53 Cluster9
AC000036.4 Cluster9
C1orf141 Cluster9
ACSL5 Cluster9
USP50 Cluster9
RP11-163M18.1 Cluster9
ZNF415 Cluster9
PAH Cluster9
RP11-566H8.3 Cluster9
RP11-863K10.2 Cluster9
RP11-386B13.3 Cluster9
RP11-404O13.1 Cluster9
TEKT4 Cluster9
RP11-606D9.1 Cluster9
RP1-207H1.3 Cluster9
RP11-92C4.3 Cluster9
CTD-2050B12.1 Cluster9
EYS Cluster9
GALR1 Cluster9
CPNE8 Cluster9
HEATR9 Cluster9

RP11-510M2.6 Cluster9
AC016909.1 Cluster9
CTC-493L21.1 Cluster9
CLYBL-AS1 Cluster9
RP3-446N13.1 Cluster9
SPAG17 Cluster9
RP11-259K15.2 Cluster9
CNIH2 Cluster9
FCRL5 Cluster9
RP11-387H17.4 Cluster9
ZHX3 Cluster9
BRIP1 Cluster9
RP11-677I18.3 Cluster9
LINC00971 Cluster9
PGR Cluster9
RP3-430A16.1 Cluster9
RP11-670N15.1 Cluster9
FRK Cluster9
CHN2 Cluster9
RP11-428J1.5 Cluster9
KIRREL3-AS2 Cluster9
RP13-653N12.1 Cluster9
MAST4 Cluster9
CCDC68 Cluster9
RP11-392P7.6 Cluster9
RP11-713M6.2 Cluster9
USP25 Cluster9
LINC01330 Cluster9
SLC14A2-AS1 Cluster9
MOSPD1 Cluster9
SLC16A7 Cluster9
