# Supplementary material for: The adult human testis transcriptional cell atlas
Source: Cell Res. 2018 Oct 12;28(12):1141–57. doi: 10.1038/s41422-018-0099-2 (PMC6274646; doi:10.1038/s41422-018-0099-2)
Supplement: Supplementary file 12 — Supplementary information, Table S4 [file 41422_2018_99_MOESM12_ESM.pdf]

Supplementary information, Table S4: Clustering Information of Early Germ Cells (Spermatogonia; Cluster 1 and 2).

Cells	States
Donor2-CGTTCTGTCCACGAAT-1	0
Donor2-TGCTGCTTCTAACTCT-1	0
Donor2-TTTGTCAGTTCGTCTC-1	0
Donor2-CACACAAAGTCGTACT-2	0
Donor2-CGCTATCCATGCCCGA-2	0
Donor3-AACTTTCAGCTGAACG-1	0
Donor3-GAACGGAGTGTTTGTG-1	0
Donor3-ACACCGGTCGGAGGTA-2	0
Donor3-GAATAAGCATGGTCAT-2	0
Donor3-GCATGTAGTCTTCGTC-2	0
Donor3-TGCCAAAAGCACAGGT-2	0
Donor1-CAGCTAATCAAACAAG-1	0
Donor1-GCGAGAACATTGTGCA-1	0
Donor1-AGCGTCGAGATGGGTC-2	0
Donor1-ATCATGGCACCACGTG-2	0
Donor1-CATGGCGGTACAGCAG-2	0
Donor1-CCGGTAGAGGCCATAG-2	0
Donor1-CGACCTTTCCAGATCA-2	0
Donor1-CGAGCACCAATCCAAC-2	0
Donor1-CGTTGGGAGTGTTGAA-2	0
Donor1-CTCACACCACTGCCAG-2	0
Donor1-GGAACTTAGATCCTGT-2	0
Donor2-ACATCAGGTAAAGGAG-1	0
Donor2-ATCTACTCAGCTGTTA-1	0
Donor2-CTAGCCTAGGCAGGTT-1	0
Donor2-GCCAAATTCATCGATG-1	0
Donor2-GGCGACTCAAGCGCTC-1	0
Donor2-GTGCTTCAGAGGGATA-1	0
Donor2-GTTCTCGCAATGGAAT-1	0
Donor2-TATGCCCAGACTACAA-1	0
Donor2-TCAGGTAGTTACGGAG-1	0
Donor2-TCCACACGTCGAATCT-1	0
Donor2-TTTGTCACAAACGTGG-1	0
Donor2-ACTGAACCACAGATTC-2	0
Donor2-ACTTACTAGGAATTAC-2	0
Donor2-ACTTGTTCATTAGGCT-2	0
Donor2-AGCTCCTGTCACAAGG-2	0
Donor2-CACAAACGTAGTAGTA-2	0
Donor2-CATCGGGGTTGATTGC-2	0
Donor2-CATGACAGTTTGACAC-2	0
Donor2-CCATGTCCAATGGTCT-2	0
Donor2-CGACTTCTCCCATTTA-2	0
Donor2-CGGCTAGTCAGTACGT-2	0
Donor2-CGTGTAAGTTACGTCA-2	0
Donor2-CTAACTTGTTAAAGTG-2	0
Donor2-CTGAAACTCACATACG-2	0
Donor2-CTTACCGGTAGCGTGA-2	0
Donor2-GAAACTCGTCACCTAA-2	0
Donor2-GAACATCTCGGATGTT-2	0
Donor2-GCGCCAAAGCGACGTA-2	0
Donor2-GCTCCTATCCGCGGTA-2	0
Donor2-GGTATTGTCCAGATCA-2	0
Donor2-GTCCTCAGTCCCTACT-2	0
Donor2-GTGTTAGAGCAGGCTA-2	0

Donor2-TAAGAGATCCAGGGCT-2 0
Donor2-TACCTATAGTATGACA-2 0
Donor2-TACTCATAGCTCAACT-2 0
Donor2-TAGTTGGCATGGTCAT-2 0
Donor2-TCGGTAACACAGGAGT-2 0
Donor2-TGACGGCCAAAGGCGT-2 0
Donor2-TGGCGCAAGACGCACA-2 0
Donor2-TTAACTCAGTAAGTAC-2 0
Donor2-TTCTTAGGTCAGCTAT-2 0
Donor2-TTCTTAGTCTCGGACG-2 0
Donor2-TTGGAACAGTTAGCGG-2 0
Donor3-AACACGTCAGGTGCCT-1 0
Donor3-ATCTGCCAGAAACCGC-1 0
Donor3-GCACATAGTTAGAACA-1 0
Donor3-GTCACAATCAACCAAC-1 0
Donor3-GTCATTTGTCCAAGTT-1 0
Donor3-GTGTTAGGTGCAGGTA-1 0
Donor3-TCGTAGAGTCTCGTTC-1 0
Donor3-TGGACGCGTGTTCTTT-1 0
Donor3-TTAACTCAGCTAGTTC-1 0
Donor3-AAACCTGGTGATGTGG-2 0
Donor3-AAGGAGCTCAAACCGT-2 0
Donor3-ACATCAGCAGGGTATG-2 0
Donor3-AGATTGCGTCTTCGTC-2 0
Donor3-AGGCCGTTCCATGAAC-2 0
Donor3-ATGTGTGAGAGCTGGT-2 0
Donor3-CAAGTTGCAGCGTAAG-2 0
Donor3-CCTTTCTAGTGTTGAA-2 0
Donor3-CGATGGCCATCTCGCT-2 0
Donor3-CGCGGTAAGGTACTCT-2 0
Donor3-CGGTTAATCCTAGGGC-2 0
Donor3-CTAGAGTGTTTAGCTG-2 0
Donor3-CTCGTACAGCCAGTAG-2 0
Donor3-CTCGTCAGTACAAGTA-2 0
Donor3-GATTCAGCAAGAAGAG-2 0
Donor3-GGTGCGTTCGGTTAAC-2 0
Donor3-GTACTTTGTTGGTTTG-2 0
Donor3-TAGGCATGTGTGACCC-2 0
Donor3-TCACGAAGTCTTGATG-2 0
Donor3-TGACTTTTCATAGCAC-2 0
Donor1-ACTGCTCCATGTAGTC-1 0
Donor1-CATCGAAGTCTACCTC-1 0
Donor1-CTTACCGCACGCGAAA-1 0
Donor1-GCAATCACAGAGTGTG-1 0
Donor1-GTAACTGGTTGGTGGA-1 0
Donor1-GTATCTTGTCAGCTAT-1 0
Donor1-TGACGGCCAGCTGTAT-1 0
Donor1-TGCCAAATCATCGCTC-1 0
Donor1-TTAGTTCGTATCTGCA-1 0
Donor1-TTGCGTCGTGACTCAT-1 0
Donor1-ACGCAGCTCTGATTCT-2 0
Donor1-ACTGTCCTCATATCGG-2 0
Donor1-AGAGCTTGTAGAGCTG-2 0
Donor1-AGGTCCGCAAAGCGGT-2 0
Donor1-ATCATGGCATAAGACA-2 0
Donor1-ATCTGCCCACATTAGC-2 0

Donor1-CAAGATCCAAGTACCT-2 0
Donor1-CACACTCCATCGGACC-2 0
Donor1-CAGGTGCAGACCGGAT-2 0
Donor1-CATATGGCAGCGAACA-2 0
Donor1-CATCAGAGTCTCGTTC-2 0
Donor1-CATCGGGTCTTGAGAC-2 0
Donor1-CCGTTCAAGGAGTTTA-2 0
Donor1-CCGTTCAAGTGCTGCC-2 0
Donor1-CGTCACTGTAGCTCCG-2 0
Donor1-CGTCAGGAGTAGCGGT-2 0
Donor1-CGTTCTGGTGATAAAC-2 0
Donor1-CTAATGGCAGTCACTA-2 0
Donor1-CTGAAACGTCTCGTTC-2 0
Donor1-CTGCGGATCGTGACAT-2 0
Donor1-CTGTGCTCAGGTCCAC-2 0
Donor1-CTTACCGCAATGACCT-2 0
Donor1-GATGCTATCATGTCTT-2 0
Donor1-GGAATAAGTCGAAAGC-2 0
Donor1-GGTATTGAGCTCAACT-2 0
Donor1-GGTATTGGTCATTAGC-2 0
Donor1-GTACTCCTCACTCCTG-2 0
Donor1-TACGGATAGTTTAGGA-2 0
Donor1-TGCCCATAGCAGATCG-2 0
Donor1-TTAGGACCACGCTTTC-2 0
Donor1-TTTCCTCAGTAGGCCA-2 0
Donor1-TTTGGTTGTTAAGAAC-2 0
Donor2-AAGGTTCAGCGATTCT-1 1
Donor2-AATCCAGTCTCCCTGA-1 1
Donor2-AGAGCGAAGATGGGTC-1 1
Donor2-CCAATCCAGTGGGTTG-1 1
Donor2-CCTTACGGTCCAAGTT-1 1
Donor2-CTCGTCAGTTCTCATT-1 1
Donor2-GCATGTACATCGGACC-1 1
Donor2-GCTGCAGTCACCGTAA-1 1
Donor2-GGATTACTCATTTGGG-1 1
Donor2-TAAGTGCAGGCATGTG-1 1
Donor2-TACAGTGAGGGCTCTC-1 1
Donor2-TGACTAGGTACCGGCT-1 1
Donor2-TGAGAGGAGGAGCGAG-1 1
Donor2-TGAGCATTCGCCAGCA-1 1
Donor2-TGCACCTTCACTTACT-1 1
Donor2-AAAGATGCATGTCTCC-2 1
Donor2-AAAGCAAGTACCATCA-2 1
Donor2-AAAGCAATCACTTCAT-2 1
Donor2-AAGGCAGGTGTTTGTG-2 1
Donor2-AAGTCTGAGCTGAACG-2 1
Donor2-AATCCAGTCCGCTGTT-2 1
Donor2-ACGTCAAGTTAGGGTG-2 1
Donor2-AGCGTCGCAATGGATA-2 1
Donor2-AGGTCCGAGAGCTGGT-2 1
Donor2-CAAGATCTCAGTTCGA-2 1
Donor2-CAGCGACCACTAAGTC-2 1
Donor2-CAGCTGGCATATACCG-2 1
Donor2-CATCGAACAGCTGGCT-2 1
Donor2-CGGCTAGAGTCACGCC-2 1
Donor2-CTTCTCTAGAAACCTA-2 1

Donor2-GACTAACCACAAGTAA-2 1
Donor2-GACTACATCCTTGGTC-2 1
Donor2-GCAGTTACATTGTGCA-2 1
Donor2-GCTGGGTGTCTAAAGA-2 1
Donor2-GCTTGAATCATATCGG-2 1
Donor2-GGAAAGCTCGCGCCAA-2 1
Donor2-GTACTTTAGATGCCTT-2 1
Donor2-GTATCTTAGAAACCAT-2 1
Donor2-TCATTACAGCCAGTAG-2 1
Donor2-TCTCTAATCCCATTTA-2 1
Donor2-TGCTGCTTCTCCCTGA-2 1
Donor3-ACGATACTCCCTGACT-1 1
Donor3-AGGGTGACACTTAACG-1 1
Donor3-AGTAGTCCATTGAGCT-1 1
Donor3-CAACTAGGTTATCGGT-1 1
Donor3-CAGCTGGGTACGCACC-1 1
Donor3-CAGTAACAGTGTACTC-1 1
Donor3-CATGGCGAGCGTAATA-1 1
Donor3-CCACGGATCCAATGGT-1 1
Donor3-GACGTTAAGCACCGCT-1 1
Donor3-GCGACCAGTCGCCATG-1 1
Donor3-GCGAGAATCGTCTGAA-1 1
Donor3-GGTATTGTCCTCTAGC-1 1
Donor3-GTAACGTTCTGAAAGA-1 1
Donor3-GTCGTAAAGCTAGTCT-1 1
Donor3-GTCTTCGCATAAGACA-1 1
Donor3-GTGCGGTAGTACACCT-1 1
Donor3-TGTGGTACAAGCTGGA-1 1
Donor3-TTTCCTCCAGCCAATT-1 1
Donor3-ATTGGACCAACGCACC-2 1
Donor3-ATTTCTGTCCTGCAGG-2 1
Donor3-CACACTCTCGAATGGG-2 1
Donor3-CACCACTCATTATCTC-2 1
Donor3-CCCAATCGTTGAGGTG-2 1
Donor3-CCGGTAGAGGAGTTGC-2 1
Donor3-CTACATTAGAAACCTA-2 1
Donor3-CTAGCCTAGCAAATCA-2 1
Donor3-CTAGCCTTCAGTACGT-2 1
Donor3-CTGCTGTTCGCAGGCT-2 1
Donor3-GCCTCTAGTTAGTGGG-2 1
Donor3-GCGAGAAGTCTAGTGT-2 1
Donor3-TGAGCCGAGAGGGATA-2 1
Donor3-TTTGGTTAGTGAACGC-2 1
Donor1-ACACCGGAGAAACCTA-1 1
Donor1-ACGATGTTCACCCGAG-1 1
Donor1-ACGGGCTAGAGTAATC-1 1
Donor1-ACTGAACAGATGGGTC-1 1
Donor1-AGACGTTTCATTTGGG-1 1
Donor1-AGCGTATCACATTCGA-1 1
Donor1-CATATGGGTGCTCTTC-1 1
Donor1-CCAGCGACAGCTGTGC-1 1
Donor1-CCCAATCGTGTGACGA-1 1
Donor1-CTACGTCTCATTATCC-1 1
Donor1-CTTAGGAGTGATAAAC-1 1
Donor1-GAAACTCAGGCCCTTG-1 1
Donor1-GACGTGCCAAAGGTGC-1 1

Donor1-GACGTTAAGAATTCCC-1 1
Donor1-GCTGGGTTCCAGGGCT-1 1
Donor1-GGGAGATTCGGTTAAC-1 1
Donor1-GTATTCTGTCGATTGT-1 1
Donor1-GTGTTAGCATTGCGGC-1 1
Donor1-TACTTGTAGCTTTGGT-1 1
Donor1-TTAGGACAGCGCCTCA-1 1
Donor1-TTTGTCAAGGACTGGT-1 1
Donor1-TTTGTCACATGGTAGG-1 1
Donor1-AAAGATGAGACAGACC-2 1
Donor1-AAAGTAGGTCGCCATG-2 1
Donor1-AAGGCAGAGTCATGCT-2 1
Donor1-ACAGCTAGTGCAACGA-2 1
Donor1-ACATACGAGTAACCCT-2 1
Donor1-ACCAGTAGTATAGGGC-2 1
Donor1-ACCGTAAAGTTCGCAT-2 1
Donor1-ACGAGCCAGCAGGTCA-2 1
Donor1-ACGATACTCATGTGGT-2 1
Donor1-ACTGCTCGTCCCTACT-2 1
Donor1-AGCAGCCCATGTTGAC-2 1
Donor1-ATCTACTTCCTTGCCA-2 1
Donor1-ATGTGTGAGTACCGGA-2 1
Donor1-CAACCTCCAGGACCCT-2 1
Donor1-CACACTCGTCTCTTTA-2 1
Donor1-CACCAGGCAGCTTCGG-2 1
Donor1-CAGCTAACAGACAGGT-2 1
Donor1-CATATTCGTCTTCAAG-2 1
Donor1-CCACTACTCATCGATG-2 1
Donor1-CCCAGTTAGTTAAGTG-2 1
Donor1-CCTCAGTTCGGCGGTT-2 1
Donor1-CGAGCCAGTCGCGAAA-2 1
Donor1-CGCTATCAGGGCTCTC-2 1
Donor1-CGTCTACCACAACGTT-2 1
Donor1-CTCGTACAGTATGACA-2 1
Donor1-CTCGTCATCCAGATCA-2 1
Donor1-CTGCCTAGTAAGGATT-2 1
Donor1-GACGTGCCAGTAAGAT-2 1
Donor1-GATGAAACACATCCAA-2 1
Donor1-GCGAGAAAGACCCACC-2 1
Donor1-GGACAAGGTCGCTTCT-2 1
Donor1-GGCTCGACATGTCTCC-2 1
Donor1-GGGACCTCAACGATCT-2 1
Donor1-GTAACGTTCACATAGC-2 1
Donor1-GTACTTTCAGCGATCC-2 1
Donor1-GTAGGCCTCCCTAACC-2 1
Donor1-GTCGTAATCTCGCATC-2 1
Donor1-TACCTATCAAACCTAC-2 1
Donor1-TACCTTATCCCTAATT-2 1
Donor1-TACTCATTCACTTCAT-2 1
Donor1-TACTTACGTCCAACTA-2 1
Donor1-TGACAACGTCAAACTC-2 1
Donor1-TGAGGGAAGCACGCCT-2 1
Donor1-TGCCCTAGTGCACCAC-2 1
Donor1-TGCGTGGTCGGATGGA-2 1
Donor1-TGTATTCAGTTAGCGG-2 1
Donor1-TTCGAAGGTCCAGTGC-2 1

Donor1-TTTCCTCGTTGGTGGA-2 1
Donor1-CGAACATTCTGATTCT-2 1
Donor1-AGTCTTTTCTTCGGTC-2 1
Donor3-AACTTTCTCCCGGATG-1 1
Donor3-CATCGGGCATGTTGAC-2 1
Donor1-ACTGAGTAGGATGCGT-2 1
Donor1-TTAGTTCTCATTGCGA-2 1
Donor1-TTTGTCAAGGATGGTC-2 1
Donor1-TGACTAGGTGTGACGA-2 1
Donor1-GACGTTACAGCCAATT-1 2
Donor1-CAAGGCCAGCCCAATT-2 2
Donor3-CGAATGTAGTTGTCGT-1 2
Donor1-CTACACCTCTAACGGT-2 2
Donor1-GTAACTGAGACGCACA-2 2
Donor2-ATAGACCCAAGCCATT-1 2
Donor2-CAAGAAATCTAACTCT-1 2
Donor2-CACATAGGTTATGTGC-1 2
Donor2-CGAACATCACCCTATC-1 2
Donor2-CGAATGTAGAGACTTA-1 2
Donor2-CGTTAGAAGTACGCCC-1 2
Donor2-CTAACTTGTTCCCGAG-1 2
Donor2-GCTCCTACACATTCGA-1 2
Donor2-GGTATTGGTCTAACGT-1 2
Donor2-TGACGGCCAGCGTTCG-1 2
Donor2-TGCGGGTCACATGGGA-1 2
Donor2-TGCTACCCAAACGTGG-1 2
Donor2-TTAGGACAGTCTTGCA-1 2
Donor2-TTCGAAGAGAAAGTGG-1 2
Donor2-TTGACTTTCCCAACGG-1 2
Donor2-ACATACGAGGCCCGTT-2 2
Donor2-ACGGGTCAGAGTAATC-2 2
Donor2-ACTTGTTCAAGCGCTC-2 2
Donor2-AGTTGGTAGCACCGTC-2 2
Donor2-CGTCACTTCGCACTCT-2 2
Donor2-GCATGCGGTAACGTTC-2 2
Donor2-GTAGGCCTCAAGGCTT-2 2
Donor2-TGATTTCAGCTAAACA-2 2
Donor2-TGTGTTTGTTGTCTTT-2 2
Donor3-AAGGTTCCATTGTGCA-1 2
Donor3-ACAGCCGCAAGACACG-1 2
Donor3-ACGGCCAAGACTGTAA-1 2
Donor3-ACGGGTCCAAGTAATG-1 2
Donor3-AGCTTGAAGGATGGAA-1 2
Donor3-CCACCTACACTGTTAG-1 2
Donor3-CTGCGGACAGGTCGTC-1 2
Donor3-CTGTTTAGTATGAAAC-1 2
Donor3-CTTGGCTAGCGCCTTG-1 2
Donor3-GAAGCAGGTTCACCTC-1 2
Donor3-GCATACAGTGCCTGCA-1 2
Donor3-GCTGGGTGTCAAACTC-1 2
Donor3-GGACGTCGTGTGCGTC-1 2
Donor3-GTCACAACAGCTGTGC-1 2
Donor3-GTCTTCGCATCTATGG-1 2
Donor3-TAAGCGTGTAGAAGGA-1 2
Donor3-TCAGCAATCTGCGGCA-1 2
Donor3-TCGCGTTCAAAGTCAA-1 2

Donor3-TGCTACCAGTCACGCC-1 2
Donor3-ACCCACTCAAACCTAC-2 2
Donor3-ACTATCTGTCGCGAAA-2 2
Donor3-AGTGGGACAATGGAAT-2 2
Donor3-ATCTACTTCTCCAACC-2 2
Donor3-ATGGGAGAGCAGGCTA-2 2
Donor3-CAGCAGCGTCTTTCAT-2 2
Donor3-CCTACCAGTTCACCTC-2 2
Donor3-CGCGGTACACAGTCGC-2 2
Donor3-CGGAGCTGTACAGTGG-2 2
Donor3-CTACATTAGTTTCCTT-2 2
Donor3-CTTACCGCATTACCTT-2 2
Donor3-GGAAAGCAGTGTCCAT-2 2
Donor3-GGGTTGCCACCAACCG-2 2
Donor3-GGTGCGTAGCTGCAAG-2 2
Donor3-TACTTGTGTCGCGTGT-2 2
Donor3-TCAGATGAGGTGCAAC-2 2
Donor3-TCCCGATTCTAACCGA-2 2
Donor3-TCGAGGCTCAACGCTA-2 2
Donor1-ACGGAGATCGTCCGTT-1 2
Donor1-AGAGCGACACAACGCC-1 2
Donor1-AGAGCTTGTCGGGTCT-1 2
Donor1-AGGGATGAGGGCTTGA-1 2
Donor1-AGGGATGGTGTCGCTG-1 2
Donor1-CAGCAGCGTATTCTCT-1 2
Donor1-CCAGCGATCAATCACG-1 2
Donor1-CTCTAATAGTAAGTAC-1 2
Donor1-CTTAACTCAGGCTGAA-1 2
Donor1-CTTCTCTCAGCGTAAG-1 2
Donor1-GAATAAGAGTCAAGGC-1 2
Donor1-GACGCGTGTTATCGGT-1 2
Donor1-GCCTCTATCAAAGACA-1 2
Donor1-GTACTTTGTTCCACAA-1 2
Donor1-GTAGTCATCAAAGACA-1 2
Donor1-GTCTCGTCAGACGCTC-1 2
Donor1-TACACGAGTGGACGAT-1 2
Donor1-TACGGGCCATCGGACC-1 2
Donor1-TACTTACCATGAGCGA-1 2
Donor1-TTCTCCTTCCGCATAA-1 2
Donor1-AAGCCGCAGCAAATCA-2 2
Donor1-ACACCAAGTCTTGCGG-2 2
Donor1-ACGGGCTTCCTAGAAC-2 2
Donor1-ACGGGTCCACGAAGCA-2 2
Donor1-ACTGATGAGTGTCCCG-2 2
Donor1-AGAATAGTCCCAAGAT-2 2
Donor1-AGTGGGATCGGTCTAA-2 2
Donor1-ATCCGAATCCCACTTG-2 2
Donor1-ATTACTCGTAAATGAC-2 2
Donor1-CATTCGCGTTGCGCAC-2 2
Donor1-CCATGTCCAAGCGCTC-2 2
Donor1-CCTATTAAGAAAGTGG-2 2
Donor1-CGCGGTACACTGCCAG-2 2
Donor1-CGCTATCTCTGCCCTA-2 2
Donor1-CGCTGGAGTCGCATCG-2 2
Donor1-CTCGAGGCACTAAGTC-2 2
Donor1-CTGAAGTTCAATACCG-2 2

Donor1-CTGATAGGTATAAACG-2 2
Donor1-CTGTTTATCCACGAAT-2 2
Donor1-GACTACAGTGCCTTGG-2 2
Donor1-GCGAGAAGTCACACGC-2 2
Donor1-GCTTGAAAGACAAAGG-2 2
Donor1-GGAATAAGTCTAGCCG-2 2
Donor1-GGAGCAATCACATGCA-2 2
Donor1-GGATGTTAGGACGAAA-2 2
Donor1-GGTGAAGTCAGAGACG-2 2
Donor1-GTCAAGTGTGCCTGGT-2 2
Donor1-TAAACCGGTCCAGTGC-2 2
Donor1-TCAGCAACAGGAACGT-2 2
Donor1-TGGCCAGAGCTACCGC-2 2
Donor1-TGGCCAGGTTGCTCCT-2 2
Donor1-TTGTAGGCAAGCCATT-2 2
Donor3-TCGGGACTCGTCACGG-2 2
Donor1-ACTGATGAGTGATCGG-2 2
Donor1-GGATGTTAGTCCGGTC-2 2
Donor2-TCAGGTATCTACCAGA-1 3
Donor2-TCGTACCGTAGGACAC-1 3
Donor2-TTGCCGTTCCGGCACA-1 3
Donor2-CAAGTTGGTCCGCTGA-2 3
Donor2-TGGTTCCCAGCGATCC-2 3
Donor3-AAAGTAGTCACAAACC-1 3
Donor3-AACTCTTTCAGAGGTG-1 3
Donor3-GAATGAATCCCTTGTG-1 3
Donor3-GATTCAGGTAGCGATG-1 3
Donor3-GGACAGAAGTGAATTG-1 3
Donor3-GTAGGCCCACATCCAA-1 3
Donor3-TCTGAGACACTCAGGC-1 3
Donor3-TGCGCAGGTTGGACCC-1 3
Donor3-AAAGATGGTAGAGCTG-2 3
Donor3-ACCTTTATCCTAGTGA-2 3
Donor3-AGCATACAGATAGGAG-2 3
Donor3-CCACTACGTGAGGGTT-2 3
Donor3-CTTACCGTCAGTGTTG-2 3
Donor3-GCATACAAGAATTCCC-2 3
Donor3-TAGAGCTTCATGCTCC-2 3
Donor1-AACTCCCCACTCTGTC-1 3
Donor1-ATCCACCCACGAAGCA-1 3
Donor1-ATTTCTGAGGCTCAGA-1 3
Donor1-CCACGGAAGCTCCTCT-1 3
Donor1-GGATTACCAGCTGTAT-1 3
Donor1-TACGGGCTCTCTGCTG-1 3
Donor1-TACGGTAGTAAGGGCT-1 3
Donor1-TAGTTGGTCACCATAG-1 3
Donor1-AACCGCGAGGCGTACA-2 3
Donor1-CACCAGGAGGTCGGAT-2 3
Donor1-CACCTTGAGCGTTGCC-2 3
Donor1-CACTCCATCATGTCTT-2 3
Donor1-CATTATCAGTGTTAGA-2 3
Donor1-CCTCTGAGTCCGCTGA-2 3
Donor1-CGGAGCTCAATGGTCT-2 3
Donor1-CGGCTAGAGAGCTTCT-2 3
Donor1-CTGCGGACACTGCCAG-2 3
Donor1-TTGCCGTGTCCTCTTG-2 3

Donor2-CGGAGTCTCTACCAGA-2 3
Donor2-GTAGTCACAATCAGAA-2 3
Donor3-AGCAGCCAGAGACTTA-1 3
Donor3-GAGTCCGTCTGTTTGT-1 3
Donor3-TAGAGCTAGCGGCTTC-1 3
Donor3-AACTCCCTCCCTCAGT-2 3
Donor3-CGGCTAGCAGACGCCT-2 3
Donor3-GTACGTATCTCGAGTA-2 3
Donor3-GTGCATAGTGTTGGGA-2 3
Donor3-TTGGCAATCCCGGATG-2 3
Donor1-TACCTATCATGGGACA-1 3
Donor1-AAATGCCAGCAGACTG-2 3
Donor1-ACTTTCATCTGCAAGT-2 3
Donor1-CAGAGAGAGTTTCCTT-2 3
Donor1-CCATGTCGTTGCGCAC-2 3
Donor1-TGGCCAGCACCACCAG-2 3
Donor2-ATAGACCCAATTCCTT-1 4
Donor2-CTGGTCTAGGATGGAA-1 4
Donor2-GTTCATTGTGTGAAAT-1 4
Donor2-AGCAGCCTCTAACTCT-2 4
Donor2-AGGCCACAGTCAAGCG-2 4
Donor2-CTCACACGTGGTCTCG-2 4
Donor2-GAACCTACAGCAGTTT-2 4
Donor2-GTGAAGGCAGGTGCCT-2 4
Donor3-ACCCACTAGTTACGGG-1 4
Donor3-ATTACTCAGGGTTTCT-1 4
Donor3-CATGCCTCAGACACTT-1 4
Donor3-CCCAGTTAGCGATAGC-1 4
Donor3-CCTACCAAGGTTCCTA-1 4
Donor3-CCTCTGAAGGTCATCT-1 4
Donor3-CGAATGTAGACGCAAC-1 4
Donor3-CTGTGCTCAAGCCATT-1 4
Donor3-GAACCTATCACAATGC-1 4
Donor3-GATCGCGCATAACCTG-1 4
Donor3-GCCTCTATCTAACTTC-1 4
Donor3-TCAATCTAGTTAGCGG-1 4
Donor3-TCATTTGTCAGTTGAC-1 4
Donor3-TGCGCAGGTCGAGATG-1 4
Donor3-ACATGGTAGTTGAGAT-2 4
Donor3-ACCCACTAGGTGCTTT-2 4
Donor3-ATTCTACAGCCCAACC-2 4
Donor3-CAGCTGGTCAGTTAGC-2 4
Donor3-CATTCGCTCTGCCCTA-2 4
Donor3-CCTCTGATCAAGATCC-2 4
Donor3-CGGCTAGTCGGTTCGG-2 4
Donor3-CTCGAAATCTACTTAC-2 4
Donor3-CTGATAGCATGTTCCC-2 4
Donor3-GCATGTAGTTCGTCTC-2 4
Donor3-GCGCAGTGTCTCCACT-2 4
Donor3-GGGAGATTCCTTGGTC-2 4
Donor3-GGGTCTGAGTAAGTAC-2 4
Donor3-GTAGTCAGTGCCTGGT-2 4
Donor3-GTATTCTGTGGCTCCA-2 4
Donor3-TACGGATCATCCCATC-2 4
Donor3-TGCGGGTAGCGAGAAA-2 4
Donor3-TTAGGACTCACTTATC-2 4

Donor1-AACACGTAGTCGCCGT-1 4
Donor1-AAGGTTCAGGAGTCTG-1 4
Donor1-ACACCGGAGAACTCGG-1 4
Donor1-ACAGCTAGTGTGCCTG-1 4
Donor1-ACGAGGATCATCGGAT-1 4
Donor1-ACTTTCAGTTGCTCCT-1 4
Donor1-AGCTCCTTCCTTAATC-1 4
Donor1-AGGCCGTTCCCTTGTG-1 4
Donor1-AGGGTGAAGATATGCA-1 4
Donor1-ATTACTCCACCAGGTC-1 4
Donor1-CCACCTAGTGCTGTAT-1 4
Donor1-CCGGTAGGTTCTCATT-1 4
Donor1-CGGAGCTGTATCTGCA-1 4
Donor1-CGTAGCGGTAATCACC-1 4
Donor1-CGTCTACAGGCCGAAT-1 4
Donor1-CTAACTTGTCGTCTTC-1 4
Donor1-CTAGAGTCACCACGTG-1 4
Donor1-GCACTCTAGGTACTCT-1 4
Donor1-GGATTACCAGTTCATG-1 4
Donor1-GGTGTTAGTCATTAGC-1 4
Donor1-GTAGGCCCAGCTGTTA-1 4
Donor1-TCGCGTTTCACGCATA-1 4
Donor1-TGCCCATCATCACGAT-1 4
Donor1-TGTATTCGTCGAATCT-1 4
Donor1-TTCCCAGTCGAATCCA-1 4
Donor1-TTCGGTCTCAGGTTCA-1 4
Donor1-TTGGCAAGTGATAAGT-1 4
Donor1-TTTGTCACATTGGGCC-1 4
Donor1-AAGTCTGCAGGCAGTA-2 4
Donor1-ACAGCTAAGGATGTAT-2 4
Donor1-ACGGAGAGTCTCATCC-2 4
Donor1-ACGGCCAGTCAGAAGC-2 4
Donor1-ACGTCAACATGGAATA-2 4
Donor1-ACGTCAAGTCTAACGT-2 4
Donor1-ACTTACTAGGGATGGG-2 4
Donor1-AGAATAGCAGTATAAG-2 4
Donor1-AGCCTAACATGCTAGT-2 4
Donor1-CACACAACAAGTAATG-2 4
Donor1-CACCACTTCAGTTTGG-2 4
Donor1-CACTCCAAGCCGTCGT-2 4
Donor1-CACTCCAGTCGCCATG-2 4
Donor1-CACTCCATCATAGCAC-2 4
Donor1-CAGCTGGTCGTCACGG-2 4
Donor1-CATATTCGTGCACGAA-2 4
Donor1-CATCGGGTCGTTTATC-2 4
Donor1-CATTCGCCAGTCACTA-2 4
Donor1-CCACCTAGTTCCAACA-2 4
Donor1-CCATGTCAGAAAGTGG-2 4
Donor1-CCTAAAGTCGTGGTCG-2 4
Donor1-CCTAGCTAGTGGAGTC-2 4
Donor1-CGATGTACAAGCTGAG-2 4
Donor1-CGCGTTTAGATATGCA-2 4
Donor1-CGCGTTTCACATGGGA-2 4
Donor1-CGGCTAGGTATAGGGC-2 4
Donor1-CGTGAGCGTCCGTGAC-2 4
Donor1-CGTGTAAAGCCTATGT-2 4

Donor1-CTAAGACGTATCAGTC-2 4
Donor1-CTACGTCCACAGATTC-2 4
Donor1-CTCATTACACTACAGT-2 4
Donor1-CTCGGAGGTCCCTACT-2 4
Donor1-CTCGTCAAGCTAAGAT-2 4
Donor1-CTCGTCATCCGAAGAG-2 4
Donor1-CTGCCTAAGGAGTTGC-2 4
Donor1-CTGCCTACATACTACG-2 4
Donor1-CTTAGGAAGATACACA-2 4
Donor1-GACACGCAGCTACCTA-2 4
Donor1-GACAGAGTCGAGAACG-2 4
Donor1-GATCTAGAGTTGAGAT-2 4
Donor1-GCACTCTAGGGTTTCT-2 4
Donor1-GCAGCCAGTGTGACCC-2 4
Donor1-GCATACAGTTCCGTCT-2 4
Donor1-GCCAAATAGCGAGAAA-2 4
Donor1-GGCAATTTCGCCTGAG-2 4
Donor1-GGGATGAAGAGGACGG-2 4
Donor1-GTACGTATCTTGTATC-2 4
Donor1-GTAGGCCAGCCGTCGT-2 4
Donor1-GTCTTCGCATATGCTG-2 4
Donor1-GTGGGTCTCCCTAATT-2 4
Donor1-TACGGGCCATTGGGCC-2 4
Donor1-TCATTTGCATGTAGTC-2 4
Donor1-TCGCGAGGTCAGCTAT-2 4
Donor1-TCTGGAATCATTATCC-2 4
Donor1-TCTGGAATCTGTCTAT-2 4
Donor1-TGCCCATAGACTACAA-2 4
Donor1-TGCCCTACACATCCAA-2 4
Donor1-TGTATTCAGGCCATAG-2 4
Donor1-TGTCCCAAGACTTTCG-2 4
Donor1-TTTCCTCTCCGTTGTC-2 4
