# Supplementary material for: The adult human testis transcriptional cell atlas
Source: Cell Res. 2018 Oct 12;28(12):1141–57. doi: 10.1038/s41422-018-0099-2 (PMC6274646; doi:10.1038/s41422-018-0099-2)
Supplement: Supplementary file 13 — Supplementary information, Table S5 [file 41422_2018_99_MOESM13_ESM.pdf]

Supplementary information, Table S5: List Genes that Show Differential Expression Patterns in Different States.

Gene	GROUP
C19orf84	1
MSL3	1
PIWIL4	1
RPL22L1	1
TUBA1A	1
NOC4L	1
CNN1	1
PHGDH	1
VIM	1
EGR4	1
DCAF12	1
RAC3	1
AZU1	1
APBB1	1
DUSP5	1
ST3GAL4	1
CRIP2	1
INCA1	1
LIN7B	1
SCT	1
CHD7	1
ACTG2	1
GAN	1
BAIAP2	1
PKM	1
SPOCD1	1
RGS14	1
CAMK2B	1
FSD1	1
HOXC9	1
PLPPR5	1
NRG2	1
RNASET2	1
GUK1	1
CSRP2	1
SPI1	1
SERTAD4-AS1	1
FNDC4	1
MROH6	1
PLPPR3	1
FOXP4	1
TMEM63B	1
PSMF1	1
KRT8	1
SURF2	1
HACD4	1
PKN1	1
AGGF1	1
SPO11	1
OPTN	1
SOX17	1
TSPAN33	1
NUDT14	1
RHOBTB3	1

FOXD1 1
SLC1A6 1
SP5 1
STRN4 1
SCARB2 1
PLPP2 1
METTL5 1
KDM2B 1
AIG1 1
FBXO47 1
PVALB 1
VCAM1 1
KLC3 1
ANKRD37 1
OXT 1
CEP131 1
FIP1L1 1
RP11-307P5.1 1
KANSL1 1
NMU 1
HTATIP2 1
TKT 1
MCRS1 1
PPP1R36 1
CEP85L 1
TMEM189 1
MYOM2 1
OLIG1 1
KRT25 1
PPP6R1 1
SF3A2 1
SNAPC2 1
DRC7 1
FAM159B 1
NKX6-2 1
C1orf233 1
RP11-96B5.4 1
MARCH10 1
PPM1J 1
RABGGTA 1
EPAS1 1
MT3 1
HSF2 1
RASGEF1A 1
KAT8 1
RPRM 1
SPINK5 1
POU3F1 1
MIF4GD 1
FMR1 1
DND1 1
RP1-69M21.2 1
EMX1 1
HLA-A 1
COL21A1 1
FAIM2 1

LANCL2 1
RBFOX3 1
TOLLIP 1
EMB 1
PALLD 1
ANXA7 1
SFMBT1 1
MFHAS1 1
CD47 1
CADM2 1
YPEL3 1
CCDC172 1
EXOSC9 1
ACE 1
MUC12 1
HOXA7 1
KCNAB1 1
HOPX 1
HCST 1
IRX2 1
PAK4 1
ASPSCR1 1
MAP2K7 1
DBX2 1
SH3GL2 1
REEP2 1
CRYL1 1
KDM1B 1
NIN 1
ACTR8 1
MAPKAPK2 1
CNOT3 1
SYCP1 1
ISL1 1
UBTD1 1
ZNF704 1
SNHG18 1
CBARP 1
TCEAL2 1
PDCD7 1
INSR 1
LMO7 1
LYSMD2 1
TESK1 1
ELMSAN1 1
SCGB3A1 1
NR2F1 1
SPEG 1
MAPK8IP1 1
SRRT 1
TDRD5 1
RP11-973H7.1 1
SMARCD3 1
SLC41A3 1
PRR5 1
AIF1L 1

DEAF1 1
AC005863.2 1
CXXC5 1
EDEM1 1
DEGS2 1
KCNH2 1
WNT3 1
AATF 1
HAGLROS 1
MAPK3 1
ACAP3 1
DLX6 1
CRYBA4 1
LGALS3 1
PGF 1
RP13-942N8.1 1
SAMM50 1
SPSB1 1
PQLC1 1
FGF9 1
CHEK2 1
AGAP3 1
ZBTB43 1
MAL2 1
DLGAP3 1
PPP1R1B 1
TSPAN12 1
MARCH2 1
PIAS4 1
MPP6 1
N4BP2L1 1
AFAP1L1 1
GBP2 1
SLC25A1 1
RAB31 1
CEBPB 1
FAM89A 1
NAALAD2 1
COBL 1
DLGAP4 1
HMSD 1
PRR35 1
ANGPTL4 1
EAPP 1
AREG 1
RASGEF1B 1
CXCL14 1
DAB2IP 1
TEAD4 1
MTURN 1
ATIC 1
CELF4 1
CHRDL2 1
H2AFY2 1
TRAM2-AS1 1
PDLIM4 1

PRKX 1
CNN3 1
ZCCHC14 1
AP2A1 1
HLA-DPA1 1
PAK6 1
POLR3C 1
RP11-491F9.1 1
CCDC110 1
RP4-639F20.1 1
CITED1 1
MPP1 1
AC097724.3 1
SH2B2 1
CA8 1
ATOH8 1
AIMP2 1
BOP1 1
DYRK4 1
ERICH5 1
RASL11B 1
ALDH3A1 1
SKIDA1 1
MED24 1
ZDHHC21 1
CACNB3 1
TESC 1
ARHGDIG 1
DNAJC1 1
DES 1
PHC2 1
INSIG1 1
ASCL2 1
SMAD6 1
NKAPL 1
DGAT2 1
STAT5B 1
FAM46A 1
ETNK2 1
ZNF236 1
GGACT 1
CAMK1 1
ARRDC3 1
RP11-386G11.5 1
CDC14B 1
SDR16C5 1
ASPHD2 1
RBP4 1
VAV3 1
LINC01447 1
PPP1R3F 1
SNHG19 1
HECA 1
FAM102A 1
ASXL3 1
GLUD1 1

NUB1 1
BAG3 1
PRKAR1B 1
PAM 1
AKR1A1 1
UHMK1 1
BAIAP2L2 1
CDH7 1
ATP1B1 1
RIMS1 1
POLN 1
CHD5 1
KCNK1 1
RAMP1 1
AP000253.1 1
RAB38 1
DUSP23 1
AVPI1 1
LINC01183 1
TSPAN15 1
RFWD3 1
TRAF7 1
TRIM71 1
DNAJA4 1
IFITM2 1
TRIM39 1
TWIST1 1
CCDC63 1
RP11-256I23.2 1
LXN 1
DYDC1 1
ADAD2 1
HLA-DPB1 1
MED27 1
RP11-255M2.3 1
BSPRY 1
SNTG2 1
LMNTD2 1
PRUNE2 1
KCNMB4 1
ASGR1 1
FAM212A 1
PELI2 1
GPRC5C 1
TTBK2 1
TDRD6 1
LRRC42 1
HMOX1 1
STX17-AS1 1
MSRA 1
SMIM1 1
NES 1
PCAT6 1
SPINT1 1
FXYD5 1
MYO1E 1

EXOC3L2 1
MMP11 1
SLC12A5 1
PARVB 1
RP11-293A21.1 1
GRINA 1
LARP6 1
RFNG 1
ERICH2 1
C1orf115 1
KSR2 1
NEO1 1
MX1 1
KMT2B 1
MAST2 1
INSL6 1
SLC25A22 1
TSC22D1 1
OLFM2 1
SWAP70 1
YAF2 1
CYP4X1 1
FKBP6 1
GPX3 1
WWP1 1
ICA1L 1
MROH8 1
THAP4 1
TRIM36 1
NBL1 1
TMEM39B 1
C19orf73 1
ARHGAP44 1
DCTN6 1
RBCK1 1
HMGCL 1
DFNB31 1
CARS2 1
ASB2 1
SMARCD2 1
STARD10 1
PPP2R5D 1
CAB39 1
PLA2G16 1
LRRC8A 1
DDR1 1
ASUN 1
MORC1 1
DOK4 1
WDR45 1
RBP1 1
TMEM266 1
AKAP12 1
PAPD7 1
SHKBP1 1
LINC01117 1

PMEPA1 1
MAGEC1 1
RP11-989E6.10 1
RUNX3 1
GOLIM4 1
MVB12B 1
HOMER3 1
FAM222A 1
SETD1A 1
NUPL2 1
CD81 1
CDH15 1
RP5-1085F17.3 1
ERCC3 1
RIMKLA 1
CEP170B 1
ADAMTS12 1
IGFBP6 1
PRKAG2-AS1 1
HOXB7 1
CEP112 1
TUSC1 1
AREL1 1
PIP5K1B 1
BRAP 1
EMP3 1
PLK2 1
RFX2 1
GABRG3 1
PLEKHH3 1
TCF7 1
LRIG1 1
DGKD 1
CAB39L 1
RBFOX2 1
TRPM4 1
SCAMP5 1
AKT1S1 1
LECT1 1
KRTDAP 1
C19orf81 1
OLA1 1
DYRK2 1
CTHRC1 1
MAP2K5 1
RAB32 1
CIART 1
RP11-108M9.4 1
TMEM247 1
IGF2 1
IGFBP4 1
PER1 1
RUNX1T1 1
MAGEB1 1
LMCD1 1
JUND 1

PCGF5 1
SGCG 1
ZNF746 1
CCNO 1
INPP5K 1
SLC35F2 1
COG1 1
TSC22D4 1
ZBED6 1
FLCN 1
ZNF628 1
MAP2K2 1
DIAPH2 1
GSS 1
STAU2 1
SEMA6D 1
ZNF496 1
BORCS5 1
MAP4K2 1
FAM69B 1
AC136616.1 1
ALS2 1
ZMYM4 1
B3GALT6 1
FAM184A 1
AC009961.3 1
KPNA4 1
STAT3 1
PDE4A 1
WNK2 1
ZC3H3 1
BRAF 1
CXADR 1
CNFN 1
AFTPH 1
CLU 1
PIK3CA 1
RIMS4 1
MGST2 1
BEX1 1
LINC01623 1
TMEM31 1
BEX4 1
HYLS1 1
S100A10 2
TUBB 2
FKBP8 2
YBX3 2
PCSK1N 2
STK24 2
MFF 2
AES 2
RPS4X 2
SERPINE2 2
SSBP3 2
ID4 2

FOXP1 2
LY6E 2
FAM92A1 2
LMO4 2
MYO6 2
ID2 2
ID1 2
YPEL2 2
ME2 2
SMARCA1 2
STK31 2
GYG1 2
ATP9A 2
C15orf61 2
IGF2BP2 2
DTNB 2
RNF146 2
FST 2
COMMD3 2
FAM150B 2
FAM26F 2
TCF3 2
TMEM14A 2
SIX1 2
BTF3L4 2
ARID4A 2
CNTNAP2 2
BLVRA 2
PBX1 2
SERINC2 2
GNAS 2
RHOC 2
ROR2 2
LPIN2 2
NDUFS4 2
CERS6 2
HES1 2
TCF4 2
VIPR2 2
ITGB1 2
BNC2 2
MLLT3 2
NEDD4L 2
FHL1 2
IGLON5 2
ATF5 2
DLK1 2
SATB1 2
ADAR 2
NFIB 2
ZBTB16 2
IFI16 2
MYO10 2
TSPYL5 2
STX3 2
PMAIP1 2

CRTAP 2
RP11-11N9.4 2
BFSP1 2
SERPING1 2
NEK6 2
THRA 2
TLK1 2
VAT1 2
PEX13 2
ARRDC4 2
EYA2 2
NFIA 2
C1QTNF4 2
ATXN1 2
DACT1 2
PALM3 2
PHF21A 2
UBXN6 2
HEY1 2
KMT2C 2
RAB5C 2
SYNE2 2
LARP1B 2
UGCG 2
TPBG 2
ELOVL5 2
SKAP2 2
ARFGEF1 2
RP11-51J9.5 2
ARL3 2
ZDHHC14 2
TDRD3 2
PIP4K2A 2
ZFP36L1 2
SGF29 2
RBMS1 2
HBP1 2
TTC39C 2
RNF130 2
CCDC144NL-AS1 2
KAT6B 2
USP42 2
BMPR1B 2
PFKL 2
CNTNAP3B 2
CACFD1 2
CCNJL 2
UACA 2
CEBPD 2
NPB 2
ECI2 2
LY6H 2
PLPP1 2
PPP2R4 2
RP11-314N14.1 2
HERC4 2

MDFI 2
DPP10-AS1 2
FAM114A1 2
VPS26B 2
CLEC11A 2
SESN2 2
LINC01089 2
WASF1 2
LINC01511 2
ZCCHC2 2
SSFA2 2
CUL4A 2
CD55 2
MAP1LC3A 2
CAPRIN2 2
DPYSL2 2
NKAIN4 2
SIRT5 2
AK4 2
NANOS2 2
GSK3B 2
LBH 2
ATG13 2
ZRSR2 2
PTPN14 2
GLTSCR2 2
ZNF428 2
UTF1 2
USP11 2
KRT18 2
GNB2L1 2
MAGEB2 2
COTL1 2
PTRHD1 2
PODXL2 2
BAG6 2
PPP2R1A 2
CDK17 2
GNB2 2
PTOV1 2
ASNS 2
DPPA2 2
SUPT5H 2
NFKBIA 2
IDH2 2
S100A11 2
NEIL2 2
BBX 2
ANXA11 2
DNAJB1 2
GPI 2
A2M 2
AKR1B1 2
SERPINI1 2
RAD21 2
RRBP1 2

C9orf16 2
BLMH 2
HENMT1 2
GPR137 2
FGFR3 2
PIWIL2 2
OSER1 2
UBA1 2
CLTB 2
CHPT1 2
RALY 2
ELOVL4 2
HABP4 2
RAB3IP 2
RAB6B 2
RP11-80F22.15 2
RPL17 2
TOMM34 2
RP11-70C1.1 2
DCAF4L1 2
CIB1 2
ANXA5 2
RP11-849I19.1 2
EPB41L3 2
KCTD13 2
MIR22HG 2
DOCK8 2
PDCL3 2
CPEB1 2
RGMA 2
ALDOA 2
ECHS1 2
MXD4 2
NUDT4 2
DLX5 2
EPC1 2
PRKAG2 2
CCDC106 2
TEX12 2
KLHL23 2
SMAD9 2
GABRB3 2
AC018755.17 2
TMEM123 2
ENHO 2
LCA5 2
SRGAP1 2
FAM43A 2
TANC2 2
ENO3 2
TDP2 2
LINC01030 2
DUSP4 2
KCNQ2 2
SLU7 2
IGSF10 2

AP1AR 2
DDX43 2
BAZ1A 2
MRPL36 2
BARX2 2
WIPF3 2
XAB2 2
HEY2 2
CSNK1E 2
SIAH2 2
SRP54 2
PLEKHA3 2
UPP1 2
SLC9A3R2 2
DYRK1A 2
RAP1GDS1 2
MAGEB16 2
HEBP2 2
SFXN1 2
SUSD4 2
CCDC117 2
EMC3 2
TATDN1 2
KDM7A 2
BIN1 2
PALM 2
BOK 2
GPR27 2
YAP1 2
MRPL37 2
KLF7 2
PARD3 2
RP11-506F3.1 2
MRGBP 2
CHN1 2
TIMMDC1 2
CAST 2
GKAP1 2
CTNNBIP1 2
ZBTB20 2
ARID3B 2
COPS4 2
CXXC4 2
KLHL35 2
ANXA2 2
CBL 2
CDC123 2
ZFAND3 2
DSCR3 2
PSMD14 2
MGAT4A 2
GAREM1 2
CLN6 2
SMIM19 2
WTIP 2
ABRACL 2

DTNBP1 2
AGBL5 2
PRDX6 2
FAM228B 2
SAMD11 2
CHTF8 2
MT-ATP8 2
ADA 2
NRG1 2
PLEKHO1 2
BRD4 2
SLC40A1 2
KCNN2 2
BCAP29 2
NRIP1 2
ATP6V1E2 2
C5orf38 2
MSRB3 2
MIIP 2
SMOX 2
IFT88 2
HMGB3 2
ASZ1 2
CDYL2 2
TSPAN13 2
PTAR1 2
USP46 2
AAED1 2
NCK2 2
RBKS 2
RET 2
GALNS 2
MAGEF1 2
ICA1 2
CBX7 2
FGFR1 2
DEDD2 2
EIF4ENIF1 2
FERMT2 2
APPBP2 2
HNRNPH2 2
SLC26A11 2
UBE2L6 2
HSPA4L 2
DRAM2 2
RAB20 2
CXCL9 2
PACS2 2
CLASP1 2
ISG15 2
PRKACB 2
MRPL47 2
ATP5S 2
CCDC3 2
PRDM6 2
APBA2 2

GPM6B 2
ZNF787 2
LMO2 2
FTCD 2
PPM1A 2
RGS17 2
LINC01481 2
CIB2 2
ZNF827 2
ZNF521 2
AIP 2
PDE4DIP 2
NINJ1 2
SERTAD2 2
LBR 2
EPB41L4A 2
AFF1 2
FAM207A 2
AC002467.7 2
RP11-693J15.6 2
CCDC94 2
GRAMD1A 2
PRMT9 2
MTUS1 2
ZNF106 2
GSTO1 2
L3MBTL3 2
STXBP6 2
ACTN1 2
FYN 2
KLF3 2
ARHGAP32 2
RP11-736K20.6 2
SOBP 2
CCDC91 2
TFAP2A 2
NT5DC1 2
GPC1 2
ATP6V1C1 2
PPP2R2D 2
CDS1 2
MINK1 2
MYH9 2
TP53TG1 2
RGS22 2
IRS2 2
CBR1 2
ARID3A 2
TAF4B 2
TNNI3 2
NR1D1 2
PPP1R1A 2
GSDMD 2
AKAP7 2
TARBP1 2
C14orf37 2

INTS6 2
FN1 2
ZNF701 2
CTC-241N9.1 2
EPHX2 2
DST 2
PPP1R9A 2
TPTEP1 2
LINC01582 2
L1TD1 3
NANOS3 3
CITED2 3
ASB9 3
PABPC4 3
C12orf75 3
ZNF462 3
MEX3B 3
GFRA1 3
TMEM55A 3
ADAMTS6 3
TJP1 3
IRF2BPL 3
TMEM14C 3
MEF2C 3
FAM25G 3
PTPRS 3
RP11-101E5.1 3
ZNF518A 3
RGS10 3
GCC2 3
TCF7L2 3
RAB11B 3
SEPP1 3
UXS1 3
SOCS1 3
FARP1 3
ZC3H13 3
DPPA4 3
CD74 3
LUZP4 3
LTBP1 3
TMEM181 3
RP4-625H18.2 3
FOXC2 3
TADA3 3
TLK2 3
CTSF 3
HMG20B 3
ETV5 3
ZEB2 3
PCMTD1 3
SLC25A31 3
NACC2 3
CCDC144A 3
TPI1 3
ZBED5-AS1 3

SLC7A10 3
HHEX 3
ZFAND2B 3
BAMBI 3
GCNT2 3
GSTZ1 3
SDC2 3
EID1 3
CCND2 3
HNF1B 3
GFRA2 3
SSBP2 3
TSTD1 3
AC002454.1 3
IRF2BP2 3
ALKBH7 3
AGMO 3
RBMX 3
GNAQ 3
CYFIP1 3
DAZAP2 3
GRB14 3
ZNF217 3
SPRY1 3
GRN 3
CCDC109B 3
COX8A 3
A1BG 3
IGF2BP1 3
SEPT11 3
OGFR 3
COX14 3
SERINC5 3
AK5 3
RP3-460G2.2 3
TRIB2 3
TPM2 3
ARID5B 3
AKR1C3 3
NME4 3
RP11-81A22.5 3
DUSP6 3
GNAL 3
ROBO1 3
VGLL4 3
DBN1 3
PIANP 3
KRAS 3
CCDC92 3
IFIT1 3
NME3 3
TACC2 3
RGS2 3
USP12 3
CTNND2 3
RFXANK 3

MRPL28 3
CDCA7L 3
PDK2 3
PNRC1 3
CACNB4 3
GTF2I 3
PYGB 3
IRF3 3
FAM21A 3
ITPKB 3
CAMK2G 3
METTL9 3
ELK3 3
PTPRG 3
WDR60 3
KLHDC8B 3
BNC1 3
SMAP2 3
SLC35D2 3
FLJ20021 3
MRAS 3
NUDT7 3
TMEM251 3
PLS3 3
OTUD1 3
DCTN2 3
AP001372.2 3
UBE2H 3
COL9A3 3
RAB4B 3
MTMR14 3
TDRD1 3
NIPSNAP3A 3
GABARAPL1 3
TMEM261 3
LRP5L 3
ARPC1B 3
SERTAD3 3
RNF8 3
LYPD6 3
SPATC1L 3
MPND 3
POPDC3 3
IMMP2L 3
ZSCAN5A 3
NAA10 3
FGGY 3
TGIF1 3
FAT1 3
RABGAP1 3
NREP 3
ZNF362 3
MARK4 3
NUDT11 3
CDKN1B 3
HOMER1 3

SPSB3 3
FOXN3 3
RHOG 3
TXNDC12 3
TCL1B 3
SGK1 3
RELL2 3
BROX 3
ANP32A 3
TAF7L 3
TATDN2 3
RABL6 3
TXNIP 3
GLIS3 3
BMPR1A 3
PGM1 3
TCN2 3
BEND4 3
ISOC2 3
TJP2 3
ZMYND8 3
BCORL1 3
KLHL42 3
FKBP5 3
FKBP1B 3
TTLL1 3
VAMP5 3
PHF8 3
RP11-219A15.1 3
DHRS11 3
PNMAL1 3
HDHD3 3
EVPLL 3
TP53INP1 3
RBM24 3
SALL4 3
SCAND1 3
TAF10 3
TFDP3 3
PRKD1 3
ACOT13 3
FLJ37035 3
PHF6 3
KLF12 3
PPCS 3
RNF187 3
TLN2 3
IMPA2 3
U2AF1L4 3
CD24 3
NAA60 3
MSRB2 3
NDN 3
RLN2 3
STX7 3
MSI2 3

LSM10 3
CMTM7 3
CELSR2 3
RTCB 3
RP4-816N1.6 3
C8orf82 3
PEX5 3
KIAA1671 3
TIAM1 3
CCDC28B 3
HERC1 3
SLC25A13 3
SNX7 3
TRMT2A 3
SYNRG 3
PCBD1 3
FAM3C 3
CPNE1 3
TOB1 3
FAM21C 3
PCLO 3
C1orf122 3
RP3-512B11.3 3
SMIM10L1 3
LIPT2 3
TNFAIP8 3
LINC01413 3
ZNF532 3
RNF44 3
STX4 3
CCDC71 3
ADI1 3
USP19 3
LYRM5 3
PPP1R10 3
LEPROT 3
IMP3 3
DEK 4
CHAF1A 4
UBE2C 4
DMRT1 4
BIRC5 4
COX20 4
NENF 4
CDT1 4
PRC1 4
ZWINT 4
MKI67 4
RRM2 4
CCNA2 4
SPC25 4
CENPA 4
CDCA5 4
TYMS 4
ASPM 4
WDR34 4

DSN1 4
DHFR 4
TK1 4
NUF2 4
DIAPH3 4
SNRNP25 4
CDK1 4
DPCD 4
MND1 4
ESCO2 4
MYBL2 4
UHRF1 4
PCNA 4
HIST1H2AA 4
CDCA7 4
NCAPG 4
MCM7 4
ISOC1 4
BRI3BP 4
PBK 4
KIF22 4
DNAJC9 4
FBXO5 4
SLF1 4
E2F1 4
PDLIM1 4
RAD51 4
NUDT1 4
ITGB3BP 4
MBD4 4
PKMYT1 4
CENPW 4
CASP8AP2 4
RIBC2 4
FBLN2 4
GTSE1 4
SPC24 4
CDC45 4
STRA13 4
ASF1B 4
MSH2 4
RFC2 4
SNAPIN 4
KIT 4
YY2 4
RSPH1 4
POLD2 4
WDR76 4
CFAP53 4
SKA3 4
GPC4 4
CTBP2 4
PGP 4
DCTPP1 4
MCM2 4
NCAPH2 4

CDCA3 4
CDCA8 4
RMI2 4
SYNC 4
SSX2IP 4
POLD3 4
C1GALT1 4
RPA3 4
CD99 4
CPD 4
TPRKB 4
WEE1 4
POLR3K 4
XRCC2 4
DCAF12L1 4
MYO1B 4
MCM5 4
EFHD1 4
CENPQ 4
KLHL15 4
HIST1H1A 4
HJURP 4
ECI1 4
GMNN 4
KIAA0101 4
BRCA1 4
MELK 4
SKA1 4
DDB2 4
ILF3-AS1 4
C21orf58 4
PLTP 4
ARHGEF26 4
HACD2 4
PNMA1 4
CHRNA5 4
MLX 4
CHEK1 4
CDKN3 4
NAPRT 4
HIST1H1C 4
TEX9 4
WNK3 4
CARHSP1 4
TMEM203 4
FEN1 4
APOA1BP 4
MDM1 4
PARPBP 4
E2F2 4
TRAF3IP1 4
RRAGD 4
RNF4 4
LIMS1 4
ARL6IP6 4
C19orf48 4

FOXM1 4
FRAT2 4
FAM60A 4
ZNF492 4
HOXB3 4
ZNF98 4
MCM4 4
MSH6 4
KEAP1 4
ZWILCH 4
POLR1C 4
PCGF2 4
GLO1 4
SEPSECS-AS1 4
CKAP4 4
KCTD5 4
KIF2C 4
MTSS1 4
CNP 4
RBPMS2 4
CLPTM1L 4
ATAD5 4
ZNF711 4
DLGAP5 4
ENOPH1 4
TCF19 4
ZFYVE9 4
DCAF7 4
TSEN2 4
SH3BGRL3 4
ANLN 4
TTK 4
STIL 4
RP5-821D11.7 4
EXO1 4
GXYLT1 4
FANCD2 4
SCML2 4
TOPBP1 4
DSCC1 4
CENPM 4
LRRC45 4
TP53I13 4
PXK 4
MTERF3 4
INCENP 4
WDR90 4
C22orf46 4
NUSAP1 4
SLC25A11 4
MAD2L1BP 4
POLG 4
PLK1 4
DNA2 4
WHSC1 4
EXOSC5 4

FMNL2 4
CST6 4
DHCR24 4
KIAA1524 4
RIIAD1 4
ZSWIM6 4
RCAN3 4
MTFR2 4
CCNE1 4
KIF15 4
VCPKMT 4
GCAT 4
KIF26A 4
RMI1 4
ATP2A1-AS1 4
C11orf88 4
NFIX 4
LINC00221 4
BLM 4
AGPS 4
PDZD8 4
EFCAB14 4
MASTL 4
DIDO1 4
HYOU1 4
NPAT 4
PIM1 4
VCPIP1 4
SNAPC3 4
KIF23 4
CEP55 4
KCTD20 4
AFMID 4
TNKS2 4
CXorf57 4
CHAMP1 4
AUTS2 4
SPAG5 4
CPNE7 4
RBMXL3 4
HBA2 4
IGDCC3 4
HOMER2 4
HMMR 4
AURKB 4
SHCBP1 4
OIP5 4
ANP32E 4
TRAIP 4
BUB1B 4
CASC5 4
TRIAP1 4
CCND1 4
RACGAP1 4
HNRNPA0 4
SLC31A1 4

VRK1 4
GJC1 4
CENPF 4
MRPS2 4
NUP107 4
C16orf13 4
NDC80 4
IFRD2 4
RAD51AP1 4
NMNAT1 4
TCTN2 4
SF3B3 4
SLC35B3 4
MEN1 4
AEN 4
CCZ1 4
RBM26 4
JPX 4
RP11-379H18.1 4
LMNB2 4
SGOL2 4
NEK2 4
DDIAS 4
APOPT1 4
BUB1 4
KIF20A 4
MYH10 4
TMEM231 4
MECR 4
AGTRAP 4
SEPSECS 4
RADIL 4
POLE2 4
HS2ST1 4
HEXIM1 4
NCAPH 4
CRIP1 4
DDX56 4
EME1 4
PRPF4 4
CENPE 4
CHMP4B 4
ALG6 4
SAAL1 4
TMEM55B 4
WDR77 4
MDC1 4
DEPDC1 4
SKP2 4
CCNT2 4
TGFBR1 4
FGF14-AS2 4
AURKA 4
CKAP5 4
THAP9-AS1 4
UBR7 4

GADD45B 4
IER5 4
RAD18 4
NCAPD2 4
RRP1B 4
PPTC7 4
UBXN11 4
TONSL 4
USP39 4
OXNAD1 4
FBXO45 4
ARHGEF39 4
CPSF1 4
C16orf91 4
VTA1 4
PP7080 4
TARDBP 4
ZNF138 4
PRSS50 5
CTCFL 5
PRSS21 5
SYCP3 5
DPEP3 5
SYCE3 5
PAGE1 5
CLNS1A 5
MYDGF 5
SERBP1 5
BOLL 5
SMC1B 5
MEIOC 5
TEX101 5
DDX21 5
CALR 5
HORMAD1 5
LAMTOR1 5
SELT 5
VCX2 5
C19orf60 5
SRP9 5
FAM173A 5
SDF2L1 5
FAM9B 5
TCL1A 5
REC8 5
SSX3 5
ATP6V0B 5
GINS2 5
TMSB10 5
HSPA5 5
SSR4 5
C1D 5
PNMA5 5
CT55 5
SAR1B 5
SIL1 5

COX7B 5
NDUFC1 5
DMRTB1 5
DAZL 5
ZCCHC17 5
TERF1 5
PPP1R14A 5
SEP15 5
HSP90B1 5
COA1 5
DNAJC19 5
FDX1 5
COX17 5
TUBA3D 5
LRPAP1 5
HDDC3 5
CRELD2 5
SEC11A 5
MED30 5
BOLA3 5
GTF3C6 5
EI24 5
CNIH1 5
TOP2A 5
HBQ1 5
SYCE1 5
MRPS26 5
KRTCAP3 5
FMR1NB 5
PDIA6 5
ZNF280B 5
WDR83OS 5
ATRAID 5
C1orf43 5
TMED10 5
PCMT1 5
DENR 5
C19orf52 5
ZDHHC4 5
CHCHD10 5
MANF 5
TM2D1 5
TMED9 5
CENPU 5
CLSPN 5
MRPL18 5
BHMG1 5
SPESP1 5
TIMM8B 5
TAF12 5
NDUFAF3 5
HIGD1A 5
SQLE 5
ATP6V0E1 5
PIGS 5
RSRP1 5

TXNDC15 5
SDHD 5
PAK1IP1 5
CDKN2D 5
KDELR2 5
SLC35B2 5
TCTEX1D2 5
DHRS13 5
FAM105A 5
UNK 5
TRIM44 5
MLEC 5
TMX1 5
POLR3H 5
C8orf59 5
MYLIP 5
MEIOB 5
ESCO1 5
TMEM179B 5
ZNF544 5
ERGIC3 5
COQ10A 5
E2F4 5
ACP2 5
PPIB 5
MCFD2 5
MEST 5
VMP1 5
NDUFS5 5
CLN5 5
PDCL2 5
TUBA3E 5
MLF2 5
RTN3 5
CHGB 5
YEATS4 5
GPAA1 5
EXT2 5
LPCAT3 5
HSPA2 5
MYL6 5
BLOC1S6 5
HELLS 5
PCOLCE 5
BLOC1S2 5
DNMT1 5
C11orf73 5
TIPIN 5
EFCAB2 5
SSR2 5
IQCB1 5
ATF6B 5
CT45A10 5
CUTC 5
DNPH1 5
VBP1 5

MED31 5
GINM1 5
AUP1 5
SMDT1 5
CCNDBP1 5
BRD8 5
PAIP1 5
FAM122A 5
NMRAL1 5
WRB 5
CKB 5
PRIM1 5
KRIT1 5
FAM98C 5
RAB1A 5
HMCES 5
NT5C 5
ZNF593 5
ACTL8 5
RIC8A 5
DPH2 5
YIPF6 5
M6PR 5
SOHLH2 5
TUSC3 5
SPEN 5
CSPP1 5
EIF2AK1 5
PGRMC1 5
H2AFY 5
U82695.5 5
RBM44 5
SMIM20 5
RBM28 5
TMX2 5
APMAP 5
STMND1 5
SLC39A1 5
PRPSAP2 5
NCSTN 5
ZNF280C 5
METTL10 5
YDJC 5
TBL2 5
TMEM167A 5
TMEM107 5
DR1 5
DCK 5
UGT8 5
SEC22C 5
CTSA 5
TRAPPC1 5
C9orf142 5
RAB30-AS1 5
TFB2M 5
RECK 5

SSSCA1 5
AC240274.1 5
NUS1 5
HIBADH 5
PRMT5 5
TCTN3 5
R3HDM2 5
NMT2 5
LRRCC1 5
POLR2D 5
MEI1 5
QTRT1 5
RBFA 5
CD46 5
IDH1 5
C3orf33 5
BFAR 5
BET1 5
ST3GAL5 5
ANKRD54 5
NOP16 5
SPDL1 5
MNAT1 5
OS9 5
CCDC24 5
FANCA 5
NARS 5
NPC1 5
NRIP3 5
CDC6 5
ECT2 5
NAT10 5
SKA2 5
NUCB1 5
ZNF800 5
MDK 5
ACRC 5
HSPA1A 5
UHRF1BP1L 5
TCTN1 5
LYRM2 5
TEX11 5
MPDU1 5
PSPH 5
TMEM30A 5
FBXO3 5
FCGRT 5
CEP135 5
ANKRD39 5
DCAF4 5
GINS1 5
DLEU2 5
SYCE2 5
SLBP 5
TUBA3C 5
HMGB2 5

APRT 5
NDUFA11 5
TXN 5
MAD2L1 5
NIFK 5
MRPL15 5
MESDC2 5
APP 5
IFT22 5
ORC6 5
SUV39H2 5
RNASEH2A 5
NOC3L 5
CD3EAP 5
FAM96A 5
IDI1 5
DERL3 5
DBI 5
TIGAR 5
RCL1 5
UBE2Q2 5
STARD7 5
TUBA1C 5
RHNO1 5
C20orf27 5
DPP7 5
SMC6 5
H2AFX 5
LARP1 5
CERCAM 5
ENOSF1 5
ARL6IP1 5
CBY1 5
PPP5C 5
DFFA 5
SRRM2-AS1 5
MAGOHB 5
DCPS 5
PCNP 5
CCPG1 5
TSR1 5
MAT2A 5
DNAAF5 5
RAD54L2 5
NEK4 5
ACAT1 5
CNOT6 5
ATPAF1 5
FGFR4 5
PPT1 5
FBXW2 5
C15orf48 6
TEX19 6
PEG10 6
C5orf47 6
SCML1 6

STRA8 6
KIF5B 6
CAPZA1 6
EMC7 6
MMP25-AS1 6
F12 6
CCDC73 6
ETV7 6
TMEM50B 6
RP11-890B15.2 6
C12orf65 6
ACTR3 6
CYSTM1 6
CUTA 6
SELK 6
PIGK 6
CGREF1 6
DMKN 6
CYP20A1 6
ZRANB2 6
AMN 6
DNAJB9 6
RNF34 6
VPS36 6
FUOM 6
SPRYD7 6
POP5 6
CNTRL 6
RP5-1139I1.1 6
GDE1 6
CLDN10 6
NFE2L1 6
RP11-366H4.1 6
ZNF75A 6
C10orf35 6
GTF2H2 6
ARL1 6
ORMDL1 6
CEP290 6
LMNA 6
NAA20 6
OCIAD1 6
SEC11C 6
ZNF232 6
MFSD4 6
EIF2S1 6
C11orf85 6
RP11-53B5.1 6
OAZ2 6
LTBP4 6
STRIP2 6
TM7SF2 6
KDELR1 6
FES 6
KCNQ1OT1 6
COMMD8 6

STK25 6
PSORS1C1 6
CTSL 6
FUCA1 6
ANKRD36C 6
H1F0 6
GIGYF2 6
ACADVL 6
TOP2B 6
REEP5 6
FOXD3-AS1 6
NPTX2 6
SCRN2 6
YIPF4 6
MRPL11 6
ZNF91 6
ARHGAP9 6
LARS 6
CHRNE 6
MYADM 6
C1orf228 6
SPCS3 6
MTFR1 6
TMEM242 6
LYRM9 6
GGCT 6
HOOK2 6
UFL1 6
THAP7 6
FLYWCH2 6
UNC50 6
JADE3 6
GALNT11 6
LY6K 6
ACBD3 6
PFDN6 6
TMEM141 6
SDCBP2 6
FAM208B 6
CISD3 6
ANKRD31 6
RNF141 6
PSMD5-AS1 6
C4orf27 6
CHMP4A 6
FAM216A 6
GEMIN2 6
MRC2 6
NUDT17 6
GNPDA1 6
PDE6D 6
RNFT1 6
OTUD6A 6
GABPB2 6
TP53I3 6
ARHGAP5 6

TMEM80 6
GPAT2 6
PKN2 6
ATP5G1 6
TAF11 6
HSD17B14 6
TSPYL2 6
HAUS5 6
FPGS 6
IARS 6
SEMA3B 6
PPIC 6
CNPY3 6
C1orf146 6
ITPA 6
SFRP5 6
XX-CR54.1 6
NOL8 6
TRAM1 6
CCDC47 6
TMC7 6
SMIM4 6
PIK3IP1 6
SLMAP 6
TMEM216 6
ITM2C 6
TIMM22 6
PDIK1L 6
PROCR 6
SLC39A6 6
C14orf1 6
LTBP3 6
TIPRL 6
TPST1 6
CT47B1 6
VWA9 6
SLC25A4 6
EPRS 6
RDH11 6
THOC7 6
STEAP1 6
HINT3 6
NEURL1 6
ZCCHC9 6
TTC26 6
SLC30A5 6
UBA5 6
GOPC 6
DNAAF3 6
CES4A 6
RP11-620J15.3 6
GEMIN6 6
NUDT9 6
C11orf70 6
IFT81 6
MCM3AP-AS1 6

ISY1 6
FBXO6 6
NT5C3A 6
C19orf57 6
EIF2B1 6
CES2 6
TMX4 6
PXYLP1 6
CCDC152 6
MAP7 6
ANKRD36 6
LRRC41 6
KMT5C 6
JMJD8 6
PLA2G12A 6
CISD2 6
MYBBP1A 6
SFR1 6
FADS1 6
AARS 6
TINF2 6
ATP6AP2 6
MAGEA10 6
GPR160 6
RP11-69G7.1 6
RBM6 6
METTL14 6
FXN 6
TMED7 6
TUBB8 6
FAM9A 6
RHBDD3 6
EP400 6
RALGAPA1 6
SNX13 6
RPAP3 6
GTF2H3 6
PDHA1 6
TMEM52 6
SBF2-AS1 6
DHRSX 6
TACC1 6
B9D1 6
DHRS4-AS1 6
MKRN2 6
NFKBIE 6
MED7 6
HTRA2 6
FAM157C 6
ASH1L 6
C17orf64 6
CREG1 6
MMP9 6
GGT7 6
ATG16L2 6
TWISTNB 6

CCDC79 6
FOXRED2 6
CCDC113 6
VARS 6
FNDC3A 6
SDR39U1 6
AK3 6
HS1BP3 6
DMC1 6
ZNF559 6
CDK18 6
ZFC3H1 6
C8orf88 6
RPAIN 6
PDPK1 6
FXYD1 6
RILP 6
SYTL1 6
UTP23 6
SENP1 6
SYCP2 6
ATG9B 6
ZNF17 6
KLRG1 6
VEZT 6
YTHDC2 6
C5orf15 6
KIAA0100 6
HCFC2 6
KMT2A 6
CRELD1 6
NUMB 6
KCTD9 6
C17orf75 6
IFNAR1 6
TMEM33 6
LLNLR-245B6.1 6
TMEM206 6
LPGAT1 6
KIDINS220 6
KLHDC9 6
RP11-78J21.4 6
ZFP62 6
HSDL2 6
ERLIN2 6
MFSD3 6
MYT1 6
GNPTG 6
SLC7A6 6
RP11-181C3.1 6
SUMF2 6
DHX34 6
SORL1 6
SH3YL1 6
RRN3 6
PGAP2 6

PROSER1 6
MESP1 6
URB1 6
FAM9C 6
MBIP 6
SVIL-AS1 6
PGM2L1 6
RNGTT 6
ARMT1 6
BSCL2 6
CRY1 6
ACO1 6
POU2F2 6
FAM219B 6
LINC00667 6
FAAH 6
C18orf25 6
RP11-152N13.5 6
UBE3D 6
TRAFD1 6
C7orf49 6
ZNF883 6
DNAAF2 6
ZPBP 6
SMIM13 6
TUBB2A 6
RC3H2 6
POLK 6
SLC11A2 6
ZNF398 6
DZIP1 6
DPF1 6
HPCAL1 6
METTL17 6
TCHH 6
TMUB1 6
DSEL 6
ABCC5 6
FAM41C 6
AKAP1 6
TMCO6 6
EFCAB13 6
CBWD2 6
STEAP1B 6
ZBED5 6
RALGPS2 6
EYA3 6
RANBP6 6
SPOPL 6
RBM26-AS1 6
NEAT1 6
KIAA1211L 6
ATPAF2 6
CCDC136 6
ETNK1 6
CMB9-22P13.1 6

PTGR2 6
MIS12 6
TMEM138 6
STT3A 6
CLUH 6
SOX15 6
CMTM3 6
SPIRE1 6
ZFAND1 6
KLHL22 6
CAAP1 6
ARHGEF10 6
GCHFR 6
UTP15 6
PGGT1B 6
TM7SF3 6
BORCS8 6
DPY19L4 6
MTMR6 6
NUP43 6
MAFG-AS1 6
MFN2 6
TOP1 6
STAG3 6
PUS1 6
COQ2 6
PI4K2B 6
ZNF326 6
EPB41L4B 6
CEP164 6
RNF103 6
SLC27A5 6
SLC5A5 6
SRSF1 6
ZDHHC13 6
ICE1 6
RAB11FIP1 6
SAMD8 6
NET1 6
DIMT1 6
CDK8 6
INTS5 6
RP13-1032I1.7 6
LMAN2L 6
SEC24B 6
ZNF551 6
TMEM234 6
PEX3 6
COQ3 6
CEP41 6
PCGF6 6
FCHO2 6
NECAP1 6
OSTM1 6
LIG3 6
PXN-AS1 6

SNHG25 6
ZNF146 6
TM9SF1 6
ZNF791 6
